# Polyacetylenes with (hetero)aryl-, styryl-, and amino-phenothiazinyl sidechains: synthesis and photophysics†

**DOI:** 10.1039/d4ra01912a

**Published:** 2024-04-02

**Authors:** Wladislaw Pisetsky, Thomas J. J. Müller

**Affiliations:** a Heinrich Heine University Düsseldorf, Faculty of Mathematics and Natural Sciences, Institute of Organic Chemistry and Macromolecular Chemistry Universitätsstrasse 1 D-40225 Düsseldorf Germany ThomasJJ.Mueller@uni-duesseldorf.de

## Abstract

A novel generation of 7-aryl phenothiazinyl substituted polyacetylenes is readily accessible *via* controlled rhodium-catalyzed polymerization of the corresponding 3-ethynyl 7-aryl phenothiazines. The monomers are synthesized by Suzuki coupling, Heck coupling, or Buchwald–Hartwig amination, and Bestmann–Ohira reaction. This allows for the introduction of electron donating and releasing substituents with different ligation patterns. The obtained polymers display narrow molecular weight distributions, with very few exceptions, and are soluble in many organic solvents. The photophysical properties of novel monosubstituted polyacetylenes and corresponding monomers were compared. While the monomers exhibit strong emission in solution with quantum yields of up to 0.84 only selected polymers are luminescent (*Φ*_f_ = 0.06) and display moderate Stokes shifts and positive emission solvatochromism.

## Abstract 2

A new series of soluble monosubstituted polyacetylenes bearing phenothiazines units with various ligation patterns is readily available by modern rhodium-catalyzed polymerization of corresponding ethynyl-phenothiazines.

## Graphical abstract

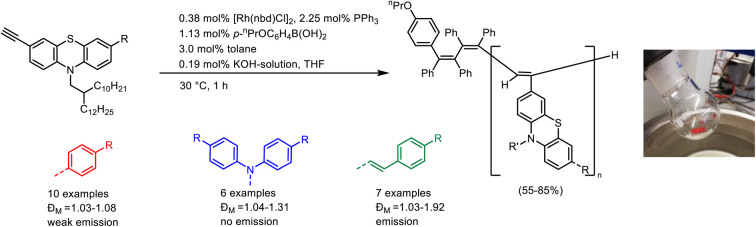

## Introduction

In recent years the venerable electron-rich phenothiazine heterocycles have stimulated increasing scientific interest and have been successfully established in organic electronics, such as organic light-emitting diodes (OLEDs), organic field-effect transistors (OFETs), dye-sensitized solar cells (DSSCs), molecular wires and organic hole-transport layers.^1–3^ Due to their low reversible oxidation potential they qualify as good donor molecules, and butterfly shaped phenothiazines can be easily oxidized to give stable planar colorful radical cations.^4^ The optical and electrochemical properties of phenothiazines can be tuned by introducing substituents in 3- and 7-position using cross-coupling reactions, such as Suzuki and Sonogashira coupling.^5–7^ As previously shown by our group, the first oxidation potentials of phenothiazines can be lowered using monodisperse oligophenothiazines that are accessible *via* one-pot bromine–lithium exchange-borylation–Suzuki (BLEBS) coupling.^8^ Cyclic voltammetry revealed an electronic intramolecular communication between the phenothiazine units, which can be affected by integration of covalently bound organometallic communicators, such as ferrocene.^9^ The self-assembly characteristics of oligophenothiazines bearing thioacetate groups were successfully achieved by chemisorbing the moieties on gold surfaces.^10,11^ However, solubility issues impeded the applicability of early generations of oligophenothiazines.^12,13^

Therefore, we became mesmerized to generate a novel conjugated organic polymer, uniting the interesting semiconductivity of polyacetylene with the light emission of the electron-rich heterocycle of phenothiazine. Among the conjugated polymers especially the archetypal polyacetylene and novel generations of mono- and disubstituted polyacetylenes received scientific interest, showing broad applications as devices in, for instance, xerography and photovoltaic cells.^14–17^

Very recently, we synthesized and characterized a series of novel emissive phenothiazinyl merocyanine substituted polyacetylenes accessible *via* rhodium catalysis following Taniguchi's protocol.^18,19^ Indeed, the photonic properties of phenothiazinyl merocyanines are transferable into the corresponding polymers. As monosubstituted polyacetylenes are typically regarded to be non-emissive due to unfavorable energetics of ground and excited states these results were quite surprising.^20^ In addition, well-known solubility issues of polyacetylenes could be overcome by introducing a branched aliphatic chain.

Here, we focus on the synthesis of (oligo)phenothiazinyl substituted polyacetylenes bearing electron withdrawing and donating groups with different ligation patterns for investigating the structure–property relationships.

## Results and discussion

### Monomer synthesis and polymerization

**Scheme 1:**
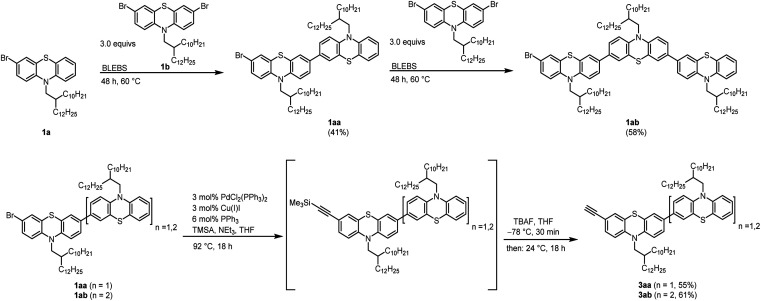
Synthesis of unsymmetrical oligophenothiazines 1aa–ab*via* BLEBS sequence and following synthesis of alkynylated oligophenothiazines 3aa–3ab by Sonogashira-coupling-desilylation-sequence.

The synthesis of an asymmetric alkynylated phenothiazine dimer and trimer was started with a one-pot BLEBS sequence of the mono brominated 1a and dibrominated phenothiazine 1b moieties according to our established protocol.^8^ In order to overcome well-known solubility issues, a branched alkyl substituent was introduced by *N*-alkylation of the phenothiazine moiety.^12^ The dibrominated phenothiazine 1b was used in excess to minimize the formation of unfavorable symmetrical oligophenothiazines *via* double BLEBS sequence (Scheme 1).

The unsymmetrical brominated phenothiazines 1aa and 1ab were transferred into the corresponding alkynylated moieties 3aa and 3ab in a one-pot fashion by Sonogashira-coupling-desilylation-sequence using (trimethylsilyl)acetylene (TMSA) as a coupling reagent and tetrabutylammonium fluoride (TBAF) to deprotect the TMS group.^19^

**Scheme 2:**
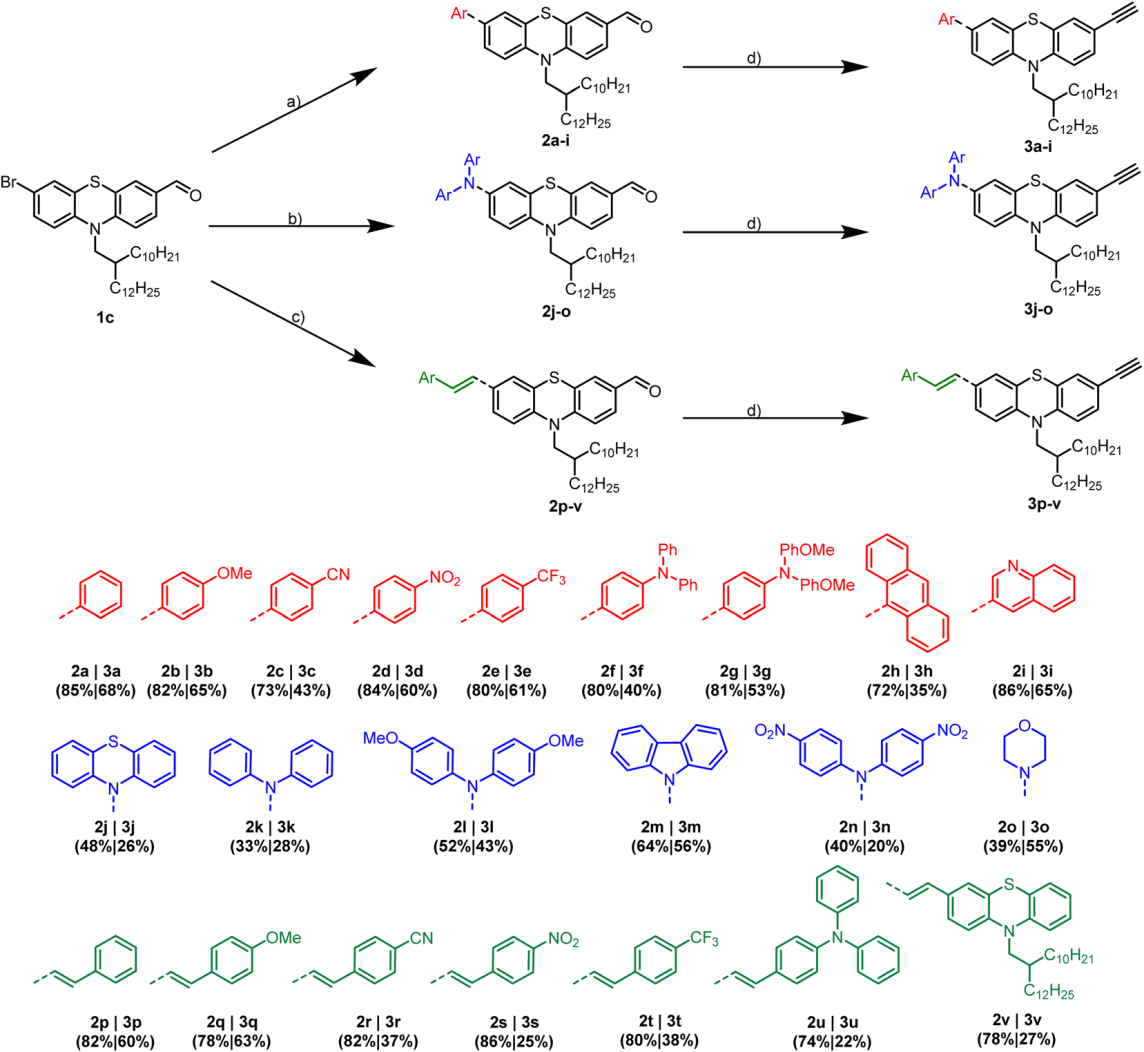
Synthesis of 3-substituted phenothiazinyl aldehydes 2a–v. (a) [Pd(PPh_3_)_4_], K_2_CO_3_, DME/H_2_O, 115 °C, 16 h; (b) Pd(dba)_2_, NaO^*t*^Bu, [(*t*-Bu)_3_PH]BF_4_, 1,4-dioxane, 110 °C, 18–48 h; or: Cu powder, 18-crown-6, K_2_CO_3_, *o*-DCB, 180 °C, 48 h; (c) Pd_2_(dba)_3_, NaHCO_3_, cataCXium PtB, DMF, 140 °C, 16 h and of alkynylated phenothiazines 3a–v (d) Bestmann–Ohira reagent, K_2_CO_3_, MeOH, THF, room temp, 16 h.

The preparation of 3-substituted alkynylated phenothiazines 3 commences from bifunctional 3-bromo-7-formyl phenothiazine 1c as starting material (Scheme 2). As recently published, 1c is readily accessible by *N*-alkylation of 10*H*-phenothiazine with a branched swallowtail, followed by two-step Vilsmeier formylation–bromination.^20^ Since cross-coupling reactions are a convenient method to generate libraries of substituted phenothiazines with various ligation patterns, we exploit the reactivity of phenothiazines and introduce substituents in 3- and 7-positions of the phenothiazine unit.

In the first step, electron donating and withdrawing aryl-substituents are introduced *via* Suzuki coupling using Pd(PPh_3_)_4_ and K_2_CO_3_ following our protocol^5^ to give arylated products 2a–i in good yields (71–82%).

The donor strength of the substituents can be increased by coupling of secondary amines *via* Buchwald–Hartwig arylamination of 1c.^21^ The corresponding phenothiazines 2j–o are obtained in moderate yields (33–64%). Selective secondary amines are synthesized *via* Ullmann coupling since Buchwald–Hartwig arylamination is unsuccessful. Especially, electron-poor secondary amines are quite unreactive (see ESI†). Regarding the morpholino substituted derivative 2o, an l-proline catalyzed variation of Ullmann coupling is required to obtain the desired product.

Furthermore, the π-system of the phenothiazine moieties can be extended by Heck coupling of substituted styrenes with 1c in the presence of Pd_2_dba_3_ and cataCXium® PtB as a ligand.^7^ The vinylated compounds 2p–v are isolated in good yields (74–82%). Finally, Bestmann–Ohira reaction of 2a–v furnishes alkynylated phenothiazines 3a–v in moderate yields, following mild conditions using a methanol–water mixture as a solvent at room temperature.^6^

The structures of the novel aldehydes 2a–v and monomers 3a–v are unambiguously characterized by ^1^H and ^13^C NMR, IR spectroscopy and mass spectrometry. The purity of the bulk material is supported by combustion analysis.

**Scheme 3:**
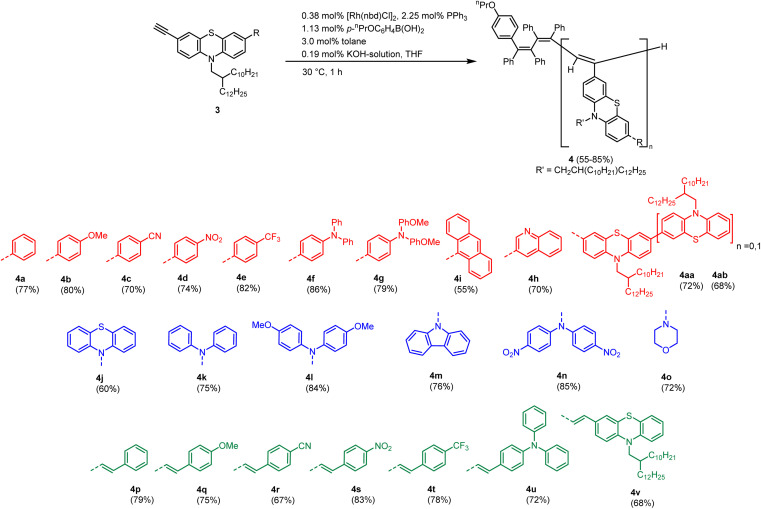
Synthesis of phenothiazinyl-substituted polymers 4 by rhodium-catalyzed polymerization of 3-alkynyl phenothiazines.

The polymerization of the substituted alkynylated phenothiazines 3a–v and 3aa–3ab is accomplished following the recently optimized protocol by Taniguchi *via* rhodium catalysis (Scheme 3).^18^ In contrast to monomers, which are isolated as colorful oils, the corresponding polymers are precipitated in methanol furnishing orange solids. Interestingly, the obtained polymers 4 show different morphologies, including rubbers, powders, and brittle glasses.

The novel polymers 4 are characterized using gel permeation chromatography (GPC), IR spectroscopy, UV-vis absorption and emission spectroscopy. As already observed for parent polyacetylenes, NMR spectra reveal considerably broadened signals hampering a proper structural characterization.^19^

The solubility-promoting 2-decyl-tetradecyl *N*-substituent on the phenothiazine allows for characterization of all polymers except for anthracenyl substituted polymer 4i, which is completely insoluble. The polymerization data are investigated using GPC measurements.

As already observed for parent polymers, the polydispersities of phenothiazinyl substituted polyacetylenes are strongly dependent on the substitution pattern at position 7 of the phenothiazine moiety. Additionally, GPC data reveal an additional dependence on the ligation pattern. In contrast to recently published phenothiazinyl merocyanine substituted polyacetylenes,^19^ the obtained polymers generally show narrow weight distributions, despite applying same reaction conditions. On the one hand, aryl-substituted polymers 4a–h and amino-substituted polymers 4j–o show low dispersities (1.04–1.13) and high molecular weights, hinting at the living nature of this rhodium catalyzed polymerization (Table 1). On the other hand, vinyl substituted polymers 4p–4v show wider spread of molecular weights and broad dispersities. Noteworthy, nitro substituted derivatives 4d, 4n and 4s display significantly broader molecular weight distributions and lower molecular weights, which might be rationalized by interchain charge-transfer interactions of the electron-rich phenothiazinyl moieties with electron-deficient nitrostyryl parts.

**Table 1:** Molecular weight distributions of the polymers 4 and of corresponding unsubstituted pendant 4w (R = H) determined from GPC traces (in THF, flow rate 1 mL min^−1^, *T* = 293 K)

Polymer	*M*_w_ [kDa]	*M*_n_ [kDa]	*Đ*_M_	Polymer	*M*_w_ [kDa]	*M*_n_ [kDa]	*Đ*_M_
4a	205	188	1.11	4l	25.9	23.8	1.09
4b	106	103	1.06	4m	9.82	9.28	1.08
4c	138	133	1.04	4n	92.3	70.2	1.31
4d	1227	859	1.43	4o	34.9	32.7	1.06
4e	140	135	1.04	4p	68.2	66.2	1.03
4f	311	294	1.05	4q	300	156	1.92
4g	107	103	1.06	4r	392	201	1.95
4h	285	263	1.08	4s	187	11.8	15.8
4i	—	—	—	4t	93.2	90.0	1.03
4aa	107	97.1	1.11	4u	109	85.5	1.28
4ab	76.2	67.1	1.19	4v	57.7	57.8	1.01
4j	18.8	17.6	1.06	4w^19^	60.0	55.8	1.05
4k	12.3	11.2	1.13				

An additional trend can be observed regarding the oligophenothiazine polymers 4aa–4ab. In this case, longer sidechains clearly lead to broader molecular weight distributions. Comparing triad 4ab to unsubstituted pendant 4w (R = H),^19^ significantly higher polydispersities become evident indicating a low control of the polymerization (see Table 1).

### Photophysical properties

The photophysical properties of monomers 3 and the corresponding polymers 4 were studied *via* UV-vis and fluorescence spectroscopy. All spectra were recorded in dichloromethane since significant emission was observed in polar solvents.

Expectedly, alkynylated phenothiazines 3 show typical photonic properties for phenothiazines. Longest wavelength absorption maxima *λ*_max,abs_ are between 334–431 nm including additional absorption bands in UV region caused by π–π* transition. Obviously, the absorption behavior is influenced by the electronic properties of the aryl substituents. A redshift of the longest wavelength absorption maxima within each series of monomers was observed for derivatives carrying electron withdrawing substituents. Therefore, the absorption maximum of nitro-substituted derivative 3n lies at 406 nm in contrast to donor substituted phenothiazine 3j bearing an electron-donating phenothiazine group (328 nm). Further regularities were observed comparing aryl substituted moieties 3a–i and corresponding vinyl substituted derivatives 3p–v. The extension of the π-system causes a redshifted absorption from 334 to 374 nm. The same trend can be seen with phenothiazine dyad 3aa and triad 3ab.

Except for nitro-substituted derivatives, monomers 3 display a pronounced emission in dichloromethane. The emission maxima lie between 466 and 570 nm. Again, the strongest redshift in emission is recorded for the vinylated acceptor substituted derivative 3r. Within the series, highest fluorescence quantum yields are detected for 3c (0.76), 3e (0.69), 3r (0.87) and 3t (0.84), bearing electron withdrawing cyano and trifluoromethyl substituents.

**Fig. 1:**
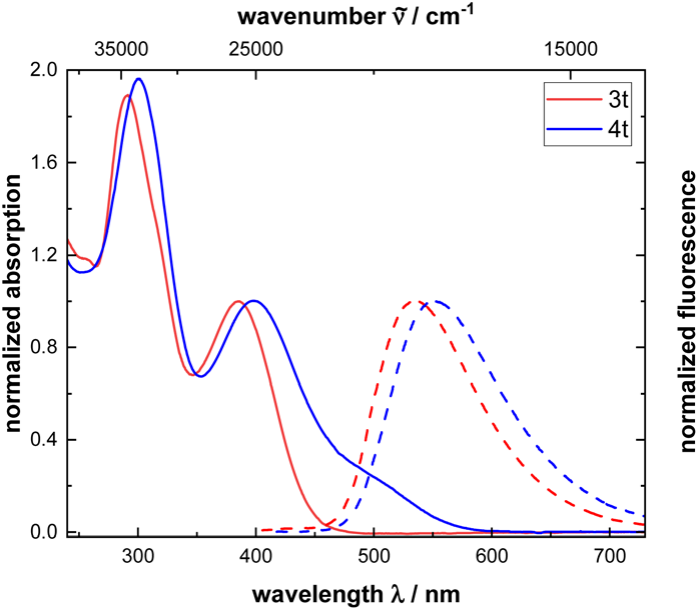
UV-vis absorption and emission spectra of monomer 3t and polymer 4t recorded in dichloromethane (*c*(3t,4t) = 10^−5^ M, *T* = 293 K).

The optical properties of monomers 3 can be compared to the corresponding polymers 4. Generally, the absorption and emission spectra of all monomers and polymers show a similar shape. However, for the polymers 4 additional bands are observed at 500 nm, caused by the absorption of the polyene backbone. Noteworthy, the band appears as a broad shoulder. The remaining absorption maxima of polymers 4 coincide with the corresponding monomers since optical properties are determined by the phenothiazine moiety. A juxtaposition of monomer 3t and polymer 4t shows a redshifted absorption and emission. In contrast to alkynylated phenothiazines 3 and phenothiazinyl merocyanine substituted polyacetylenes (Fig. 1 and Table 2),^19^ polymers 4 are mostly weakly emissive (see ESI, Table S9†). Emission spectra are recorded, but the quantum yields are not determined since emission is very weak. Noteworthy, to measure the emission spectra of the polymers 4, excitation is not achieved at the longest wavelength absorption band (shoulder at around 500 nm), as usually, but at the next hypsochromically shifted maximum.

**Table 2:** Photophysical data of monomer 3t and polymer 4t recorded in dichloromethane (*c*(3t,4t) = 10^−5^ M, *T* = 293 K)

	*λ*_max,abs_ [nm] (*ε* [L mol^−1^ cm^−1^])	*λ*_max,em_ [nm]	*Φ*_F_	Stokes shift Δ*ν̃*^a^ [cm^−1^]
3t	385 (18 600), 292 (35 500)	535	0.84	7300
4t	500 (7 041 500), 399 (29 152 800), 301 (56 996 000)	551	0.06	6900

a Δ*ν̃* = 1/*λ*_max,abs_ – 1/*λ*_max,em_ [cm^–1^].

**Fig. 2:**
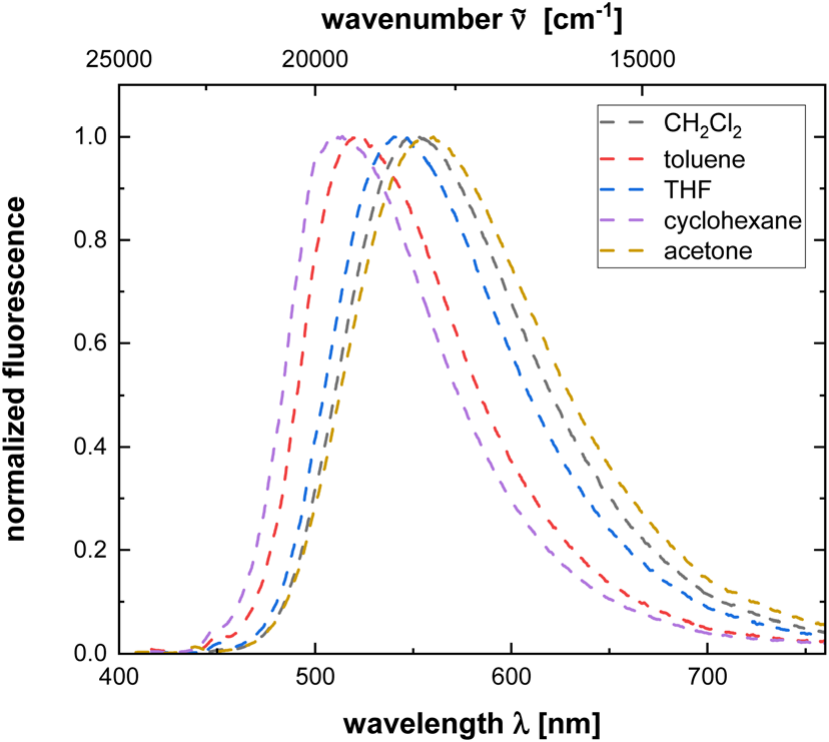
Emission spectra of polymer 4t recorded in solvents of different polarity (*c*(4t) = 10^−5^ M, *T* = 293 K).

Exceptionally polymers 4r and 4t show a moderate emission in dichloromethane with fluorescence quantum yields of 0.06, however quantum yields are significantly lower than corresponding monomers. Moreover, both polymers exhibit pronounced polarity effects including positive emission solvatochromism (see Fig. 2 and Table 3). The lower fluorescence quantum yield in comparison to phenothiazinyl merocyanine substituted polyacetylenes^19^ and the lower energy of the longest wavelength absorption maxima of these series indicate that the sidechain chromophore only contributes to an emission of the polymer, if the excitation energy stems from the appended fluorophore moieties.

**Table 3:** Photophysical data of polymer 4t recorded in solvents of different polarity (*c*(4t) = 10^−5^ M, *T* = 293 K)

Solvent	*λ*_max,em_ [nm]	Stokes shift Δ*ν̃* [cm^−1^]
Cyclohexane	514	5800
Toluene	522	6000
THF	543	6700
Dichloromethane	551	6900
Acetone	558	7700

## Conclusion

In summary, we have successfully extended the recently published library of novel soluble phenothiazinyl substituted polyacetylenes obtained *via* rhodium catalysis and analyzed their structure–property relationship. Three series of substituted alkynyl phenothiazines bearing electron withdrawing and donating substituents with different ligation patterns were synthesized applying Suzuki coupling, Buchwald–Hartwig arylamination, Heck coupling and Bestmann–Ohira reaction. In contrast to parent polyacetylenes, most obtained polymers display narrow molecular weight distributions and low dispersities supporting living nature of polymerization. The photophysical properties of obtained alkynylated phenothiazines were compared to corresponding polymers. Contrary to highly emissive monomers, especially donor substituted polyacetylenes can be regarded as non-emissive. Among the series, only two polymers show moderate emission in dichloromethane (0.06), including emission solvatochromism.

## Conflicts of interest

There are no conflicts to declare.

## Supplementary Material

RA-014-D4RA01912A-s001
