# Supplementary material for: Polyacetylenes with (hetero)aryl-, styryl-, and amino-phenothiazinyl sidechains: synthesis and photophysics
Source: RSC Adv. 2024 Apr 2;14(15):10638–43. doi: 10.1039/d4ra01912a (PMC10985593; doi:10.1039/d4ra01912a)
Supplement: RA-014-D4RA01912A-s001 [file RA-014-D4RA01912A-s001.pdf]

Electronic Supplementary Material (ESI) for RSC Advances.
This journal is © The Royal Society of Chemistry 2024

**Polyacetylenes with (Hetero)Aryl-, Styryl-, and Amino-Phenothiazinyl Sidechains – Synthesis and Photophysics**

Wladislaw Pisetsky [a] and Thomas J. J. Müller*[a]

Heinrich Heine University Düsseldorf, Faculty of Mathematics and Natural Sciences, Institute of Organic Chemistry and Macromolecular Chemistry, Universitätsstrasse 1, D-40225 Düsseldorf, Germany

E-mail: ThomasJJ.Mueller@uni-duesseldorf.de

## Table of contents

1. General considerations ..... 2

2.0 Syntheses ..... 4

2.1 Syntheses and analytical data of precursors and monomers ..... 4

2.1.1 General procedure I (GP1) for the synthesis of the mono-brominated phenothiazine dyad and triad via BLEBS sequence ..... 4

2.1.2 General procedure II (GP2) for the Suzuki coupling ..... 8

2.1.3 General procedure III (GP3) for the Buchwald-Hartwig arylamination ..... 19

2.1.4 General procedure IV (GP4) for the Ullmann coupling ..... 22

4.2.1 General procedure V (GP5) for the *Heck*-coupling ..... 26

2.2 Synthesis and analytical data of the monomers **3** ..... 35

2.2.1 General procedure VI (GP6) for the Ohira-Bestmann reaction ..... 35

2.2.2 General procedure VII (GP7) for the Sonogashira coupling ..... 59

2.3 Synthesis and analytical data of the polymers ..... 63

2.3.1 General procedure VIII (GP8) for the polymerization of the alkynylated Phenothiazines ..... 63

3.0 UV/Vis absorption and emission spectra of monomers **3** and polymers **4**. ..... 80

4.0 IR spectra ..... 109

5.0 GPC traces of polymers **4** ..... 136

6.0 NMR spectra ..... 150

7.0 References ..... 197

# 1. General considerations

All reactions in this work were carried out under inert conditions (nitrogen atmosphere), unless otherwise stated. For this purpose, the heated and sintered Schlenk tubes or single/multi-neck flasks used as reaction vessels were filled via Schlenk and cannula techniques. Dry tetrahydrofuran, 1,4-dioxane and dichloromethane were obtained by using *MB-SPS-800* of *MBraun Inertgas-Systeme GmbH*. Triethylamine was dried via distillation and stored in a Schlenk flask under nitrogen atmosphere over KOH.

For column chromatography, silica gel 60 (mesh 70 – 230, particle size 0.04 – 0.063 mm) from *Macherey-Nagel* or *Sigma-Aldrich* was used and the products were purified by flash technique at a positive pressure of 1.8 – 2.0 bar. All solvents for column chromatography have been distilled. The crude products were adsorbed on *Celite*® 545 from *Carl Roth GmbH*. For thin-layer chromatography (for monitoring the reaction progress), silica-coated precast aluminium foils $F_{254}$ from *Merck KGaA* were used. Detection was performed by using UV light (λ = 254 nm).

3-Bromo-10-(2-decyltetradecyl)-10*H*-phenothiazine **1a**[1], 3,7-dibromo-10-(2-decyltetradecyl)-10*H*-phenothiazine **1b**[1], 7-bromo-10-(2-decyltetradecyl)-10*H*-phenothiazine-3-carbaldehyde **1c**[2], 10-(2-decyltetradecyl)-10*H*-[3,10'-biphenothiazine]-7-carbaldehyde **2j**[3], 7-(9*H*-carbazol-9-yl)-10-(2-decyltetradecyl)-10H-phenothiazine-3-carbaldehyde **2m**[3] were synthesized according to literature procedures. All commercially available chemicals were purchased from *Acros*, *Merck*, *Alfa Aesar*, *VWR*, *Roth*, *Fluorochem* or taken from the inventory of group and used without further purification.

$^{1}$H, $^{13}$C and 135-DEPT NMR spectra were recorded using AV III 600 and AV III 300 instruments from *Bruker*. Deuterated acetone, dimethyl sulfoxide, and tetrahydrofuran served as solvents, and their resonance was calibrated as an internal standard ($^{1}$H NMR: acetone-d$_6$: $\delta$ = 2.05, DMSO-d$_6$: $\delta$ = 2.49, THF-d$_8$: $\delta$ = 1.73, 3.58; $^{13}$C NMR: acetone-d$_6$: $\delta$ = 29.8, DMSO-d$_6$: $\delta$ = 39.5, THF-d$_8$: $\delta$ = 25.37, 67.57). The spin multiplicities were abbreviated as followed: s (singlet), d (doublet), t (triplet), dd (doublet from doublet), dt (doublet from triplet), q (quartet), quint. (quintet) and m (multiplet). The assignment of quaternary carbon nuclei, methine, methylene, and methyl groups was made by 135-DEPT spectra. When describing $^{13}$C NMR spectra, primary carbon nuclei were abbreviated as $CH_3$, secondary as $CH_2$, tertiary as CH, and quaternary as $C_{quat}$. EI measurements were performed on the *Finnigan MAT 8200* instrument. ESI-HR mass spectra measured on the *UHR-QTOF maXis 4G* from *Bruker Daltronics*. MALDI spectra were measured using a *MALDI-TOF Ultraflex I* from *Bruker Daltronics*. IR spectra were recorded neat using a *Shimadzu IRAffinity-1* with ATR technique. The intensities of the IR absorption bands were abbreviated as s (strong), m (medium) and w (weak). Elemental

analyses were performed using *Perkin Elmer Series II Analyser 2400* and *Elementar vario MICRO CUBE* instruments at the Institute of Pharmaceutical and Medicinal Chemistry, Heinrich Heine University, Düsseldorf, Germany.

The UV/Vis spectra were recorded on a *UV/VIS/NIR Cary 4000* spectrometer from *Edinburgh Instruments*. The compounds were measured in high purity solvent ($CH_2Cl_2$, HPLC grade) at room temp in 1 cm quartz cuvettes from *Hellma GmbH*. The extinction coefficients were determined via Lambert-Beer's law. For this purpose, the absorption spectra of the compounds were recorded at five different concentrations (concentration series). The concentrations of the polymers have been calculated by the number average molecular weight $M_n$. The molar absorption coefficient was determined as the slope of the linear regression of an absorbance versus concentration plot. The fluorescence spectra were recorded on a calibrated fluorescence spectrometer from *Hitachi F-7000*. Fluorescence quantum yields were measured by integrating sphere using a *FS5* spectrofluorometer from *Edinburgh Instruments*. Additionally, fluorescence quantum yields were determined by relative method with Coumarin 153 and Nile Blue as references.

The number average molecular weight $M_n$, the weight average molecular weight $M_w$, and the polydispersity $M_w/M_n$ of the synthesized polymers were determined by gel permeation chromatography in THF with polystyrene as a standard using a *PSS SECurity SEC system*.

# 2.0 Syntheses

## 2.1 Syntheses and analytical data of precursors and monomers

### 2.1.1 General procedure I (GP1) for the synthesis of the mono-brominated phenothiazine dyad and triad via BLEBS sequence

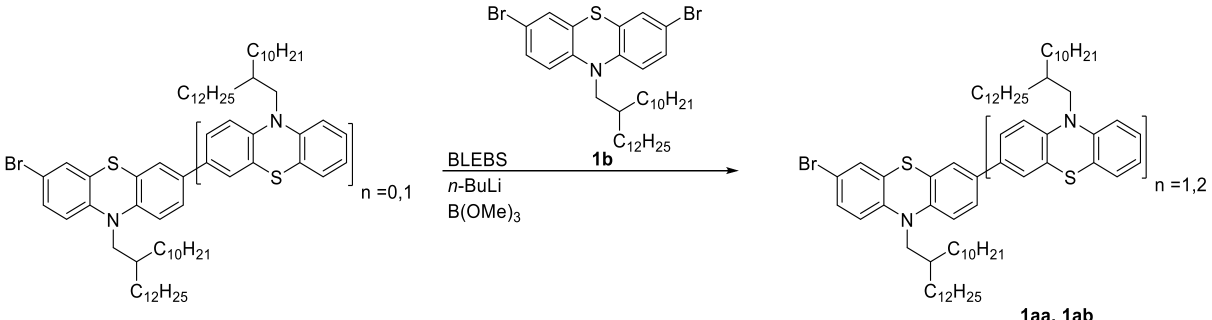


3-bromo-10-(2-decyltetradecyl)-10*H*-phenothiazine **1a** or **1aa** (1.0 equiv) was placed in a dry Schlenk tube under nitrogen atmosphere and dissolved in 3 mL/mmol dry THF. The solution was degassed for 15 min with nitrogen and cooled down to –78 °C by an acetone/dry ice bath for additional 15 min. Then, a 1M solution of *n*-BuLi in hexanes (1.1 equivs) was added via syringe. The yellow solution was stirred for 15 min at –78 °C. After addition of trimethylborate (1.15 equivs) the colorless solution was stirred for additional 15 min at –78 °C. After heating up to room temp tetrakis(triphenylphosphane)palladium(0) (5.0 mol%), KO$^t$Bu (1.1 equivs), and 3,7-dibromo-10-(2-decyltetradecyl)-10*H*-phenothiazine **1b** (3.0 equivs) were added under nitrogen atmosphere. The reaction mixture was stirred at 60 °C (oil bath) for 48 h. The crude product was transferred to a separating funnel. After addition of a saturated $Na_2SO_3$ solution the aqueous phase was extracted three times with diethyl ether. The organic phases were dried with anhydrous magnesium sulfate and filtered. The crude product was adsorbed on Celite® and purified via flash chromatography on silica gel.

**Table S1.** Experimental details for the synthesis of oligophenothiazines 1**aa-1ab**.

| entry | bromine | THF | n-BuLi | $B(OMe)_3$ | [Pd] | $KO^tBu$ | **1b** | Yields |
|---|---|---|---|---|---|---|---|---|
| 1 | 0.614 g (1 mmol) of **1a** | 3 mL | 0.75 mL (1.1 mmol) | 0.125 mL (1.15 mmol) | 57.7 mg (0.05 mmol) | 124.8 mg (1.1 mmol) | 2.07 g (3.0 mmol) | 0.476 g (41%) of **1aa** |
| 2 | 0.390 g (0.34 mmol) of **1aa** | 1mL | 0.26 mL (0.374 mmol) | 0.042 mL (0.391 mmol) | 19.6 mg (0.017 mmol) | 72.18 mg (0.374 mmol) | 0.693 g (1.0 mmol) | 0.330 g (58%) of **1ab** |

**7-Bromo-10,10'-bis(2-decyltetradecyl)-10*H*,10'*H*-3,3'-biphenothiazine (1aa)**

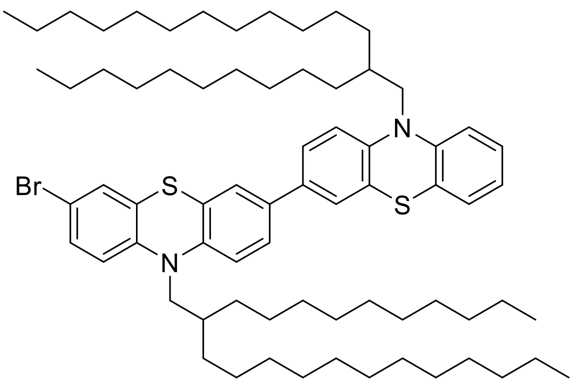


$C_{72}H_{111}BrN_2S_2$
[1148.72]

According to **GP1** and after flash chromatography on silica gel (*n*-hexane/dichloromethane 100:1) compound **1aa** (230 mg, 52%) was obtained as a yellow oil.

$\mathbf{R_f}$ (*n*-hexane/dichloromethane 100:1): 0.34.

**$^1$H NMR (300 MHz, THF-d$_8$):** $\delta$ 0.82 – 0.92 (m, 12H), 1.14-1.53 (m, 80H), 1.96 – 2.03 (m, 2H), 3.58 (s, 1H), 3.81 – 3.92 (m, 4H), 6.91 – 7.02 (m, 2H), 7.03 – 7.12 (m, 3H), 7.15 – 7.25 (m, 2H), 7.29 – 7.36 (m, 2H), 7.37 – 7.41 (m, 2H), 7.41 – 7.49 (m, 2H).

**$^{13}$C NMR (150 MHz, THF-d$_8$):** $\delta$ 14.5 (4 $CH_3$), 23.6 (2 $CH_2$), 25.6 (2 $CH_2$), 25.7 (4 $CH_2$), 25.8 (2 $CH_2$), 27.20 (2 $CH_2$), 27.23 (2 $CH_2$), 30.37 (2 $CH_2$), 30.48 (2 $CH_2$), 30.64 (2 $CH_2$), 30.67 (2 $CH_2$), 30.68 (2 $CH_2$), 30.7 (2 $CH_2$), 31.0 (2 $CH_2$), 32.5 (2 $CH_2$), 32.6 (2 $CH_2$), 32.9 (2 $CH_2$), 35.65 (CH), 35.67 (CH), 52.2 (2 $CH_2$), 52.3 (2 $CH_2$), 67.7 (2 $CH_2$), 67.8 (2 $CH_2$), 68.0 (2 $CH_2$), 115.06 ($C_{quat}$), 116.9 (CH), 117.1 (CH), 117.3 (CH), 118.3 (CH), 123.2 (CH), 125.9 (CH), 126.0 (2 CH), 126.2 (CH), 126.4 ($C_{quat}$), 126.5 ($C_{quat}$), 127.3 ($C_{quat}$), 128.0 (CH), 128.2 (CH), 128.9 ($C_{quat}$), 130.4 (CH), 130.77 (CH), 135.1 ($C_{quat}$), 135.8 ($C_{quat}$), 145.3 ($C_{quat}$), 145.9 ($C_{quat}$), 146.1 ($C_{quat}$), 146.7 ($C_{quat}$).

**MALDI-MS (m/*z*):** 1148.75 $[M]^+$.

**IR $\tilde{\nu}$ [cm$^{-1}$]:** 3061 (w), 2953 (w), 2920 (s), 2851 (m), 2679 (w), 1601 (w), 1578 (w), 1485 (w), 1452 (s), 1414 (w), 1377 (w), 1333 (w), 1250 (m), 1217 (w), 1159 (w), 1132 (w), 1107 (w), 1080 (w), 1063 (w), 1040 (w), 962 (w), 926 (w), 874 (w), 808 (s), 745 (s), 721 (w), 610 (w).

**Anal** calcd for $C_{72}H_{111}BrN_2S_2$ (1148.7): C 75.28, H 9.74, N 2.44, S 5.58; Found: C 74.99, H 9.55, N 2.36, S 5.39.

**7-Bromo-10,10',10''-tris(2-decyltetradecyl)-10*H*,10'*H*,10''*H*-[3,3',7',3'']-terphenothiazine (1ab)**

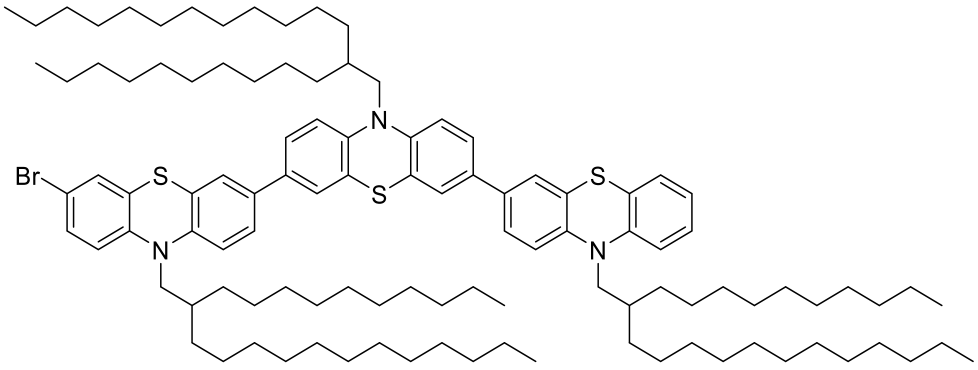

$C_{108}H_{166}BrN_3S_3$
[1682.62]

According to **GP1** and after flash chromatography on silica gel (*n*-hexane/dichloromethane 100:1) compound **1ab** (330 mg, 58%) was obtained as a yellow oil.

**$R_f$** (*n*-hexane/dichloromethane 100:5): 0.33.

**$^1$H NMR (300 MHz, acetone-d$_6$):** $\delta$ 0.85 – 0.95 (m, 18H), 1.23 – 1.50 (m, 120H), 1.97 – 2.02 (m, 1H), 3.80 – 3.90 (m, 6H), 6.90 – 6.96 (m, 2H), 6.97 – 7.05 (m, 5H), 7.12 – 7.21 (m, 2H), 7.25 – 7.32 (m, 2H), 7.32 – 7.43 (m, 8H).

**$^{13}$C NMR (75 MHz, acetone-d$_6$):** $\delta$ 14.7 (3CH$_3$), 14.8 (3CH$_3$), 23.6 (2 CH$_2$), 26.71 (CH$_2$), 26.73 (3CH$_2$), 27.1 (3CH$_2$), 27.2 (3CH$_2$), 30.3 (3CH), 30.4 (3CH) 32.3 (3CH$_2$), 32.4 (3CH$_2$), 32.8 (3CH$_2$), 35.4 (3CH$_2$), 52.1 (3CH$_2$), 115.0 (2CH), 115.2 (2CH), 117.2 (3CH), 117.5 (CH), 118.1 (CH), 123.3 (CH), 125.8 (CH), 126.0 (CH), 126.1 (CH), 126.26 (CH), 126.32 (CH), 127.0 (CH), 128.2 (3CH), 128.5 (2C$_{quat}$), 130.4 (2C$_{quat}$), 130.91 (2C$_{quat}$), 134.93 (2C$_{quat}$), 135.6 (C$_{quat}$), 144.98 (2C$_{quat}$), 145.42 (2C$_{quat}$), 146.1 (2C$_{quat}$), 146.5 (2C$_{quat}$).

**MALDI-MS (m/*z*):** 1682.2 [M]$^+$.

**IR $\tilde{\nu}$ [cm$^{-1}$]:** 3944 (w), 3923 (w), 3904 (w), 3854 (w), 3838 (w), 3478 (w), 3329 (w), 3119 (w), 2955 (w), 2920 (s), 2853 (m), 2369 (w), 2018 (w), 1456 (s), 1418 (w), 1377 (w), 1368 (w), 1339 (w), 1248 (m), 1234 (w), 1219 (w), 1184 (w), 1163 (w), 1152 (w), 1107 (w), 1072 (w), 1038 (w), 1026 (w), 966 (w), 893 (w), 876 (w), 808 (m), 745 (m), 719 (w).

**Anal** calcd for $C_{108}H_{166}BrN_3S_3$ (1682.6): C 77.09, H 9.94, N 2.50, S 5.72; Found: C 77.25, H 9.66, N 2.53, S 5.53.

### 2.1.2 General procedure II (GP2) for the Suzuki coupling

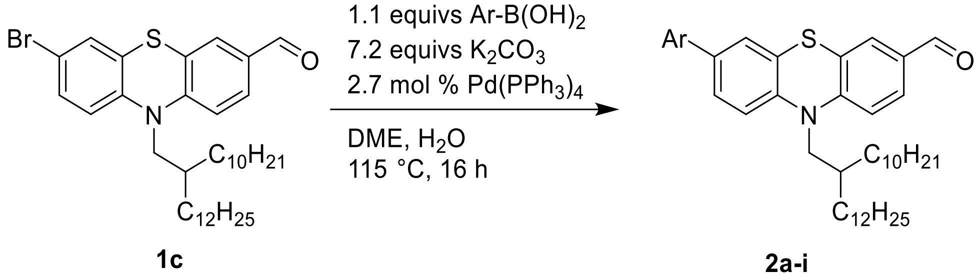


Aldehyde **1c (**1.0 equiv, 0.642 g, 1.0 mmol), the boronic acid (1.1 equivs, 1.1 mmol), $K_2CO_3$ (7.2 equivs, 0.995 g, 7.2 mmol) and tetrakis(triphenylphosphane)palladium(0) (0.031 g, 2.7 mol%) were placed in a dry Schlenk tube and dissolved in 2.7 mL water and 5.5 mL DME. The solution was degassed for 15 min with nitrogen and then stirred at 115 °C (oil bath) for 16 h. After addition of a saturated $Na_2SO_3$ solution the reaction mixture was transferred to a separating funnel. The aqueous phase was extracted three times with dichloromethane. The combined organic phases were dried with anhydrous magnesium sulfate and filtered. The crude product was adsorbed on Celite® and purified via flash chromatography on silica gel.

**Table S2.** Experimental details for the synthesis of phenothiazinyl aldehydes **2a-i**.

| entry | boronic acid | 1 |
|---|---|---|
| 1 | 0.134 g (1.1 mmol) phenylboronic acid | 0.525 g (82%) of **2a** |
| 2 | 0.167 g (1.1 mmol) 4-methoxyphenylboronic acid | 0.550 g (82%) of **2b** |
| 3 | 0.162 g (1.1 mmol) 4-cyanophenylboronic acid | 0.432 g (65%) of **2c** |
| 4 | 0.183 g (1.1 mmol) 4-nitrophenylboronic acid | 0.573 g (84%) of **2d** |
| 5 | 0.209 g (1.1 mmol) | 0.500 g (71%) of **2e** |

| | | |
|---|---|---|
| | 4-(trifluoromethyl) phenylboronic acid | |
| 6 | 0.318 g (1.1 mmol) 4-(diphenylamino) phenylboronic acid | 0.624 g (77%) of **2f** |
| 7 | 0.384 g (1.1 mmol) (4-(bis(4 methoxyphenyl)amino) phenyl)boronic acid | 0.710 g (82%) of **2g** |
| 8 | 0.244 g (1.1 mmol) 9-anthraceneboronic acid | 0.594 g (80%) of **2h** |
| 9 | 0.190 g (1.1 mmol) 3-quinolineboronic acid | 0.550 g (80%) of **2i** |

**10-(2-Decyltetradecyl)-7-phenyl-10*H*-phenothiazine-3-carbaldehyde (2a)**

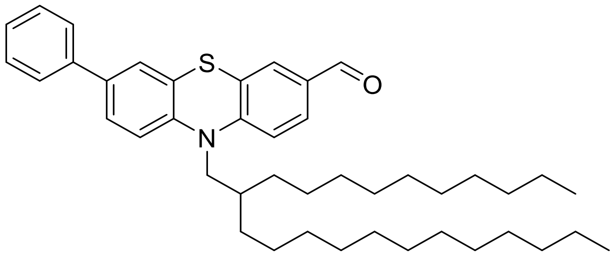

$C_{43}H_{61}NOS$
[640.03]

According to **GP2** and after flash chromatography on silica gel (*n*-hexane/diethyl ether 20:1) compound **2a** (525 mg, 82%) was obtained as a yellow oil.

**R**$_f$ (*n*-hexane/diethyl ether 30:1): 0.30

**$^1$H NMR (300 MHz, acetone-d$_6$):** $\delta$ 0.79 – 0.94 (m, 6H), 1.19 – 1.51 (m, 40H), 2.07 – 2.10 (m, 1H), 4.00 (d, $^3J$ = 7.2 Hz, 2H), 7.18 – 7.25 (m, 2H), 7.30 – 7.37 (m, 1H), 7.40 – 7.49 (m, 3H), 7.54 (dd, $^3J$ = 8.4, $^4J$ = 1.9, 1H), 7.61 – 7.68 (m, 3H), 7.76 (dd, $^3J$ = 8.4, $^4J$ = 1.91 1H), 9.86 (s, 1H).

**$^{13}$C NMR (75 MHz, acetone-d$_6$):** 14.38 ($CH_3$), 14.39 ($CH_3$), 23.3 (2 $CH_2$), 26.71 ($CH_2$), 26.73 ($CH_2$), 30.45 ($CH_2$), 30.46 ($CH_2$), 30.48 ($CH_2$), 30.49 ($CH_2$), 30.66 ($CH_2$), 30.68 ($CH_2$), 30.71 ($CH_2$), 30.74 ($CH_2$), 30.75 ($CH_2$), 30.76 ($CH_2$), 30.78 ($CH_2$), 30.97 ($CH_2$), 30.98 ($CH_2$), 30.99 ($CH_2$), 31.5 ($CH_3$), 32.67 ($CH_2$), 32.68 ($CH_2$), 33.1 (CH), 35.5 ($CH_2$), 52.4 ($CH_2$), 117.0 (CH), 118.3 ($C_{quat}$), 126.1 ($C_{quat}$), 126.36 (CH), 126.41 (CH), 127.0 (CH), 127.2 (2 CH), 128.1 (CH), 128.9 (CH), 129.8 (2 CH), 131.0 (CH), 132.6 ($C_{quat}$), 137.3 ($C_{quat}$), 140.3 ($C_{quat}$), 144.4 ($C_{quat}$), 152.0 ($C_{quat}$), 190.5 (CH).

**MS (EI) m/z (%):** 640 ([M], 100), 316 ([$C_{20}H_{14}NOS$]$^+$), 51), 302 ([$C_{19}H_{12}NOS$]$^+$, 31), 284 (21), 57 ([$C_4H_9$]$^+$, 40).

**IR $\tilde{\nu}$ [cm$^{-1}$]:** 3057 (w), 2922 (s), 2851 (m), 2722 (w), 1688 (s), 1601 (m), 1580 (s), 1510 (w), 1464 (s), 1416 (w), 1396 (w), 1375 (w), 1339 (m), 1308 (w), 1279 (w), 1248 (m), 1198 (s), 1163 (w), 1146 (w), 1103 (w), 1076 (w), 1040 (w), 1022 (w), 999 (w), 918 (w), 897 (w), 881 (w), 818 (m), 760 (s), 743 (w), 721 (w), 686 (s), 685 (w), 648 (w), 610 (w).

**MS(ESI-HRMS) m/z** calcd. for [$C_{43}H_{61}NOS+H^+$]: 650.4547; Found: 650.4550.

**10-(2-Decyltetradecyl)-7-(4-methoxyphenyl)-10*H*-phenothiazin-3-carbaldehyde (2b)**

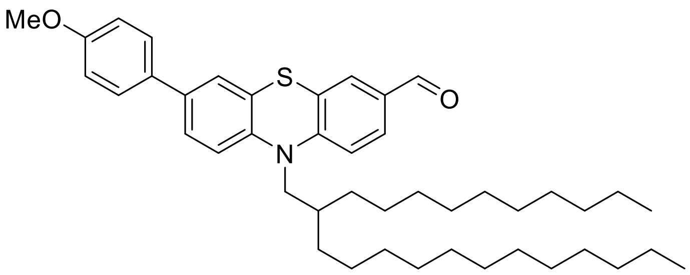


$C_{44}H_{63}NO_2S$
[670.05]

According to **GP2** and after flash chromatography on silica gel (*n*-hexane/diethyl ether 10:1) compound **2b** (550 mg, 82%) was obtained as a yellow oil.

**R**$_f$ (*n*-hexane/diethyl ether 2:1): 0.41

**$^1$H NMR (600 MHz, acetone-d$_6$):** $\delta$ 0.84 – 0.90 (m, 6H), 1.16 – 1.45 (m, 40H), 2.06 – 2.07 (m, 1H), 3.83 (s, 3 H), 3.99 (d, $^3J$ = 7.2 Hz, 2 H), 7.00 (d, $^3J$ = 8.8 Hz, 2 H), 7.17 (d, $^3J$ = 8.5 Hz, 1H), 7.22 (d, $^3J$ = 8.4 Hz, 1 H), 7.42 (d, $^4J$ = 2.1 Hz, 1H), 7.48 (dd, $^3J$ = 8.4, $^4J$ = 2.2 Hz, 1H), 7.57 (d, $^3J$ = 8.8 Hz, 2H), 7.66 (d, $^4J$ = 1.9 Hz, 1H), 7.75 (dd, $^3J$ = 8.4, $^4J$ = 1.9, 1 H), 9.85 (s, 1H).

**$^{13}$C NMR (75 MHz, acetone-d$_6$):** $\delta$ 14.38 ($CH_3$), 14.39 ($CH_3$), 23.4 (2 $CH_2$), 26.91 ($CH_2$), 26.93 ($CH_2$), 30.45 ($CH_2$), 30.46 ($CH_2$), 30.48 ($CH_2$), 30.49 ($CH_2$), 30.66 ($CH_2$), 30.68 ($CH_2$), 30.71 (CH2), 30.74 ($CH_2$), 30.75 ($CH_2$), 30.76 ($CH_2$), 30.78 ($CH_2$), 30.97 ($CH_2$), 30.98 ($CH_2$), 30.99 ($CH_2$), 31.5 ($CH_3$), 32.68 ($CH_2$), 32.69 ($CH_2$), 33.1 (CH), 52.4 ($CH_2$), 55.6 ($CH_3$), 115.1 (2 CH), 116.9 (CH), 118.3 (CH), 125.9 (CH), 126.0 ($C_{quat}$), 126.4 ($C_{quat}$), 126.5 (CH), 128.3 (2 CH, 128.9 (CH), 130.7 (CH), 132.5 ($C_{quat}$), 132.7 ($C_{quat}$), 137.2 ($C_{quat}$), 143.7 ($C_{quat}$), 152.1 ($C_{quat}$), 160.3 ($C_{quat}$), 190.5 (CH).

**MS (EI) m/z (%):** 670 ([M], 100), 346 ([$C_{21}H_{16}NO_2S$]$^+$, 85), 332 ([$C_{20}H_{14}NO_2S$]$^+$, 40), 314 (25).

**IR $\tilde{\nu}$ [cm$^{-1}$]**: 2922 (s), 2851 (s), 2725 (w), 1688 (s), 1603 (m), 1580 (s), 1562 (w), 1553 (w), 1520 (w), 1495 (m), 1464 (s), 1441 (w), 1416 (w), 1391 (w), 1373 (w), 1339 (w), 1304 (w), 1283 (w), 1244 (s), 1198 (s), 1179 (s), 1161 (w), 1146 (w), 1103 (w), 1049 (m), 1028 (m), 1007 (w), 920 (w), 895 (w), 883 (w), 831 (w), 814 (s), 773 (w), 739 (m), 719 (w), 702 (w), 687 (w), 669 (w), 625 (w).

**Anal** calcd for $C_{44}H_{63}NO_2S$ (670.1): C 78.87, H 9.48, N 2.09, S 4.78; Found: C 78.98, H 9.55, N 2.22, S 4.95.

**4-(10-(2-Decyltetradecyl)-7-formyl-10*H*-phenothiazin-3-yl)benzonitrile (2c)**

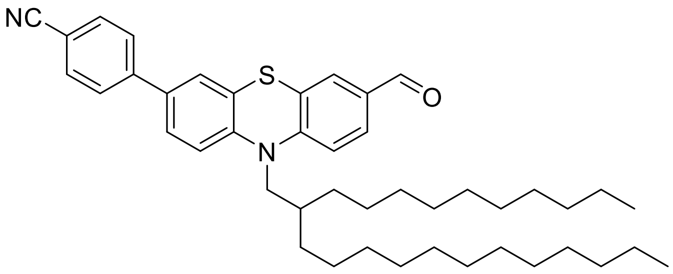


$C_{44}H_{60}N_2OS$
[665.04]

According to **GP2** and after flash chromatography on silica gel (*n*-hexane/diethyl ether 5:1) compound **2c** (432 mg, 65%) was obtained as an orange resin.

**R**$_f$ (*n*-hexane/diethyl ether 5:1): 0.44.

**$^1$H NMR (600 MHz, acetone-d$_6$):** $\delta$ 0.81 – 0.95 (m, 6H), 1.21 – 1.42 (m, 40H), 1.98 – 2.03 (m, 1H), 4.03 (d, $^3J$ = 7.3, 2H), 7.34 (dd, $^3J$ = 8.5, $^4J$ = 2.1 Hz, 2H), 7.57 (d, $^4J$ = 2.2 Hz, 1H), 7.62 (dd, $^3J$ = 8.5, $^4J$ = 2.3, Hz, 1H), 7.74 (d, $^4J$ = 1.9 Hz, 1H), 7.82 (dd, $^3J$ = 8.4, $^4J$ =1.9, 1H), 7.87 (d, $^3J$ = 8.7 Hz, 2H), 7.92 (d, $^3J$ = 8.7 Hz, 2H), 9.95 (s, 1H).

**$^{13}$C NMR (75 MHz, acetone-d$_6$):** $\delta$ 14.38 ($CH_3$), 14.39 ($CH_3$), 23.4 (2 $CH_2$), 26.91 ($CH_2$), 26.93 ($CH_2$), 30.45 ($CH_2$), 30.46 ($CH_2$), 30.48 ($CH_2$), 30.49 ($CH_2$), 30.66 ($CH_2$), 30.68 ($CH_2$), 30.71 (CH2), 30.74 ($CH_2$), 30.75 ($CH_2$), 30.76 ($CH_2$), 30.78 ($CH_2$), 30.97 ($CH_2$), 30.98 ($CH_2$), 30.99 ($CH_2$), 31.5 ($CH_3$), 32.68 ($CH_2$), 32.69 ($CH_2$), 33.1 (CH), 35.5 ($CH_2$), 52.5 ($CH_2$), 111.5 ($C_{quat}$), 117.3 (CH), 118.4 (CH) , 119.4 ($C_{quat}$), 126.2 (CH), 126.4 (CH), 126.7 ($C_{quat}$), 127.5 ($C_{quat}$), 128.0 (2 CH), 128.9 (CH), 130.8 (CH), 132.8 ($C_{quat}$), 133.6 (2 CH), 135.1 ($C_{quat}$), 144.7 ($C_{quat}$), 145.7 ($C_{quat}$), 151.6 ($C_{quat}$), 190.5 (CH).

**MS (EI) m/z (%):** 665 ([M], 100), 341 ([$C_{21}H_{13}N_2OS$]$^+$, 100), 309 (35).

**IR $\tilde{\nu}$ [cm$^{-1}$]**: 2922 (s), 2851 (s), 2722 (w), 2359 (w), 2343 (w), 2320 (w), 2226 (m), 1688 (s), 1605 (s), 1578 (s), 1558 (m), 1464 (s), 1416 (w), 1373 (m), 1339 (m), 1279 (m), 1248 (m), 1198 (s), 1180 (w), 1146 (w), 1103 (m), 1055 (w), 1045 (w), 1034 (w), 1016 (w), 1001 (w), 953 (w), 918 (w), 895 (w), 887 (w), 843 (m), 814 (s), 760 (w), 739 (m), 721 (m), 702 (w), 687 (w), 660 (w), 640 (w), 627 (w).

**Anal** calcd for $C_{44}H_{60}N_2OS$ (665.0): C 79.47, H 9.09, N 4.21, S 4.82; Found: C 79.40, H 9.07, N 4.23, S 5.08.

**10-(2-Decyltetradecyl)-7-(4-nitrophenyl)-10*H*-phenothiazin-3-carbaldehyde (2d)**

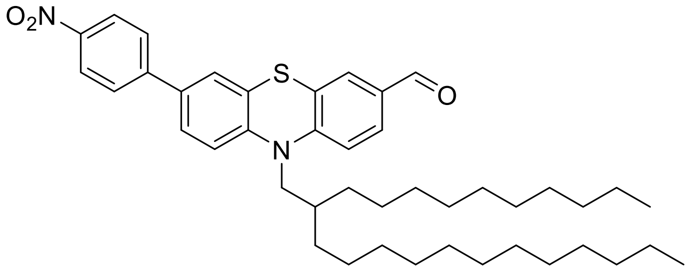

$C_{43}H_{60}N_2O_3S$
[685.02]

According to **GP2** and after flash chromatography on silica gel (*n*-hexane/diethyl ether 10:1) compound **2d** (573 mg, 84%) was obtained as a red oil.

**R*f*** (*n*-hexane/diethyl ether 2:1): 0.37.

**$^1$H NMR (600 MHz, acetone-d$_6$):** $\delta$ 0.84 – 0.89 (m, 6H), 1.15 – 1.40 (m, 40H), 2.06 – 2.08 (m, 1H), 4.03 (d, $^3J$ = 7.2 Hz, 2H), 7.27 (d, $^3J$ = 3.8 Hz, 1H), 7.28 (d, $^3J$ = 3.9 Hz, 1H), 7.63 (d, $^4J$ = 2.2 Hz, 1H), 7.68 (d, $^3J$ = 1.9 Hz, 1H), 7.70 (dd, $^3J$ = 8.5, $^4J$ = 2.2 Hz, 1H), 7.78 (dd, $^3J$ = 8.4, $^4J$ = 1.9 Hz, 1H), 7.96 (d, $^3J$ = 8.9 Hz, 2H), 8.30 (d, $^3J$ = 8.9 Hz, 2H), 9.87 (s, 1H).

**$^{13}$C NMR (75 MHz, acetone-d$_6$):** $\delta$ 14.38 ($CH_3$), 14.39 ($CH_3$), 23.4 (2 $CH_2$), 26.91 ($CH_2$), 26.93 ($CH_2$), 30.45 ($CH_2$), 30.46 ($CH_2$), 30.48 ($CH_2$), 30.49 ($CH_2$), 30.66 ($CH_2$), 30.68 ($CH_2$), 30.71 ($CH_2$), 30.74 ($CH_2$), 30.75 ($CH_2$), 30.76 ($CH_2$), 30.78 ($CH_2$), 30.97 ($CH_2$), 30.98 ($CH_2$), 30.99 ($CH_2$), 31.5 ($CH_3$), 32.68 ($CH_2$), 32.69 ($CH_2$), 33.1 (CH), 35.5 ($CH_2$), 52.5 ($CH_2$), 117.3 (CH), 118.5 (CH), 124.9 (2 CH), 126.2 ($C_{quat}$), 126.5 ($C_{quat}$), 126.9 (CH), 127.7 (CH), 128.1 (2 CH), 128.9 (CH), 130.8 (CH), 132.9 ($C_{quat}$), 134.6 ($C_{quat}$), 145.9 ($C_{quat}$), 146.7 ($C_{quat}$), 147.9 ($C_{quat}$), 151.5 ($C_{quat}$), 190.5 (CH).

**MS (EI) m/z (%):** 685 ([M], 100), 361 ([$C_{20}H_{13}N_2O_3S$]$^+$,100), 348 ([$C_{19}H_{11}N_2O_3S$]$^+$, 30), 329 (20), 315 (15), 167 (15), 149 (25), 112 (20), 97 (15), 83 (20), 57 ([$C_4H_9$]$^+$, 40).

**IR $\tilde{\nu}$ [cm$^{-1}$]**: 2920 (m), 2851 (m), 1680 (s), 1593 (m), 1578 (s), 1564 (w), 1551 (w), 1512 (s), 1464 (s), 1373 (w), 1337 (s), 1310 (w), 1287 (m), 1250 (m), 1160 (s), 1111 (m), 920 (m), 854 (s), 818 (m), 754 (m), 743 (w), 702 (m).

**Anal** calcd for $C_{43}H_{60}N_2O_3S$ (685.0): C 75.39, H 8.83, N 3.99, S 4.68; Found: C 75.19, H 8.68, N 3.99, S 4.72.

**10-(2-Decyltetradecyl)-7-(4-(trifluoromethyl)phenyl)-10*H*-phenothiazine-3-carbaldehyde (2e)**

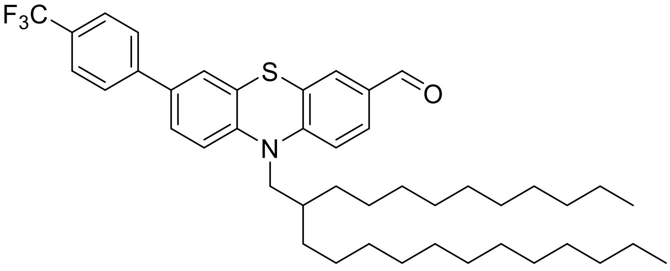

$C_{44}H_{60}F_3NOS$
[708.03]

According to **GP2** and after flash chromatography on silica gel (*n*-hexane/diethyl ether 15:1) compound **2e** (500 mg, 71%) was obtained as a yellow oil.

**R***f* (*n*-hexane/diethyl ether 15:1): 0.33

**$^1$H NMR (600 MHz, acetone-d$_6$):** $\delta$ 0.82 – 0.90 (m, 6H), 1.16 – 1.44 (m, 40H), 2.06 – 2.08 (m, 1H), 4.02 (d, $^3J$ = 7.2 Hz, 2H), 7.25 (dd, $^3J$ = 8.5, $^4J$ = 2.1 Hz, 2H), 7.57 (d, $^4J$ = 2.2 Hz, 1H), 7.63 (dd, $^3J$ = 8.5, $^4J$ = 2.2 Hz, 1H), 7.67 (d, $^4J$ = 1.9, 1H), 7.76 – 7.79 (m, 3H), 7.88 (d, $^3J$ = 8.2 Hz, 2H), 9.86 (s, 1H).

**$^{13}$C NMR (75 MHz, acetone-d$_6$):** $\delta$ 14.38 ($CH_3$), 14.39 ($CH_3$), 23.4 (2 $CH_2$), 26.91 ($CH_2$), 26.93 ($CH_2$), 30.45 ($CH_2$), 30.46 ($CH_2$), 30.48 ($CH_2$), 30.49 ($CH_2$), 30.66 ($CH_2$), 30.68 ($CH_2$), 30.71 (CH2), 30.74 ($CH_2$), 30.75 ($CH_2$), 30.76 ($CH_2$), 30.78 ($CH_2$), 30.97 ($CH_2$), 30.98 ($CH_2$), 30.99 ($CH_2$), 31.5 ($CH_3$), 32.68 ($CH_2$), 32.69 ($CH_2$), 33.1 (CH), 35.4 ($CH_2$), 52.5 ($CH_2$), 117.2 (CH), 118.4 (CH), 126.2 ($C_{quat}$), 126.4 ($C_{quat}$), 126.6 ($CF_3$), 126.7 (CH), 127.4 (CH), 127.8 (4CH), 128.9 (CH), 1293. ($C_{quat}$), 130.8 (CH), 132.8 ($C_{quat}$), 135.5 ($C_{quat}$), 144.2 ($C_{quat}$), 145.4 ($C_{quat}$), 151.7 ($C_{quat}$), 190.5 (CH).

**MS (EI) m/z (%):** 708 ([M], 100), 341 ([$C_{21}H_{13}F_3NOS$]$^+$, 95), 352 (30).

**IR $\tilde{\nu}$ [cm$^{-1}$]**: 3049 (w), 2922 (s), 2853 (m), 2722 (w), 1688 (s), 1616 (w), 1603 (w), 1580 (m), 1564 (w), 1553 (w), 1526 (w), 1495 (w), 1464 (s), 1416 (w), 1375 (w), 1323 (s), 1283 (w), 1263 (w), 1250 (w), 1180 (s), 1163 (s), 1125 (s), 1111 (w), 1072 (s), 1045 (w), 1036 (w), 1015 (m), 999 (w), 953 (w), 920 (w), 887 (w), 843 (m), 814 (s), 768 (w), 741 (w), 723 (w), 706 (w), 685 (w), 675 (w), 648 (w), 625 (w).

**Anal** calcd for $C_{44}H_{60}F_3NOS$ (708.0): C 76.64, H 8.54, N 1.98, S 4.53; Found: C 74.89, H 8.81, N 1.98, S 4.34.

**10-(2-Decyltetradecyl)-7-(4-(diphenylamino)phenyl)-10*H*-phenothiazine-3-carbaldehyde (2f)**

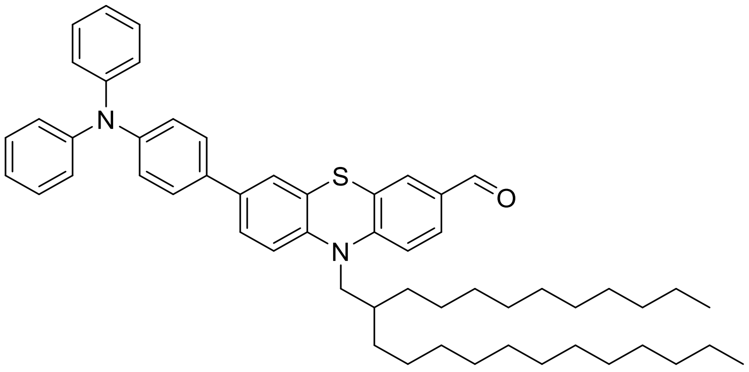

$C_{55}H_{70}N_2OS$
[807.24]

According to **GP2** and after flash chromatography on silica gel (*n*-hexane/diethyl ether 25:1) compound **2f** (624 mg, 77%) was obtained as a yellow oil.

**$R_f$** (*n*-hexane/diethyl ether 25:1): 0.40.

**$^1$H NMR (300 MHz, acetone-d$_6$):** $\delta$ 0.81 – 0.92 (m, 6H), 1.16 – 1.46 (m, 40H), 2.07 - 2.08 (m, 1H), 3.99 (d, $^3J$ = 7.1 Hz, 2H), 7.03 – 7.12 (m, 8H), 7.15 – 7.25 (m, 2H), 7.26 – 7.38 (m, 4H), 7.46 (d, $^4J$ = 2.1 Hz, 1H), 7.52 (dd, $^3J$ = 8.5, $^4J$ = 2.2 Hz, 1H), 7.57 (d, $^3J$ = 8.6 Hz, 2H), 7.66 (d, $^4J$ = 1.9 Hz, 1H), 7.75 (dd, $^3J$ = 8.4, $^4J$ = 1.9 Hz, 1H), 9.85 (s, 1H).

**$^{13}$C NMR (150 MHz, acetone-d$_6$):** $\delta$ 14.38 ($CH_3$), 14.39 ($CH_3$), 23.4 (2 $CH_2$), 26.91 ($CH_2$), 26.93 ($CH_2$), 30.45 ($CH_2$), 30.46 ($CH_2$), 30.48 ($CH_2$), 30.49 ($CH_2$), 30.66 ($CH_2$), 30.68 ($CH_2$), 30.71 (CH2), 30.74 ($CH_2$), 30.75 ($CH_2$), 30.76 ($CH_2$), 30.78 ($CH_2$), 30.97 ($CH_2$), 30.98 ($CH_2$), 30.99 ($CH_2$), 31.5 ($CH_3$), 32.68 ($CH_2$), 32.69 ($CH_2$), 33.1 (CH), 35.4 ($CH_2$), 52.5 ($CH_2$), 117.0 (CH), 118.3 (CH), 124.1 (2 CH), 124.5 (2 CH), 125.3 (4 CH), 125.9 (CH), 126.0 ($C_{quat}$), 126.3 ($C_{quat}$), 126.5 (CH), 128.0 (2 CH), 128.9 (CH), 130.3 (4 CH), 130.7 (CH), 132.5 ($C_{quat}$), 134.3 ($C_{quat}$), 136.8 ($C_{quat}$), 143.9 ($C_{quat}$), 148.1 ($C_{quat}$), 148.6 (2 $C_{quat}$), 152.0 ($C_{quat}$), 190.5 (CH).

**MS (EI) m/z (%):** 807 ([M], 100), 483 ($[C_{32}H_{23}N_2OS]^+$, 35), 469 ($[C_{31}H_{21}N_2OS]^+$, 65), 451 (20), 242 (30), 226 (40).

**IR $\tilde{\nu}$ [cm$^{-1}$]**: 3059 (w), 3034 (w), 2922 (s), 2851 (s), 2718 (w), 2617 (w), 1688 (s), 1653 (w), 1580 (s), 1558 (m), 1539 (w), 1518 (w), 1491 (s), 1464 (s), 1416 (w), 1393 (w), 1373 (w), 1327 (m), 1312 (w), 1273 (s), 1250 (m), 1198 (s), 1180 (w), 1146 (w), 1103 (w), 1074 (w), 1047 (w), 1028 (w), 1013 (w), 999 (w), 961 (w), 920 (w), 895 (w), 883 (w), 814 (s), 752 (s), 739 (w), 721 (w), 694 (s), 669 (w), 617 (w).

**Anal** calcd for $C_{55}H_{70}N_2OS$ (807.2): C 81.84, H 8.74, N 3.47, S 3.97; Found: C 82.06, H 8.98, N 3.51, S 4.14.

**7-(4-(Bis(4-methoxyphenyl)amino)phenyl)-10-(2-decyltetradecyl)-10*H*-phenothiazine-3-carbaldehyde (2g)**

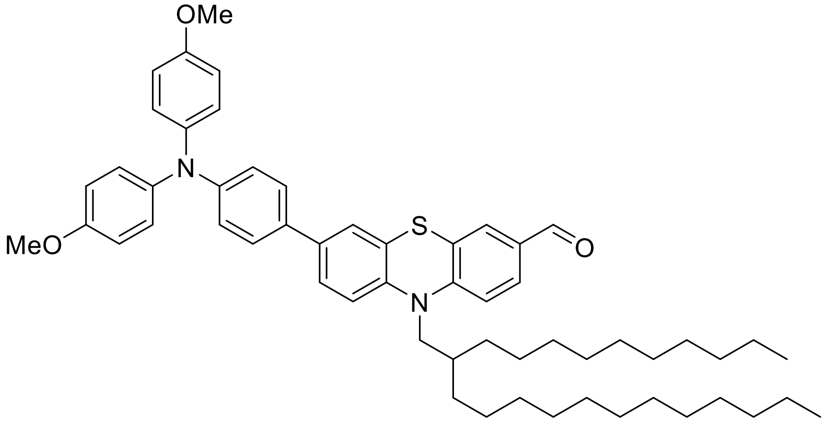

$C_{55}H_{70}N_2OS$
[867.29]

According to **GP2** and after flash chromatography on silica gel (*n*-hexane/diethyl ether 5:1) compound **2g** (710 mg, 82%) was obtained as a yellow oil.

**R***f* (*n*-hexane/diethyl ether 25:1): 0.32.

**$^1$H NMR (300 MHz, acetone-d$_6$):** $\delta$ 0.83 – 0.90 (m, 6H), 1.17 – 1.47 (m, 40H), 2.07 – 2.08 (m, 1H), 3.79 (s, 6H), 3.98 (d, $^3J$ = 7.2 Hz, 2H), 6.84 – 6.97 (m, 6H), 7.04 – 7.11 (m, 4H), 7.15 (d, $^3J$ = 8.5 Hz, 1H), 7.21 (d, $^3J$ = 8.5 Hz, 1H), 7.41 (d, $^4J$ = 2.1 Hz, 1H), 7.45 - 7.50 (m, 3H), 7.65 (d, $^4J$ = 1.9, 1H), 7.74 (dd, $^3J$ = 8.4, $^4J$ = 1.9 Hz, 1H), 9.84 (s, 1H).

**$^{13}$C NMR (150 MHz, acetone-d$_6$):** $\delta$ 14.38 ($CH_3$), 14.39 ($CH_3$), 23.4 (2 $CH_2$), 26.91 ($CH_2$), 26.93 ($CH_2$), 30.45 ($CH_2$), 30.46 ($CH_2$), 30.48 ($CH_2$), 30.49 ($CH_2$), 30.66 ($CH_2$), 30.68 ($CH_2$), 30.71 (CH2), 30.74 ($CH_2$), 30.75 ($CH_2$), 30.76 ($CH_2$), 30.78 ($CH_2$), 30.97 ($CH_2$), 30.98 ($CH_2$), 30.99 ($CH_2$), 31.5 ($CH_2$), 32.68 ($CH_2$), 32.69 ($CH_2$), 33.1 (CH), 35.4 ($CH_2$), 52.5 ($CH_2$), 55.7 (2 $CH_3$), 115.7 (4 CH), 116.9 (CH), 118.3 (CH), 121.0 (2 CH), 125.6 (CH), 126.0 ($C_{quat}$), 126.2 CH), 126.3 ($C_{quat}$), 127.7 (6 CH), 128.9 (CH), 130.7 (CH), 131.9 ($C_{quat}$), 132.5 ($C_{quat}$), 137.1 ($C_{quat}$), 141.5 (2 $C_{quat}$), 143.5 ($C_{quat}$), 149.3 ($C_{quat}$), 152.1 ($C_{quat}$), 157.3 (2 $C_{quat}$), 190.4 (CH).

**MS (EI) m/z (%):** 867 ([M], 100), 543 ([$C_{34}H_{27}N_2O_3S$]$^+$, 15), 529 ([$C_{33}H_{25}$N2$O_3$S]$^+$, 45), 272 (25).

**IR $\tilde{\nu}$ [cm$^{-1}$]**: 3038 (w), 2995 (w), 2922 (s), 2851 (s), 2722 (w), 1688 (s), 1601 (m), 1580 (m), 1560 (w), 1503 (s), 1460 (s), 1441 (w), 1416 (w), 1375 (w), 1319 (w), 1275 (w), 1238 (s), 1196 (m), 1179 (w), 1165 (w), 1146 (w), 1103 (m), 1009 (w), 918 (w), 895 (w), 883 (w), 826 (s), 816 (s), 871 (w), 727 (w), 721 (w), 694 (w), 687 (w).

**Anal** calcd for $C_{55}H_{70}N_2OS$ (867.3): C 78.84, H 8.60, N 3.23, S 3.70; Found: C 79.02, H 8.70, N 3.34, S 3.99.

**10-(2-Decyltetradecyl)-7-(quinolin-3-yl)-10*H*-phenothiazine-3-carbaldehyde (2h)**

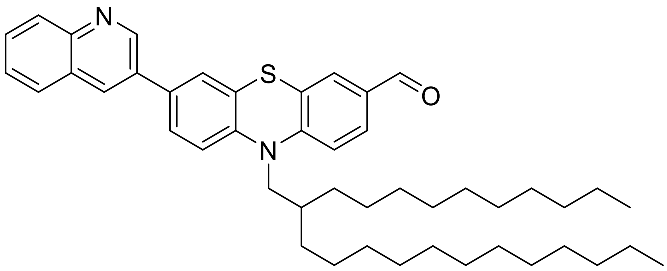

$C_{46}H_{62}N_2OS$
[691.08]

According to **GP2** and after flash chromatography on silica gel (*n*-hexane/diethyl ether 2:1) compound **2h** (594 mg, 80%) was obtained as a yellow oil.

**R**$_f$ (*n*-hexane/diethyl ether 2:1): 0.35.

**$^1$H NMR (600 MHz, acetone-d$_6$):** $\delta$ 0.81 – 0.88 (m, 6H), 1.17 – 1.45 (m, 40H), 2.06 – 2.11 (m, 1H), 4.03 (d, $^3J$ = 7.2 Hz, 2H), 7.26 (d, $^3J$ = 8.5 Hz, 1H), 7.29 (d, $^3J$ = 8.5 Hz, 1H), 7.59 – 7.65 (m, 1H), 7.69 (dd, $^3J$ = 6.5, $^4J$ = 2.0 Hz, 2H), 7.73 – 7.77 (m, 2H), 7.78 (dd, $^3J$ = 8.4, $^4J$ = 1.9 Hz, 1H), 7.99 – 8.02 (m, 1H), 8.05 – 8.08 (m, 1H), 8.52 – 8.54 (m, 1H), 9.22 (d, $^4J$ = 2.4 Hz, 1H), 9.87 (s, 1H).

**$^{13}$C NMR (75 MHz, acetone-d$_6$):** $\delta$ 14.38 ($CH_3$), 14.39 ($CH_3$), 23.4 (2 $CH_2$), 26.91 ($CH_2$), 26.93 ($CH_2$), 30.45 ($CH_2$), 30.46 ($CH_2$), 30.48 ($CH_2$), 30.49 ($CH_2$), 30.66 ($CH_2$), 30.68 ($CH_2$), 30.71 (CH2), 30.74 ($CH_2$), 30.75 ($CH_2$), 30.76 ($CH_2$), 30.78 ($CH_2$), 30.97 ($CH_2$), 30.98 ($CH_2$), 30.99 ($CH_2$), 31.5 ($CH_3$), 32.68 ($CH_2$), 32.69 ($CH_2$), 33.2 (CH), 35.5 ($CH_2$), 52.5 ($CH_2$), 117.2 (CH), 118.6 (CH), 126.2 ($C_{quat}$), 126.5 ($C_{quat}$), 126.8 (CH), 127.5 (CH), 127.8 (CH), 128.9 (CH), 129.0 ($C_{quat}$), 129.1 (CH), 130.0 (2 CH), 130.8 (CH), 132.7 ($C_{quat}$), 132.9 (CH), 133.0 ($C_{quat}$), 134.1 ($C_{quat}$), 145.1 ($C_{quat}$), 148.4 ($C_{quat}$), 150.1 (CH), 151.8 ($C_{quat}$), 190.5 CH).

**MS (EI) m/z (%):** 691 ([M], 100), 367 ($[C_{23}H_{15}N_2OS]^+$, 90), 354 ($[C_{22}H_{13}$N2OS$]^+$, 45), 335 (30), 325 (15), 184 (20), 57 ($[C_4H_9]^+$, 15).

**IR $\tilde{\nu}$ [cm$^{-1}$]**: 2922 (s), 2851 (s), 2723 (w), 1688 (s), 1603 (m), 1580 (s), 1564 (w), 1555 (w), 1456 (s), 1416 (w), 1396 (w), 1373 (w), 1340 (m), 1308 (w), 1288 (w), 1271 (w), 1250 (m), 1229 (w), 1198 (s), 1161 (w), 1146 (w), 1125 (w), 1101 (w), 1053 (w), 1040 (w), 1015 (w), 999 (w), 962 (w), 957 (w), 887 (w), 878 (w), 858 (w), 816 (s), 785 (m), 750 (s), 737 (w), 723 (m), 704 (w), 687 (w), 638 (w).

**Anal** calcd for $C_{46}H_{62}N_2OS$ (691.1): C 79.95, H 9.04, N 4.05, S 4.64; Found: C 79.91, H 9.31, N 4.00, S 4.68.

**7-(Anthracen-9-yl)-10-(2-decyltetradecyl)-10*H*-phenothiazine-3-carbaldehyde (2i)**

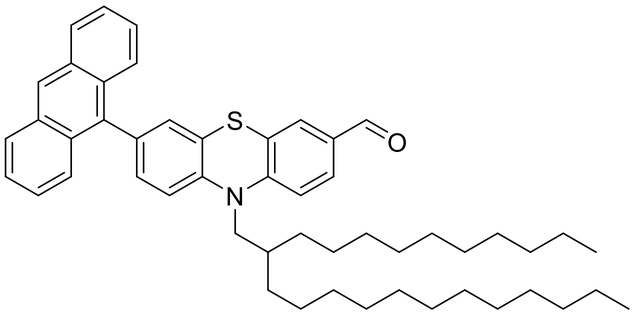

$C_{51}H_{65}NOS$
[740.15]

According to **GP2** and after flash chromatography on silica gel (*n*-hexane/diethyl ether 20:1) compound **2i** (550 mg, 80%) was obtained as a yellow oil.

**R*f*** (*n*-hexane/diethyl ether 20:1): 0.45.

**$^1$H NMR (600 MHz, acetone-d$_6$):** $\delta$ 0.80 – 0.88 (m, 6H), 1.15 – 1.52 (m, 40H), 2.19 (m, 1H), 4.12 (d, $^3J$ = 7.2 Hz, 2H), 7.25 (d, $^4J$ = 2.0 Hz, 1H), 7.30 (d, $^4J$ = 2.0 Hz, 1H), 7.33 (d, $^3J$ = 8.6 Hz, 1H), 7.38 – 7.44 (m, 3H), 7.48 – 7.53 (m, 2H), 7.67 (dd, $^3J$ = 8.8, $^4J$ = 1.0 Hz, 2H), 7.71 (d, $^4J$ = 1.9 Hz, 1H), 7.82 (dd, $^3J$ = 8.4, $^4J$ = 1.9 Hz, 1H), 8.13 (d, $^3J$ = 8.6 Hz, 2H), 8.63 (m, 1H), 9.89 (s, 1H).

**$^{13}$C NMR (75 MHz, acetone-d$_6$):** $\delta$ 14.38 ($CH_3$), 14.39 ($CH_3$), 23.4 (2 $CH_2$), 26.91 ($CH_2$), 26.93 ($CH_2$), 30.45 ($CH_2$), 30.46 ($CH_2$), 30.48 ($CH_2$), 30.49 ($CH_2$), 30.66 ($CH_2$), 30.68 ($CH_2$), 30.71 (CH2), 30.74 ($CH_2$), 30.75 ($CH_2$), 30.76 ($CH_2$), 30.78 ($CH_2$), 30.97 ($CH_2$), 30.98 ($CH_2$), 30.99 ($CH_2$), 31.5 ($CH_3$), 32.68 ($CH_2$), 32.69 ($CH_2$), 33.2 (CH), 35.4 ($CH_2$), 52.6 ($CH_2$), 117.2 (CH), 118.1 (CH), 125.9 ($C_{quat}$), 126.1 (2 CH), 126.5 ($C_{quat}$), 126.6 (2 CH), 127.2 (2 CH), 127.7 (2 $C_{quat}$), 129.1 (CH), 129.4 (2 CH), 130.6 (CH), 130.7 (CH), 131.1 ($C_{quat}$), 131.5 (CH), 132.4 (2 $C_{quat}$), 132.7 ($C_{quat}$), 134.8 ($C_{quat}$), 136.3 ($C_{quat}$), 144.7 (Cv), 152.2 ($C_{quat}$), 190.5 (CH).

**MS (EI) m/z (%):** 740 ([M], 40), 643 (20), 416 ($[C_{28}H_{18}NOS]^+$, 95), 402 ($[C_{27}H_{16}NOS]^+$, 50), 384 (30), 320 (20), 208 (20), 110 (20), ), 85 ($[C_6H_{13}]^+$, 15), 71 ($[C_5H_{11}]^+$, 20), 57 ($[C_4H_9]^+$, 40).

**IR $\tilde{\nu}$ [cm$^{-1}$]**: 3076 (w), 3049 (w), 3030 (w), 2718 (w), 1688 (s), 1603 (m), 1580 (s), 1562 (w), 1520 (w), 1499 (w), 1464 (s), 1443 (w), 1411 (m), 1393 (w), 1373 (w), 1358 (m), 1339 (w), 1306 (m), 1250 (m), 1198 (s), 1148 (w), 1099 (w), 1042 (w), 1015 (w), 1001 (w), 957 (w), 918 (w), 883 (m), 843 (m), 816 (s), 791 (m), 758 (w), 735 (s), 702 (w), 687 (w), 675 (w).

**Anal** calcd for $C_{51}H_{65}NOS$ (740.2): C 82.76, H 8.85, N 1.89, S 4.33; Found: C 82.72, H 8.86, N 1.81, S 4.45.

### 2.1.3 General procedure III (GP3) for the Buchwald-Hartwig arylamination

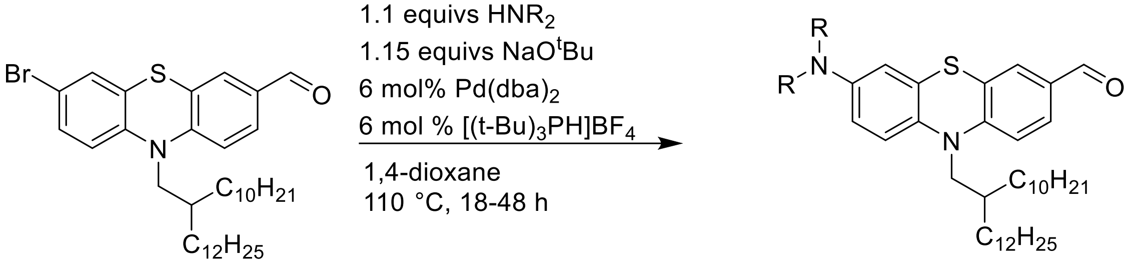


Compound **1c (**1.0 equiv, 0.642 g, 1.0 mmol), the secondary amine (1.1 equivs), sodium *t*-butoxide (1.15 equivs, 0.111 g, 1.15 mmol), tri-tert-butylphosphonium tetrafluoroborate (17 mg, 6.0 mol%) and $Pd(dba)_2$ (35 mg, 6.0 mol%) were placed in a dry Schlenk tube under nitrogen atmosphere and dissolved in 3 mL dry 1,4 dioxane. The solution was degassed for 10 min with nitrogen and then stirred for 24 h at 110 °C. The crude product was diluted in dichloromethane and washed with saturated sodium sulfite solution. Then the solution was extracted three times with dichloromethane. The combined organic phases were dried with anhydrous magnesium sulfate. After filtration the crude product was adsorbed on Celite® and purified via flash chromatography on silica gel (*n*-hexane/diethyl ether).

**Table S3:** Experimental details for the synthesis of phenothiazinyl aldehydes **2j-l**.

| Entry | amine | Yield of **9** |
|---|---|---|
| 1 | 0.219 g of 10*H* phenothiazine | 346 mg (48%) of **2j** |
| 2 | 0.186 g of diphenylamine | 235 mg (32%) of **2k** |
| 3 | 0.252 g bis(4-methoxyphenyl)amine | 411 mg (52%) of **2l** |

**10-(2-Decyltetradecyl)-7-(diphenylamino)-10*H*-phenothiazine-3-carbaldehyde (2k)**

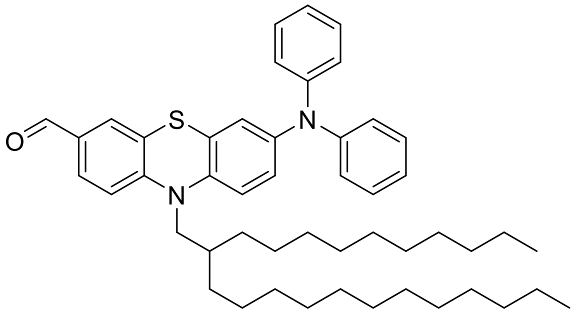


$C_{49}H_{66}N_2OS$
[731.14]

According to **GP3** and after flash chromatography on silica gel (*n*-hexane/diethyl ether 40:1) compound **2k** (235 mg, 32%) was obtained as a yellow oil.

**$R_f$ (*n*-hexane/diethyl ether 40:1):** 0.40

**$^1$H NMR (600 MHz, acetone-d$_6$):** $\delta$ 0.84 – 0.90 (m, 6H), 1.20 – 1.44 (m, 40H), 2.06 – 2.07 (m, 1H), 3.95 (d, $^3J$ = 7.2 Hz, 2H), 6.87 (d, $^4J$ = 2.5 Hz, 1H), 6.93 (dd, $^3J$ = 8.7, $^4J$ = 2.6 Hz, 1H), 7.01 – 7.05 (m, 6H), 7.07 (d, $^3J$ = 8.8 Hz, 1H), 7.21 (d, $^3J$ = 8.5 Hz, 1 H), 7.26 – 7.31 (m, 4H), 7.61 (d, $^4J$ = 1.9 Hz, 1H), 7.75 (dd, $^3J$ = 8.4, $^4J$ = 1.9 Hz, 1H), 9.84 (s, 1H).

**$^{13}$C NMR (75 MHz, acetone-d$_6$):** $\delta$ 14.38 ($CH_3$), 14.39 ($CH_3$), 23.4 (CH), 26.74 ($CH_2$), 26.77 ($CH_2$), 30.45 ($CH_2$), 30.46 ($CH_2$), 30.48 ($CH_2$), 30.49 ($CH_2$), 30.66 ($CH_2$), 30.68 ($CH_2$), 30.71 ($CH_2$), 30.75 ($CH_2$), 30.76 ($CH_2$), 30.78 ($CH_2$), 30.97 ($CH_2$), 30.98 ($CH_2$), 30.99 ($CH_2$), 31.99 ($CH_2$), 32.11 ($CH_3$), 32.66 ($CH_2$), 32.67 ($CH_2$), 35.4 (CH), 52.4 ($CH_2$), 116.9 (CH), 118.7 (CH), 123.7 (2 CH), 124.1 (CH), 124.5 (4 CH), 124.7 (CH), 126.0 ($C_{quat}$), 126.5 ($C_{quat}$), 128.9 (CH), 130.3 (4 CH), 130.7 (CH), 132.4 ($C_{quat}$), 140.5 ($C_{quat}$), 144.8 ($C_{quat}$), 148.6 (2 $C_{quat}$), 152.2 ($C_{quat}$), 190.4 (CH).

**MS (EI) m/z (%):** 731 ([M], 50), 407 ([$C_{26}H_{19}N_2OS$]$^+$, 25), 393 ([$C_{25}H_{17}N_2OS$]$^+$, 100) 57 ([$C_4H_9$]$^+$, 20).

**IR $\tilde{\nu}$ [cm$^{-1}$]**: 2920 (s), 2851 (m), 2722 (w), 1688 (s), 1593 (s), 1578 (w), 1555 (w), 1491 (m), 1460 (s), 1412 (w), 1371 (w), 1331 (w), 1308 (w), 1296 (w), 1273 (m), 1260 (w), 1250 (w), 1196 (s), 1153 (w), 1142 (w), 1099 (w), 1074 (w), 1028 (w), 999 (w), 957 (w), 920 (w), 897 (w), 870 (w), 814 (m), 750 (s), 719 (w), 694 (s), 650 (w), 633 (w).

**Anal** calcd for $C_{49}H_{66}N_2OS$ (731.1): C 80.50, H 9.10, N 3.83, S 4.38; Found: C 80.61, H 9.10, N 3.85, S 4.55.

**7-(Bis(4-methoxyphenyl)amino)-10-(2-decyltetradecyl)-10*H*-phenothiazine-3-carbaldehyde (2l)**

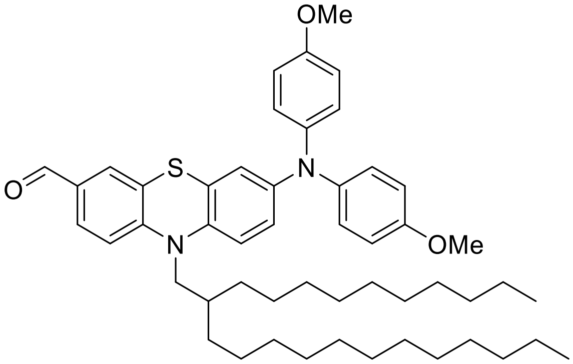


$C_{51}H_{70}N_2O_3S$
[791.19]

According to **GP3** and after flash chromatography on silica gel (*n*-hexane/diethyl ether 20:1) compound **2l** (411 mg, 52%) was obtained as a yellow oil.

**R*f* (*n*-hexane/diethyl ether 20:1):** 0.36

**$^1$H NMR (600 MHz, acetone-d$_6$):** *δ* 0.84 – 0.90 (m, 6H), 1.20 – 1.39 (m, 40H), 2.00 – 2.04 (m, 1 H), 3.78 (s, 6H), 3.90 (d, $^3J$ = 7.1 Hz, 2H), 6.69 (d, $^4J$ = 2.6 Hz, 1H), 6.76 (dd, $^3J$ = 8.8, $^4J$ = 2.6 Hz, 1H), 6.85 – 6.91 (m, 4H), 6.97 (d, $^3J$ = 8.9 Hz, 1H), 6.98 – 7.02 (m, 4H), 7.17 (d, $^3J$ = 8.4 Hz, 1H), 7.59 (d, $^4J$ = 1.9 Hz, 1H), 7.72 (dd, $^3J$ = 8.4, $^4J$ = 1.9 Hz, 1H), 9.82 (s, 1H).

**$^{13}$C NMR (75 MHz, acetone-d$_6$):** *δ* 14.38 ($CH_3$), 14.39 ($CH_3$), 23.4 (CH), 26.74 ($CH_2$), 26.77 ($CH_2$), 30.45 ($CH_2$), 30.46 ($CH_2$), 30.48 ($CH_2$), 30.49 ($CH_2$), 30.66 ($CH_2$), 30.68 ($CH_2$), 30.71 ($CH_2$), 30.75 ($CH_2$), 30.76 ($CH_2$), 30.78 ($CH_2$), 30.97 ($CH_2$), 30.98 ($CH_2$), 30.99 ($CH_2$), 31.99 ($CH_2$), 32.01 ($CH_3$), 32.66 ($CH_2$), 32.67 ($CH_2$), 35.4 (CH), 52.3 ($CH_2$), 55.7 (2 $CH_3$), 115.6 (4 CH), 116.6 (CH), 118.4 (CH), 120.5 (CH), 121.1 (CH), 125.9 ($C_{quat}$), 126.2 ($C_{quat}$), 127.0 (4 CH), 128.9 (CH), 130.7 (CH), 132.1 ($C_{quat}$), 138.2 ($C_{quat}$), 141.7 (2 $C_{quat}$), 146.2 ($C_{quat}$), 152.5 ($C_{quat}$), 157.0 (2 $C_{quat}$), 190.3 (CH).

**MS (EI) m/z (%):** 791 ([M], 50), 453 ($[C_{27}H_{21}N_2O_3S]^+$, 90), 57 ($[C_4H_9]^+$, 15).

**IR $\tilde{\nu}$ [cm$^{-1}$]**: 2922 (m), 2851 (m), 2178 (w), 1686 (m), 1599 (w), 1582 (w), 1551 (w), 1503 (s), 1460 (s), 1441 (w), 1406 (w), 1375 (w), 1321 (w), 1308 (w), 1296 (w), 1238 (s), 1198 (m), 1180 (w), 1169 (w), 1146 (w), 1103 (w), 1038 (m), 1009 (w), 959 (w), 918 (w), 897 (w), 883 (w), 866 (w), 826 (m), 781 (w), 762 (w), 716 (w), 685 (w), 640 (w).

**Anal** calcd for $C_{51}H_{70}N_2O_3S$ (791.2): C 77.42, H 8.92, N 3.54, S 4.05; Found: C 77.61, H 8.94, N 3.54, S 4.05.

## 2.1.4 General procedure IV (GP4) for the Ullmann coupling

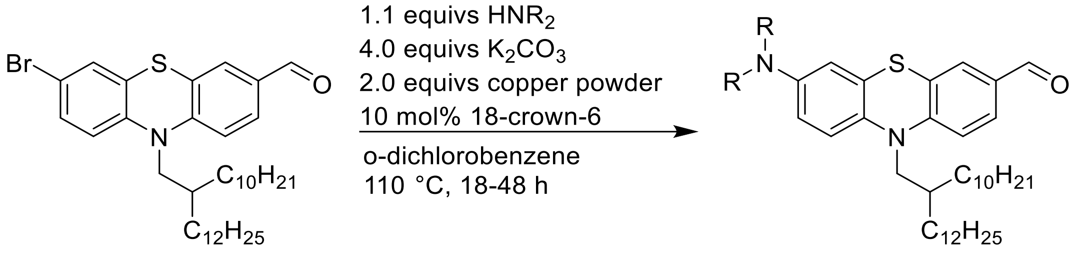


**1c** **2m-n**

Compound **1c (**1.0 mmol, 0.642 g, 1.0 equiv), the secondary amine (1.1 equivs), anhydrous potassium carbonate (4.0 equivs, 0.553 g, 4.0 mmol) , copper powder (2.0 equivs, 0.127 g, 2.0 mmol) and 18-crown-6 (0.026 g, 10 mol%) were placed in a dry Schlenk tube under nitrogen atmosphere and dissolved in 6 mL dry o-dichlorobenzene. The solution was degassed for 10 min with nitrogen and afterwards stirred for 48 h at 190 °C. The crude product was adsorbed on Celite® and purified via flash chromatography on silica gel (*n*-hexane/diethyl ether).

**Table 4.** Experimental details for the synthesis of phenothiazinyl aldehydes **2m-n**.

| Entry | amine | Yield of **2** |
|---|---|---|
| 1 | 0.184 g of 9*H*-carbazole | 463 mg (64%) of **2m** |
| 2 | 0.219 g of bis(4-nitrophenyl)amine | 330 mg (40%) of **2n** |

**7-(Bis(4-nitrophenyl)amino)-10-(2-decyltetradecyl)-10*H*-phenothiazine-3-carbaldehyde (2n)**

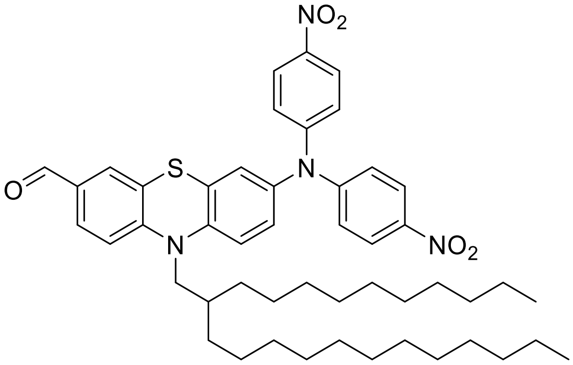

$C_{49}H_{64}N_4O_5S$
[821.13]

According to **GP4** and after flash chromatography on silica gel (*n*-hexane/dichloromethane 1:1) compound **2n** (330 mg, 40%) was obtained as a red oil.

**R*f* (dichloromethane):** 0.38

**$^1$H NMR (600 MHz, acetone-d$_6$):** $\delta$ 0.87 (m, 6H), 1.20 – 1.45 (m, 40H), 2.06 – 2.08 (m, 1H), 4.01 (d, $^3J$ = 7.2 Hz, 2H), 7.18 (d, $^4J$ = 2.5 Hz, 1H), 7.21 (dd, $^3J$ = 8.6, $^4J$ = 2.5 Hz, 1H), 7.26 (d, $^3J$ = 8.7 Hz, 1H), 7.27 - 7.29 (m, 1H), 7.29 (d, $^3J$ = 9.2 Hz, 4H), 7.65 (d, $^4J$ = 1.9 Hz, 1H), 7.80 (dd, $^3J$ = 8.4, $^4J$ = 1.9 Hz, 1H), 8.19 (d, $^3J$ = 9.2 Hz, 4H), 9.86 (s, 1H).

**$^{13}$C NMR (75 MHz, acetone-d$_6$):** $\delta$ 14.38 ($CH_3$), 14.39 ($CH_3$), 23.4 ($CH_2$) 26.74 ($CH_2$), 26.77 ($CH_2$), 30.45 ($CH_2$), 30.46 ($CH_2$), 30.49 ($CH_2$), 30.66 ($CH_2$), 30.68 ($CH_2$), 30.71 ($CH_2$), 30.74 ($CH_2$), 30.75 ($CH_2$), 30.76 ($CH_2$), 30.78 ($CH_2$), 30.97 ($CH_2$), 30.98 ($CH_2$), 30.99 ($CH_2$), 32.00 ($CH_2$), 32.02 ($CH_2$), 32.64 ($CH_2$), 32.66 ($CH_2$), 35.5 (CH), 52.6 ($CH_2$), 117.4 (CH), 119.4 (CH), 123.3 (4 CH), 126.0 ($C_{quat}$), 126.2 (4 CH), 127.4 (CH), 127.7 ($C_{quat}$), 128.0 (CH), 129.0 (CH), 130.9 (CH), 132.9 ($C_{quat}$), 141.4 ($C_{quat}$), 143.6 (2 $C_{quat}$), 144.2 ($C_{quat}$), 151.6 ($C_{quat}$), 152.8 (2 $C_{quat}$), 190.5 (CH).

**MS (EI) m/z (%):** 821 ([M], 100), 497 ([$C_{26}H_{17}N_4O_5S$]$^+$, 90), 483 ([$C_{25}H_{15}N_4O_5S$]$^+$, 90), 437 (30), 57 ([$C_4H_9$]$^+$, 40).

**IR $\tilde{\nu}$ [cm$^{-1}$]**: 2922 (m), 2851 (m), 1688 (m), 1595 (m), 1580 (m), 1493 (m), 1460 (m), 1416 (w), 1404 (w), 1375 (w), 1337 (m), 1300 (s), 1279 (s), 1198 (m), 1180 (w), 1157 (w), 1144 (w), 1109 (s), 1042 (w), 1007 (w), 955 (w), 922 (w), 897 (w), 872 (w), 843 (m), 818 (m), 775 (w), 750 (m), 737 (w), 725 (w), 691 (m9; 679 (w), 648 (w), 633 (w), 621 (w).

**Anal** calcd for $C_{49}H_{64}N_4O_5S$ (821.1): C 71.67, H 7.86, N 6.82, S 3.90; Found: C 71.53, H 7.67, N 6.65, S 3.97.

**10-(2-Decyltetradecyl)-7-morpholino-10*H*-phenothiazine-3-carbaldehyde (2o)**

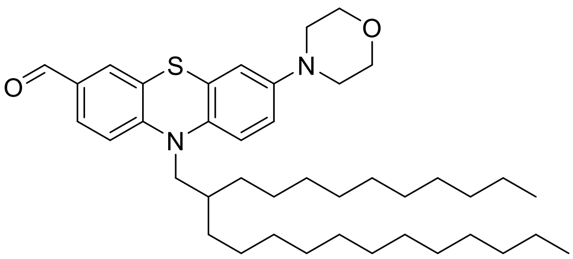

[$C_{41}H_{64}N_2O_2S$]
649.04

Compound **1c** (1.0 equiv, 0.642 g, 1.0 mmol), potassium iodide (0.019 g, 10 mol%), anhydrous potassium carbonate (2.0 equivs, 0.276 g, 2.0 mmol) and *L*-proline (0.023 g, 20 mol%) were placed in a dry Schlenk tube under nitrogen atmosphere and dissolved in 1.3 mL dry DMSO. Then morpholine (3.0 equivs, 0.261 g (3.0 mmol) was added via syringe. The solution was degassed for 10 min with nitrogen and afterwards stirred for 24 h at 100 °C. Then another portion of morpholine (1.5 equivs, 0.130 g, 1.5 mmol), anhydrous potassium carbonate (1.0 mmol, 0.138 g, 1.0 equiv), copper iodide (15 mg, 10 mol%) and *L*-proline (7 mg, 6 mol%) were added under nitrogen atmosphere to the solution after cooling to room temp. The suspension was stirred for addition 24 h at 100 °C. The crude product was diluted in dichloromethane and extracted tree times with water. The combined organic phases were dried with anhydrous magnesium sulfate. After filtration the crude product was adsorbed on Celite® and purified via flash chromatography on silica gel (*n*-hexane/diethyl ether 30:1-1:1). Compound **2o** (0.25 g, 39%) was obtained as a yellow oil.

**$R_f$ (*n*-hexane/diethyl ether 1:1): 0.38**

**$^1$H NMR (600 MHz, acetone-d$_6$):** $\delta$ 0.83 - 0.92 (m, 6H), 1.18 – 1.45 (m, 40H), 1.96 - 2.02 (m, 1H), 3.07 – 3.09 (m, 4H), 3.73 – 3.78 (m, 4H), 3.90 (d, $^3J$ = 7.2 Hz, 2H), 6.79 (d, $^4J$ = 2.8 Hz, 1H), 6.85 (dd, $^3J$ = 8.9, $^4J$ = 2.8 Hz, 1H), 7.01 (d, $^3J$ = 8.9 Hz, 1H), 7.14 (d, $^3J$ = 8.5 Hz, 1H), 7.61 (d, $^4J$ = 1.9 Hz, 1H), 7.71 (dd, $^3J$ = 8.4, $^4J$ = 1.9 Hz, 1H), 9.81 (s, 1H).

**$^{13}$C NMR (75 MHz, acetone-d$_6$):** $\delta$ 14.38 ($CH_3$), 14.39 ($CH_3$), 23.4 (2 $CH_2$), 26.78 ($CH_2$), 30.45 ($CH_2$), 30.46 ($CH_2$), 30.48 ($CH_2$), 30.49 ($CH_2$), 30.59 ($CH_2$), 30.60 ($CH_2$), 30.71 ($CH_2$), 30.74 ($CH_2$), 30.75 ($CH_2$), 30.76 ($CH_2$), 30.78 ($CH_2$), 30.97 ($CH_2$), 30.99 ($CH_2$), 32.00 ($CH_2$), 32.02 ($CH_2$), 32.64 ($CH_2$), 32.66 ($CH_2$), 35.5 (CH), 50.3 (2 $CH_2$), 52.3 ($CH_2$), 67.3 (2 $CH_2$), 115.3 (CH), 115.7 (CH), 116.3 (CH), 118.4 (CH), 126.1 ($C_{quat}$), 126.3 ($C_{quat}$), 128.7 (CH), 130.7 (CH), 131.9 ($C_{quat}$), 137.1 ($C_{quat}$), 149.3 ($C_{quat}$), 152.8 ($C_{quat}$), 190.3 (CH).

**MS (EI) m/z (%):** 649 ([M], 55), 325 ([$C_{18}H_{17}N_2O_2S$]$^+$, 35), 311 ([$C_{17}H_{15}N_2O_2S$]$^+$, 100), 57 ([$C_4H_9$]$^+$, 15).

**IR $\tilde{\nu}$ [cm$^{-1}$]**: 2953 (w), 2920 (s), 2851 (s), 2718 (w), 2162 (w), 1684 (s), 1603 (m), 1582 (m), 1553 (w), 1503 (m), 1474 (s), 1464 (s), 1451 (w), 1414 (w), 1375 (w), 1333 (w), 1310 (w), 1283 (w), 1254 (m), 1229 (s), 1198 (s), 1146 (w), 1123 (m), 1070 (w), 1061 (w), 1036 (w), 1026 (w), 1003 (w), 953 (s), 920 (w), 897 (w), 870 (w), 845 (w), 812 (m), 746 (m), 721 (m), 691 (w), 648 (w), 629 (w).

**Anal** calcd. for $C_{41}H_{64}N_2O_2S$ (649.0): C 75.87, H 76.03, N 4.32, S 4.94; Found: C 76.03, H 10.06, N 4.41, S 5.05.

### 4.2.1 General procedure V (GP5) for the *Heck*-coupling

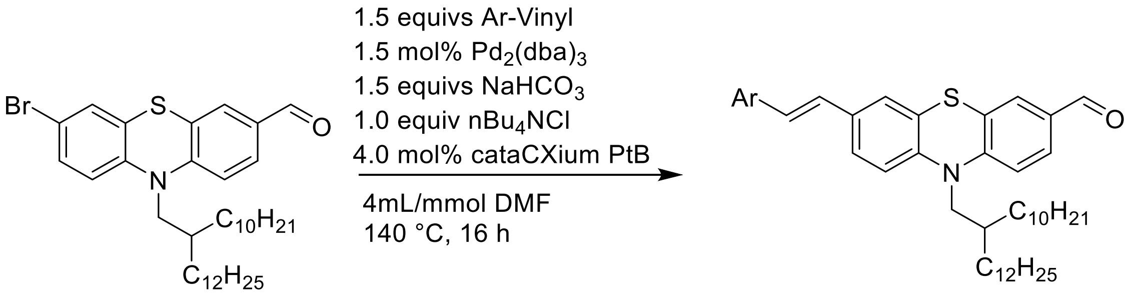


**1a** **2p-v**

Compound **1c** (1.0 equiv, 0.321 g, 0.5 mmol), $Pd_2(dba)_3$ (6.8 mg, 1.5 mol%), $NaHCO_3$ (1.5 equivs, 0.063 g, 0.75 mmol), *n*$Bu_4NCl$ (1.0 equiv, 0.139 g, 0.5 mmol), cataCXium*PtB (6.0 mg, 4.0 mol%) and the vinylated compound (1.5 equivs) were placed in a dry Schlenk tube under nitrogen atmosphere and dissolved in 2 mL of dry DMF. The reaction mixture was stirred for 16 h at 140 °C. The crude product was adsorbed on Celite® and purified via flash chromatography on silica gel (*n*-hexane/diethyl ether).

**Table S5.** Experimental details for the synthesis of vinylated phenothiazines **2p-v**.

| Entry | vinylated compound | Yield of **2** |
|---|---|---|
| 1 | 0.078 g (0.085 mL) of styrene | 463 mg (82%) of **2p** |
| 2 | 0.101 g (0.101 mL) of 4-methoxystyrene | 330 mg (78%) of **2q** |
| 3 | 0.096 g (0.096 mL) of 4-cyanostyrene | 291 mg (92%) of **2r** |
| 4 | 0.111 g (0.096 mL) of 4-nitrostyrene | 303 mg (86%) of **2s** |
| 5 | 0.129 g (0.110 mL) of 4-trifluoromethylstyrene | 294 mg (80%) of **2t** |

| | | |
|---|---|---|
| 6 | 0.203 g of *N*,*N*-diphenyl-4-vinylaniline | 312 mg (74%) of **2u** |
| 7 | 0.310 g 0-(2-decyltetradecyl)-3-vinyl-10*H*-phenothiazine | 899 mg (80%) of **2v** |

**(*E*)-10-(2-Decyltetradecyl)-7-styryl-10*H*-phenothiazine-3-carbaldehyde (2p)**

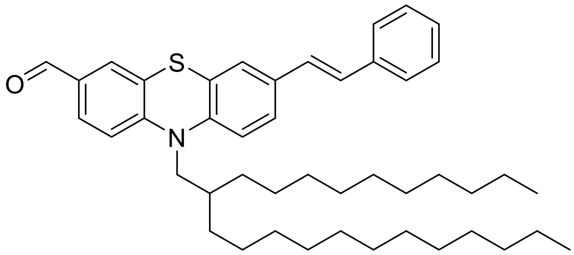

$C_{45}H_{63}NOS$
[666.07]

According to **GP5** and after flash chromatography on silica gel (*n*-hexane/diethyl ether 35:1) compound **2p** (271 mg, 82%) was obtained as a yellow oil.

**R*f*** (*n*-hexane/diethyl ether 30:1): 0.35.

**$^1$H NMR (300 MHz, acetone-d$_6$):** $\delta$ 0.81 – 0.92 (m, 6H), 1.13 – 1.51 (m, 40H), 1.98 – 2.04 (m, 1H), 3.99 (d, $^3J$ = 7.2 Hz, 2H), 7.13 (d, $^3J$ = 8.2 Hz, 1H), 7.17 – 7.19 (m, 2H), 7.20 – 7.29 (m, 2H), 7.32 – 7.40 (m, 2 H), 7.43 – 7.49 (m, 2 H), 7.55 – 7.61 (m, 2 H), 7.66 (d, $^4J$ = 1.9 Hz, 1H), 7.75 (dd, $^3J$ = 8.4, $^4J$ = 1.9 Hz, 1H), 9.85 (s, 1H).

**$^{13}$C NMR (75 MHz, acetone-d$_6$):** $\delta$ 14.38 ($CH_3$), 14.39 ($CH_3$), 23.4 (CH), 26.72 ($CH_2$), 26.76 ($CH_2$), 30.42 ($CH_2$), 30.45 ($CH_2$), 30.47 ($CH_2$), 30.49 ($CH_2$), 30.66 ($CH_2$), 30.68 ($CH_2$), 30.71 ($CH_2$), 30.75 ($CH_2$), 30.76 ($CH_2$), 30.78 ($CH_2$), 30.97 ($CH_2$), 30.98 ($CH_2$), 30.99 ($CH_2$), 31.9 ($CH_2$), 32.11 ($CH_3$), 32.67 ($CH_2$), 32.68 ($CH_2$), 52.5 ($CH_2$), 117.1 (CH), 118.1 ($C_{quat}$), 125.85 (CH), 125.9 (CH), 126.2 ($C_{quat}$), 127.1 (CH), 127.3 (2 CH), 127.9 (CH), 128.3 (CH), 128.7 (CH), 128.8 (CH), 129.5 (2 CH), 130.7 (CH), 132.6 ($C_{quat}$), 134.3 ($C_{quat}$), 138.4 ($C_{quat}$), 144.3 ($C_{quat}$), 151.9 ($C_{quat}$), 190.5 (CH).

**MS (EI) m/z (%):** 666 ([M], 80), 342 ($[C_{22}H_{16}NOS]^+$, 100), 328 ($[C_{21}H_{14}NOS]^+$, 45), 310 (30), 57 ($[C_4H_9]^+$, 25).

**IR $\tilde{\nu}$ [cm$^{-1}$]**: 3663 (w), 2990 (w), 2955 (w), 2920 (s), 2851 (m), 2722 (w), 1688 (s), 1599 (m), 1578 (s), 1564 (w), 1553 (w), 1495 (w), 1462 (s), 1404 (w), 1377 (w), 1337 (m), 1304 (m), 1281 (w), 1248 (m), 1198 (s), 1155 (w), 1146 (w), 1101 (w), 1057 (w), 1028 (w), 957 (m), 918 (w), 895 (w), 883 (w), 845 (w), 810 (m), 750 (m), 735 (w), 719 (m), 691 (s), 648 (w), 625 (w).

**Anal** calcd. for $C_{50}H_{64}N_4O_4S$ (666.1): C 81.15, H 9.53, N 2.10, S 4.81; Found: C 81.17, H 9.38, N 2.05, S 4.65.

**(*E*)-10-(2-Decyltetradecyl)-7-(4-methoxystyryl)-10*H*-phenothiazine-3-carbaldehyde (2q)**

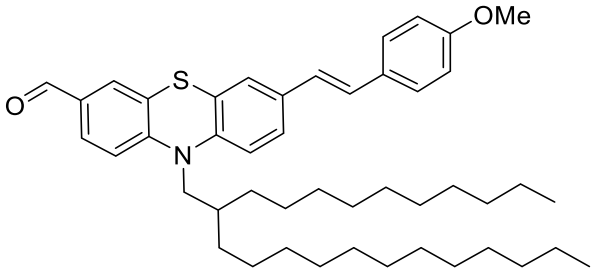

$C_{46}H_{65}NO_2S$
[696.09]

According to **GP5** and after flash chromatography on silica gel (*n*-hexane/diethyl ether 35:1) compound **2q** (277 mg, 78%) was obtained as a yellow oil.

**R*f*** (*n*-hexane/diethyl ether 20:1): 0.40.

**$^1$H NMR (600 MHz, acetone-d$_6$):** $\delta$ 0.84 – 0.90 (m, 6H), 1.19 – 1.41 (m, 40H), 2.00 – 2.03 (m, 1H), 3.98 (d, $^3J$ = 7.2 Hz, 2H), 6.93 (d, 2H), 7.01 (d, $^3J$ = 16.4 Hz, 1H), 7.10 (d, $^3J$ = 8.5 Hz, 1H), 7.13 (d, $^3J$ = 16.4 Hz, 1H), 7.21 (d, $^3J$ = 8.5 Hz, 1H), 7.40 (d, $^4J$ = 2.1 Hz, 1H), 7.42 (dd, $^3J$ = 8.5, $^4J$ = 2.1 Hz, 1H), 7.51 (d, $^3J$ = 8.6 Hz, 2H), 7.65 (d, $^4J$ = 1.9 Hz, 1H), 7.74 (dd, $^3J$ = 8.4, $^4J$ = 2.3 Hz, 1H), 9.85 (s, 1H).

**$^{13}$C NMR (150 MHz, acetone-d$_6$):** $\delta$ 14.37 ($CH_3$), 14.39 ($CH_3$), 23.4 (CH), 26.72 ($CH_2$), 26.76 ($CH_2$), 30.42 ($CH_2$), 30.45 ($CH_2$), 30.47 ($CH_2$), 30.49 ($CH_2$), 30.66 ($CH_2$), 30.68 ($CH_2$), 30.71 ($CH_2$), 30.75 ($CH_2$), 30.76 ($CH_2$), 30.78 ($CH_2$), 30.97 ($CH_2$), 30.98 ($CH_2$), 30.99 ($CH_2$), 31.9 ($CH_2$), 32.11 ($CH_3$), 32.67 ($CH_2$), 32.68 ($CH_2$), 52.4 ($CH_2$), 55.6 ($CH_3$), 115.0 (2 CH), 117.0 (CH), 118.1 (CH), 125.59 (CH), 125.64 (CH), 125.8 ($C_{quat}$), 126.3 ($C_{quat}$), 126.7 (CH), 128.4 (CH), 128.5 (2 CH), 128.8 (CH), 130.7 (CH), 131.1 ($C_{quat}$), 132.5 ($C_{quat}$), 134.7 ($C_{quat}$), 143.9 ($C_{quat}$), 152.0 ($C_{quat}$), 160.4 ($C_{quat}$), 190.5 (CH).

**MS (EI) m/z (%):** 692 ([M], 95), 372 ($[C_{23}H_{18}NO_2S]^+$, 85), 358 ($[C_{22}H_{16}NO_2S]^+$, 75), 340 (20), 57 ($[C_4H_9]^+$, 30).

**IR $\tilde{\nu}$ [cm$^{-1}$]**: 3015 (w), 2999 (w), 2922 (s), 2851 (s), 2716 (w), 1688 (s), 1599 (m), 1578 (m), 1564 (w), 1551 (w), 1510 (s), 1464 (s), 1443 (w), 1416 (w), 1404 (w), 1373 (w), 1339 (w), 1302 (w), 1290 (w), 1248 (s), 1198 (s), 1173 (m), 1146 (w), 1101 (w), 1036 (m), 1003 (w), 959 (w), 918 (w), 895 (w), 883 (w), 849 (w), 822 (s), 772 (w), 746 (w).

**Anal** calcd. for $C_{46}H_{65}NO_2S$ (696.1): C 79.37, H 9.41, N 2.01, S 4.61; Found: C 79.16, H 9.43, N 1.99, S 4.51.

**(*E*)-4-(2-(10-(2-Decyltetradecyl)-7-formyl-10*H*-phenothiazine-3-yl)vinyl)benzonitrile (2r)**

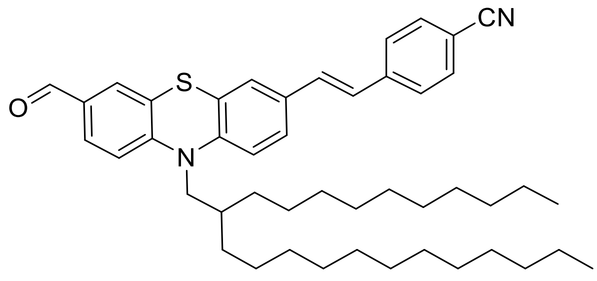

$C_{46}H_{62}N_2OS$
[691.08]

According to **GP5** and after flash chromatography on silica gel (*n*-hexane/diethyl ether 5:1) compound **2r** (290 mg, 82%) was obtained as a yellow oil.

**$R_f$** (*n*-hexane/diethyl ether 5:1): 0.30.

**$^1$H NMR (300 MHz, acetone-d$_6$):** $\delta$ 0.81 – 0.92 (m, 6H), 1.15 – 1.52 (m, 40H), 2.02 – 2.05 (m, 1H), 4.00 (d, $^3J$ = 7.2 Hz, 2H), 7.16 (d, $^3J$ = 8.4 Hz, 1H), 7.22 – 7.25 (m, 1H), 7.27 (d, $^3J$ = 16.4 Hz, 1H), 7.39 (d, $^3J$ = 16.4 Hz, 1H), 7.48 – 7.54 (m, 2H), 7.66 (d, $^4J$ = 1.9 Hz, 1H), 7.71 – 7.81 (m, 5H), 9.86 (s, 1H).

**$^{13}$C NMR (75 MHz, acetone-d$_6$):** $\delta$ 14.37 ($CH_3$), 14.39 ($CH_3$), 23.4 (CH), 26.72 ($CH_2$), 26.76 ($CH_2$), 30.42 ($CH_2$), 30.45 ($CH_2$), 30.47 ($CH_2$), 30.49 ($CH_2$), 30.66 ($CH_2$), 30.68 ($CH_2$), 30.71 ($CH_2$), 30.75 ($CH_2$), 30.76 ($CH_2$), 30.78 ($CH_2$), 30.97 ($CH_2$), 30.98 ($CH_2$), 30.99 ($CH_2$), 31.9 ($CH_2$), 32.11 ($CH_3$), 32.67 ($CH_2$), 32.68 ($CH_2$), 52.5 ($CH_2$), 111.1 ($C_{quat}$), 117.2 (CH), 118.2 (CH), 119.5 ($C_{quat}$), 126.0 ($C_{quat}$), 126.2 ($C_{quat}$), 126.3 (CH), 126.8 (CH), 127.8 (CH), 127.9 (2 CH), 128.8 (CH), 130.8 (CH), 131.7 (CH), 132.7 ($C_{quat}$), 133.3 (2 CH), 133.4 ($C_{quat}$), 143.1 ($C_{quat}$), 145.2 ($C_{quat}$), 151.7 ($C_{quat}$), 190.5 (CH).

**MS (EI) m/z (%):** 691 ([M], 70), 367 ($[C_{23}H_{15}N_2OS]^+$, 100), 353 ($[C_{22}H_{13}N_2OS]^+$, 45), 335 (40), 57 ($[C_4H_9]^+$, 15).

**IR $\tilde{\nu}$ [cm$^{-1}$]**: 2949 (w), 2922 (s), 2851 (s), 2725 (w), 2222 (m), 1684 (s), 1634 (w), 1597 (s), 1578 (s), 1551 (m), 1506 (m), 1464 (s), 1416 (w), 1344 (m), 1283 (m), 1263 (w), 1250 (w), 1219 (w), 1196 (s), 1173 (m), 1146 (w), 1101 (w), 968 (m), 918 (w), 905 (w), 893 (w), 868 (w), 824 (s), 806 (w), 756 (w), 725 (w), 712 (w), 683 (w), 648 (w).

**Anal** calcd. for $C_{46}H_{62}N_2OS$ (691.1): C 79.95, H 9.04, N 4.05, S 4.64; Found: C 79.89, H 9.26, N 3.86, S 4.39.

**(*E*)-10-(2-Decyltetradecyl)-7-(4-nitrostyryl)-10*H*-phenothiazine-3-carbaldehyde (2s)**

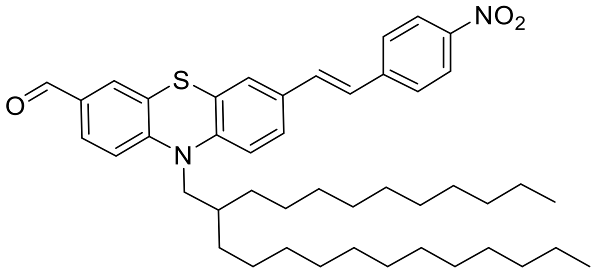


$C_{45}H_{62}N_2O_3S$
[711.06]

According to **GP5** and after flash chromatography on silica gel (*n*-hexane/diethyl ether 5:1) compound **2s** (303 mg, 86%) was obtained as a red oil.

**R$_f$ (*n*-hexane/diethyl ether 5:1):** 0.35

**$^1$H NMR (300 MHz, acetone-d$_6$):** *δ* 0.80 – 0.91 (m, 6H), 1.17 – 1.48 (m, 40H), 1.98 – 2.04 (m, 1H), 4.01 (d, $^3J$ = 7.2 Hz, 2H), 7.15 – 7.20 (m, 1H), 7.25 (d, $^3J$ = 8.4 Hz, 1H), 7.35 (d, $^3J$ = 16.4 Hz, 1H), 7.46 (d, $^3J$ = 16.4 Hz, 1H), 7.52 – 7.57 (m, 2H), 7.67 (d, $^4J$ = 1.9 Hz, 1H), 7.77 (dd, $^3J$ = 8.4, $^4J$ = 1.9 Hz, 1H), 7.84 (d, $^3J$ = 8.9 Hz, 2H), 8.23 (d, $^3J$ = 9.1 Hz, 2H), 9.86 (s, 1H).

**$^{13}$C NMR (75 MHz, acetone-d$_6$):** *δ* 14.38 ($CH_3$), 14.40 ($CH_3$), 23.4 (CH), 26.72 ($CH_2$), 26.76 ($CH_2$), 30.42 ($CH_2$), 30.45 ($CH_2$), 30.47 ($CH_2$), 30.49 ($CH_2$), 30.66 ($CH_2$), 30.68 ($CH_2$), 30.71 ($CH_2$), 30.75 ($CH_2$), 30.76 ($CH_2$), 30.78 ($CH_2$), 30.97 ($CH_2$), 30.98 ($CH_2$), 30.99 ($CH_2$), 31.9 ($CH_2$), 32.11 ($CH_3$), 32.67 ($CH_2$), 32.68 ($CH_2$), 52.2 ($CH_2$), 117.2 (CH), 118.2 ($C_{quat}$), 124.8 (2 CH), 126.0 ($C_{quat}$), 126.1 ($C_{quat}$), 126.3 (CH), 126.4 (CH), 126.6 (CH), 127.9 (2 CH), 128.0 (CH), 128.9 (CH), 130.8 (CH), 132.7 (CH), 132.8 ($C_{quat}$), 133.3 ($C_{quat}$), 145.3 ($C_{quat}$), 145.4 ($C_{quat}$), 147.5 ($C_{quat}$), 190.5 (CH).

**MS (EI) m/z (%):** 711 ([M], 65), 387 ($[C_{22}H_{15}N_2O_3S]^+$, 100), 373 ($[C_{21}H_{13}N_2O_3S]^+$, 30), 355 (20), 57 ($[C_4H_9]^+$, 25).

**IR $\tilde{\nu}$ [cm$^{-1}$]**: 2951 (w), 2922 (m), 2851 (m), 1684 (s), 1591 (w), 1576 (m), 1564 (w), 1551 (w), 1504 (m), 1460 (s), 1335 (s), 1325 (w), 1285 (m), 1265 (w), 1250 (w), 1219 (w), 1196 (s), 1163 (w), 1148 (w), 1107 (m), 968 (w), 918 (w), 905 (w), 864 (w), 837 (w), 818 (w), 808 (w), 746 (w), 719 (w), 691 (w).

**Anal** calcd. for $C_{45}H_{62}N_2O_3S$ (711.1): C 76.01, H 8.79, N 4.07, S 4.51; Found: C 76.31, H 9.15, N 3.94, S 4.47.

**(*E*)-10-(2-Decyltetradecyl)-7-(4-(trifluoromethyl)styryl)-10*H*-phenothiazine-3-carbaldehyde (2t)**

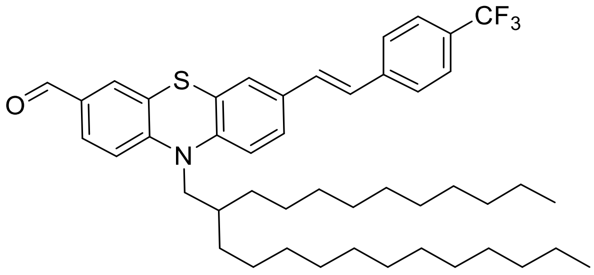

$C_{46}H_{62}F_3NOS$
[734.06]

According to **GP5** and after flash chromatography on silica gel (*n*-hexane/diethyl ether 25:1) compound **2t** (294 mg, 80%) was obtained as a red oil.

**R*f*** (*n*-hexane/diethyl ether 25:1): 0.30

**$^1$H NMR (300 MHz, acetone-d$_6$):** 0.81 – 0.92 (m, 6H), 1.14 – 1.50 (m, 40H), 1.97 – 2.03 (m, 1 H), 3.98 (d, $^3J$ = 7.2 Hz, 2H), 7.14 (d, $^3J$ = 8.3 Hz, 1H), 7.22 (d, $^3J$ = 8.6 Hz, 1H), 7.30 (d, $^3J$ = 9.1 Hz, 1H), 7.46 – 7.53 (m, 2H), 7.64 – 7.72 (m, 3H), 7.73 – 7.83 (m, 4H), 9.85 (s, 1H).

**$^{13}$C NMR (75 MHz, acetone-d$_6$):** $\delta$ 14.38 ($CH_3$), 14.40 ($CH_3$), 23.3 (CH), 26.70 ($CH_2$), 26.74 ($CH_2$), 30.42 ($CH_2$), 30.45 ($CH_2$), 30.47 ($CH_2$), 30.49 ($CH_2$), 30.66 ($CH_2$), 30.68 ($CH_2$), 30.71 ($CH_2$), 30.75 ($CH_2$), 30.76 ($CH_2$), 30.78 ($CH_2$), 30.97 ($CH_2$), 30.98 ($CH_2$), 30.99 ($CH_2$), 31.9 ($CH_2$), 32.11 ($CH_3$), 32.66 ($CH_2$), 32.67 ($CH_2$), 35.5 (CH), 52.5 ($CH_2$), 117.1 (CH), 118.1 (CH), 125.9 ($C_{quat}$), 126.2 (CH), 126.2 ($C_{quat}$), 126.4 ($CF_3$), 126.9 (CH), 127.6 (2 CH), 127.7 (CH), 128.8 (CH), 129.0 ($C_{quat}$), 130.78 (2 CH), 130.82 (CH), 132.7 ($C_{quat}$), 133.6 ($C_{quat}$), 142.5 ($C_{quat}$), 144.9 ($C_{quat}$), 151.7 ($C_{quat}$), 190.4 (CH).

**MS (EI) m/z (%):** 734 ([M], 75), 410 ($[C_{23}H_{15}F_3NOS]^+$, 100), 397 ($[C_{22}H_{13}F_3NOS]^+$, 35), 378 (30), 173 (30).

**IR $\tilde{\nu}$ [cm$^{-1}$]**: 2922 (m), 2853 (m), 2725 (w), 1688 (m), 1612 (w), 1599 (m), 1578 (m), 1553 (w), 1499 (w), 1462 (s), 1416 (w), 1373 (w), 1321 (s), 1283 (w), 1248 (w), 1196 (m), 1186 (w), 1161 (w), 1107 (s), 1067 (s), 1043 (w), 1015 (m), 968 (w), 949 (w), 918 (w), 895 (w), 864 (w), 826 (m), 812 (w), 739 (w), 718 (m), 685 (w), 658 (w).

**Anal** calcd. for $C_{46}H_{62}F_3NOS$ (734.1): C 75.27, H 8.51, N 1.91, S 4.37; Found: C 75.23, H 8.79, N 1.87, S 4.29.

**(*E*)-10-(2-Decyltetradecyl)-7-(4-(diphenylamino)styryl)-10*H*-phenothiazine-3-carbaldehyde (2u)**

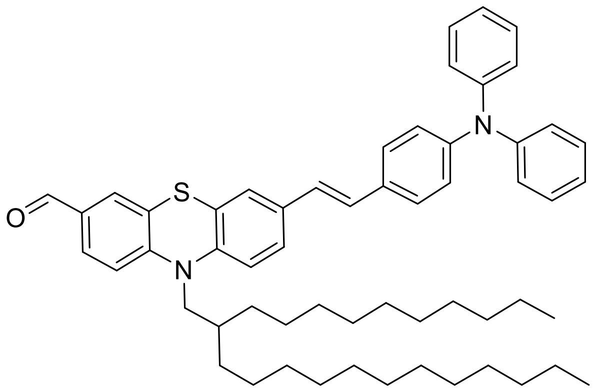

$C_{57}H_{72}N_2OS$
[833.28]

According to **GP5** and after flash chromatography on silica gel (*n*-hexane/diethyl ether 35:1) compound **2u** (312 mg, 74%) was obtained as a yellow oil.

**R*f*** (*n*-hexane/diethyl ether 35:1): 0.30

**$^1$H NMR (300 MHz, acetone-d$_6$):** $\delta$ 0.82 – 0.93 (m, 6H), 1.16 – 1.53 (m, 40H), 2.06 - 2.08 (m, 1H), 3.98 (d, $^3J$ = 7.2 Hz, 1H), 6.96 – 7.04 (m, 2H), 7.05 – 7.15 (m, 9H), 7.22 (d, $^3J$ = 8.5 Hz, 1H), 7.27 – 7.36 (m, 4H), 7.39 – 7.45 (m, 2H), 7.46 – 7.53 (m, 2H), 7.65 (d, $^4J$ = 1.9 Hz, 1H), 7.75 (dd, $^3J$ = 8.4, $^4J$ = 1.9 Hz, 1H), 9.85 (s, 1H).

**$^{13}$C NMR (75 MHz, acetone-d$_6$):** $\delta$ 14.39 ($CH_3$), 14.40 ($CH_3$), 23.4 (CH), 26.74 ($CH_2$), 26.78 ($CH_2$), 30.42 ($CH_2$), 30.45 ($CH_2$), 30.47 ($CH_2$), 30.49 ($CH_2$), 30.66 ($CH_2$), 30.68 ($CH_2$), 30.71 ($CH_2$), 30.75 ($CH_2$), 30.76 ($CH_2$), 30.78 ($CH_2$), 30.97 ($CH_2$), 30.98 ($CH_2$), 30.99 ($CH_2$), 31.9 ($CH_2$), 32.11 ($CH_3$), 32.66 ($CH_2$), 32.67 ($CH_2$), 35.4 (CH), 52.5 ($CH_2$), 117.0 (CH), 118.1 ($C_{quat}$), 123.9 (CH), 124.03 (CH), 124.06 (2 CH), 124.3 (2 CH), 125.26 (4 CH), 125.7 (CH), 125.8 ($C_{quat}$), 126.9 (CH), 128.2 (CH), 128.3 (2 CH), 130.3 (4 CH), 130.6 (CH), 130.7 (CH), 132.5 ($C_{quat}$), 132.8 ($C_{quat}$), 134.6 ($C_{quat}$), 144.0 ($C_{quat}$), 148.2 ($C_{quat}$), 148.5 (2 $C_{quat}$), 151.9 ($C_{quat}$), 190.4 (CH).

**MS (EI) m/z (%):** 833 ([M], 2), 591 (55), 268 (100), 255 (50), 236 (30).

**IR $\tilde{\nu}$ [cm$^{-1}$]**: 3026 (w), 2922 (s), 2851 (m), 2722 (w), 1688 (s), 1589 (s), 1578 (w), 1564 (w), 1506 (s), 1493 (s), 1464 (s), 1416 (w), 1404 (w), 1373 (w), 1329 (m), 1312 (m), 1288 (s), 1198 (s), 1177 (w), 1153 (w), 1146 (w), 1101 (w), 1074 (w), 1028 (w), 999 (w), 959 (m), 941 (w), 918 (w), 895 (w), 881 (w), 853 (w), 820 (s), 752 (s), 723 (s), 694 (s).

**Anal** calcd. for $C_{57}H_{72}N_2OS$ (833.3): C 82.16, H 8.71, N 3.36, S 3.85; Found: C 82.11, H 8.79, N 3.27, S 3.87.

**(*E*)-10-(2-decyltetradecyl)-7-(2-(10-(2-decyltetradecyl)-10*H*-phenothiazin-3-yl)vinyl)-10*H*-phenothiazine-3-carbaldehyde (2v)**

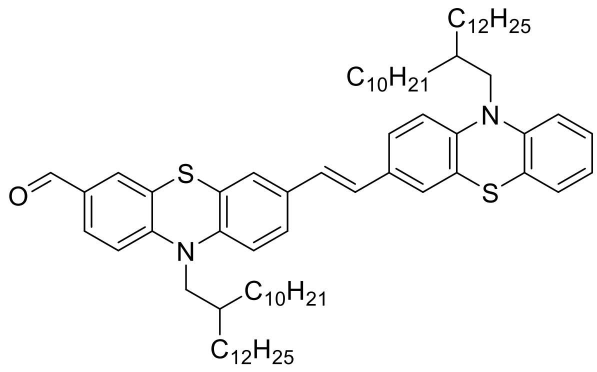

$C_{75}H_{114}N_2OS_2$
[1123.87]

According to **GP5** and after flash chromatography on silica gel (*n*-hexane/diethyl ether 30:1) compound **2v** (899 mg, 80%) was obtained as a yellow oil.

**R*f*** (*n*-hexane/diethyl ether 30:1): 0.30

**$^1$H NMR (300 MHz, acetone-d$_6$):** 0.82 – 0.92 (m, 12H), 1.16 – 1.53 (m, 80H), 1.97 – 2.03 (m, 1H), 3.86 (d, $^3J$ = 7.1 Hz, 2H), 3.95 (d, $^3J$ = 7.2 Hz, 2H), 6.91 – 6.99 (m, 1H), 7.00 – 7.10 (m, 4H), 7.13 – 7.23 (m, 3H), 7.34 – 7.42 (m, 3H), 7.63 (d, $^3J$ = 1.9 Hz, 1H), 7.73 (dd, $^3J$ = 8.4, $^4J$ = 1.9 Hz, 1H), 9.84 (s, 1H).

**$^{13}$C NMR (75 MHz, acetone-d$_6$):** $\delta$ 14.4 (4 $CH_3$), 23.4 (4 $CH_2$), 26.7 (2 $CH_2$), 26.8 ($CH_2$), 26.8 (2 $CH_2$), 26.9 (2 $CH_2$), 31.9 (2 $CH_2$), 32.1 (4 $CH_2$), 32.7 (4 $CH_2$), 35.3 ($CH_2$), 35.4 ($CH_2$), 52.1 ($CH_2$), 52.5 ($CH_2$), 117.0 (CH), 117.2 (CH), 118.1 ($C_{quat}$), 123.4 (CH), 125.66 ($C_{quat}$), 125.69 ($C_{quat}$), 125.8 (CH), 125.9 (CH), 126.2 (CH), 126.3 (CH), 126.7 (2 CH), 126.9 (CH), 127.5 ($C_{quat}$), 128.2 (CH), 128.27 (CH), 128.34 (CH), 128.8 (CH), 130.7 (CH), 132.5 ($C_{quat}$), 133.1 ($C_{quat}$), 134.5 ($C_{quat}$), 144.0 ($C_{quat}$), 145.9 ($C_{quat}$), 146.5 ($C_{quat}$), 151.9 ($C_{quat}$), 190.4 (CH).

**MS MALDI:** 1123.17 [M]

**IR $\tilde{\nu}$ [cm$^{-1}$]:** 3015 (w), 2920 (s), 2851 (m), 2718 (w), 1692 (m), 1643 (w), 1597 (w), 1578 (w), 1564 (w), 1555 (w), 1499 (w), 1460 (s), 1443 (w), 1412 (w), 1400 (w), 1375 (w), 1339 (m), 1308 (w), 1285 (w), 1248 (m), 1198 (s), 1161 (w), 1146 (w), 1132 (w), 1101 (w), 1038 (w), 999 (w), 955 (w), 920 (w), 895 (w), 876 (w), 845 (w), 814 (m), 745 (s), 719 (m), 685 (w), 640 (w), 633 (w), 615 (w).

**MS(ESI-HRMS) m/z** calcd. for [$C_{75}H_{114}N_2OS_2$+$H^+$] = 1123.8445; Found: 1123.8427.

## 2.2 Synthesis and analytical data of the monomers 3

### 2.2.1 General procedure VI (GP6) for the Ohira-Bestmann reaction

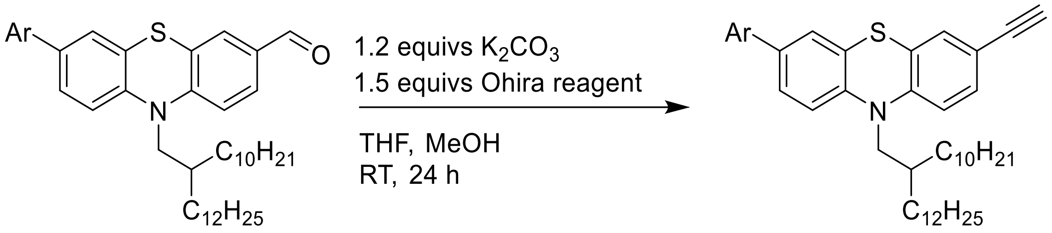


**2a-v** **3a-v**

Aldehyde **2** (1.0 equiv) and $K_2CO_3$ (1.2 equivs) were placed in a dry Schlenk tube and dissolved in 5 mL/mmol dry THF and 5 mL/mmol dry methanol. After addition of dimethyl (1-diazo-2-oxopropyl)-phosphonate (Ohira-Bestmann reagent) (1.5 equivs) under nitrogen atmosphere the solution was stirred at room temp for 18 h. The reaction mixture was diluted in water and diethyl ether and washed three times with dichloromethane. The organic phases were dried with anhydrous magnesium sulfate and filtered. The crude product was adsorbed on Celite® and purified via flash chromatography on silica gel.

**Table S6.** Experimental details for the synthesis of alkynylated phenothiazines **3a-v**.

| entry | aldehyde **2** | $K_2CO_3$ | Ohira-Bestmann reagent | THF | MeOH | **3** |
|---|---|---|---|---|---|---|
| 1 | 0.500 g (0.78 mmol) of **2a** | 0.129 g (0.94 mmol) | 0.26 mL (1.17 mmol) | 3.9 mL | 3.9 mL | 0.210 g (40%) of **3a** |
| 2 | 0.400 g (0.60 mmol) of **2b** | 0.100 g (0.72 mmol) | 0.14 mL (0.90 mmol) | 3.0 mL | 3.0 mL | 0.250 g (62%) of **3b** |
| 3 | 0.300 g (0.45 mmol) of **2c** | 0.074 g (0.54 mmol) | 0.10 mL (0.68 mmol) | 2.3 mL | 2.3 mL | 0.100 g (30%) of **3c** |
| 4 | 0.300 g (0.44 mmol) of **2d** | 0.073 g (0.53 mmol) | 0.09 mL (0.60 mmol) | 2.2 mL | 2.2 mL | 0.180 g (60%) of **3d** |
| 5 | 0.400 g (0.56 mmol) of **2e** | 0.092 g (0.67 mmol) | 0.13 mL (0.84 mmol) | 2.8 mL | 2.8 mL | 0.240 g (61%) of **3e** |
| 6 | 0.316 g (0.40 mmol) of **2f** | 0.110 g (0.80 mmol) | 0.09 mL (0.61 mmol) | 2.0 mL | 2.0 mL | 0.120 g (38%) of **3f** |

| | | | | | | |
|---|---|---|---|---|---|---|
| 7 | 0.600 g (0.69 mmol) of **2g** | 0.114 g (0.83 mmol) | 0.16 mL (1.03 mmol) | 3.5 mL | 3.5 mL | 0.300 g (50%) of **3g** |
| 8 | 0.400 g (0.58 mmol) of **2h** | 0.096 g (0.63 mmol) | 0.13 mL (0.87 mmol) | 2.9 mL | 2.9 mL | 0.130 g (33%) of **3h** |
| 9 | 0.170 g (0.22 mmol) of **2i** | 0.036 g (0.26 mmol) | 0.05 mL (0.33 mmol) | 1.1 mL | 1.1 mL | 0.050 g (29%) of **3i** |
| 10 | 0.346 g (0.46 mmol) of **2j** | 0.075 g (0.54 mmol) | 0.10 mL (0.68 mmol) | 2.3 mL | 2.3 mL | 0.170 g (50%) of **3j** |
| 11 | 0.180 g (0.25 mmol) of **2k** | 0.042 g (0.31 mmol) | 0.06 mL (0.38 mmol) | 1.3 mL | 1.3 mL | 0.052 g (28%) of **3k** |
| 12 | 0.407 g (0.50 mmol) of **2l** | 0.083 g (0.61 mmol) | 0.11 mL (0.75 mmol) | 2.5 mL | 2.5 mL | 0.183 g (83%) of **3l** |
| 13 | 0.407 g (0.50 mmol) of **2m** | 0.083 g (0.61 mmol) | 0.11 mL (0.75 mmol) | 2.5 mL | 2.5 mL | 0.205 g (56%) of **3m** |
| 14 | 0.409 g (0.50 mmol) of **2n** | 0.083 g (0.61 mmol) | 0.11 mL (0.75 mmol) | 2.5 mL | 2.5 mL | 0.090 g (22%) of **3n** |
| 15 | 0.182 g (0.28 mmol) of **2o** | 0.047 g (0.34 mmol) | 0.06 mL (0.42 mmol) | 1.4 mL | 1.4 mL | 0.101 g (55%) of **3o** |
| 16 | 0.280 g (0.42 mmol) of **2p** | 0.070 g (0.51 mmol) | 0.09 mL (0.68 mmol) | 2.1 mL | 2.1 mL | 0.098 g (35%) of **3p** |
| 17 | 0.300 g (0.43 mmol) of **2q** | 0.072 g (0.52 mmol) | 0.10 mL (0.69 mmol) | 2.2 mL | 2.2 mL | 0.190 g (63%) of **3q** |
| 18 | 0.250 g (0.36 mmol) of **2r** | 0.060 g (0.43 mmol) | 0.08 mL (0.54 mmol) | 1.8 mL | 1.8 mL | 0.092 g (37%) of **3r** |

| | | | | | | |
|---|---|---|---|---|---|---|
| 19 | 0.330 g (0.48 mmol) of **2s** | 0.080 g (0.58 mmol) | 0.11 mL (0.72 mmol) | 2.4 mL | 2.4 mL | 0.083 g (25%) of **3s** |
| 20 | 0.428 g (0.58 mmol) of **2t** | 0.097 g (0.70 mmol) | 0.13 mL (0.87 mmol) | 2.9 mL | 2.9 mL | 0.155 g (38%) of **3t** |
| 21 | 0.250 g (0.30 mmol) of **2u** | 0.050 g (0.36 mmol) | 0.07 mL (0.45 mmol) | 1.5 mL | 1.5 mL | 0.050 g (22%) of **3u** |
| 22 | 0.500 g (0.44 mmol) of **2v** | 0.073 g (0.53 mmol) | 0.10 mL (0.66 mmol) | 2.2 mL | 2.2 mL | 0.132 g (27%) of **3v** |

**10-(2-Decyltetradecyl)-3-ethynyl-7-phenyl-10*H*-phenothiazine (3a)**

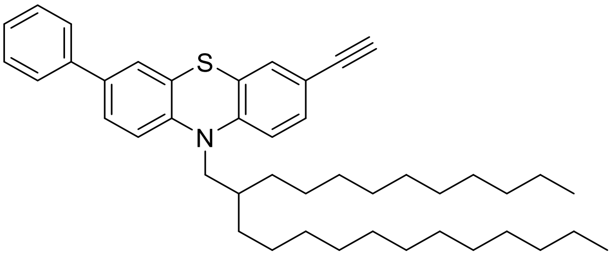

$C_{44}H_{61}NS$
[636.04]

According to **GP6** and after flash chromatography on silica gel (*n*-hexane/diethyl ether 20:1) compound **3a** (210 mg, 40%) was obtained as a yellow oil.

**R*f*** (*n*-hexane/diethyl ether 20:1): 0.38.

**$^1$H NMR (300 MHz, acetone-d$_6$):** $\delta$ 0.81 – 0.94 (m, 6H), 1.19 – 1.47 (m, 40H), 1.98 – 2.02 (m, 1H), 3.59 (s, 1H), 3.91 (d, $^3J$ = 7.2 Hz, 2H), 7.05 (d, $^3J$ = 8.4 Hz, 1H), 7.14 (d, $^3J$ = 8.4 Hz, 1H), 7.26 (d, $^4J$ = 1.9 Hz, 1H), 7.30 – 7.35 (m, 2H), 7.39 – 7.47 (m, 3H), 7.51 (dd, $^3J$ = 8.4, $^4J$ = 1.9 Hz, 1H), 7.60 – 7.65 (m, 2H).

**$^{13}$C NMR (75 MHz, acetone-d$_6$):** $\delta$ 14.38 ($CH_3$), 14.39 ($CH_3$), 23.4 (2 $CH_2$), 26.91 ($CH_2$), 26.93 ($CH_2$), 30.45 ($CH_2$), 30.46 ($CH_2$), 30.48 ($CH_2$), 30.49 ($CH_2$), 30.66 ($CH_2$), 30.68 ($CH_2$), 30.71 (CH2), 30.74 ($CH_2$), 30.75 ($CH_2$), 30.76 ($CH_2$), 30.78 ($CH_2$), 30.97 ($CH_2$), 30.98 ($CH_2$), 30.99 ($CH_2$), 31.5 ($CH_3$), 32.68 ($CH_2$), 32.69 ($CH_2$), 33.1 (CH), 35.3 ($CH_2$), 52.2 ($CH_2$), 83.5 (CH), 84.2 ($C_{quat}$) 117.06 (CH), 117.12 ($C_{quat}$), 117.8 (CH), 126.18 ($C_{quat}$), 126.2 (CH), 126.3 (CH), 126.9 (CH), 127.2 (2 CH), 128.0 (CH), 129.7 (2 CH), 131.2 (CH), 132.2 (CH), 136.7 ($C_{quat}$), 140.5 ($C_{quat}$), 145.3 ($C_{quat}$), 147.1 ($C_{quat}$).

**MS (EI) m/z (%):** 636 ([M], 100), 312 ($[C_{21}H_{14}NS]^+$, 40), 298 ($[C_{20}H_{12}NS]^+$, 30), 280 (18), ), 57 ($[C_4H_9]^+$, 8).

**IR $\tilde{\nu}$ [cm$^{-1}$]**: 3306 (w), 3030 (w), 2953 (w), 2920 (s), 2851 (m), 2108 (w), 1601 (w), 1581 (w), 1506 (w), 1460 (s), 1396 (w), 1377 (w), 1337 (m), 1298 (w), 1275 (w), 1248 (m), 1198 (w), 1180 (w), 1163 (w), 1146 (w), 1105 (w), 1074 (w), 1051 (w), 1022 (w), 883 (m), 849 (w), 818 (m), 793 (w), 760 (s), 739 (w), 719 (w), 696 (s), 675 (w), 648 (w).

**Anal.** calcd for $C_{44}H_{61}NS$ (636.0): C 83.09, H 9.67, N 2.20, S 5.04; Found: C 83.36, H 9.88, N 2.14, S 5.29.

**10-(2-Decyltetradecyl)-3-ethynyl-7-(4-methoxyphenyl)-10*H*-phenothiazin (3b)**

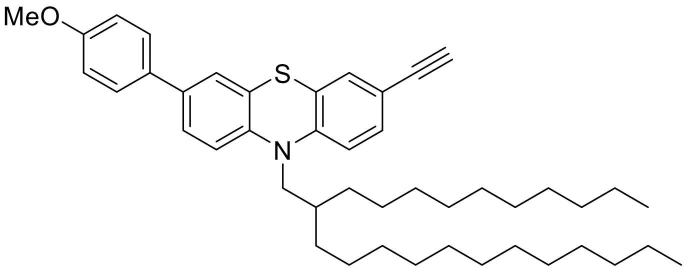


$C_{45}H_{63}NOS$
[666.07]

According to **GP6** and after flash chromatography on silica gel (*n*-hexane/diethyl ether 20:1) compound **3b** (250 mg, 62%) was obtained as a yellow oil.

**R*f*** (*n*-hexane/diethyl ether 20:1): 0.35.

**$^1$H NMR (600 MHz, acetone-d$_6$):** $\delta$ 0.87 (m, 6H), 1.20 – 1.47 (m, 40H), 1.99 – 2.03 (m, 1H), .59 (s, 1H), 3.83 (s, 3H), 3.90 (d, $^3J$ = 7.2 Hz, 2H), 6.99 (d, $^3J$ = 8.8 Hz, 2H), 7.04 (d, $^3J$ = 8.5 Hz, 1H), 7.11 (d, $^3J$ = 8.5 Hz, 1H), 7.26 (d, $^4J$ = 1.9 Hz, 1H), 7.32 (dd, $^3J$ = 8.4, $^4J$ = 2.0 Hz, 1H), 7.39 (d, $^4J$ = 2.2 Hz, 1H), 7.45 (dd, $^3J$ = 8.4, $^4J$ = 2.2 Hz, 1H), 7.56 (d, $^3J$ = 8.7 Hz, 2H).

**$^{13}$C NMR (75 MHz, acetone-d$_6$):** $\delta$ 14.38 ($CH_3$), 14.39 ($CH_3$), 23.4 (2 $CH_2$), 26.91 ($CH_2$), 26.93 ($CH_2$), 30.45 ($CH_2$), 30.46 ($CH_2$), 30.48 ($CH_2$), 30.49 ($CH_2$), 30.66 ($CH_2$), 30.68 ($CH_2$), 30.71 (CH2), 30.74 ($CH_2$), 30.75 ($CH_2$), 30.76 ($CH_2$), 30.78 ($CH_2$), 30.97 ($CH_2$), 30.98 ($CH_2$), 30.99 ($CH_2$), 31.5 ($CH_3$), 32.68 ($CH_2$), 32.69 ($CH_2$), 33.1 (CH), 35.3 ($CH_2$), 52.2 ($CH_2$), 54.7 ($CH_3$), 78.8 (CH), 83.6 ($C_{quat}$), 115.1 (2 CH), 117.0 (CH), 117.7 (CH), 125.9 (CH), 126.1 ($C_{quat}$), 126.3 ($C_{quat}$), 126.4 (CH), 128.2 (2 CH), 130.3 (CH), 131.2 (CH), 132.2 ($C_{quat}$), 132.9 ($C_{quat}$), 136.5 ($C_{quat}$), 144.6 ($C_{quat}$), 147.3 ($C_{quat}$), 160.2 ($C_{quat}$).

**MS (EI) m/z (%):** 666 ([M], 100), 342 ($[C_{22}H_{16}NOS]^+$, 75), 328 ($[C_{21}H_{14}NOS]^+$, 55), 310 (25), 57 ($[C_4H_9]^+$, 15).

**IR $\tilde{\nu}$ [cm$^{-1}$]**: 3304 (w), 3034 (w), 2997 (w), 2953 (w), 2922 (s), 2851 (s), 2108 (w), 1271 (w), 1609 (w), 1582 (w), 1518 (w), 1495 (m), 1460 (s), 1422 (w), 1393 (w), 1377 (w), 1339 (m), 1308 (w), 1290 (w), 1261 (w), 1244 (s), 1179 (m), 1163 (w), 1146 (w), 1103 (w), 1043 (w), 1028 (w), 1009 (w), 980 (w), 939 (w), 883 (m), 814 (s), 766 (w), 721 (w), 696 (w), 664 (w), 648 (m).

**Anal** calcd for $C_{45}H_{63}NOS$ (666.1): C 81.15, H 9.53, N 2.10, S 4.81; Found: C 80.97, H 9.63, N 1.98, S 4.56.

**4-(10-(2-Decyltetradecyl)-7-ethynyl-10*H*-phenothiazine-3-yl)benzonitrile (3c)**

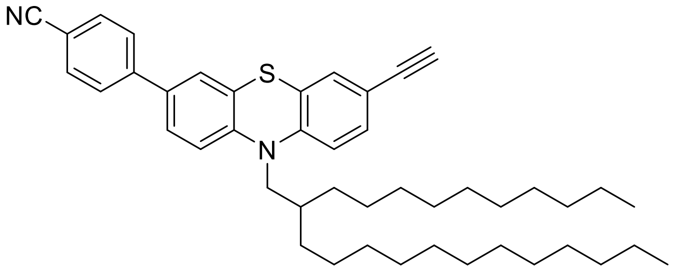

$C_{58}H_{74}N_2O_2S$
[661.05]

According to **GP6** and after flash chromatography on silica gel (*n*-hexane/diethyl ether 15:1) compound **3c** (100 mg, 30%) was obtained as an orange oil.

**R***f* (*n*-hexane/diethyl ether 15:1): 0.35.

**$^1$H NMR (600 MHz, acetone-d$_6$):** $\delta$ 0.83 – 0.90 (m, 6H), 1.17 – 1.39 (m, 40H), 2.03 – 2.06 (m, 1H), 3.61 (s, 1H), 3.94 (d, $^3J$ = 7.2 Hz, 2H), 7.08 (d, $^3J$ = 8.4 Hz, 1H), 7.20 (d, $^3J$ = 8.5 Hz, 1H), 7.27 (d, $^4J$ = 1.9 Hz, 1H), 7.34 (dd, $^3J$ = 8.4, $^4J$ =1.9 Hz, 1H), 7.55 (d, $^4J$ = 2.2 Hz, 1H), 7.62 (dd, $^3J$ = 8.5, $^4J$ = 2.2 Hz, 1H), 7.82 (d, $^3J$ = 8.2 Hz, 2H), 7.87 (d, $^3J$ = 8.3 Hz, 2H).

**$^{13}$C NMR (75 MHz, acetone-d$_6$):** $\delta$ 14.38 ($CH_3$), 14.39 ($CH_3$), 23.4 (2 $CH_2$), 26.91 ($CH_2$), 26.93 ($CH_2$), 30.45 ($CH_2$), 30.46 ($CH_2$), 30.48 ($CH_2$), 30.49 ($CH_2$), 30.66 ($CH_2$), 30.68 ($CH_2$), 30.71 (CH2), 30.74 ($CH_2$), 30.75 ($CH_2$), 30.76 ($CH_2$), 30.78 ($CH_2$), 30.97 ($CH_2$), 30.98 ($CH_2$), 30.99 ($CH_2$), 31.5 ($CH_3$), 32.68 ($CH_2$), 32.69 ($CH_2$), 33.1 (CH), 35.3 ($CH_2$), 52.2 ($CH_2$), 79.1 (CH), 83.5 ($C_{quat}$), 111.3 ($C_{quat}$), 117.3 (CH), 117.4 ($C_{quat}$), 117.9 (CH), 119.4 ($C_{quat}$), 126.0 ($C_{quat}$), 126.5 ($C_{quat}$), 126.6 (CH), 127.3 (CH), 127.9 (2 CH), 131.2 (CH), 132.3 (CH), 133.5 (2 CH), 134.4 ($C_{quat}$), 144.8 ($C_{quat}$), 146.6 ($C_{quat}$), 146.8 ($C_{quat}$).

**MS (EI) m/z (%):** 661 ([M], 100), 337 ($[C_{22}H_{13}N_2S]^+$,95), 323 ($[C_{21}H_{11}N_2S]^+$, 40), 305 (35), 57 ($[C_4H_9]^+$, 20).

**IR $\tilde{\nu}$ [cm$^{-1}$]**: 2953 (w), 2922 (s), 2851 (s), 2797 (w), 2735 (w), 1682 (s), 1593 (m), 1578 (s), 1553 (m), 1510 (s), 1456 (s), 1420 (w), 1389 (w), 1377 (w), 1337 (s), 1310 (w), 1287 (m), 1196 (s), 1111 (m), 920 (w), 887 (w), 856 (w), 818 (s), 754 (s), 743 (w), 721 (w), 702 (m).

**Anal** calcd for $C_{58}H_{74}N_2O_2S$ (661.0): C 81.76, H 9.15, N 4.24, S 4.85; Found: C 81.84, H 9.24, N 4.17, S 5.03.

**10-(2-Decyltetradecyl)-3-ethynyl-7-(4-nitrophenyl)-10*H*-phenothiazine (3d)**

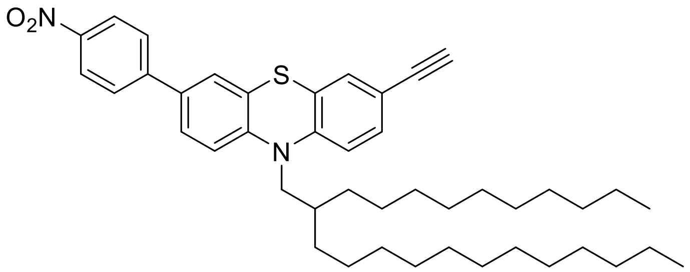


$C_{45}H_{60}N_2S$
[681.04]

According to **GP6** and after flash chromatography on silica gel (*n*-hexane/diethyl ether 25:1) compound **3d** (180 mg, 60%) was obtained as a red oil.

**R*f*** (*n*-hexane/diethyl ether 25:1): 0.48.

**$^1$H NMR (600 MHz, acetone-d$_6$):** $\delta$ 0.83 – 0.90 (m, 6H), 1.16 – 1.35 (m, 40H), 2.01 – 2.03 (m, 1H), 3.62 (s, 1H), 3.94 (d, $^3J$ = 7.2 Hz, 2H), 7.08 (d, $^3J$ = 8.5 Hz, 1H), 7.21 (d, $^3J$ = 8.5 Hz, 1H), 7.27 (d, $^4J$ = 1.9 Hz, 1H), 7.34 (dd, $^3J$ = 8.4, $^4J$ = 2.0 Hz, 1H), 7.60 (d, $^4J$ = 2.2 Hz, 1H), 7.67 (dd, $^3J$ = 8.5, $^4J$ = 2.2 Hz, 1H), 7.94 (d, $^3J$ = 8.9 Hz, 2H), 8.29 (d, $^3J$ = 8.9 Hz, 2H).

**$^{13}$C NMR (75 MHz, acetone-d$_6$):** $\delta$ 14.38 ($CH_3$), 14.39 ($CH_3$), 23.4 (2 $CH_2$), 26.91 ($CH_2$), 26.93 ($CH_2$), 30.45 ($CH_2$), 30.46 ($CH_2$), 30.48 ($CH_2$), 30.49 ($CH_2$), 30.66 ($CH_2$), 30.68 ($CH_2$), 30.71 (CH2), 30.74 ($CH_2$), 30.75 ($CH_2$), 30.76 ($CH_2$), 30.78 ($CH_2$), 30.97 ($CH_2$), 30.98 ($CH_2$), 30.99 ($CH_2$), 31.5 ($CH_3$), 32.68 ($CH_2$), 32.69 ($CH_2$), 33.1 (CH), 35.3 ($CH_2$), 52.2 ($CH_2$), 79.1 (CH), 83.4 ($C_{quat}$), 117.3 (CH), 117.5 ($C_{quat}$), 117.9 (CH), 124.9 (2 CH), 126.0 ($C_{quat}$), 126.6 ($C_{quat}$), 126.8 (CH), 127.6 (CH), 128.0 (2 CH), 131.2 (CH), 132.4 (CH), 133.9 ($C_{quat}$), 146.7 ($C_{quat}$), 146.8 ($C_{quat}$), 146.9 ($C_{quat}$), 147.7 ($C_{quat}$).

**MS (EI) m/z (%):** 681 ([M], 100), 357 ($[C_{21}H_{13}N_2O_2S]^+$,85), 344 ($[C_{20}H_{11}N_2O_2S]^+$, 30), 325 (35), 297 (15), 57 ($[C_4H_9]^+$, 20).

**IR $\tilde{\nu}$ [cm$^{-1}$]**: 3304 (w), 3046 (w), 3028 (w), 2922 (s), 2851 (s), 2727 (w), 2108 (w), 1603 (w), 1582 (w), 1499 (w), 1460 (s), 1443 (w), 1412 (w), 1396 (w), 1377 (w), 1358 (m), 1335 (w), 1292 (w), 1248 (m), 1221 (w), 1198 (w), 1157 (w), 1148 (w), 1140 (w), 1103 (m), 955 (w), 930 (w), 883 (s), 843 (m), 816 (s), 791 (m), 735 (s), 721 (w), 700 (w), 648 (m), 629 (w).

**Anal** calcd for $C_{45}H_{60}N_2S$ (681.0): C 77.60, H 8.88, N 4.11, S 4.71; Found: C 77.42, H 8.89, N 4.01, S 4.73.

**10-(2-Decyltetradecyl)-3-ethynyl-7-(4-(trifluoromethyl)phenyl)-10*H*-phenothiazine (3e)**

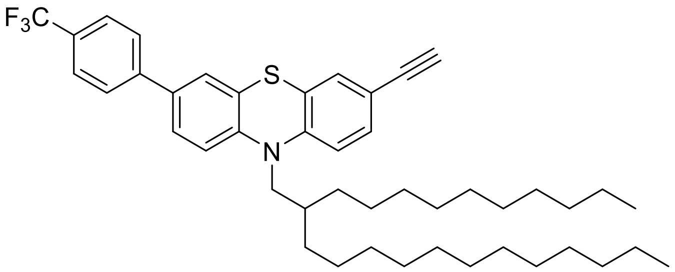

$C_{45}H_{60}F_3NS$
[704.04]

According to **GP6** and after flash chromatography on silica gel (*n*-hexane/diethyl ether 25:1) compound **3e** (240 mg, 61%) was obtained as a yellow oil.

**R*f*** (*n*-hexane/diethyl ether 25:1): 0.33.

**$^1$H NMR (600 MHz, acetone-d$_6$):** $\delta$ 0.83 - 0.89 (m, 6H), 1.19 – 1.43 (m, 40H), 2.01 – 2.03 (m, 1H), 3.61 (s, 1H), 3.93 (d, $^3J$ = 7.2 Hz, 2H), 7.07 (d, $^3J$ = 8.5 Hz, 1H), 7.19 (d, $^3J$ = 8.5 Hz, 1H), 7.27 (d, $^4J$ = 1.9 Hz, 1H), 7.33 (dd, $^3J$ = 8.4, $^3J$ = 1.9, 1H), 7.53 (d, $^4J$ = 2.2 Hz, 1H), 7.60 (dd, $^3J$ = 8.5, $^4J$ = 2.2 Hz, 1H), 7.76 (d, $^3J$ = 8.2 Hz, 2H), 7.87 (d, $^3J$ = 8.1 Hz, 2H).

**$^{13}$C NMR (75 MHz, acetone-d$_6$):** $\delta$ 14.38 ($CH_3$), 14.39 ($CH_3$), 23.4 (2 $CH_2$), 26.91 ($CH_2$), 26.93 ($CH_2$), 30.45 ($CH_2$), 30.46 ($CH_2$), 30.48 ($CH_2$), 30.49 ($CH_2$), 30.66 ($CH_2$), 30.68 ($CH_2$), 30.71 (CH2), 30.74 ($CH_2$), 30.75 ($CH_2$), 30.76 ($CH_2$), 30.78 ($CH_2$), 30.97 ($CH_2$), 30.98 ($CH_2$), 30.99 ($CH_2$), 31.5 ($CH_3$), 32.68 ($CH_2$), 32.69 ($CH_2$), 33.1 (CH), 35.3 ($CH_2$), 52.2 ($CH_2$), 79.0 (CH), 83.5 ($C_{quat}$), 117.2 (CH), 117.4 ($C_{quat}$), 117.9 (CH), 126.1 ($C_{quat}$), 126.5 ($C_{quat}$), 126.5 ($CF_3$), 127.3 (CH), 127.7 (4 CH), 129.2 ($C_{quat}$), 129.4 ($C_{quat}$), 131.2 (CH), 132.3 (CH), 134.8 ($C_{quat}$), 144.3 ($C_{quat}$), 146.3 ($C_{quat}$), 146.9 ($C_{quat}$).

**MS (EI) m/z (%):** 704 ([M], 100), 380 ($[C_{22}H_{13}F_3NS]^+$, 90), 366 ($[C_{21}H_{11}F_3NS]^+$, 45), 348 (40).

**IR $\tilde{\nu}$ [cm$^{-1}$]**: 3006 (w), 3034 (w), 2953 (w), 2922 (s), 2853 (m), 2108 (w), 1616 (m), 1603 (w), 1582 (w), 1522 (w), 1495 (w), 1460 (s), 1420 (w), 1396 (w), 1377 (w), 1323 (s), 1302 (w), 1279 (w), 1263 (w), 1250 (w), 1219 (w), 1192 (w), 1163 (s), 1125 (s), 1111 (w), 1072 (s), 1047 (w), 1036 (w), 1015 (m), 966 (w), 953 (w), 937 (w), 885 (m), 843 (m), 814 (s), 764 (w), 719 (w), 692 (w), 648 (m), 608 (m).

**Anal** calcd for $C_{45}H_{60}F_3NS$ (704.0): C 76.77, H 8.59, N 1.99, S 4.55; Found: C 76.85, H 8.69, N 2.02, S 4.65.

**4-(10-(2-Decyltetradecyl)-7-ethynyl-10*H*-phenothiazin-3-yl)-*N*,*N*-diphenylaniline (3f)**

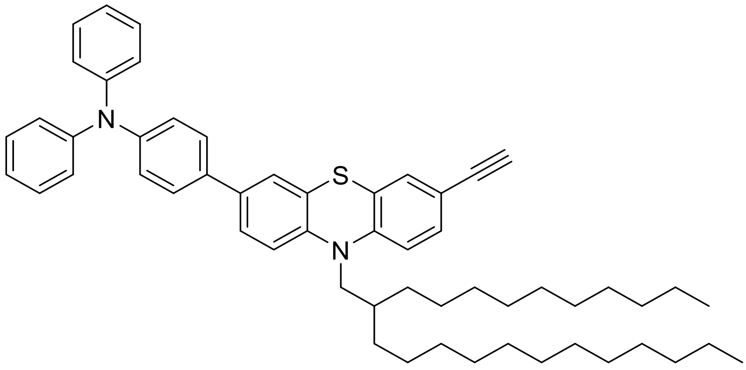

$C_{56}H_{70}N_2S$
[803.25]

According to **GP6** and after flash chromatography on silica gel (*n*-hexane/diethyl ether 50:1) compound **3f** (120 mg, 38%) was obtained as a yellow oil.

**R**$_f$ (*n*-hexane/diethyl ether 50:1): 0.75.

**$^1$H NMR (600 MHz, acetone-d$_6$):** $\delta$ 0.84 – 0.89 (m, 6H), 1.16 – 1.44 (m, 40H), 2.01 – 2.04 (m, 1H), 3.59 (s, 1H), 3.90 (d, $^3J$ = 7.2 Hz, 2H), 7.04 – 7.10 (m, 9H), 7.12 (d,$^3J$ = 8.5 Hz, 1H), 7.26 (d, $^4J$ = 1.9 Hz, 1H), 7.28 – 7.35 (m, 5H), 7.43 (d, $^4J$ = 2.2 Hz, 1H), 7.49 (dd, $^3J$ = 8.5, $^4J$ = 2.2 Hz, 1H), 7.56 (d, $^3J$ = 8.7 Hz, 2H).

**$^{13}$C NMR (75 MHz, acetone-d$_6$):** $\delta$ 14.38 ($CH_3$), 14.39 ($CH_3$), 23.4 (2 $CH_2$), 26.91 ($CH_2$), 26.93 ($CH_2$), 30.45 ($CH_2$), 30.46 ($CH_2$), 30.48 ($CH_2$), 30.49 ($CH_2$), 30.66 ($CH_2$), 30.68 ($CH_2$), 30.71 (CH2), 30.74 ($CH_2$), 30.75 ($CH_2$), 30.76 ($CH_2$), 30.78 ($CH_2$), 30.97 ($CH_2$), 30.98 ($CH_2$), 30.99 ($CH_2$), 31.5 ($CH_3$), 32.68 ($CH_2$), 32.69 ($CH_2$), 33.1 (CH), 35.3 ($CH_2$), 52.2 ($CH_2$), 78.9 (CH), 83.6 ($C_{quat}$), 117.0 (CH), 117.1 ($C_{quat}$), 117.8 (CH), 124.0 (2 CH), 124.6 (2 CH), 125.2 (4 CH), 125.8 (CH), 126.17 ($C_{quat}$), 126.22 ($C_{quat}$), 126.4 (CH), 128.0 (2 CH), 130.3 (4 CH), 131.2 (CH), 132.2 (CH), 134.5 ($C_{quat}$), 136.1 ($C_{quat}$), 144.9 ($C_{quat}$), 147.2 ($C_{quat}$), 148.0 ($C_{quat}$), 148.6 (2 $C_{quat}$).

**MS (EI) m/z (%):** 803 ([M], 100), 479 ($[C_{33}H_{23}N_2S]^+$, 40), 465 ($[C_{32}H_{21}N_2S]^+$, 65), 240 (35), 233 (25).

**IR $\tilde{\nu}$ [cm$^{-1}$]**: 3310 (w), 3300 (w), 3061 (w), 3034 (w), 2920 (s), 2851 (s), 2108 (w), 1585 (s), 1541 (w), 1516 (w), 1493 (s), 1456 (s), 1393 (w), 1377 (w), 1327 (w), 1314 (w), 1273 (s), 1250 (m), 1196 (w), 1179 (w), 1146 (w), 1105 (w), 1074 (w), 1049 (w), 1028 (w), 1013 (w), 999 (w), 976 (w), 961 (w), 937 (w), 920 (w), 883 (m), 837 (w), 814 (s), 752 (s), 723 (w), 694 (s), 644 (m).

**Anal** calcd for $C_{56}H_{70}N_2S$ (803.3): C 83.74, H 8.78, N 3.49, S 3.99; Found: C 83.73, H 8.54, N 3.52, S 4.01.

**4-(10-(2-Decyltetradecyl)-7-ethynyl-10H-phenothiazin-3-yl)-*N*,*N*-bis(4 methoxyphenyl)aniline (3g)**

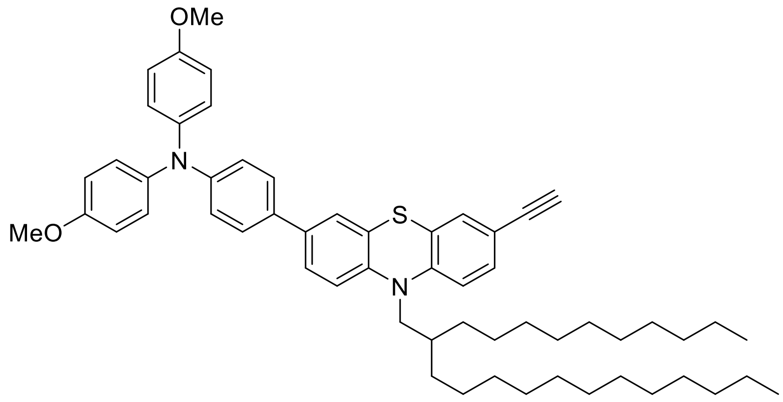

$C_{58}H_{74}N_2O_2S$
[863.30]

According to **GP6** and after flash chromatography on silica gel (*n*-hexane/diethyl ether 10:1) compound **3g** (300 mg, 50%) was obtained as a yellow oil.

**R**$_f$ (*n*-hexane/diethyl ether 10:1): 0.58.

**$^1$H NMR (600 MHz, acetone-d$_6$):** $\delta$ 0.84 – 0.90 (m, 6H), 1.18 – 1.45 (m, 40H), 1.97 – 2.03 (m, 1 H), 3.58 (s, 1H), 3.79 (s, 6 H), 3.89 (d, $^3J$ = 7.2 Hz, 3H), 6.87 – 6.94 (m, 6H), 7.01 – 7.12 (m, 6H), 7.25 (d, $^4J$ = 1.9 Hz, 1H), 7.31 (dd, $^3J$ = 8.4, $^4J$ = 1.9 Hz, 1H), 7.39 (d, $^4J$ = 2.2 Hz, 1H), 7.42 – 7.49 (m, 3H).

**$^{13}$C NMR (75 MHz, acetone-d$_6$):** $\delta$ 14.38 ($CH_3$), 14.39 ($CH_3$), 23.4 (2 $CH_2$), 26.91 ($CH_2$), 26.93 ($CH_2$), 30.45 ($CH_2$), 30.46 ($CH_2$), 30.48 ($CH_2$), 30.49 ($CH_2$), 30.66 ($CH_2$), 30.68 ($CH_2$), 30.71 (CH2), 30.74 ($CH_2$), 30.75 ($CH_2$), 30.76 ($CH_2$), 30.78 ($CH_2$), 30.97 ($CH_2$), 30.98 ($CH_2$), 30.99 ($CH_2$), 31.5 ($CH_3$), 32.68 ($CH_2$), 32.69 ($CH_2$), 33.1 (CH), 35.3 ($CH_2$), 52.2 ($CH_2$), 55.7 (2 $CH_3$), 78.8 (CH), 83.6 ($C_{quat}$), 115.7 (4 CH), 117.0 (2 CH), 117.7 (CH), 121.0 (4 CH), 125.6 (CH), 126.09 ($C_{quat}$), 126.11 ($C_{quat}$), 126.2 ($C_{quat}$), 127.6 (CH), 127.7 (4 CH), 131.15 (CH), 131.19 ($C_{quat}$), 132.2 (CH), 136.5 ($C_{quat}$), 141.6 (2 $C_{quat}$), 144.5 ($C_{quat}$), 147.3 ($C_{quat}$), 149.1 ($C_{quat}$), 157.3 (2 $C_{quat}$).

**MS (EI) m/z (%):** 863 ([M], 100), 539 ($[C_{35}H_{27}N_2O_2S]^+$, 15), 525 ($[C_{34}H_{25}N_2O_2S]^+$, 40), 270 (20), 246 (15), 57 ($[C_4H_9]^+$, 15).

**IR $\tilde{\nu}$ [cm$^{-1}$]**: 3302 (w), 3287 (w), 3034 (w), 2995 (w), 2922 (s), 2851 (s), 2108 (w), 1603 (m), 1582 (w), 1504 (s), 1495 (w), 1460 (s), 1441 (w), 1393 (w), 1377 (w), 1317 (m), 1273 (w), 1238 (s), 1194 (w), 1179 (w) 1165 (w), 1148 (w), 1105 (m), 1036 (s), 1011 (w), 939 (w), 912 (w), 883 (w), 816 (s), 781 (w), 752 (w), 718 (w), 696 (w), 648 (w).

**Anal** calcd for $C_{58}H_{74}N_2O_2S$ (863.3): C 80.69, H 8.64, N 3.24, S 3.71; Found: C 80.96, H 8.92, N 3.16, S 3.62.

**10-(2-Decyltetradecyl)-3-ethynyl-7-(quinolin-3-yl)-10*H*-phenothiazine (3h)**

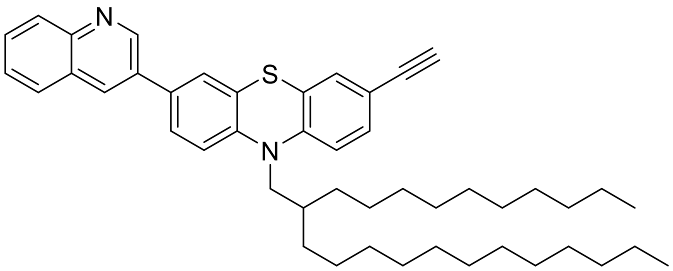

$C_{52}H_{65}NS$
[687.09]

According to **GP6** and after flash chromatography on silica gel (*n*-hexane/diethyl ether 5:1) compound **3h** (130 mg, 33%) was obtained as a yellow oil.

**R$_f$** (*n*-hexane/diethyl ether 5:1): 0.56.

**$^1$H NMR (600 MHz, acetone-d$_6$):** $\delta$ 1.19 – 1.42 (m, 40H), 2.06 – 2.07 (m, 1H), 3.61 (s, 1H), 3.96 (d, $^3J$ = 7.2 Hz, 2H), 7.09 (d, $^3J$ = 8.5 Hz, 1H), 7.25 (d, $^3J$ = 8.5 Hz, 1H), 7.29 (d, $^4J$ = 1.9 Hz, 1H), 7.35 (dd, $^3J$ = 8.4, $^4J$ = 1.9 Hz, 1H), 7.60 – 7.64 (m, 1H), 7.68 (d, $^3J$ = 2.2 Hz, 1 H), 7.71 – 7.77 (m, 2H), 7.99 – 8.03 (m, 1H), 8.05 – 8.07 (m, 1H), 8.52 – 8.53 (m, 1H), 9.22 (d, $^3J$ = 2.4 Hz, 1H).

**$^{13}$C NMR (75 MHz, acetone-d$_6$):** $\delta$ 14.38 ($CH_3$), 14.39 ($CH_3$), 23.4 (2 $CH_2$), 26.91 ($CH_2$), 26.93 ($CH_2$), 30.45 ($CH_2$), 30.46 ($CH_2$), 30.48 ($CH_2$), 30.49 ($CH_2$), 30.66 ($CH_2$), 30.68 ($CH_2$), 30.71 (CH2), 30.74 ($CH_2$), 30.75 ($CH_2$), 30.76 ($CH_2$), 30.78 ($CH_2$), 30.97 ($CH_2$), 30.98 ($CH_2$), 30.99 ($CH_2$), 31.5 ($CH_3$), 32.68 ($CH_2$), 32.69 ($CH_2$), 33.1 (CH), 35.3 ($CH_2$), 52.2 ($CH_2$), 79.0 (CH), 83.5 ($C_{quat}$), 117.2 (CH), 117.3 ($C_{quat}$), 118.1 (CH), 126.1 ($C_{quat}$), 126.67 ($C_{quat}$), 126.72 (CH), 127.4 (CH), 127.8 (CH), 129.0 ($C_{quat}$), 129.1 (CH), 129.98 (CH), 130.02 (CH), 131.2 (CH), 132.3 (CH), 132.8 (CH), 133.1 ($C_{quat}$), 133.4 ($C_{quat}$), 146.0 ($C_{quat}$), 147.0 ($C_{quat}$), 148.3 ($C_{quat}$), 150.1 (CH).

**MS (EI) m/z (%):** 687 (100), 735 ([M], 100), 363 ([$C_{24}H_{15}N_2S$]$^+$, 75), 349 ([$C_{23}H_{13}N_2S$]$^+$, 40), 331 (25), 182 (15), 57 ([$C_4H_9$]$^+$, 100).

**IR $\tilde{\nu}$ [cm$^{-1}$]**: 3304 (w), 2922 (s), 2851 (s), 2621 (w), 2158 (w), 2108 (w), 1605 (w), 1585 (w), 1503 (w), 1456 (s), 1427 (w), 1396 (m), 1377 (w), 1341 (s), 1296 (w), 1250 (s), 1229 (w), 1200 (w), 1163 (w), 1142 (w), 1125 (w), 1103 (m), 1042 (w), 1018 (w), 962 (w), 939 (w), 910 (w), 883 (s), 858 (w), 816 (s), 785 (m), 750 (s), 719 (w), 698 (w), 644 (w), 617 (w).

**Anal** calcd for $C_{52}H_{65}NS$ (687.1): C 82.16, H 9.10, N 4.08, S 4.67; Found: C 82.15, H 9.24, N 3.99, S 4.68.

**3-(Anthracen-9-yl)-10-(2-decyltetradecyl)-7-ethynyl-10*H*-phenothiazine (3i)**

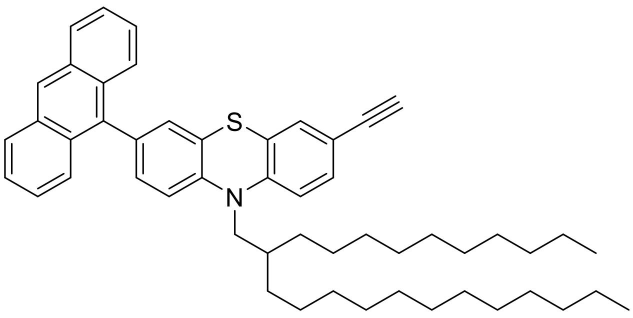

$C_{52}H_{65}NS$
[736.16]

According to **GP6** and after flash chromatography on silica gel (*n*-hexane/diethyl ether 50:1) compound **3i** (50 mg, 29%) was obtained as a yellow oil.

**R**$_f$ (*n*-hexane/diethyl ether 50:1): 0.70.

**$^1$H NMR (600 MHz, acetone-d$_6$):** $\delta$ 0.81 – 0.88 (m, 6H), 1.18 – 1.50 (m, 40H), 2.13 – 2.20 (m, 1H), 3.61 (s, 1 H), 4.03 (d, $^3J$ = 7.2 Hz, 2H), 7.15 (d, $^3J$ = 8.5 Hz, 1H), 7.22 (d, $^4J$ = 2.0 Hz, 1H), 7.26 (dd, $^3J$ = 8.1, $^4J$ = 2.0 Hz, 1H), 7.30 (d, $^4J$ = 2.0 Hz, 1H), 7.33 – 7.35 (m, 1H), 7.37 – 7.42 (m, 3H), 7.48 – 7.52 (m, 2H), 7.67 (dd, $^3J$ = 8.8, $^4J$ =1.0 Hz, 2H), 8.12 (d, $^3J$ = 8.4 Hz, 2H), 8.62 (s, 1H).

**$^{13}$C NMR (75 MHz, acetone-d$_6$):** $\delta$ 14.38 ($CH_3$), 14.39 ($CH_3$), 23.4 (2 $CH_2$), 26.91 ($CH_2$), 26.93 ($CH_2$), 30.45 ($CH_2$), 30.46 ($CH_2$), 30.48 ($CH_2$), 30.49 ($CH_2$), 30.66 ($CH_2$), 30.68 ($CH_2$), 30.71 (CH2), 30.74 ($CH_2$), 30.75 ($CH_2$), 30.76 ($CH_2$), 30.78 ($CH_2$), 30.97 ($CH_2$), 30.98 ($CH_2$), 30.99 ($CH_2$), 31.5 ($CH_3$), 32.68 ($CH_2$), 32.69 ($CH_2$), 33.1 (CH), 35.3 ($CH_2$), 52.2 ($CH_2$), 79.0 (CH), 83.6 ($C_{quat}$), 117.26 (CH), 117.29 ($C_{quat}$), 117.5 (CH), 126.0 ($C_{quat}$), 126.1 (CH), 126.4 ($C_{quat}$), 126.5 (2 CH), 127.2 (2 CH), 127.6 (CH), 129.3 (2 CH), 130.51 (CH) 130.52 (CH), 131.2 (2 $C_{quat}$), 131.3 (2 CH), 132.3 (2 $C_{quat}$), 132.4 (CH), 134.1 ($C_{quat}$), 136.5 ($C_{quat}$), 145.6 ($C_{quat}$), 147.3 ($C_{quat}$).

**MS (EI) m/z (%):** 784 (45), 735 ([M], 100), 460 (35), 444 (25), 412 ($[C_{29}H_{18}NS]^+$, 70), 398 ($[C_{28}H_{16}NS]^+$, 40), 368 (25), 228 (35), 200 (55), 129 (30), 69 (45), 57 ($[C_4H_9]^+$, 100).

**IR $\tilde{\nu}$ [cm$^{-1}$]**: 3304 (w), 3050 (w), 2922 (s), 2851 (s), 2727 (w), 2108 (w), 1603 (w), 1582 (w), 1499 (w), 1460 (s), 1443 (w), 1411 (m), 1396 (w), 1377 (w), 1358 (m), 1335 (w), 1292 (w), 1248 (s), 1221 (w), 1198 (w), 1157 (w), 1148 (w), 1140 (w), 1103 (w), 1045 (w), 1030 (w), 1015

(m), 955 (w), 930 (w), 883 (s), 843 (s), 816 (s), 791 (m), 735 (s), 721 (w), 700 (w), 648 (m), 629 (w).

**Anal** calcd. for $C_{52}H_{65}NS$ (736.2): C 84.84, H 8.90, N 1.90, S 4.36; Found: C 84.61, H 9.17, N 1.82, S 4.20

**10-(2-Decyltetradecyl)-7-ethynyl-10*H*-3,10'-biphenothiazine (3j)**

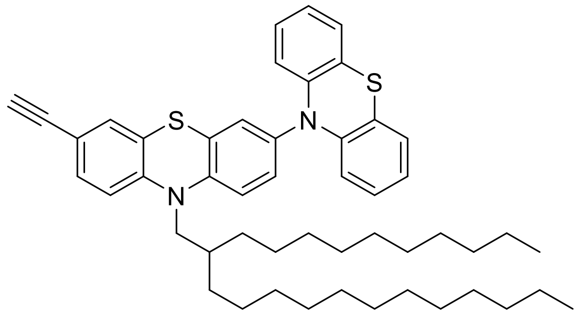

$C_{50}H_{64}N_2S_2$
757.20

According to **GP6** and after flash chromatography on silica gel (*n*-hexane/diethyl ether 50:1) compound **3j** (170 mg, 50%) was obtained as a yellow oil.

**R*f*** (*n*-hexane/diethyl ether 50:1): 0.80

**$^1$H NMR (300 MHz, acetone-d$_6$):** $\delta$ 0.80 – 1.00 (m, 6H), 1.19 – 1.52 (m, 40H), 2.09 – 2.17 (m, 1H), 3.62 (s, 1H), 3.97 (d, $^3J$ = 7.2 Hz, 2H), 6.28 (dd, $^3J$ = 8.0, $^3J$ = 1.5 Hz, 2H), 6.78 – 6.98 (m, 4H), 7.02 (dd, $^3J$ = 7.4, $^4J$ = 1.8 Hz, 2 H), 7.12 (d, $^3J$ = 8.5 Hz, 1H), 7.20 – 7.31 (m, 3H), 7.31 – 7.42 (m, 2H).

**$^{13}$C NMR (150 MHz, acetone-d$_6$):** $\delta$ 14.35 ($CH_3$), 14.36 ($CH_3$), 23.3 (CH), 26.78 ($CH_2$), 26.83 ($CH_2$), 30.45 ($CH_2$), 30.46 ($CH_2$), 30.48 ($CH_2$), 30.49 ($CH_2$), 30.66 ($CH_2$), 30.68 ($CH_2$), 30.71 ($CH_2$), 30.75 ($CH_2$), 30.76 ($CH_2$), 30.78 ($CH_2$), 30.97 ($CH_2$), 30.98 ($CH_2$), 30.99 ($CH_2$), 31.99 ($CH_2$), 32.11 ($CH_3$), 32.66 ($CH_2$), 32.67 ($CH_2$), 35.3 (CH), 52.3 ($CH_2$), 79.1 (CH), 83.4 ($C_{quat}$), 116.9 (2 CH), 117.4 (CH), 117.6 ($C_{quat}$), 119.2 (CH), 120.8 (2 $C_{quat}$), 123.5 (2 CH), 126.1 ($C_{quat}$), 127.4 (2 CH), 128.0 (2 CH), 128.1 ($C_{quat}$), 130.3 (CH), 131.0 (CH), 131.3 (CH), 132.4 (CH), 136.5 ($C_{quat}$), 145.1 (2 $C_{quat}$), 146.2 ($C_{quat}$), 146.9 ($C_{quat}$).

**MS (EI) m/z (%):** 757 ([M], 60), 433 ($[C_{27}H_{17}N_2S_2]^+$, 35), 419 ($[C_{26}H_{15}N_2S_2]^+$, 100), 401 (20), 198 (25), 57 ($[C_4H_9]^+$, 25).

**IR $\tilde{\nu}$ [cm$^{-1}$]**: 2920 (m), 2851 (m), 2345 (w), 1593 (w), 1572 (w), 1497 (w), 1456 (s), 1441 (m), 1396 (w), 1377 (w), 1333 (w), 1304 (m), 1236 (m), 1152 (w), 1128 (w), 1101 (w), 1082 (w),

1043 (w), 922 (w), 883 (w), 845 (w), 818 (m), 777 (w), 741 (s), 716 (w), 710 (w), 698 (w), 611 (w).

**Anal** calcd. for $C_{50}H_{64}N_2S_2$ (725.1): C 79.31, H 8.52, N 3.70, S 8.47; Found: C 79.55, H 8.72, N 3.64, S 8.57.

**10-(2-Decyltetradecyl)-7-ethynyl-*N*,*N*-diphenyl-10*H*-phenothiazine-3-amine (3k)**

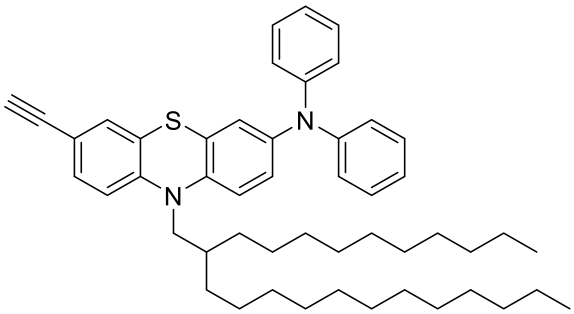

$C_{50}H_{66}N_2S$
[727.15]

According to **GP6** and after flash chromatography on silica gel (*n*-hexane/diethyl ether 70:1) compound **3k** (52 mg, 28%) was obtained as a yellow oil.

**R***f* (*n*-hexane/diethyl ether 70:1): 0.8.

**$^1$H NMR (600 MHz, acetone-d$_6$):** $\delta$ 0.84 – 0.90 (m, 6H), 1.19 – 1.47 (m, 40H), 1.99 – 2.04 (m, 1H), 3.58 (s, 1H), 3.85 (d, $^3J$ = 7.2 Hz, H), 6.86 (d, $^4J$ = 2.5 Hz, 1 H), 6.92 (dd, $^3J$ = 8.7, $^4J$ = 2.5 Hz, 1H), 6.99 – 7.04 (m, 8H), 7.21 (d, $^4J$ = 1.9 Hz, 1H), 7.24 – 7.29 (m, 4H), 7.32 (dd, $^3J$ = 8.4, $^4J$ = 1.9 Hz, 1H).

**$^{13}$C NMR (75 MHz, acetone-d$_6$):** $\delta$ 14.38 ($CH_3$), 14.39 ($CH_3$), 23.4 (CH), 26.74 ($CH_2$), 26.77 ($CH_2$), 30.45 ($CH_2$), 30.46 ($CH_2$), 30.48 ($CH_2$), 30.49 ($CH_2$), 30.66 ($CH_2$), 30.68 ($CH_2$), 30.71 ($CH_2$), 30.75 ($CH_2$), 30.76 ($CH_2$), 30.78 ($CH_2$), 30.97 ($CH_2$), 30.98 ($CH_2$), 30.99 ($CH_2$), 31.99 ($CH_2$), 32.11 ($CH_3$), 32.63 ($CH_2$), 32.64 ($CH_2$), 35.2 (CH), 52.1 ($CH_2$), 76.9 (CH), 81.8 ($C_{quat}$), 116.91 ($C_{quat}$) , 116.96 (CH), 118.1 (CH), 123.5 (2 CH), 124.3 (4 CH), 124.4 (CH), 125.0 (CH), 126.0 ($C_{quat}$), 126.7 ($C_{quat}$), 130.2 (4 CH), 131.2 (CH), 132.2 (CH), 141.7 ($C_{quat}$), 144.1 ($C_{quat}$), 147.4 ($C_{quat}$), 148.7 (2 $C_{quat}$).

**MS (EI) m/z (%):** 727 ([M], 45), 403 ([$C_{27}H_{19}N_2S$]$^+$, 20), 389 ([$C_{26}H_{17}N_2S$]$^+$, 100), 121 (20), 57 ([$C_4H_9$]$^+$, 15).

**IR $\tilde{\nu}$ [cm$^{-1}$]**: 3292 (w), 2922 (s), 2851 (s), 2110 (w), 1587 (m), 1493 (s), 1456 (s), 1398 (w), 1377 (w), 1329 (w), 1310 (w), 1296 (w), 1273 (m), 1260 (m), 1250 (w), 1229 (w), 1175 (w),

1153 (w), 1144 (w), 1103 (w), 1076 (w), 1043 (w), 1028 (w), 957 (w), 883 (w), 868 (w), 816 (m), 750 (s), 719 (w), 694 (s), 640 (w).

**Anal** calcd. for $C_{50}H_{66}N_2S$ (727.2): C 82.59, H 9.15, N 3.85, S 4.41; Found: C 82.57, H 9.06, N 3.83, S 4.40.

**10-(2-Decyltetradecyl)-7-ethynyl-*N*,*N*-bis(4-methoxyphenyl)-10*H*-phenothiazine-3-amine (3l)**

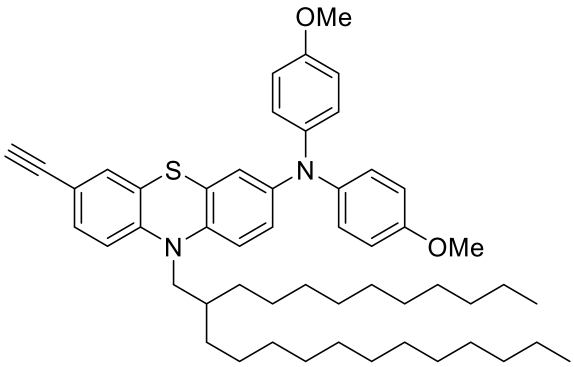

$C_{52}H_{70}N_2O_2S$
[786.52]

According to **GP6** and after flash chromatography on silica gel (*n*-hexane/diethyl ether 70:1) compound **3l** (183 mg, 43%) was obtained as a yellow oil.

**R*f*** (*n*-hexane/diethyl ether 30:1): 0.28.

**$^1$H NMR (600 MHz, acetone-d$_6$):** $\delta$ 0.83 – 0.90 (m, 6H), 1.19 – 1.47 (m, 40H), 1.96 – 2.03 (m, 1H), 3.56 (s, 1H), 3.78 (s, 6H), 3.81 (d, $^3J$ = 7.2 Hz, 2H), 6.69 (d, $^4J$ = 2.6 Hz, 1H), 6.76 (dd, $^3J$ = 8.8, $^4J$ = 2.6 Hz, 1H), 6.85 – 6.89 (m, 4H), 6.92 (d, $^3J$ = 8.8 Hz, 1H), 6.96 – 7.01 (m, 5H), 7.19 (d, $^4J$ = 1.9 Hz, 1H), 7.30 (dd, $^3J$ = 8.4, $^4J$ = 2.0 Hz, 1H).

**$^{13}$C NMR (75 MHz, acetone-d$_6$):** $\delta$ 14.38 ($CH_3$), 14.39 ($CH_3$), 23.4 (CH), 26.74 ($CH_2$), 26.77 ($CH_2$), 30.45 ($CH_2$), 30.46 ($CH_2$), 30.48 ($CH_2$), 30.49 ($CH_2$), 30.66 ($CH_2$), 30.68 ($CH_2$), 30.71 ($CH_2$), 30.75 ($CH_2$), 30.76 ($CH_2$), 30.78 ($CH_2$), 30.97 ($CH_2$), 30.98 ($CH_2$), 30.99 ($CH_2$), 31.99 ($CH_2$), 32.01 ($CH_3$), 32.66 ($CH_2$), 32.67 ($CH_2$), 35.3 (CH), 52.0 ($CH_2$), 55.7 (2 $CH_3$), 78.7 (CH), 83.7 ($C_{quat}$), 115.6 (4 CH), 116.6 ($C_{quat}$), 116.7 (CH), 117.9 (CH), 121.0 (CH), 121.5 (CH), 126.0 ($C_{quat}$), 126.3 ($C_{quat}$), 126.8 (4 CH), 131.1 (CH), 132.2 (CH), 139.6 ($C_{quat}$), 141.9 (2 $C_{quat}$), 145.6 ($C_{quat}$), 147.7 ($C_{quat}$), 156.8 (2 $C_{quat}$).

**MS (EI) m/z (%):** 787 ([M], 55), 463 ($[C_{29}H_{23}N_2O_2S]^+$, 15), 449 ($[C_{28}H_{21}N_2O_2S]^+$, 100).

**IR $\tilde{\nu}$ [cm$^{-1}$]**: 3306 (w), 3038 (w), 2994 (w), 2922 (m), 2851 (m), 2108 (w), 1601 (w), 1582 (w), 1503 (s), 1460 (s), 1441 (w), 1400 (w), 1377 (w), 1321 (w), 1296 (w), 1238 (s), 1179 (w), 1169

(w), 1105 (w), 1038 (m), 1009 (w), 959 (w), 916 (w), 883 (w), 868 (w), 826 (m), 818 (w), 806 (w), 781 (w), 750 (w), 719 (w), 698 (w), 648 (w).

**Anal** calcd. for $C_{52}H_{70}N_2O_2S$ (786.5): C 79.34, H 8.96, N 3.56, S 4.07; Found: C 79.55, H 9.03, N 3.55, S 4.00.

**3-(9*H*-Carbazol-9-yl)-10-(2-decyltetradecyl)-7-ethynyl-10*H*-phenothiazine (3m)**

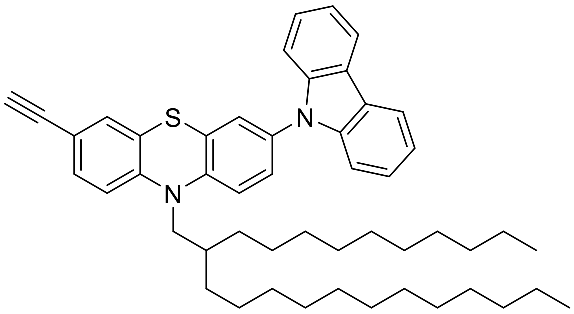

$C_{50}H_{64}N_2S$
[725.14]

According to **GP6** and after flash chromatography on silica gel (*n*-hexane/diethyl ether 50:1) compound **3m** (205 mg, 56%) was obtained as a yellow oil.

**R*f*** (*n*-hexane/diethyl ether 50:1): 0.63.

**$^1$H NMR (600 MHz, acetone-d$_6$):** $\delta$ 0.83 – 0.89 (m, 6H), 1.21 – 1.44 (m, 40H), 2.09 – 2.13 (m, 1H), 3.62 (s, 1H), 4.00 (d, $^3J$ = 7.2 Hz, 2H), 7.13 (d, $^3J$ = 8.5 Hz, 1H), 7.26 – 7.29 (m, 2H), 7.30 (d, $^4J$ = 1.9 Hz, 1H), 7.33 – 7.39 (m, 4H), 7.40 – 7.43 (m, 3H), 7.45 (dd, $^3J$ = 8.5, $^4J$ = 2.4 Hz, 1H), 8.19 – 8.21 (m, 2H).

**$^{13}$C NMR (75 MHz, aeceton-d$_6$):** $\delta$ 14.35 ($CH_3$), 14.36 ($CH_3$), 23.3 (CH), 26.78 ($CH_2$), 26.83 ($CH_2$), 30.45 ($CH_2$), 30.46 ($CH_2$), 30.48 ($CH_2$), 30.49 ($CH_2$), 30.66 ($CH_2$), 30.68 ($CH_2$), 30.71 ($CH_2$), 30.75 ($CH_2$), 30.76 ($CH_2$), 30.78 ($CH_2$), 30.97 ($CH_2$), 30.98 ($CH_2$), 30.99 ($CH_2$), 31.99 ($CH_2$), 32.11 ($CH_3$), 32.66 ($CH_2$), 32.67 ($CH_2$), 35.3 (CH), 52.3 ($CH_2$), 79.1 (CH), 83.5 ($C_{quat}$), 110.5 (2 CH), 117.4 (CH), 117.5 ($C_{quat}$), 118.5 (CH), 120.8 (2 CH), 121.1 (2 CH), 124.1 (2 $C_{quat}$), 126.0 ($C_{quat}$), 126.6 (CH), 126.9 (2 CH), 127.3 (CH), 127.4 (Cquart), 131.3 (CH), 132.5 (CH), 133.3 ($C_{quat}$), 141.9 (2 $C_{quat}$), 145.5 ($C_{quat}$), 147.0 ($C_{quat}$).

**MS (EI) m/z (%):** 725 ([M], 75), 401 ($[C_{27}H_{17}N_2S]^+$, 70), 387 ($[C_{26}H_{15}N_2S]^+$, 100), 369 (35), 57 ($[C_4H_9]^+$, 25).

**IR $\tilde{\nu}$ [cm$^{-1}$]**: 3304 (w), 3055 (w), 2922 (s), 2160 (w), 2108 (w), 1969 (w), 1597 (w), 1584 (w), 1503 (m), 1462 (s), 1452 (s), 1396 (w), 1333 (m), 1312 (m), 1394 €, 1231 (s), 1179 (w), 1148

(w), 1119 (w), 1099 (w), 1045 (w), 1026 (w), 1015 (w), 1003 (w), 970 (w), 918 (w), 883 (w), 843 (w), 818 (m), 783 (w), 746 (s), 723 (s), 648 (m).

**Anal** calcd. for $C_{50}H_{64}N_2S$ (725.1): C 82.82, H 8.90, N 3.86, S 4.42; Found: C 83.05, H 8.92, N 3.90, S 4.68.

**10-(2-Decyltetradecyl)-7-ethynyl-*N*,*N*-bis(4-nitrophenyl)-10*H*-phenothiazine-3-amine (3n)**

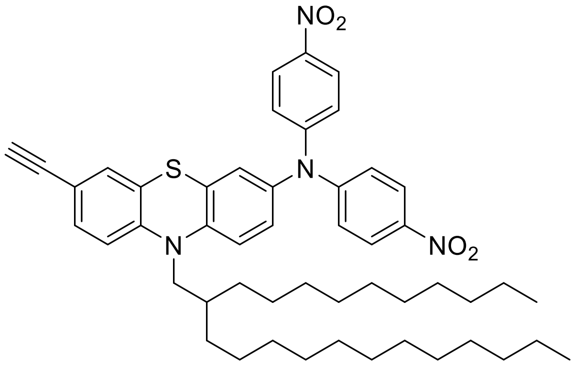


$C_{50}H_{64}N_4O_4S$
[817.15]

According to **GP6** and after flash chromatography on silica gel (*n*-hexane/diethyl ether 10:1) compound **3n** (90 mg, 20%) was obtained as a red oil.

**R*f*** (*n*-hexane/diethyl ether 10:1): 0.38.

**$^1$H NMR (300 MHz, acetone-d$_6$):** $\delta$ 0.81 – 0.92 (m, 6H), 1.15 – 1.54 (m, 40H), 1.98 – 2.04 (m, 1H), 3.61 (s, 1H), 3.91 (d, $^3J$ = 7.2 Hz, 2H), 7.09 (d, $^3J$ = 8.4 Hz, 1H), 7.12 – 7.19 (m, 3H), 7.23 (d, $^4J$ = 1.9 Hz, 1H), 7.28 (d, $^3J$ = 9.2 Hz, 4H), 7.35 (dd, $J$ = 8.4, 2.0 Hz, 1H), 8.18 (d, $^3J$ = 9.2 Hz, 4H).

**$^{13}$C NMR (75 MHz, acetone-d$_6$):** $\delta$ 14.38 ($CH_3$), 14.39 ($CH_3$), 23.4 (CH), 26.72 ($CH_2$), 26.76 ($CH_2$), 30.42 ($CH_2$), 30.45 ($CH_2$), 30.47 ($CH_2$), 30.49 ($CH_2$), 30.66 ($CH_2$), 30.68 ($CH_2$), 30.71 ($CH_2$), 30.75 ($CH_2$), 30.76 ($CH_2$), 30.78 ($CH_2$), 30.97 ($CH_2$), 30.98 ($CH_2$), 30.99 ($CH_2$), 31.9 ($CH_2$), 32.11 ($CH_3$), 32.67 ($CH_2$), 32.68 ($CH_2$), 52.2 ($CH_2$), 79.2 (CH), 83.4 ($C_{quat}$), 117.4 (CH), 117.6 ($C_{quat}$), 118.8 (CH), 123.1 (4 CH), 125.8 ($C_{quat}$), 126.2 (4 CH), 127.4 (CH), 127.8 ($C_{quat}$), 127.9 (CH), 131.3 (CH), 132.5 (CH), 140.7 ($C_{quat}$), 143.5 (2 $C_{quat}$), 145.2 ($C_{quat}$), 146.7 ($C_{quat}$), 152.8 (2 $C_{quat}$).

**MS (EI) m/z (%):** 817 ([M], 95), 493 ($[C_{27}H_{17}N_4O_4S]^+$, 70), 479 ($[C_{26}H_{15}N_4O_4S]^+$, 100), 461 (15), 433 (25), 57 ($[C_4H_9]^+$, 25).

**IR $\tilde{\nu}$ [cm$^{-1}$]**: 3296 (w), 3076 (w), 2922 (m), 2851 (m), 2687 (w), 2642 (w), 2594 (w), 2444 (w), 2108 (w), 1597 (m), 1580 (s), 1510 (w), 1493 (s), 1456 (s), 1420 (w), 1400 (w), 1377 (w), 1337 (s), 1302 (s), 1279 (s), 1250 (w), 1179 (w), 1144 (w), 1109 (s), 1043 (w), 1005 (w), 955 (w), 924 (w), 883 (w), 843 (m), 818 (m), 770 (w), 712 (w), 691 (w), 648 (w), 621 (w).

**Anal** calcd. for $C_{50}H_{64}N_4O_4S$ (817.2): C 73.49, H 7.89, N 6.86, S 3.92; Found: C 73.35, H 7.92, N 6.70, S 3.99.

**4-(10-(2-Decyltetradecyl)-7-ethynyl-10*H*-phenothiazine-3-yl)morpholine (3o)**

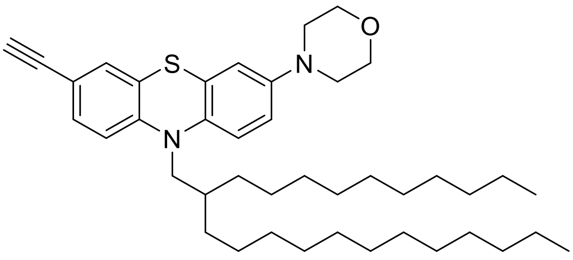

$C_{42}H_{64}N_2OS$
[645.06]

According to **GP6** and after flash chromatography on silica gel (*n*-hexane/diethyl ether 70:1) compound **3o** (101 mg, 55%) was obtained as a yellow oil.

**R*f*** (*n*-hexane/diethyl ether 70:1): 0.41.

**$^1$H NMR (600 MHz, acetone-d$_6$):** $\delta$ 0.85 – 0.91 (m, 6H), 1.18 – 1.43 (m, 40H), 1.94 – 2.00 (m, 1H), 3.04 – 3.08 (m, 4 H), 3.55 (s, 1H), 3.73 – 3.78 (m, 4H), 3.80 (d, $^3J$ = 7.2 Hz, 2H), 6.77 (d, $^4J$ = 2.8 Hz, 1H), 6.83 (dd, $^3J$ = 8.9, $^4J$ = 2.8 Hz, 1H), 6.93 – 6.99 (m, 2H), 7.21 (d, $^4J$ = 1.9 Hz, 1H), 7.28 (dd, $^3J$ = 8.4, $^4J$ = 1.9 Hz, 1H).

**$^{13}$C NMR (75 MHz, acetone-d$_6$):** $\delta$ 14.38 ($CH_3$), 14.39 ($CH_3$), 23.4 (2 $CH_2$), 26.78 ($CH_2$), 30.45 ($CH_2$), 30.46 ($CH_2$), 30.48 ($CH_2$), 30.49 ($CH_2$), 30.59 ($CH_2$), 30.60 ($CH_2$), 30.71 ($CH_2$), 30.74 ($CH_2$), 30.75 ($CH_2$), 30.76 ($CH_2$), 30.78 ($CH_2$), 30.97 ($CH_2$), 30.99 ($CH_2$), 32.00 ($CH_2$), 32.02 ($CH_2$), 32.64 ($CH_2$), 32.66 ($CH_2$), 35.3 (CH), 50.5 (2 $CH_2$), 52.0 ($CH_2$), 67.4 (2 $CH_2$), 78.6 (CH), 83.9 ($C_{quat}$), 115.4 (CH), 115.8 (CH), 116.3 ($C_{quat}$), 116.5 (CH), 117.9 (CH), 126.1 ($C_{quat}$), 126.5 ($C_{quat}$), 131.0 (CH), 132.1 (CH), 138.3 ($C_{quat}$), 148.0 ($C_{quat}$), 148.8 ($C_{quat}$).

**MS (EI) m/z (%):** 645 ([M], 50), 321 ([$C_{19}H_{17}N_2OS$]$^+$, 35), 307 ([$C_{18}H_{15}N_2OS$]$^+$, 100).

**IR $\tilde{\nu}$ [cm$^{-1}$]**: 3316 (w), 2954 (w), 2920 (s), 2851 (s), 2332 (w), 2108 (w), 1605 (w), 1585 (w), 1501 (m), 1462 (s), 1149 (w), 1412 (w), 1398 (w), 1377 (w), 1362 (w), 1329 (w), 1302 (w), 1254 (s), 1227 (s), 1173 (w), 1123 (s), 1070 (w), 1053 (w), 1038 (w), 953 (s), 932 (w), 883 (w), 866 (w), 843 (w), 814 (m), 800 (w), 719 (w), 702 (w), 679 (w), 644 (m).

**Anal** calcd. for $C_{42}H_{64}N_2OS$ (645.1): C 78.21, H 10.00, N 4.34, S 4.97; Found: C 78.27, H 10.26, N 4.09, S 4.82.

**(*E*)-10-(2-Decyltetradecyl)-3-ethynyl-7-styryl-10*H*-phenothiazine (3p)**

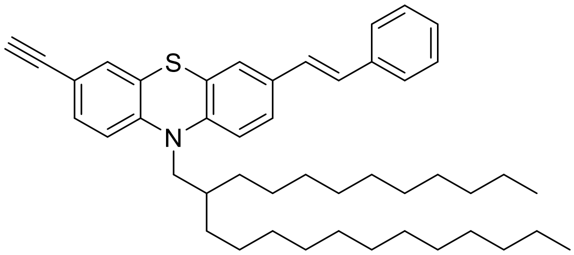

$C_{46}H_{63}NS$
[662.08]

According to **GP6** and after flash chromatography on silica gel (*n*-hexane/diethyl ether 70:1) compound **3p** (98 mg, 35%) was obtained as a yellow oil.

**R*f*** (*n*-hexane/diethyl ether 70:1): 0.80.

**$^1$H NMR (300 MHz, acetone-d$_6$):** $\delta$ 0.82 – 0.92 (m, 6H), 1.18 – 1.48 (m, 40H), 1.95 – 2.02 (m, 1H), 3.59 (s, 1H), 3.89 (d, $^3J$ = 7.2 Hz, 2H), 7.01 – 7.09 (m, 2H), 7.14 – 7.17 (m, 2H), 7.23 – 7.28 (m, 2 H), 7.29 – 7.34 (m, 2H), 7.34 – 7.39 (m, 1H), 7.40 – 7.46 (m, 2H), 7.54 – 7.60 (m, 2H).

**$^{13}$C NMR (75 MHz, acetone-d$_6$):** $\delta$ 14.38 ($CH_3$), 14.39 ($CH_3$), 23.4 (CH), 26.72 ($CH_2$), 26.76 ($CH_2$), 30.42 ($CH_2$), 30.45 ($CH_2$), 30.47 ($CH_2$), 30.49 ($CH_2$), 30.66 ($CH_2$), 30.68 ($CH_2$), 30.71 ($CH_2$), 30.75 ($CH_2$), 30.76 ($CH_2$), 30.78 ($CH_2$), 30.97 ($CH_2$), 30.98 ($CH_2$), 30.99 ($CH_2$), 31.9 ($CH_2$), 32.11 ($CH_3$), 32.67 ($CH_2$), 32.68 ($CH_2$), 52.1 ($CH_2$), 78.9 (CH), 83.6 ($C_{quat}$), 117.07 (CH), 117.13 ($C_{quat}$), 117.6 (CH), 125.8 (CH), 126.0 ($C_{quat}$), 126.1 ($C_{quat}$), 127.0 (CH), 127.2 (2 CH), 128.1 (CH), 128.23 (CH), 128.25 (CH), 129.5 (2 CH), 131.2 (CH), 132.2 (CH), 133.6 ($_{quat}$), 138.5 ($C_{quat}$), 145.3 ($C_{quat}$), 147.0 ($C_{quat}$).

**MS (EI) m/z (%):** 662 ([M], 80), 338 ([$C_{23}$H16NS]$^+$, 100), 324 ([$C_{22}H_{14}NS$]$^+$, 70), 306 (30), 57 ([$C_4H_9$]$^+$, 25).

**IR $\tilde{\nu}$ [cm$^{-1}$]**: 3304 (w), 3024 (w), 2953 (w), 2920 (s), 2851 (s), 2108 (w), 1688 (w), 1599 (w), 1580 (w), 1495 (m), 1460 (s), 1396 (w), 1377 (w), 1337 (m), 1304 (w), 1248 (m), 1196 (w), 1155 (w), 1144 (w), 1103 (w), 1072 (w), 1042 (w), 1028 (w), 1001 (w), 980 (w), 957 (m), 937 (w), 920 (w), 883 (w), 843 (w), 814 (m), 768 (w), 750 (m), 721 (w), 691 (m), 648 (w).

**Anal** calcd. for $C_{46}H_{63}NS$ (662.2): C 83.45, H 9.59, N 2.12, S 4.84; Found: C 83.19, H 9.39, N 2.15, S 4.82.

**(*E*)-10-(2-Decyltetradecyl)-3-ethynyl-7-(4-methoxystyryl)-10*H*-phenothiazine (3q)**

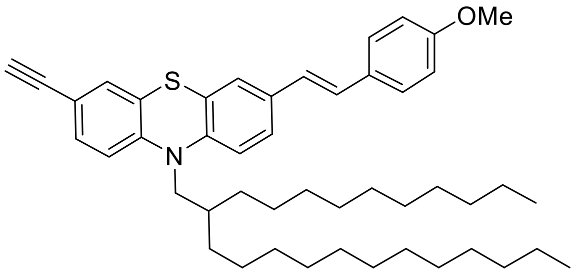


$C_{47}H_{65}NOS$
[692.10]

According to **GP6** and after flash chromatography on silica gel (*n*-hexane/diethyl ether 70:1) compound **3q** (190 mg, 63%) was obtained as a yellow oil.

**R*f*** (*n*-hexane/diethyl ether 70:1): 0.80.

**$^1$H NMR (300 MHz, acetone-d$_6$):** $\delta$ 0.82 – 0.92 (m, 6H), 1.17 – 1.48 (m, 40H), 1.96 – 2.03 (m, 1H), 3.60 (s, 1H), 3.81 (s, 3H), 3.88 (d, $^3J$ = 7.2 Hz, 2H), 6.93 (d, $^3J$ = 8.7 Hz, 2H), 7.01 – 7.06 (m, 3H), 7.07 – 7.15 (m, 1H), 7.25 (d, $^4J$ = 1.9 Hz, 1H), 7.31 (dd, $^3J$ = 8.4, $^4J$ = 1.9 Hz, 1H), 7.36 – 7.41 (m, 2H), 7.50 (d, $^3J$ = 8.7 Hz, 2H).

**$^{13}$C NMR (75 MHz, acetone-d$_6$):** $\delta$ 14.37 ($CH_3$), 14.39 ($CH_3$), 23.4 (CH), 26.72 ($CH_2$), 26.76 ($CH_2$), 30.42 ($CH_2$), 30.45 ($CH_2$), 30.47 ($CH_2$), 30.49 ($CH_2$), 30.66 ($CH_2$), 30.68 ($CH_2$), 30.71 ($CH_2$), 30.75 ($CH_2$), 30.76 ($CH_2$), 30.78 ($CH_2$), 30.97 ($CH_2$), 30.98 ($CH_2$), 30.99 ($CH_2$), 31.9 ($CH_2$), 32.11 ($CH_3$), 32.67 ($CH_2$), 32.68 ($CH_2$), 52.1 ($CH_3$), 52.6 ($CH_2$), 78.9 (CH), 83.6 ($C_{quat}$), 114.9 (2 CH), 117.0 (2 CH), 117.5 (CH), 125.5 ($C_{quat}$), 125.8 ($C_{quat}$), 125.9 ($C_{quat}$), 126.1 ($C_{quat}$), 126.6 (CH), 127.9 (CH), 128.5 (2 CH), 131.1 (2 CH), 132.2 (CH), 134.0 ($C_{quat}$), 144.8 ($C_{quat}$), 147.1 ($C_{quat}$), 160.3 ($C_{quat}$).

**MS (EI) m/z (%):** 692 ([M], 95), 368 ($[C_{24}H_{18}NOS]^+$, 90), 354 ($[C_{23}H_{16}NOS]^+$, 80), 336 (25), 57 ($[C_4H_9]^+$, 25).

**IR $\tilde{\nu}$ [cm$^{-1}$]**: 3306 (w), 3019 (w), 2920 (s), 2851 (m), 2108 (w), 1607 (m), 1578 (w), 1510 (s), 1460 (s), 1398 (w), 1337 (w), 1296 (w), 1248 (s), 1211 (w), 1173 (m), 1148 (w), 1107 (w), 1036 (m), 957 (m), 883 (w), 849 (w), 820 (s), 772 (w), 721 (w), 698 (w), 648 (w).

**Anal** calcd. for $C_{50}H_{64}N_4O_4S$ (692.1): C 81.57, H 9.47, N 2.02, S 4.63; Found: C 81.81, H 9.37, N 2.07, S 4.52.

**(*E*)-4-(2-(10-(2-Decyltetradecyl)-7-ethynyl-10*H*-phenothiazine-3-yl)vinyl)benzonitrile (3r)**

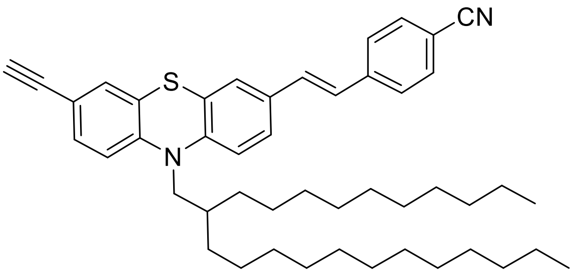

$C_{47}H_{62}N_2S$
[687.09]

According to **GP6** and after flash chromatography on silica gel (*n*-hexane/diethyl ether 15:1) compound **3r** (92 mg, 37%) was obtained as a yellow oil.

**R*f*** (*n*-hexane/diethyl ether 10:1): 0.70.

**$^1$H NMR (300 MHz, acetone-d$_6$):** $\delta$ 0.81 – 0.92 (m, 6H), 1.17 – 1.49 (m, 40H), 1.97 – 2.02 (m, 1H), 3.60 (s, 1H), 3.90 (d, $^2J$ = 7.2 Hz, 2H), 7.05 (d, $^3J$ = 8.4 Hz, 1H), 7.09 (d, $^3J$ = 8.3 Hz, 1H), 7.20 – 7.27 (m, 2H), 7.30 – 7.40 (m, 2H), 7.44 – 7.50 (m, 2H), 7.71 – 7.78 (m, 4H).

**$^{13}$C NMR (75 MHz, acetone-d$_6$):** $\delta$ 14.38 ($CH_3$), 14.40 ($CH_3$), 23.4 (CH), 26.72 ($CH_2$), 26.76 ($CH_2$), 30.42 ($CH_2$), 30.45 ($CH_2$), 30.47 ($CH_2$), 30.49 ($CH_2$), 30.66 ($CH_2$), 30.68 ($CH_2$), 30.71 ($CH_2$), 30.75 ($CH_2$), 30.76 ($CH_2$), 30.78 ($CH_2$), 30.97 ($CH_2$), 30.98 ($CH_2$), 30.99 ($CH_2$), 31.9 ($CH_2$), 32.11 ($CH_3$), 32.67 ($CH_2$), 32.68 ($CH_2$), 52.2 ($CH_2$), 79.0 (CH), 83.5 ($C_{quat}$), 111.0 ($C_{quat}$), 117.2 (CH), 117.3 ($C_{quat}$), 117.6 (CH), 119.5 ($C_{quat}$), 126.0 ($C_{quat}$), 126.1 ($C_{quat}$), 126.3 (CH), 126.4 (CH), 127.7 (CH), 127.8 (2 CH), 131.2 (CH), 131.9 (CH), 132.3 (CH), 132.8 ($C_{quat}$), 133.3 (2 CH), 143.2 ($C_{quat}$), 146.1 ($C_{quat}$), 146.8 ($C_{quat}$).

**MS (EI) m/z (%):** 687 ([M], 75), 363 ($[C_{24}H_{15}N_2S]^+$, 100), 349 ($[C_{23}H_{13}N_2S]^+$, 60), 331 (35), 57 ($[C_4H_9]^+$, 25).

**IR $\tilde{\nu}$ [cm$^{-1}$]**: 2920 (m), 2851 (m), 2224 (w), 1682 (m), 1599 (m), 1574 (m), 1551 (w), 1504 (m), 1460 (s), 1402 (w), 1377 (w), 1335 (s), 1323 (w), 1283 (w), 1248 (m), 1217 (w), 1196 (s), 1175 (w), 1146 (w), 1107 (m), 1040 (w), 1001 (w), 968 (m), 953 (w), 937 (w), 918 (w), 905 (w), 889 (w), 864 (m), 820 (s), 808 (w), 746 (w), 717 (s), 685 (m), 646 (w).

**Anal** calcd. for $C_{47}H_{62}N_2S$ (687.1): C 82.16, H 9.10, N 4.08, S 4.67; Found: C 82.38, H 9.37, N 4.04, S 4.66.

**(*E*)-10-(2-Decyltetradecyl)-3-ethynyl-7-(4-nitrostyryl)-10*H*-phenothiazine (3s)**

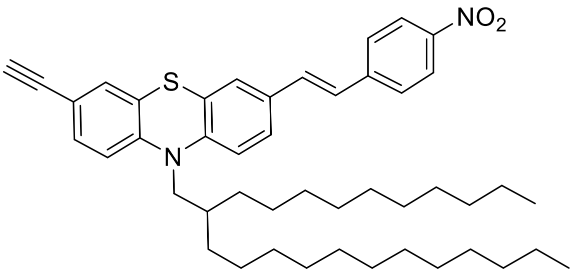

$C_{46}H_{62}N_2O_2S$
[707.07]

According to **GP6** and after flash chromatography on silica gel (*n*-hexane/diethyl ether 15:1) compound **3s** (83 mg, 25%) was obtained as a red oil.

**R*f*** (*n*-hexane/diethyl ether 15:1): 0.70.

**$^1$H NMR (600 MHz, acetone-d$_6$):** *δ* 0.85 – 0.88 (m, 6H), 1.18 – 1.43 (m, 40H), 1.99 – 2.04 (m, 1H), 3.61 (s, 1H), 3.91 (d, $^3J$ = 7.2 Hz, H), 7.06 (d, $^3J$ = 8.4 Hz, 1 H), 7.10 (d, $^3J$ = 8.4 Hz, 1H), 7.26 (d, $^4J$ = 1.9 Hz, 1H), 7.29 – 7.34 (m, 2H), 7.43 (d, $^3J$ = 16.4 Hz, 1H), 7.49 (d, $^4J$ = 2.0 Hz, 1H), 7.50 – 7.52 (m, 1H), 7.82 (d, $^3J$ = 8.9 Hz, 2H), 8.23 (d, $^3J$ = 9.0 Hz, 2H).

**$^{13}$C NMR (150 MHz, acetone-d$_6$):** *δ* 14.38 ($CH_3$), 14.40 ($CH_3$), 23.3 (CH), 26.73 ($CH_2$), 26.77 ($CH_2$), 30.42 ($CH_2$), 30.45 ($CH_2$), 30.47 ($CH_2$), 30.49 ($CH_2$), 30.66 ($CH_2$), 30.68 ($CH_2$), 30.71 ($CH_2$), 30.75 ($CH_2$), 30.76 ($CH_2$), 30.78 ($CH_2$), 30.97 ($CH_2$), 30.98 ($CH_2$), 30.99 ($CH_2$), 31.9 ($CH_2$), 32.11 ($CH_3$), 32.67 ($CH_2$), 32.68 ($CH_2$), 52.2 ($CH_2$), 79.0 (CH), 83.5 ($C_{quat}$), 117.3 (CH), 117.4 ($C_{quat}$), 117.6 (CH), 124.8 (2 CH), 125.9 (CH), 126.0 ($C_{quat}$), 126.1 ($C_{quat}$), 126.4 (CH), 127.8 (2 CH), 127.9 (CH), 131.2 (CH), 132.3 (CH), 132.7 ($C_{quat}$), 132.9 (CH), 145.4 ($C_{quat}$), 146.3 ($C_{quat}$), 146.7 ($C_{quat}$), 147.5 ($C_{quat}$).

**MS (EI) m/z (%):** 707 ([M], 85), 383 ($[C_{23}H_{15}N_2O_2S]^+$, 100), 369 ($[C_{22}H_{13}N_2O_2S]^+$, 35), 321 (20), 64 (15), 57 ($[C_4H_9]^+$, 25).

**IR $\tilde{\nu}$ [cm$^{-1}$]**: 3304 (w), 2920 (s), 2851 (m), 2108 (w), 1630 (w), 1591 (m), 1578 (m), 1506 (m), 1460 (s), 1400 (w), 1377 (w), 1337 (s), 1321 (w), 1300 (w), 1263 (w), 1250 (m), 1194 (m), 1159 (w), 1148 (w), 1109 (m), 1047 (w), 1013 (w), 966 (w), 953 (w), 937 (w), 893 (w), 862 (m), 827 (m), 808 (w), 748 (w), 743 (w), 723 (w), 712 (w), 691 (w), 648 (w).

**Anal** calcd. for $C_{46}H_{62}N_2O_2S$ (707.1): C 78.14, H 8.84, N 3.96, S 4.53; Found: C 78.30, H 9.10, N 3.85, S 4.46.

**(*E*)-10-(2-Decyltetradecyl)-3-ethynyl-7-(4-(trifluoromethyl)styryl)-10*H*-phenothiazine (3t)**

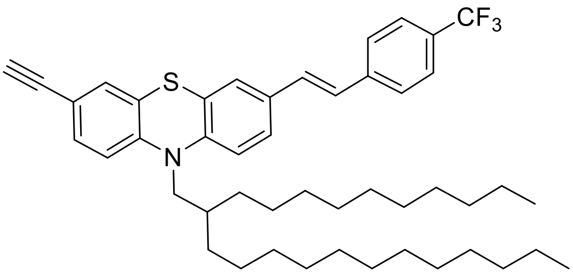

$C_{47}H_{62}F_3NS$
[730.08]

According to **GP6** and after flash chromatography on silica gel (*n*-hexane/diethyl ether 50:1) compound **3t** (36 mg, 36%) was obtained as a yellow oil.

**R***f* (*n*-hexane/diethyl ether 50:1): 0.80

**$^1$H NMR (300 MHz, acetone-d$_6$):** $\delta$ 0.81 – 0.93 (m, 6H), 1.09 – 1.53 (m, 40H), 1.97 – 2.02 (m, 1H), 3.60 (s, 1H), 3.90 (d, $^3J$ = 7.2 Hz, 2H), 7.00 – 7.13 (m, 2H), 7.20 – 7.28 (m, 2H), 7.29 – 7.37 (m, 2H), 7.43 – 7.52 (m, 2H), 7.68 (d, $^3J$ = 8.3 Hz, 2H), 7.78 (d, $^3J$ = 8.2 Hz, 2H).

**$^{13}$C NMR (75 MHz, acetone-d$_6$):** $\delta$ 14.37 ($CH_3$), 14.39 ($CH_3$), 23.4 (CH), 26.74 ($CH_2$), 26.78 ($CH_2$), 30.42 ($CH_2$), 30.45 ($CH_2$), 30.47 ($CH_2$), 30.49 ($CH_2$), 30.66 ($CH_2$), 30.68 ($CH_2$), 30.71 ($CH_2$), 30.75 ($CH_2$), 30.76 ($CH_2$), 30.78 ($CH_2$), 30.97 ($CH_2$), 30.98 ($CH_2$), 30.99 ($CH_2$), 31.9 ($CH_2$), 32.11 ($CH_3$), 32.66 ($CH_2$), 32.67 ($CH_2$), 35.4 (CH), 52.2 ($CH_2$), 79.0 (CH), 83.5 ($C_{quat}$), 117.2 (CH), 117.3 ($C_{quat}$), 117.6 (CH), 126.02 ($C_{quat}$), 126.05 ($C_{quat}$), 126.2 ($CF_3$), 126.37 (CH), 126.43 (CH), 126.5 (CH), 127.5 (CH), 127.6 (2 CH), 131.0 (CH), 131.2 (2 CH), 132.3 (CH), 132.9 ($C_{quat}$), 142.6 ($C_{quat}$), 145.9 ($C_{quat}$), 146.0 ($C_{quat}$), 146.9 ($C_{quat}$).

**MS (EI) m/z (%):** 730 ([M], 85), 406 ($[C_{24}H_{15}F_3NS]^+$, 100), 392 ($[C_{23}H_{13}F_3NS]^+$, 60), 374 (35), 264 (25), 57 ($[C_4H_9]^+$, 25).

**IR $\tilde{\nu}$ [cm$^{-1}$]**: 3306 (w), 2922 (s), 2853 (w), 2641 (w), 2108 (w), 1612 (w), 1601 (w), 1580 (w), 1495 (w), 1460 (s), 1416 (w), 1400 (w), 1377 (w), 1321 (s), 1248 (m), 1184 (w), 1165 (m), 1125 (s), 1109 (w), 1067 (s), 1045 (w), 1015 (m), 961 (w), 949 (w), 883 (w), 862 (w), 822 (s), 721 (w), 710 (w), 648 (w).

**Anal** calcd. for $C_{47}H_{62}F_3NS$ (730.1): C 77.32, H 8.56, N 1.92, S 4.39; Found: C 77.53, H 8.81, N 1.89, S 4.48.

**(*E*)-4-(2-(10-(2-Decyltetradecyl)-7-ethynyl-10*H*-phenothiazine-3-yl)vinyl)-*N*,*N*-diphenylaniline (3u)**

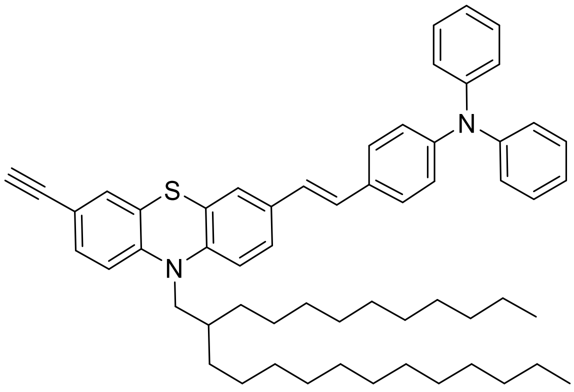

$C_{58}H_{72}N_2S$
[829.29]

According to **GP6** and after flash chromatography on silica gel (*n*-hexane/diethyl ether 50:1) compound **3u** (55 mg, 22%) was obtained as a yellow oil.

**R***f* (*n*-hexane/diethyl ether 50:1): 0.85

**$^1$H NMR (300 MHz, acetone-d$_6$):** $\delta$ 0.84 – 0.90 (m, 6H), 1.17 – 1.45 (m, 40H), 1.90 – 2.08 (m, 1H), 3.59 (s, 1H), 3.87 (d, $^3J$ = 7.1 Hz, 2H), 6.96 – 7.11 (m, 12H), 7.24 (d, $^3J$ = 1.9 Hz, 1H), 7.26 – 7.33 (m, 5H), 7.36 – 7.41 (m, 2H), 7.44 – 7.51 (m, 2H).

**$^{13}$C NMR (75 MHz, acetone-d$_6$):** $\delta$ 14.41 ($CH_3$), 14.42 ($CH_3$), 23.4 (CH), 26.75 ($CH_2$), 26.79 ($CH_2$), 30.42 ($CH_2$), 30.45 ($CH_2$), 30.47 ($CH_2$), 30.49 ($CH_2$), 30.66 ($CH_2$), 30.68 ($CH_2$), 30.71 ($CH_2$), 30.75 ($CH_2$), 30.76 ($CH_2$), 30.78 ($CH_2$), 30.97 ($CH_2$), 30.98 ($CH_2$), 30.99 ($CH_2$), 32.02 ($CH_2$), 32.11 ($CH_3$), 32.66 ($CH_2$), 32.67 ($CH_2$), 35.3 (CH), 52.1 ($CH_2$), 78.9 (CH), 83.6 ($C_{quat}$), 117.0 (CH), 117.1 ($C_{quat}$), 117.5 (CH), 124.0 (2 CH), 124.3 (2 CH), 125.2 (4 CH), 125.6 (CH), 125.9 ($C_{quat}$), 126.1 ($C_{quat}$), 126.6 (CH), 126.8 (CH), 127.8 (CH), 128.2 (2 CH), 130.3 (4 CH), 131.2 (CH), 132.2 (CH), 132.9 ($C_{quat}$), 133.9 ($C_{quat}$), 145.0 ($C_{quat}$), 147.1 ($C_{quat}$), 148.1 ($C_{quat}$), 148.5 (2 $C_{quat}$).

**MS (EI) m/z (%):** 829 ([M], 5), 587 (65), 264 (100), 251 (40).

**IR $\tilde{\nu}$ [cm$^{-1}$]**: 3300 (w), 3026 (w), 2922 (s), 2851 (s), 2108 (w), 1722 (w), 1688 (w), 1589 (s), 1545 (w), 1506 (s), 1493 (s), 1460 (s), 1396 (w), 1377 (w), 1329 (m), 1314 (w), 1275 (s), 1250 (w), 1211 (w), 1177 (w), 1155 (w), 1103 (w), 1074 (w), 1042 (w), 1028 (w), 1015 (w), 999 (w), 959 (w), 941 (w), 920 (w), 883 (w), 854 (w), 818 (s), 752 (s), 721 (w), 694 (s), 644 (w), 621 (w).

**Anal** calcd. for $C_{58}H_{72}N_2S$ (829.3): C 84.00, H 8.75, N 3.38, S 3.87; Found: C 84.24, H 8.74, N 3.36, S 3.88.

**(*E*)-10-(2-Decyltetradecyl)-3-(2-(10-(2-decyltetradecyl)-10*H*-phenothiazine-3-yl)vinyl)-7-ethynyl-10*H*-phenothiazine (3v)**

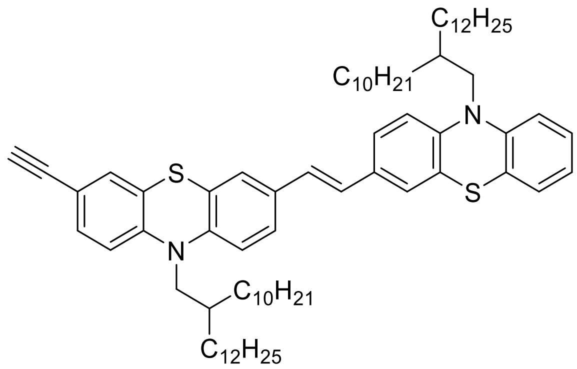

$C_{76}H_{114}N_2S_2$
[1119.88]

According to **GP6** and after flash chromatography on silica gel (*n*-hexane/diethyl ether 50:1) compound **3v** (132 mg, 27%) was obtained as a yellow oil.

**R***f* (*n*-hexane/diethyl ether 50:1): 0.80

**$^1$H NMR (300 MHz, acetone-d$_6$):** $\delta$ 0.85 – 0.91 (m, 12H), 1.23 – 1.44 (m, 80H), 2.04 – 2.06 (m, 1H), 3.86 – 3.89 (m, 4H), 6.91 – 6.98 (m, 1H), 7.00 – 7.10 (m, 6H), 7.14 – 7.21 (m, 3H), 7.24 (d, $^3J$ = 1.9 Hz, 1H), 7.29 – 7.32 (m, 1H), 7.36 – 7.40 (m, 3H)

14.4 (4 $CH_3$), 23.4 (4 $CH_2$), 26.7 (2 $CH_2$), 26.8 ($CH_2$), 26.8 (2 $CH_2$), 26.9 (2 $CH_2$), 31.9 (2 $CH_2$), 32.1 (4 $CH_2$), 32.7 (4 $CH_2$), 35.3 ($CH_2$), 35.4 ($CH_2$), 52.1 ($CH_2$), 52.5 ($CH_2$),

**$^{13}$C NMR (75 MHz, acetone-d$_6$):** $\delta$ 14.7 (4 $CH_3$), 23.6 (4 $CH_2$), 27.04 (2 $CH_2$), 27.08 (2 $CH_2$), 27.11 (2 $CH_2$), 32.12 (2 $CH_2$), 32.25 (2 $CH_2$), 32.28 (2 $CH_2$), 32.35 ($CH_2$), 35.41 ($CH_2$), 35.5 ($CH_2$), 52.15 ($CH_2$), 52.20 ($CH_2$), 78.9 (CH), 83.7 ($C_{quat}$), 116.8 (CH), 117.0 (CH), 117.30 (CH), 117.34 (CH), 123.3 (CH), 123.8 (CH), 125.7 (CH), 126.0 ($C_{quat}$), 126.1 ($C_{quat}$), 126.6 (CH), 126.7 (CH), 126.8 ($C_{quat}$), 127.1 (CH), 128.2 (CH), 128.3 (CH), 131.20 (CH), 131.24 (CH), 132.1 (CH), 133.1 ($C_{quat}$), 133.7 ($C_{quat}$), 144.9 ($C_{quat}$), 145.7 ($C_{quat}$), 145.9 ($C_{quat}$), 146.3 ($C_{quat}$), 146.9 ($C_{quat}$), 147.2 ($C_{quat}$).

**MS MALDI:** 1119.05 [M]

**IR $\tilde{\nu}$ [cm$^{-1}$]:** 3304 (w), 2951 (w), 2920 (s), 2851 (m), 2108 (w), 1599 (w), 1491 (w), 1460 (s), 1445 (w), 1396 (w), 1339 (w), 1288 (w), 1248 (m), 1161 (w), 1132 (w), 1103 (w), 1038 (w), 1020 (w), 1003 (w), 995 (w), 955 (w), 928 (w), 883 (w), 814 (m), 745 (w), 719 (w), 696 (w), 669 (w), 648 (w).

**Anal** calcd. for $C_{76}H_{114}N_2S_2$ (1119.9): C 81.51, H 10.26, N 2.50, S 5.73; Found: C 81.52, H 10.51, N 2.48, S 5.56.

### 2.2.2 General procedure VII (GP7) for the Sonogashira coupling

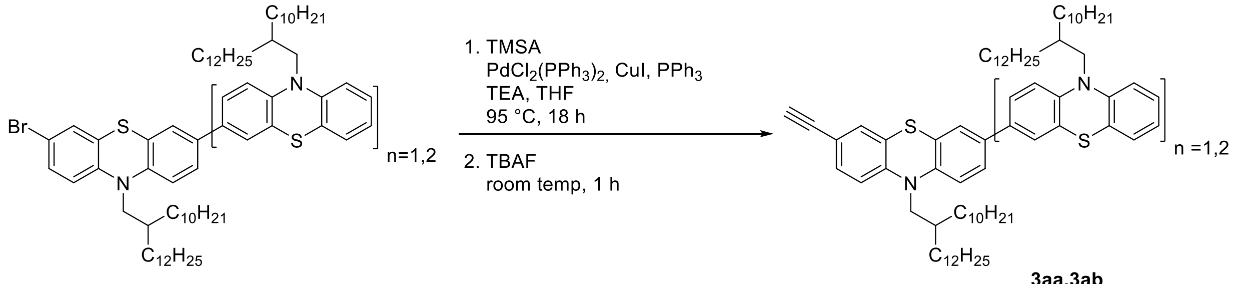


The mono-brominated phenothiazine **1aa** or **1ab** (1.0 equiv) bis(triphenylphosphane)palladium(II) dichloride (3 mol%), Cu(I)I (6 mol%) and triphenylphosphane (6 mol%) were placed in a dry Schlenk tube under inert atmosphere and dissolved in 1.5 mL/mmol dry THF and 7.5 mL/mmol dry triethylamine. After addition of 1 trimethylsilylacetylene (1.5 equivs) the solution was degassed for 15 min with nitrogen and then stirred at 95 °C (oil bath) for 18 h. After cooling down to room temp a 1M solution of TBAF (1.8 equivs) was added via syringe under nitrogen atmosphere. The reaction mixture was stirred at room temp for 1 h. Then 10 mL/mmol of a saturated $NH_4Cl$ solution were added. After additional 15 min the crude was transferred to a separating funnel and the aqueous phase was extracted three times with dichloromethane. The organic phases were dried with anhydrous magnesium sulfate and filtered. The crude product was adsorbed on Celite® and purified via flash chromatography on silica gel.

**Table S7.** Experimental details for the synthesis of alkynylated oligophenothiazines **3aa-3ab**.

| entry | bromine | [Pd] | CuI | $PPh_3$ | TEA | THF | TBAF | alkyne |
|---|---|---|---|---|---|---|---|---|
| 1 | 0.450 g (0.39 mmol) of **1aa** | 8.2 mg (0.011 mmol) | 2.2 mg (0.011 mmol) | 6.1 mg (0.022 mmol) | 3 mL | 0.6 mL | 0.7 mL | 0.148 g (35%) of **3aa** |
| 2 | 0.300 g (0.18 mmol) **1ab** | 3.8 mg (0.005 mmol) | 1.0 mg (0.005 mmol) | 2.8 mg (0.010 mmol) | 1.35 mL | 0.3 mL | 0.3 mL | 0.176 g (61%) of **3ab** |

**10,10'-bis(2-decyltetradecyl)-7-ethynyl-10*H*,10'*H*-3,3'-biphenothiazine (3aa)**

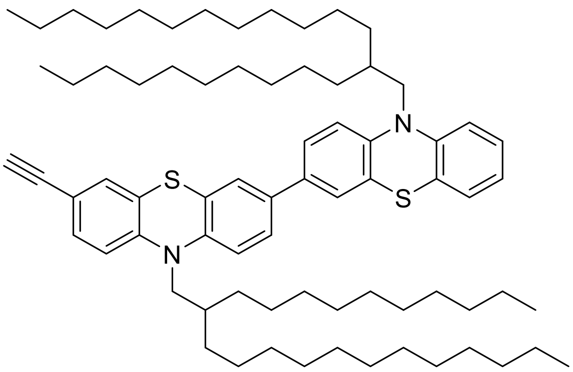

$C_{74}H_{112}N_2S_2$
[1093.84]

According to **GP7** and after flash chromatography on silica gel (*n*-hexane/dichloromethane 10:1) compound **3aa** (148 mg, 35%) was obtained as a yellow oil.

**$R_f$** (*n*-hexane/dichloromethane 10:1): 0.55.

**$^1$H NMR (300 MHz, THF-d$_8$):** $\delta$ 0.82 – 0.92 (m, 12H), 1.16 – 1.50 (m, 80H), 1.91 – 2.08 (m, 2H), 3.46 (s, 1 H), 3.76-3.88 (m, 4H), 6.84 – 7.03 (m, 5H), 7.09-7.17 (m, 2H), 7.20 – 7.28 (m, 2H), 7.34 – 7.42 (m, 4H).

**$^{13}$C NMR (75 MHz, THF-d$_8$):** $\delta$ 14.5 (4 $CH_3$), 23.6 (2 $CH_2$), 25.5 (2 $CH_2$), 25.7 (2 $CH_2$), 26.0 (2 $CH_2$), 27.18 (2 $CH_2$), 27.23 (2 $CH_2$), 30.36 (2 $CH_2$), 30.45 (2 $CH_2$), 30.47 (2 $CH_2$), 30.61 (2 $CH_2$), 30.66 (2 $CH_2$), 30.68 (2 $CH_2$), 30.69 (2 $CH_2$), 31.0 (2 $CH_2$), 32.5(2 $CH_2$), 32.6 (2 $CH_2$), 33.0 (2 $CH_2$), 35.6 (CH), 35.7 (CH), 52.2 ( 2 $CH_2$), 52.3 ( 2 $CH_2$), 67.5 (2 $CH_2$), 67.8 (2 $CH_2$), 68.1 (2 $CH_2$) 78.5 ($C_{quat}$), 83.7 (CH), 116.6 (2 CH), 116.9 (CH), 117.1 (CH), 117.3 (CH), 123.2 (CH), 125.86 (CH), 125.92 (2), 126.47 ($C_{quat}$), 126.50 (2 $C_{quat}$), 126.52 ($C_{quat}$), 127.3 ($C_{quat}$), 128.0 (CH), 128.2 (CH), 131.4 (CH), 132.0 (CH), 135.1 ($C_{quat}$), 135.8 ($C_{quat}$), 145.1 ($C_{quat}$), 145.9 ($C_{quat}$), 146.7 ($C_{quat}$), 146.1 ($C_{quat}$).

**MALDI-MS (m/*z*):** 1094.8 $[M]^+$.

**IR $\tilde{\nu}$ [cm$^{-1}$]:** 3308 (w), 3061 (w), 2953 (w), 2920 (s), 2851 (m), 2110 (w), 1603 (w), 1578 (w), 1491 (w), 1456 (s), 1416 (w), 1396 (w), 1377 (w), 1337 (w), 1248 (m), 1186 (w), 1165 (w), 1132 (w), 1105 (W), 1082 (w), 1038 (w), 1028 (w), 984 (w), 907 (w), 876 (w), 854 (w), 808 (m), 746 (m), 721 (w), 708 (w), 694 (w), 683 (w), 637 (w).

**Anal** calcd for $C_{74}H_{112}N_2S_2$ (1093.8): C 81.26, H 10.32, N 2.56, S 5.86; Found: C 81.04, H 10.24, N 2.54, S 5.71.

**10,10',10''-Tris(2-decyltetradecyl)-7-ethynyl-10*H*,10'*H*,10''*H*-[3,3',7',3'']-terphenothiazine (3ab)**

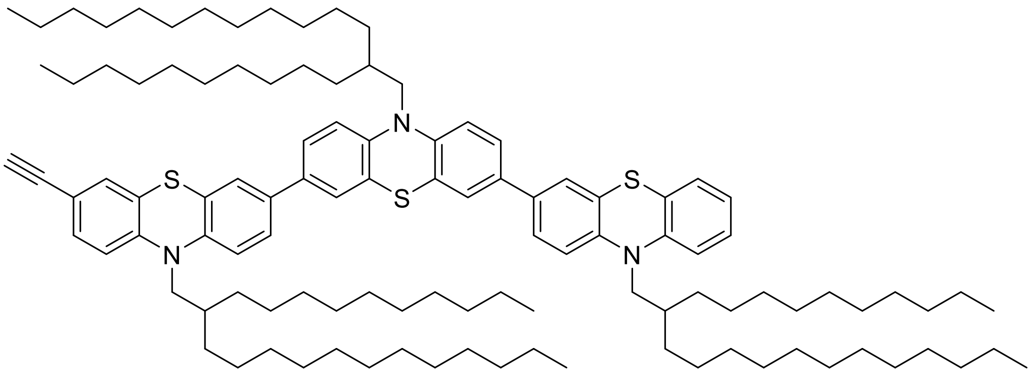

$C_{110}H_{167}N_3S_3$
[1627.75]

According to **GP7** and after flash chromatography on silica gel (*n*-hexane/dichloromethane 10:1) compound **3ab** (176 mg, 61%) was obtained as a yellow oil.

**$R_f$** (*n*-hexane/dichloromethane 10:1): 0.42.

**$^1$H NMR (300 MHz, acetone-d$_6$):** $\delta$ 0.84 – 0.94 (m, 18H), 1.24 – 1.49 (m, 120H), 2.07 – 2.08 (m, 1H), 3.50 (s, 1H), 3.83 – 3.93 (m, 6H), 6.90 – 6.98 (m, 1H), 6.97 – 7.97 (m, 5H), 7.12 – 7.19 (m, 2H), 7.22 (d, $^4J$ = 1.9 Hz, 1H), 7.29 (dd, $^3J$ = 8.4, $^4J$ = 1.9 Hz, 1H), 7.33 – 7.46 (m, 9H).

**$^{13}$C NMR (75 MHz, acetone-d$_6$):** $\delta$ 14.7 (3CH$_3$), 14.8 (3CH$_3$), 23.6 (2 CH$_2$), 26.71 (CH$_2$), 26.73 (3CH$_2$), 27.1 (3CH$_2$), 27.2 (3CH$_2$), 30.3 (3CH), 30.4 (3CH) 32.3 (3CH$_2$), 32.4 (3CH$_2$), 32.8 (3CH$_2$), 35.5 (3CH$_2$), 52.4 (3CH$_2$), 78.81 (C$_{quat}$), 83.78 (CH), 116.6 (CH), 116.9 (CH), 117.1 (3CH), 117.4 (CH), 123.2 (CH), 125.8 (2CH), 126.0 (2CH), 126.16 (2CH), 126.22 (2CH), 126.6 (2CH), 126.7 (2CH), 127.0 (CH), 128.2 (CH), 129.2 (CH), 129.3 (CH), 131.3 (CH), 132.1 (CH), 132.6 (CH), 132.7 (CH), 134.8 (2C$_{quat}$), 135.0 (C$_{quat}$), 135.1 (C$_{quat}$), 135.5 (C$_{quat}$), 144.6 (2C$_{quat}$), 145.2 (2C$_{quat}$), 145.4 (2C$_{quat}$), 146.4 (2C$_{quat}$), 146.9 (2C$_{quat}$).

**MALDI-MS (m/*z*):** 1626.2 [M]$^+$.

**IR $\tilde{\nu}$ [cm$^{-1}$]:** 3904 (w), 3692 (w), 2955 (w), 2922 (s), 2853 (m), 2723 (w), 2359 (w), 1607 (w), 1456 (s), 1418 (w), 1377 (w), 1335 (w), 1306 (w), 1250 (m), 1236 (w), 1188 (w), 1150 (w), 1134 (w), 1105 (w), 1076 (w), 1057 (w), 1042 (w), 1028 (w), 968 (w), 908 (w), 876 (w), 808 (m), 746 (w), 712 (w), 648 (w).

**Anal** calcd for $C_{110}H_{167}N_3S_3$ (1626.2) C 81.17, H 10.34, N 2.58, S 5.91; Found: C 80.90, H 10.13, N 2.53, S 5.64.

## 2.3 Synthesis and analytical data of the polymers

### 2.3.1 General procedure VIII (GP8) for the polymerization of the alkynylated Phenothiazines

Preparation of the catalyst solution (The solution was prepared in a 10-fold excess):

$[Rh(nbd)Cl]_2$ (0.02 equivs, 0.14 mg, 0.3 µmol), 4-propoxyphenylboronic acid (0.06 equivs, 0.16 mg, 0.9 µmol) and diphenylacetylene (0.16 equivs, 0.43 mg, 2.4 µmol,) were placed in a Schlenk tube under nitrogen atmosphere and dissolved in 30 µL dry THF. Then the reaction mixture was cooled down to 0 °C with an ice bath. After 5 min, an 50% (w/v) aqueous solution of KOH (0.1 equivs, 0.172 µL, 1.5 µmol) of was added. The resulting solution was stirred for another 5 min. Finally, 0.47 mg (1.8 µmol, 0.16 equivs) of triphenylphosphane was added under nitrogen atmosphere.

Polymerization:

Monomer **3** (1.0 equiv, 0.08 mmol) was placed in a Schlenk tube under nitrogen atmosphere and dissolved in 0.2 mL dry THF. An aliquot of the catalyst solution was rapidly added to the monomer solution under nitrogen atmosphere. The reaction mixture was stirred at 30 °C for 1 h. Finally, the polymerization was quenched by a few drops of acetic acid. The resulting polymer was poured into 10 mL of methanol. The precipitate was suspended in methanol, sonicated in an ultrasonic bath and centrifugated (**3x**).

**Table S8.** Experimental details for the synthesis of phenothiazinyl polymers **4a-v**.

| entry | monomer 3 | Yield of polymer 4 |
|---|---|---|
| **1** | 51 mg (0.08 mmol) of **3a** | 39 mg (77%) of **4a** |
| **2** | 53 mg (0.08 mmol) of **3b** | 42 mg (80%) of **4b** |
| **3** | 52 mg (0.08 mmol) of **3c** | 36 mg (70%) of **4c** |
| **4** | 55 mg (0.08 mmol) of **3d** | 41 mg (74%) of **4d** |
| **5** | 60 mg (0.08 mmol) of **3e** | 49 mg (82%) of **4e** |
| **6** | 64 mg (0.08 mmol) of **3f** | 55 mg (86%) of **4f** |
| **7** | 69 mg (0.08 mmol) of **3g** | 54 mg (79%) of **4g** |
| **8** | 54 mg (0.08 mmol) of **3h** | 39 mg (70%) of **4h** |

| | | |
|---|---|---|
| **9** | 55 mg (0.08 mmol) of **3i** | 30 mg (55%) of **4i** |
| **10** | 45 mg (0.08 mmol) of **3aa** | 32 mg (72%) of **4aa** |
| **11** | 73 mg (0.08 mmol) of **3ab** | 50 mg (68%) of **4ab** |
| **12** | 117 mg (0.08 mmol) of **3j** | 70 mg (60%) of **4j** |
| **13** | 60 mg (0.08 mmol) of **3k** | 45 mg (75%) of **4k** |
| **14** | 45 mg (0.08 mmol) of **3l** | 38 mg (84%) of **4l** |
| **15** | 63 mg (0.08 mmol) of **3m** | 48 mg (76%) of **4m** |
| **16** | 58 mg (0.08 mmol) of **3n** | 47 mg (85%) of **4n** |
| **17** | 60 mg (0.08 mmol) of **3o** | 43 mg (72%) of **4o** |
| **18** | 66 mg (0.08 mmol) of **3p** | 52 mg (79%) of **4p** |
| **19** | 53 mg (0.08 mmol) of **3q** | 40 mg (75%) of **4q** |
| **20** | 55 mg (0.08 mmol) of **3r** | 37 mg (67%) of **4r** |
| **21** | 55 mg (0.08 mmol) of **3s** | 46 mg (83%) of **4s** |
| **22** | 56 mg (0.08 mmol) of **3t** | 44 mg (78%) of **4t** |
| **23** | 58 mg (0.08 mmol) of **3u** | 42 mg (72%) of **4u** |
| **24** | 66 mg (0.08 mmol) of **3v** | 45 mg (68%) of **4v** |

**Poly[10-(2-decyltetradecyl)-3-ethynyl-7-phenyl-10*H*-phenothiazine] (4a)**

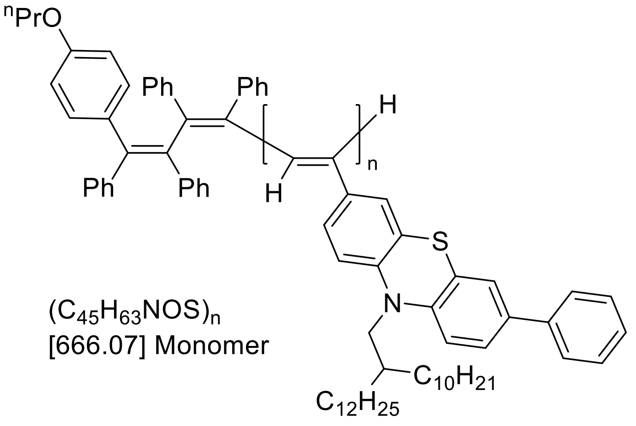


The synthesis was performed according to **GP8** to give (39 mg, 77%) of the product **4a** as an orange solid.

**IR $\tilde{\nu}$ [cm$^{-1}$]:** 3028 (w), 2953 (w), 2920 (s), 2851 (s), 1601 (m), 1582 (w), 1460 (s), 1396 (w), 1377 (w), 1335 (m), 1300 (w), 1277 (w), 1250 (m), 1217 (w), 1196 (w), 1190 (w), 1150 (w), 1103 (w), 1092 (w), 1070 (w), 1049 (w), 1042 (w), 1022 (w), 943 (w), 891 (m), 839 (w), 818 (s), 789 (w), 758 (s), 719 (m), 696 (s), 669 (w).

**GPC-RI-LS (THF):** $M_w$ = 82990 Da, $M_n$ = 73330, $M_w/M_n$ = 1.13.

**Poly[10-(2-decyltetradecyl)-3-ethynyl-7-(4-methoxyphenyl)-10*H*-phenothiazine] (4b)**

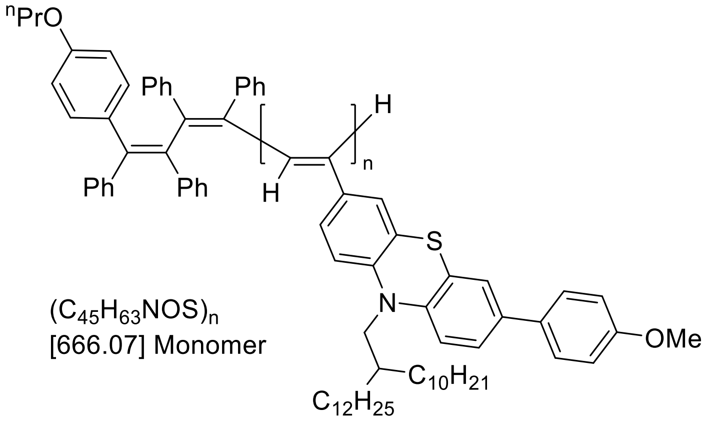


The synthesis was performed according to **GP5** to give (42 mg, 80%) of the product **4b** as an orange solid.

**IR $\tilde{\nu}$ [cm$^{-1}$]:** 2995 (w), 2920 (s), 2851 (m), 1609 (m), 1580 (w), 1518 (w), 1493 (w), 1460 (s), 1441 (w), 1423 (w), 1391 (w), 1377 (w), 1335 (m), 1308 (w), 1288 (w), 1279 (w), 1244 (s), 1179 (m), 1152 (w), 1107 (w), 1076 (w), 1049 (m), 1028 (w), 881 (w), 831 (w), 804 (s), 770 (w), 719 (w), 696 (w), 667 (w), 660 (w), 633 (w), 613 (w).

**GPC-RI-LS (THF):** $M_w$ = 102820 Da, $M_n$ = 106540, $M_w/M_n$ = 1.06.

**Poly[4-(10-(2-decyltetradecyl)-7-ethynyl-10*H*-phenothiazin-3-yl)benzonitrile] (4c)**

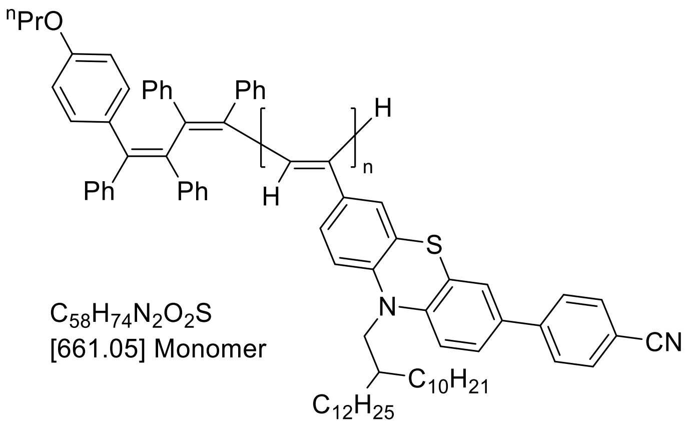

$C_{58}H_{74}N_2O_2S$
[661.05] Monomer

The synthesis was performed according to **GP8** to give (36 mg, 70%) of the product **4c** as an orange solid.

**IR $\tilde{\nu}$ [cm$^{-1}$]:** 3036 (w), 2922 (s), 2851 (s), 2521 (w), 2226 (m), 2162 (w), 2145 (w), 1967 (w), 1605 (m), 1582 (w), 1460 (s), 1418 (w), 1393 (w), 1377 (w), 1335 (m), 1300 (w), 1275 (w), 1250 (m), 1179 (w), 1163 (w), 1107 (w), 1045 (w), 1016 (w), 883 (w), 841 (m), 812 (s), 752 (w), 719 (w), 698 (w), 646 (w).

**GPC-RI-LS (THF):** $M_w$ = 137820 Da, $M_n$ = 133030 Da, $M_w/M_n$ = 1.04.

**Poly[10-(2-decyltetradecyl)-3-ethynyl-7-(4-nitrophenyl)-10*H*-phenothiazine] (4d)**

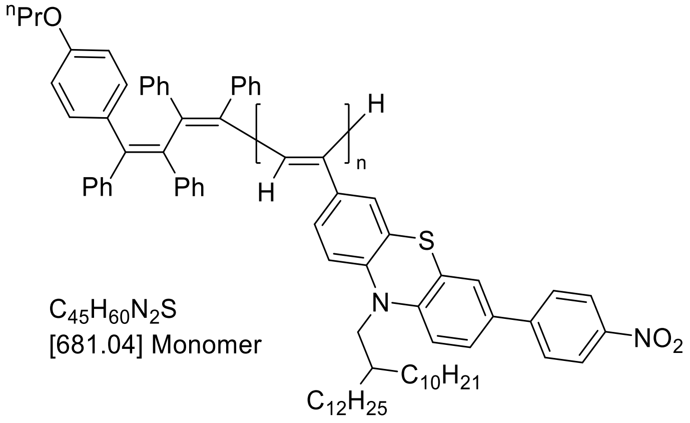

$C_{45}H_{60}N_2S$
[681.04] Monomer

The synthesis was performed according to **GP8** to give (41 mg, 74%) of the product **4d** as a red solid.

**IR $\tilde{\nu}$ [cm$^{-1}$]:** 3304 (w), 3277 (w), 2920 (s), 2851 (m), 2108 (w), 1595 (m), 1580 (w), 1539 (w), 1514 (m), 1460 (s), 1421 (w), 1393 (w), 1377 (w), 1337 (s), 1298 (w), 1281 (w), 1263 (w), 1250 (m), 1217 (w), 1186 (w), 1163 (w), 1152 (w), 1109 (w), 1047(w), 1034 (w), 1013 (w), 957 (w), 939 (w), 885 (w), 853 (m), 816 (m), 793 (w), 754 (m), 741 (w), 700 (w), 650 (w).

**GPC-RI-LS (THF):** $M_w$ = 122720 Da, $M_n$ = 85999, $M_w/M_n$ = 1.43.

**Poly[10-(2-decyltetradecyl)-3-ethynyl-7-(4-(trifluoromethyl)phenyl)-10*H*-phenothiazine] (4e)**

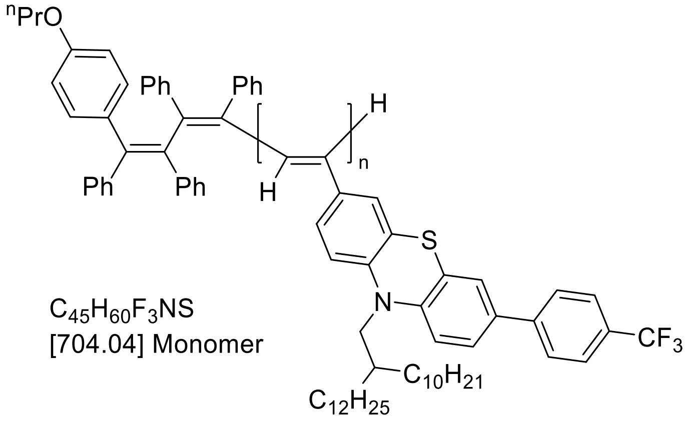


$C_{45}H_{60}F_3NS$
[704.04] Monomer

The synthesis was performed according to **GP8** to give (49 mg, 82%) of the product **4e** as an orange solid.

**IR $\tilde{\nu}$ [cm$^{-1}$]:** 2920 (m), 2851 (m), 1460 (s), 1323 (s), 1302 (w), 1279 (w), 1261 (w), 1248 (w), 1215 (w), 1165 (m), 1125 (m), 1109 (w), 1072 (m), 843 (w), 814 (m).

**GPC-RI-LS (THF):** $M_w$ = 140230 Da, $M_n$ = 134610 Da, $M_w/M_n$ = 1.04.

**Poly[4-(10-(2-decyltetradecyl)-7-ethynyl-10*H*-phenothiazin-3-yl)-*N*,*N*-diphenylaniline] (4f)**

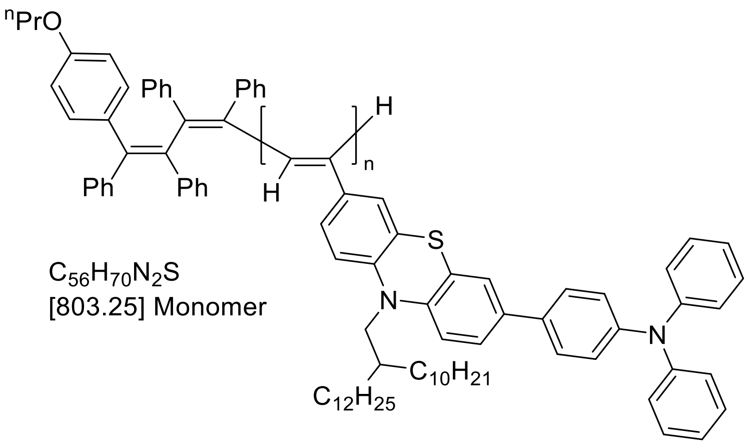


$C_{56}H_{70}N_2S$
[803.25] Monomer

The synthesis was performed according to **GP8** to give (55 mg, 86%) of the product **4f** as an orange solid.

**IR $\tilde{\nu}$ [cm$^{-1}$]:** 2920 (s), 2851 (s), 1587 (m), 1500 (m), 1464 (s), 1328 (m), 1314 (w), 1273 (m), 1254 (w), 1215 (w), 1153 (w), 812 (m), 750 (s), 694 (s).

**GPC-RI-LS (THF):** $M_w$ = 310690 Da, $M_n$ = 293550 Da, $M_w/M_n$ = 1.05.

**Poly[4-(10-(2-decyltetradecyl)-7-ethynyl-10*H*-phenothiazin-3-yl)-*N*,*N*-bis(4 methoxyphenyl)aniline] (4g)**

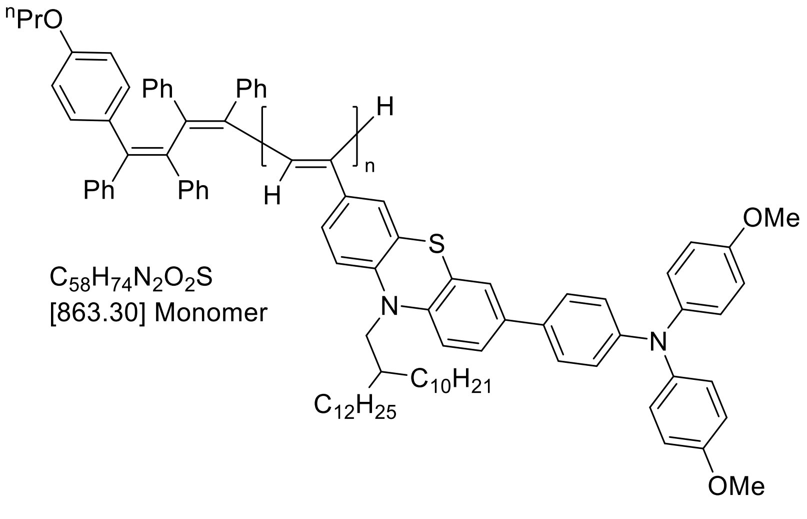


$C_{58}H_{74}N_2O_2S$
[863.30] Monomer

The synthesis was performed according to **GP8** to give (mg, %) of the product **4g** as an orange solid.

**IR $\tilde{\nu}$ [cm$^{-1}$]:** 2920 (m), 2851 (m), 1603 (w), 1503 (s), 1460 (s), 1441 (w), 1317 (w), 1238 (s), 1179 (w), 1165 (w), 1105 (w), 1035 (m), 826 (m), 812 (m), 725 (w).

**GPC-RI-LS (THF):** $M_w$ = 106540 Da, $M_n$ = 102820 Da, $M_w/M_n$ = 1.06.

### Poly[10-(2-Decyltetradecyl)-3-ethynyl-7-(quinolin-3-yl)-10*H*-phenothiazine] (4h)

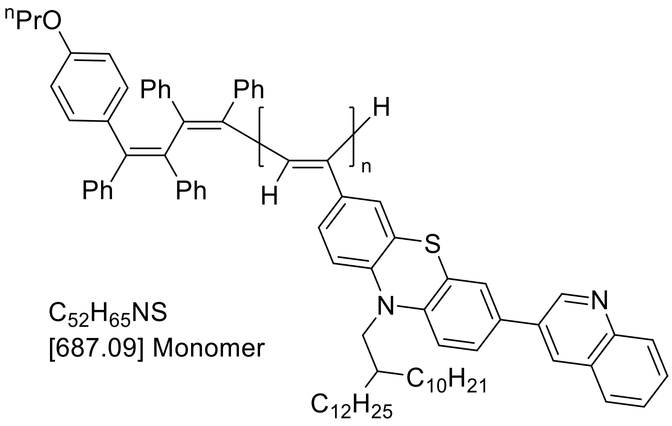


The synthesis was performed according to **GP5** to give (54 mg, 79%) of the product **4h** as an orange solid.

**IR $\tilde{\nu}$ [cm$^{-1}$]:** 2922 (s), 2851 (s), 1603 (w), 1582 (w), 1503 (w), 1456 (s), 1423 (w), 1396 (w), 1341 (m), 1290 (w), 1260 (s), 1229 (w), 1153 (w), 1142 (w), 1103 (m), 1015 (m), 962 (w), 908 (w), 876 (w), 858 (w), 810 (s), 785 (m), 748 (s), 721 (w), 698 (w), 662 (w), 619 (w).

**GPC-RI-LS (THF):** $M_w$ = 284960 Da, $M_n$ = 263140 Da, $M_w/M_n$ = 1.08.

**Poly[3-(anthracen-9-yl)-10-(2-decyltetradecyl)-7-ethynyl-10*H*-phenothiazine] (4i)**

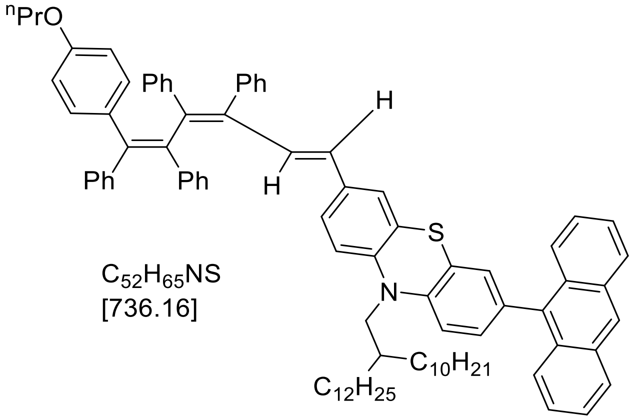

$C_{52}H_{65}NS$
[736.16]

The synthesis was performed according to **GP8** to give (30 mg, 55%) of the product **4i** as an orange solid.

**IR $\tilde{\nu}$ [cm$^{-1}$]:** 3049 (w), 2922 (s), 2851 (s), 2359 (w), 1454 (s), 1441 (w), 1412 (w), 1393 (w), 1356 (m), 1331 (w), 1296 (w), 1250 (m), 1219 (w), 1103 (w), 1042 (w), 1015 (w), 955 (w), 881 (m), 843 (m), 814 (m), 791 (m), 756 (w), 733 (s), 721 (w).

**GPC-RI-LS (THF):** $M_w$ = 73436 Da, $M_n$ = 39986 Da, $M_w/M_n$ = 1.83.

**Poly[10,10'-bis(2-decyltetradecyl)-7-ethynyl-10*H*,10'*H*-3,3'-biphenothiazine] (4aa)**

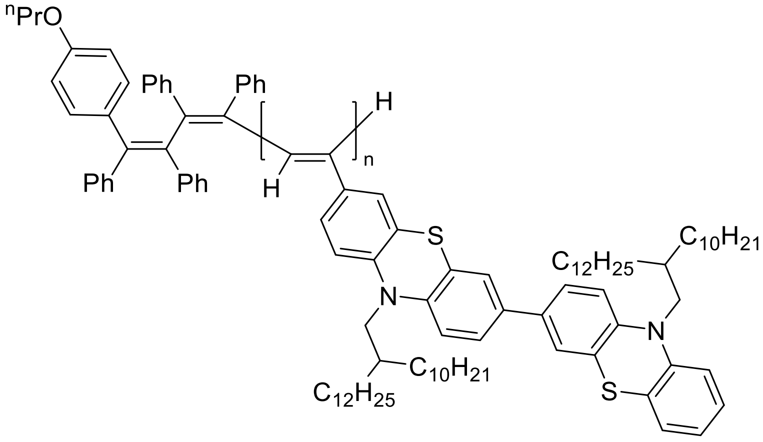

$(C_{74}H_{112}N_2S_2)_n$
[1093.84] Monomer

The synthesis was performed according to **GP8** to give (32 mg, 72%) of the product **4aa** as an orange solid.

**IR $\tilde{\nu}$ [cm$^{-1}$]:** 2955 (w), 2920 (s), 2851 (s), 1600 (w), 1578 (w), 1456 (m), 1446 (w), 1377 (w), 1335 (w), 1252 (m), 1215 (w), 1159 (w), 1153 (w), 1132 (w), 1107 (w), 1063 (w), 1040 (w), 874 (w), 849 (w), 808 (s), 745 (s), 719 (w), 696 (w), 665 (w).

**GPC-RI-LS (THF):** $M_w$ = 108624 Da, $M_n$ = 90134 Da, $M_w/M_n$ = 1.21.

**Poly[10,10',10''-tris(2-decyltetradecyl)-7-ethynyl-10*H*,10'*H*,10''*H*-[3,3',7',3''] terphenothiazine] (4ab)**

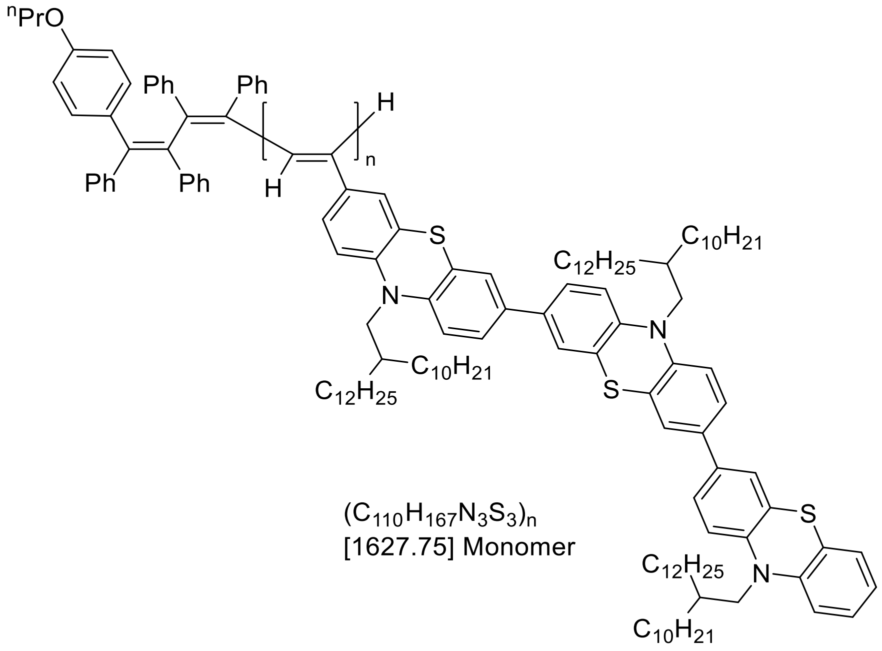


The synthesis was performed according to **GP8** to give (50 mg, 68%) of the product **4ab** as an orange solid.

**IR $\tilde{\nu}$ [cm$^{-1}$]:** 2956 (w), 2920 (s), 2851 (s), 1603 (w), 1578 (w), 1456 (s), 1414 (w), 1377 (w), 1333 (w), 1254 (m), 1213 (w), 1153 (w), 1132 (w), 1105 (w), 1063 (w), 1038 (w), 874 (w), 808 (s), 721 (s), 712 (w), 696 (w), 667 (w), 619 (w).

**GPC-RI-LS (THF):** $M_w$ = 58817 Da, $M_n$ = 14708 Da, $M_w/M_n$ = 4.00

**Poly[10-(2-decyltetradecyl)-7-ethynyl-10*H*-3,10'-biphenothiazine] (4j)**

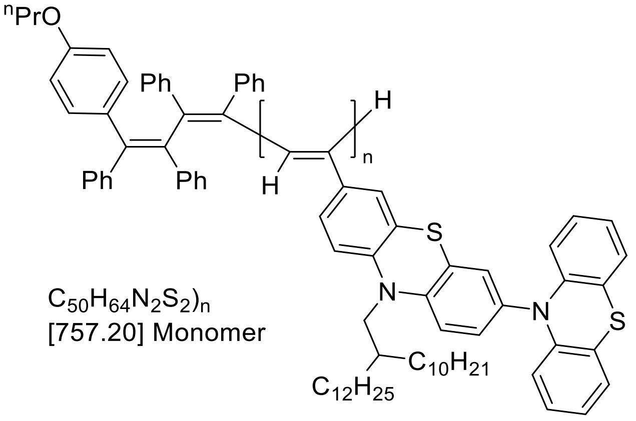


$(C_{50}H_{64}N_2S_2)_n$
[757.20] Monomer

The synthesis was performed according to **GP8** to give (45 mg, 75%) of the product **4j** as an orange solid.

**IR $\tilde{\nu}$ [cm$^{-1}$]**: 2990 (w), 2957 (w), 2922 (s), 2899 (w), 2853 (m), 1495 (w), 1460 (s), 1443 (m), 1402 (w), 1377 (w), 1335 (w), 1306 (m), 1283 (w), 1240 (m), 1128 (w), 1099 (w), 1078 (w), 1045 (s), 818 (w), 743 (s), 719 (w).

**GPC-RI-LS (THF):** $M_w$ = 17363 Da, $M_n$ = 16611 Da, $M_w/M_n$ = 1.04.

**Poly[10-(2-decyltetradecyl)-7-ethinyl-*N*,*N*-diphenyl-10*H*-phenothiazine-3-amine] (4k)**

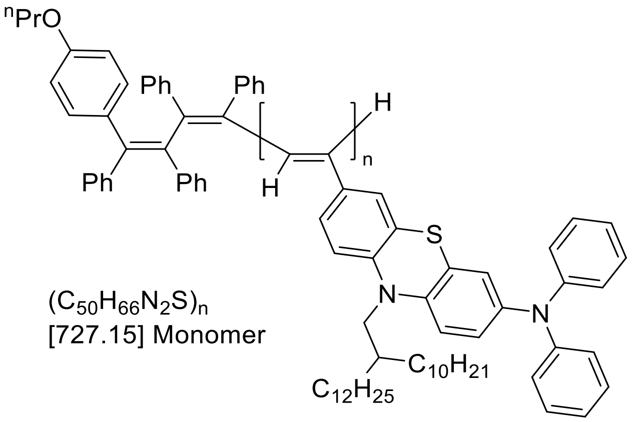


$(C_{50}H_{66}N_2S)_n$
[727.15] Monomer

The synthesis was performed according to **GP8** to give (38 mg, 84%) of the product **4k** as an orange solid.

**IR $\tilde{\nu}$ [cm$^{-1}$]**: 2922 (s), 2851 (s), 2185 (w), 2162 (w), 2154 (w), 2016 (w), 1588 (m), 1493 (s), 1468 (s), 1460 (s), 1408 (w), 1377 (w), 1329 (w), 1310 (w), 1273 (m), 1258 (m), 1219 (w), 1175 (w), 1153 (w), 1103 (w), 1030 (w), 955 (w), 870 (w), 812 (m), 779 (w), 750 (s), 719 (w), 694 (s), 633 (w).

**GPC-RI-LS (THF):** $M_w$ = 12262 Da, $M_n$ = 11194 Da, $M_w/M_n$ = 1.13.

**Poly[10-(2-decyltetradecyl)-7-ethynyl-*N*,*N*-bis(4-methoxyphenyl)-10*H*-phenothiazine-3-amine] (4l)**

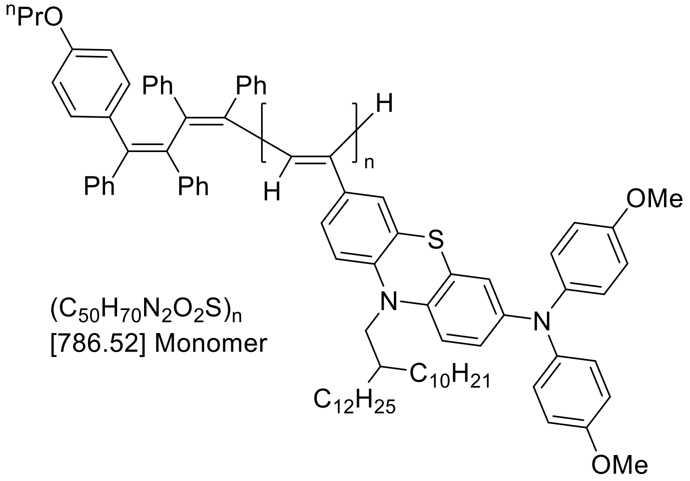


The synthesis was performed according to **GP8** to give (48 mg, 76%) of the product **4l** as an orange solid.

**IR $\tilde{\nu}$ [cm$^{-1}$]**: 2922 (m), 2851 (m), 2162 (w), 2149 (w), 2019 (w), 1971 (w), 1601 (w), 1503 (s), 1460 (s), 1441 (w), 1404 (w), 1319 (w), 1296 (w), 1238 (s), 1279 (w), 1169 (w), 1105 (w), 1040 (m), 959 (w), 870 (w), 826 (m), 814 (w), 806 (w), 781 (w), 760 (w), 750 (w), 719 (w), 689 (w), 637 (w).

**GPC-RI-LS (THF):** $M_w$ = 25907 Da, $M_n$ = 23754 Da, $M_w/M_n$ = 1.09.

**Poly[3-(9*H*-carbazol-9-yl)-10-(2-decyltetradecyl)-7-ethynyl-10*H*-phenothiazine] (4m)**

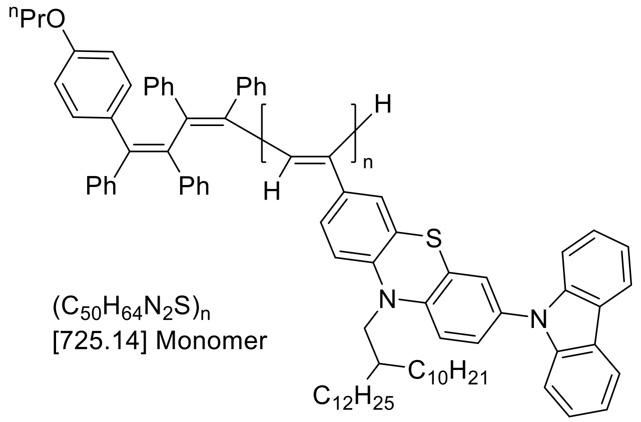

The synthesis was performed according to **GP8** to give (47 mg, 85%) of the product **4m** as an orange solid.

**IR $\tilde{\nu}$ [cm$^{-1}$]**: 2986 (w), 2955 (w), 2920 (s), 2851 (m), 1599 (w), 1503 (m), 1452 (s), 1406 (w), 1377 (w), 1333 (m), 1312 (m), 1229 (s), 1179 (w), 1148 (w), 1099 (w), 1074 (w), 1045 (w), 1015 (w), 1003 (w), 970 (w), 918 (w), 880 (w), 849 (w), 814 (m), 785 (m), 772 (w), 746 (s), 723 (s), 692 (w), 671 (w), 644 (w).

**GPC-RI-LS (THF):** $M_w$ = 26016 Da, $M_n$ = 24514 Da, $M_w/M_n$ = 1.06.

**Poly[10-(2-Decyltetradecyl)-7-ethynyl-N,N-bis(4-nitrophenyl)-10*H*-phenothiazine-3-amine] (4n)**

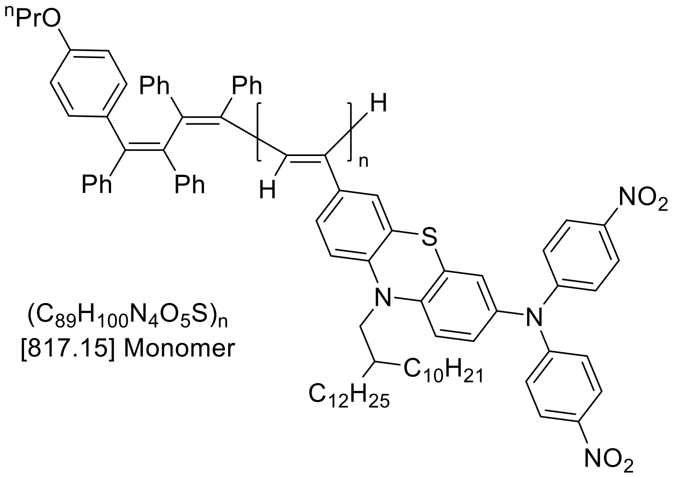


$(C_{89}H_{100}N_4O_5S)_n$
[817.15] Monomer

The synthesis was performed according to **GP8** to give (47 mg, 85%) of the product **4n** as a red solid.

**IR $\tilde{\nu}$ [cm$^{-1}$]**: 2922 (m), 2851 (m), 2359 (w), 1598 (m), 1582 (s), 1514 (w), 1495 (m), 1460 (s), 1423 (w), 1404 (w), 1377 (w), 1337 (m), 132 (s), 1281 (s), 1179 (w), 1146 (w), 1109 (s), 1005 (w), 953 (w), 922 (w), 887 (w), 876 (w), 843 (m), 816 (w), 775 (w), 750 (m), 723 (w), 710 (w), 691 (w), 679 (w).

**GPC-RI-LS (THF):** $M_w$ = 92329 Da, $M_n$ = 70191 Da, $M_w/M_n$ = 1.31.

**Poly[4-(10-(2-decyltetradecyl)-7-ethynyl-10*H*-phenothiazine-3-yl)morpholine] (4o)**

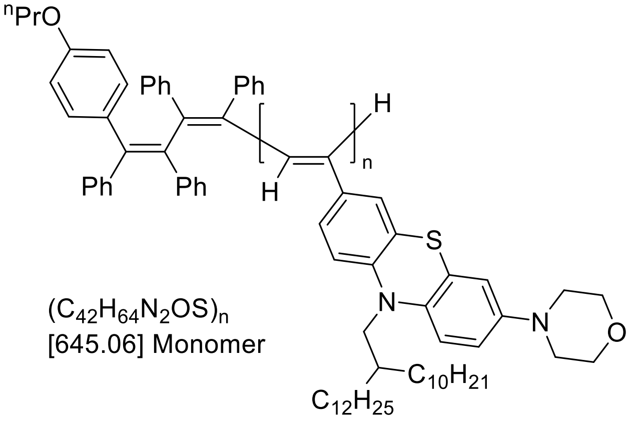


$(C_{42}H_{64}N_2OS)_n$
[645.06] Monomer

The synthesis was performed according to **GP8** to give (52 mg, 67%) of the product **4o** as an orange solid.

**IR $\tilde{\nu}$ [cm$^{-1}$]**: 2955 (w), 2922 (s), 2885 (w), 2851 (w), 2359 (m), 2160 (w), 2021 (w), 1605 (w), 1501 (w), 1474 (s), 1449 (w), 1406 (w), 1395 (w), 1379 (w), 1329 (w), 1302 (w), 1252 (m), 1227 (m), 1123 (m), 1070 (w), 1057 (w), 1047 (w), 1018 (w), 1003 (w), 953 (s), 791 (w).

**GPC-RI-LS (THF):** $M_w$ = 34943 Da, $M_n$ = 32742 Da, $M_w/M_n$ = 1.06.

**Poly[(*E*)-10-(2-Decyltetradecyl)-3-ethynyl-7-styryl-10*H*-phenothiazine] (4p)**

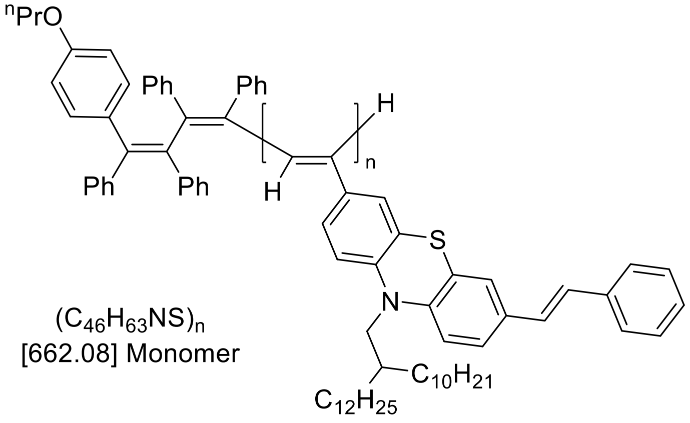


$(C_{46}H_{63}NS)_n$
[662.08] Monomer

The synthesis was performed according to **GP8** to give (40 mg, 75%) of the product **4p** as an orange solid.

**IR $\tilde{\nu}$ [cm$^{-1}$]**: 3057 (w), 3021 (w), 2957 (w), 2920 (s), 2851 (m), 2776 (w), 2650 (w), 1597 (w), 1580 (w), 1545 (w), 1495 (w), 1462 (s), 1404 (w), 1377 (w), 1337 (m), 1302 (w), 1260 (s), 1221 (w), 1194 (w), 1155 (w), 1146 (w), 1103 (s), 1022 (s), 978 (w), 957 (m), 918 (w), 880 (w), 804 (s), 748 (w), 721 (w), 689 (s), 667 (w), 638 (w).

**GPC-RI-LS (THF):** $M_w$ = 68234 Da, $M_n$ = 66191 Da, $M_w/M_n$ = 1.03.

**Poly[(*E*)-10-(2-Decyltetradecyl)-3-ethynyl-7-(4-methoxystyryl)-10*H*-phenothiazine] (4q)**

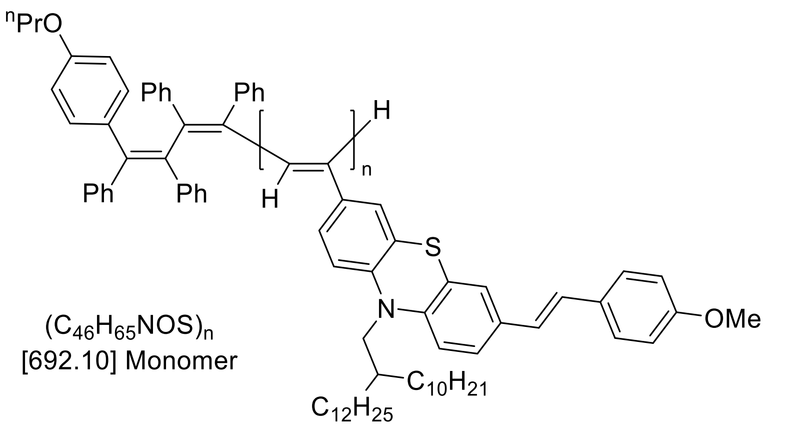


The synthesis was performed according to **GP8** to give (37 mg, 67%) of the product **4q** as an orange solid.

**IR $\tilde{\nu}$ [cm$^{-1}$]**: 2953 (w), 2920 (s), 2851 (m), 2355 (w), 2340 (w), 1607 (m), 1510 (s), 1462 (s), 1402 (w), 1377 (w), 1335 (w), 1296 (w), 1248 (s), 1211 (w), 1173 (m), 1107 (w), 1038 (m), 957 (m), 883 (w), 849 (w), 820 (s), 772 (w), 721 (w).

**GPC-RI-LS (THF):** $M_w$ = 29983 Da, $M_n$ = 15571 Da, $M_w/M_n$ = 1.92.

**Poly[(*E*)-4-(2-(10-(2-Decyltetradecyl)-7-ethynyl-10*H*-phenothiazine-3-yl)vinyl)benzonitrile] (4r)**

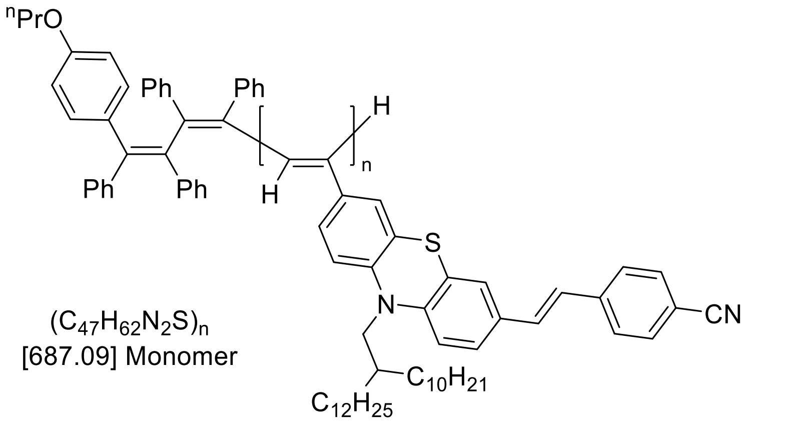


The synthesis was performed according to **GP8** to give (46 mg, 83%) of the product **4r** as an orange solid.

**IR $\tilde{\nu}$ [cm$^{-1}$]**: 3022 (w), 2920 (s), 2851 (m), 2729 (w), 2224 (m), 1630 (w), 1597 (m), 1578 (m), 1549 (w), 1506 (m), 1462 (s), 1402 (m), 1377 (w), 1335 (m), 1296 (w), 1250 (m), 1215 (w), 1196 (m), 1173 (m), 1105 (w), 1042 (w), 1018 (w), 961 (m), 883 (w), 860 (w), 820 (s), 802 (w), 721 (m), 640 (w).

**GPC-RI-LS (THF):** $M_w$ = 39244 Da, $M_n$ = 20143 Da, $M_w/M_n$ = 1.95.

### Poly[(*E*)-10-(2-Decyltetradecyl)-3-ethynyl-7-(4-nitrostyryl)-10*H*-phenothiazine] (4s)

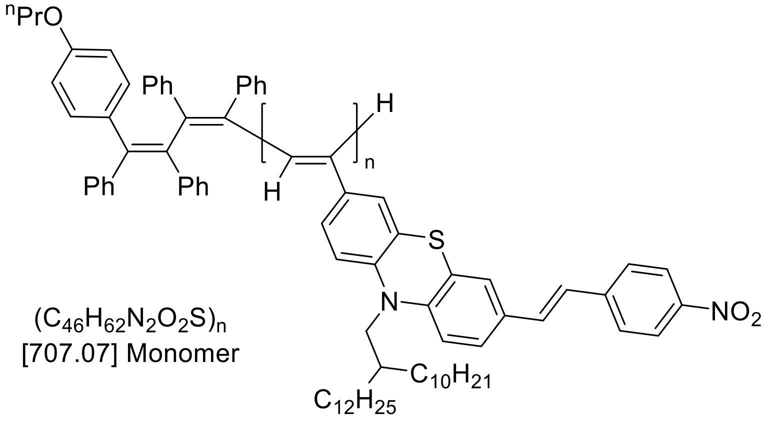


$(C_{46}H_{62}N_2O_2S)_n$
[707.07] Monomer

The synthesis was performed according to **GP8** to give (44 mg, 78%) of the product **4s** as a red solid.

**IR $\tilde{\nu}$ [cm$^{-1}$]**: 2922 (s), 2851 (m), 2671 (w), 1628 (w), 1591 (m), 1578 (m), 1514 (m), 1499 (w), 1462 (s), 1400 (w), 1337 (s), 1248 (m), 1194 (m), 1165 (w), 1107 (m), 1013 (w), 962 (w), 953 (w), 887 (w), 862 (m), 829 (w), 810 (w), 748 (w), 723 (w), 689 (w), 664 (w).

**GPC-RI-LS (THF):** $M_w$ = 1875900 Da, $M_n$ = 11835010 Da, $M_w/M_n$ = 15.8.

### Poly[(*E*)-10-(2-Decyltetradecyl)-3-ethynyl-7-(4-(trifluoromethyl)styryl)-10*H*-phenothiazine] (4t)

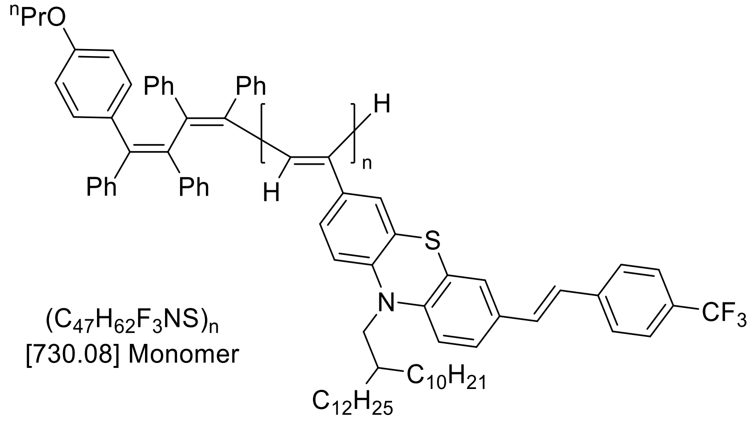


$(C_{47}H_{62}F_3NS)_n$
[730.08] Monomer

The synthesis was performed according to **GP8** to give (42 mg, 72%) of the product **4t** as an orange solid.

**IR $\tilde{\nu}$ [cm$^{-1}$]**: 2922 (s), 2851 (m), 1612 (w), 1601 (w), 1580 (w), 1495 (w), 1464 (s), 1402 (w), 1377 (w), 1321 (s), 1260 (w), 1250 (w), 1221 (w), 1184 (w), 1165 (m), 1125 (s), 1109 (m), 1067 (s), 1015 (w), 961 (w), 949 (w), 883 (w), 860 (w), 822 (m), 804 (w), 766 (w), 721 (w), 710 (w), 648 (w).

**GPC-RI-LS (THF):** $M_w$ = 93236 Da, $M_n$ = 89985 Da, $M_w/M_n$ = 1.03.

**Poly[(*E*)-4-(2-(10-(2-Decyltetradecyl)-7-ethynyl-10*H*-phenothiazin-3-yl)vinyl)-*N*,*N*-diphenylaniline (4u)**

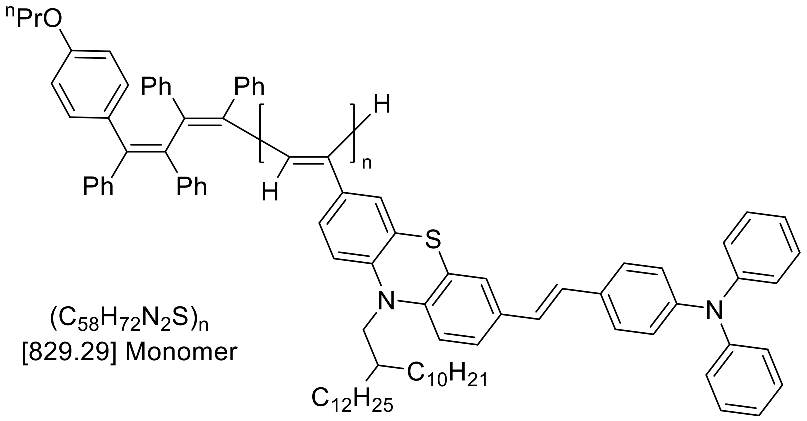


$(C_{58}H_{72}N_2S)_n$
[829.29] Monomer

The synthesis was performed according to **GP8** to give (45 mg, 68%) of the product **4u** as an orange solid.

**IR $\tilde{\nu}$ [cm$^{-1}$]**: 2920 (s), 2851 (s), 1589 (m), 1506 (s), 1493 (s), 1464 (s), 1237 (m), 1275 (s), 1211 (w), 1153 (w), 881 (w), 818 (m), 803 (w), 750 (s), 721 (w), 694 (s).

**GPC-RI-LS (THF):** $M_w$ =318800 Da, $M_n$ = 245200 Da, $M_w/M_n$ = 1.30.

**Poly[(*E*)-10-(2-decyltetradecyl)-3-(2-(10-(2-decyltetradecyl)-10*H*-phenothiazine-3-yl)vinyl)-7-ethynyl-10*H*-phenothiazine] (4v)**

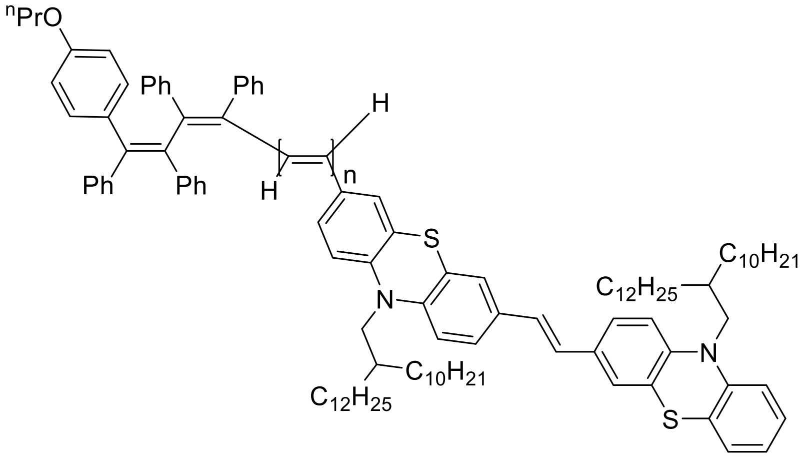


The synthesis was performed according to **GP8** to give (45 mg, 68%) of the product **4v** as an orange solid.

**IR $\tilde{\nu}$ [cm$^{-1}$]:** 3015 (w), 2953 (w), 2920 (s), 2851 (m), 1599 (w), 1576 (w), 1495 (w), 1460 (s), 1445 (w), 1400 (w), 1337 (m), 1287 (w), 1248 (m), 1219 (w), 1161 (w), 1132 (w), 1103 (w), 1038 (w), 955 (w), 926 (w), 874 (w), 841 (w), 812 (m), 745 (m), 721 (w), 694 (w), 679 (w), 667 (w), 629 (w), 617 (w).

$M_w$= 57170 Da, $M_n$= 56840, $M_w/M_n$ = 1.01

## 3.0 UV/Vis absorption and emission spectra of monomers 3 and polymers 4.

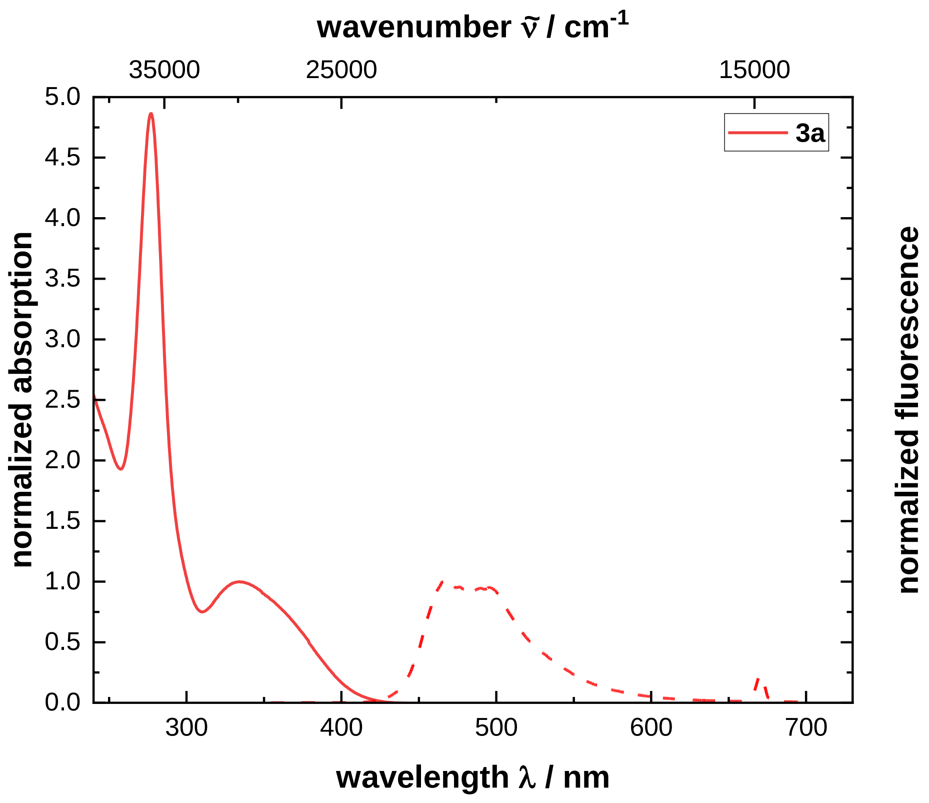


**Figure S1.** UV/Vis and normalized emission spectra (c(**3a**) = $10^{-5}$ M) of monomer **3a** (recorded in $CH_2Cl_2$, $T$ = 293 K).

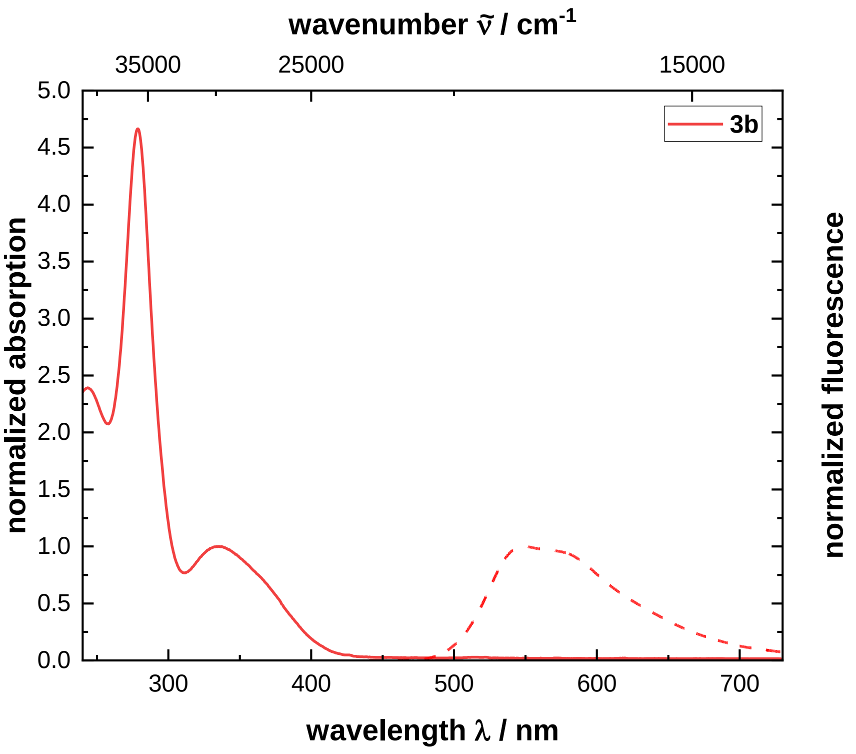


**Figure S2.** UV/Vis and normalized emission spectra (c(**3b**) = $10^{-5}$ M) of monomer **3b** (recorded in $CH_2Cl_2$, $T$ = 293 K).

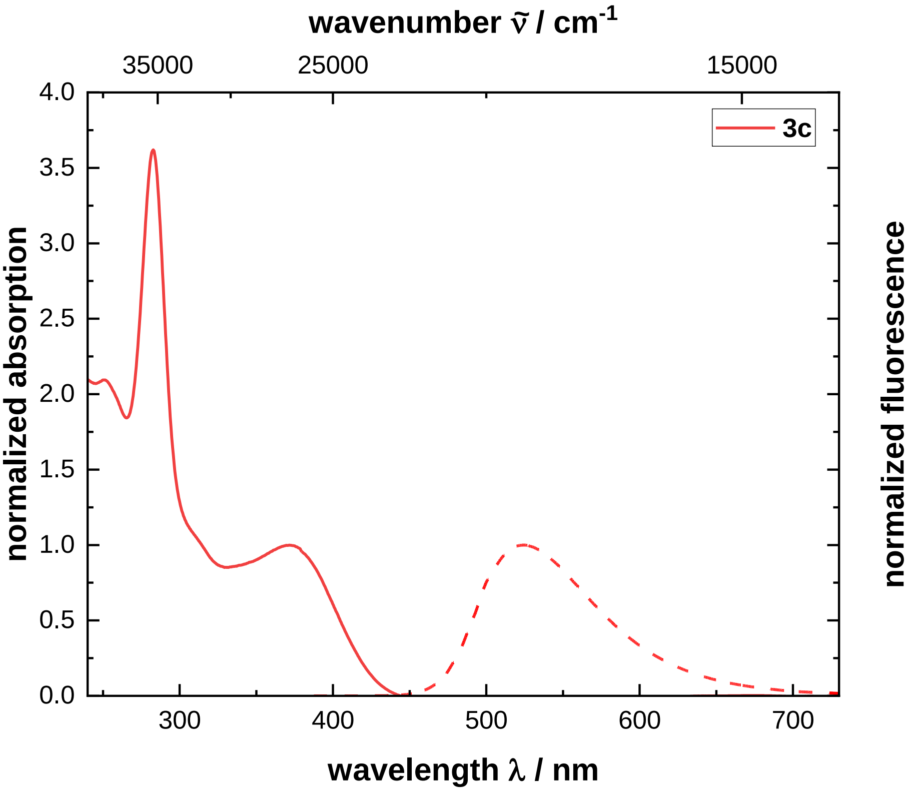


**Figure S3.** UV/Vis and normalized emission spectra (c(**3c**) = $10^{-5}$ M) of monomer **3c** (recorded in $CH_2Cl_2$, $T$ = 293 K).

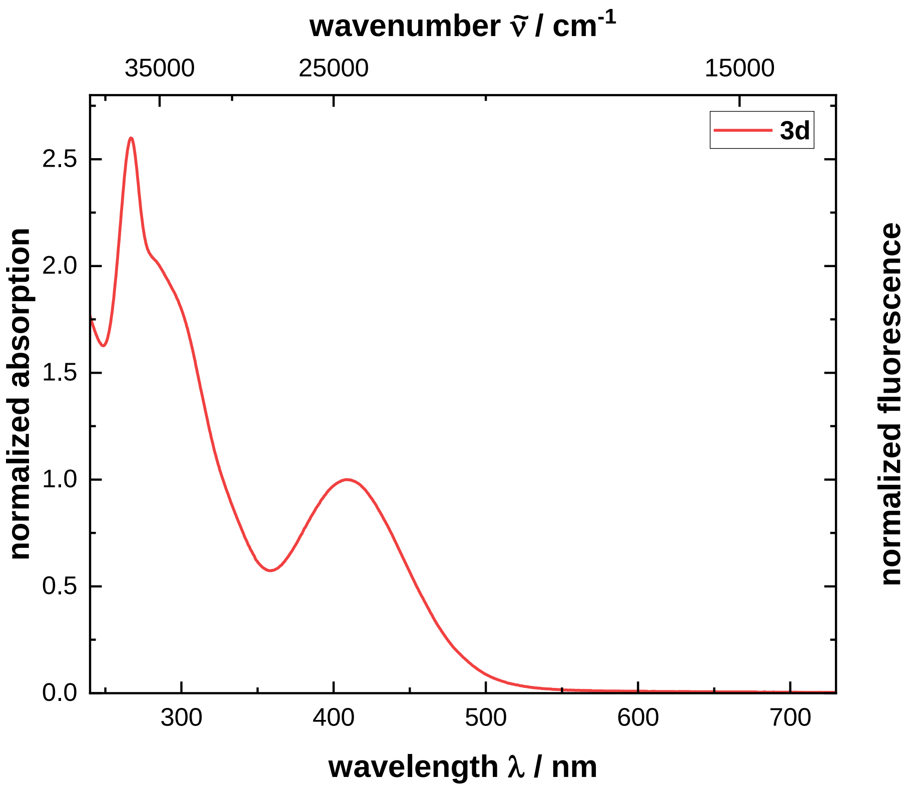


**Figure S4.** UV/Vis and normalized emission spectra (c(**3d**) = $10^{-5}$ M) of monomer **3d** (recorded in $CH_2Cl_2$, $T$ = 293 K).

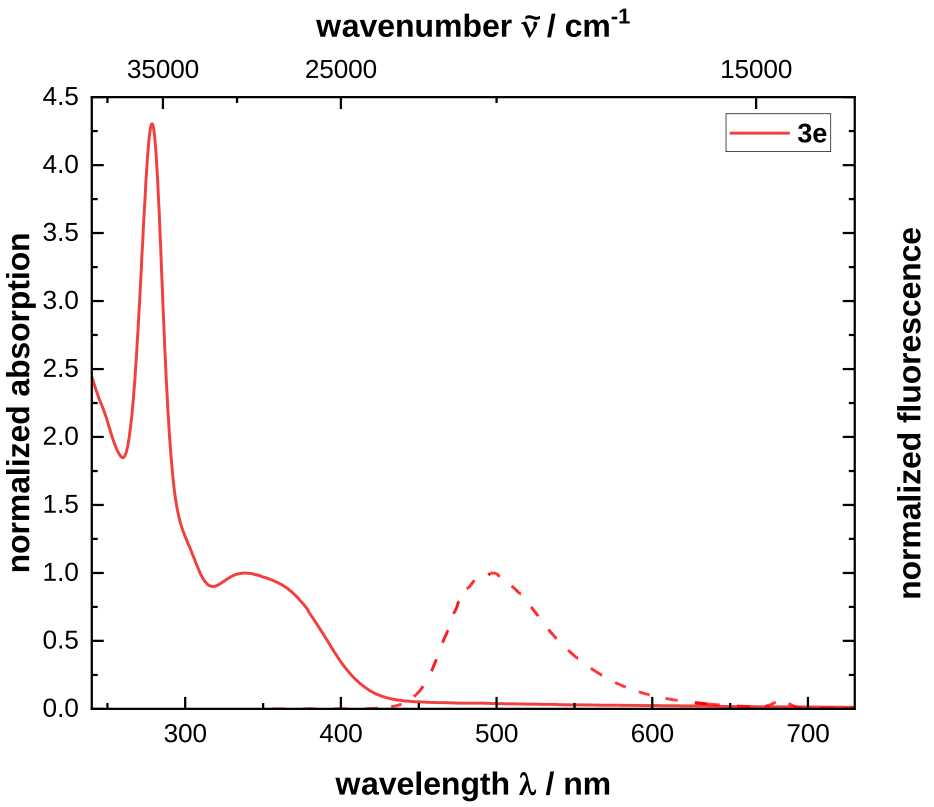


**Figure S5.** UV/Vis and normalized emission spectra (c(**3e**) = $10^{-5}$ M) of monomer **3e** (recorded in $CH_2Cl_2$, $T$ = 293 K).

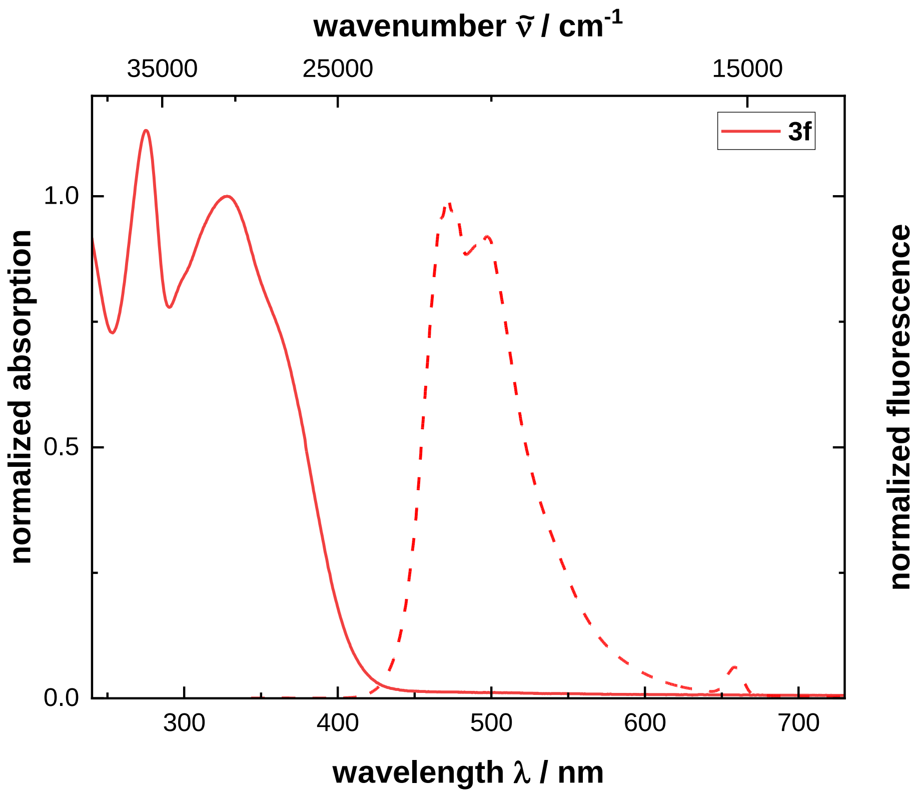


**Figure S6.** UV/Vis and normalized emission spectra (c(**3f**) = $10^{-5}$ M) of monomer **3f** (recorded in $CH_2Cl_2$, $T$ = 293 K).

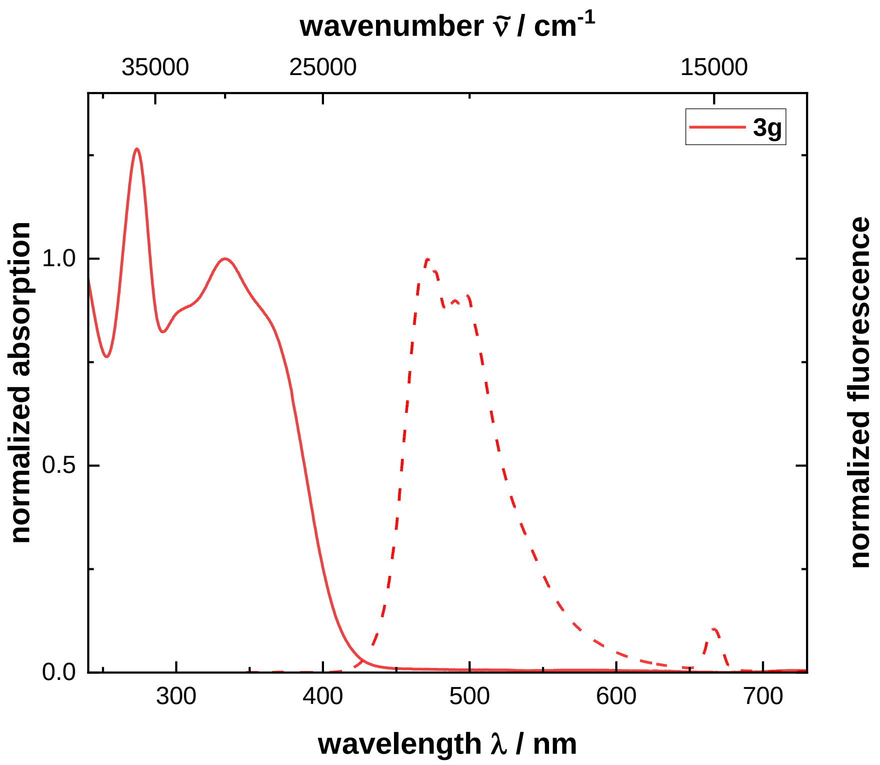


**Figure S7.** UV/Vis and normalized emission spectra (c(**3g**) = $10^{-5}$ M) of monomer **3g** (recorded in $CH_2Cl_2$, $T$ = 293 K).

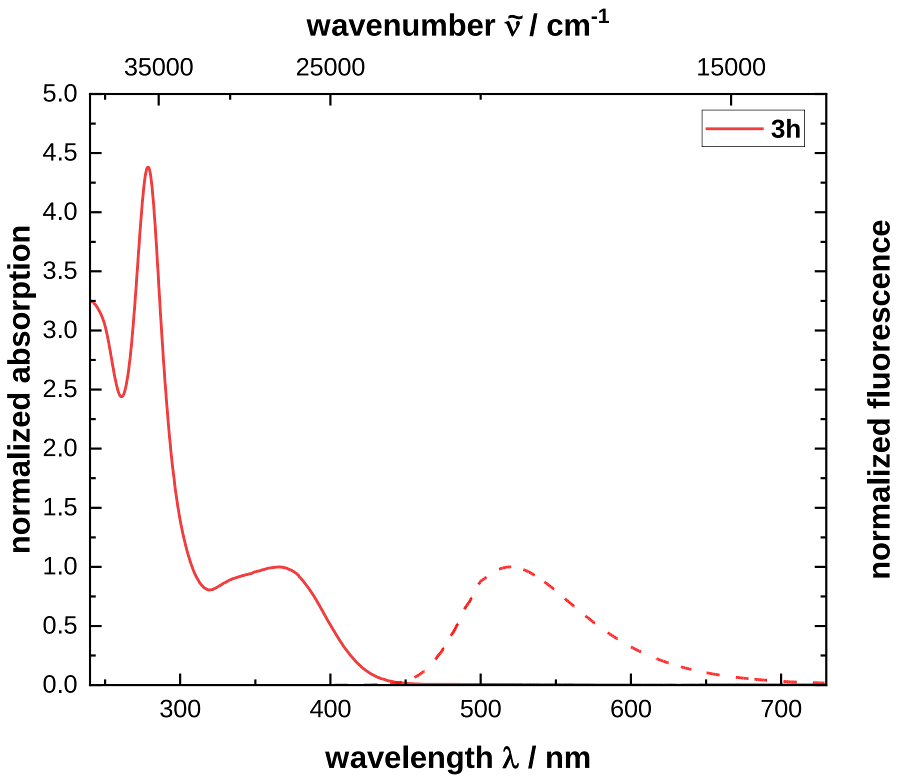


**Figure S8.** UV/Vis and normalized emission spectra (c(**3h**) = $10^{-5}$ M) of monomer **3h** (recorded in $CH_2Cl_2$, $T$ = 293 K).

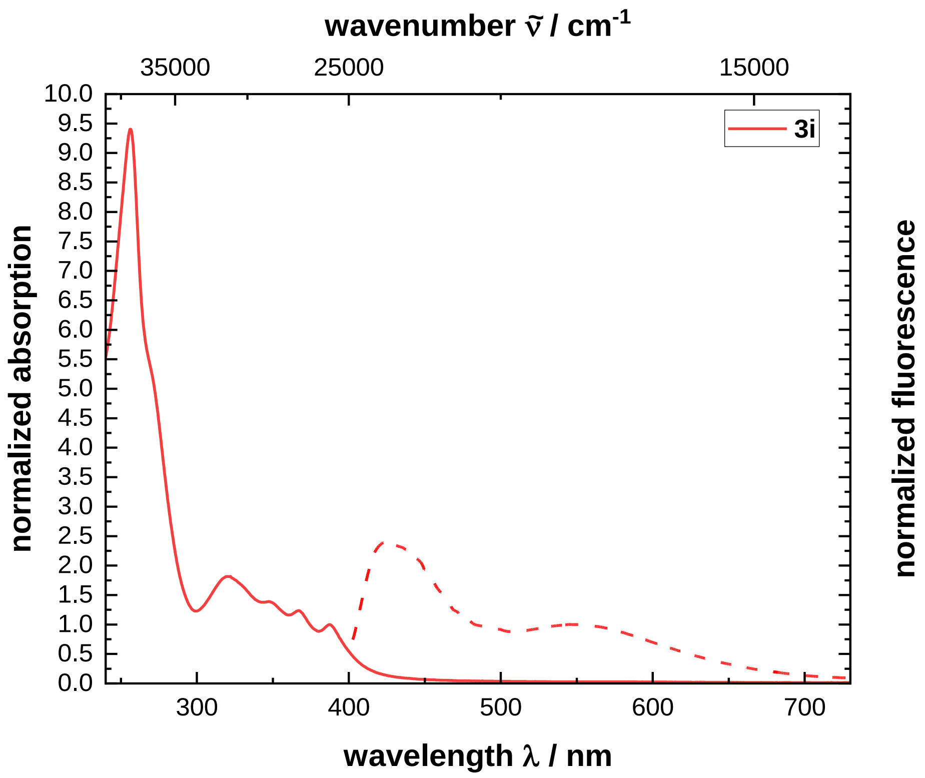


**Figure S9.** UV/Vis and normalized emission spectra (c(**3i**) = $10^{-5}$ M) of monomer **3i** (recorded in $CH_2Cl_2$, $T$ = 293 K).

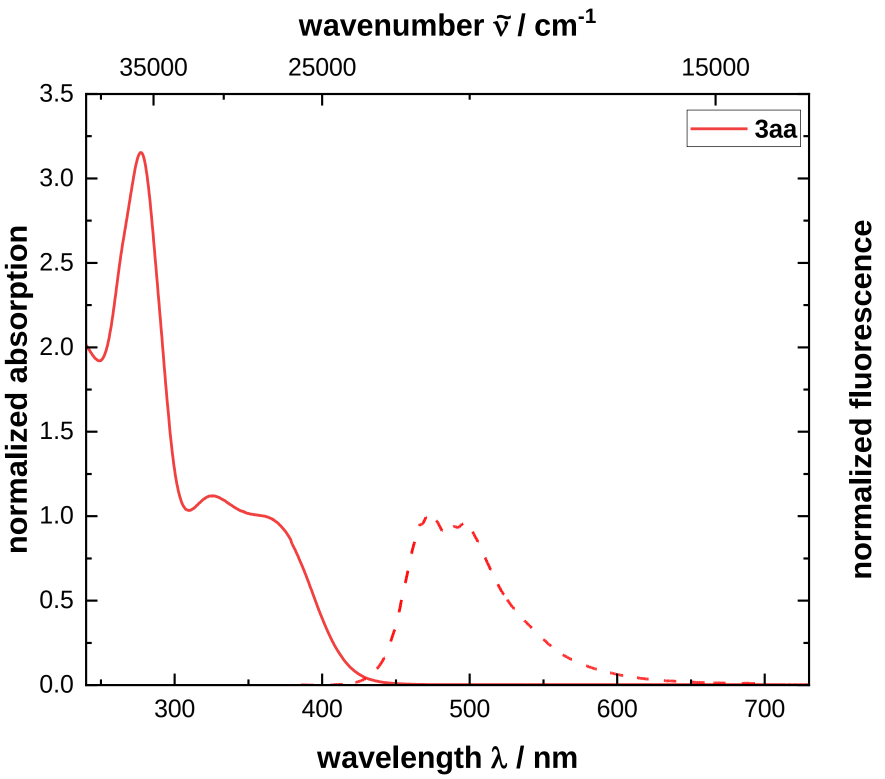


**Figure S10.** UV/Vis and normalized emission spectra (c(**3aa**) = $10^{-5}$ M) of monomer **3aa** (recorded in $CH_2Cl_2$, $T$ = 293 K).

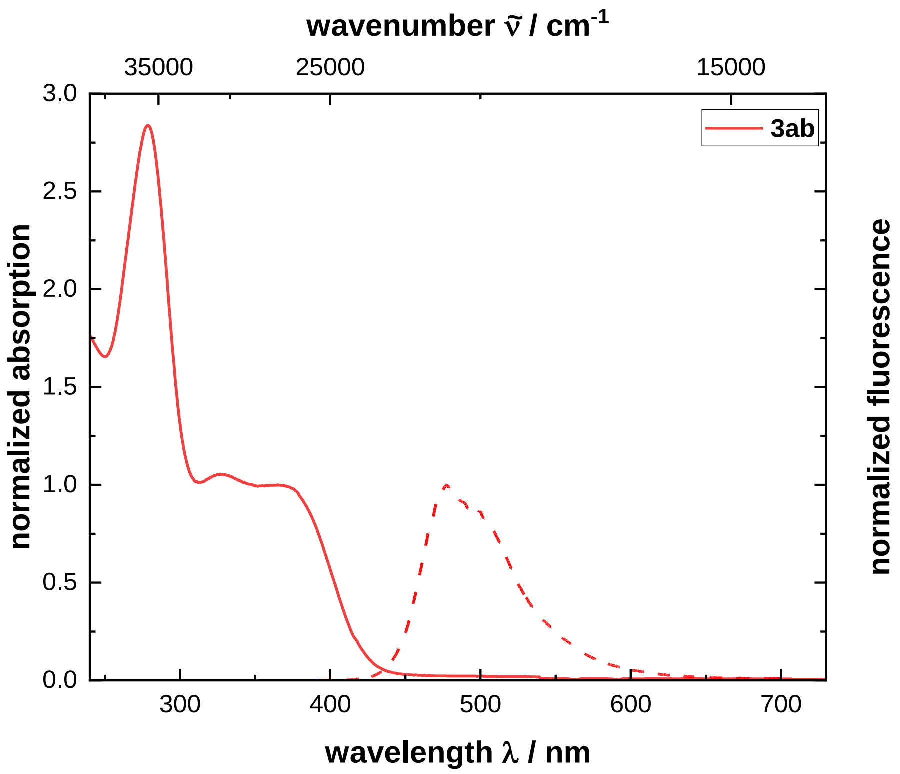


**Figure S11.** UV/Vis and normalized emission spectra (c(**3ab**) = $10^{-5}$ M) of monomer **3ab** (recorded in $CH_2Cl_2$, $T$ = 293 K).

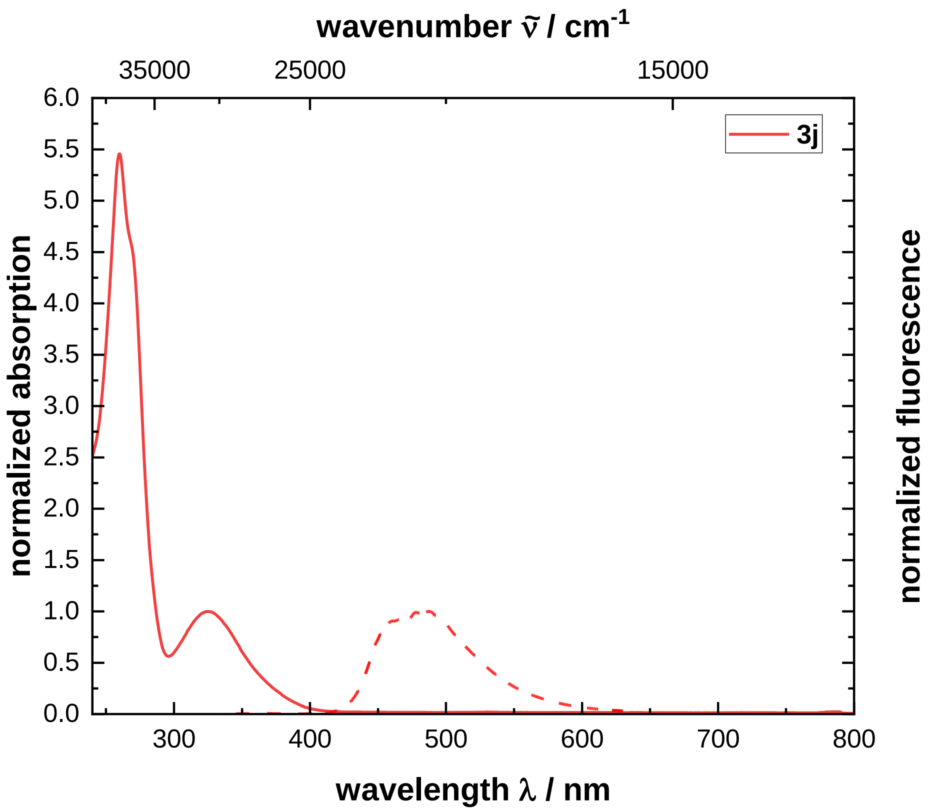


**Figure S12.** UV/Vis and normalized emission spectra (c(**3j**) = $10^{-5}$ M) of monomer **3j** (recorded in $CH_2Cl_2$, $T$ = 293 K).

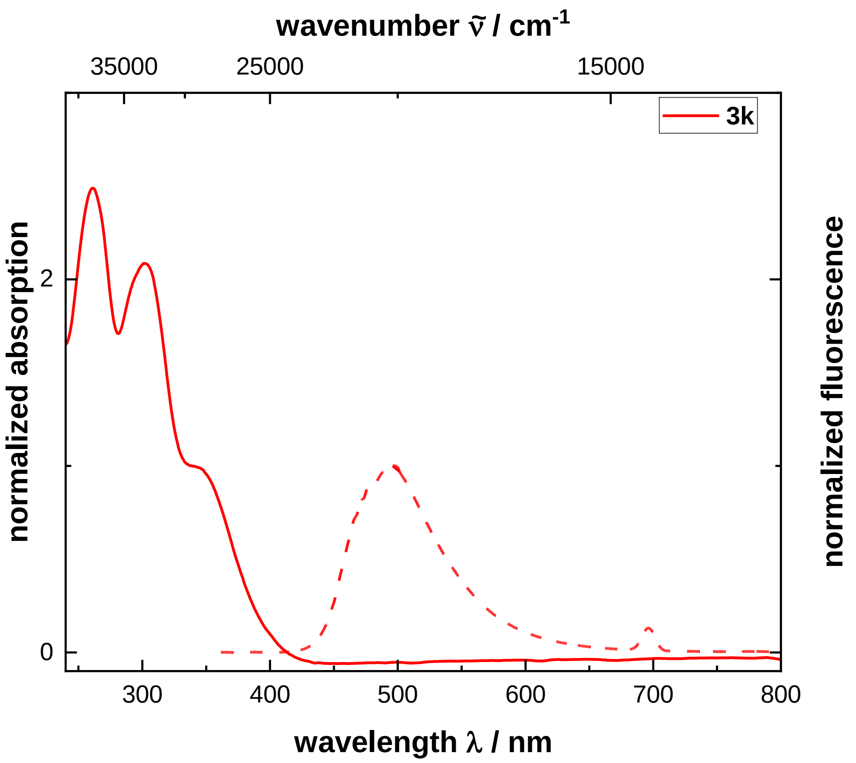


**Figure S13.** UV/Vis and normalized emission spectra (c(**3k**) = $10^{-5}$ M) of monomer **3k** (recorded in $CH_2Cl_2$, $T$ = 293 K).

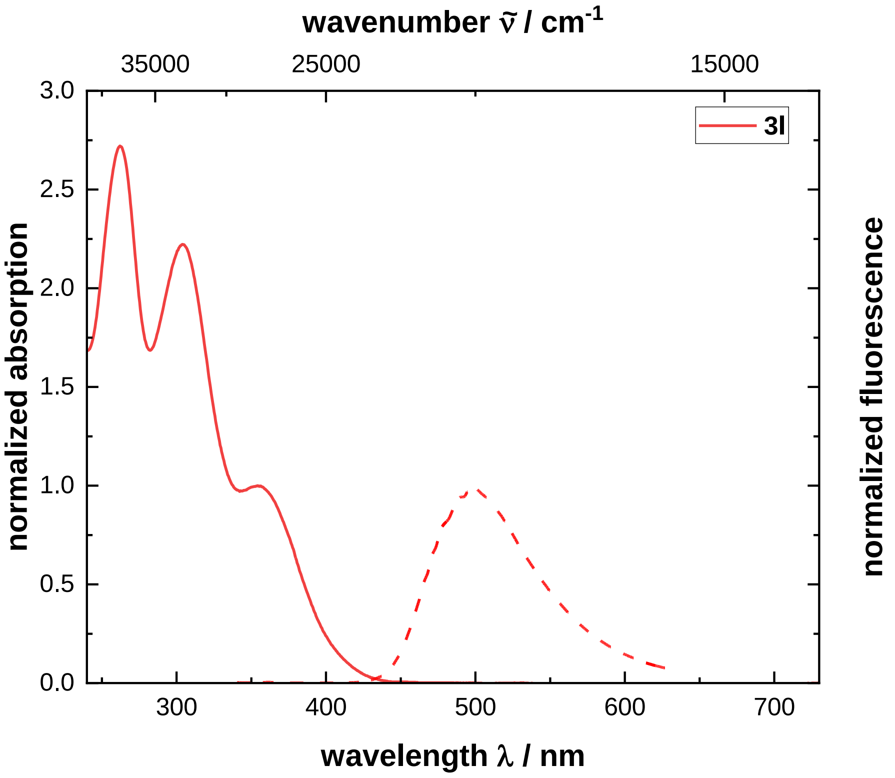


**Figure S14.** UV/Vis and normalized emission spectra (c(**3l**) = $10^{-5}$ M) of monomer **3l** (recorded in $CH_2Cl_2$, $T$ = 293 K).

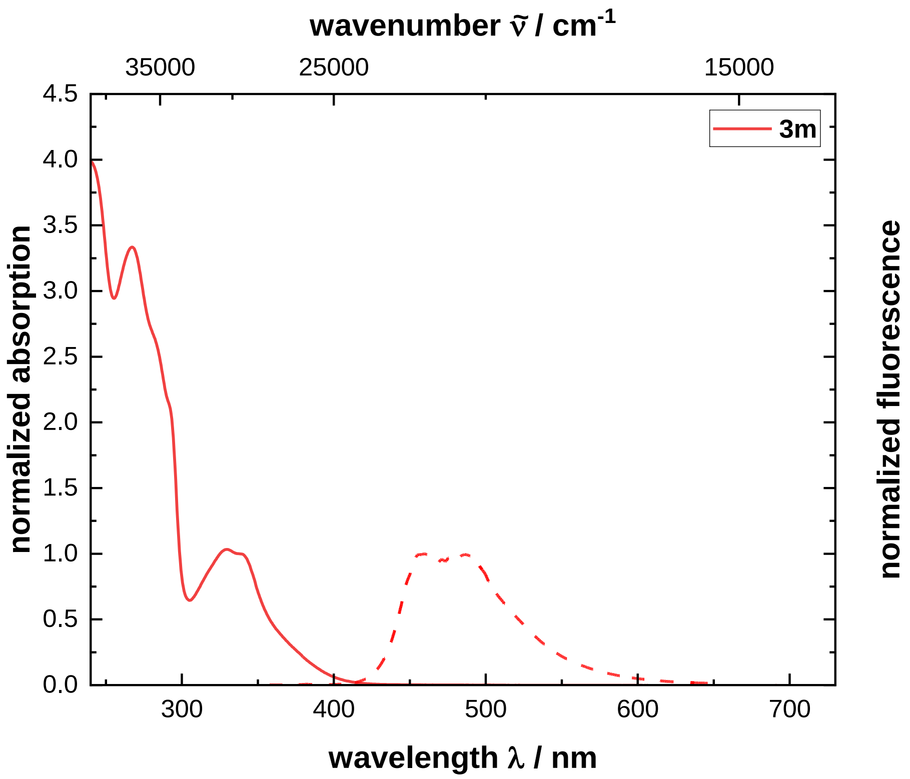


Figure S15. UV/Vis and normalized emission spectra (c(**3m**) = $10^{-5}$ M) of monomer **3m** (recorded in $CH_2Cl_2$, *T* = 293 K).

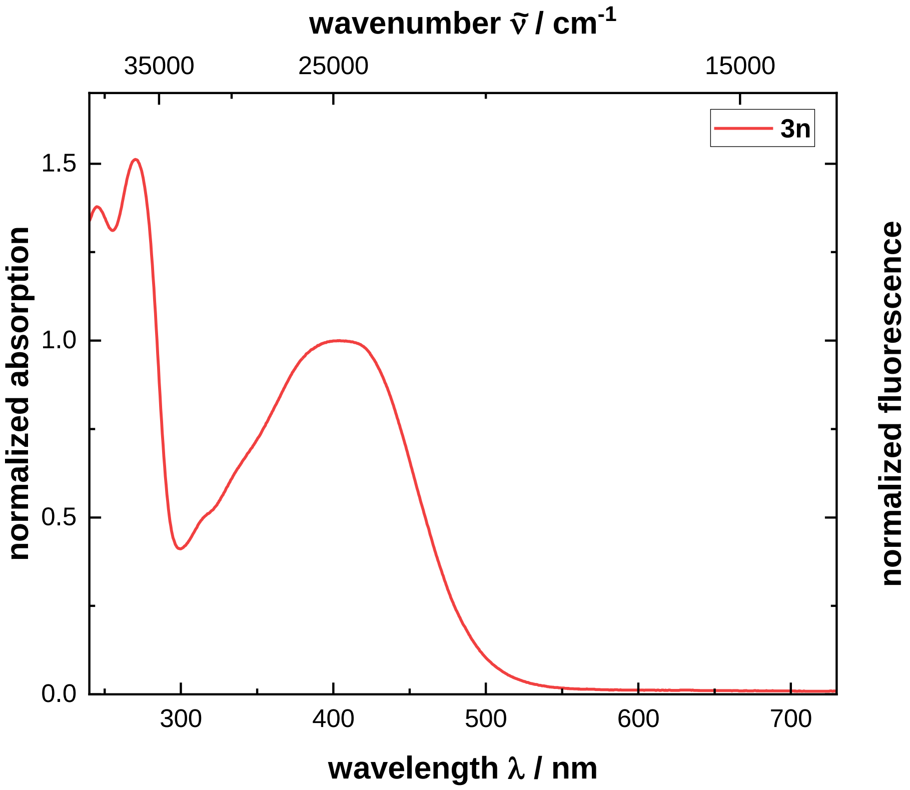


**Figure S16.** UV/Vis and normalized emission spectra (c(**3n**) = $10^{-5}$ M) of monomer **3n** (recorded in $CH_2Cl_2$, *T* = 293 K).

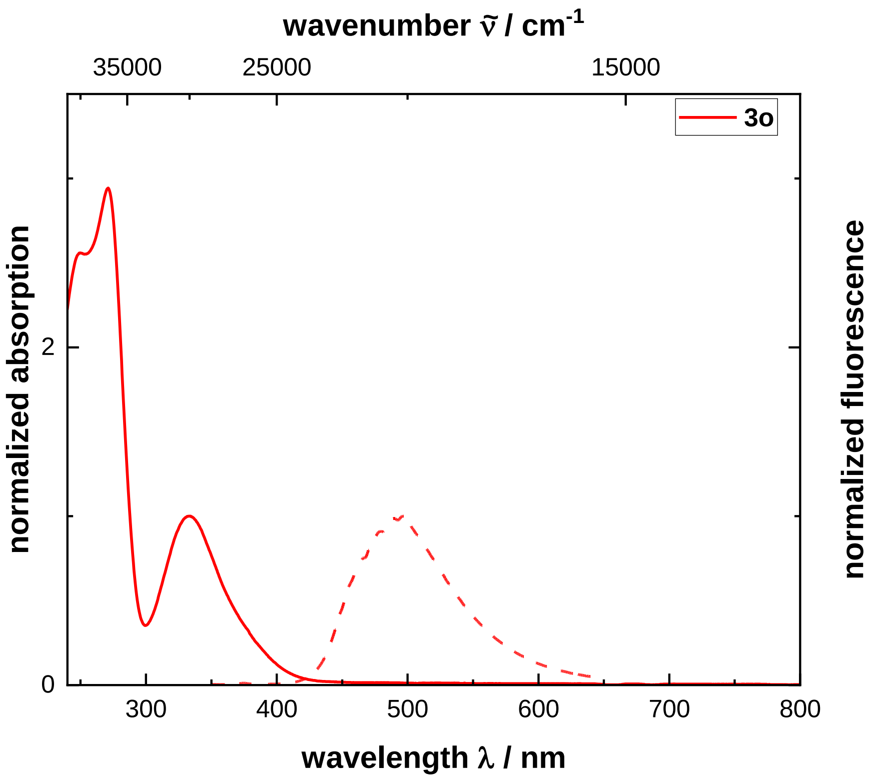


**Figure S17.** UV/Vis and normalized emission spectra (c(**3o**) = $10^{-5}$ M) of monomer **3o** (recorded in $CH_2Cl_2$, $T$ = 293 K).

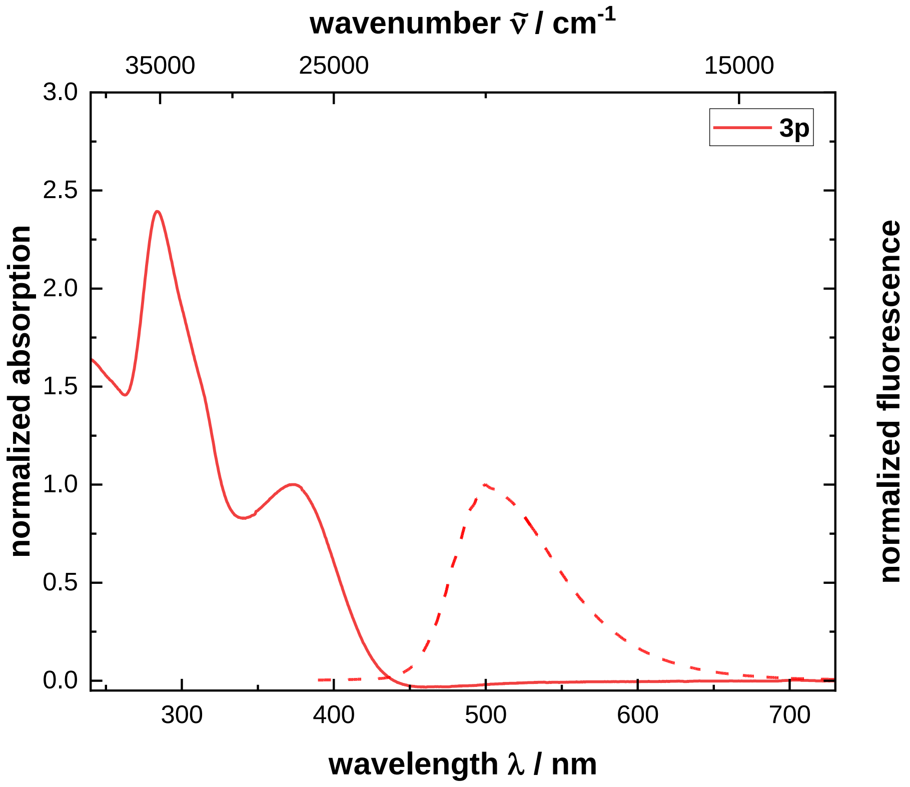


**Figure S18.** UV/Vis and normalized emission spectra (c(**3p**) = $10^{-5}$ M) of monomer **3p** (recorded in $CH_2Cl_2$, $T$ = 293 K).

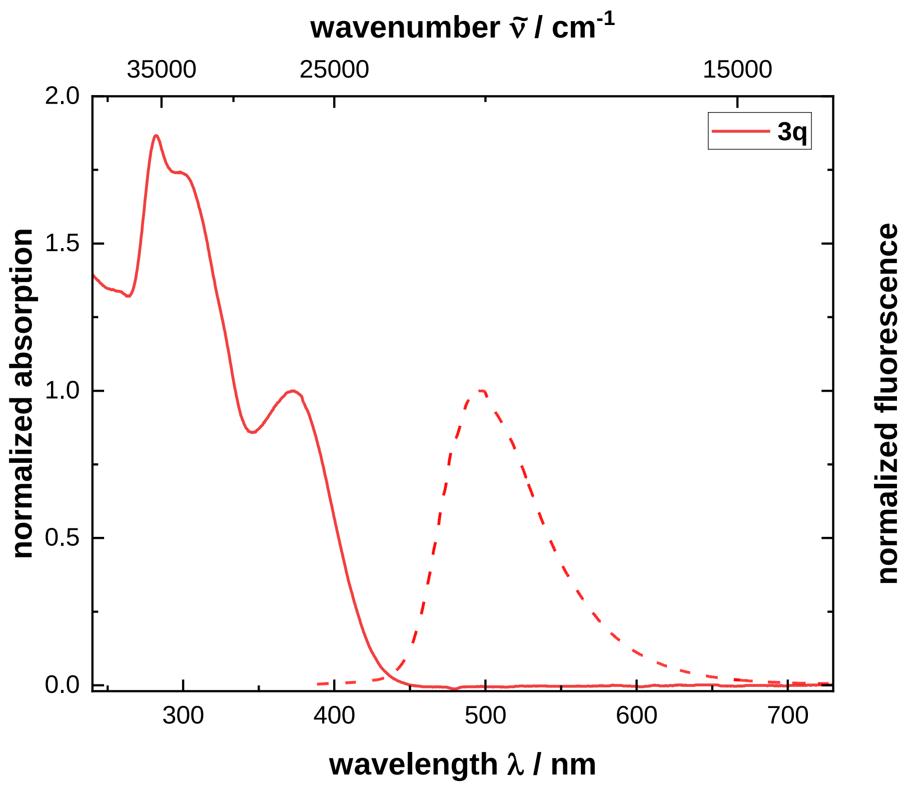


**Figure S19.** UV/Vis and normalized emission spectra (c(**3q**) = $10^{-5}$ M) of monomer **3q** (recorded in $CH_2Cl_2$, *T* = 293 K).

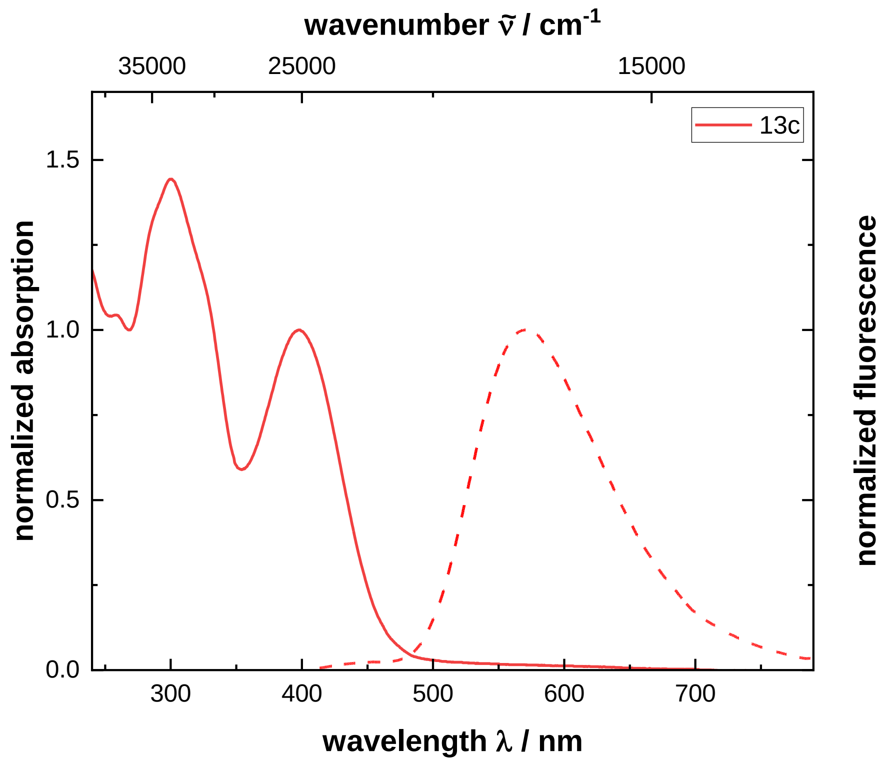


**Figure S20.** UV/Vis and normalized emission spectra (c(**3r**) = $10^{-5}$ M) of monomer **3r** (recorded in $CH_2Cl_2$, $T$ = 293 K).

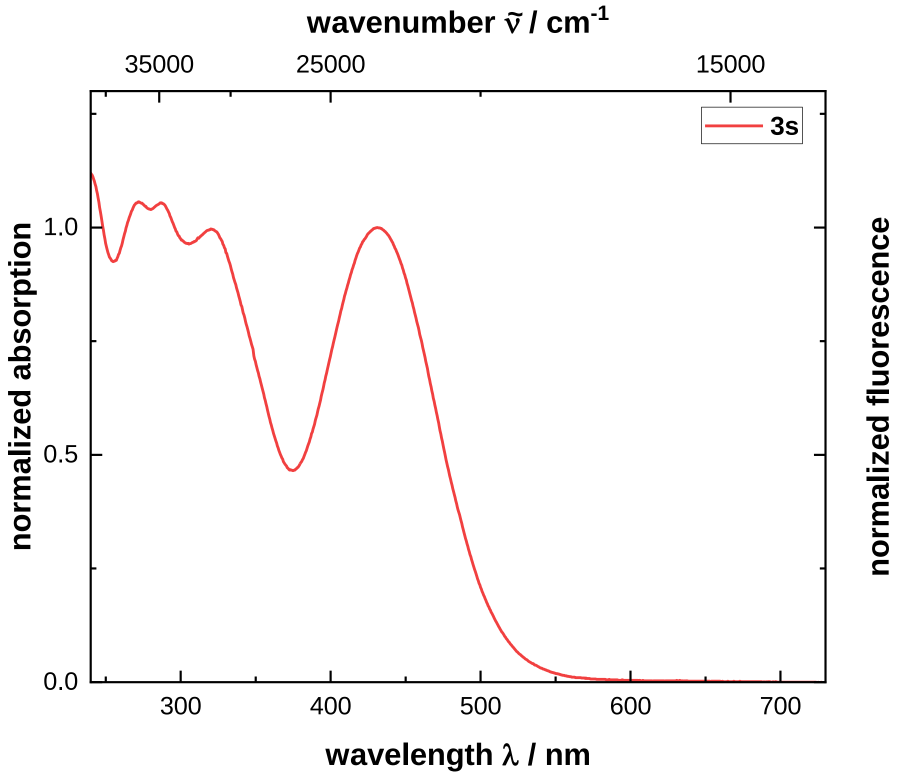


**Figure S21.** UV/Vis and normalized emission spectra (c(**3s**) = $10^{-5}$ M) of monomer **3s** (recorded in $CH_2Cl_2$, $T$ = 293 K).

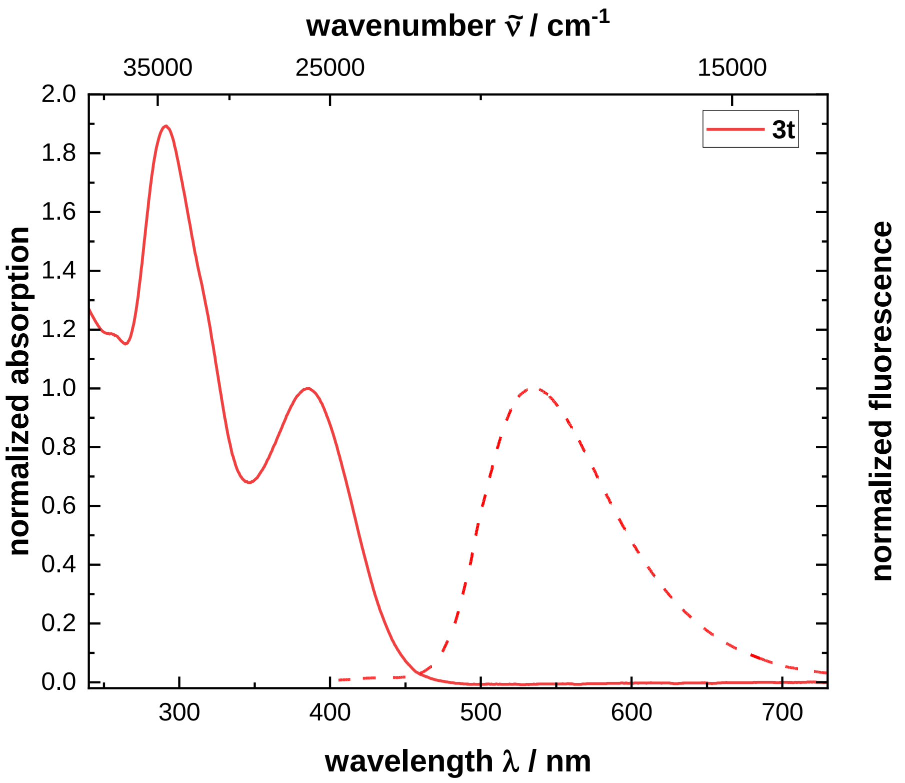


**Figure S22.** UV/Vis and normalized emission spectra (c(**3t**) = $10^{-5}$ M) of monomer **3t** (recorded in $CH_2Cl_2$, $T$ = 293 K).

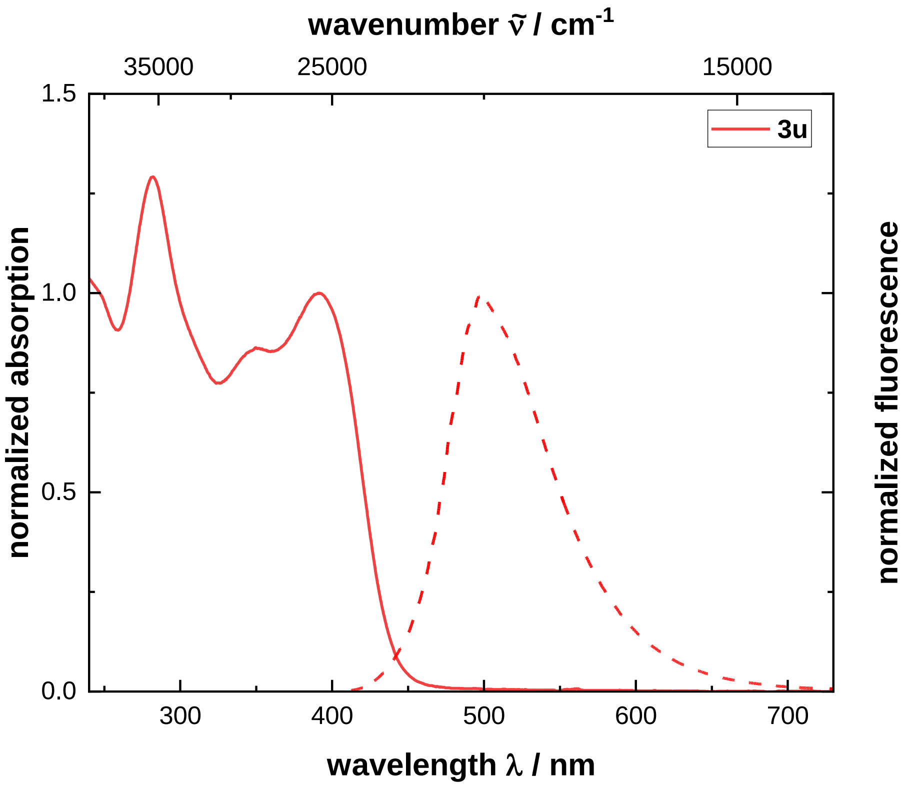


**Figure S23.** UV/Vis and normalized emission spectra (c(**3u**) = $10^{-5}$ M) of monomer **3u** (recorded in $CH_2Cl_2$, $T$ = 293 K).

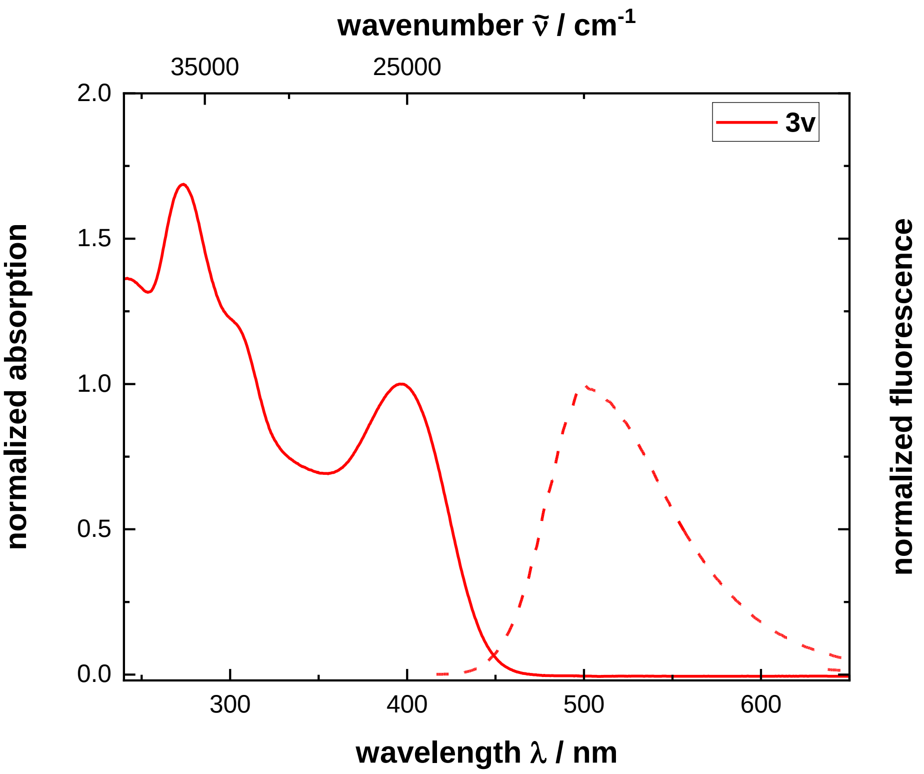


**Figure S24.** UV/Vis and normalized emission spectra (c(**3v**) = $10^{-5}$ M) of monomer **3v** (recorded in $CH_2Cl_2$, *T* = 293 K).

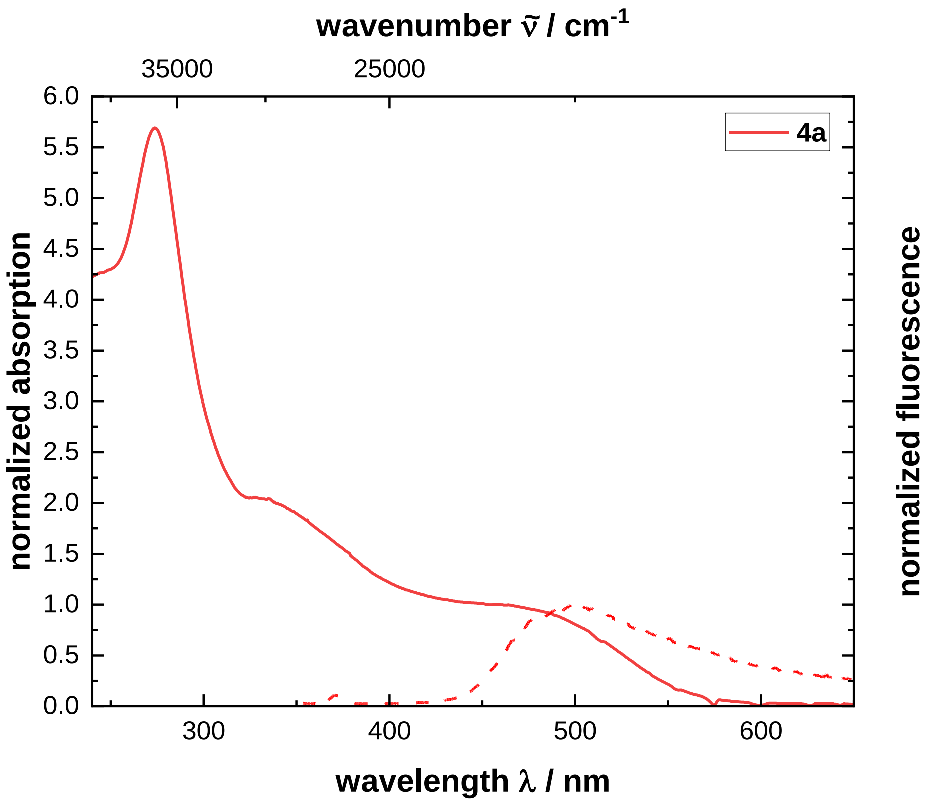


**Figure S25.** UV/Vis and normalized emission spectra (c(**3**) = $10^{-5}$ m) of polymer **4a** (recorded in $CH_2Cl_2$, *T* = 293 K).

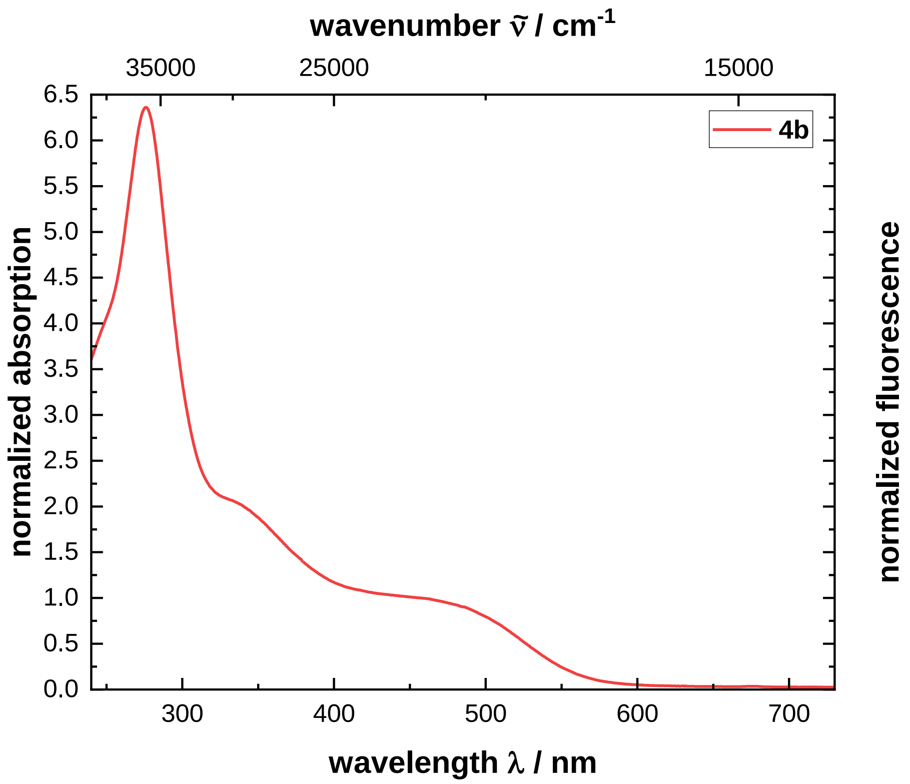


**Figure S26.** UV/Vis and normalized emission spectra (c(**4b**) = 10$^{\square 5}$ m) of polymer **4b** (recorded in $CH_2Cl_2$, *T* = 293 K).

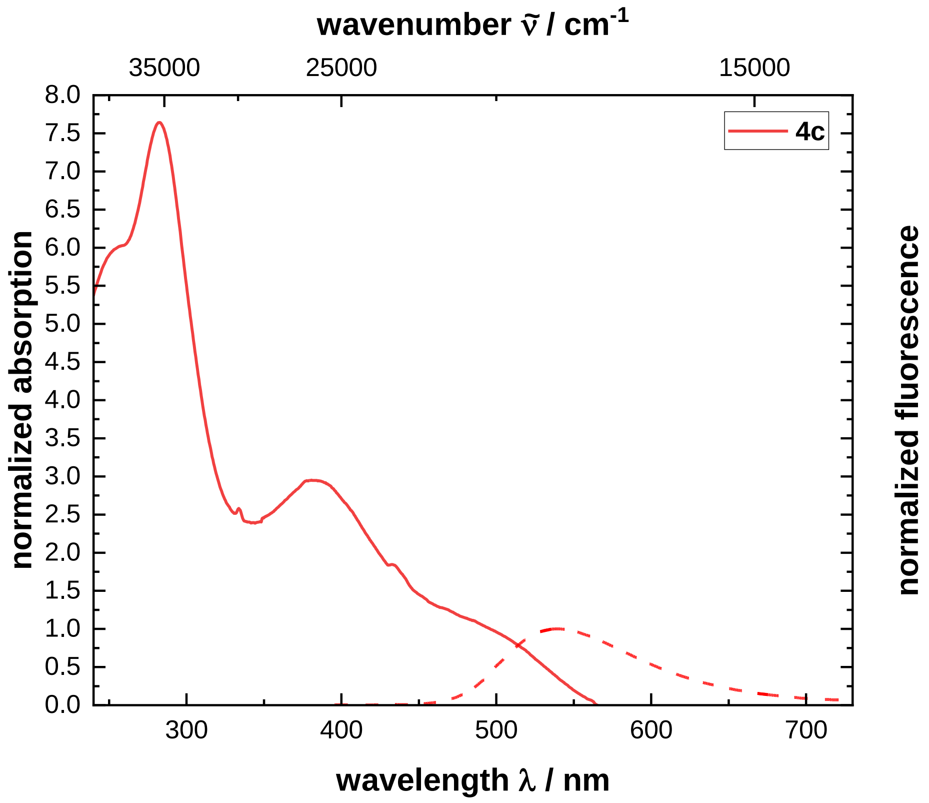


**Figure S27.** UV/Vis and normalized emission spectra (c(**4c**) = 10$^{-5}$ m) of polymer **4c** (recorded in $CH_2Cl_2$, *T* = 293 K).

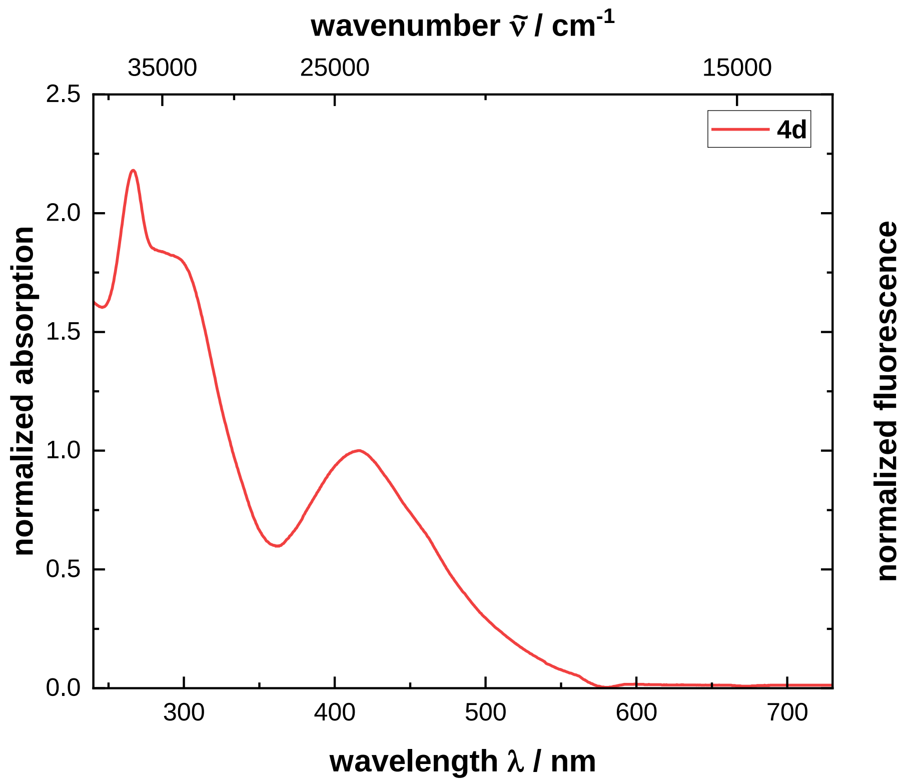


**Figure S28.** UV/Vis and normalized emission spectra (c(**4d**) = $10^{-5}$ m) of polymer **4d** (recorded in $CH_2Cl_2$, *T* = 293 K).

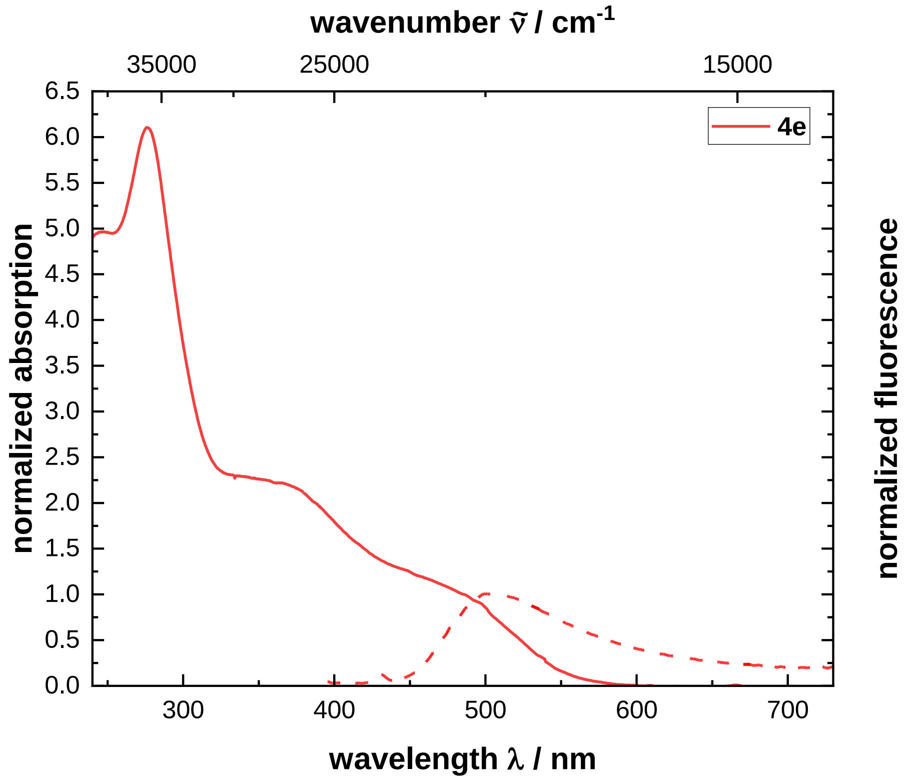


**Figure S29.** UV/Vis and normalized emission spectra (c(**4e**) = $10^{-5}$ m) of polymer **4e** (recorded in $CH_2Cl_2$, *T* = 293 K).

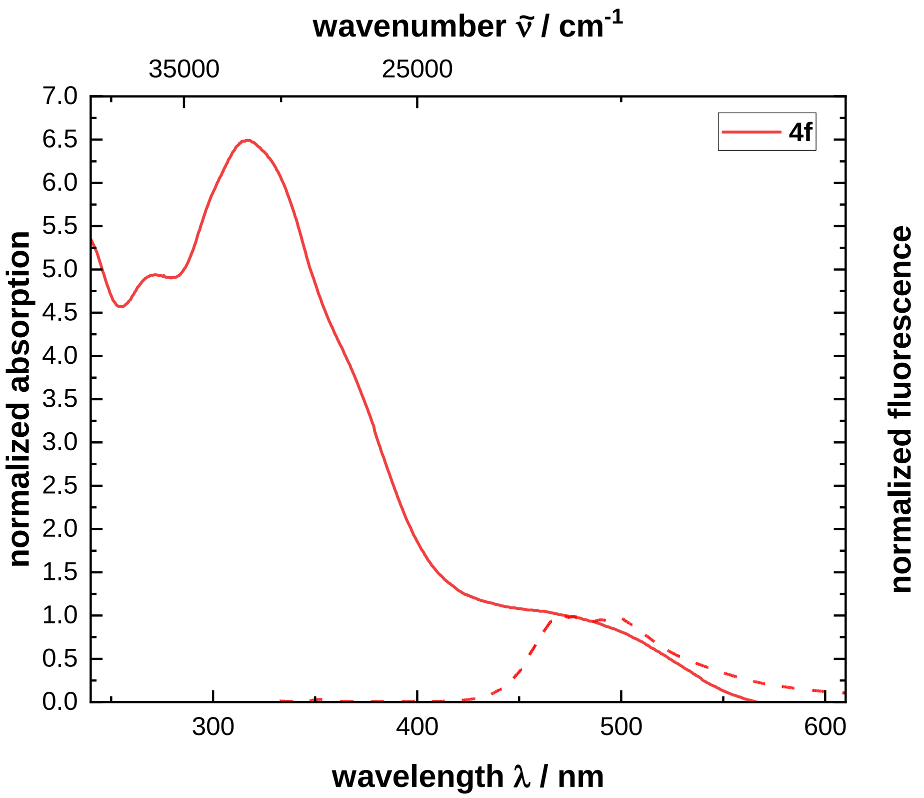


**Figure S30.** UV/Vis and normalized emission spectra (c(**4f**) = $10^{-5}$ m) of polymer **4f** (recorded in $CH_2Cl_2$, $T$ = 293 K).

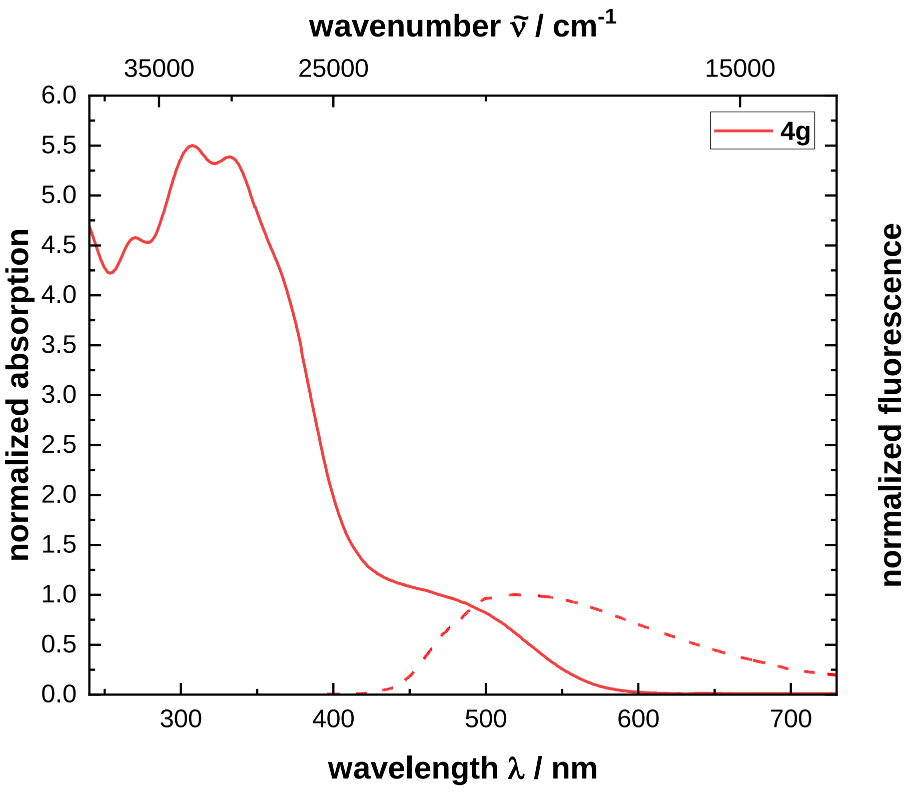


**Figure S31.** UV/Vis and normalized emission spectra (c(**4g**) = $10^{-5}$ m) of polymer **4g** (recorded in $CH_2Cl_2$, $T$ = 293 K).

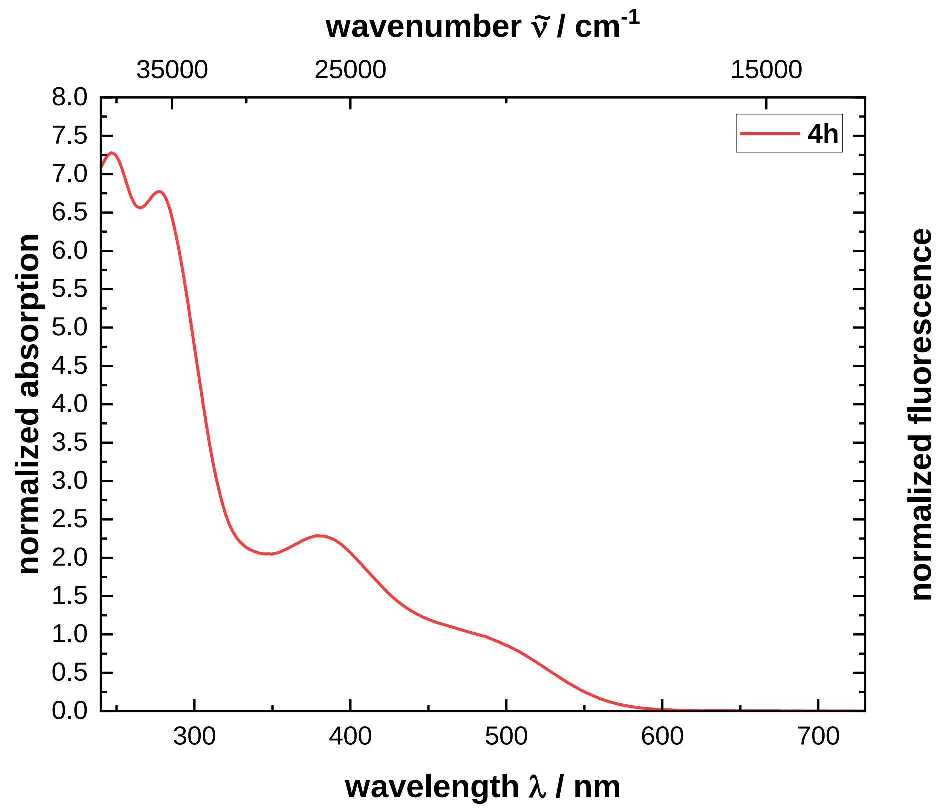


**Figure S32.** UV/Vis and normalized emission spectra (c(**4h**) = $10^{-5}$ m) of polymer **4h** (recorded in $CH_2Cl_2$, $T$ = 293 K).

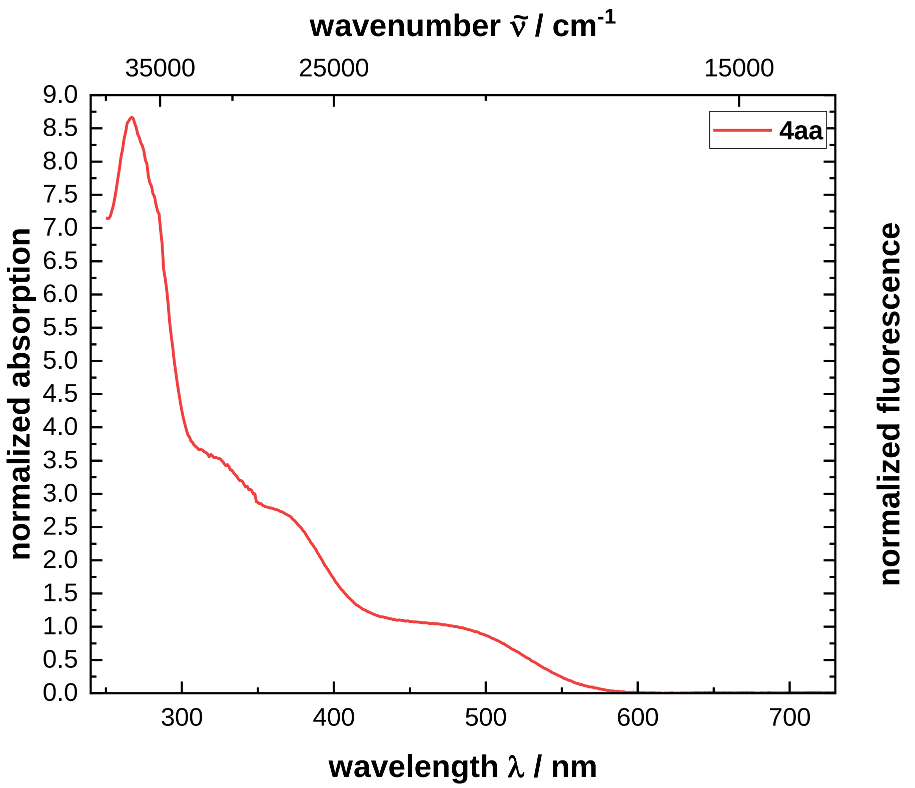


**Figure S33.** UV/Vis and normalized emission spectra (c(**4aa**) = $10^{-5}$ m) of polymer **4aa** (recorded in $CH_2Cl_2$, $T$ = 293 K).

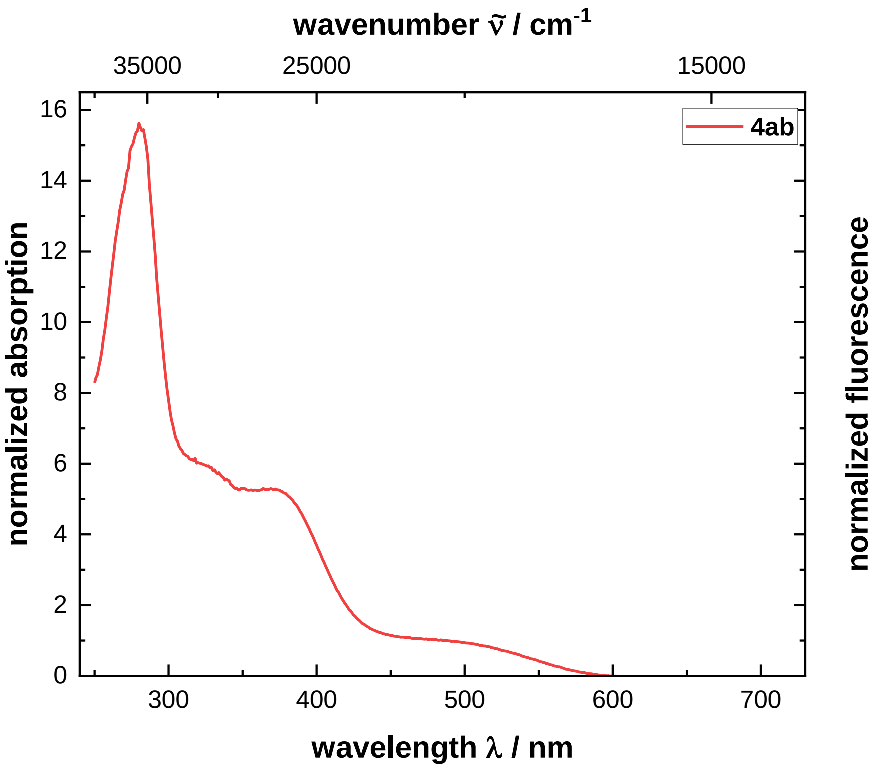


**Figure S34.** UV/Vis and normalized emission spectra (c(**4ab**) = $10^{-5}$ m) of polymer **4ab** (recorded in $CH_2Cl_2$, $T$ = 293 K).

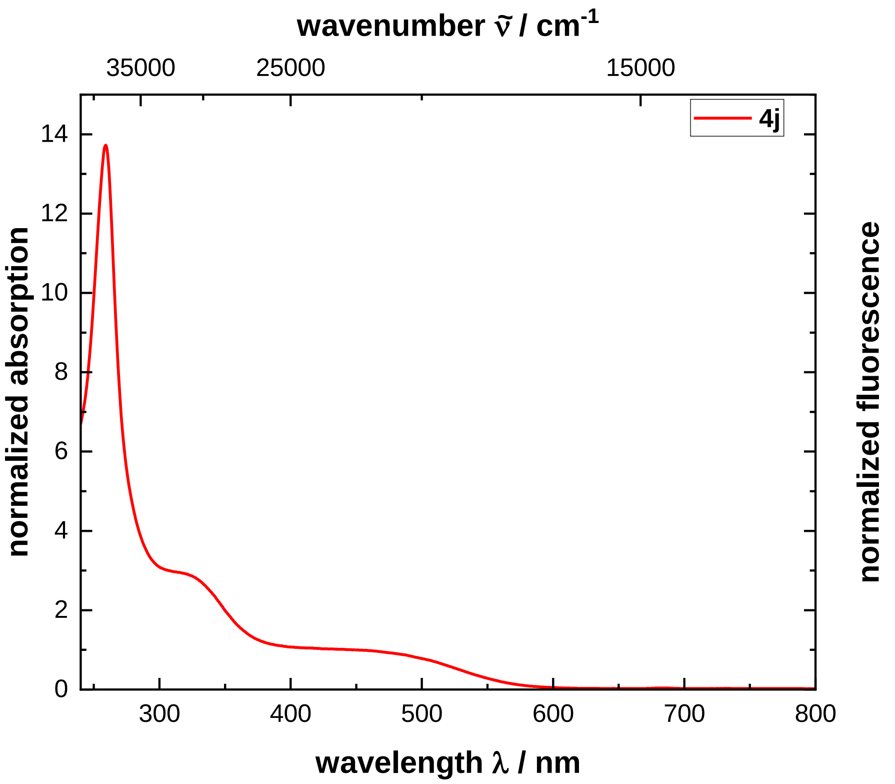


Figure S35. UV/Vis and normalized emission spectra (c(**4j**) = $10^{-5}$ m) of polymer **4j** (recorded in $CH_2Cl_2$, $T$ = 293 K).

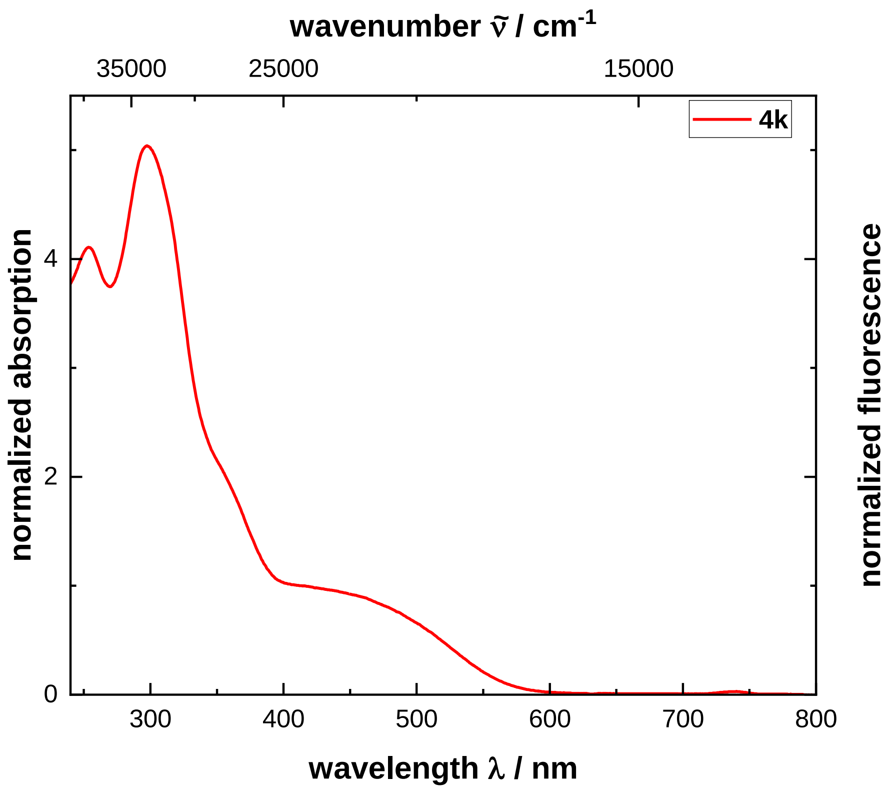


**Figure S36.** UV/Vis and normalized emission spectra (c(**4k**) = $10^{-5}$ m) of polymer **4k** (recorded in $CH_2Cl_2$, $T$ = 293 K).

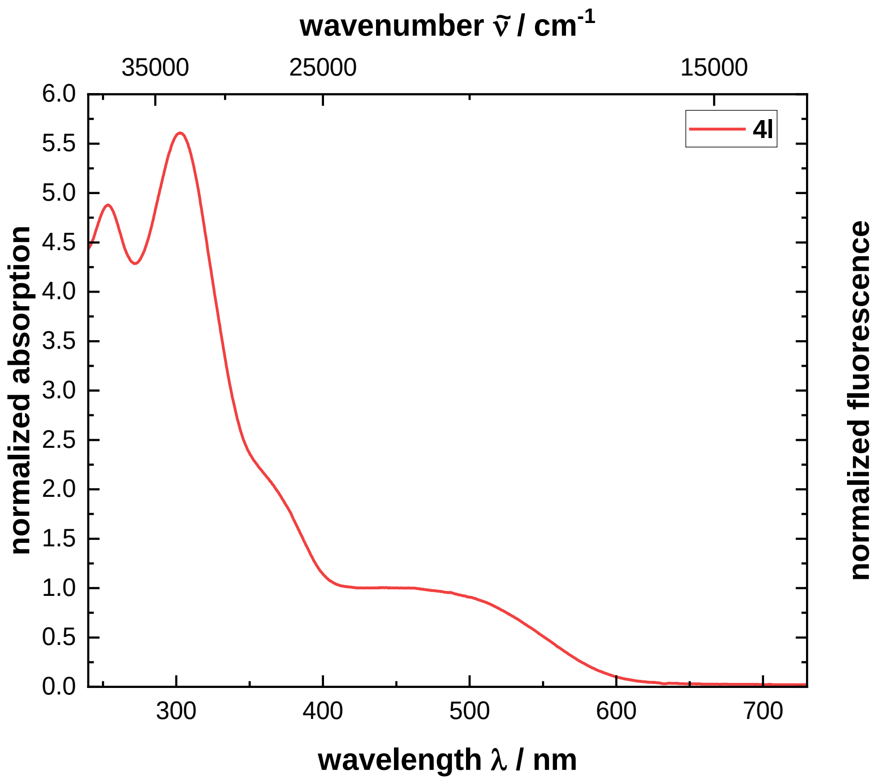


**Figure S37.** UV/Vis and normalized emission spectra (c(**4l**) = $10^{-5}$ m) of polymer **4l** (recorded in $CH_2Cl_2$, $T$ = 293 K).

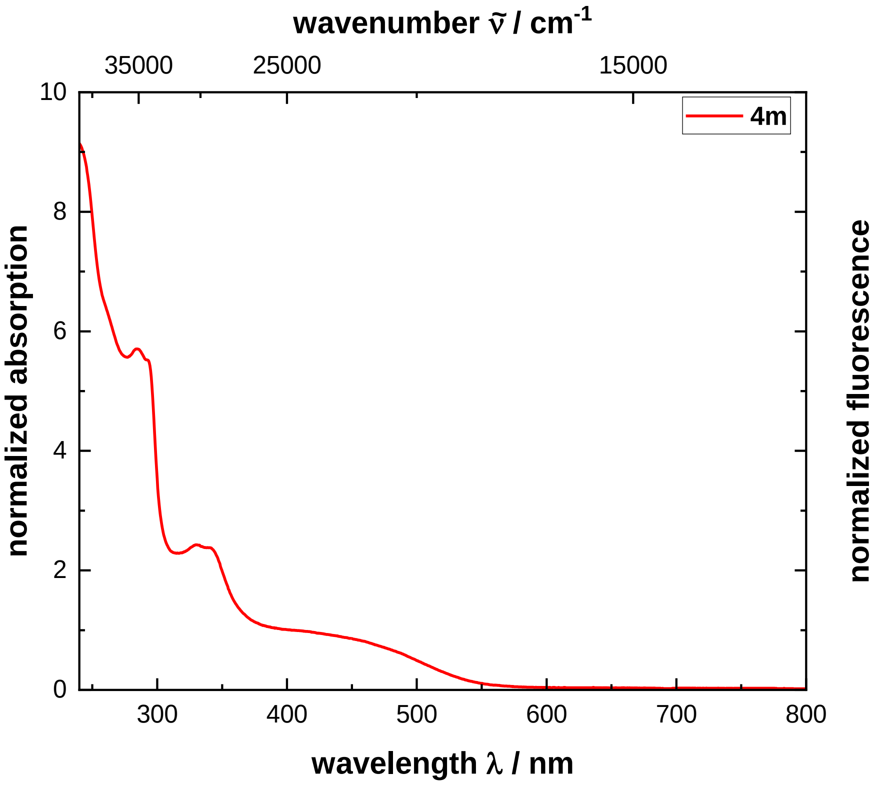


**Figure S38.** UV/Vis and normalized emission spectra (c(**4m**) = $10^{-5}$ m) of polymer **4m** (recorded in $CH_2Cl_2$, $T$ = 293 K).

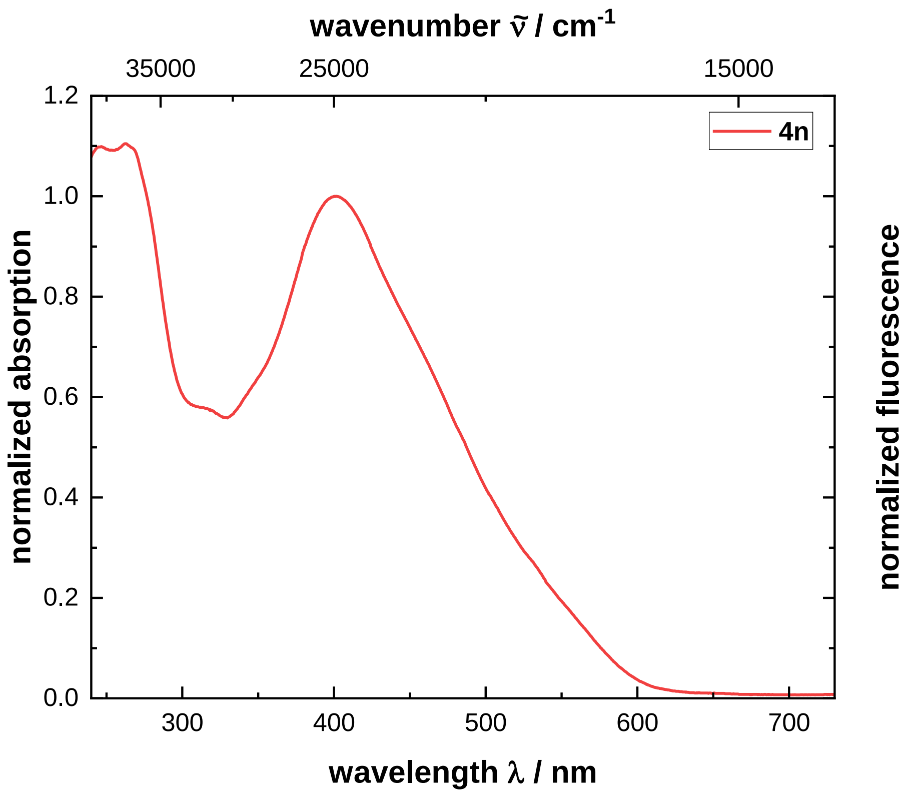


**Figure S39.** UV/Vis and normalized emission spectra (c(**4n**) = $10^{-5}$ m) of polymer **4n** (recorded in $CH_2Cl_2$, *T* = 293 K).

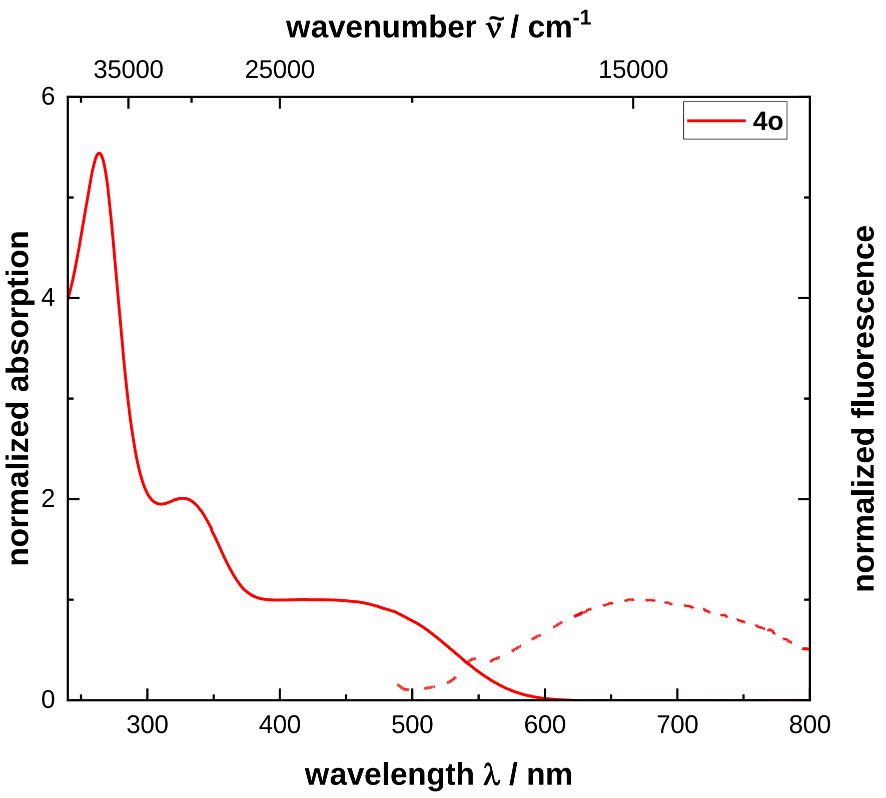


**Figure S40.** UV/Vis and normalized emission spectra (c(**4o**) = $10^{-5}$ m) of polymer **4o** (recorded in $CH_2Cl_2$, *T* = 293 K).

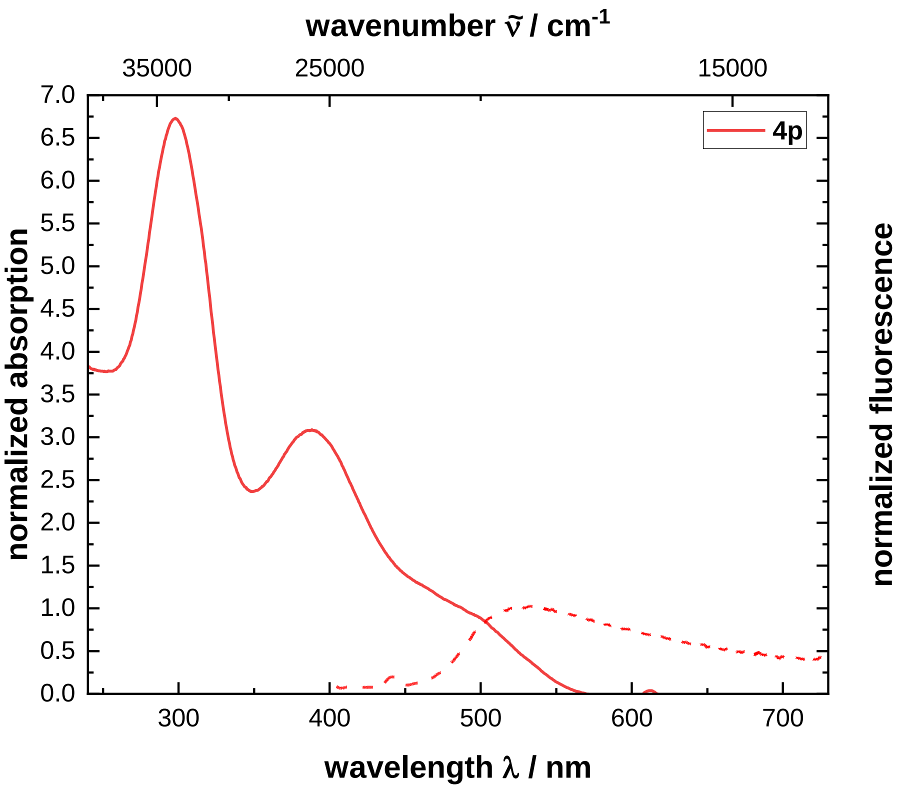


**Figure S41.** UV/Vis and normalized emission spectra (c(**4p**) = $10^{-5}$ m) of polymer **4p** (recorded in $CH_2Cl_2$, $T$ = 293 K).

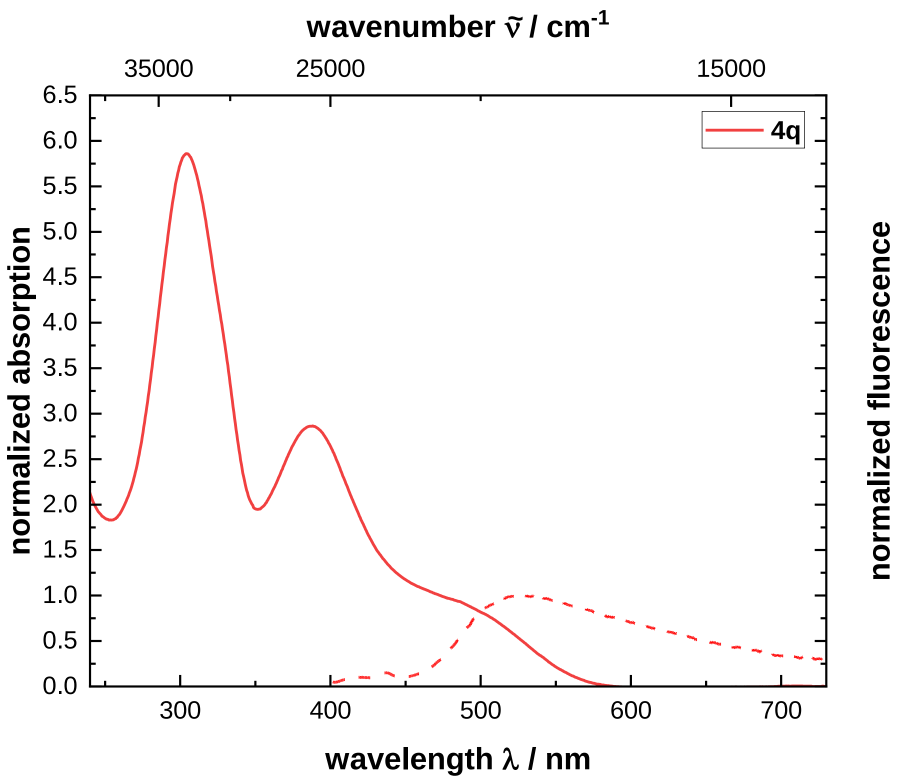


**Figure S42.** UV/Vis and normalized emission spectra (c(**4q**) = $10^{-5}$ m) of polymer **4q** (recorded in $CH_2Cl_2$, $T$ = 293 K).

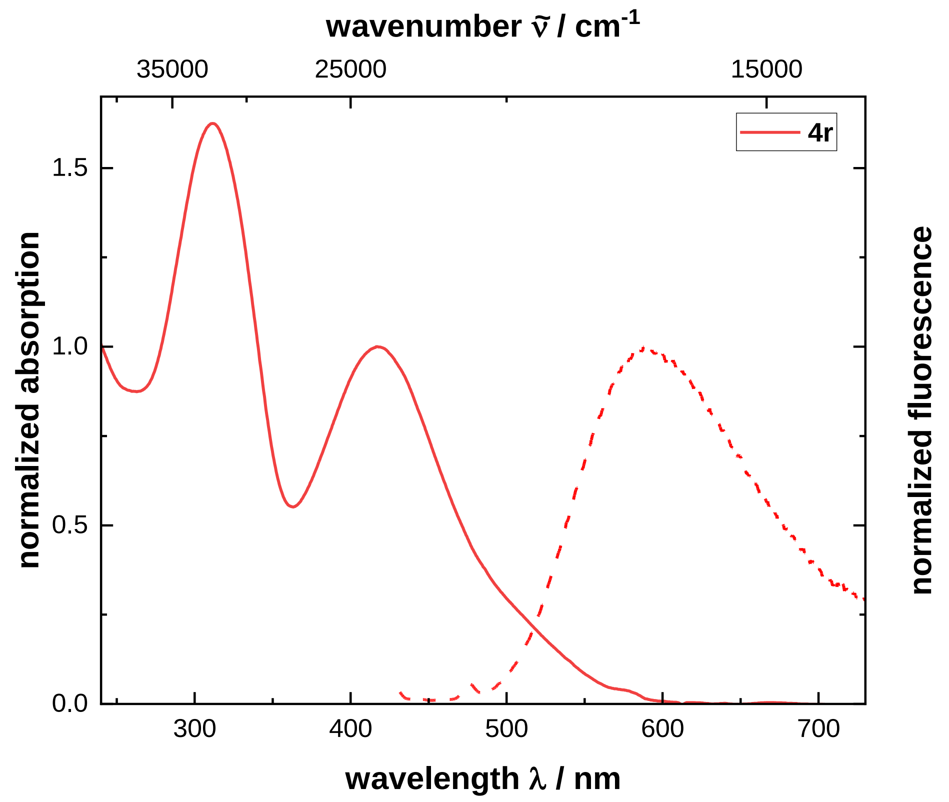


**Figure S43.** UV/Vis and normalized emission spectra (c(**4r**) = $10^{-5}$ m) of polymer **4r** (recorded in $CH_2Cl_2$, $T$ = 293 K).

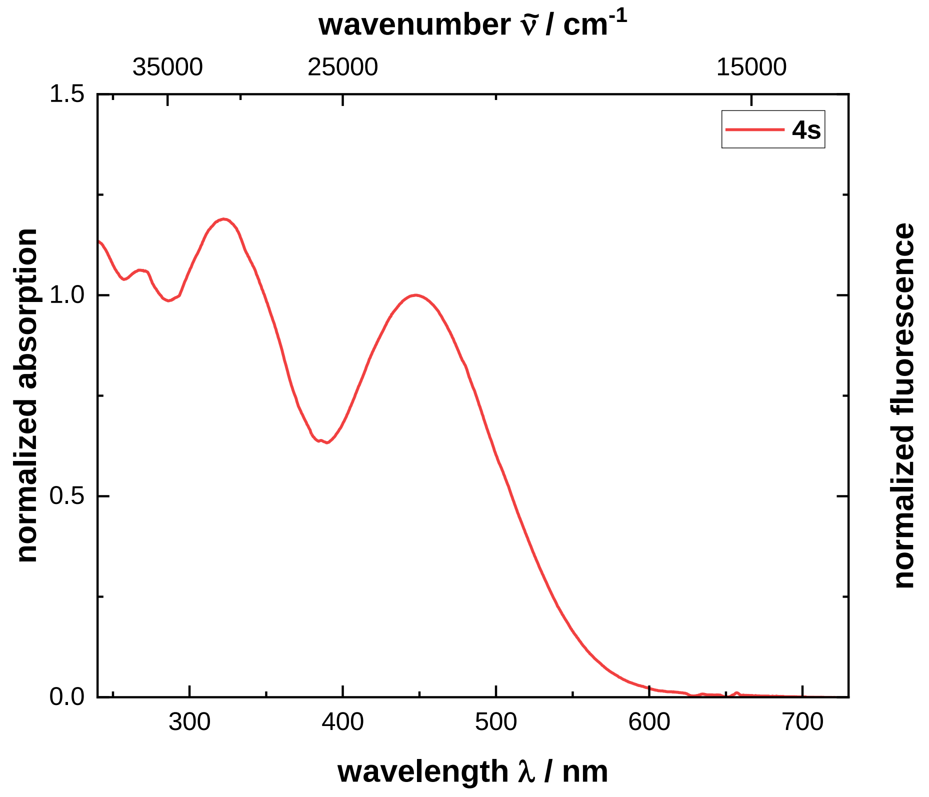


**Figure S44.** UV/Vis and normalized emission spectra (c(**4s**) = $10^{-5}$ m) of polymer **4s** (recorded in $CH_2Cl_2$, $T$ = 293 K).

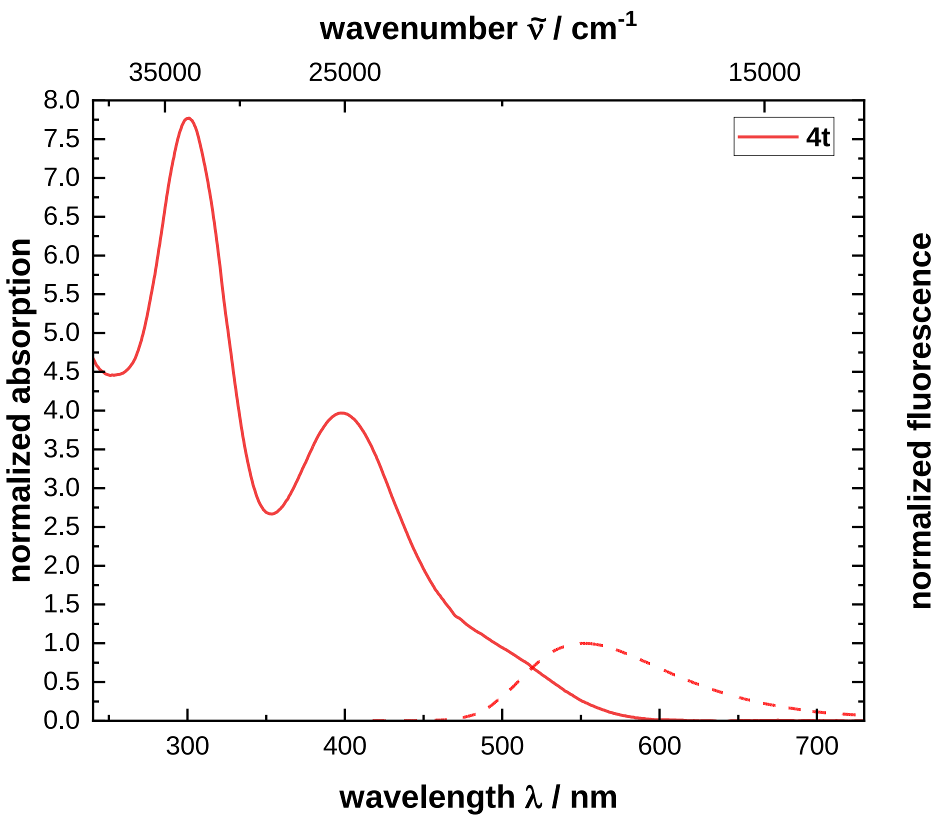


**Figure S45.** UV/Vis and normalized emission spectra (c(**4t**) = $10^{-5}$ m) of polymer **4s** (recorded in $CH_2Cl_2$, $T$ = 293 K).

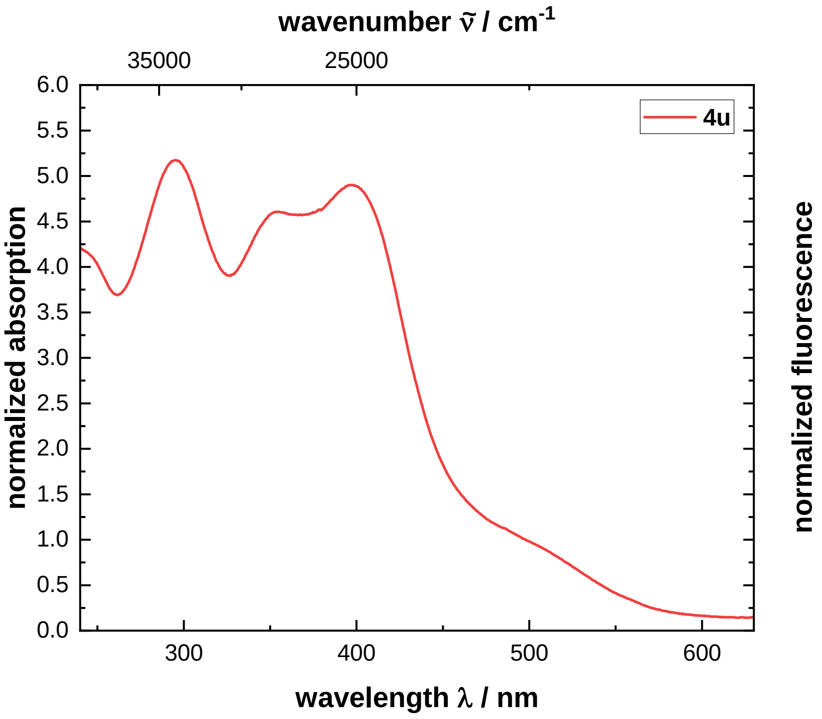


**Figure S46.** UV/Vis and normalized emission spectra (c(**4u**) = $10^{-5}$ m) of polymer **4s** (recorded in $CH_2Cl_2$, $T$ = 293 K).

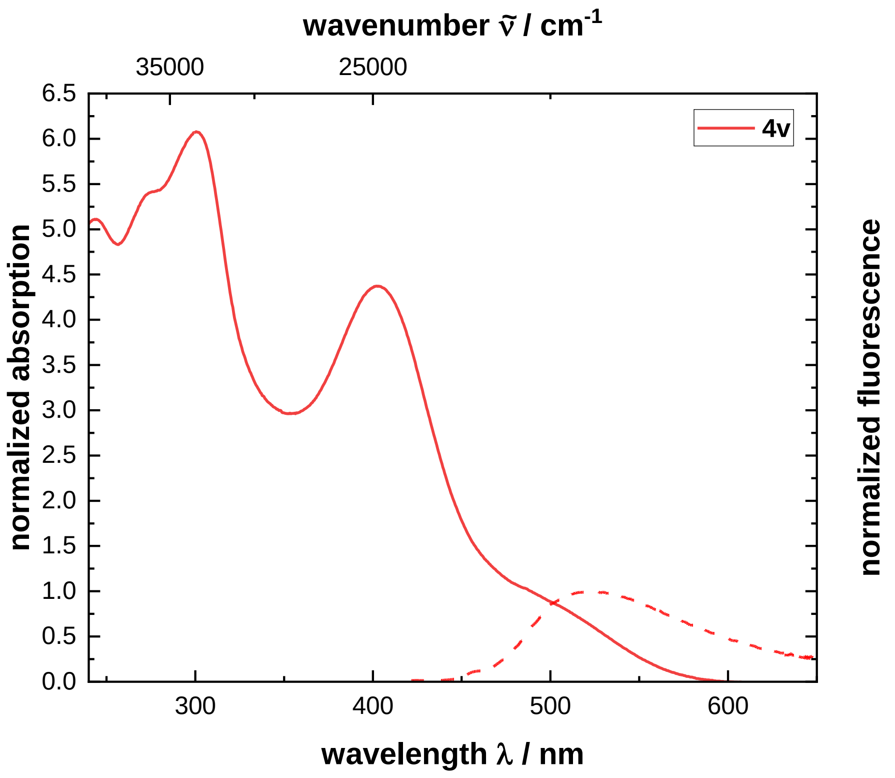


**Figure S47.** UV/Vis and normalized emission spectra (c(**4v**) = $10^{-5}$ m) of polymer **4v** (recorded in $CH_2Cl_2$, $T$ = 293 K).

**Table S9.** Photophysical data of the synthesized monomers **3** and of the corresponding polymers **4** recorded in dichloromethane (DCM).

| | $\lambda_{max,abs}$ [nm] ($\varepsilon$ [L mol$^{-1}$ cm$^{-1}$]) | $\lambda_{max,em}$ [nm] | $\Phi_F$ | Stokes shift $\Delta\tilde{\nu}$ [cm$^{-1}$][a] |
|---|---|---|---|---|
| **3a** | 334 (8200), 277 (39900) | 466 | 0.66 | 8500 |
| **3b** | 338 (10000), 279 (36500), 245 (19000) | 465 | 0.48 | 8100 |
| **3c** | 372 (11800), 283 (42900), 249 (24700) | 525 | 0.76 | 7800 |
| **3d** | 409 (9800), 293 (18800), 267 (25600) | - | - | - |
| **3e** | 342 (10400), 279 (42900) | 497 | 0.69 | 9100 |
| **3f** | 328 (43500), 275 (46600) | 471 | 0.47 | 9600 |
| **3g** | 332 (26700), 308 (23600), 273 (33800) | 471 | 0.41 | 8900 |
| **3h** | 370 (6700), 279 (29900) | 520 | 0.50 | 7800 |
| **3i** | 387 (6300), 367 (7900), 348 (8800), 320 (11600), 256 (59800) | 425 | 0.35 | 2300 |
| **3aa** | 364 (15000), 325 (16900), 277 (47600) | 471 | 0.57 | 6200 |
| **3ab** | 370 (31300), 328 (32800), 278 (89400) | 478 | 0.40 | 6100 |
| **3j** | 327 (13300), 269 (62500), 260 (75300) | 487 | 0.26 | 10000 |
| **3k** | 343 (13000), 301 (27600), 261 (32700) | 497 | 0.37 | 9000 |
| **3l** | 355 (10700), 304 (23900), 262 (29000) | 499 | 0.38 | 8100 |
| **3m** | 340 (14200), 330 (14700), 268 (46000) | 486 | 0.39 | 8800 |
| **3n** | 406 (21700), 273 (32400), 249 (29500) | - | - | - |
| **3o** | 334 (14500), 271 (42200), 252 (36700) | 497 | 0.24 | 9800 |
| **3p** | 374 (14300), 314 (21200), 283 (34900) | 500 | 0.28 | 6700 |
| **3q** | 374 (15400), 302 (26900), 281 (29600) | 499 | 0.17 | 6700 |
| **3r** | 397 (25100), 325 (28300), 301 (35900), 259 (24900) | 570 | 0.87 | 7600 |
| **3s** | 431 (25800), 321 (25500), 287 (26900), 272 (26900) | - | - | - |
| **3t** | 385 (18600), 292 (35500) | 535 | 0.84 | 7300 |
| **3u** | 395 (28000), 350 (25000), 282 (37500) | 499 | 0.29 | 5300 |

| | | | | |
|---|---|---|---|---|
| **3v** | 397 (27000), 303 (32600), 273 (45500) | 497 | 0.30 | 5200 |
| **4a** | 464 (645500), 327 (1348300), 273 (3719600) | 499 | - | 10500 |
| **4b** | 405 (535500), 288 (1266700) | 552 | - [b] | 6600 |
| **4c** | 497 (508300), 322 (640200), 260 (431000) | 686 | - | 5500 |
| **4d** | 500 (720300), 338 (1834000), 276 (5767200) | - | - | - |
| **4e** | 490 (912400), 362 (2107500), 276 (5653700) | 550 | - | 7600 |
| **3f** | 491 (1129500), 317 (9028700), 270 (6859400) | 471 | _[b] | 10300 |
| **4g** | 500 (517300), 333 (3418900), 309 (3493900), 270 (2909100) | 519 | - | 10700 |
| **4h** | 500 (2326000), 380 (6182000), 278 (18317000), 247 (19690800) | - | - | - |
| **4i** | - | - | - | - |
| **4aa** | 465 (470000), 259 (22000) | - | - | - |
| **4ab** | 468 (130000), 289 (54000) | | | |
| **4j** | 480 (89100), 324 (284700), 259 (1366400) | - | - | - |
| **4k** | 463 (54000), 358 (118200), 297 (293200), 254 (245400) | - | - | - |
| **4l** | 480 (171300), 365 (364600), 303 (989500), 253 (859600) | - | - | - |
| **4m** | 423 (86700), 339 (211300), 330 (214000), 284 (487800), | - | - | - |
| **4n** | 401 (11629500), 267 (12731300) | - | - | - |
| **4o** | 489 (471400), 326 (1083800), 264 (2885300) | - | - | - |
| **4p** | 495 (382600), 388 (1287200), 298 (2811500) | 519 | - | 6500 |

| | | | | |
|---|---|---|---|---|
| **4q** | 498 (989100), 384 (3511100), 303 (7204900) | 529 | - | 7100 |
| **4r** | 514 (1820300), 417 (7593500), 311 (12271300) | 589 | 0.05 | 7000 |
| **4s** | 449 (6906200), 322 (8179500), 268 (7324600) | - | - | - |
| **4t** | 500 (7041500), 399 (29152800), 301 (56996000) | 551 | 0.06 | 6900 |
| **4u** | 498 (2274800), 397 (11144800), 355 (10474700), 295 (11770800) | - | - | - |
| **4v** | 494 (292600), 402 (1348200), 300 (1874800), 276 (1670000) | 525 | - | 5800 |

# 4.0 IR spectra

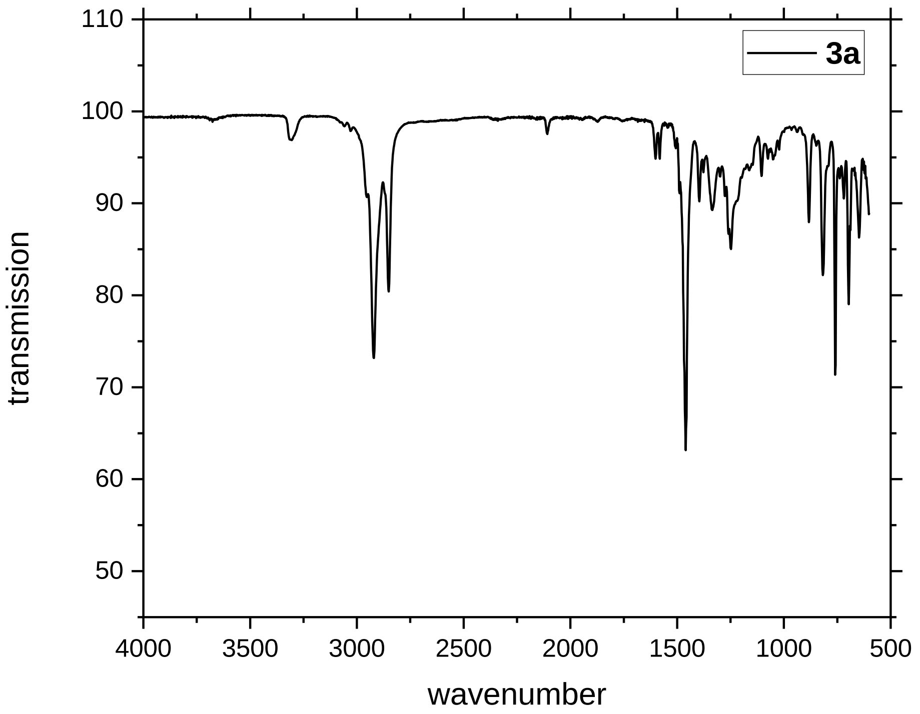


**Figure S48.** IR spectrum of monomer **3a**.

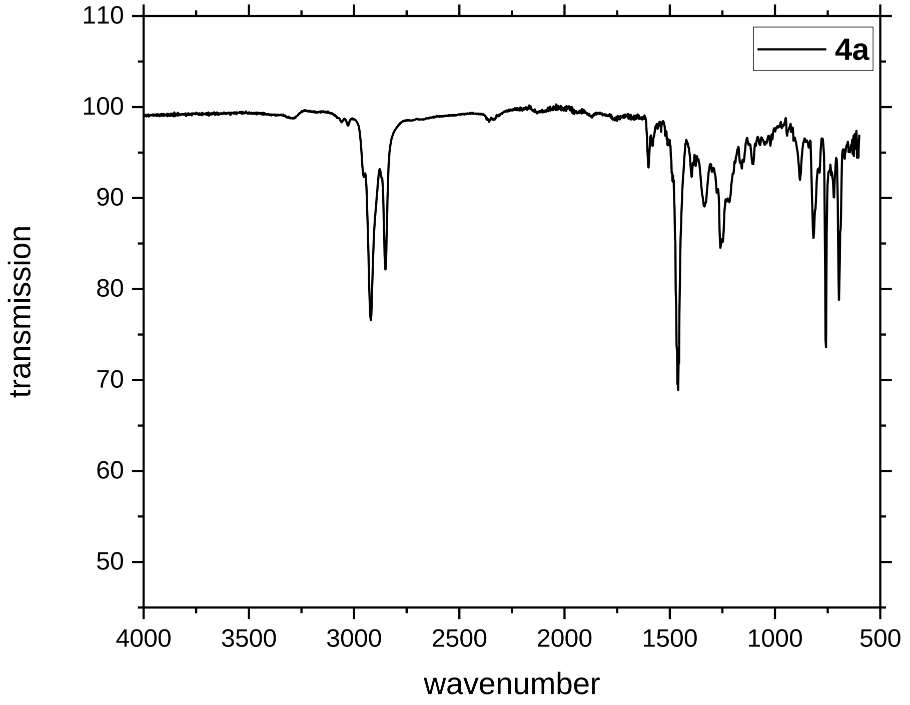


**Figure S49.** IR spectrum of polymer **4a**.

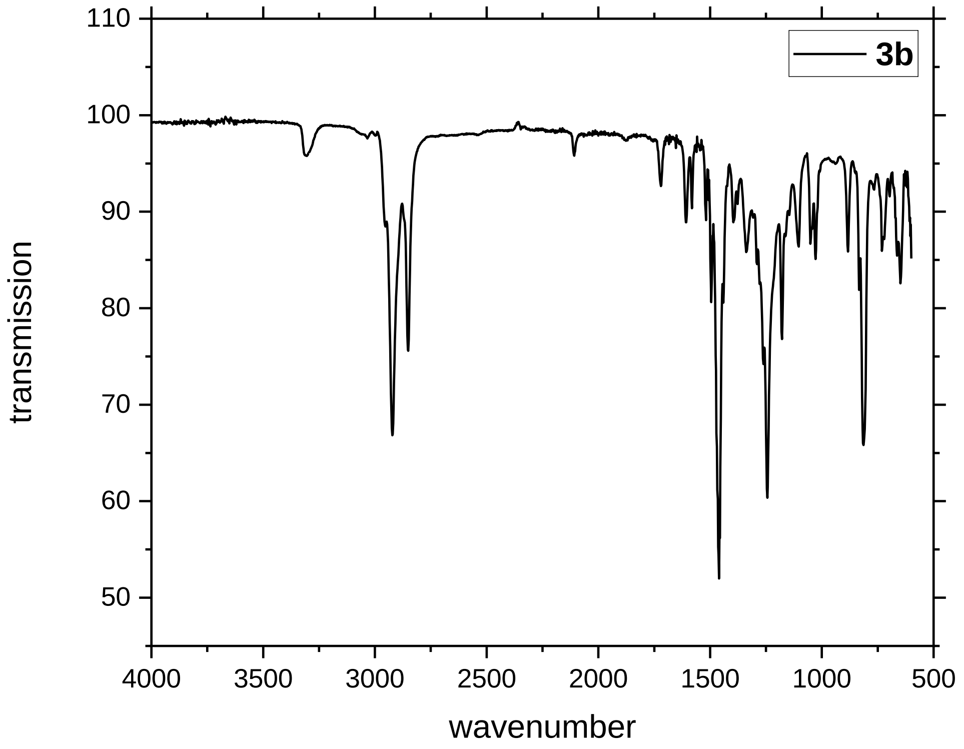


**Figure S50.** IR spectrum of monomer **3b**.

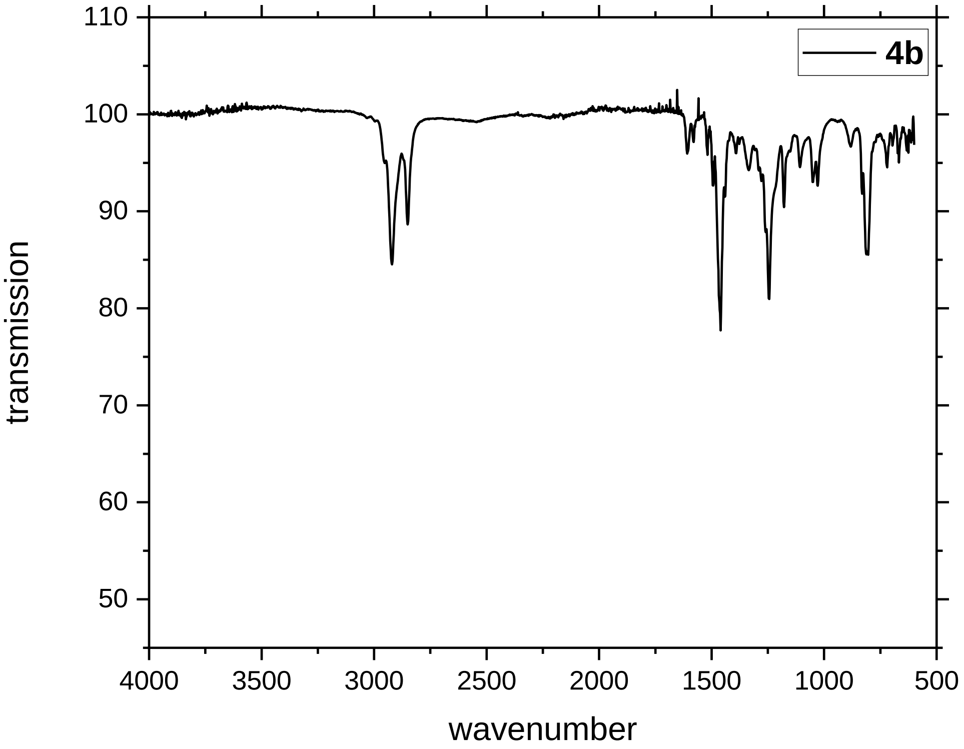


**Figure S51.** IR spectrum of polymer **4b**.

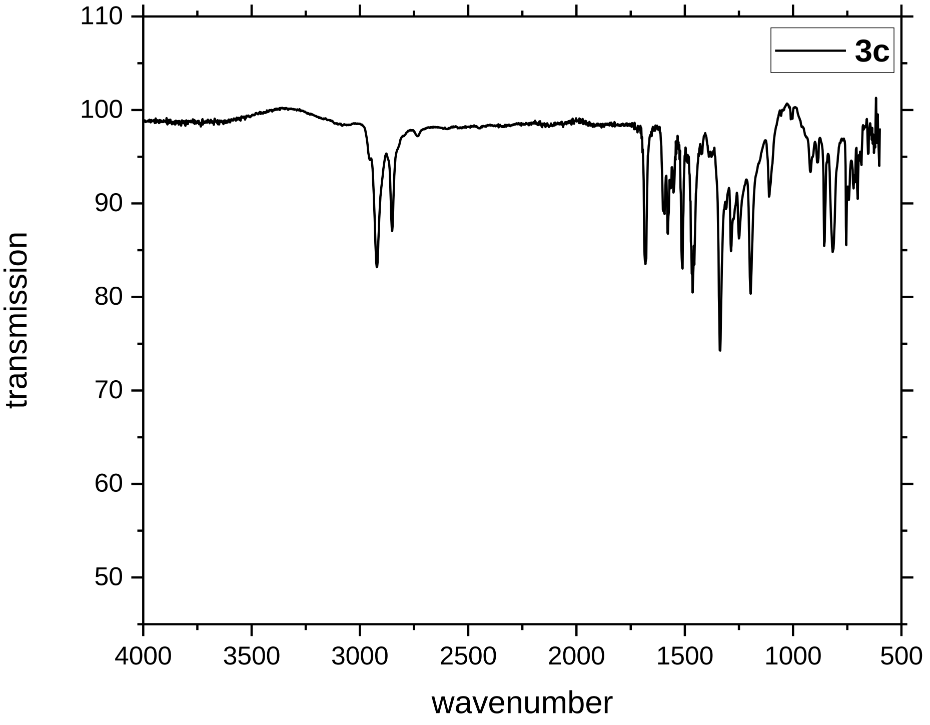


**Figure S52.** IR spectrum of monomer **3c**.

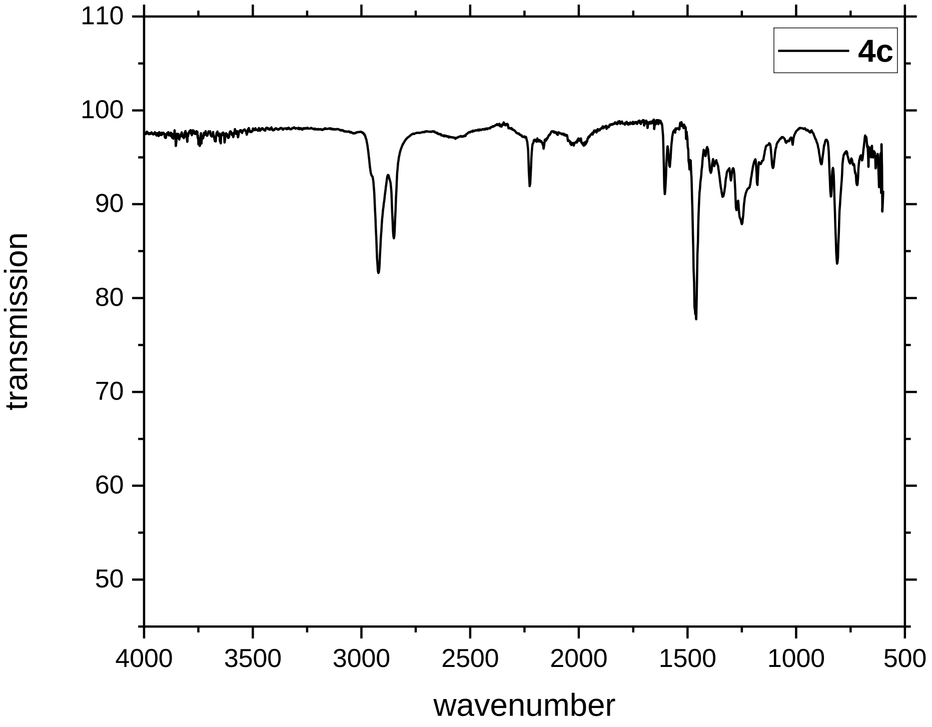


**Figure S53.** IR spectrum of polymer **4c**.

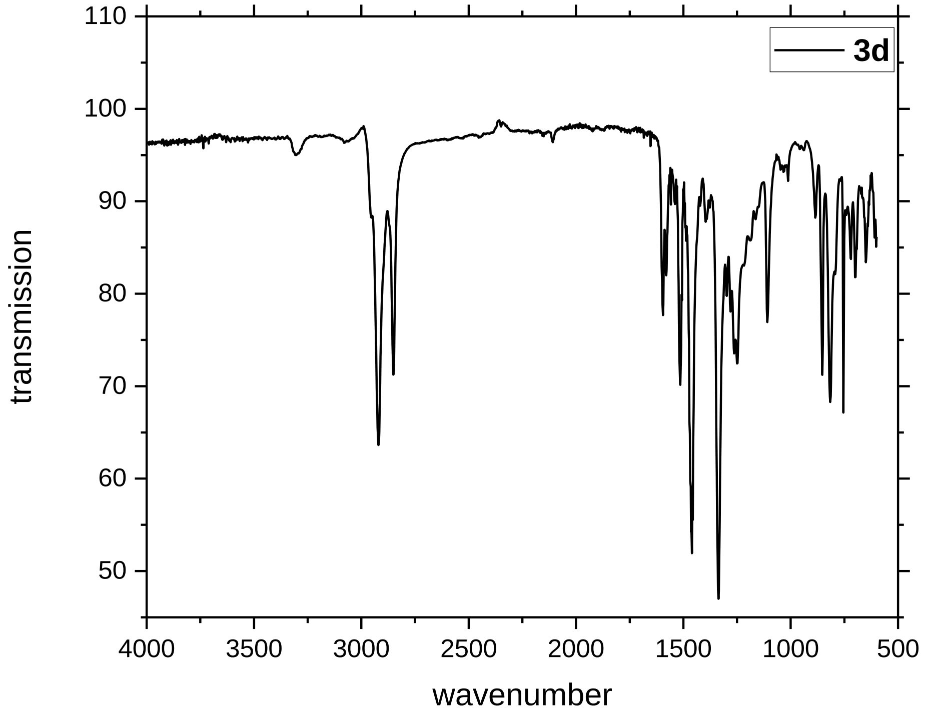


**Figure S54.** IR spectrum of monomer **3d**.

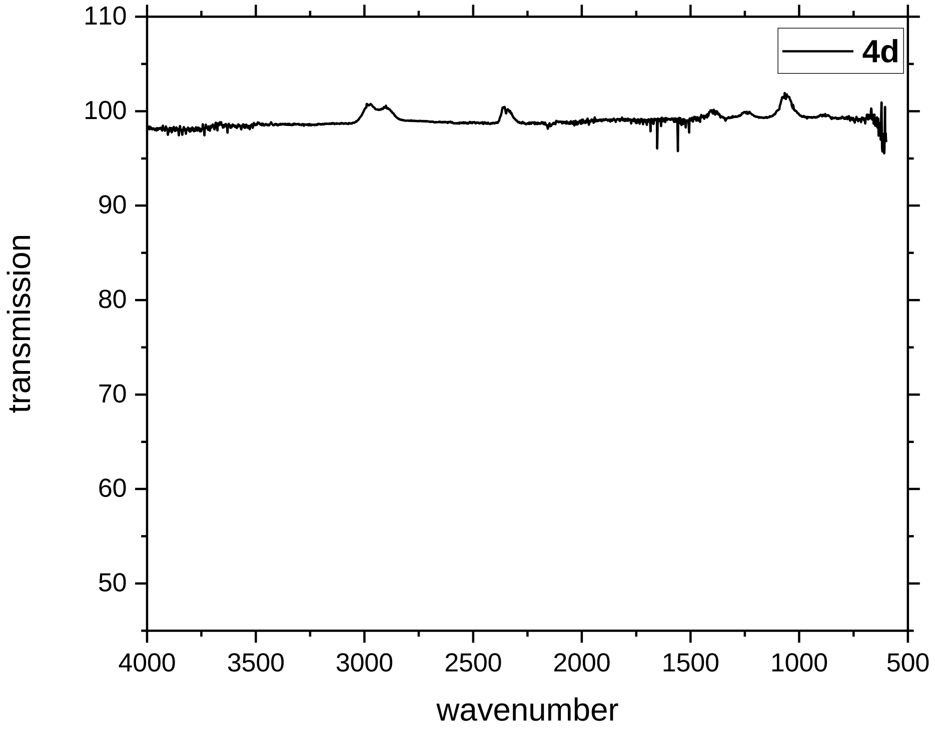


**Figure S55.** IR spectrum of polymer **4d**.

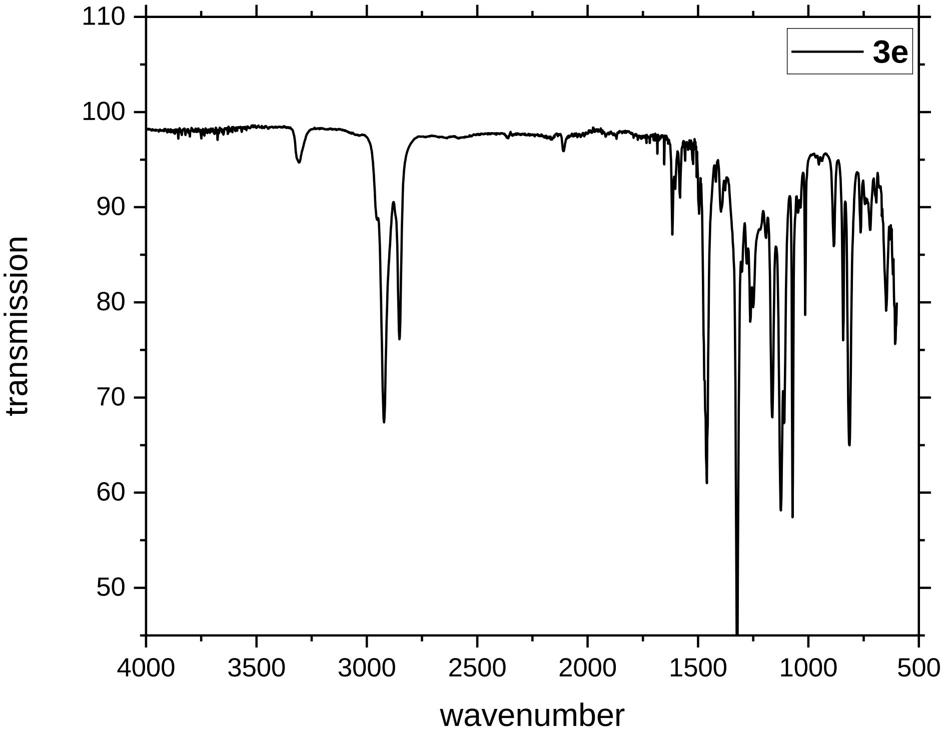


**Figure S56.** IR spectrum of monomer **3e**.

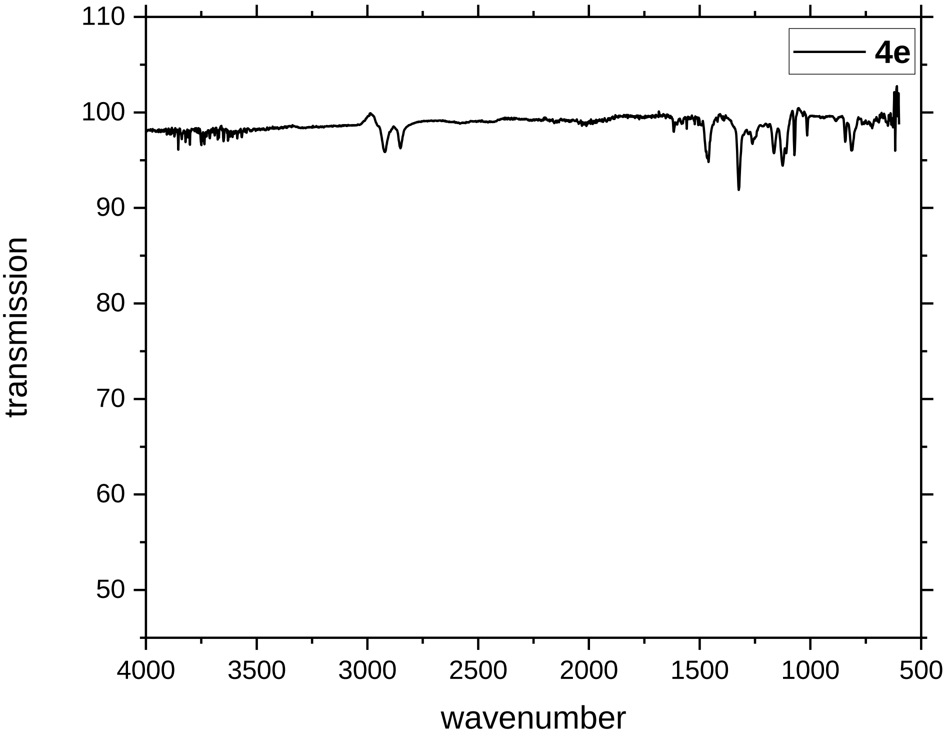


**Figure S57.** IR spectrum of polymer **4e**.

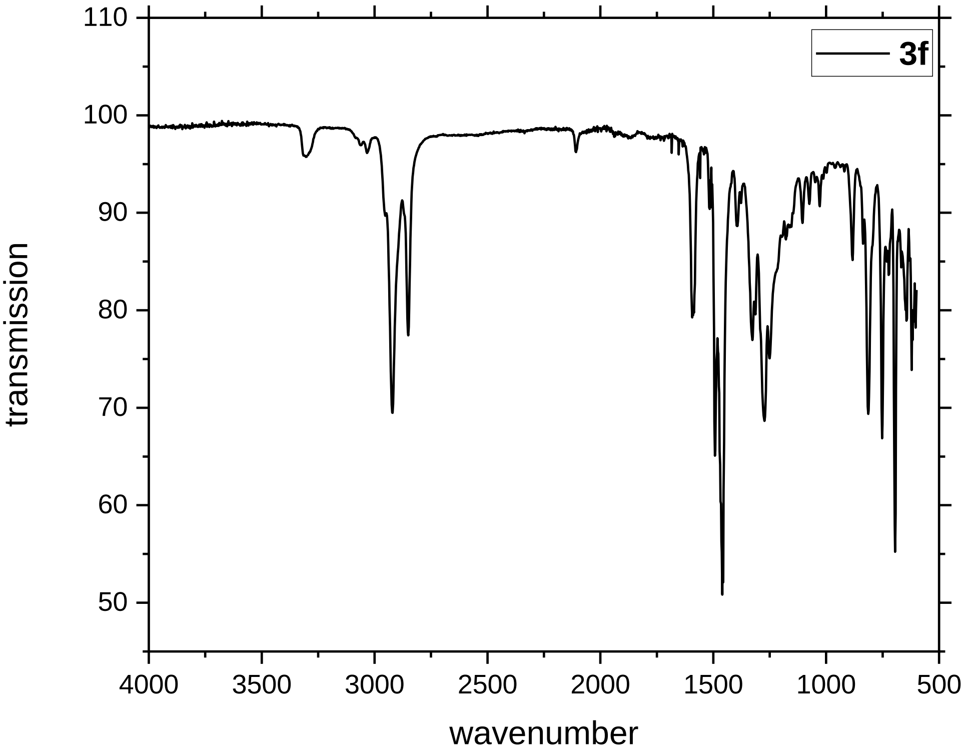


**Figure S58.** IR spectrum of monomer **3f**.

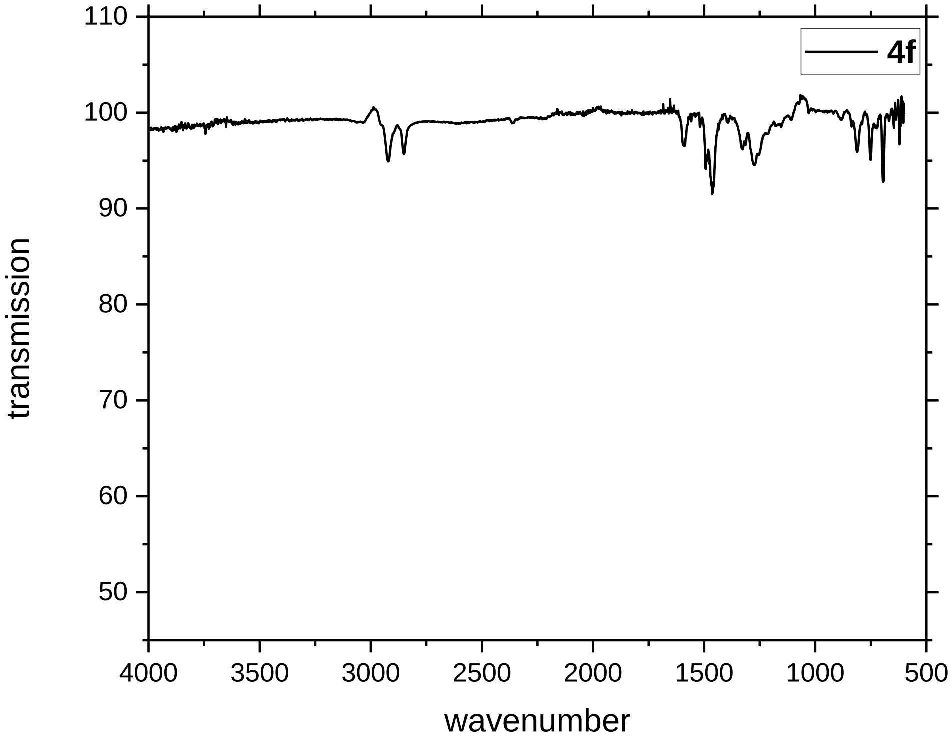


**Figure S59.** IR spectrum of polymer **4f**.

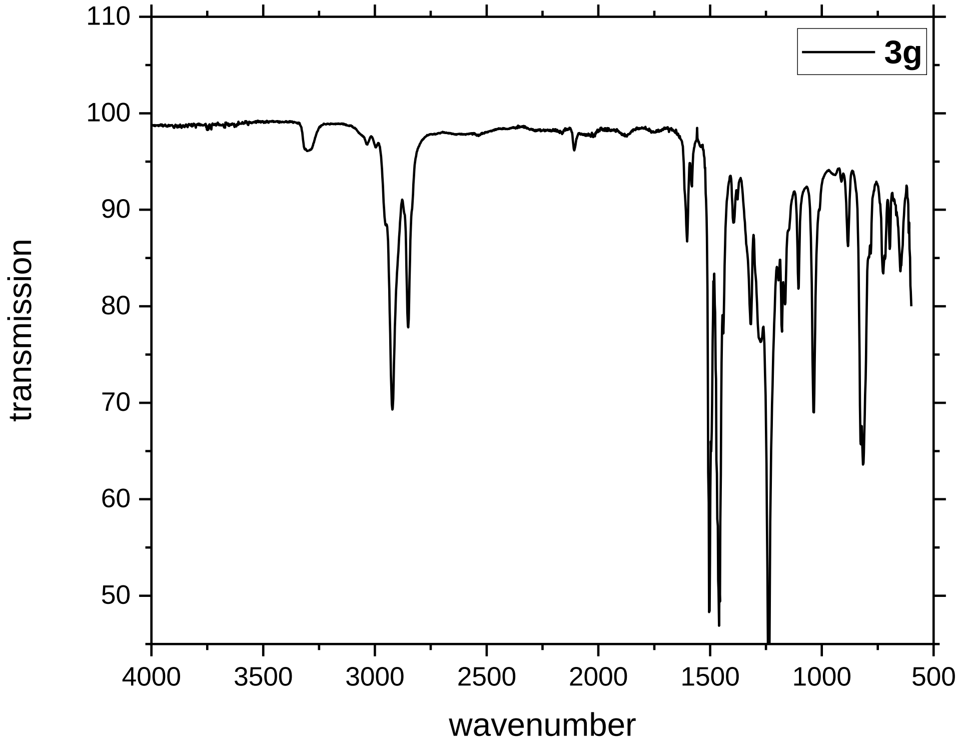


**Figure S60.** IR spectrum of monomer **3g**.

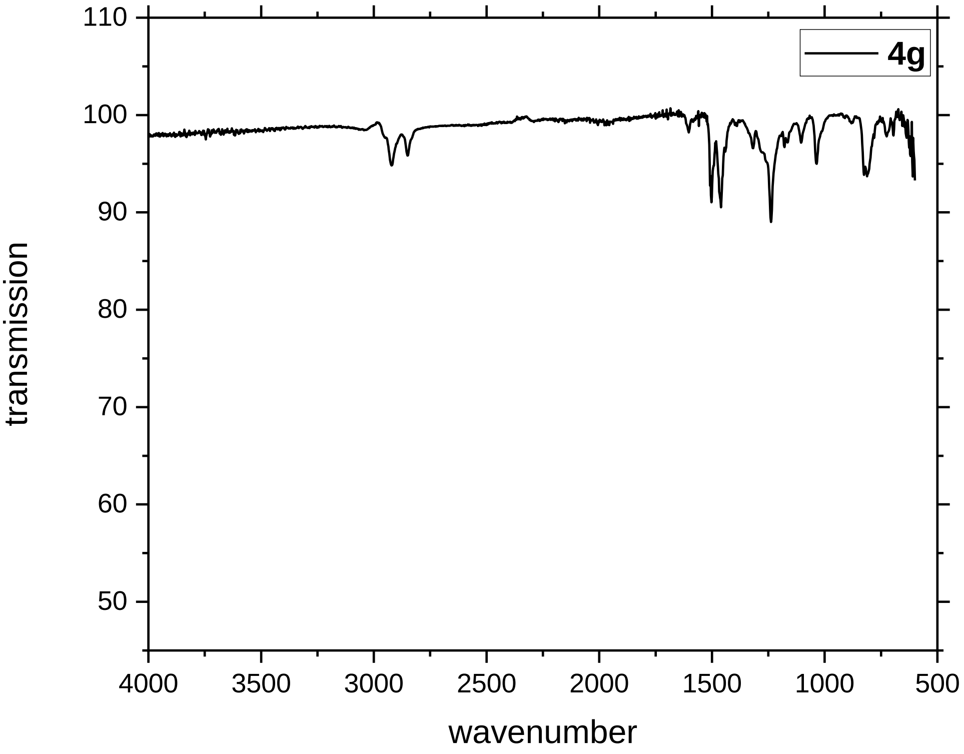


**Figure S61.** IR spectrum of polymer **4g**.

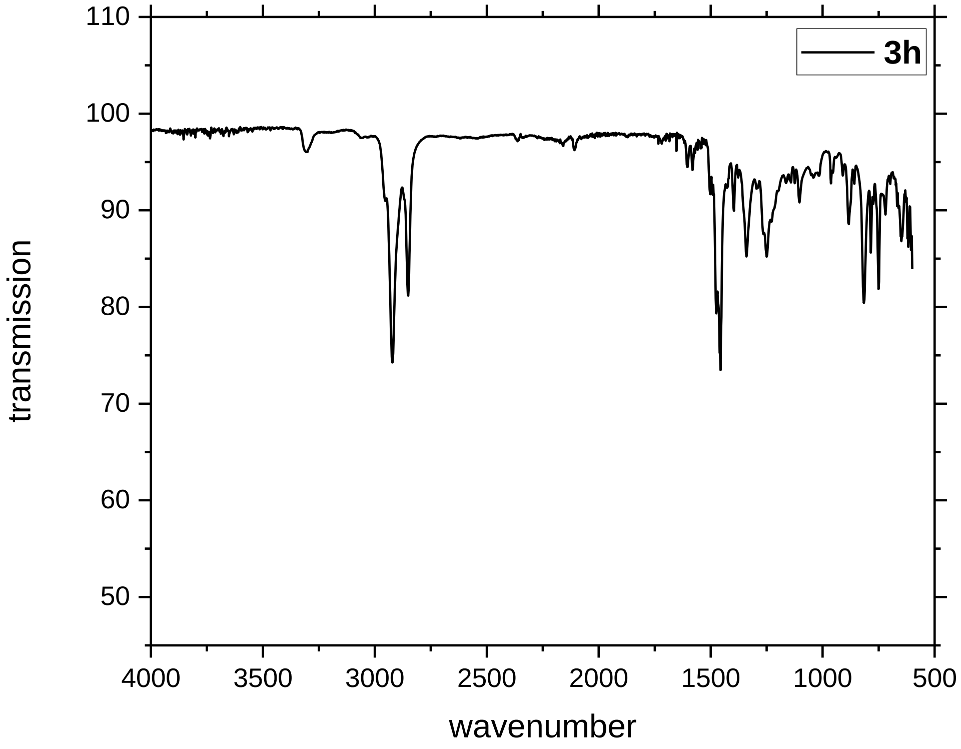


**Figure S62.** IR spectrum of monomer **3h**.

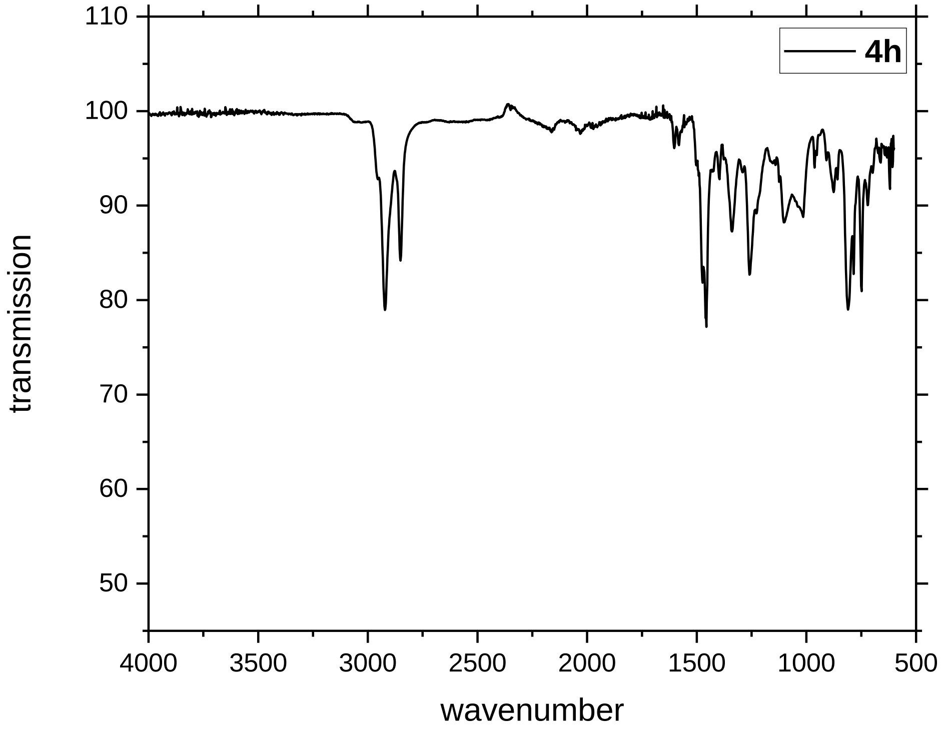


**Figure S63.** IR spectrum of polymer **4h**.

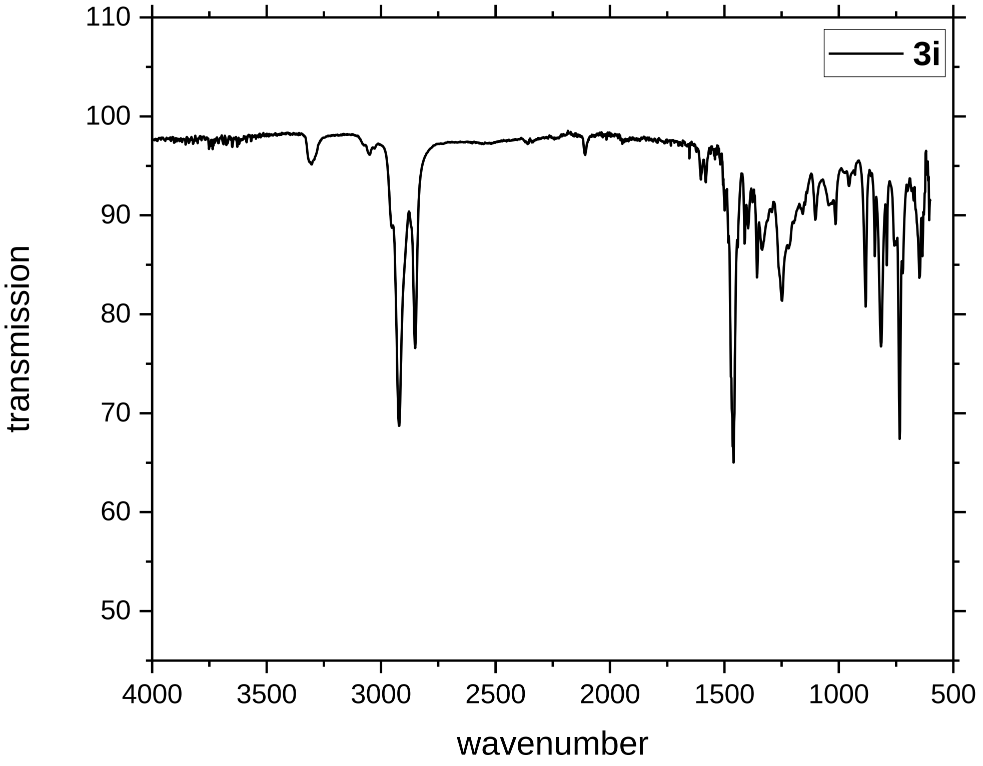


**Figure S64.** IR spectrum of monomer **3i**.

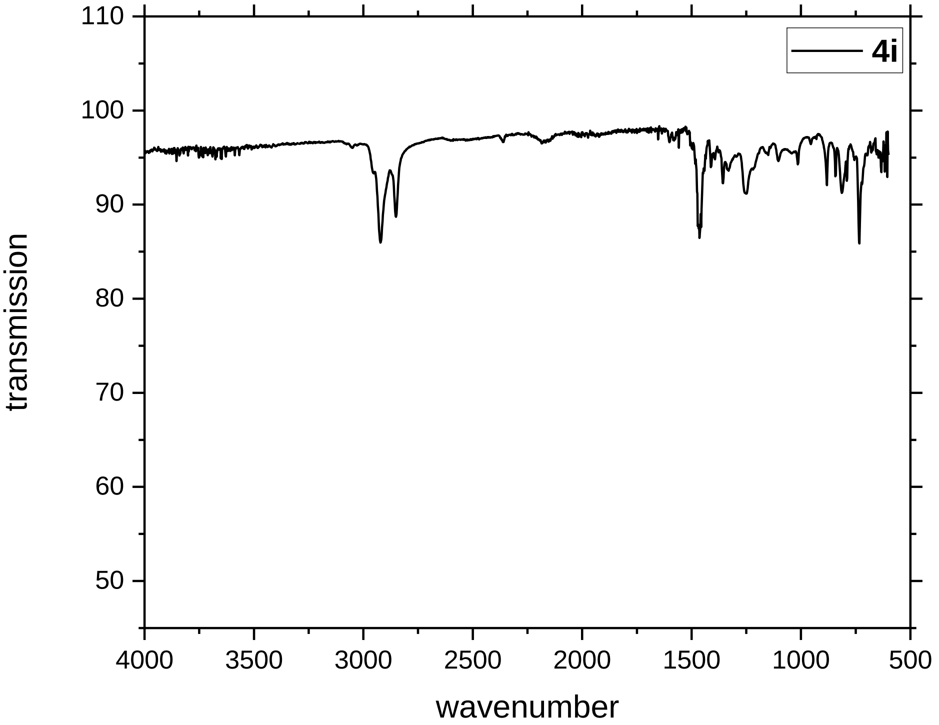


Figure S65. IR spectrum of polymer **4i**.

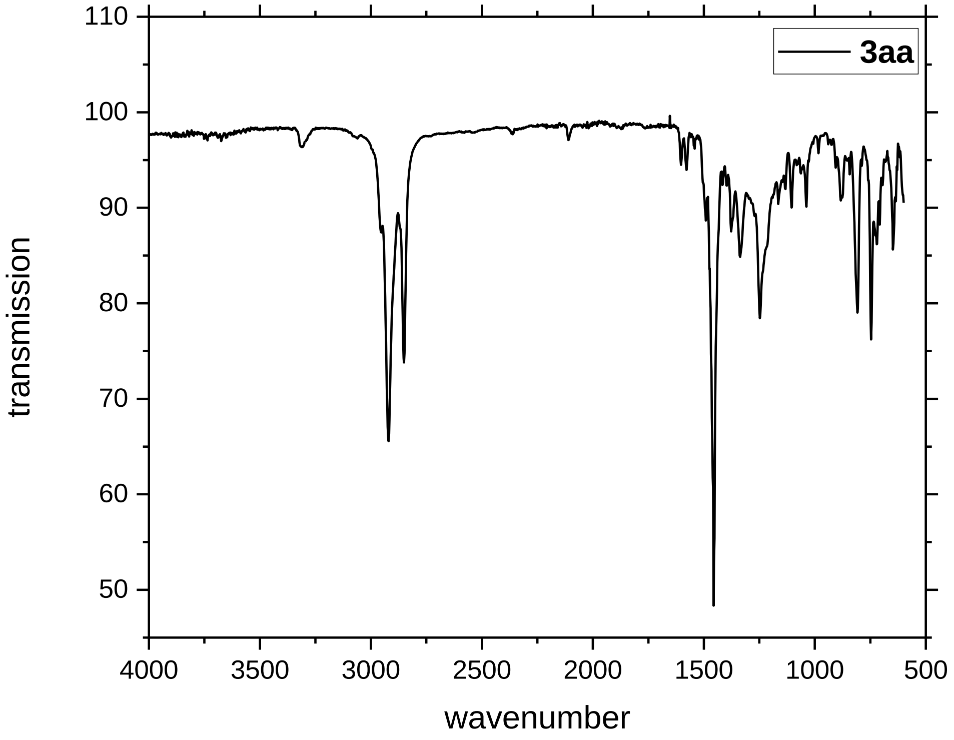


Figure S66. IR spectrum of monomer **3aa**.

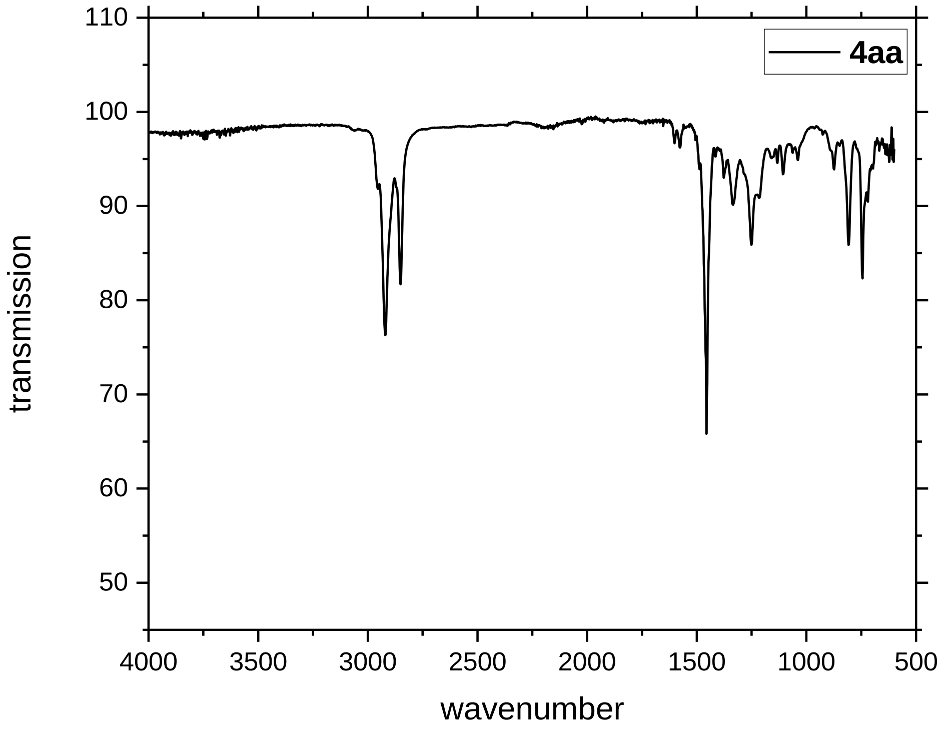


**Figure S67.** IR spectrum of polymer **4aa**.

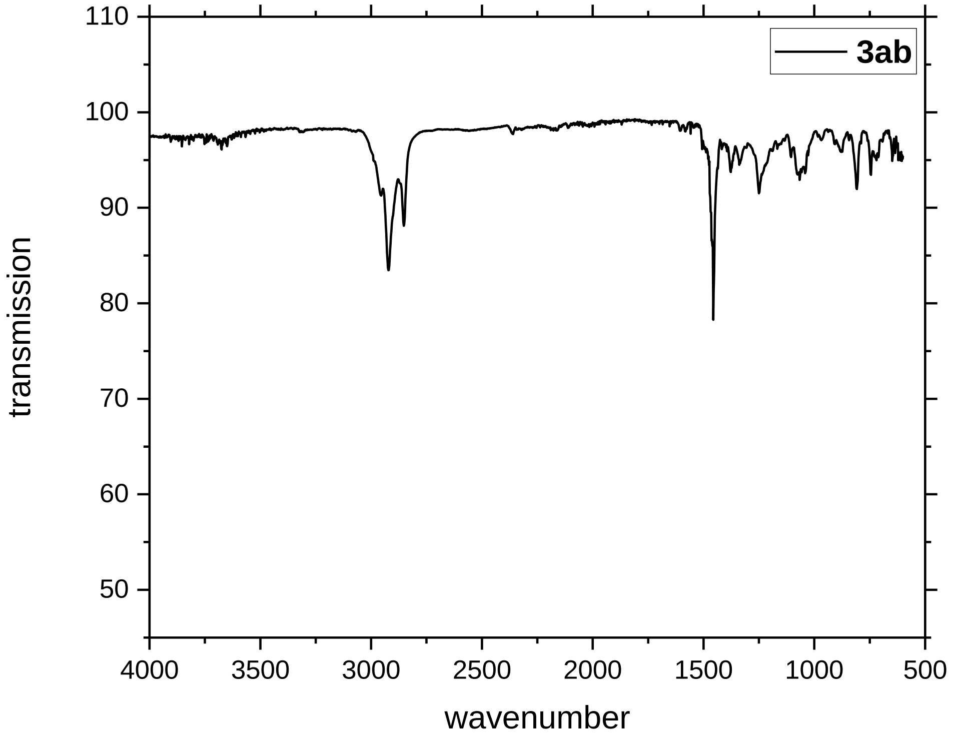


**Figure S68.** IR spectrum of monomer **3ab**.

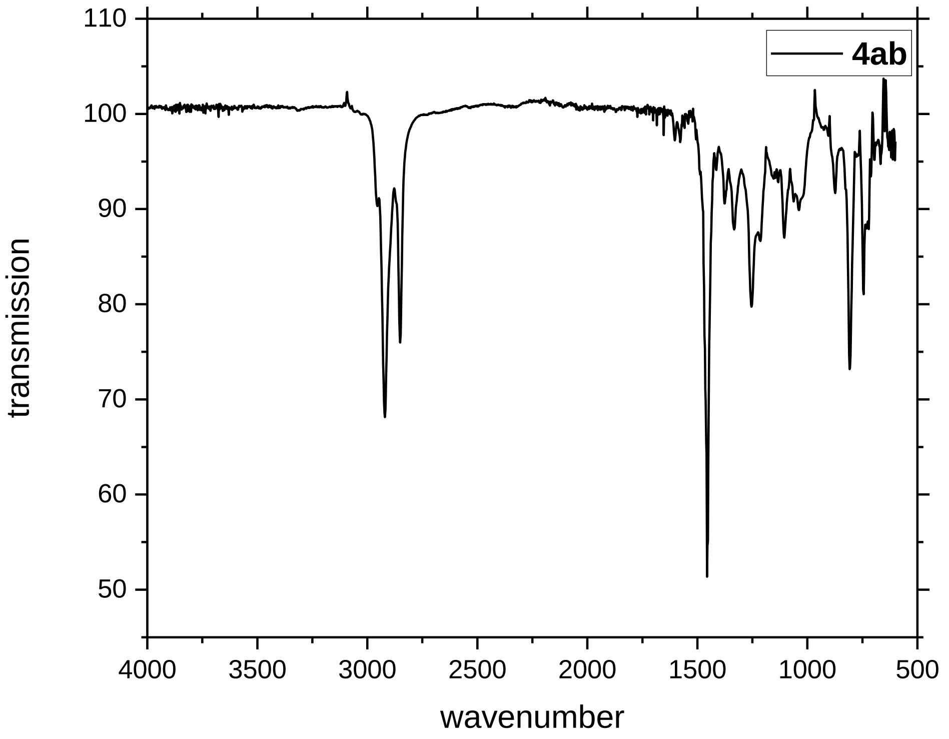


**Figure S69.** IR spectrum of polymer **4ab**.

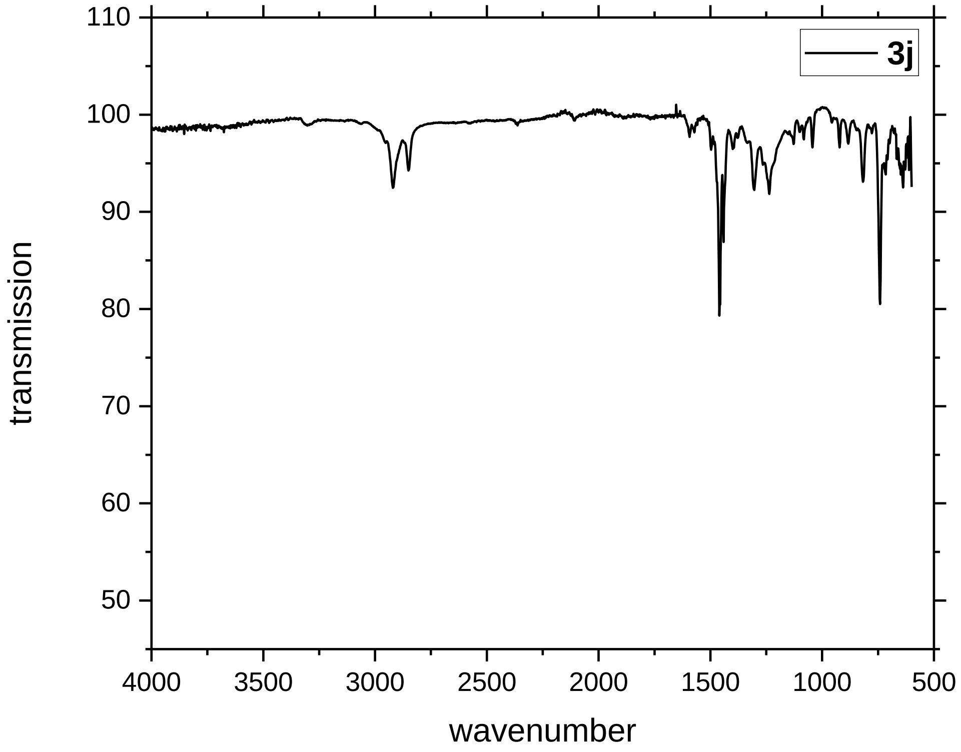


**Figure S70.** IR spectrum of monomer **3j**.

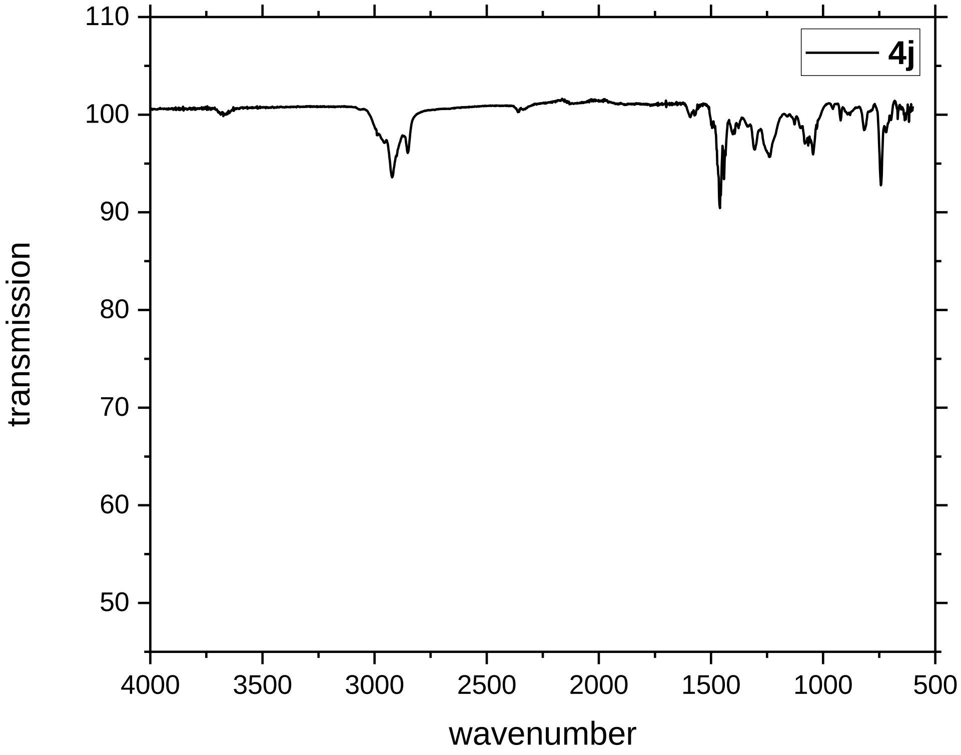


**Figure S71.** IR spectrum of polymer **4j**.

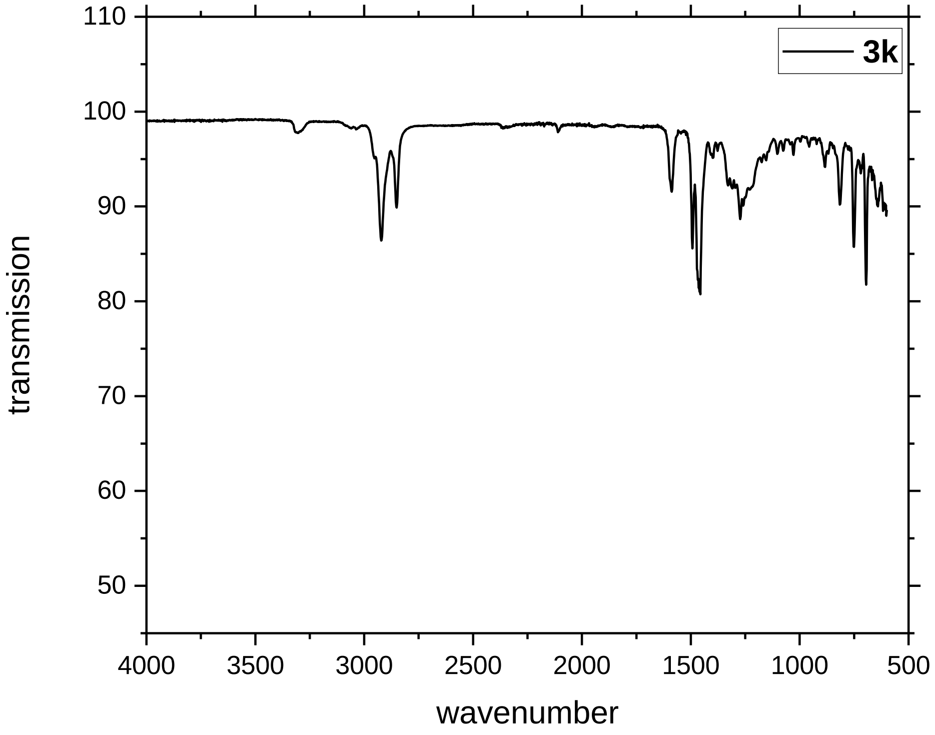


**Figure S72.** IR spectrum of monomer **3k**.

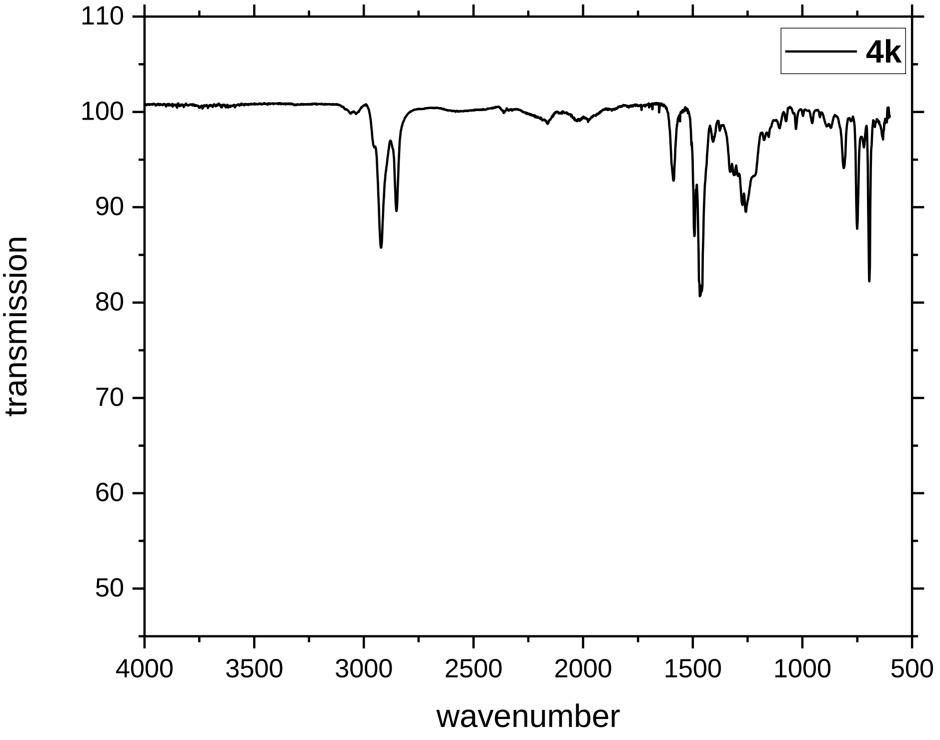


**Figure S73.** IR spectrum of polymer **4k**.

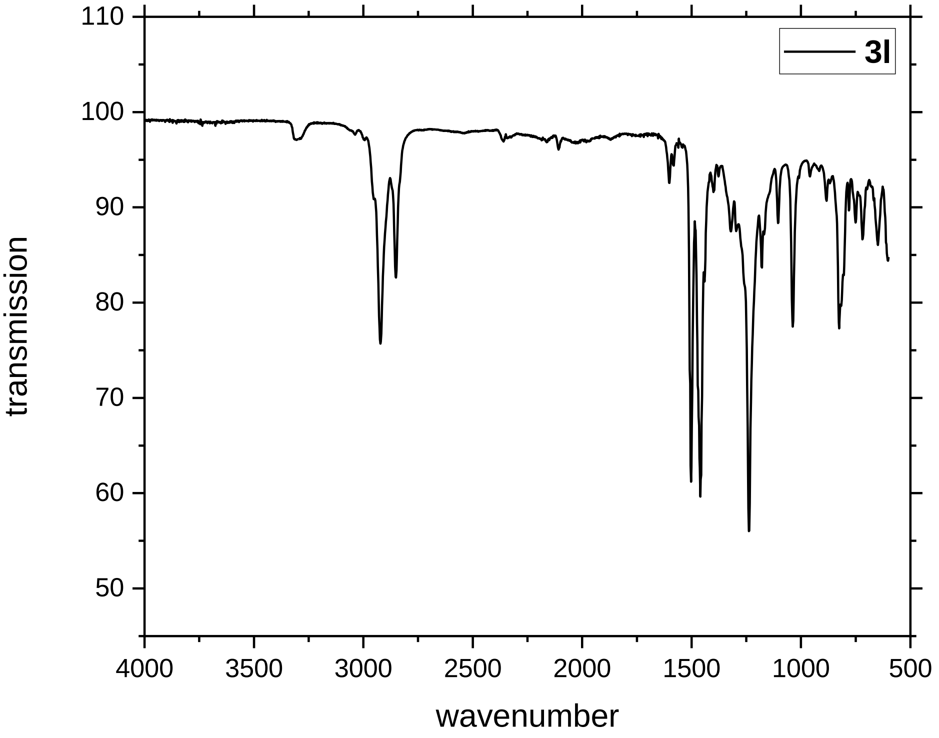


**Figure S74.** IR spectrum of monomer **3l**.

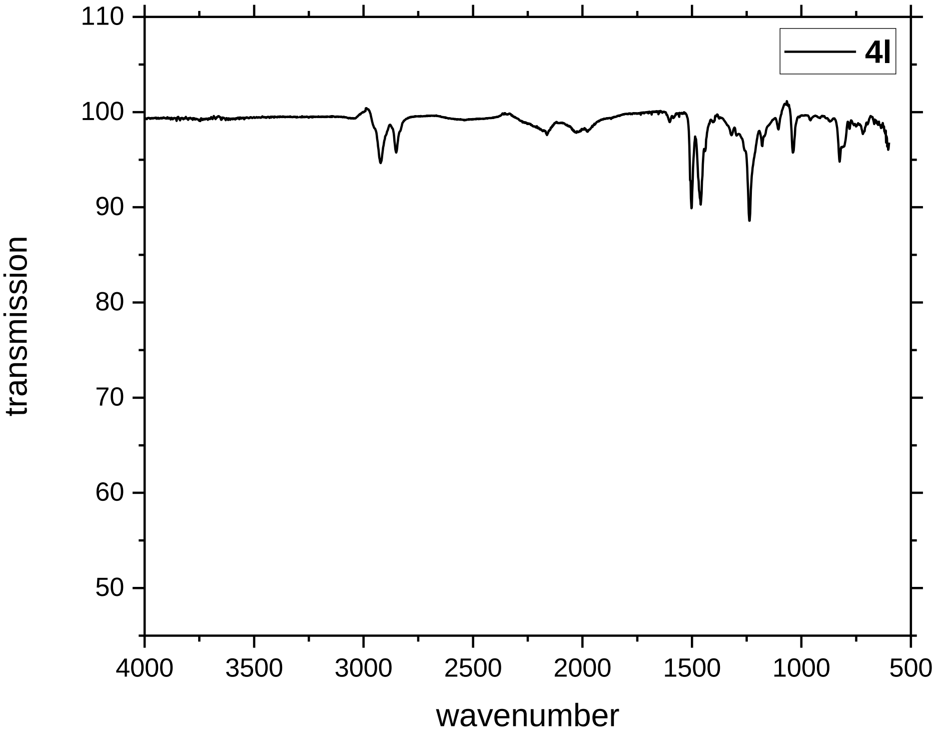


**Figure S75.** IR spectrum of polymer **4l**.

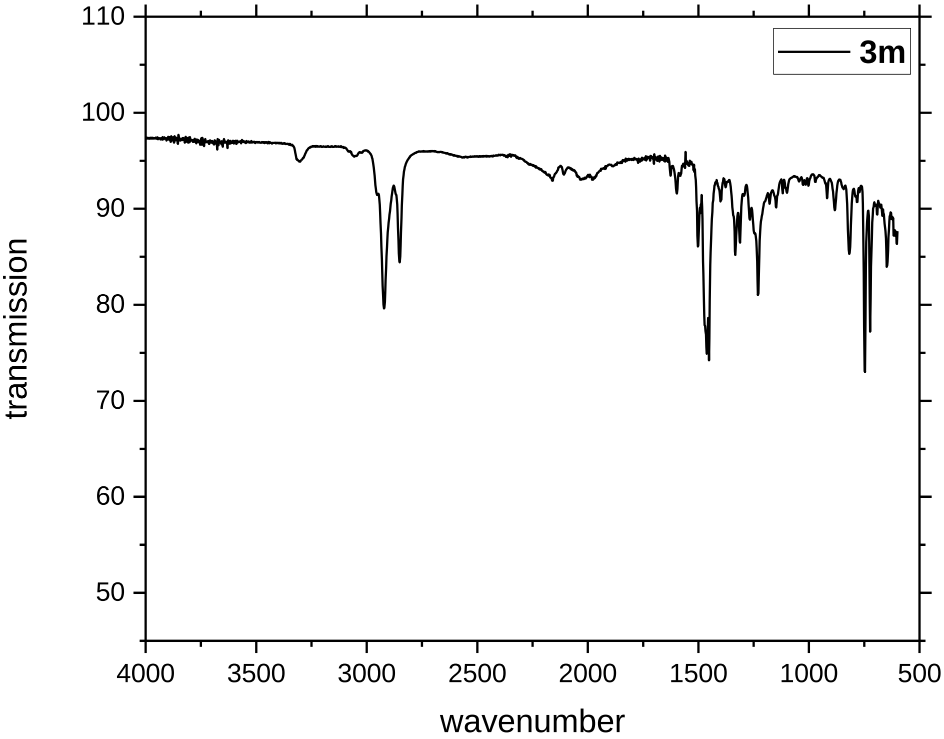


**Figure S76.** IR spectrum of monomer **3m**.

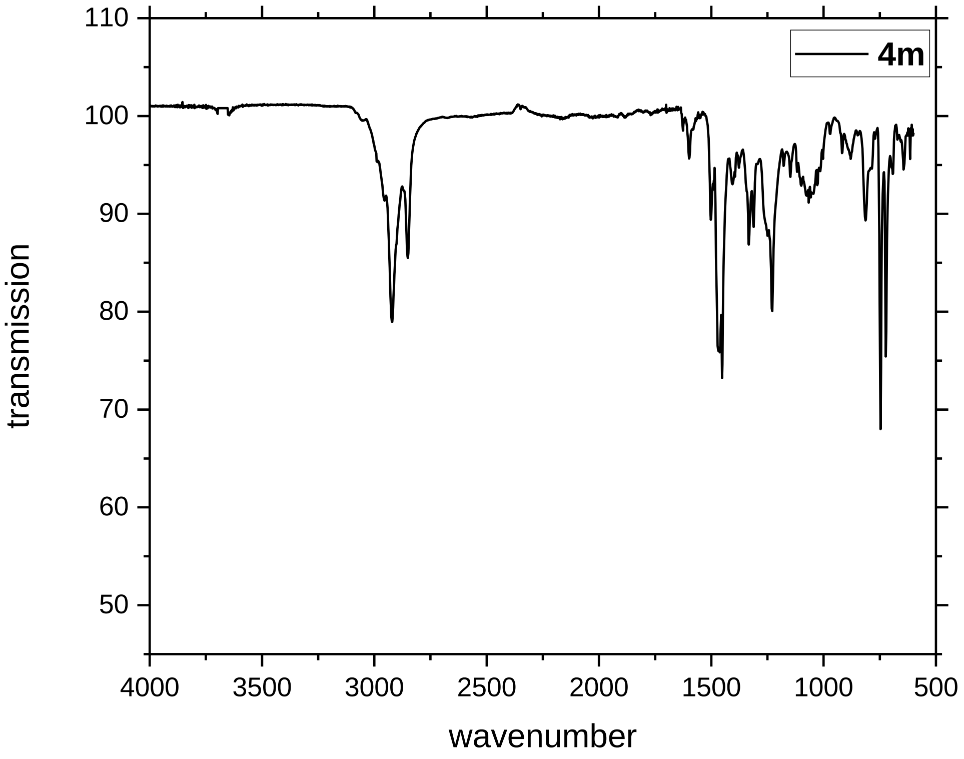


**Figure S77.** IR spectrum of polymer **4m**.

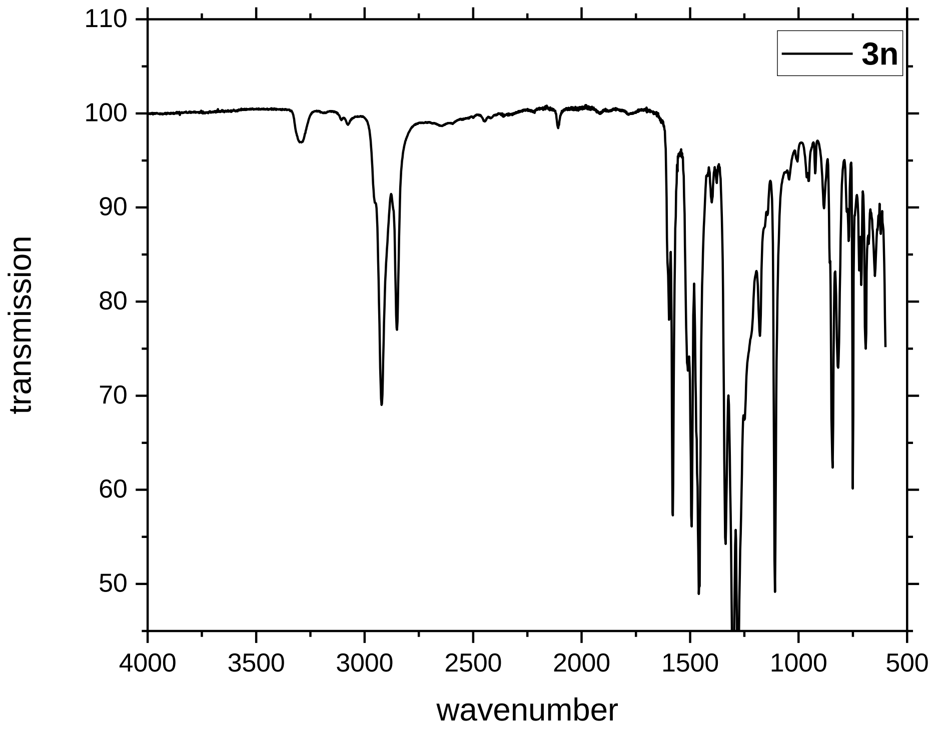


**Figure S78.** IR spectrum of monomer **3n**.

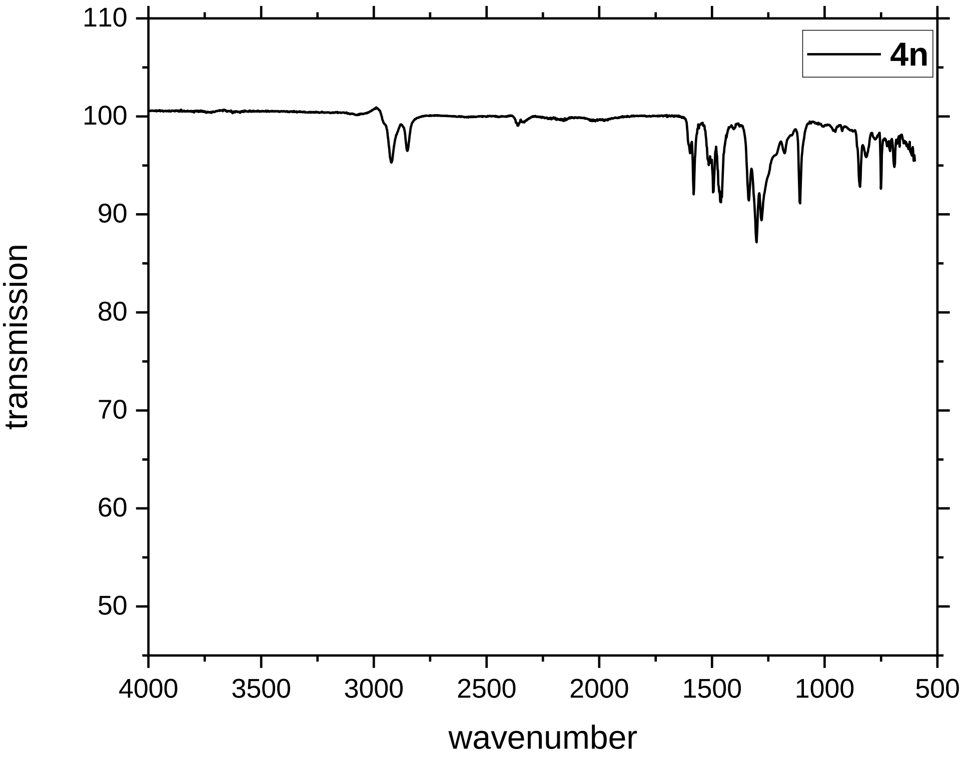


**Figure S79.** IR spectrum of polymer **4n**.

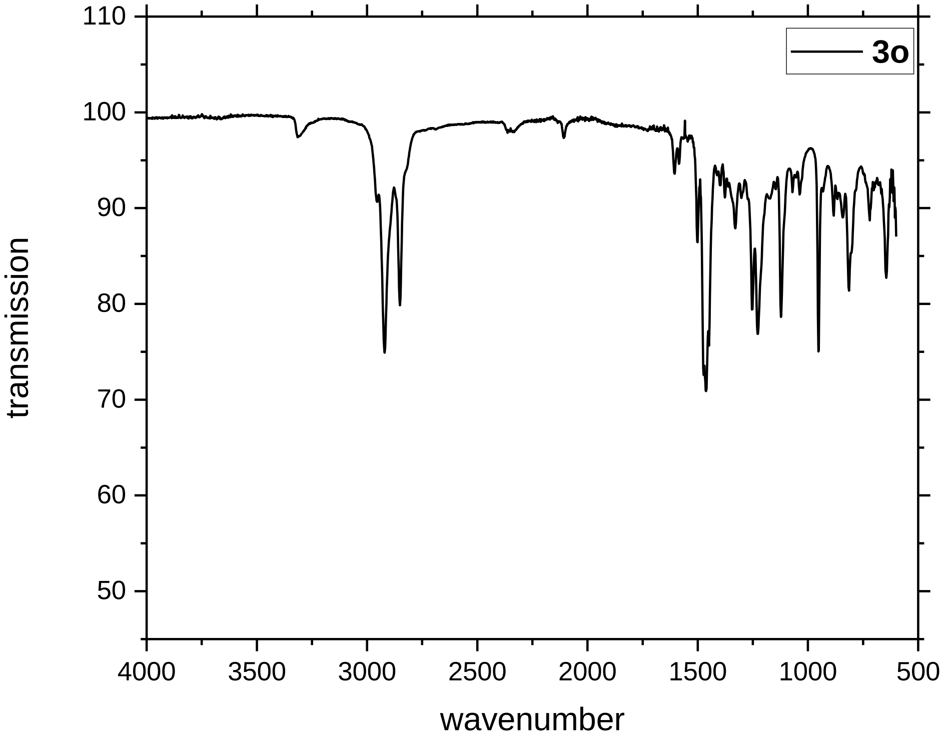


**Figure S80.** IR spectrum of monomer **3o**.

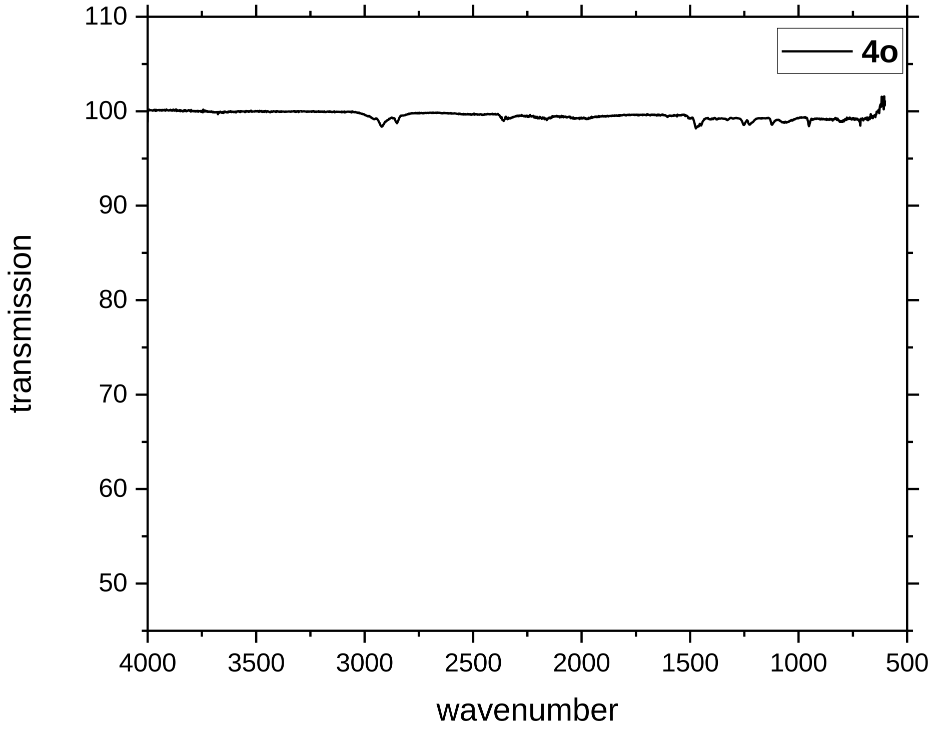


**Figure 81.** IR spectrum of polymer **4o**.

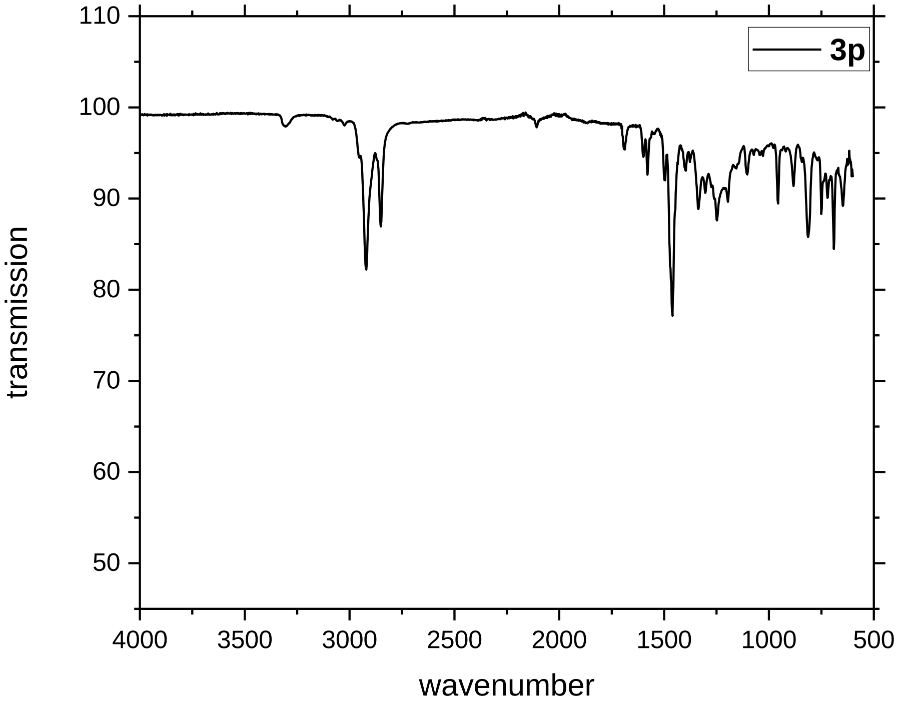


**Figure S82.** IR spectrum of monomer **3p**.

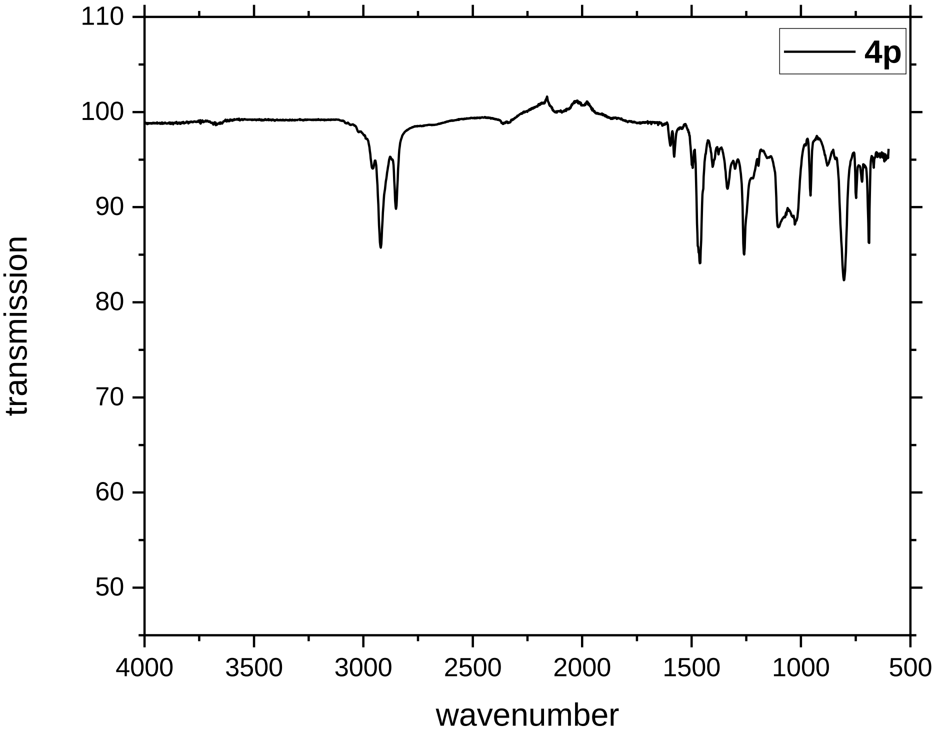


Figure S83. IR spectrum of polymer **4p**.

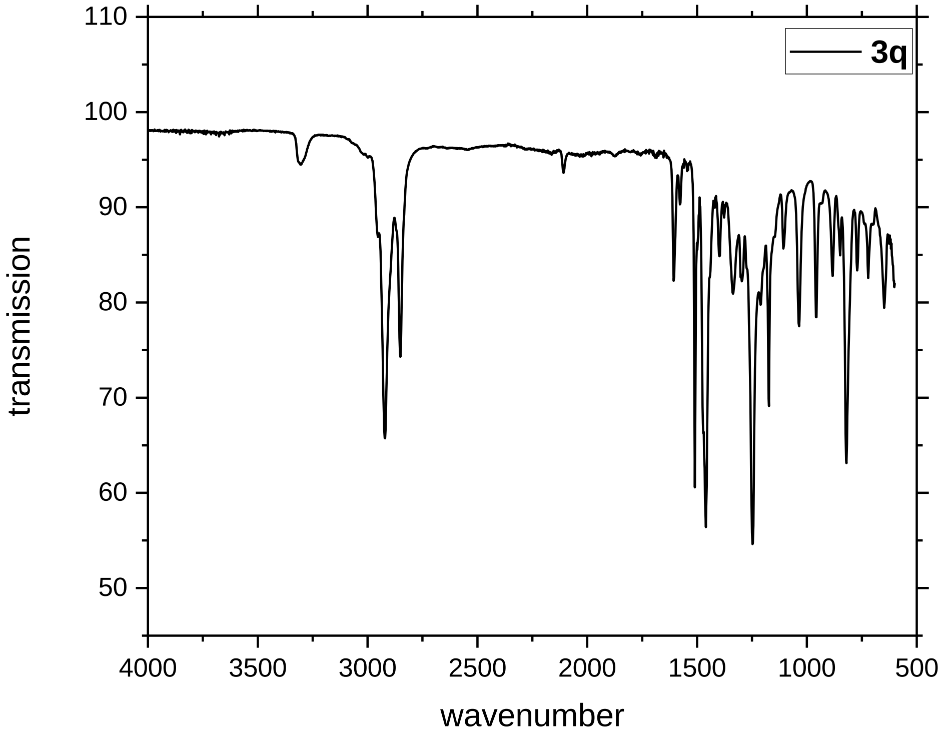


**Figure S84.** IR spectrum of monomer **3q**.

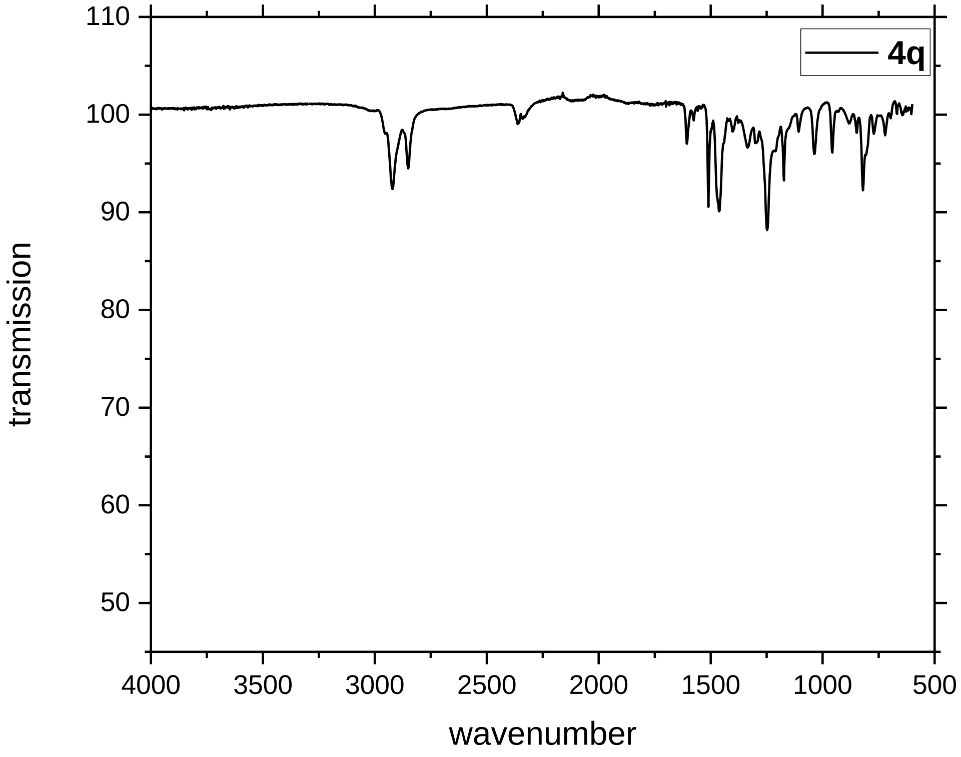


**Figure S85.** IR spectrum of polymer **4q**.

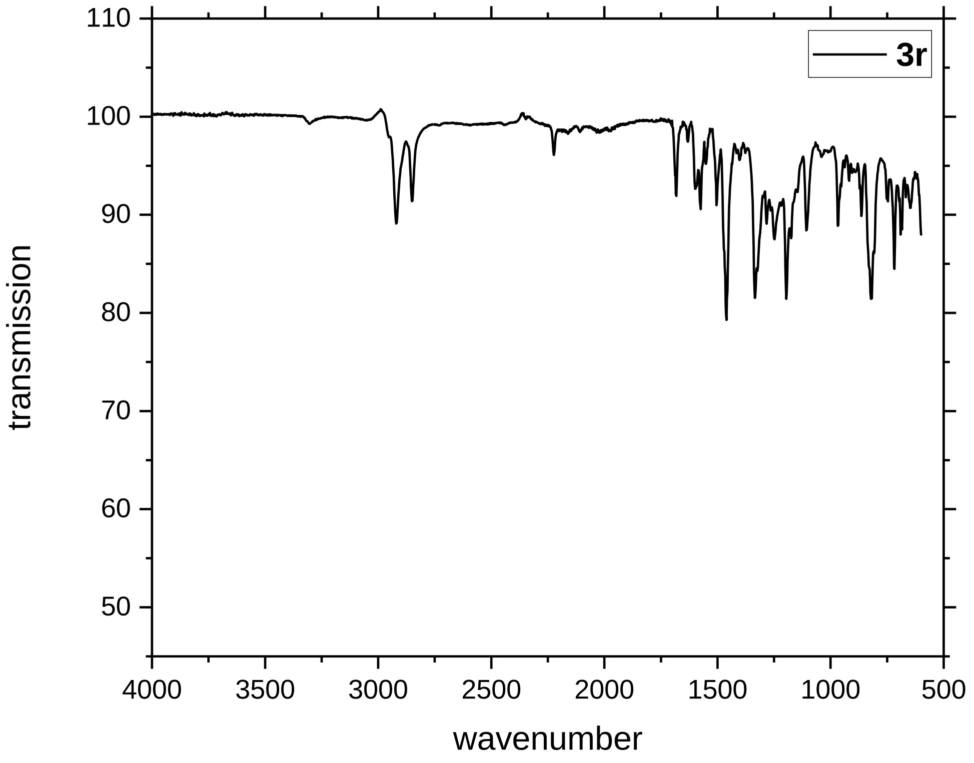


**Figure S86.** IR spectrum of monomer **3r**.

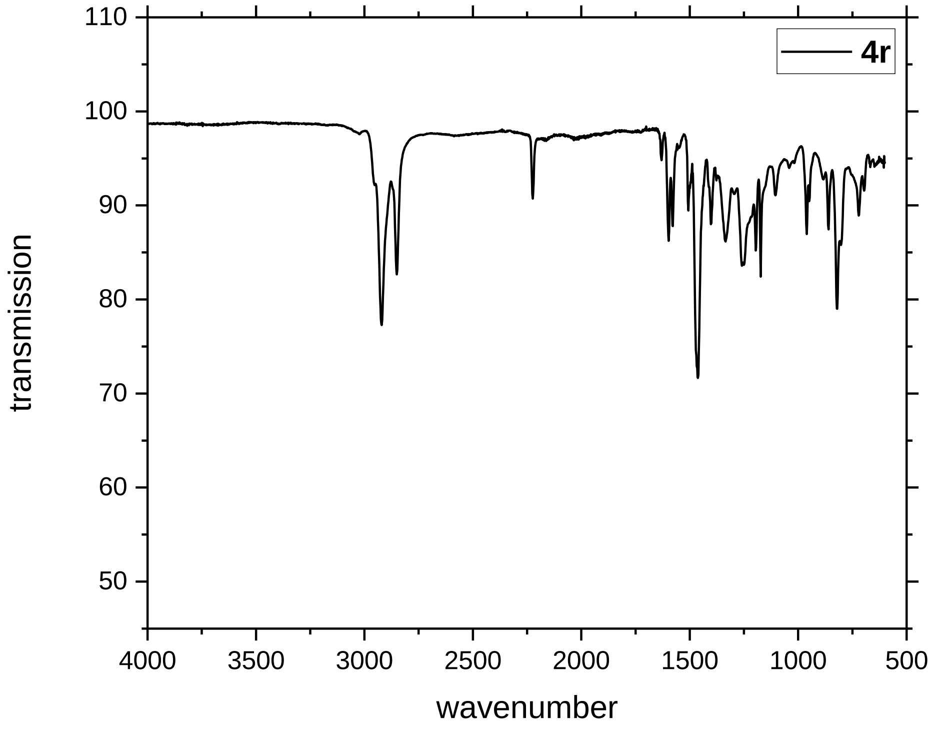


**Figure S87.** IR spectrum of polymer **4r**.

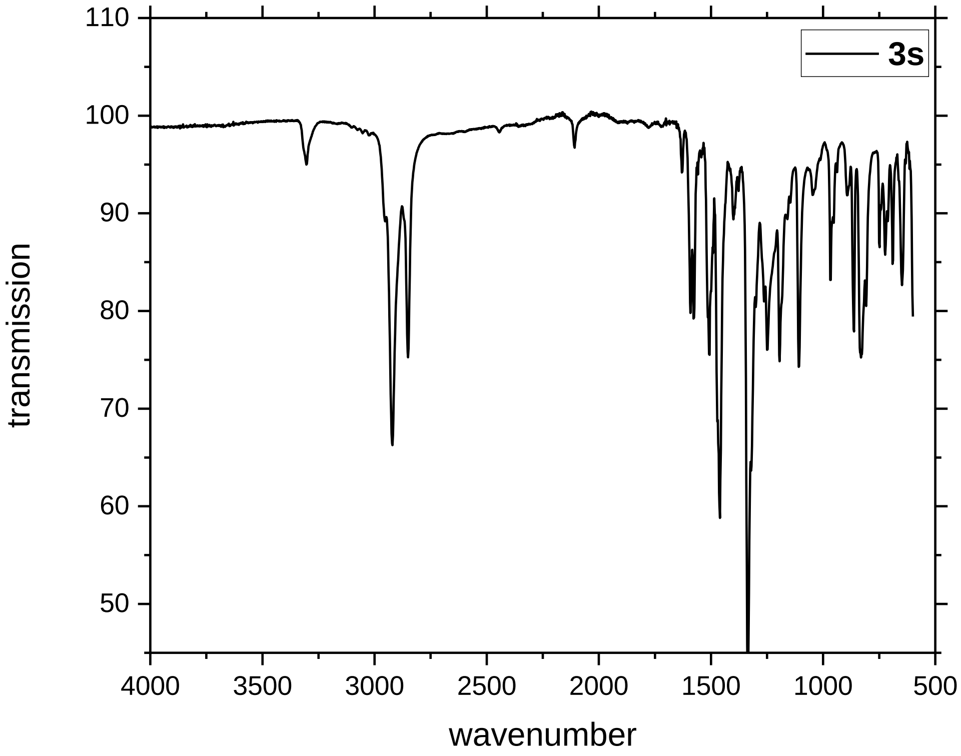


**Figure S88.** IR spectrum of monomer **3s**.

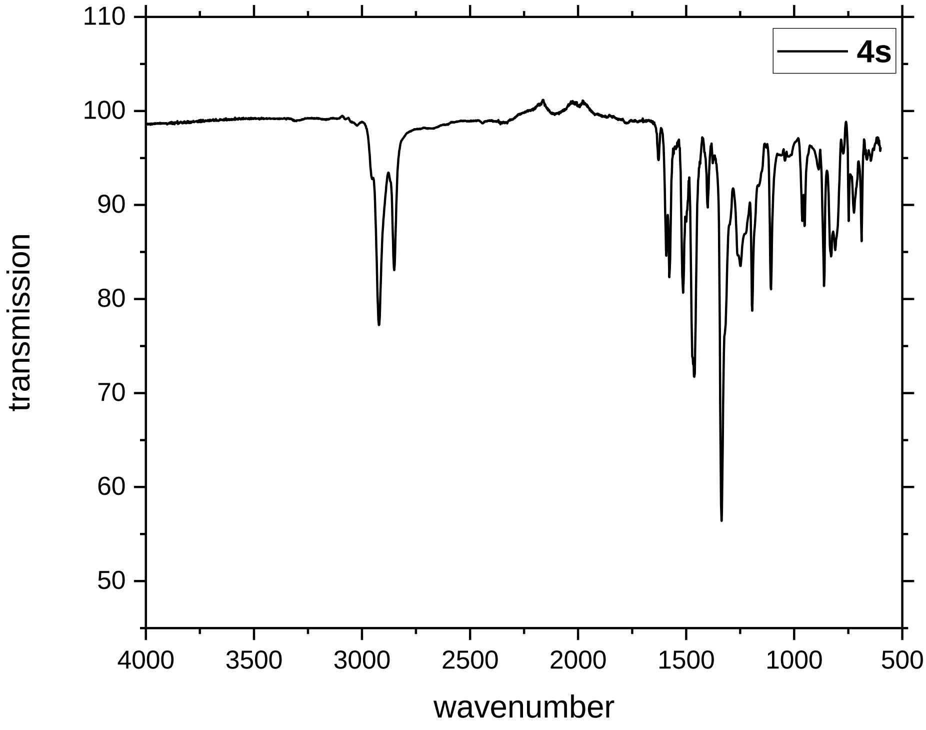


**Figure S89.** IR spectrum of polymer **4s**.

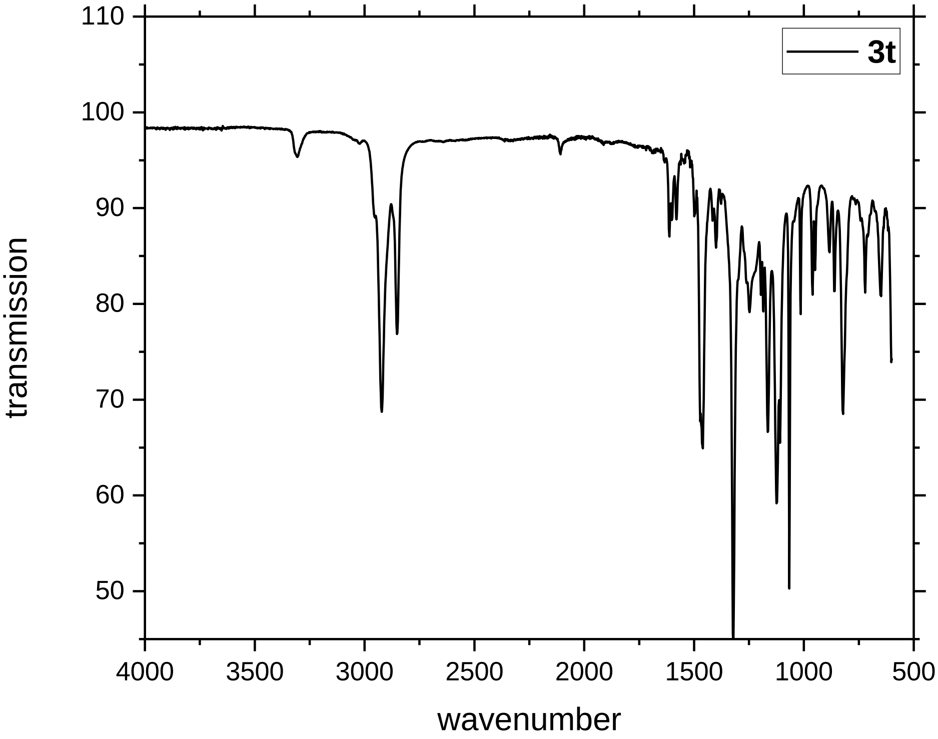


**Figure S90.** IR spectrum of monomer **3t**.

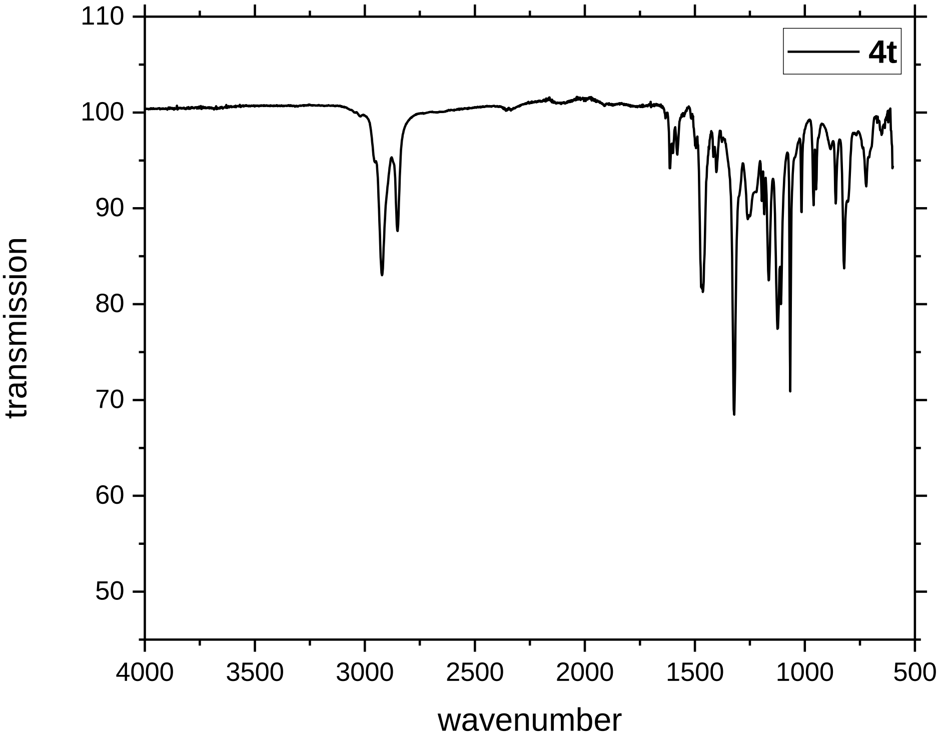


**Figure S91.** IR spectrum of polymer **4t**.

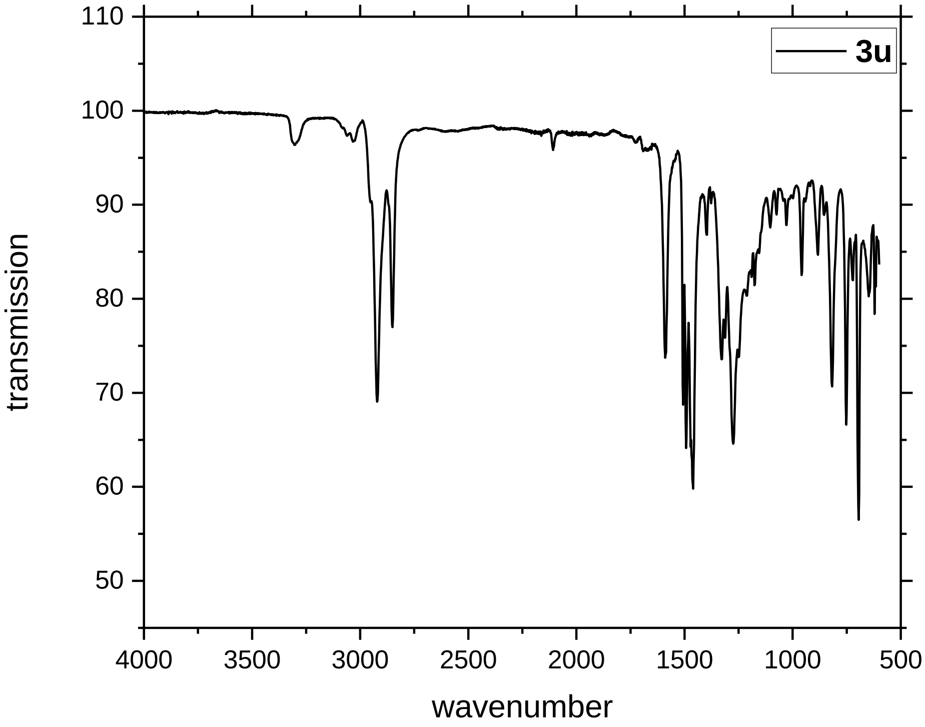


**Figure S92.** IR spectrum of monomer **3u**.

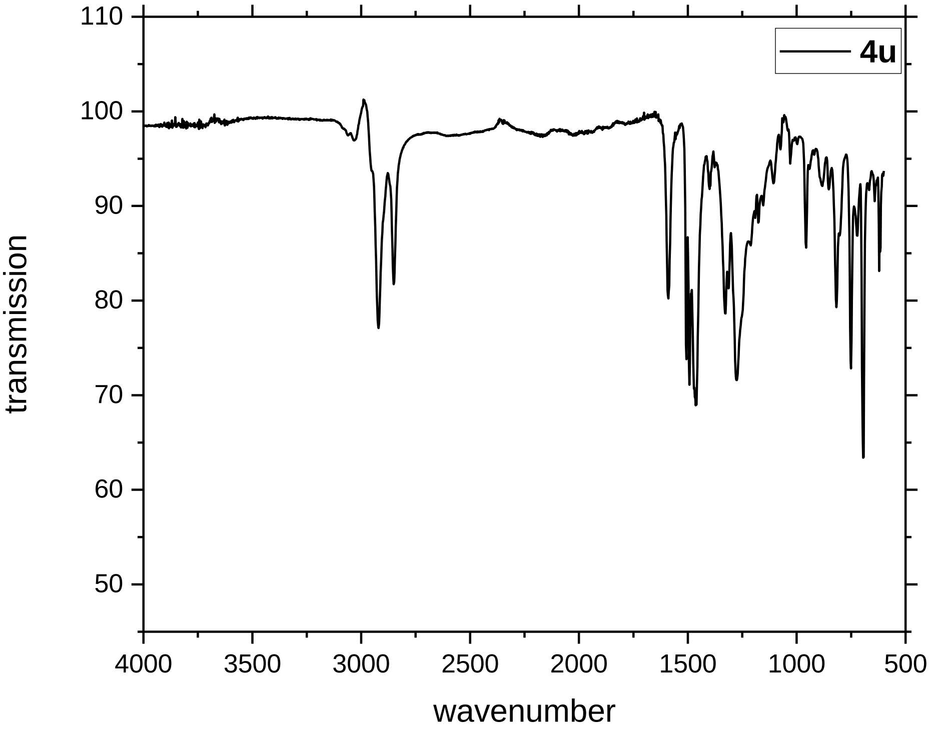


**Figure S93.** IR spectrum of polymer **4u**.

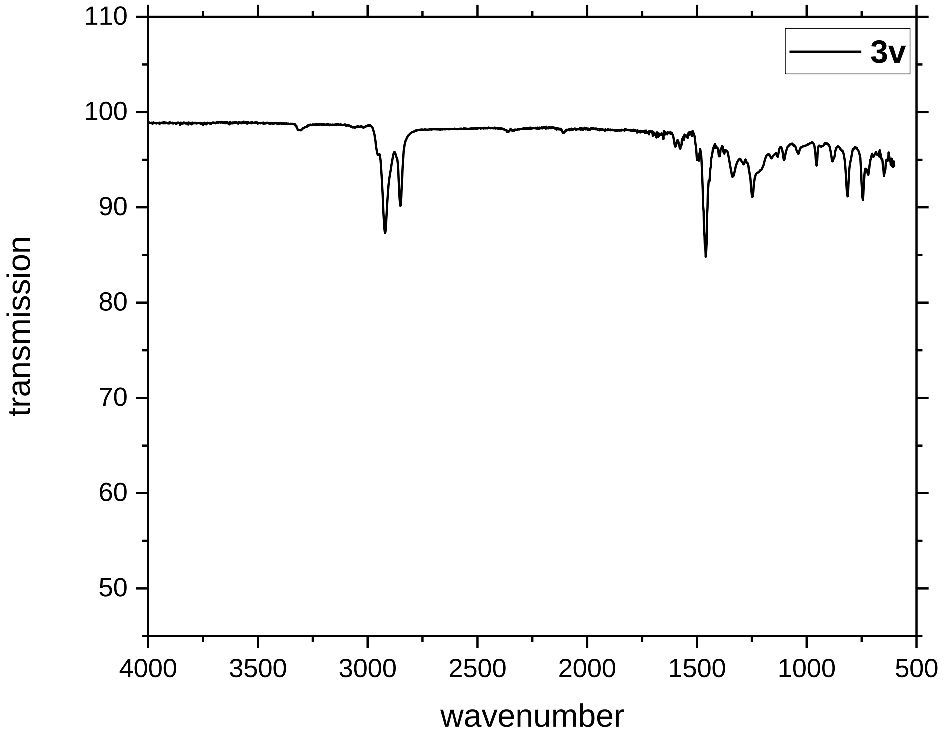


**Figure S94.** IR spectrum of monomer **3v**.

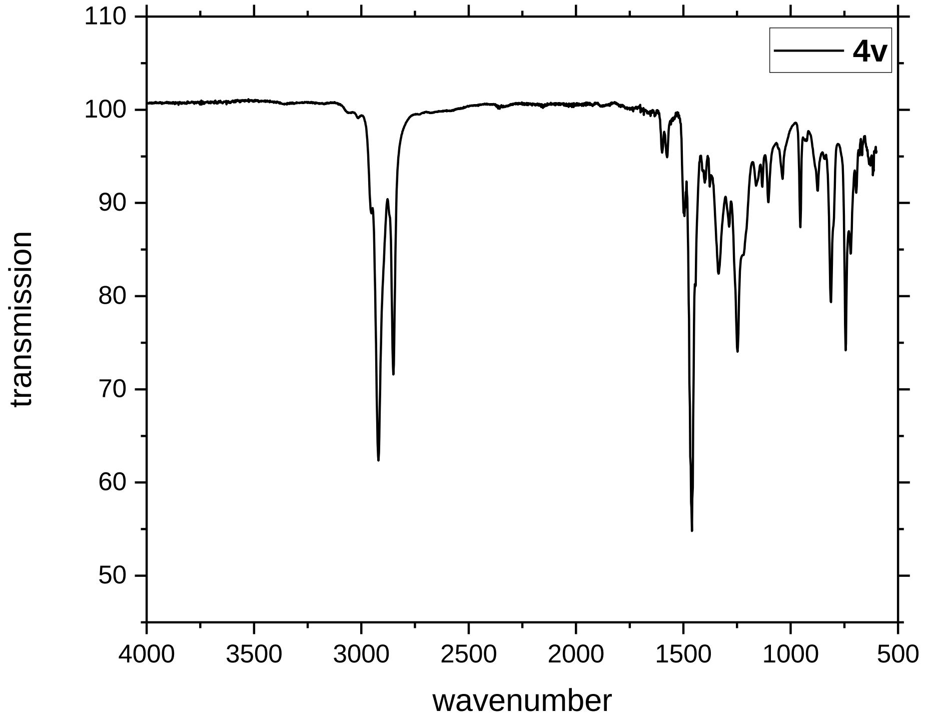


**Figure S95.** IR spectrum of polymer **4v**.

## 5.0 GPC traces of polymers 4

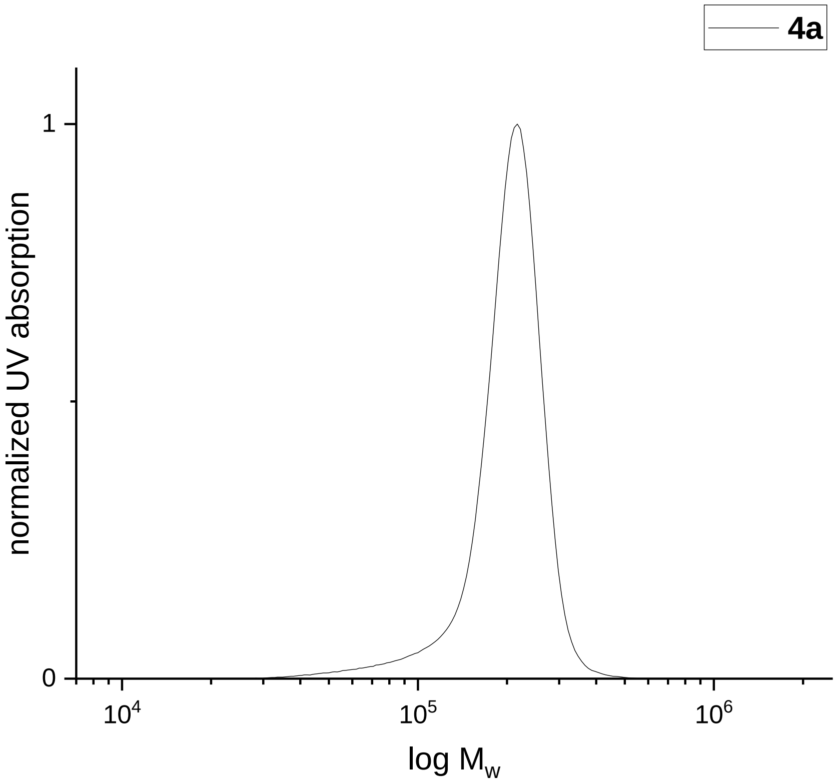


**Figure S96.** GPC trace of polymer **4a** (eluent THF, flow rate 1 mL/min, $T$ = 293 K).

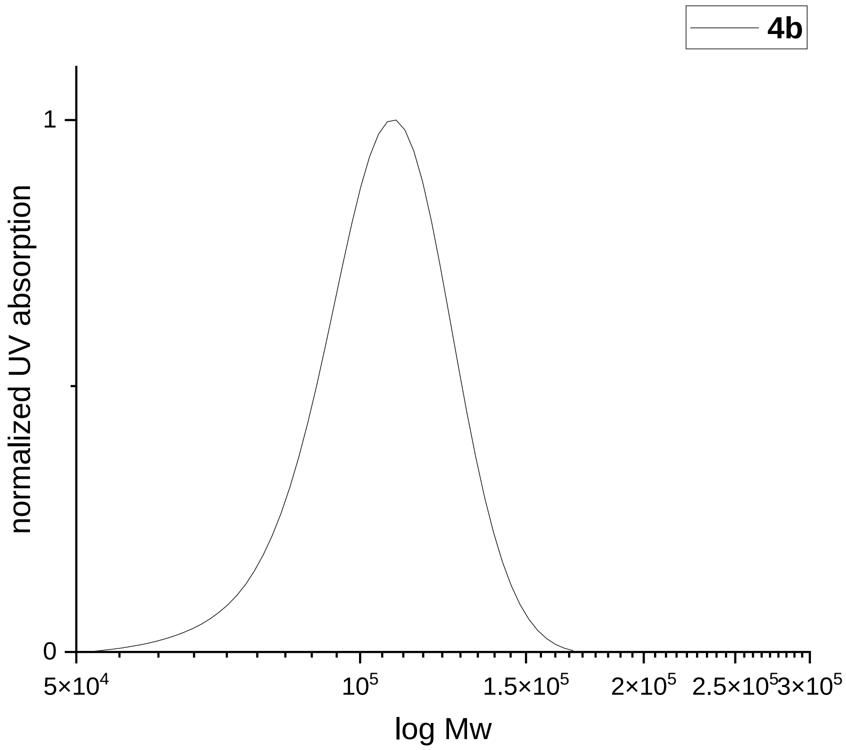


**Figure S97.** GPC trace of polymer **4b** (eluent THF, flow rate 1 mL/min, $T$ = 293 K).

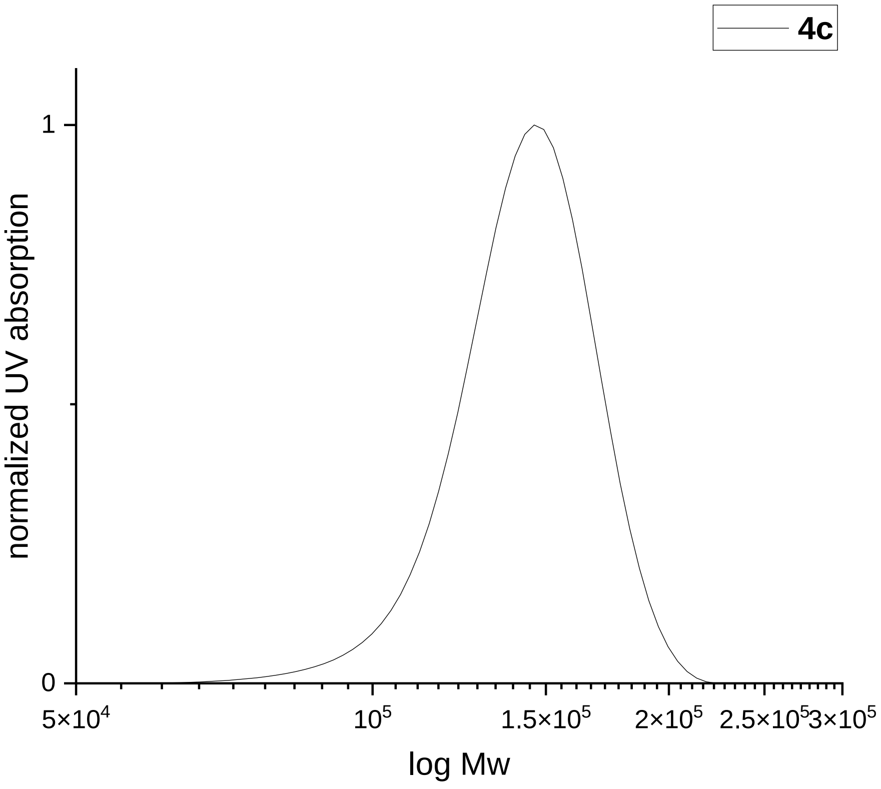


**Figure S98.** GPC trace of polymer **4c** (eluent THF, flow rate 1 mL/min, $T$ = 293 K).

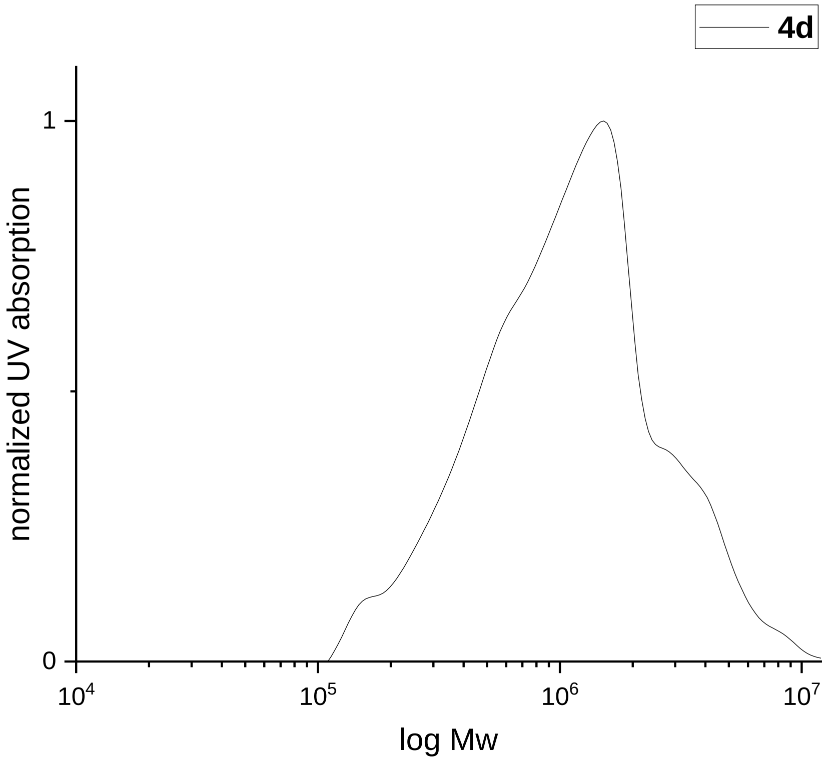


**Figure S99.** GPC trace of polymer **4d** (eluent THF, flow rate 1 mL/min, $T$ = 293 K).

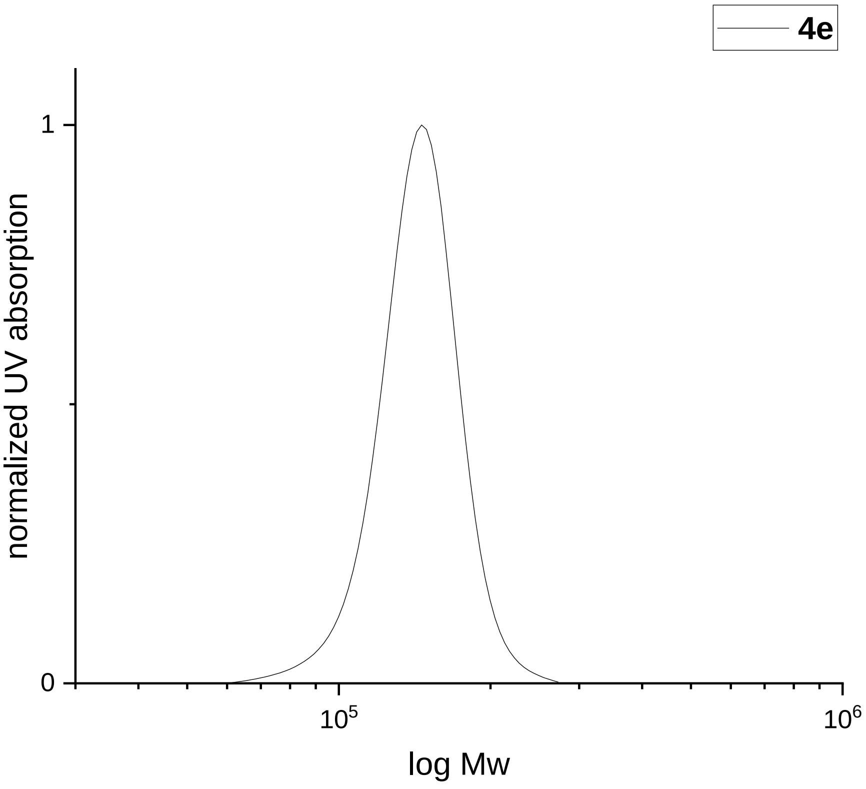


**Figure S100.** GPC trace of polymer **4e** (eluent THF, flow rate 1 mL/min, *T* = 293 K).

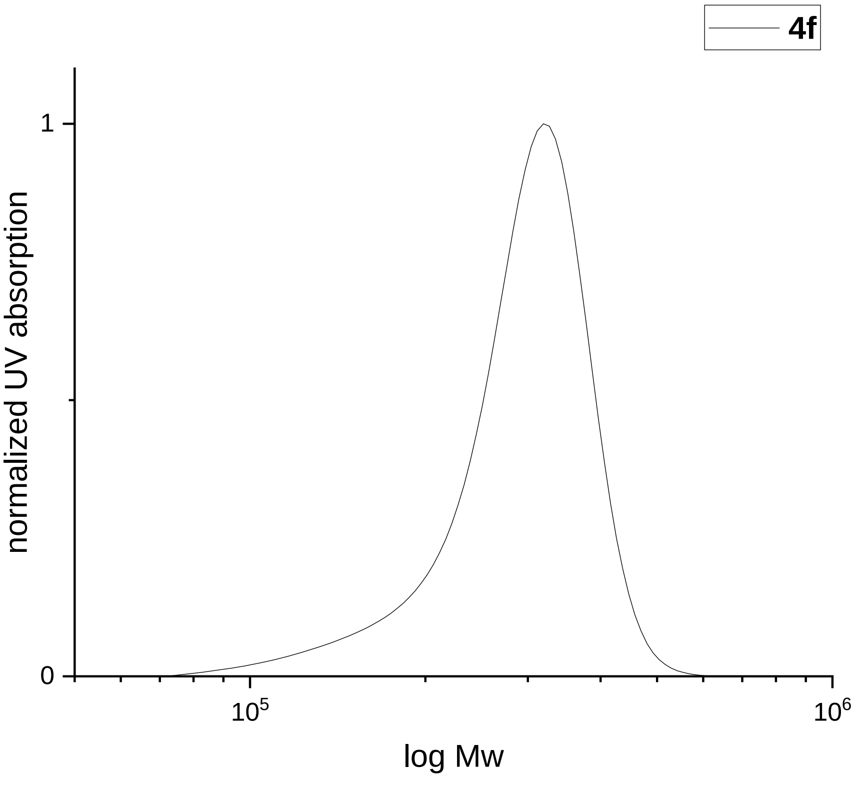


**Figure S101.** GPC trace of polymer **4f** (eluent THF, flow rate 1 mL/min, *T* = 293 K).

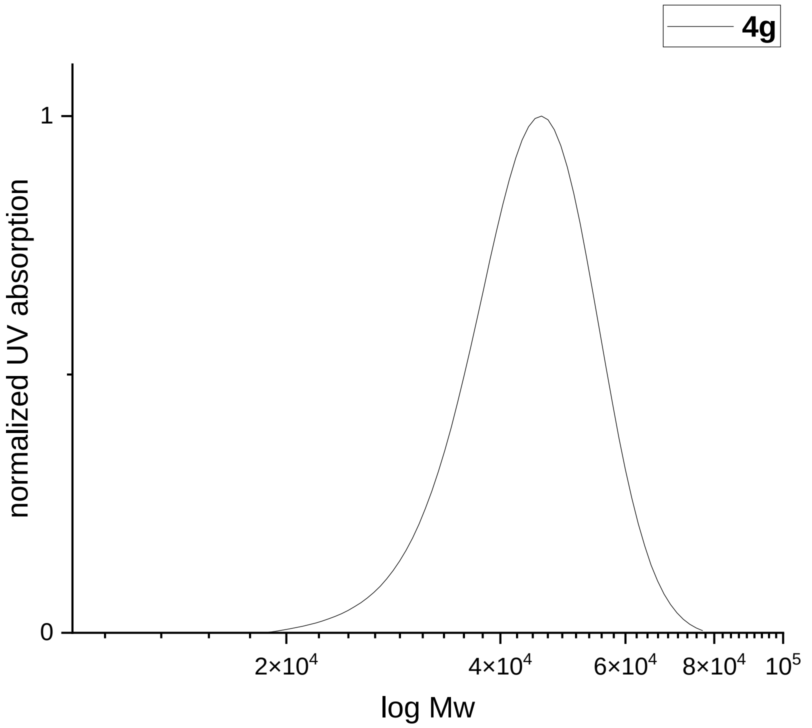


**Figure S102.** GPC trace of polymer **4g** (eluent THF, flow rate 1 mL/min, *T* = 293 K).

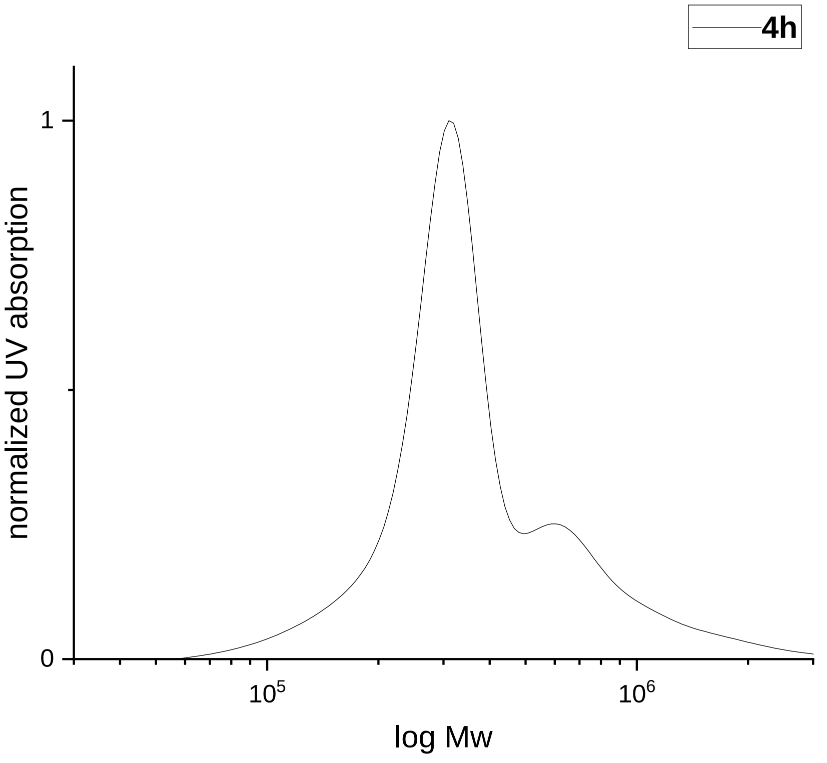


**Figure S103.** GPC trace of polymer **4h** (eluent THF, flow rate 1 mL/min, *T* = 293 K).

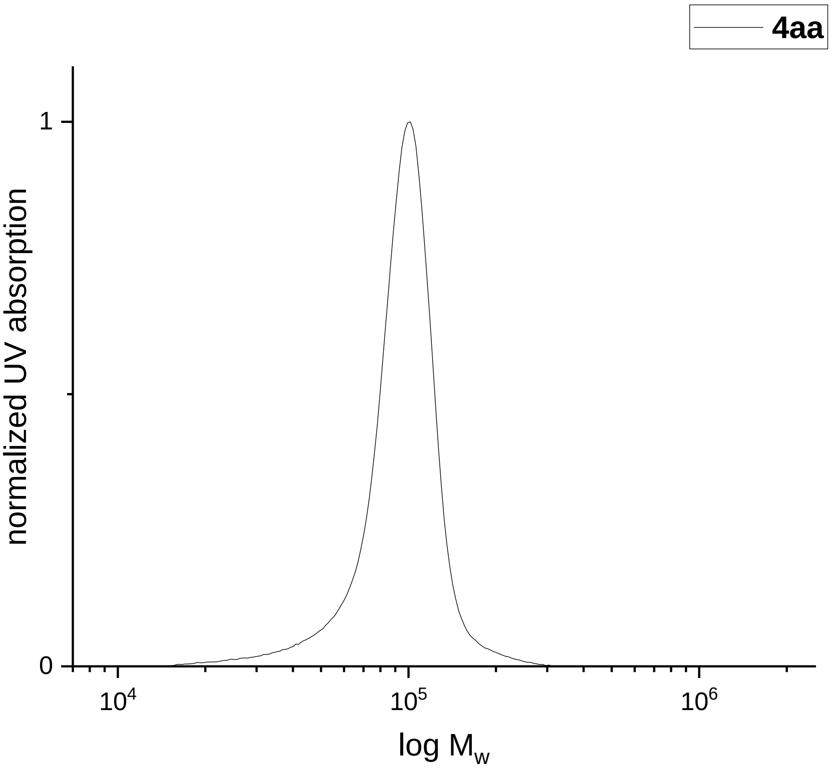


**Figure S104.** GPC trace of polymer **4aa** (eluent THF, flow rate 1 mL/min, *T* = 293 K).

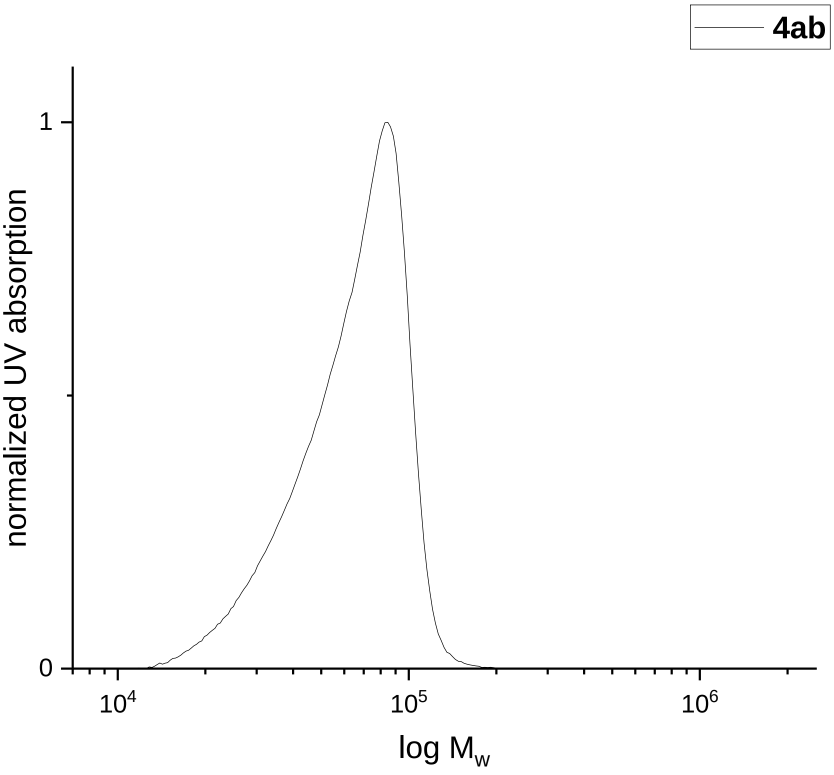


**Figure S105.** GPC trace of polymer **4ab** (eluent THF, flow rate 1 mL/min, T = 293 K).

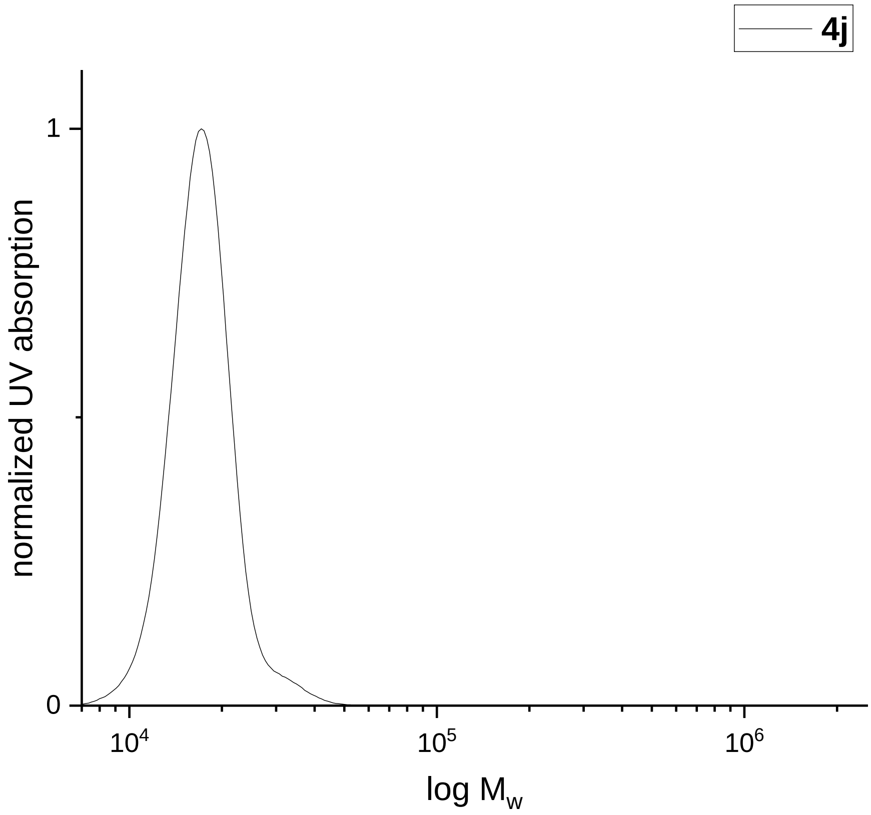


**Figure S106.** GPC trace of polymer **4j** (eluent THF, flow rate 1 mL/min, T = 293 K).

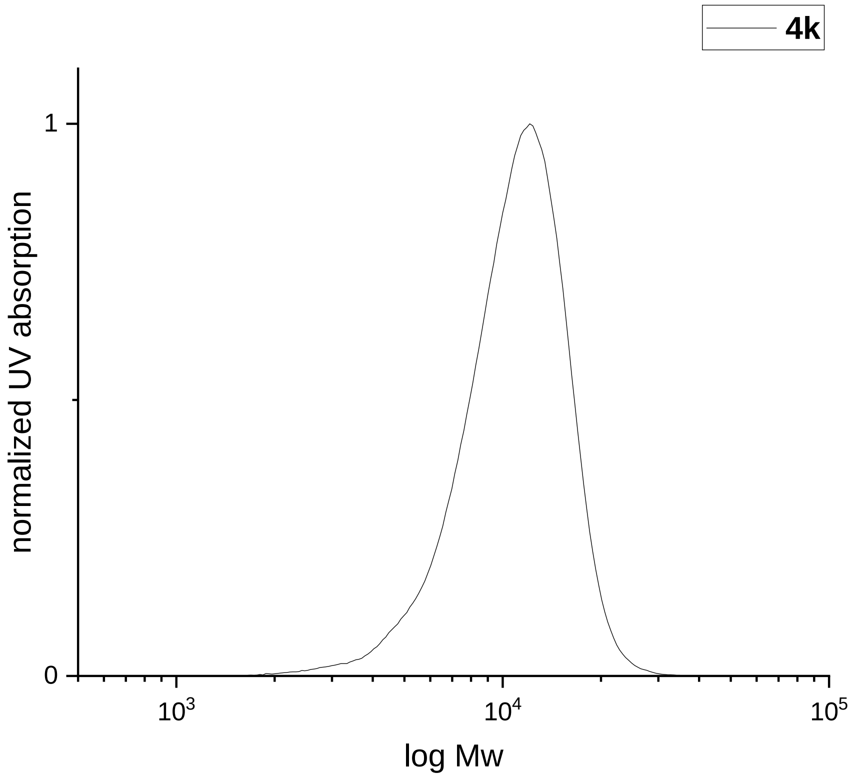


**Figure S107.** GPC trace of polymer **4k** (eluent THF, flow rate 1 mL/min, T = 293 K).

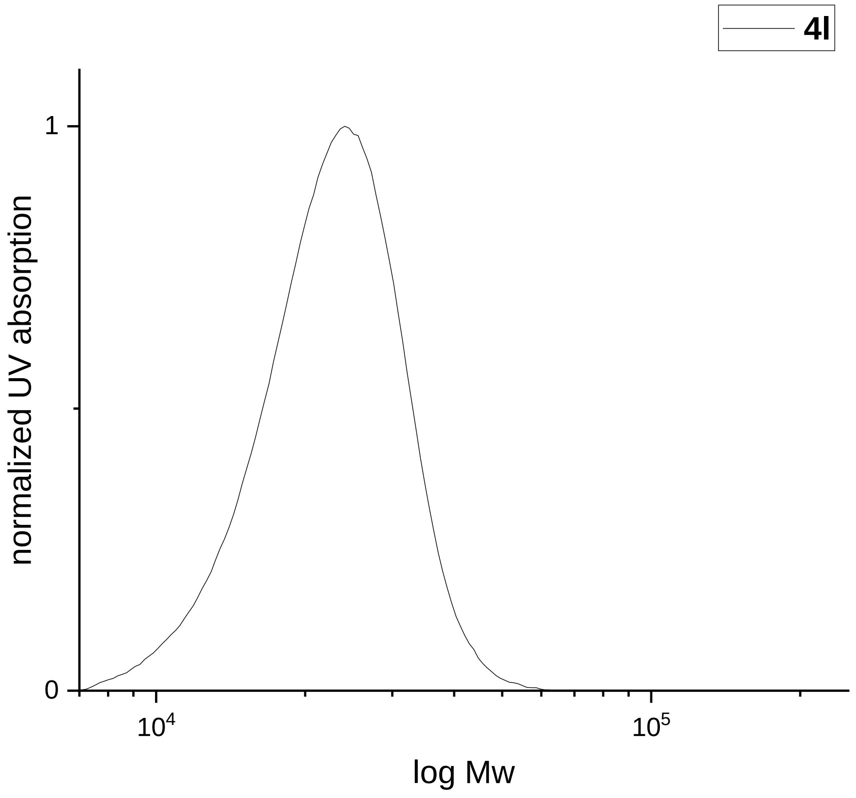


**Figure S108.** GPC trace of polymer **4l** (eluent THF, flow rate 1 mL/min, T = 293 K).

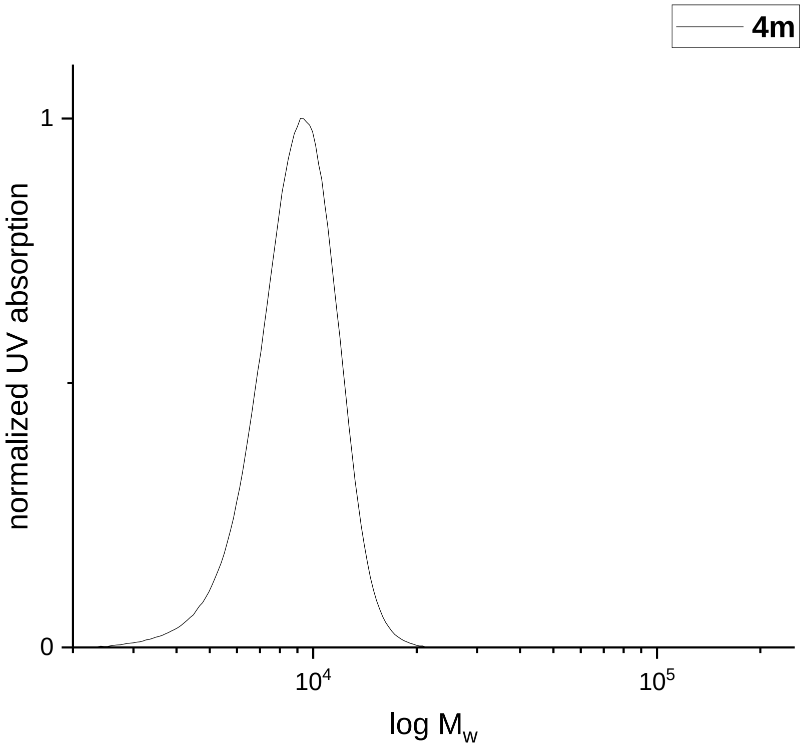


**Figure S109.** GPC trace of polymer **4m** (eluent THF, flow rate 1 mL/min, T = 293 K).

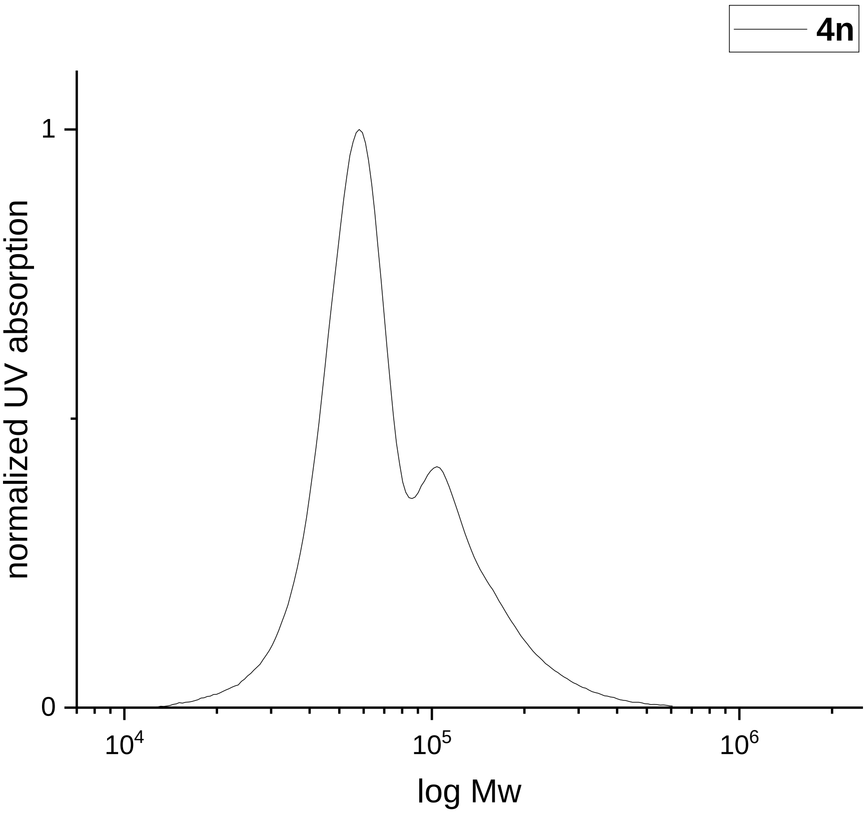


**Figure S110.** GPC trace of polymer **4n** (eluent THF, flow rate 1 mL/min, T = 293 K).

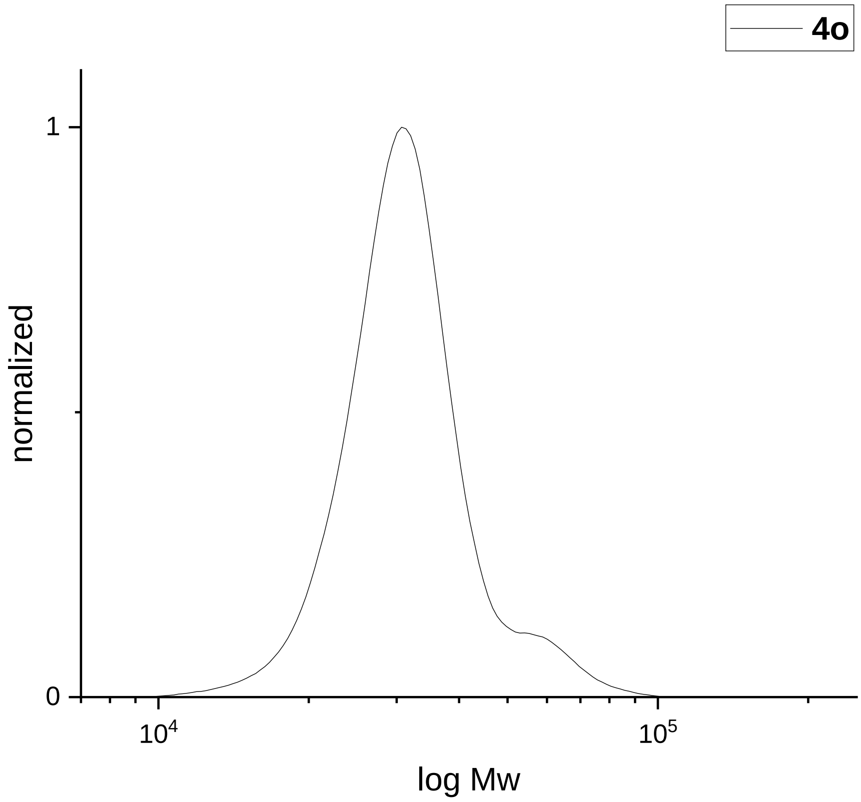


**Figure S111.** GPC trace of polymer **4o** (eluent THF, flow rate 1 mL/min, T = 293 K).

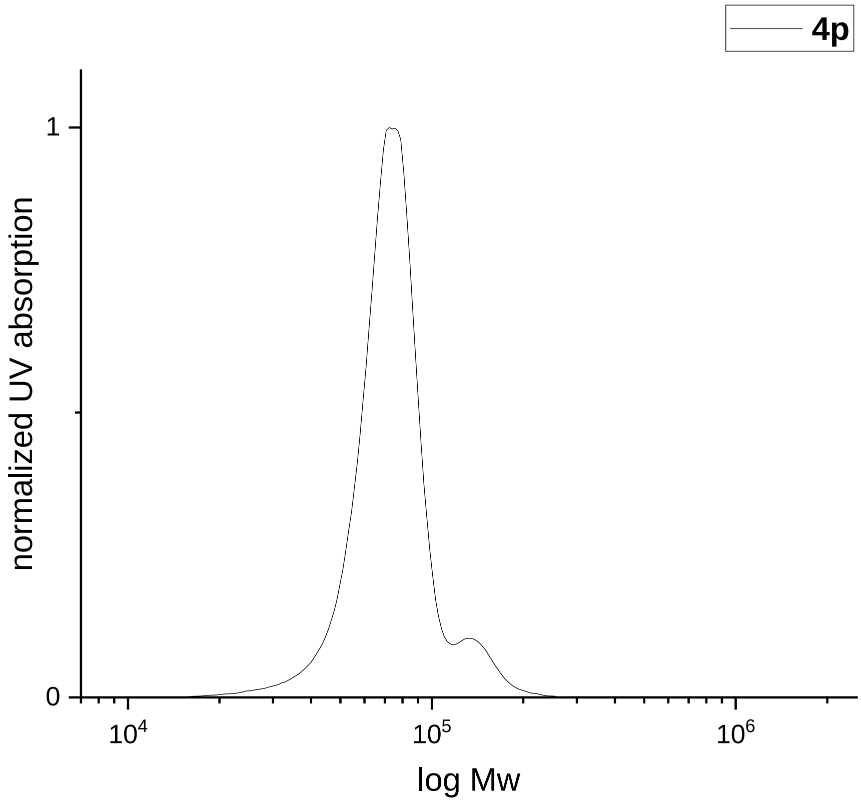


**Figure S112.** GPC trace of polymer **4p** (eluent THF, flow rate 1 mL/min, T = 293 K).

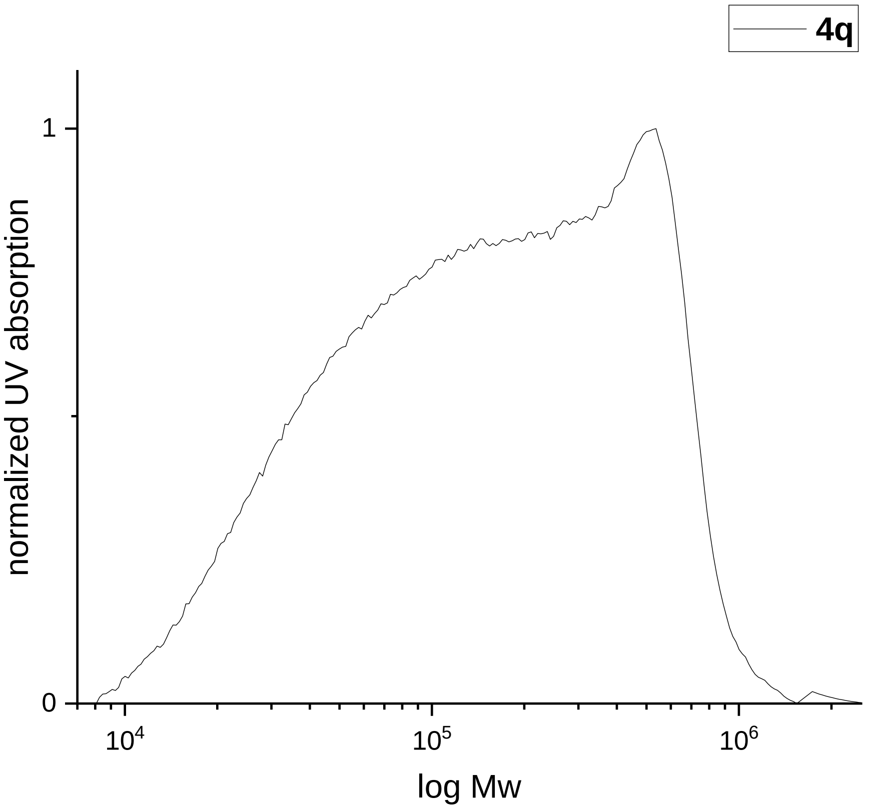


**Figure S113.** GPC trace of polymer **4q** (eluent THF, flow rate 1 mL/min, T = 293 K).

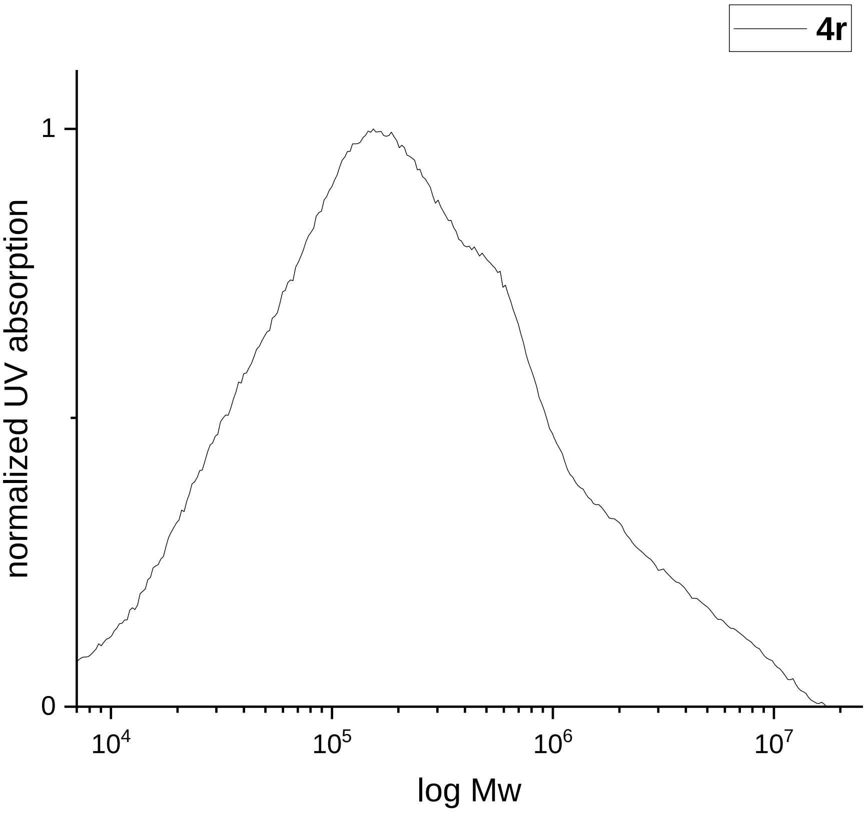


**Figure S114.** GPC trace of polymer **4r** (eluent THF, flow rate 1 mL/min, T = 293 K).

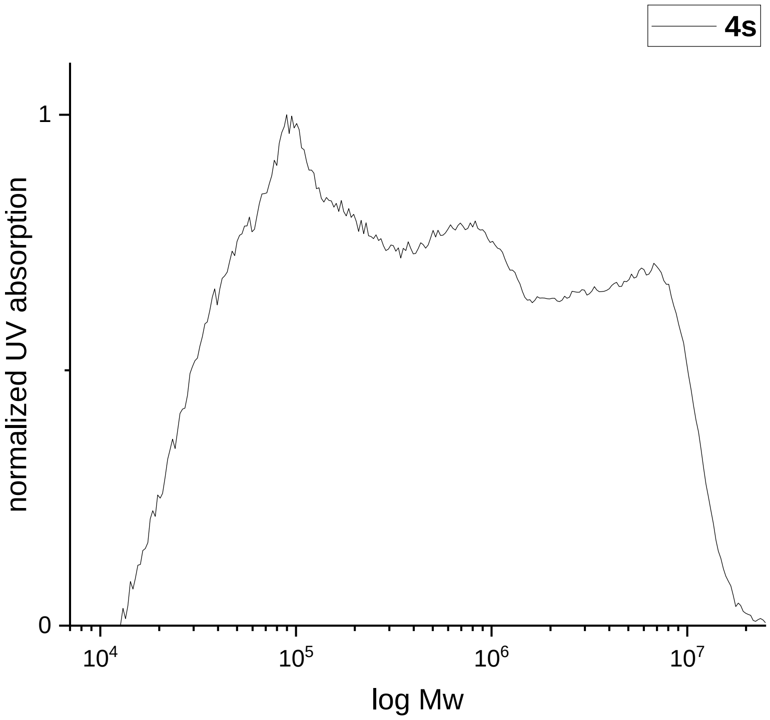


**Figure S115.** GPC trace of polymer **4s** (eluent THF, flow rate 1 mL/min, T = 293 K).

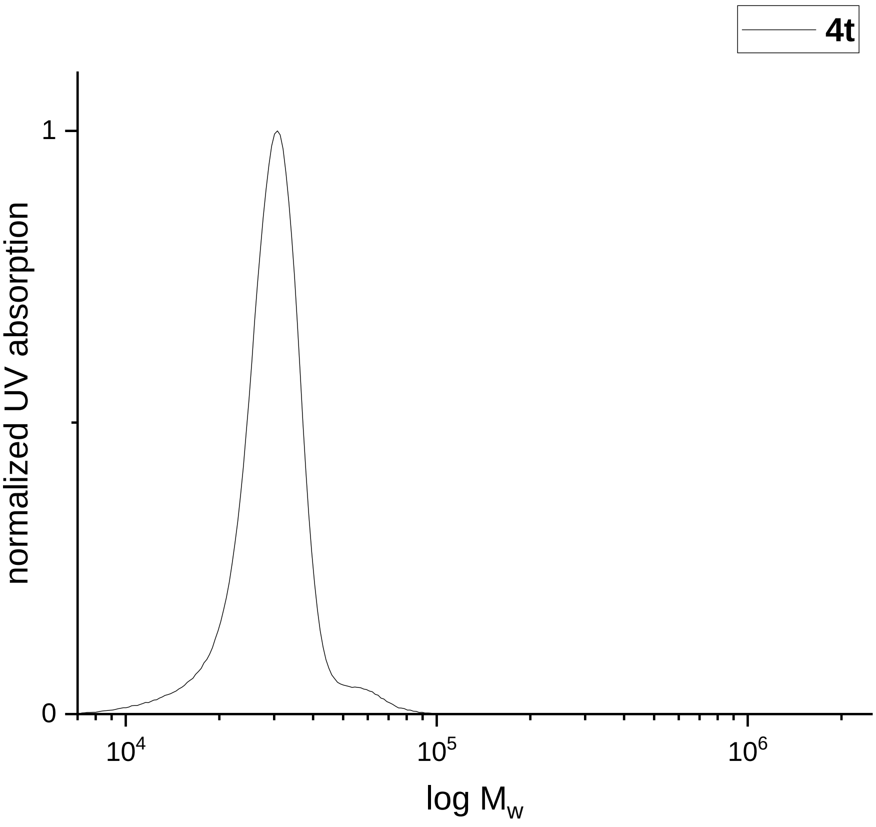


**Figure S116.** GPC trace of polymer **4t** (eluent THF, flow rate 1 mL/min, T = 293 K).

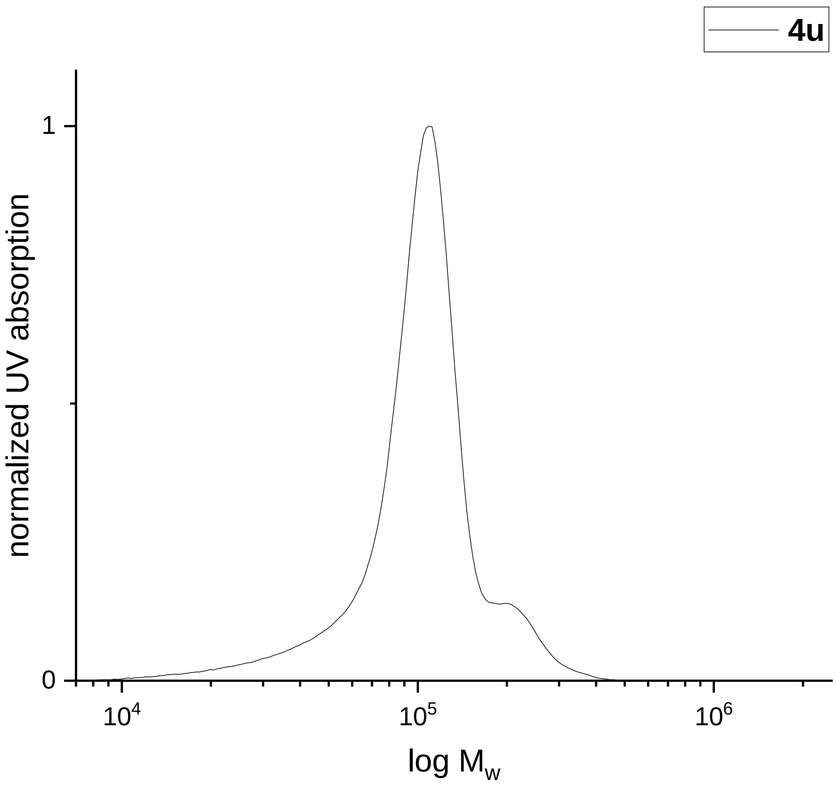


**Figure S117.** GPC trace of polymer **4u** (eluent THF, flow rate 1 mL/min, T = 293 K).

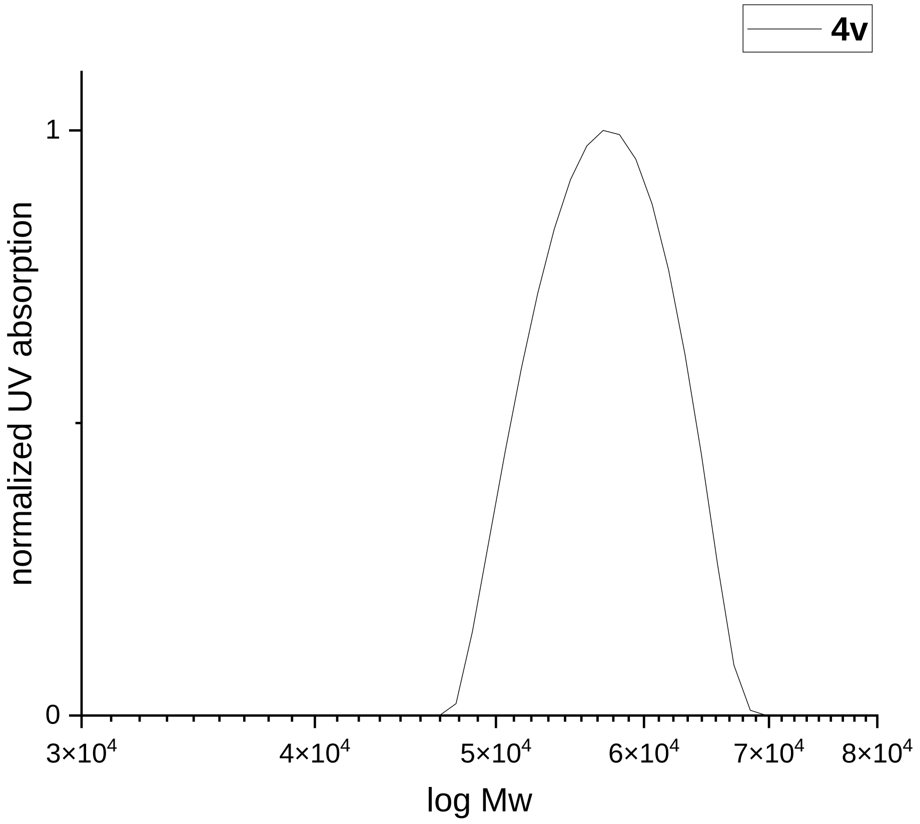


**Figure S118.** GPC trace of polymer **4v** (eluent THF, flow rate 1 mL/min, T = 293 K).

# 6.0 NMR spectra

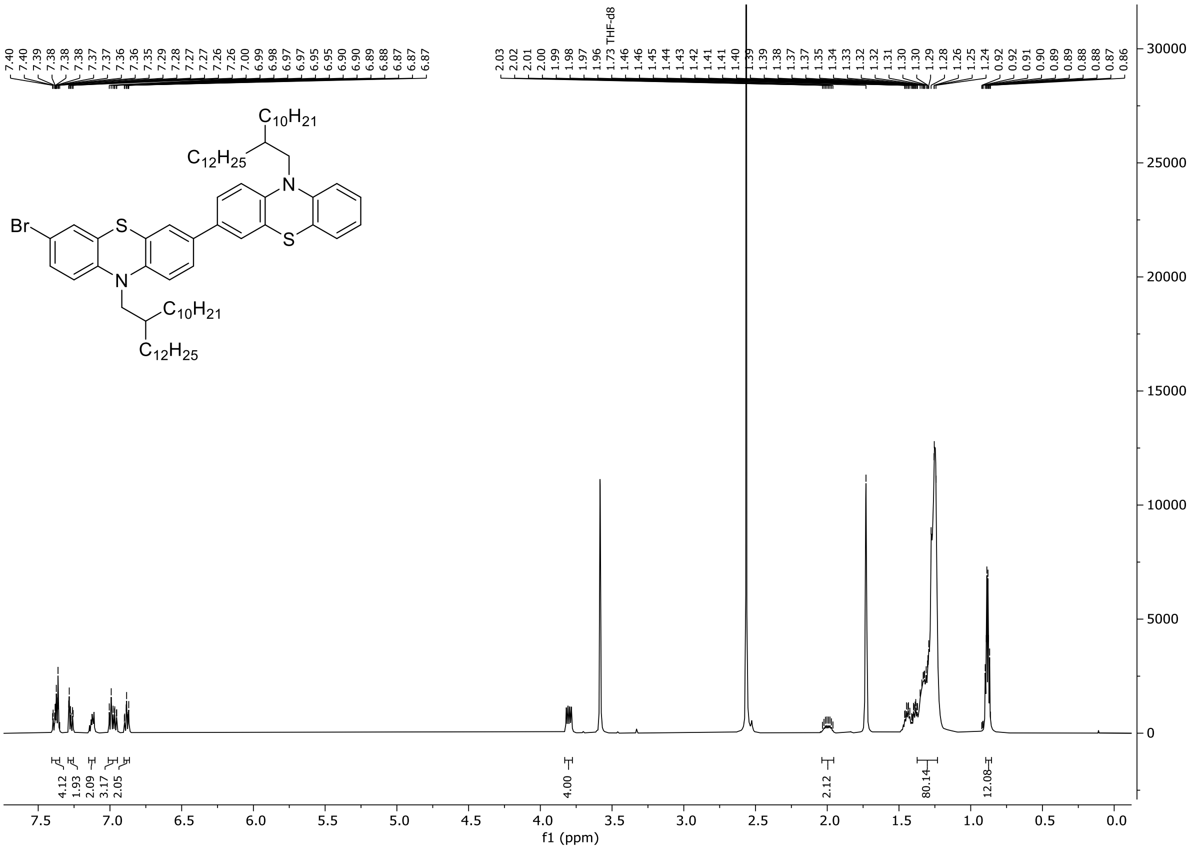

**Figure S119.** $^{1}H$ NMR spectrum of compound **1aa** (600 MHz, acetone-$d_6$, $T$ = 293 K)

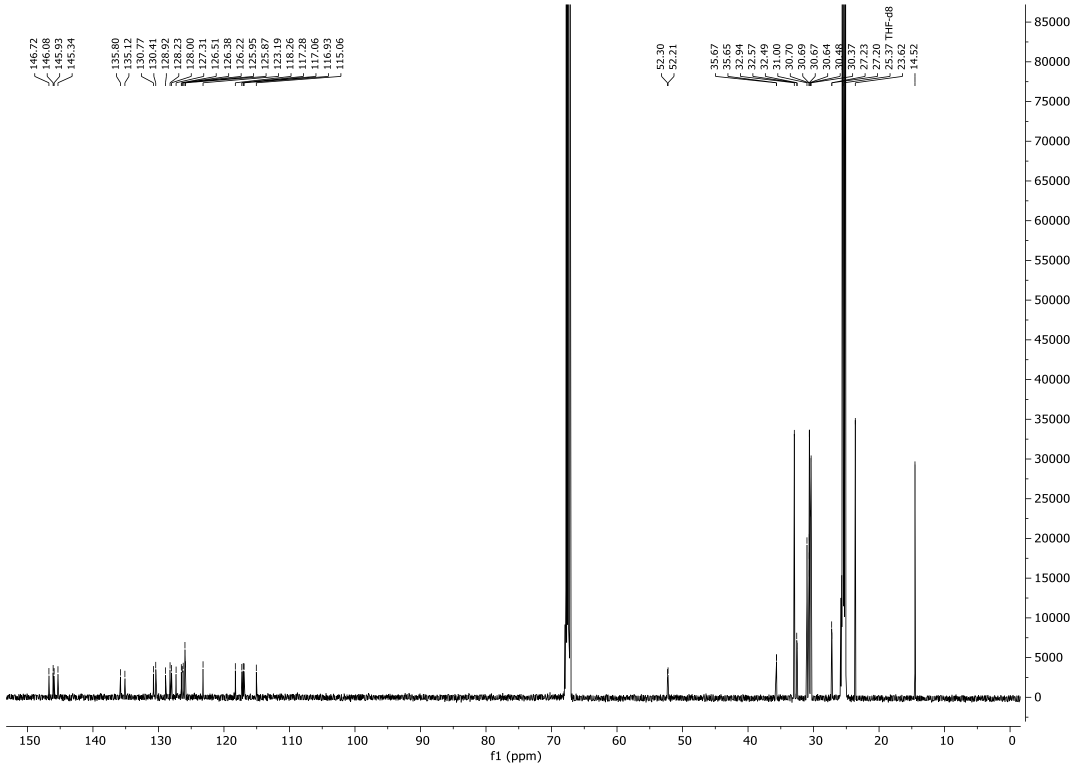

**Figure S120.** $^{13}C$ NMR spectrum of compound **1aa** (150 MHz, acetone-$d_6$, $T$ = 293 K).

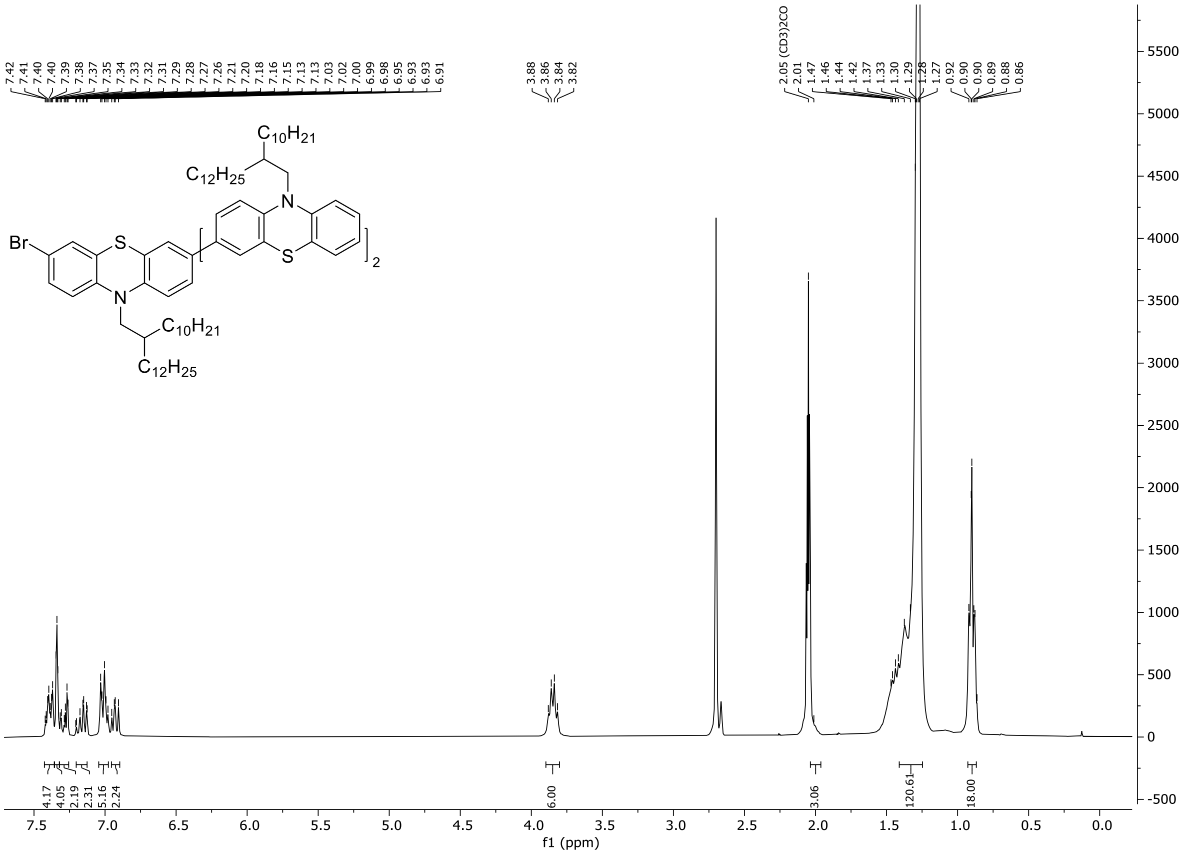


**Figure S121.** $^{1}H$ NMR spectrum of compound **1ab** (300 MHz, acetone-$d_6$, *T* = 293 K).

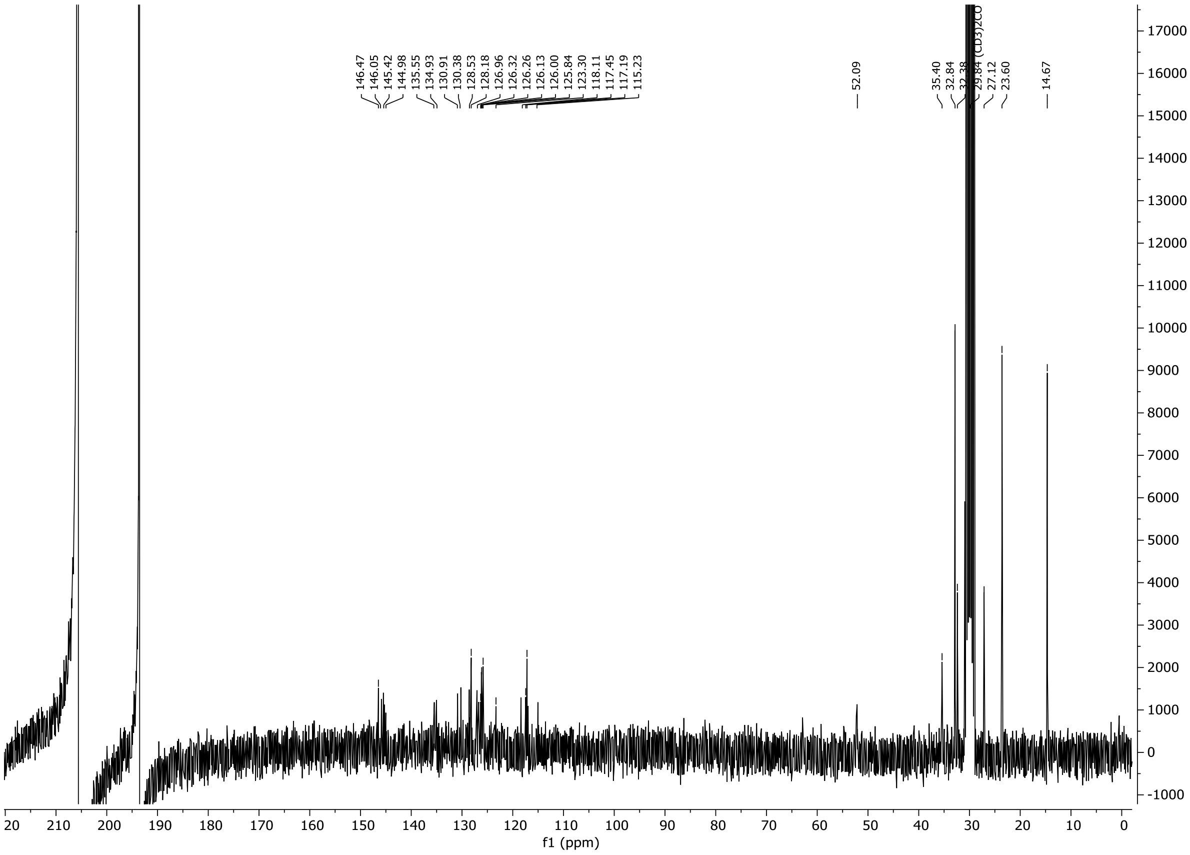


**Figure S122.** $^{13}C$ NMR spectrum of compound **1ab** (75 MHz, acetone-$d_6$, *T* = 293 K).

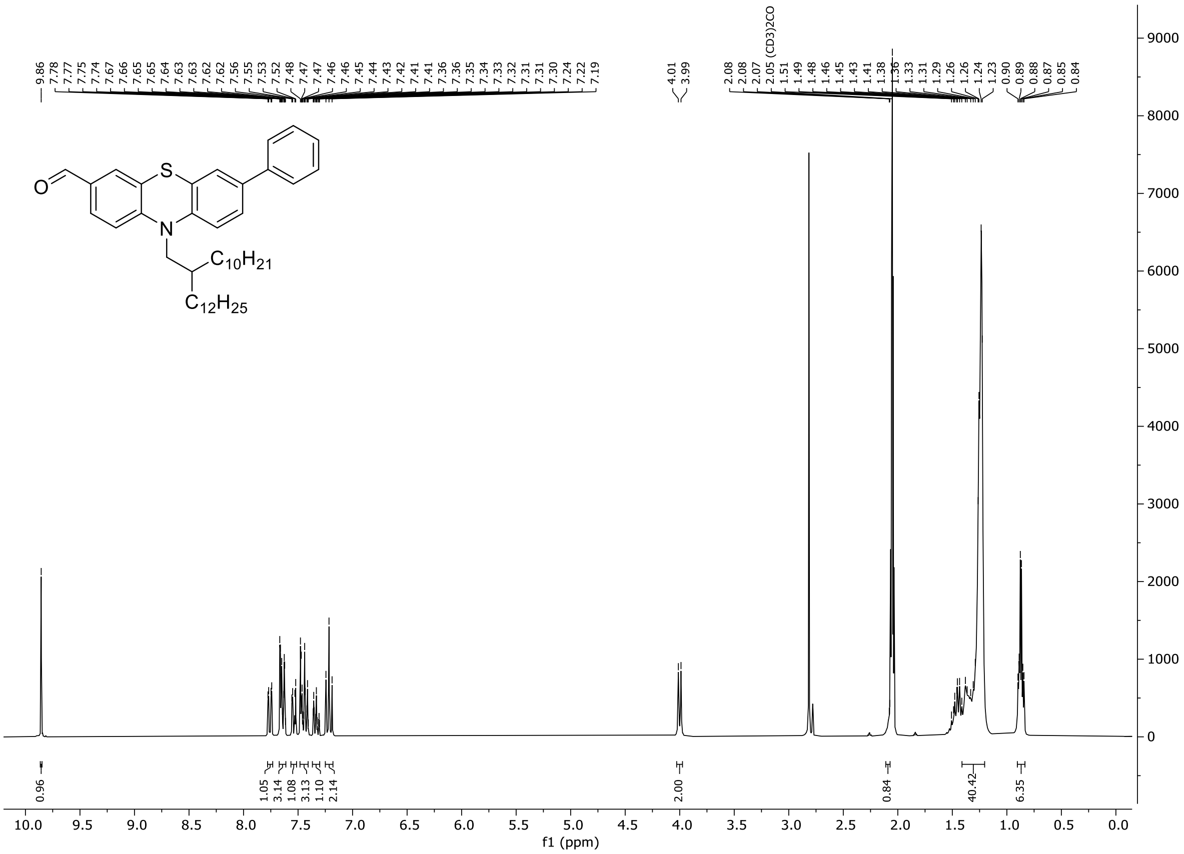

**Figure S123.** $^1$H NMR spectrum of compound **2a** (300 MHz, acetone-$d_6$, *T* = 293 K).

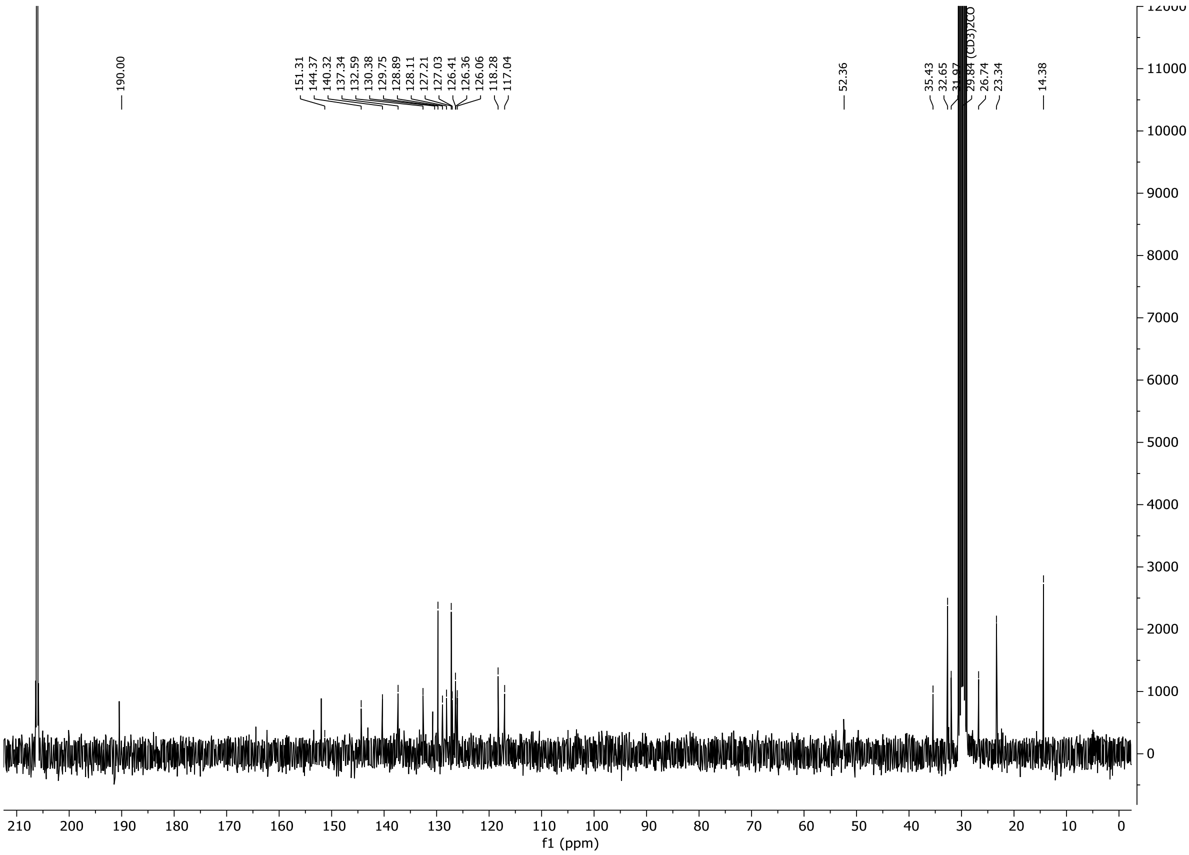

**Figure S124.** $^{13}$C NMR spectrum of compound **2a** (75 MHz, acetone-$d_6$, *T* = 293 K).

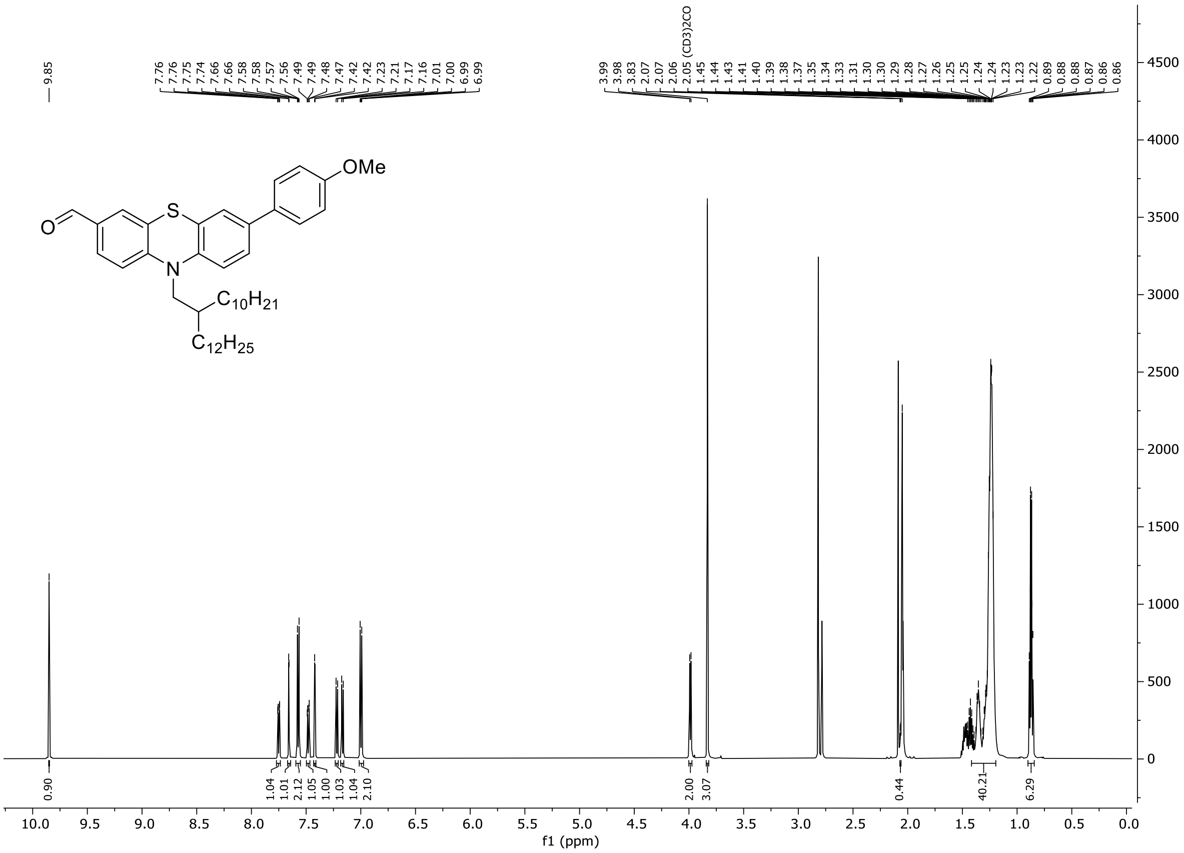

**Figure S125.** $^{1}$H NMR spectrum of compound **2b** (600 MHz, acetone-$d_6$, $T$ = 293 K).

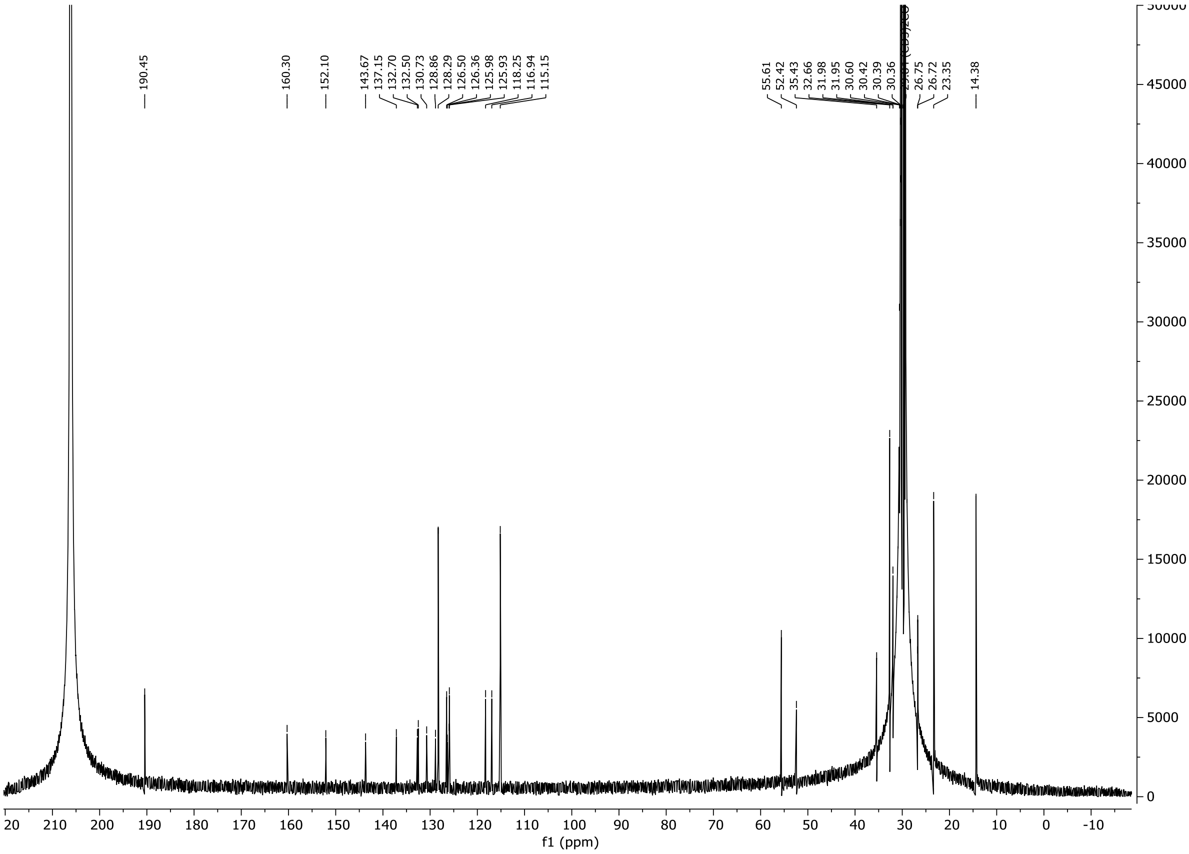

**Figure S126.** $^{13}$C NMR spectrum of compound **2b** (150 MHz, acetone-$d_6$, $T$ = 293 K).

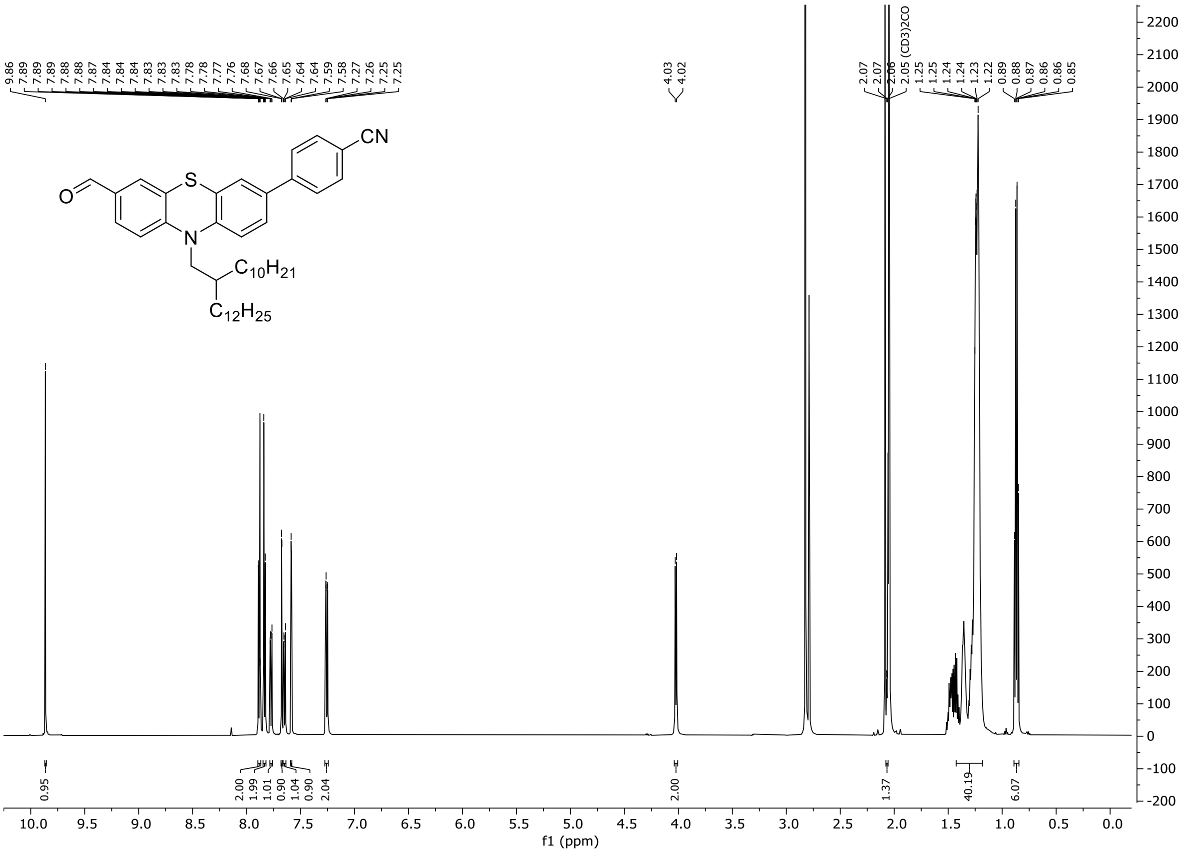

**Figure S127.** $^{1}H$ NMR spectrum of compound **2c** (600 MHz, acetone-$d_6$, *T* = 293 K).

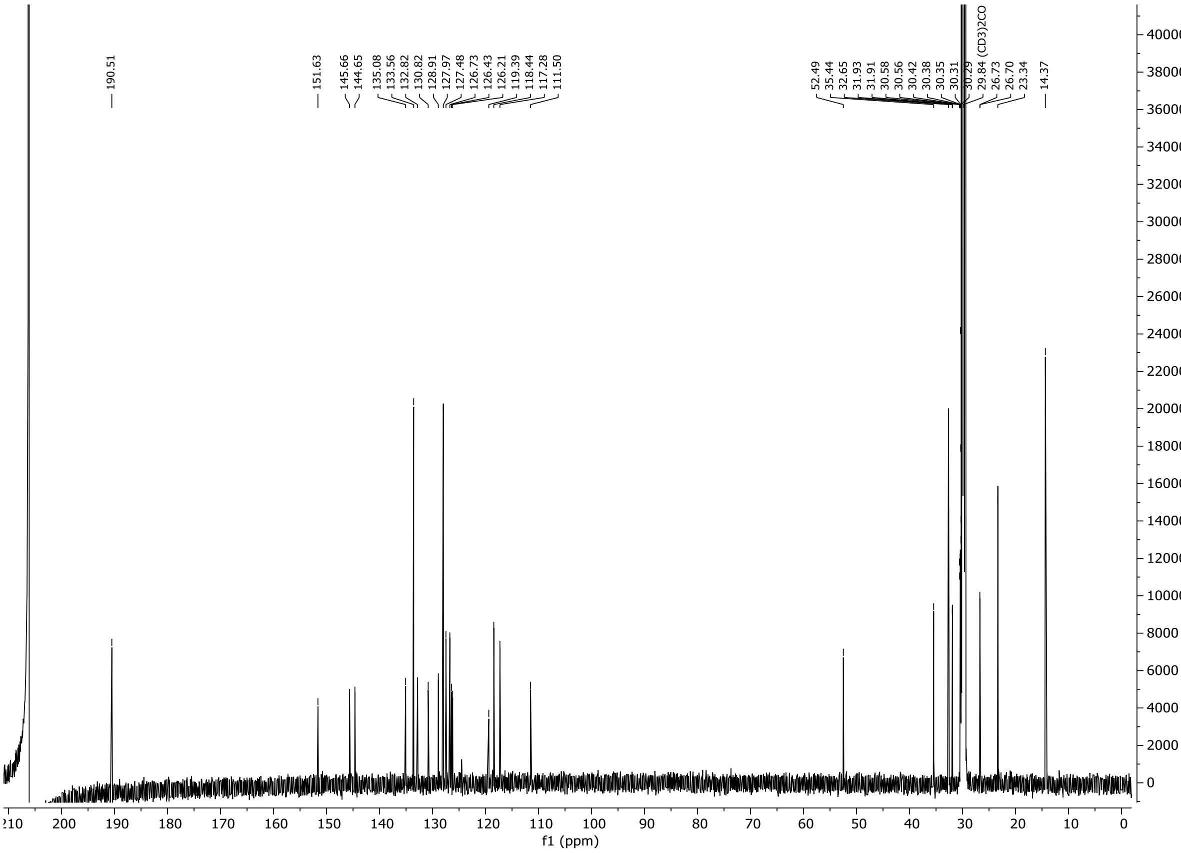

**Figure 128.** $^{13}C$ NMR spectrum of compound **2c** (150 MHz, acetone-$d_6$, *T* = 293 K).

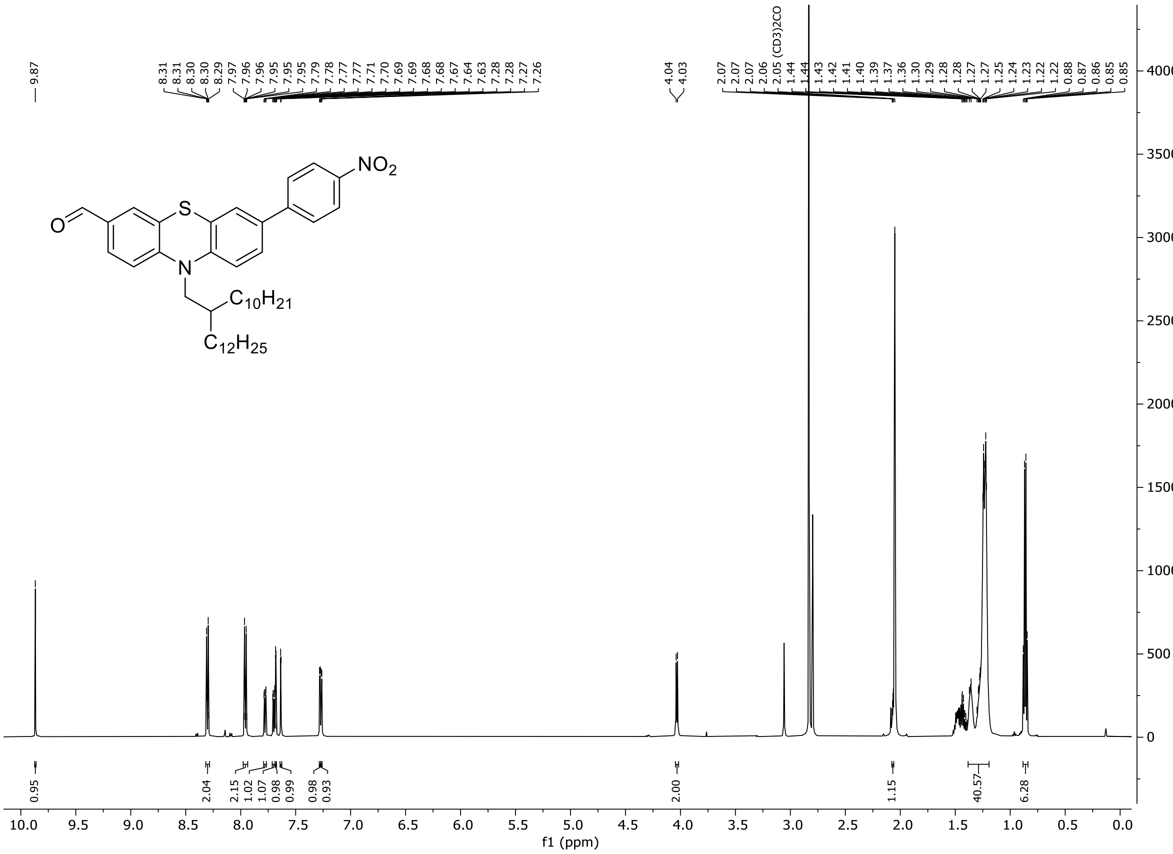

**Figure S129.** $^{1}$H NMR spectrum of compound **2d** (600 MHz, acetone-d$_6$, $T$ = 293 K).

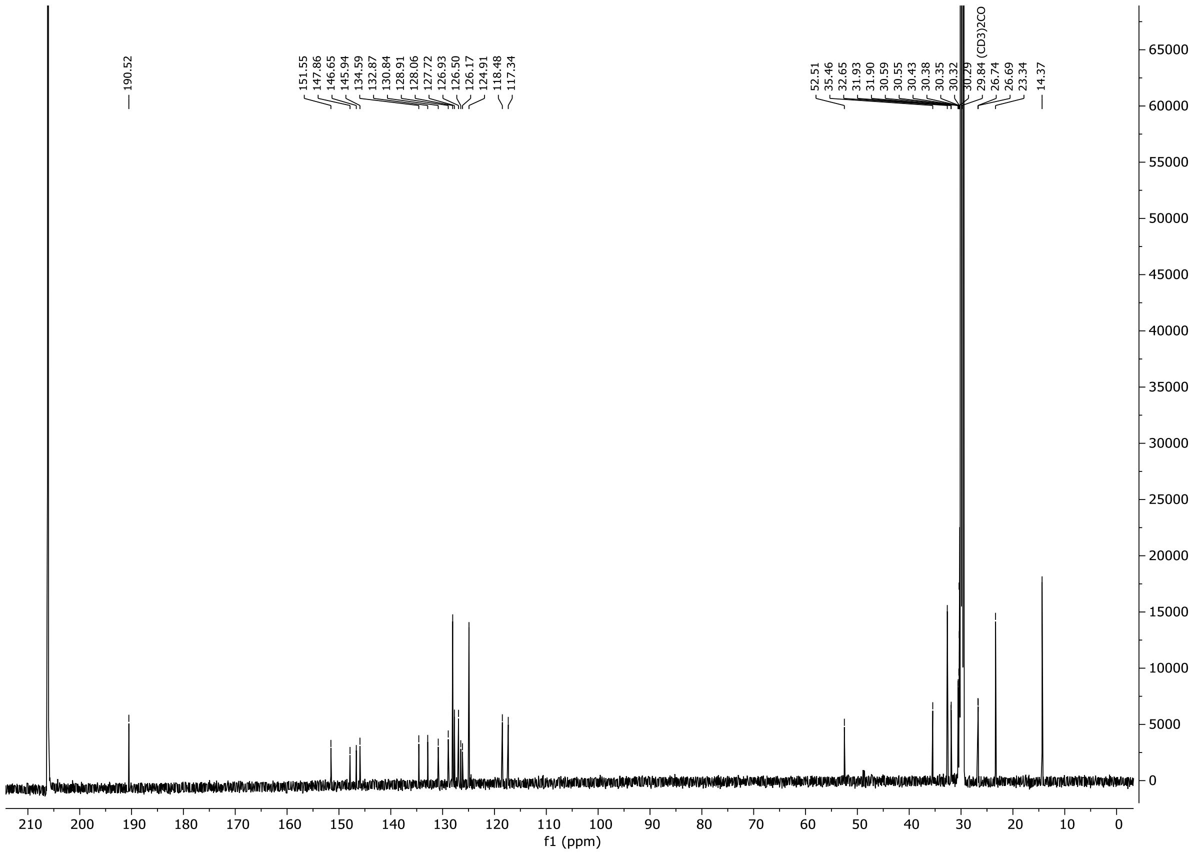


**Figure S130.** $^{13}C$ NMR spectrum of compound **2d** (150 MHz, acetone-$d_6$, $T$ = 293 K).

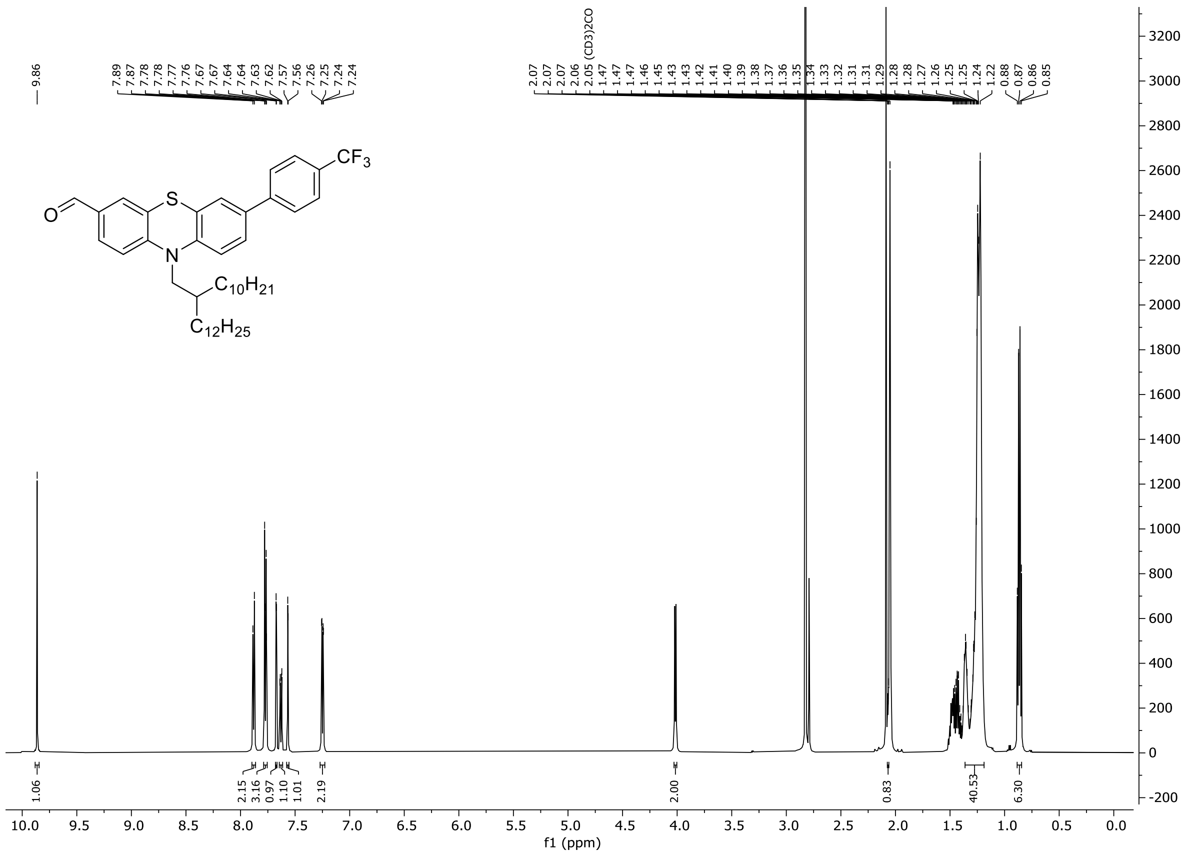

**Figure S131.** $^{1}$H NMR spectrum of compound **2e** (300 MHz, acetone-d$_6$, *T* = 293 K).

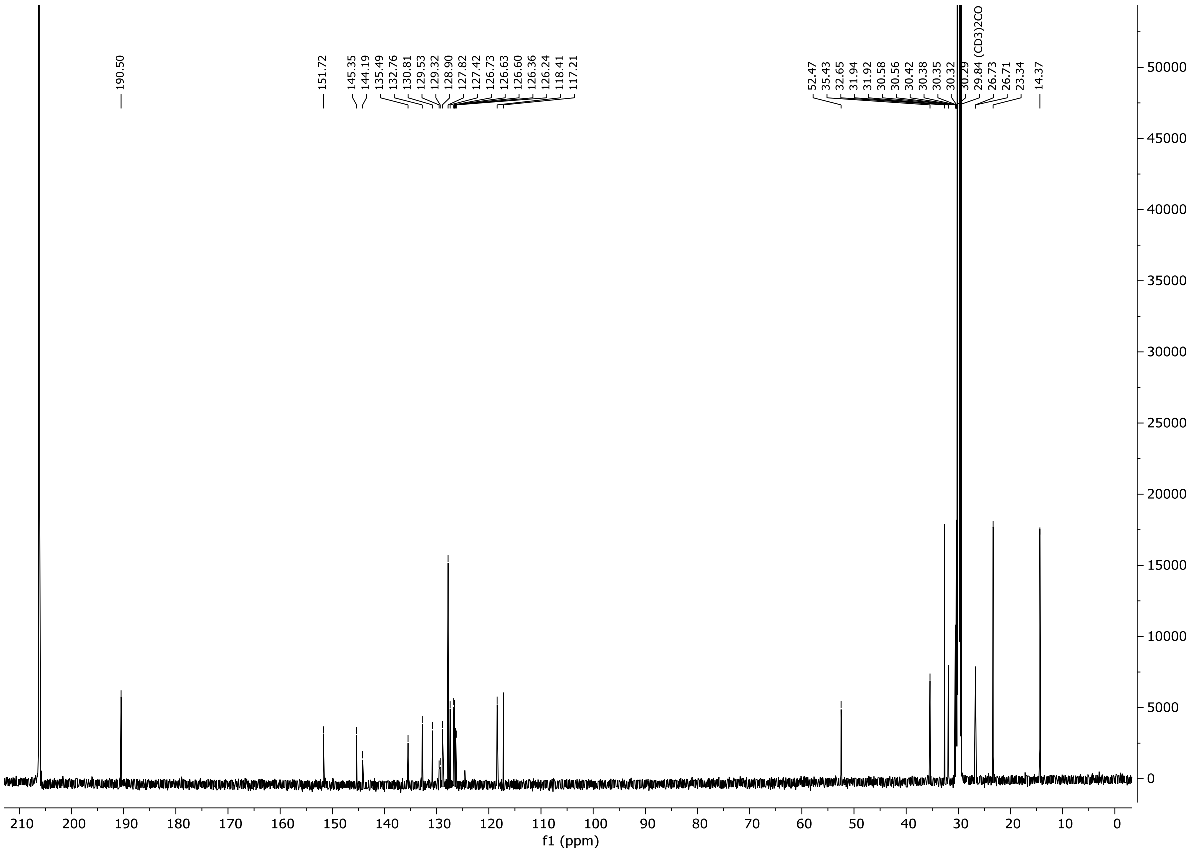


**Figure S132.** $^{13}$C NMR spectrum of compound **2e** (75 MHz, acetone-d$_6$, *T* = 293 K).

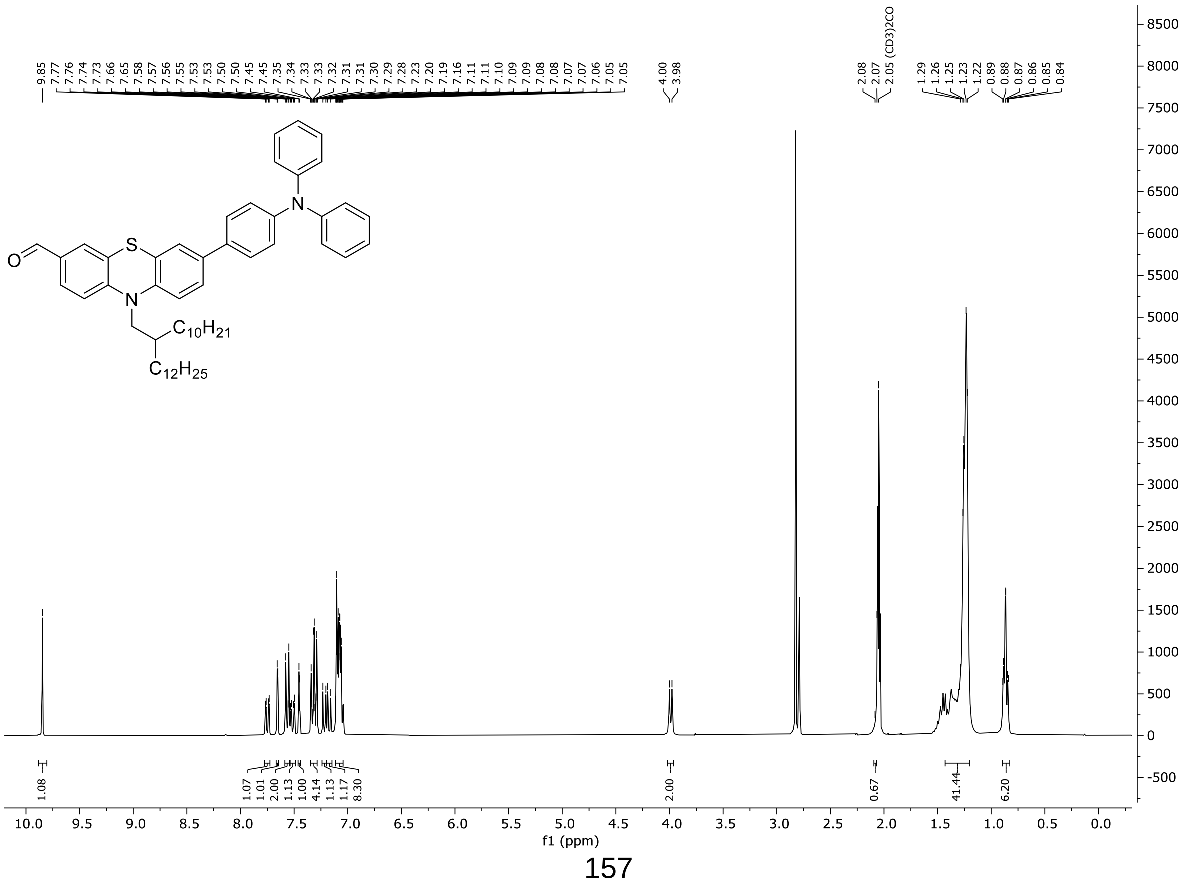

**Figure S133.** $^{1}$H NMR spectrum of compound **2f** (300 MHz, acetone-d$_6$, $T$ = 293 K).

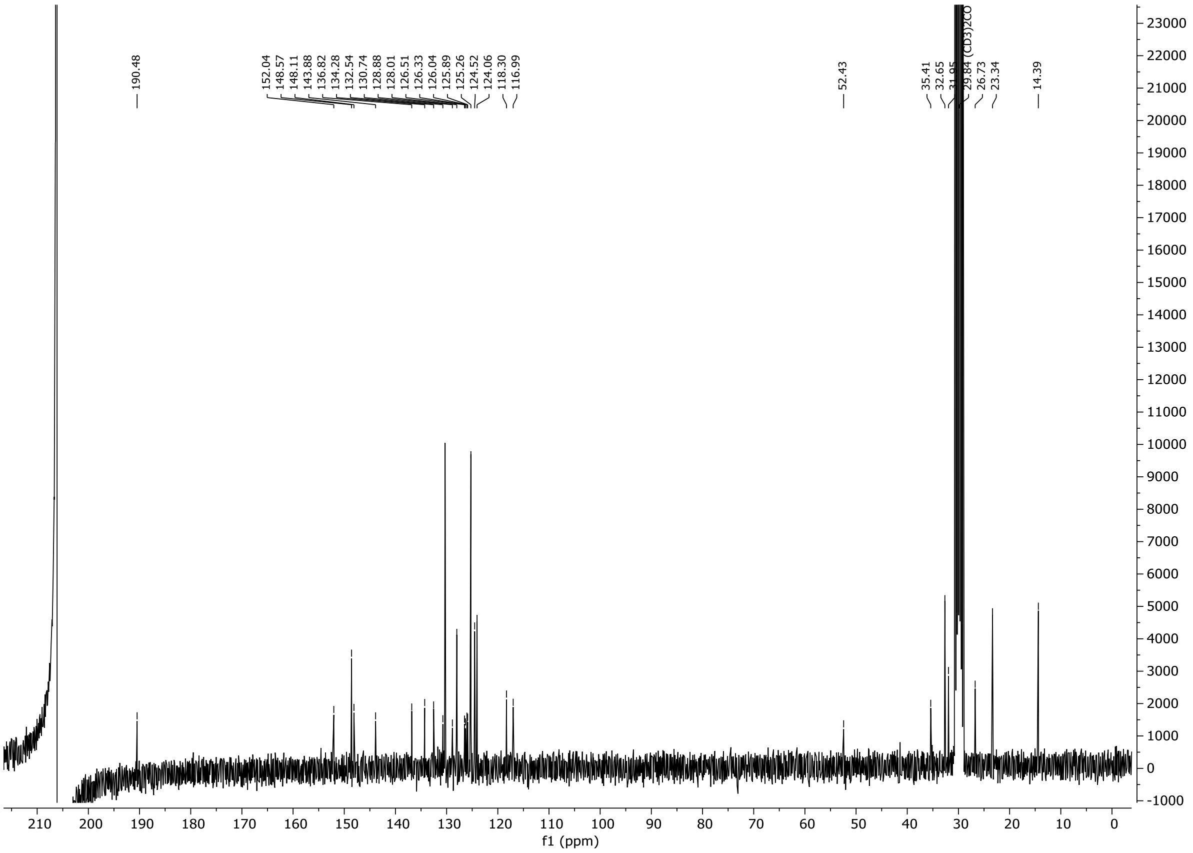


**Figure S134.** $^{13}$C NMR spectrum of compound **2f** (75 MHz, acetone-d$_6$, $T$ = 293 K).

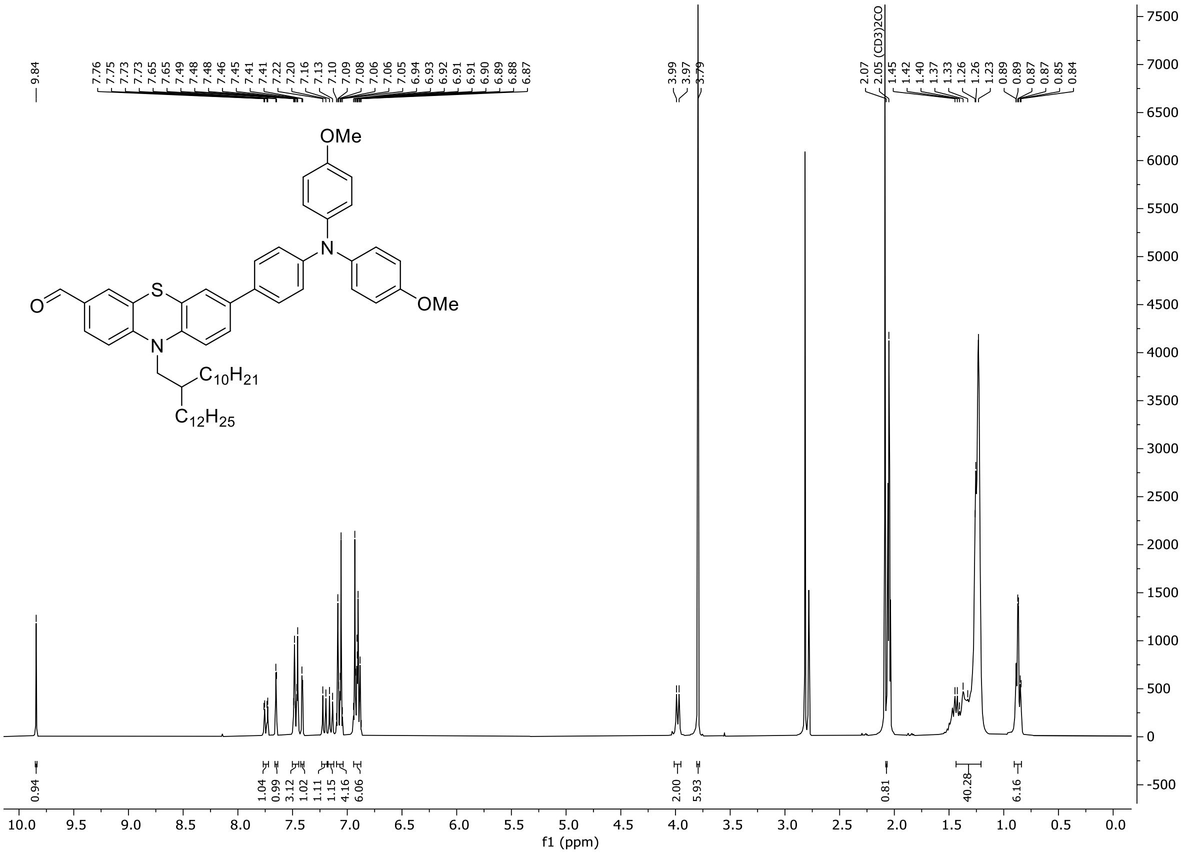

**Figure S135.** $^{1}H$ NMR spectrum of compound **2g** (300 MHz, acetone-$d_6$, $T$ = 293 K).

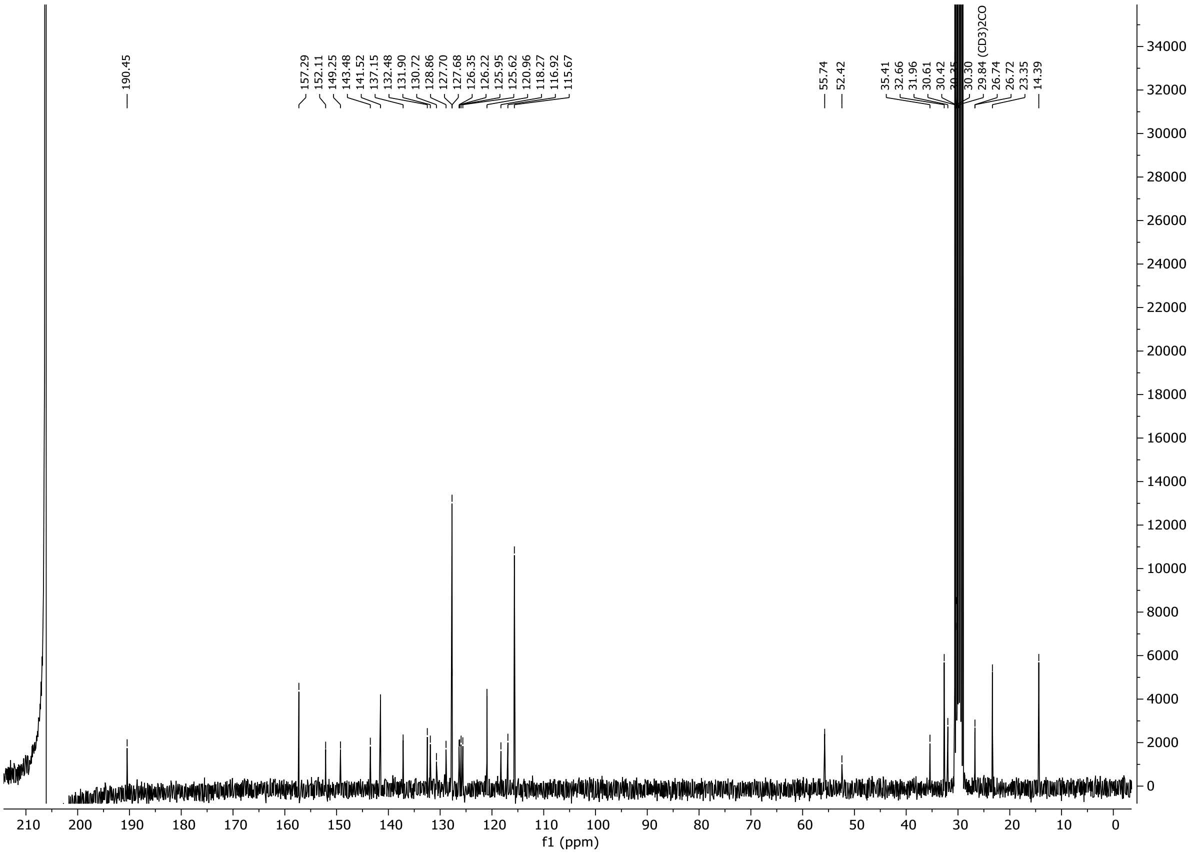

**Figure S136.** $^{13}C$ NMR spectrum of compound **2g** (75 MHz, acetone-$d_6$, $T$ = 293 K).

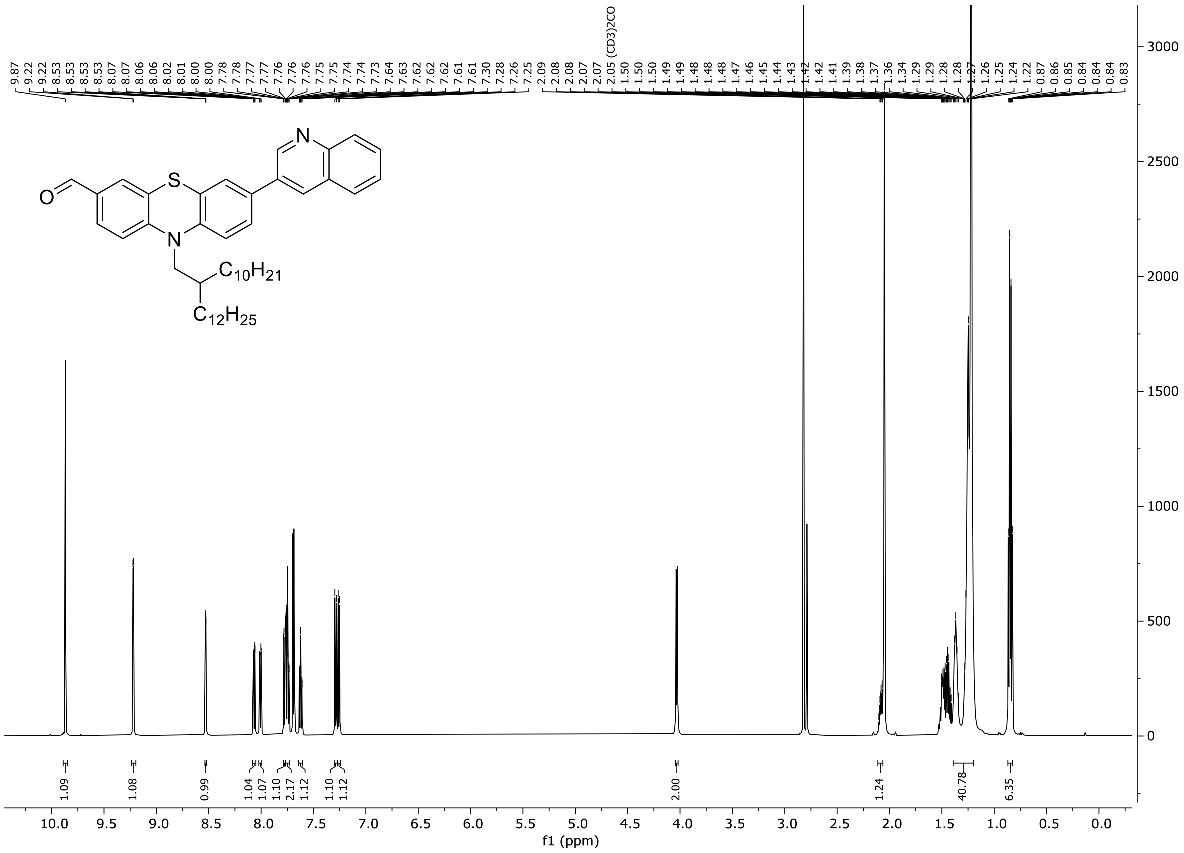

**Figure S137.** $^{1}$H NMR spectrum of compound **2h** (300 MHz, acetone-d$_6$, $T$ = 293 K).

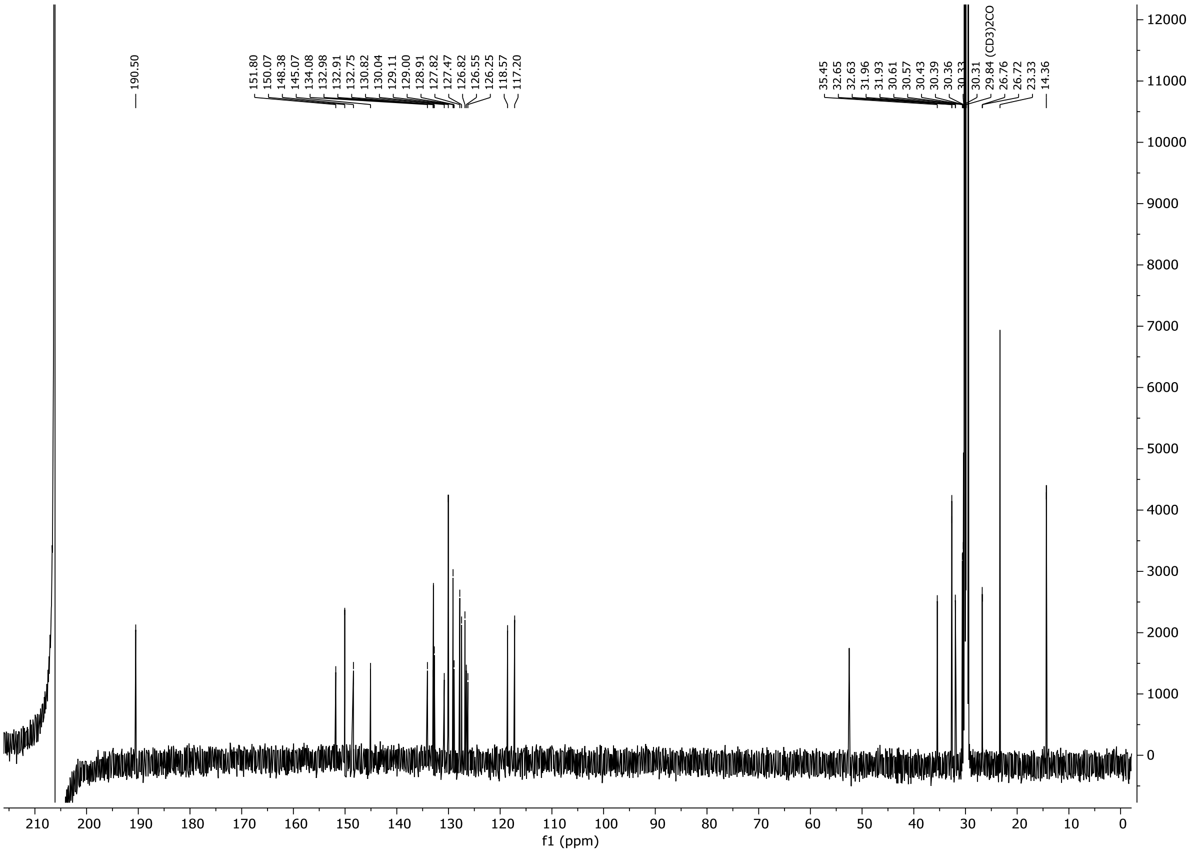

**Figure S138.** $^{13}$C NMR spectrum of compound **2h** (75 MHz, acetone-d$_6$, $T$ = 293 K).

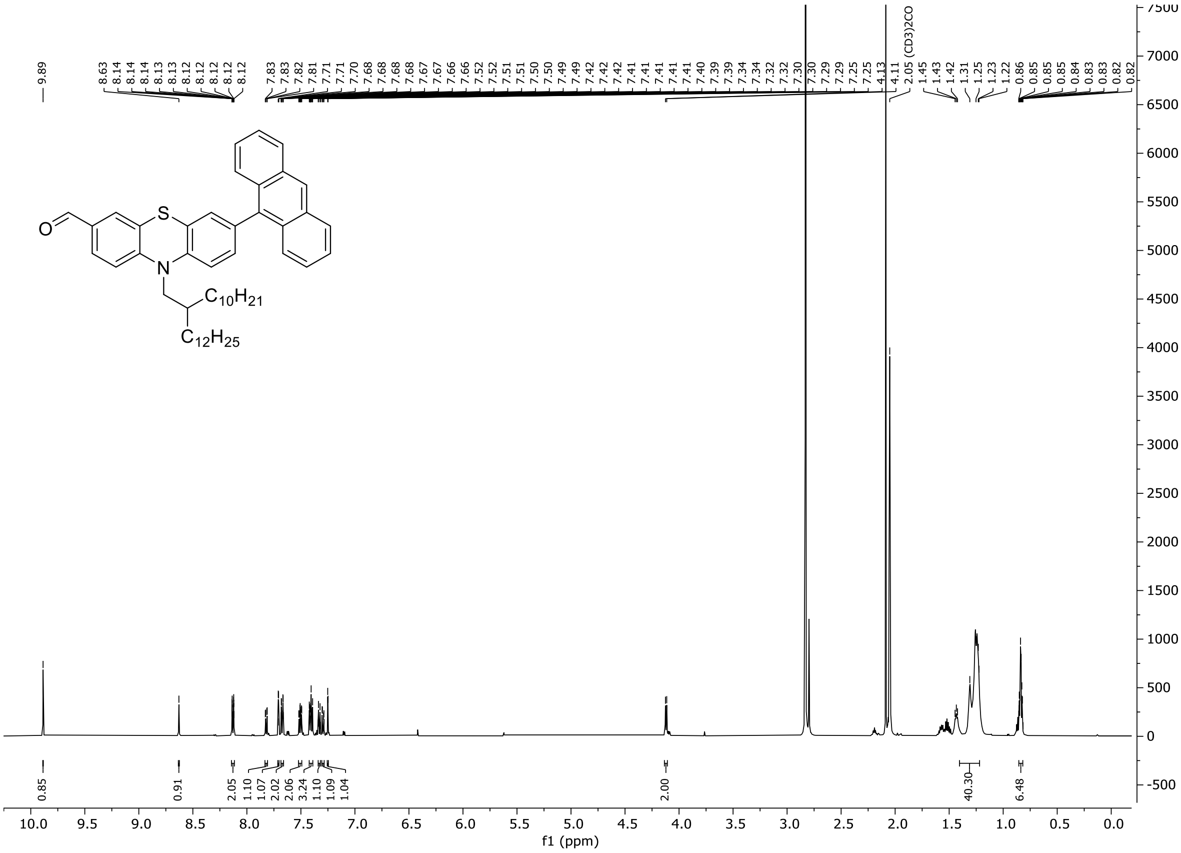

**Figure S139.** $^{1}H$ NMR spectrum of compound **2i** (300 MHz, acetone-$d_6$, $T$ = 293 K).

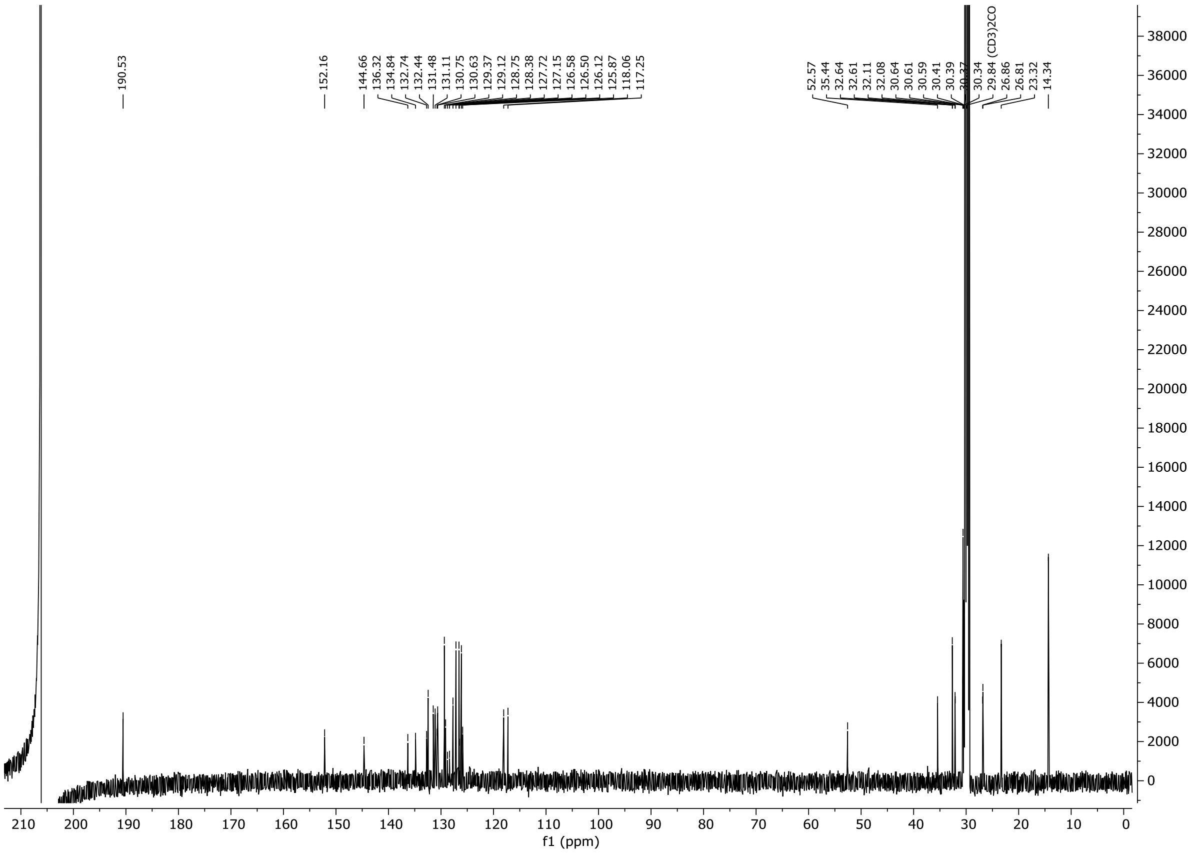

**Figure S140.** $^{13}C$ NMR spectrum of compound **2i** (75 MHz, acetone-$d_6$, $T$ = 293 K).

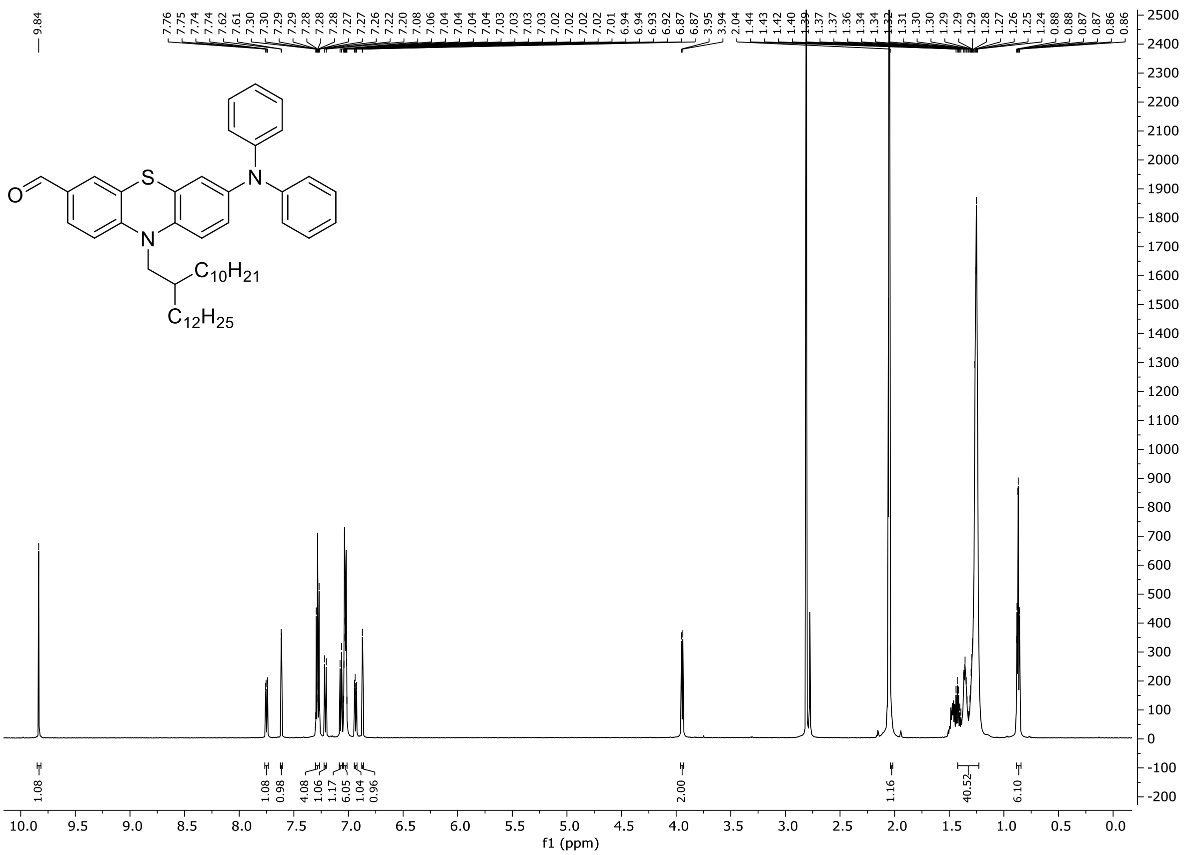

**Figure S141.** $^{1}$H NMR spectrum of compound **2k** (300 MHz, acetone-$d_6$, $T$ = 293 K).

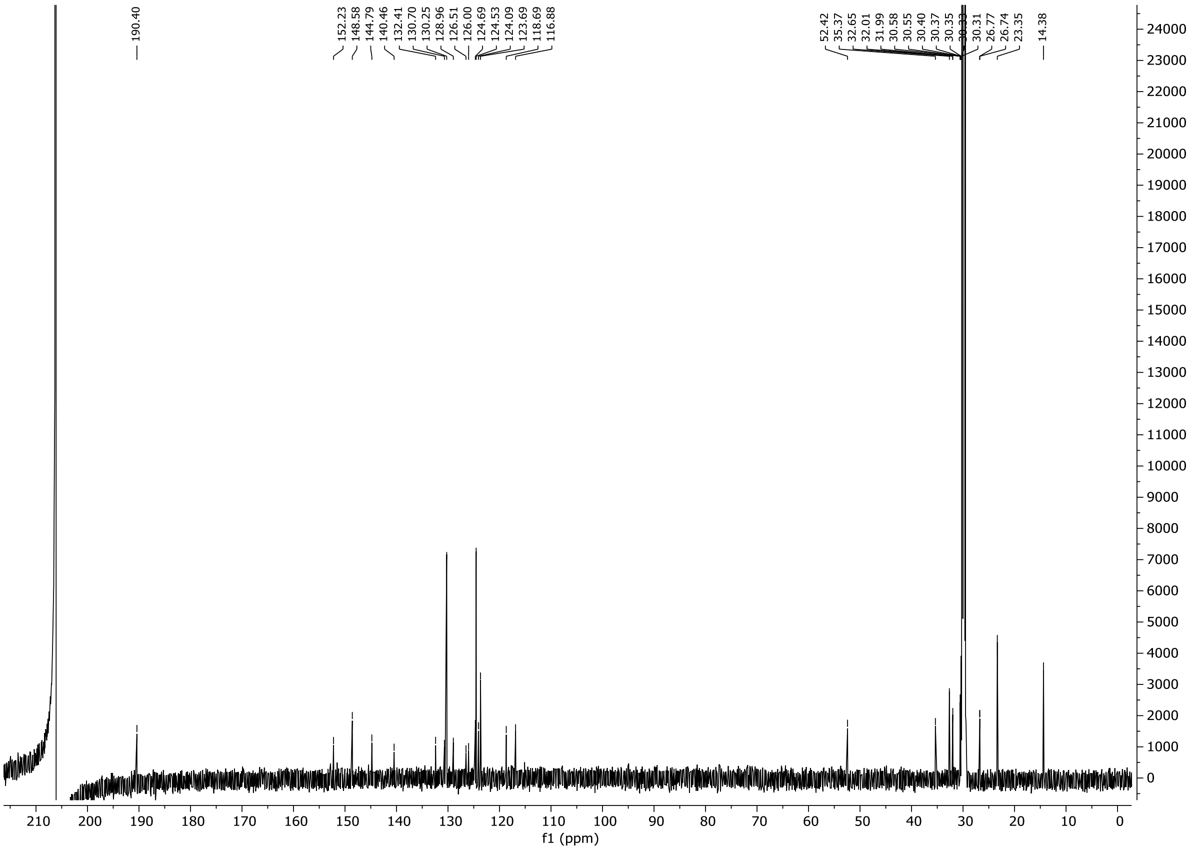

**Figure S142.** $^{13}$C NMR spectrum of compound **2k** (75 MHz, acetone-$d_6$, $T$ = 293 K).

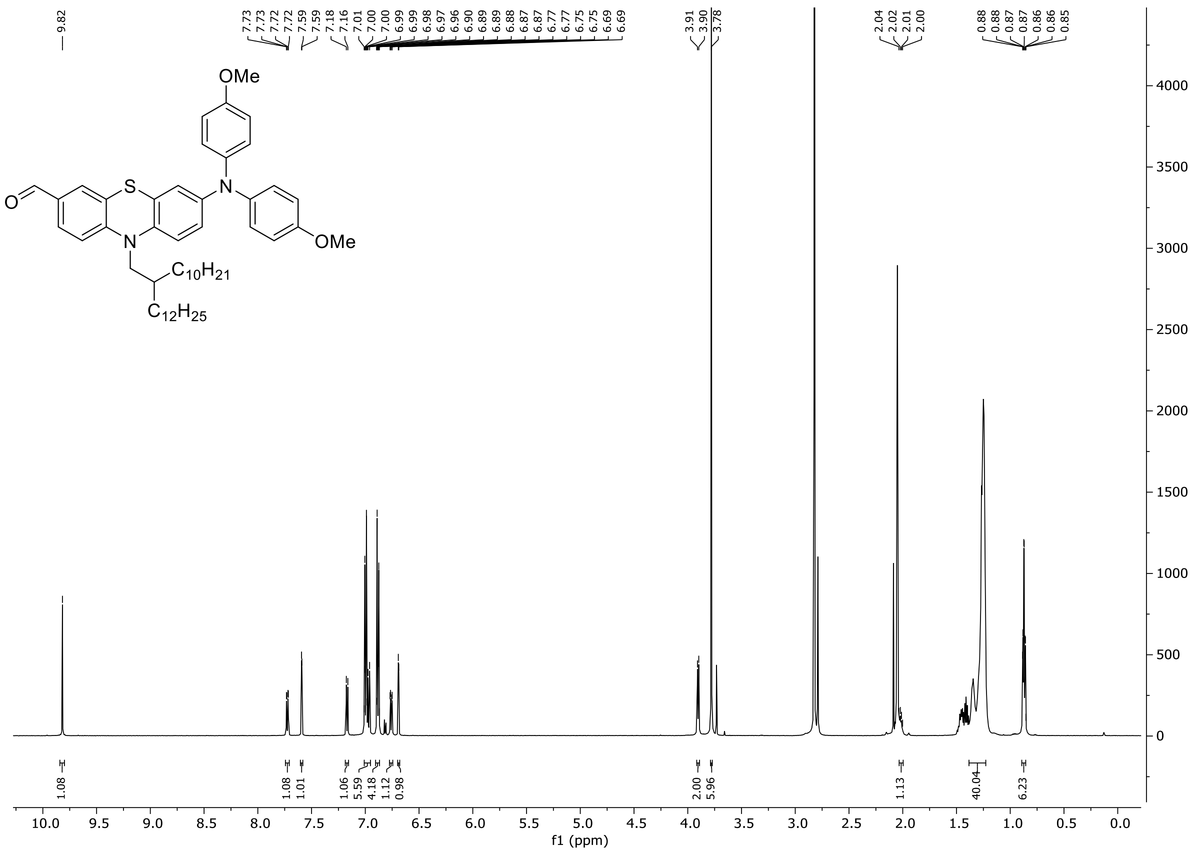

**Figure S143.** $^{1}H$ NMR spectrum of compound **2l** (300 MHz, acetone-d$_6$, *T* = 293 K).

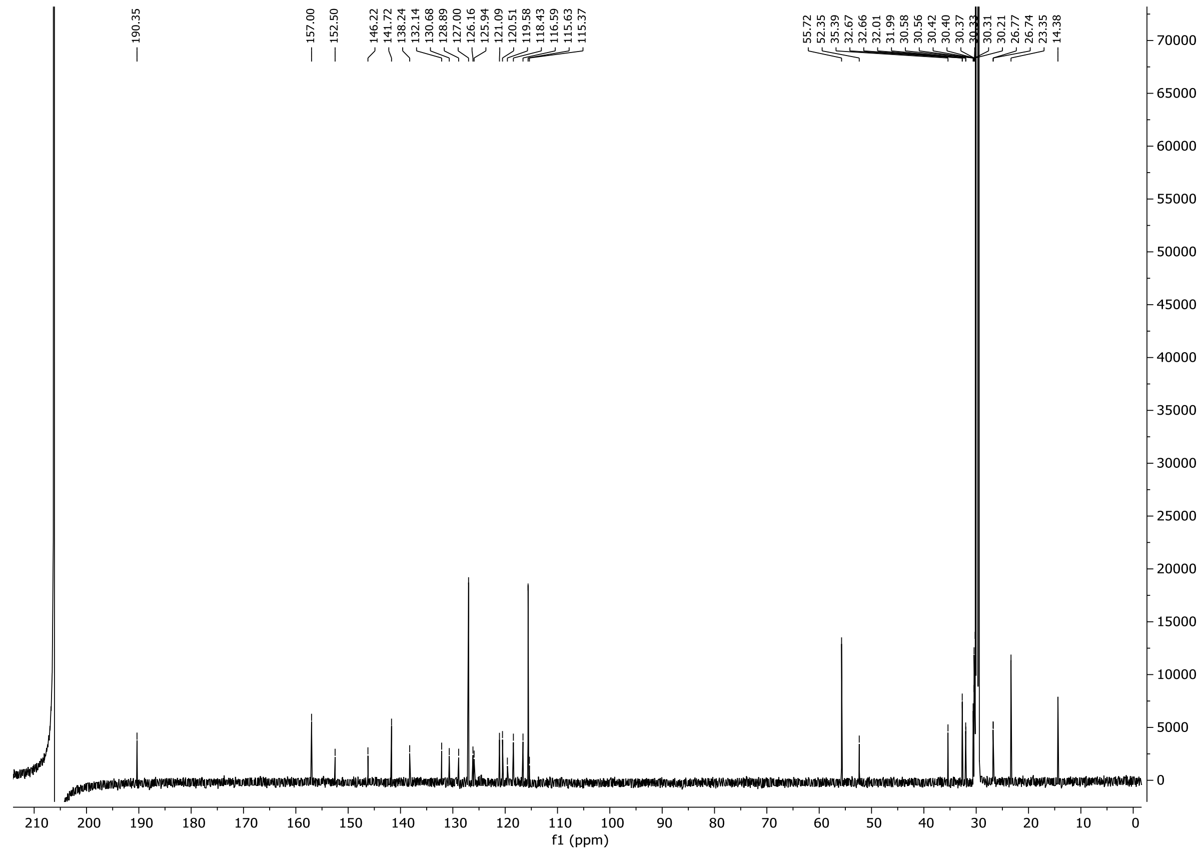

**Figure S144.** $^{13}C$ NMR spectrum of compound **2l** (75 MHz, acetone-d$_6$, *T* = 293 K).

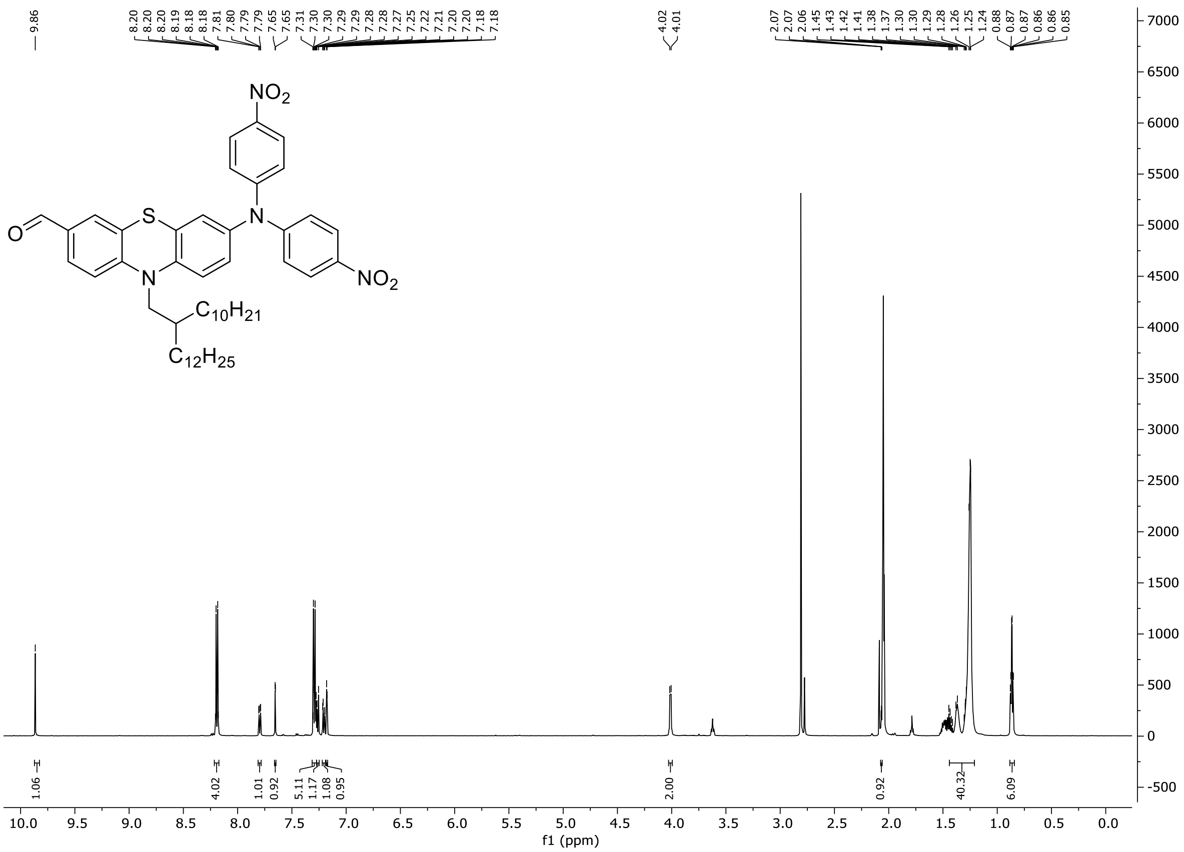


**Figure S145.** $^{1}$H NMR spectrum of compound **2n** (300 MHz, acetone-d$_6$, $T$ = 293 K).

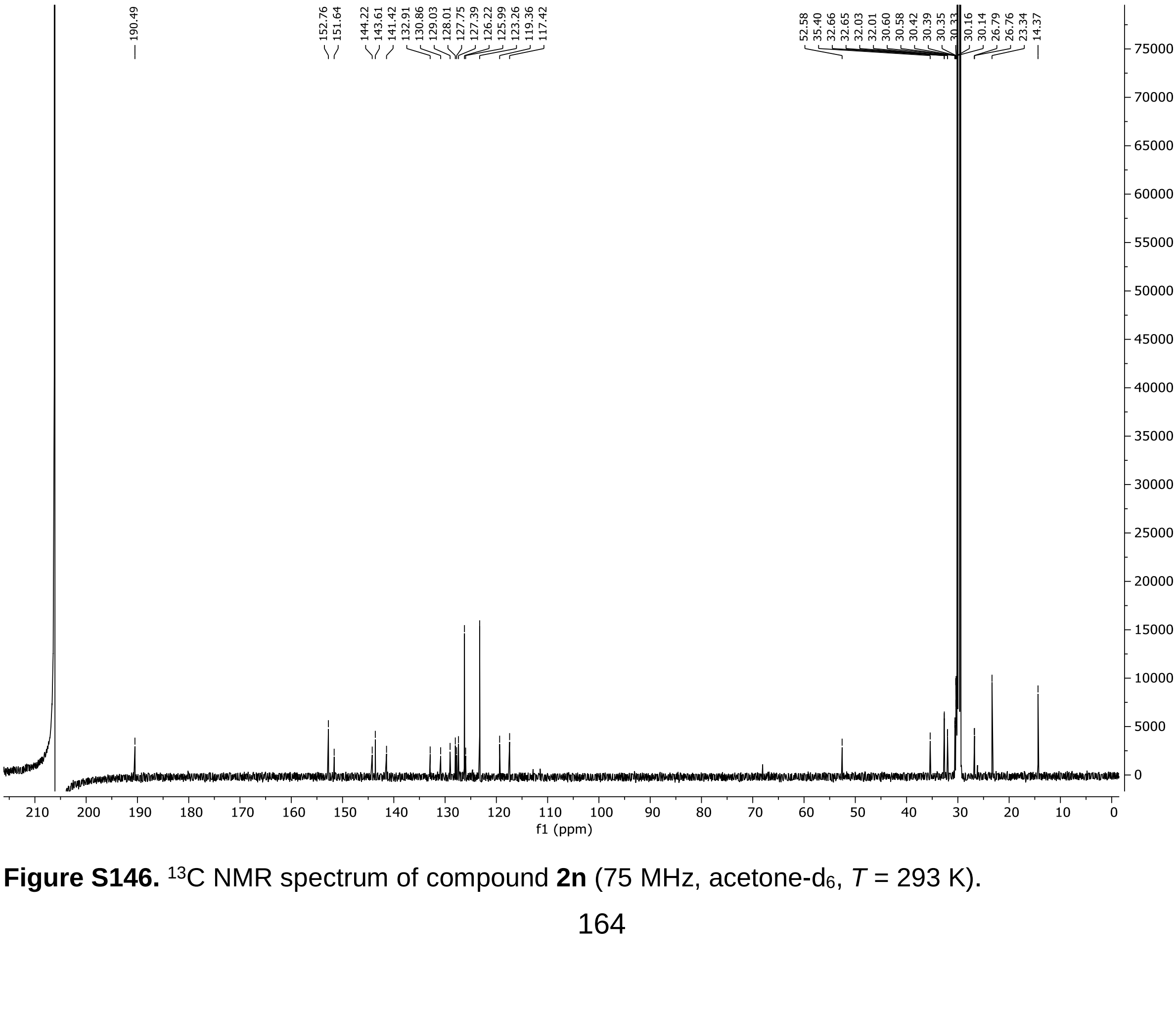


**Figure S146.** $^{13}$C NMR spectrum of compound **2n** (75 MHz, acetone-d$_6$, $T$ = 293 K).

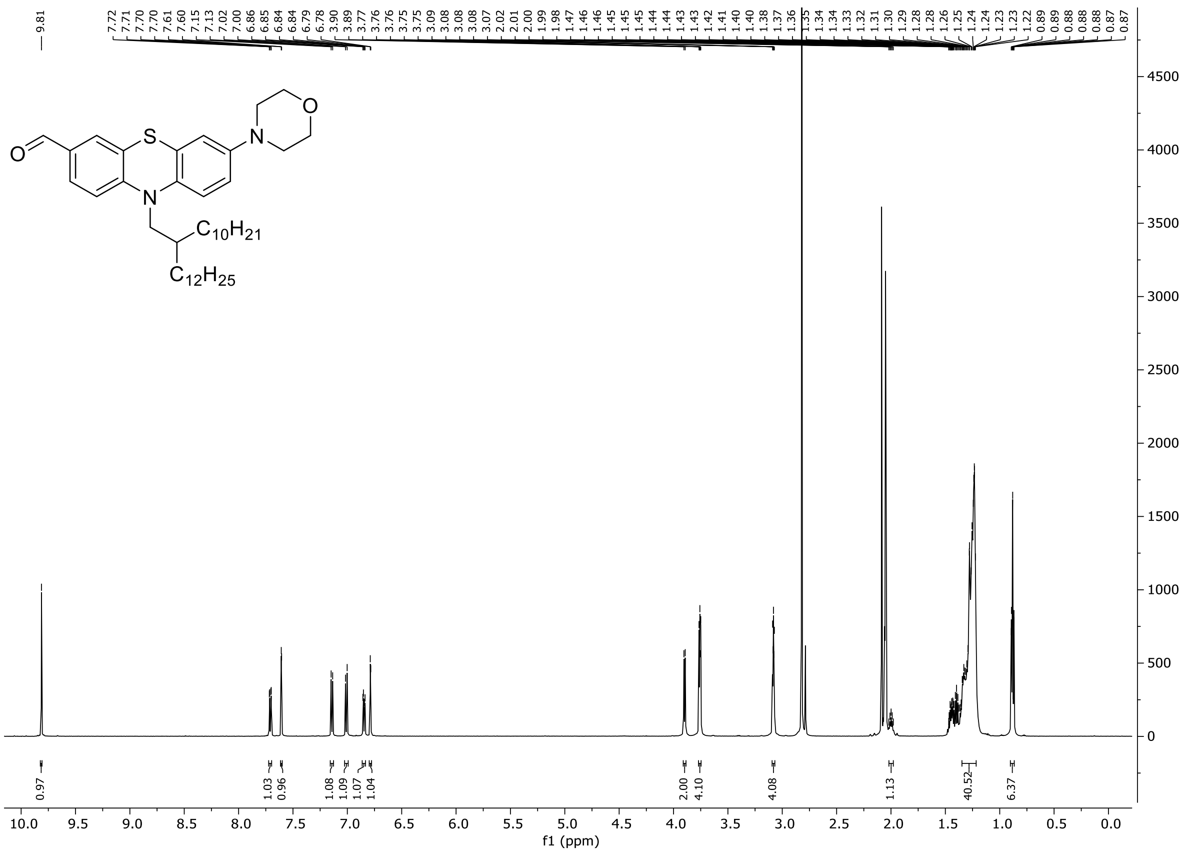


**Figure S147.** $^{1}H$ NMR spectrum of compound **2o** (300 MHz, acetone-d$_6$, $T$ = 293 K).

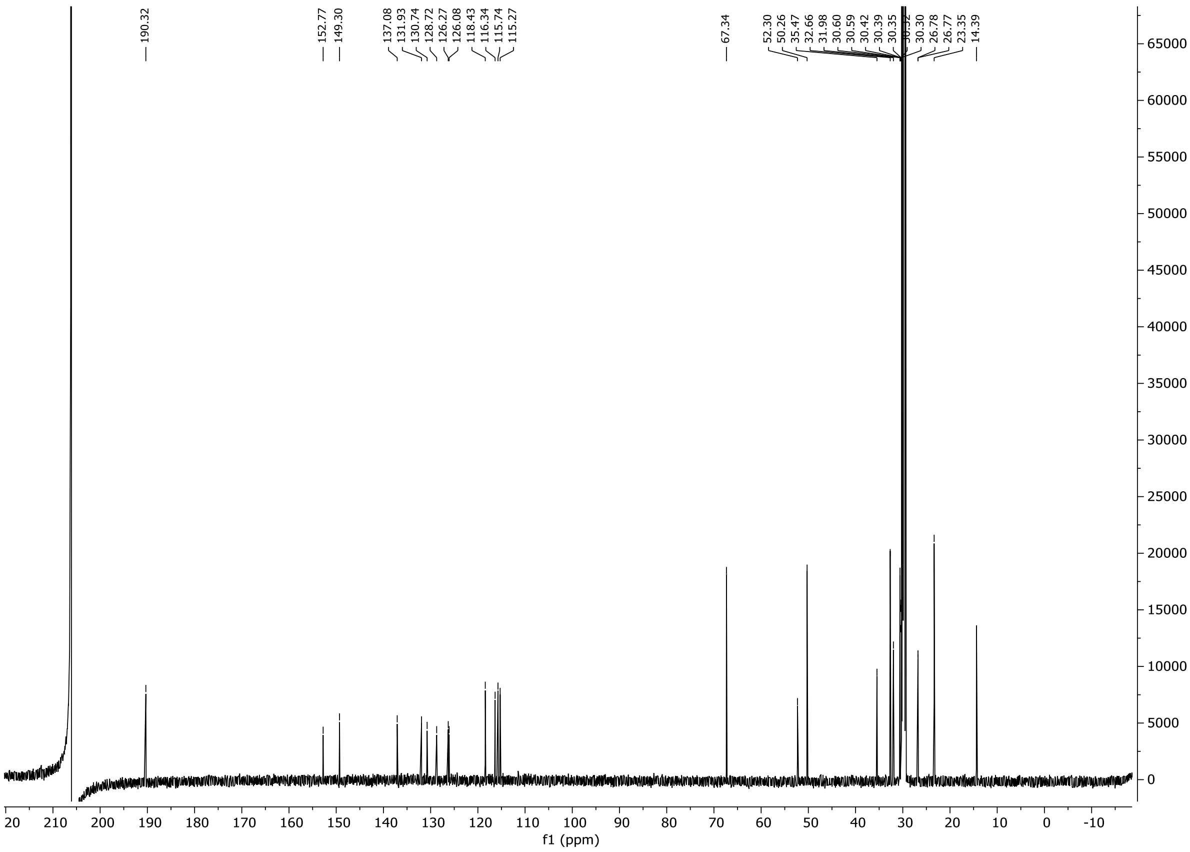


**Figure S148.** $^{13}C$ NMR spectrum of compound **2o** (75 MHz, acetone-d$_6$, $T$ = 293 K).

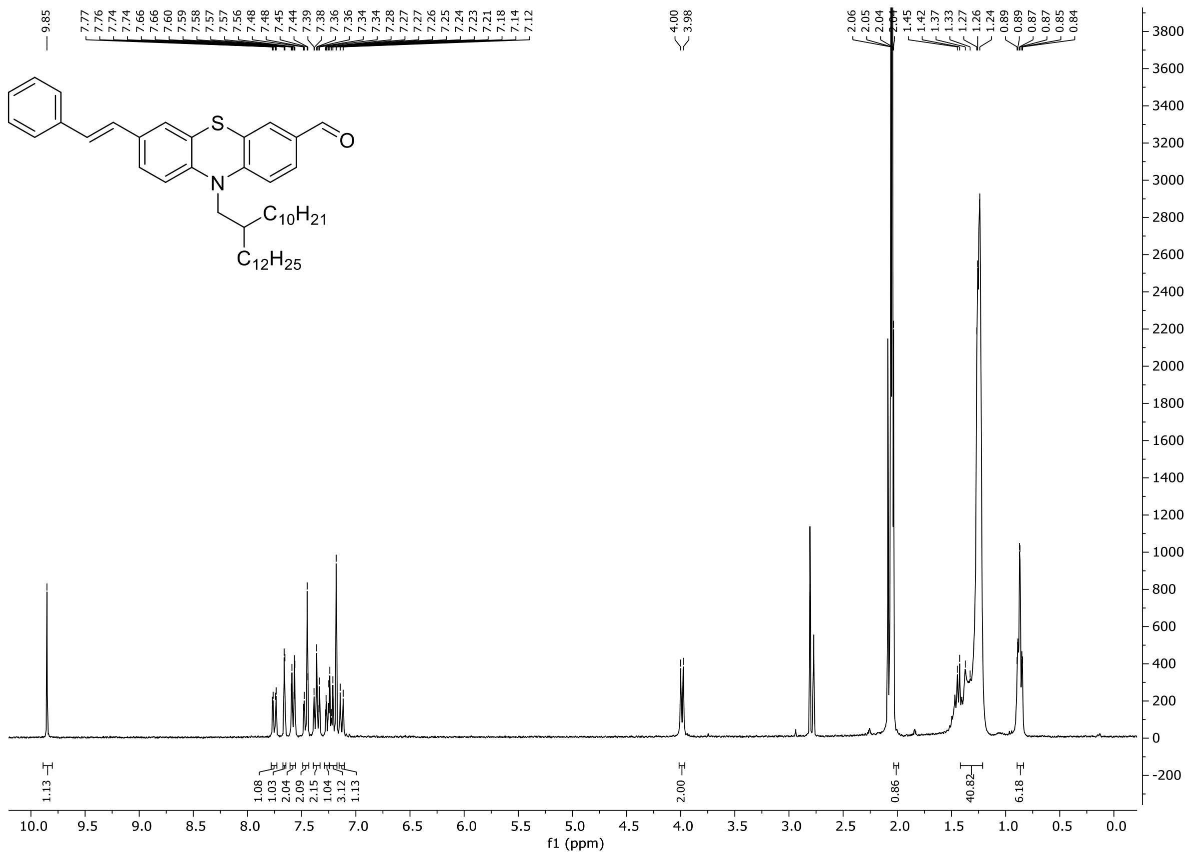


**Figure S149.** $^{1}H$ NMR spectrum of compound **2p** (300 MHz, acetone-d$_6$, $T$ = 293 K).

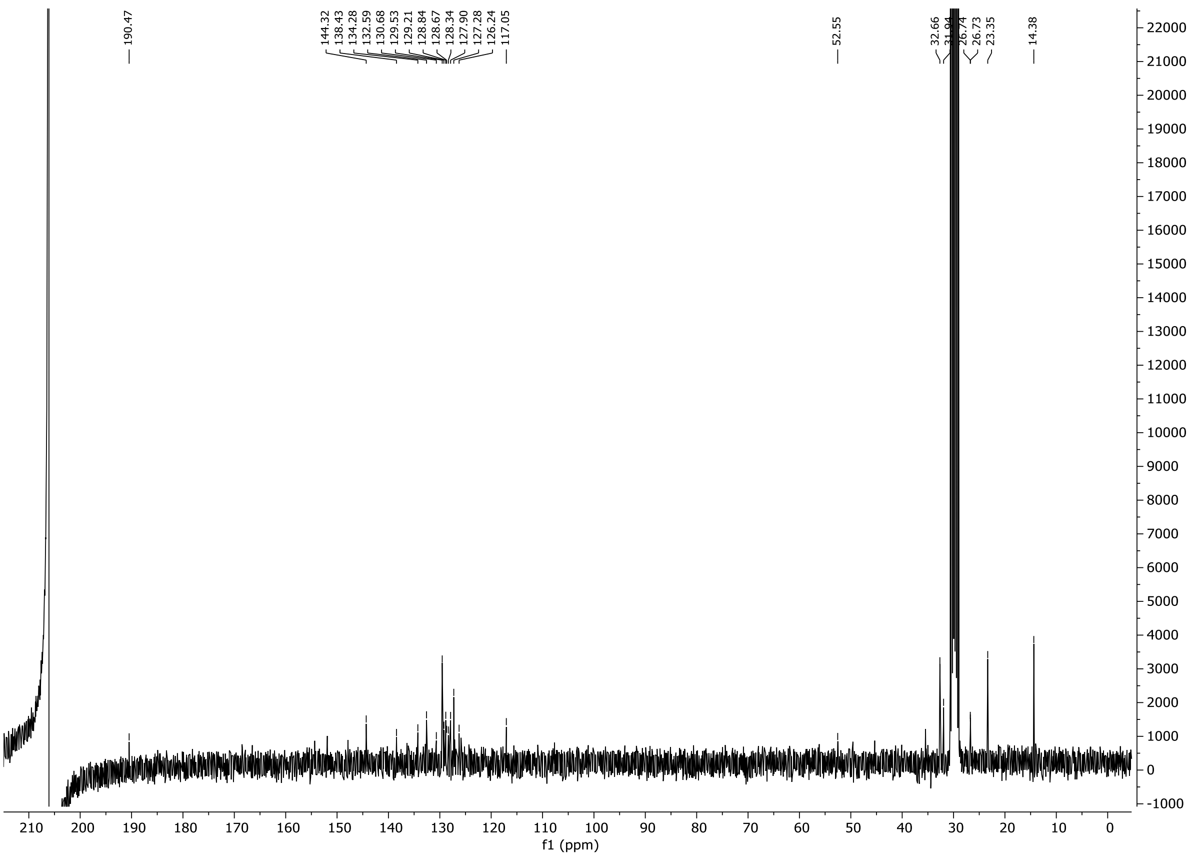

**Figure S150.** $^{13}C$ NMR spectrum of compound **2p** (75 MHz, acetone-$d_6$, $T$ = 293 K).

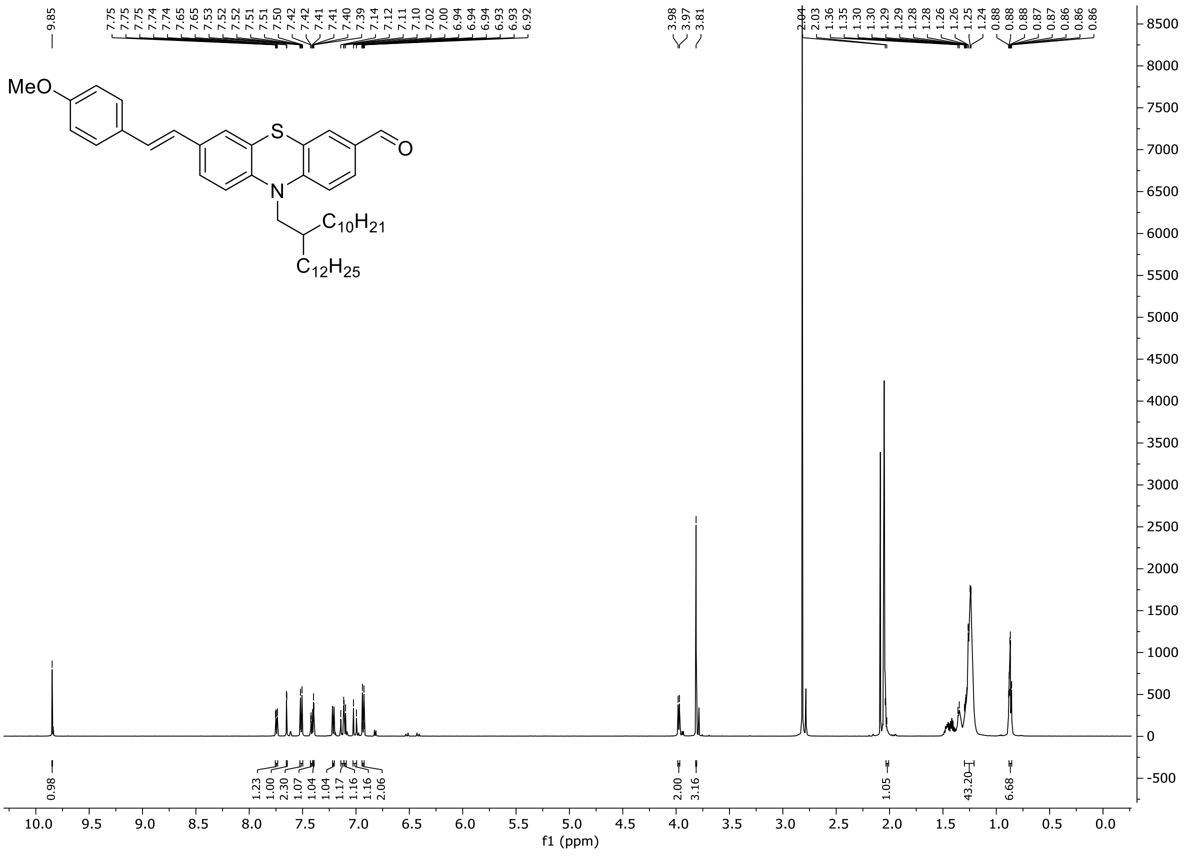

**Figure S151.** $^{1}H$ NMR spectrum of compound **2q** (300 MHz, acetone-$d_6$, $T$ = 293 K).

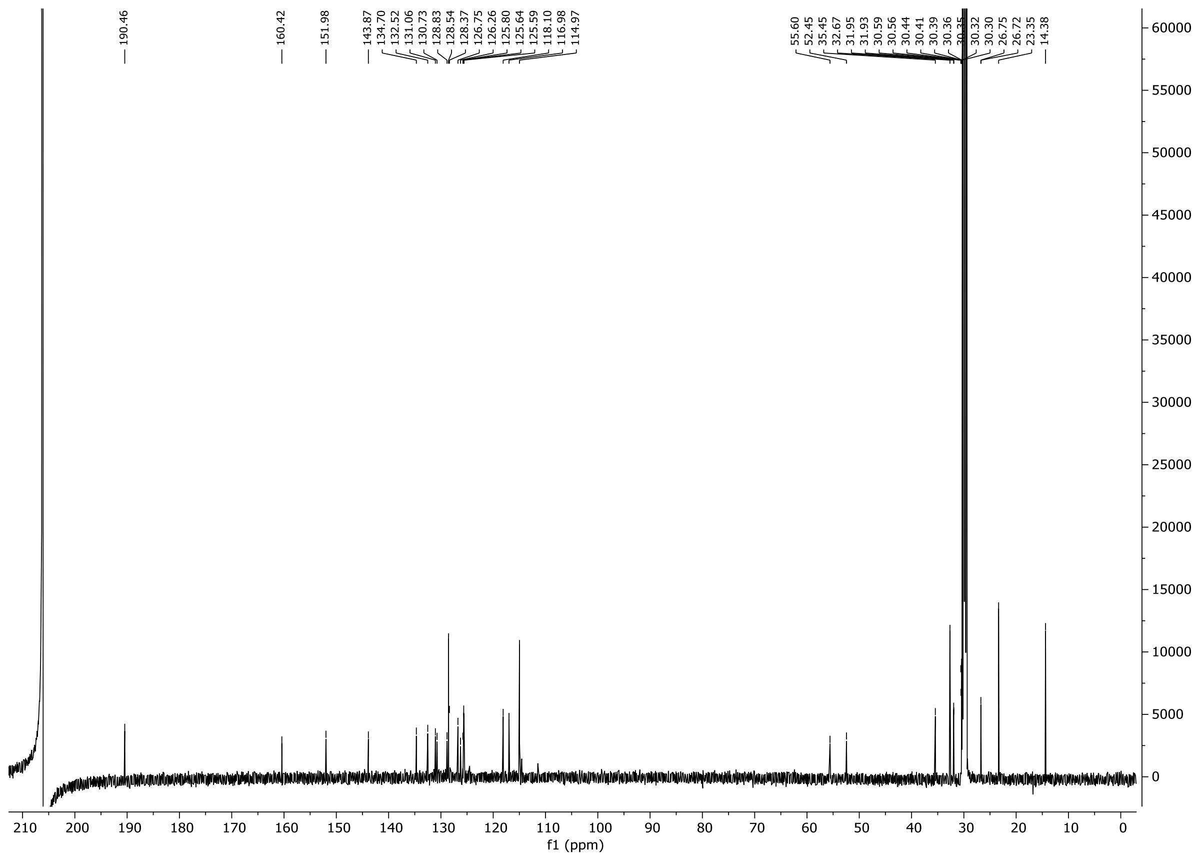

**Figure S152.** $^{13}$C NMR spectrum of compound **2q** (75 MHz, acetone-d$_6$, $T$ = 293 K).

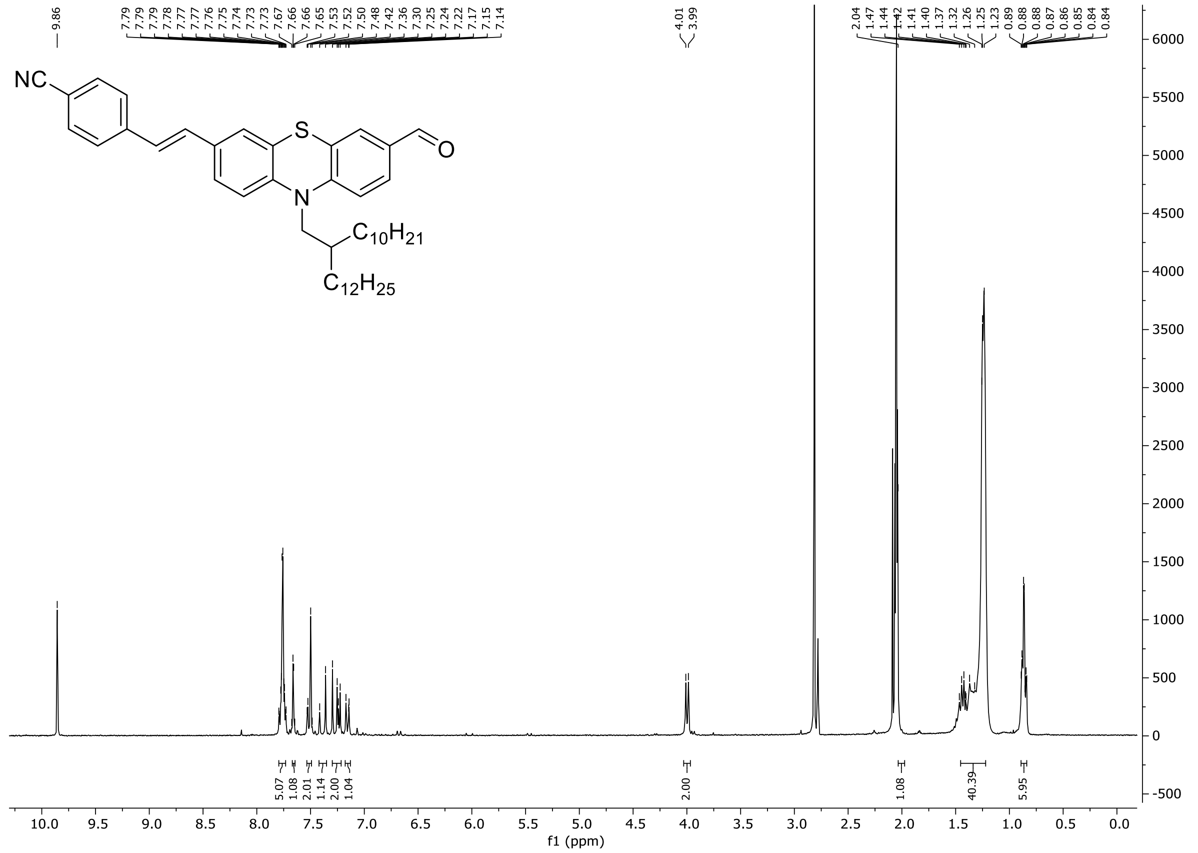


**Figure S153.** $^{1}$H NMR spectrum of compound **2r** (300 MHz, acetone-d$_6$, $T$ = 293 K).

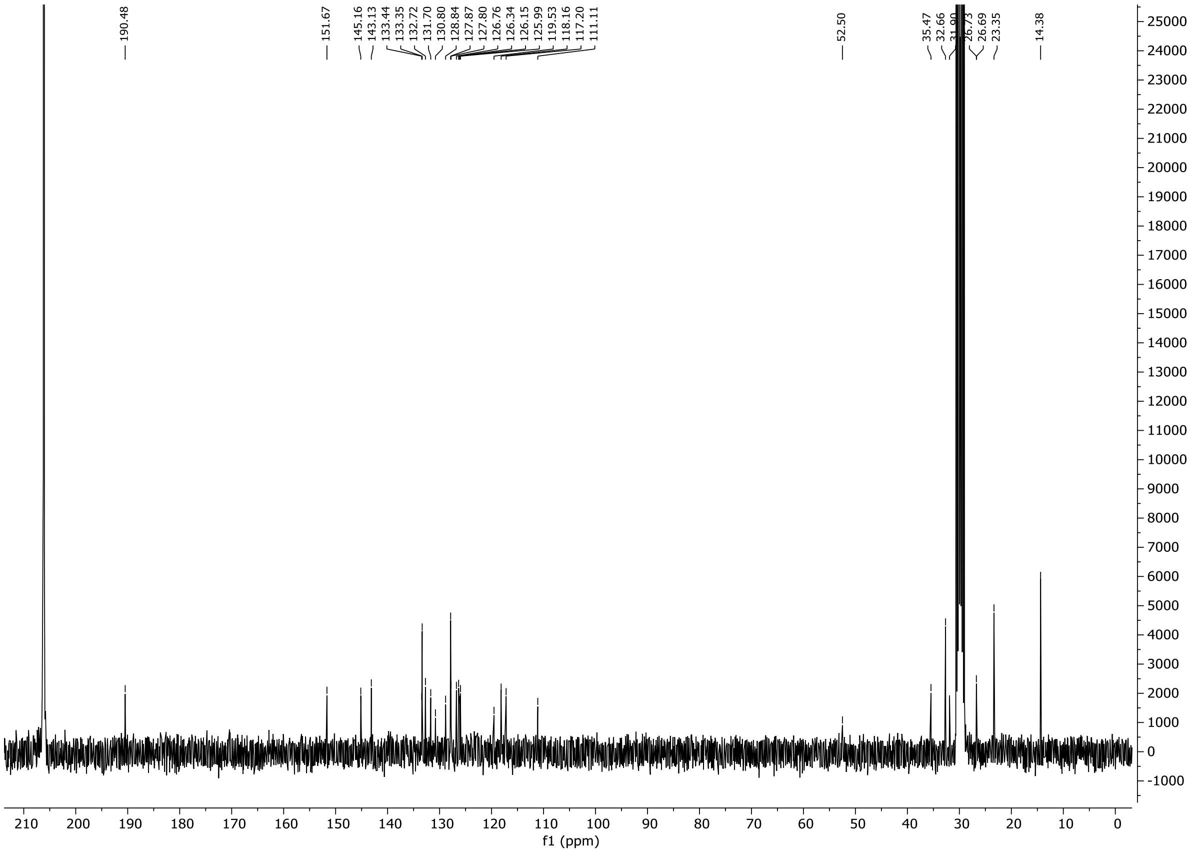

**Figure S154.** $^{13}$C NMR spectrum of compound **2r** (75 MHz, acetone-d$_6$, $T$ = 293 K).

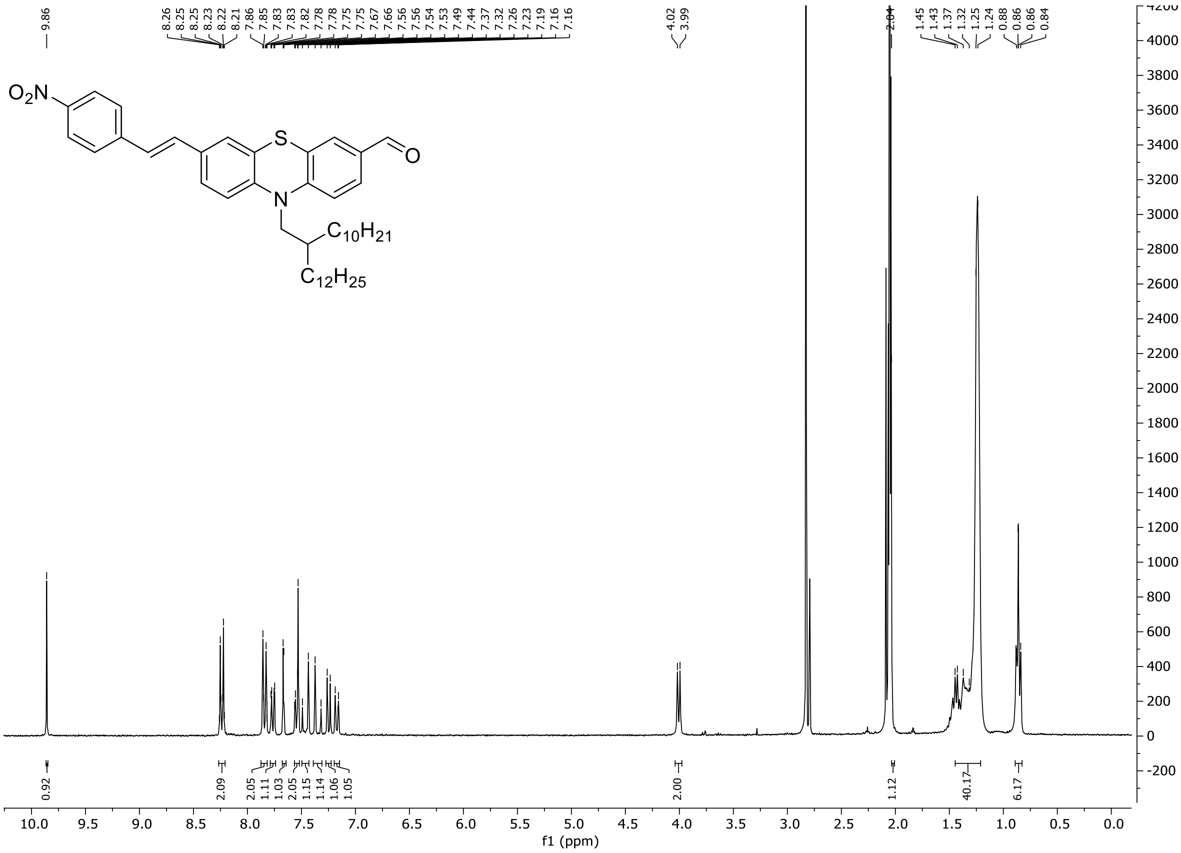

**Figure S155.** $^{1}$H NMR spectrum of compound **2s** (300 MHz, acetone-d$_6$, $T$ = 293 K).

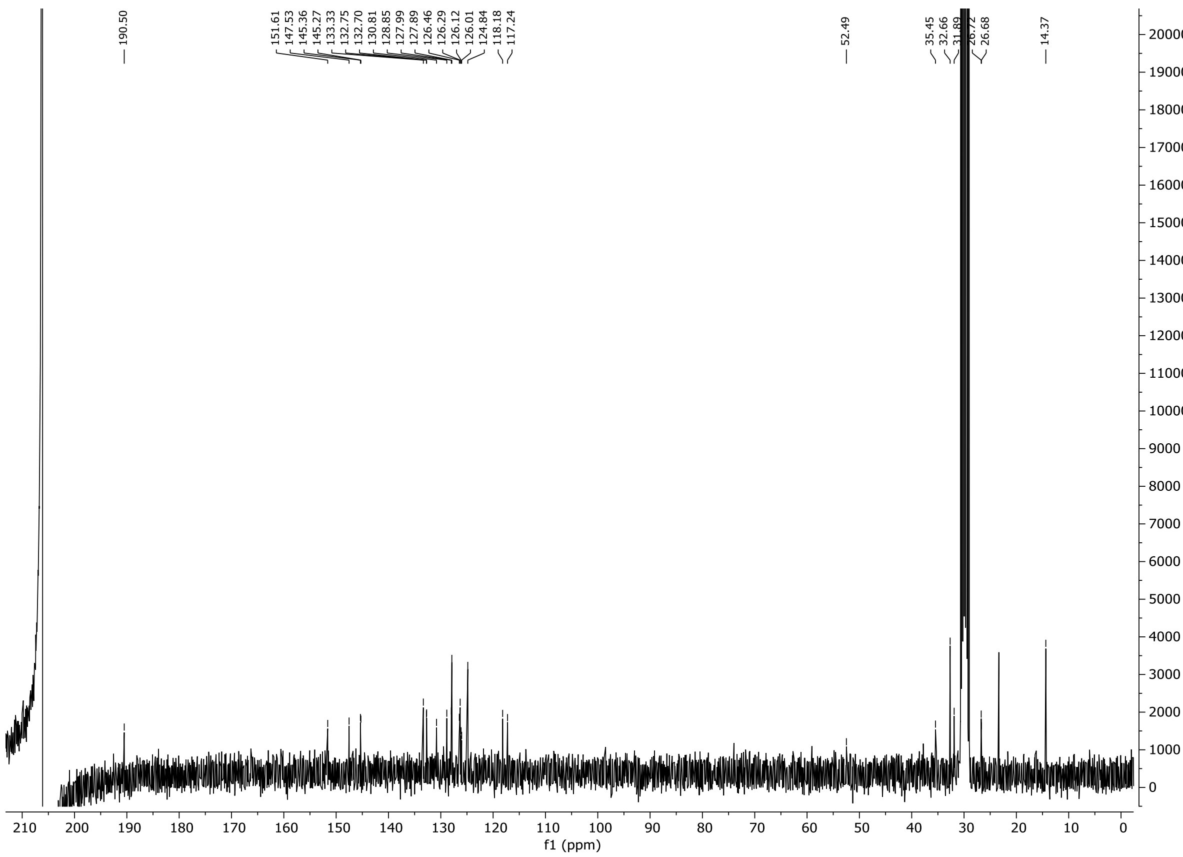

**Figure S156.** $^{13}C$ NMR spectrum of compound **2s** (75 MHz, acetone-d$_6$, *T* = 293 K).

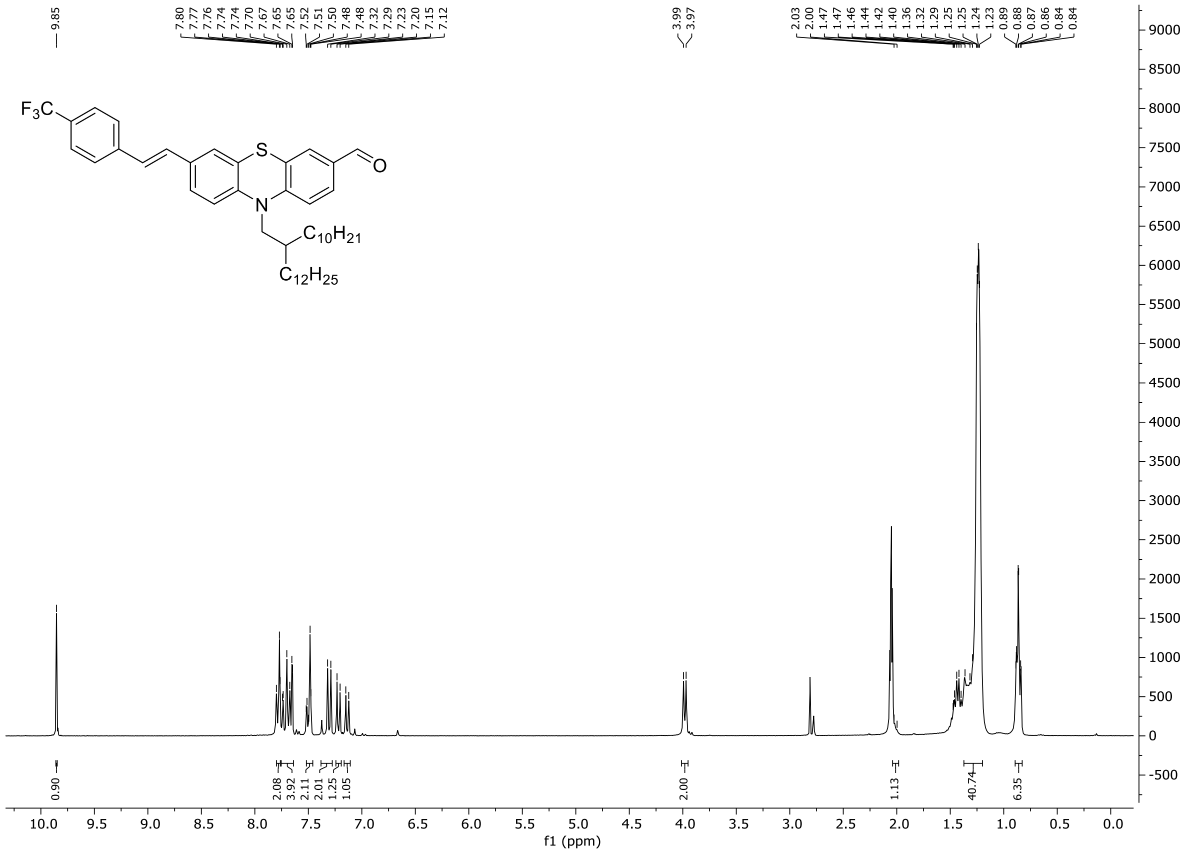

**Figure S157.** $^{1}H$ NMR spectrum of compound **2t** (300 MHz, acetone-d$_6$, *T* = 293 K).

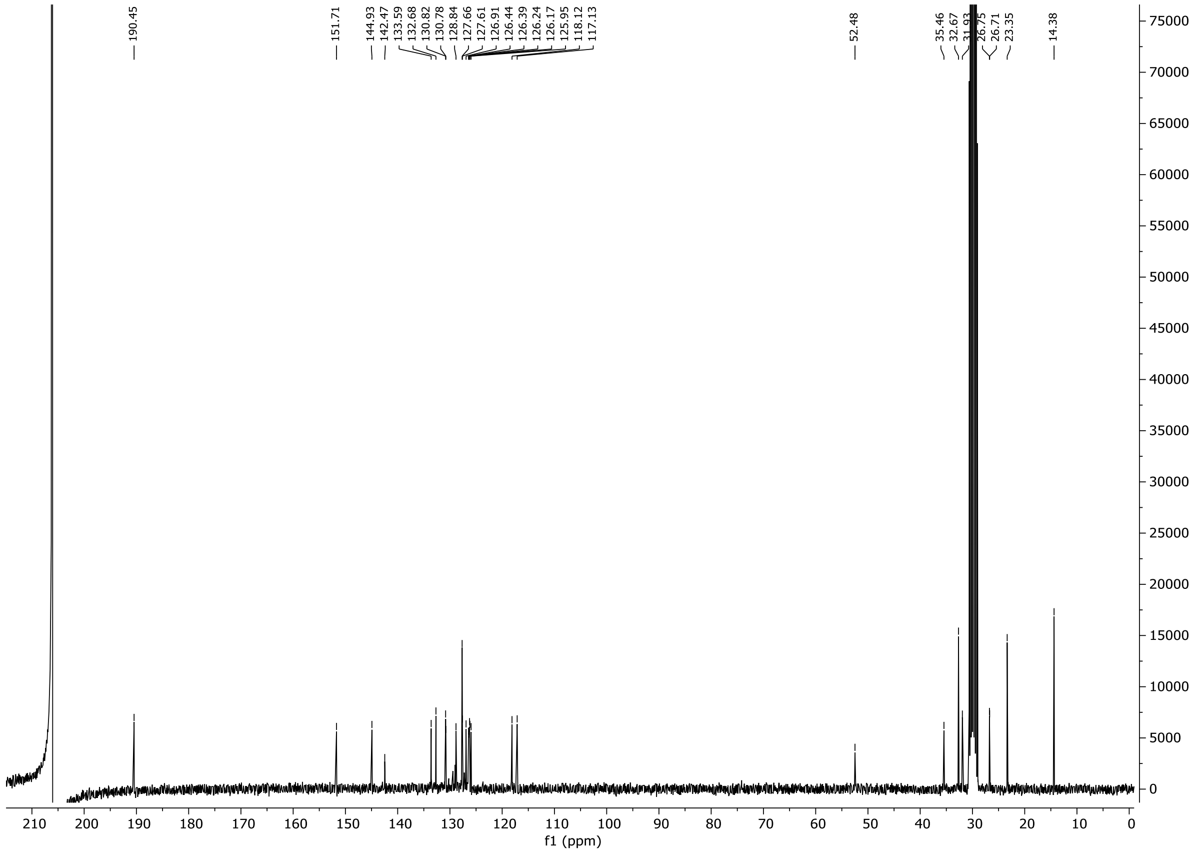

**Figure S158.** $^{13}$C NMR spectrum of compound **2t** (75 MHz, acetone-d$_6$, $T$ = 293 K).

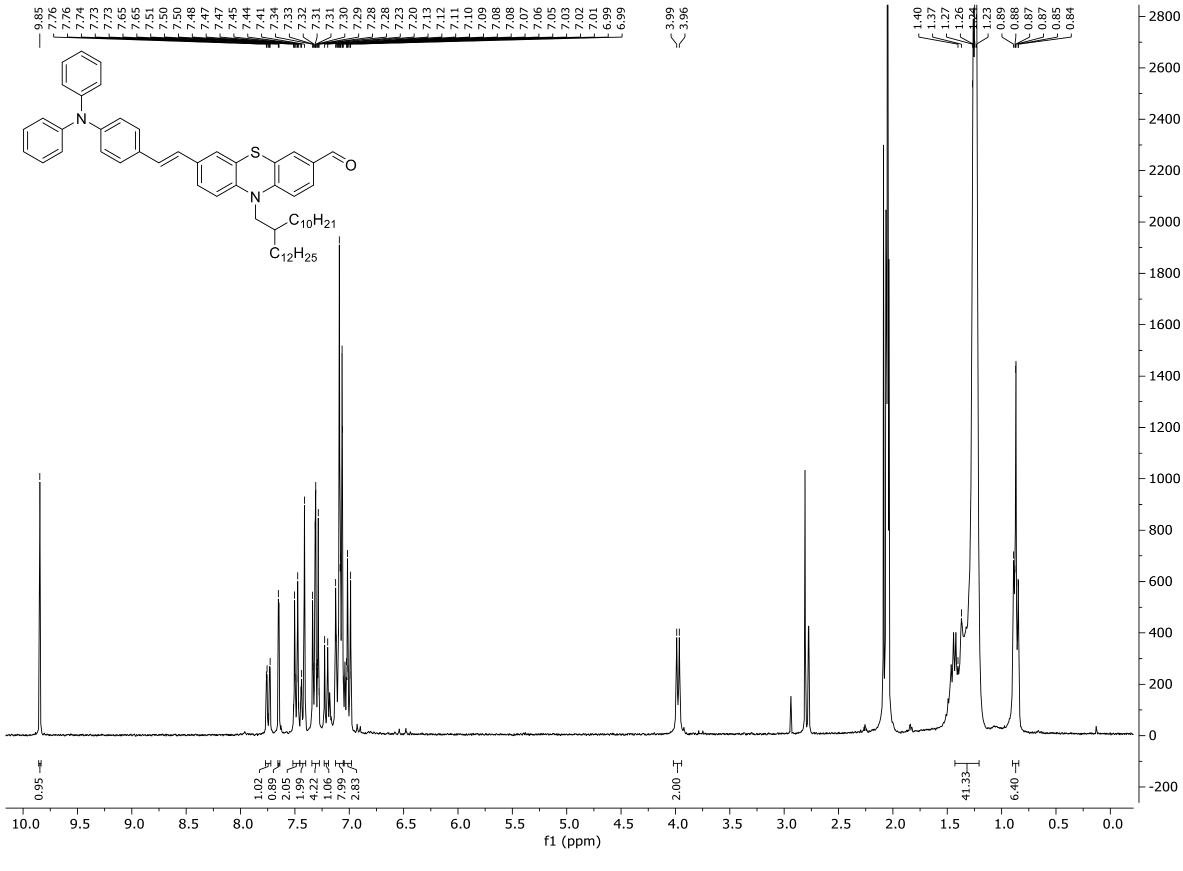


**Figure 159.** $^{1}$H NMR spectrum of compound **2u** (300 MHz, acetone-d$_6$, $T$ = 293 K).

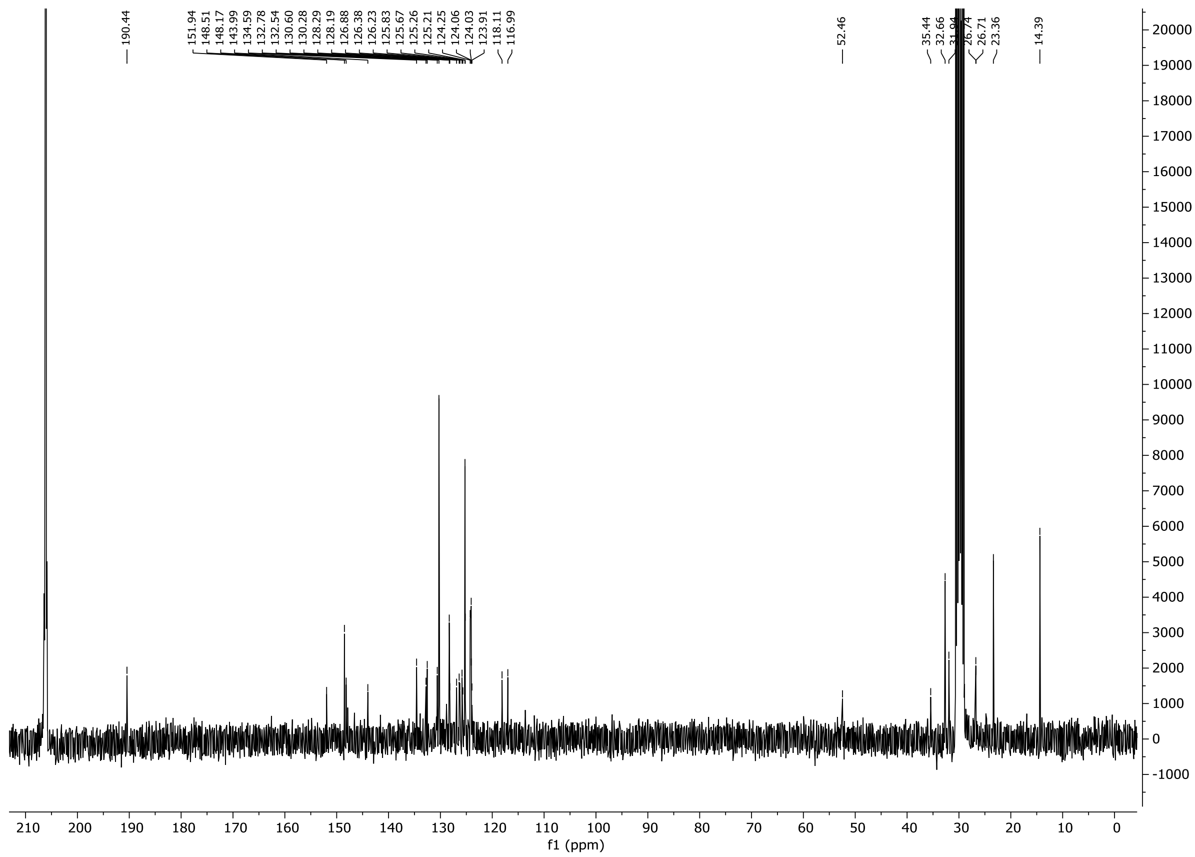

**Figure S160.** $^{13}$C NMR spectrum of compound **2u** (75 MHz, acetone-d$_6$, $T$ = 293 K).

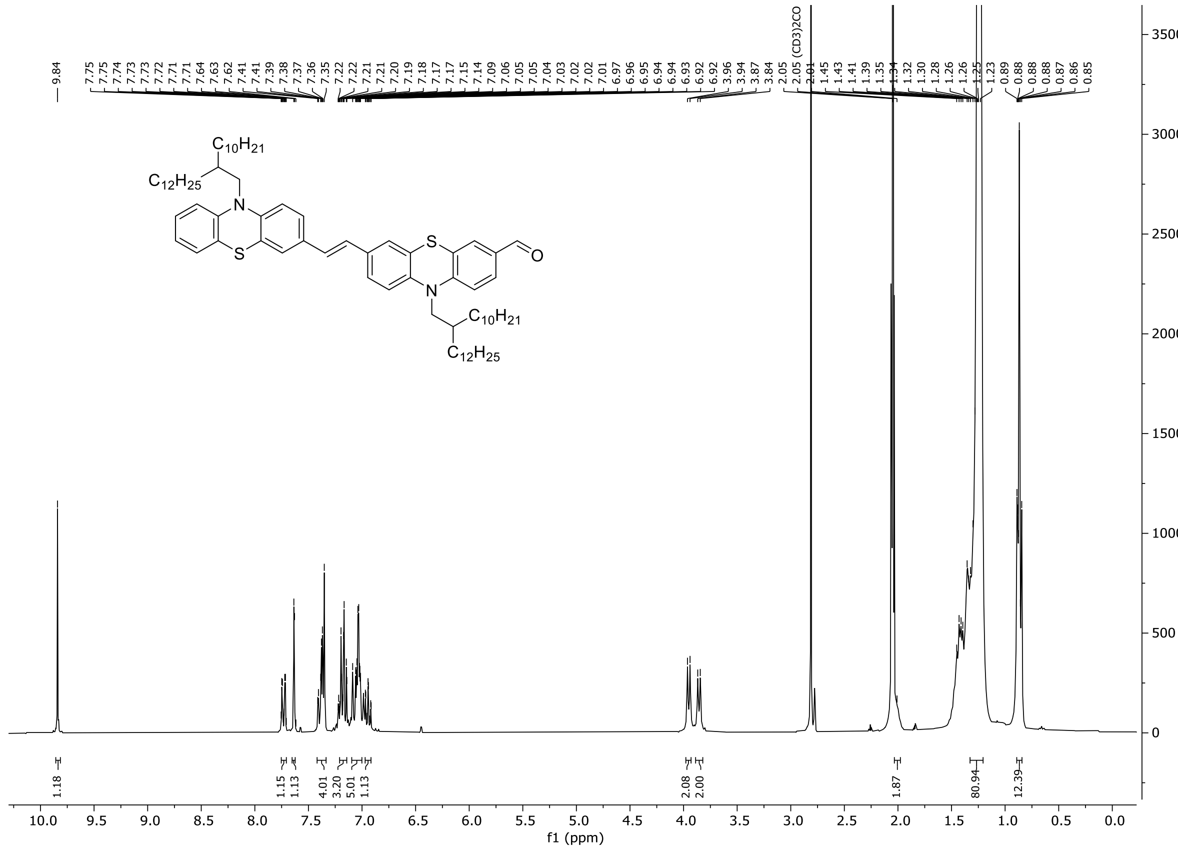

**Figure S161.** $^{1}$H NMR spectrum of compound **2v** (300 MHz, acetone-d$_6$, $T$ = 293 K).

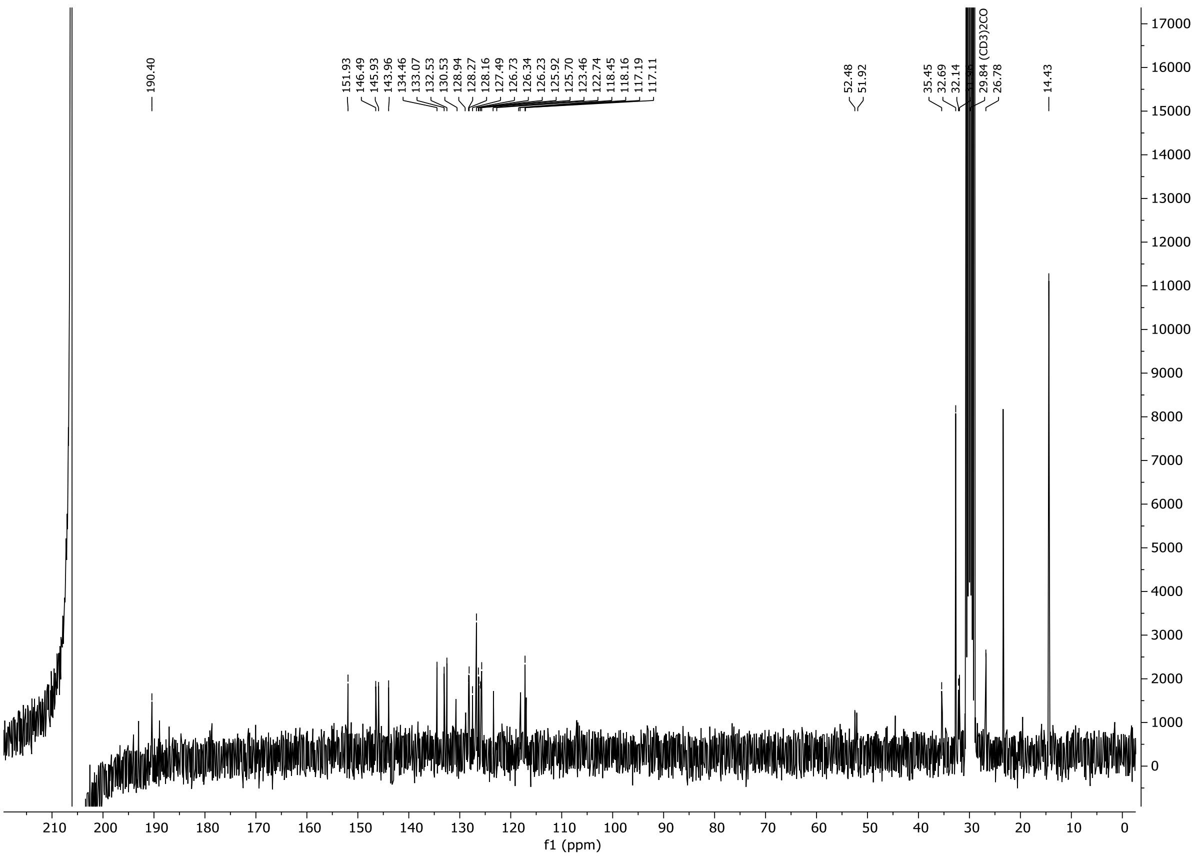

**Figure S162.** $^{13}C$ NMR spectrum of compound **2v** (75 MHz, acetone-$d_6$, $T$ = 293 K).

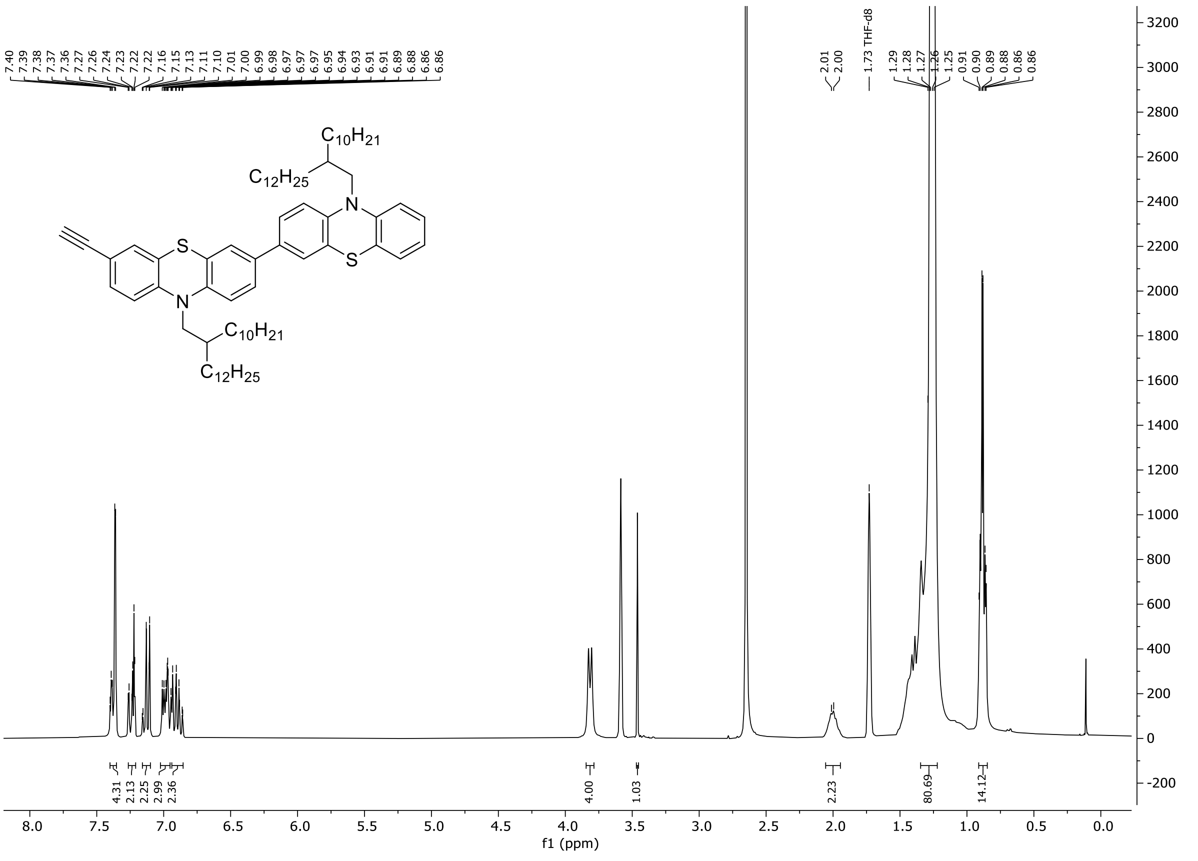

**Figure S163.** $^{1}H$ NMR spectrum of compound **3aa** (300 MHz, THF-$d_8$, $T$ = 293 K).

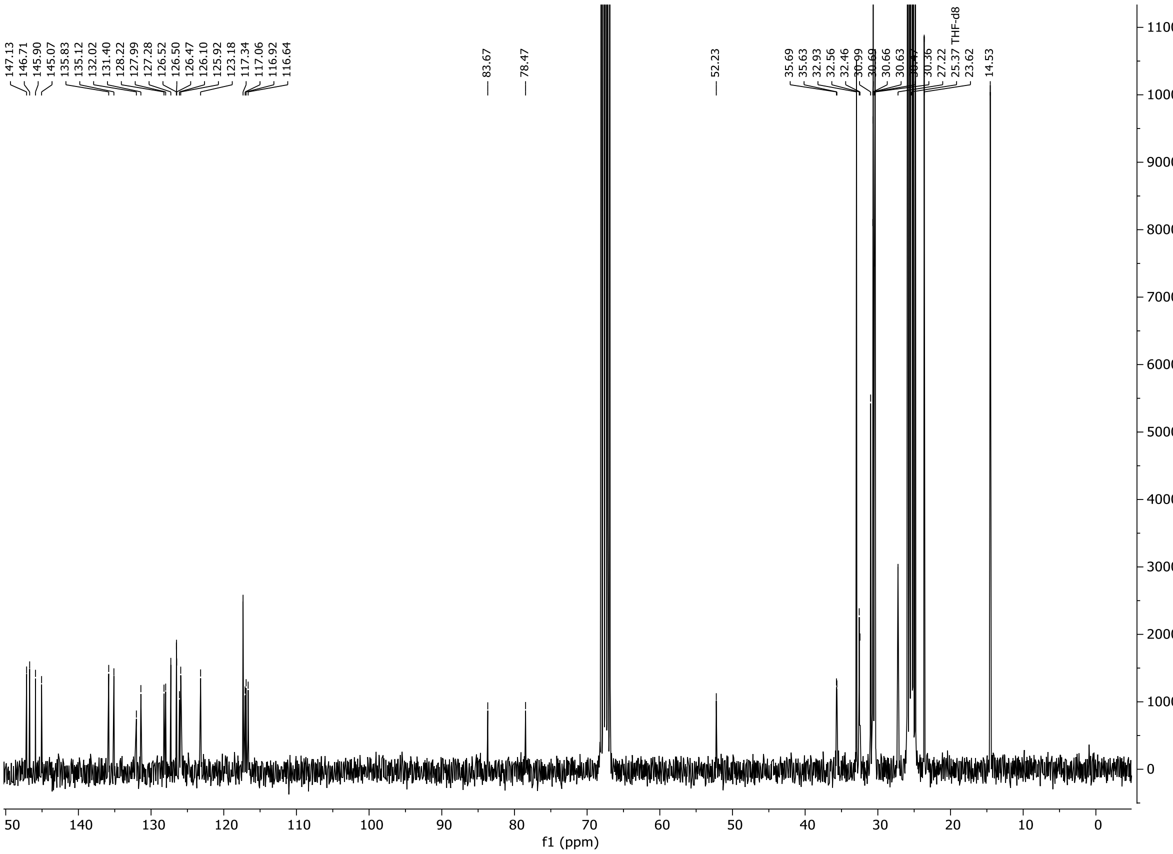


**Figure S164.** $^{13}$C NMR spectrum of compound **3aa** (75 MHz, THF-d$_8$, $T$ = 293 K).

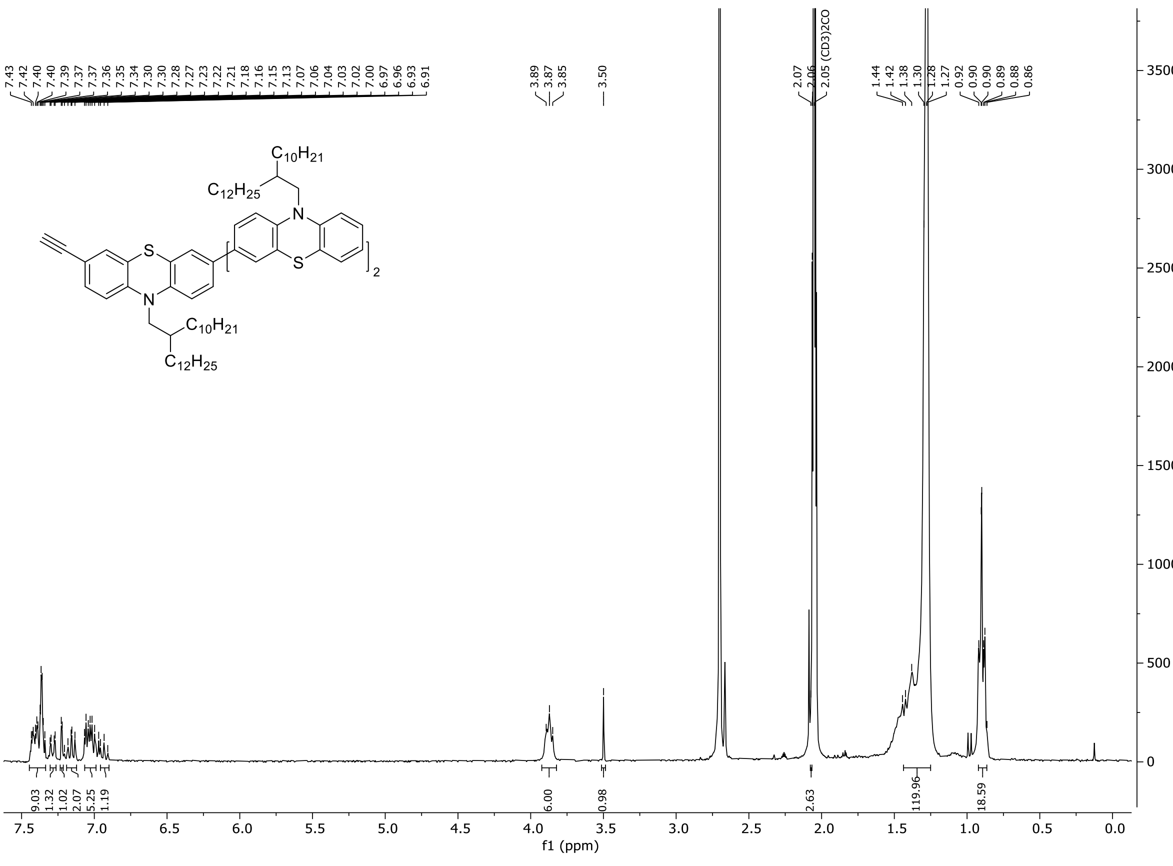


**Figure S165.** $^{1}$H NMR spectrum of compound **3ab** (300 MHz, acetone-d$_6$, $T$ = 293 K).

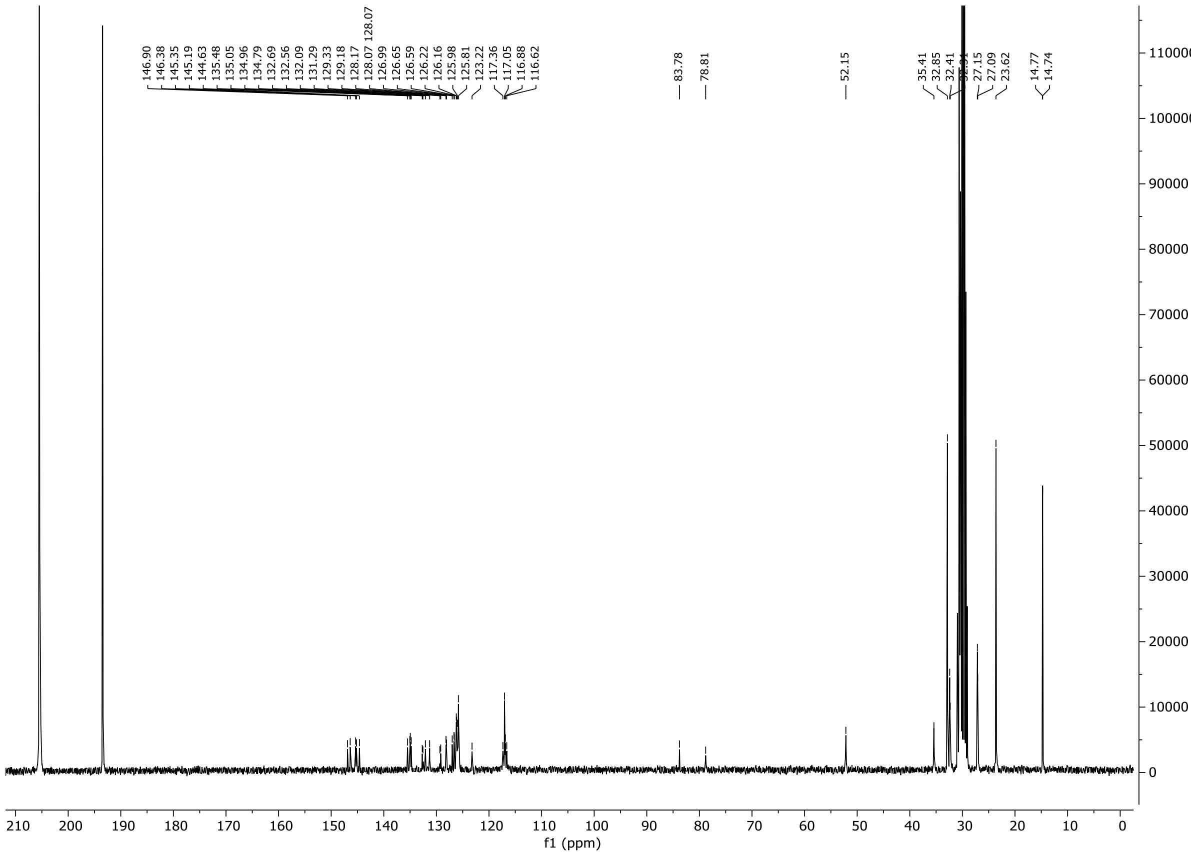


**Figure S166.** $^{13}$C NMR spectrum of compound **3ab** (75 MHz, acetone-d$_6$, *T* = 293 K).

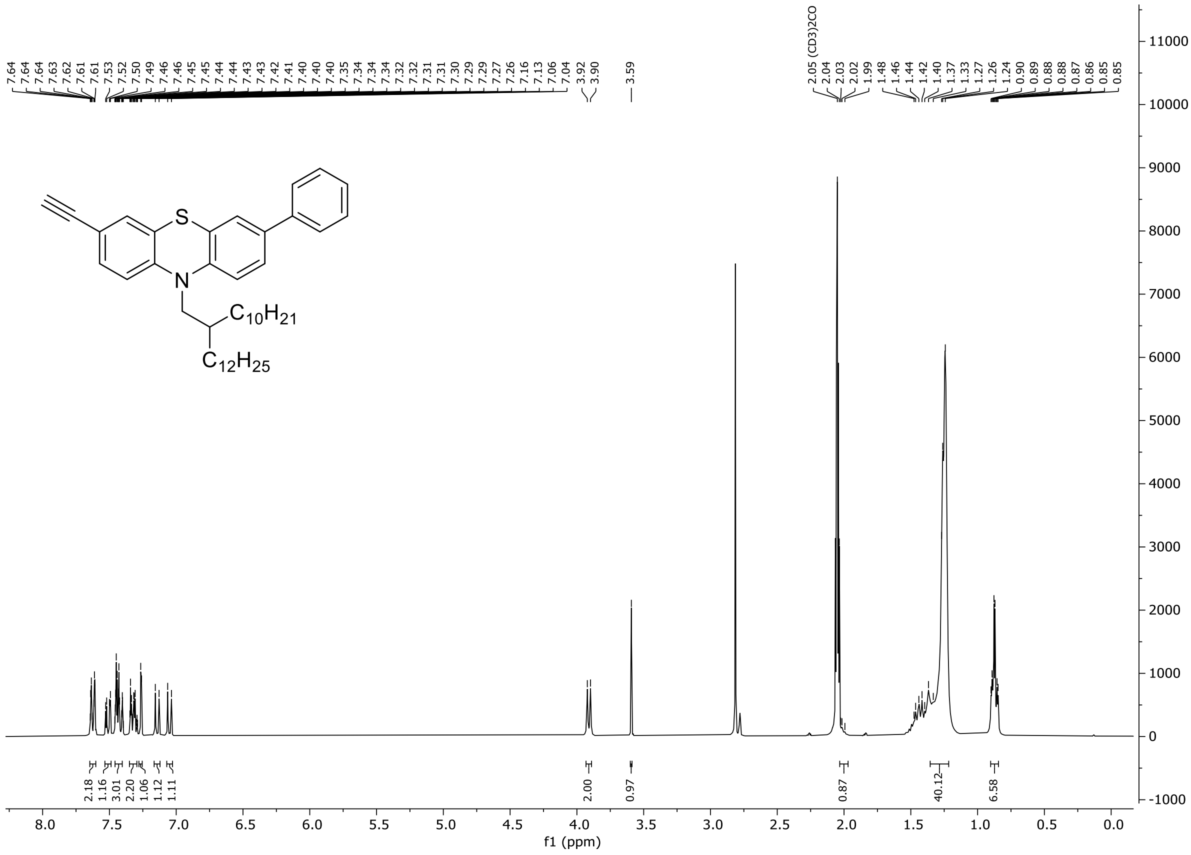


**Figure S167.** $^{1}$H NMR spectrum of compound **3a** (300 MHz, acetone-d$_6$, *T* = 293 K).

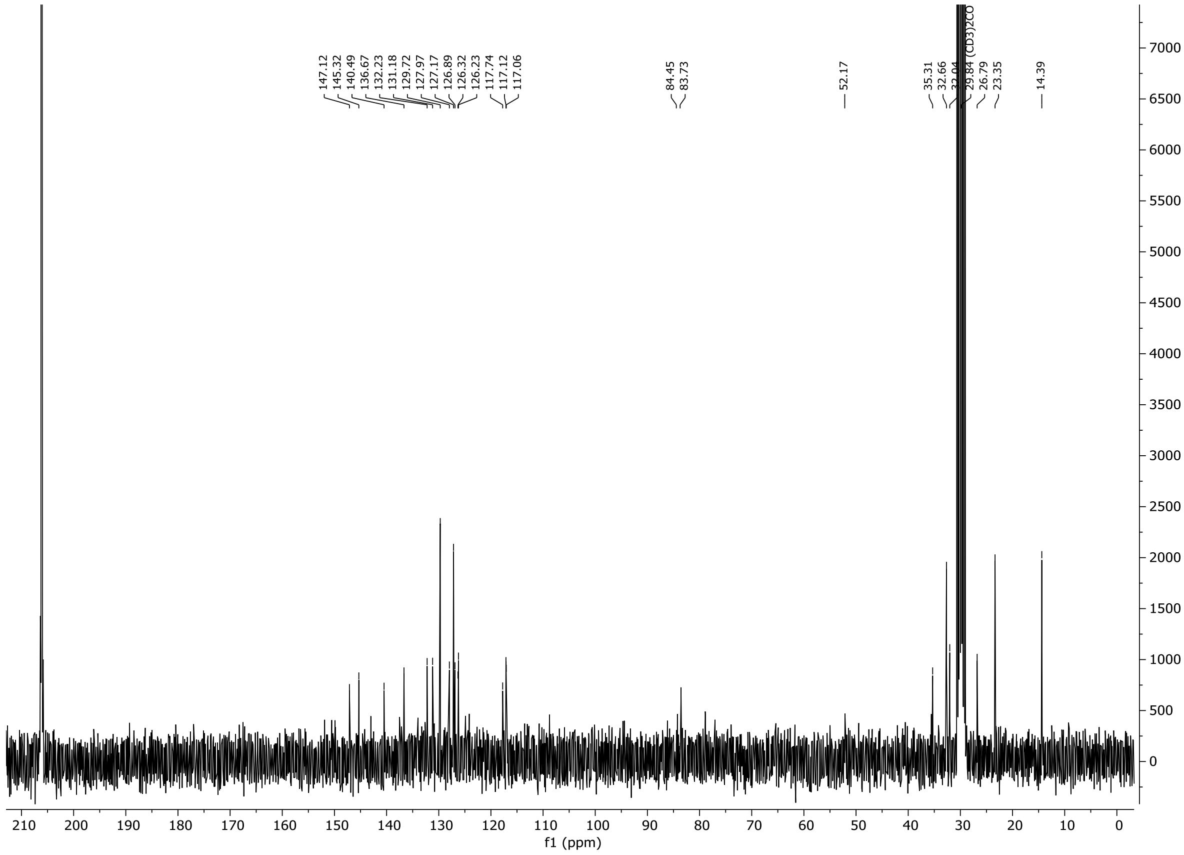

**Figure S168.** $^{13}C$ NMR spectrum of compound **3a** (75 MHz, acetone-$d_6$, $T$ = 293 K).

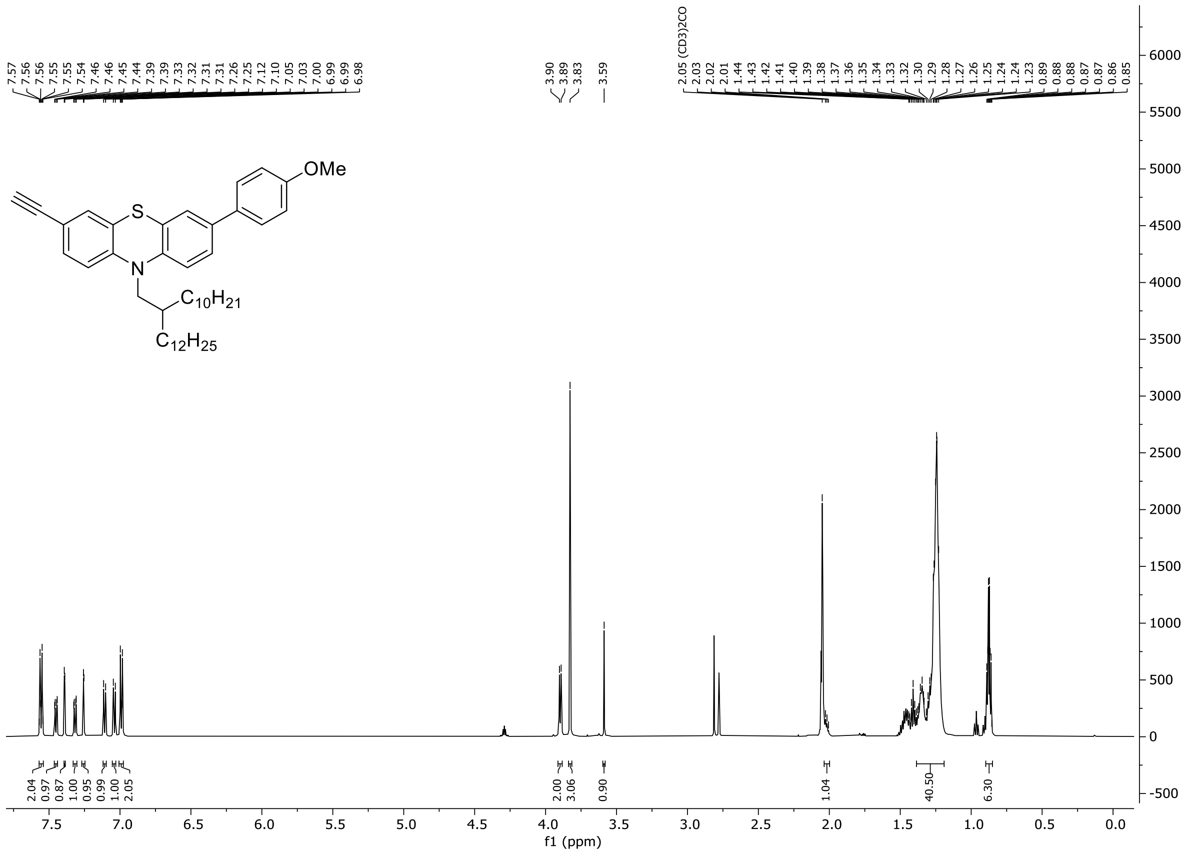

**Figure S169.** $^{1}H$ NMR spectrum of compound **3b** (300 MHz, acetone-$d_6$, $T$ = 293 K).

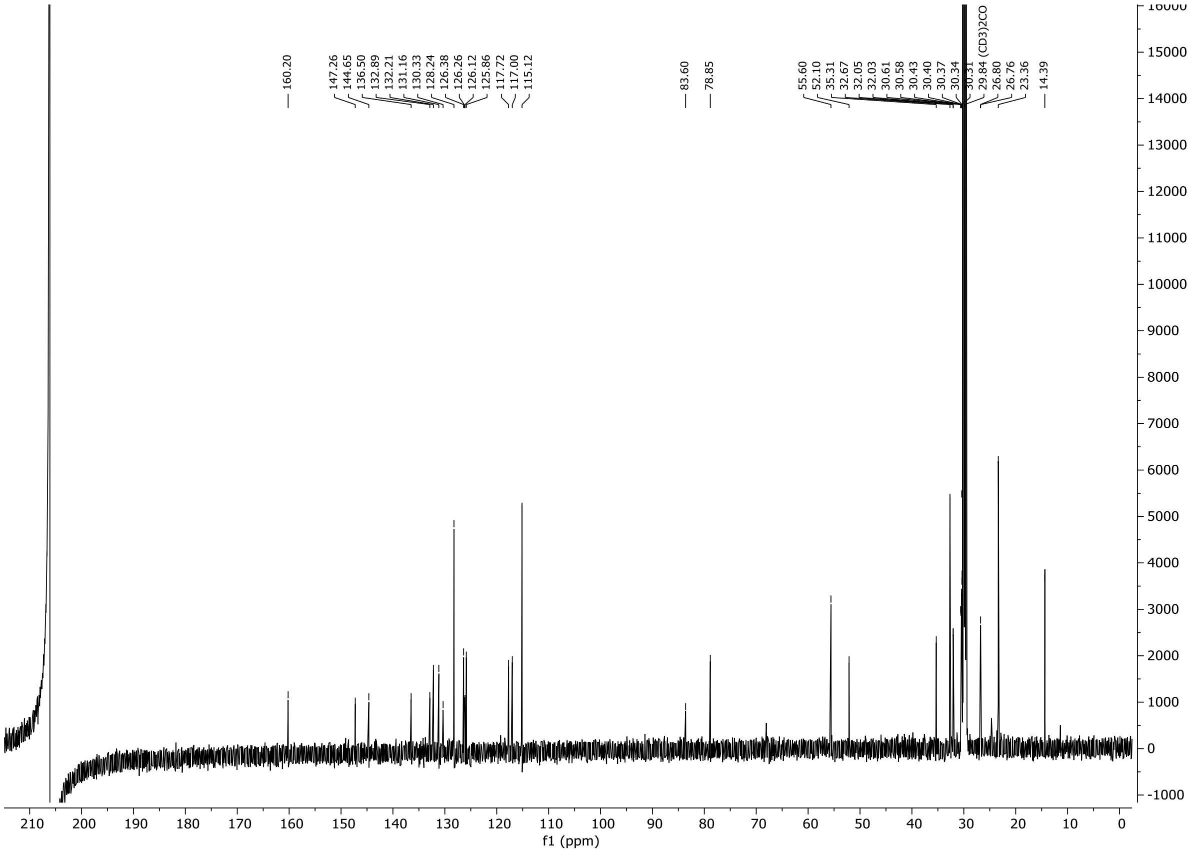


**Figure S170.** $^{13}C$ NMR spectrum of compound **3b** (75 MHz, acetone-d$_6$, $T$ = 293 K).

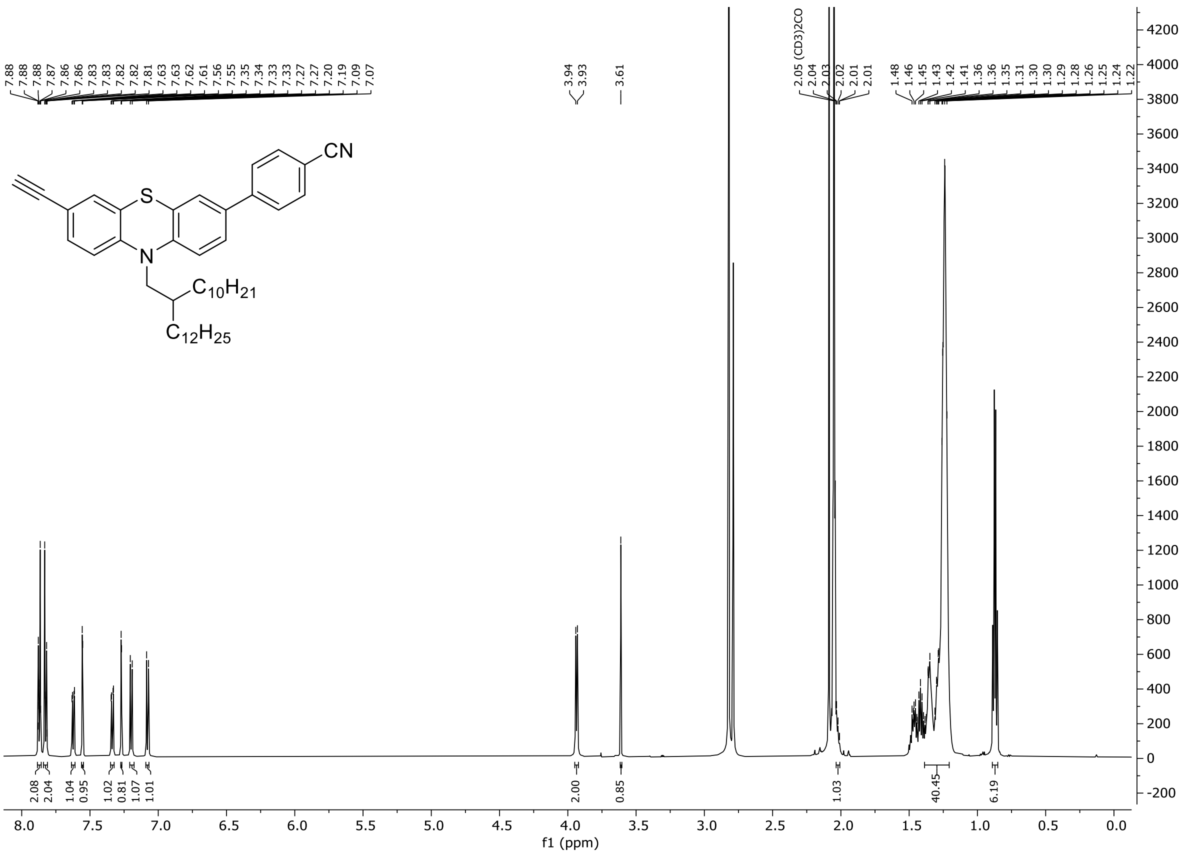


**Figure S171.** $^{1}H$ NMR spectrum of compound **3c** (300 MHz, acetone-d$_6$, $T$ = 293 K).

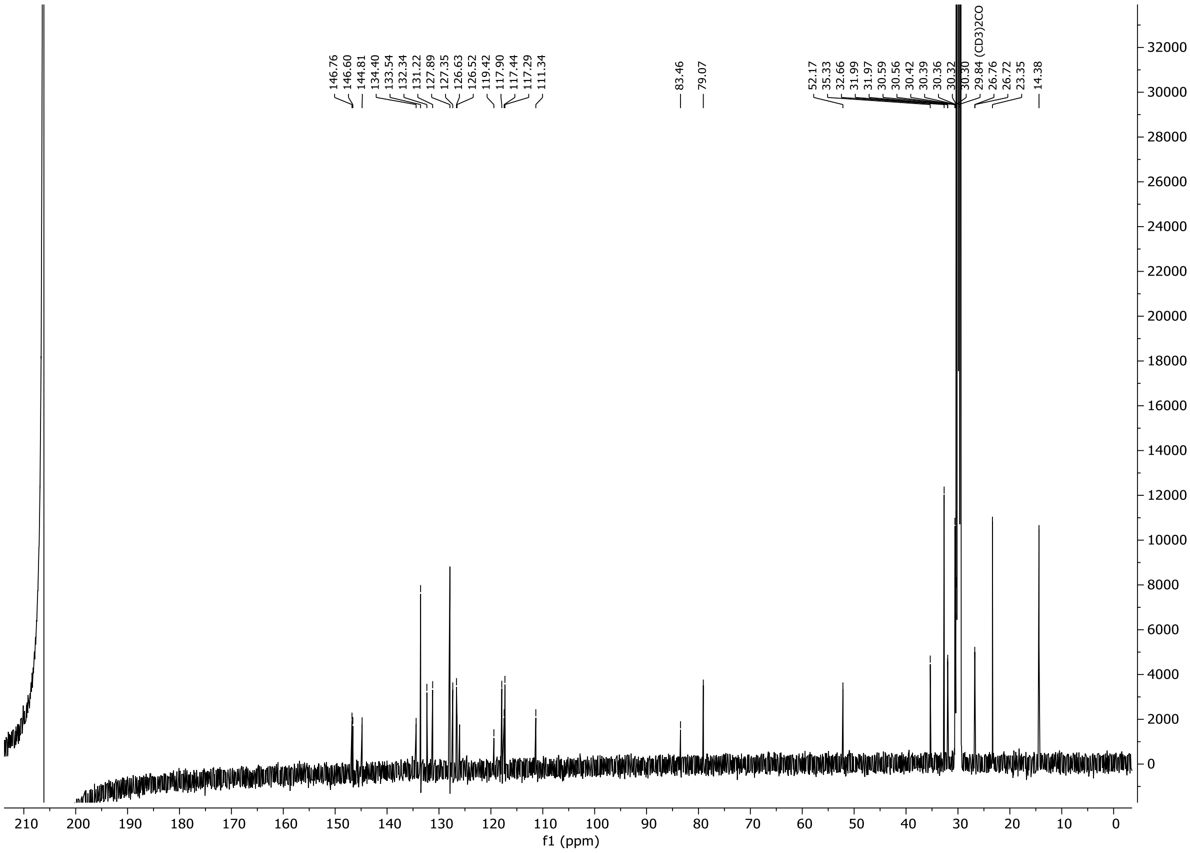

**Figure S172.** $^{13}$C NMR spectrum of compound **3c** (75 MHz, acetone-d$_6$, $T$ = 293 K).

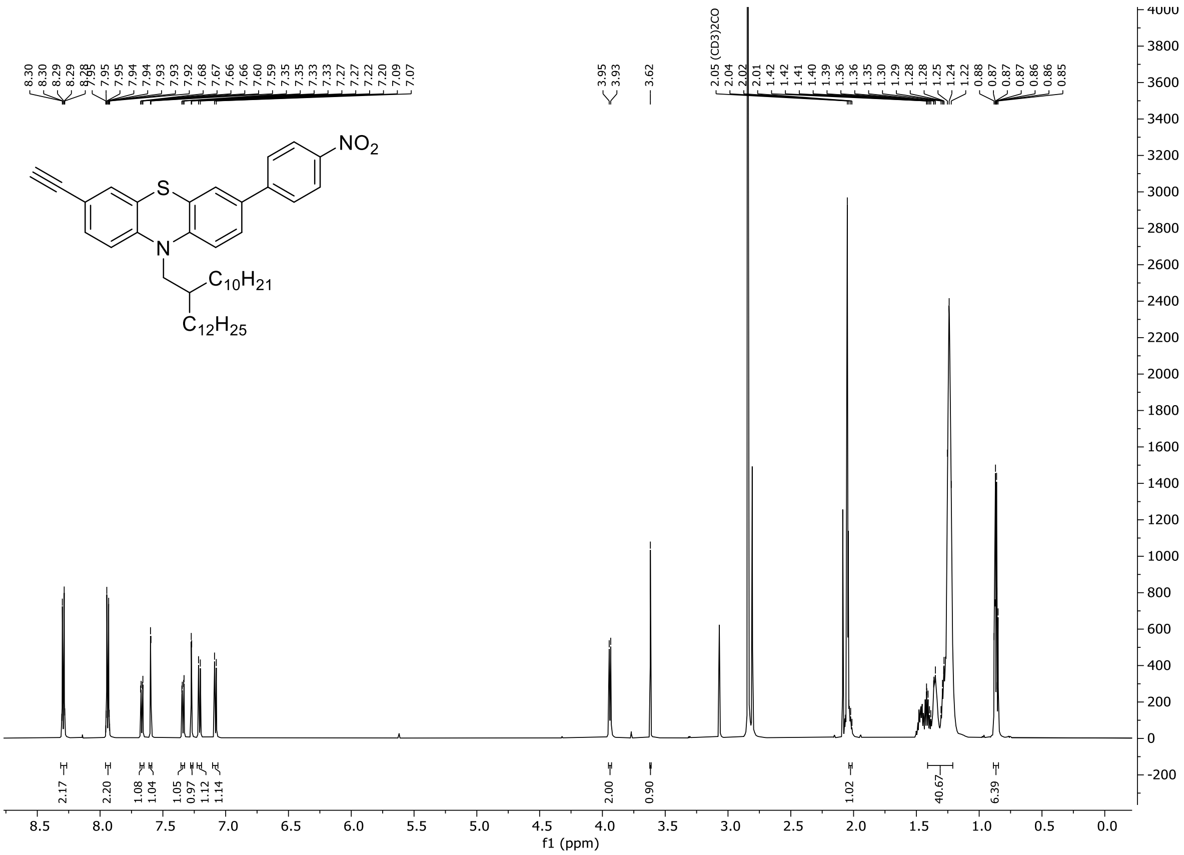

**Figure S173.** $^{1}$H NMR spectrum of compound **3d** (300 MHz, acetone-d$_6$, $T$ = 293 K).

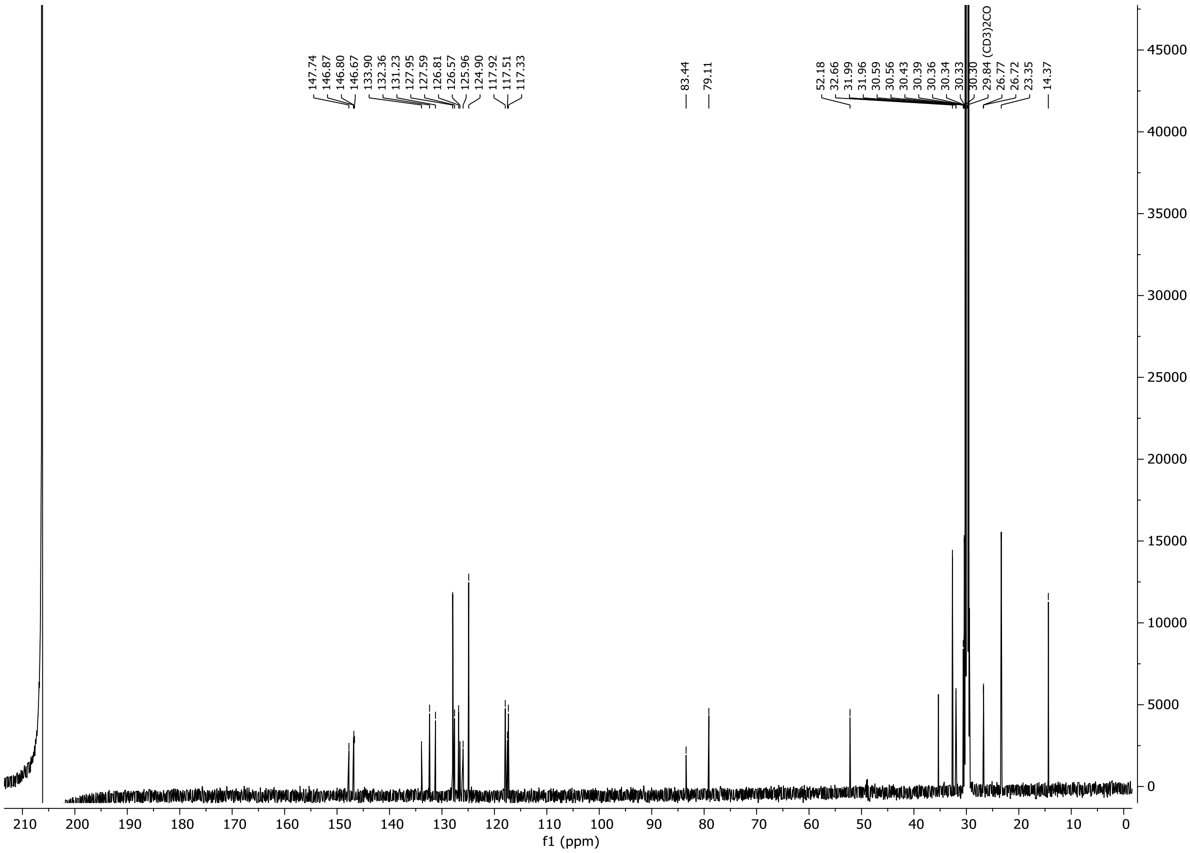


**Figure S174.** $^{13}C$ NMR spectrum of compound **3d** (75 MHz, acetone-d6, *T* = 293 K).

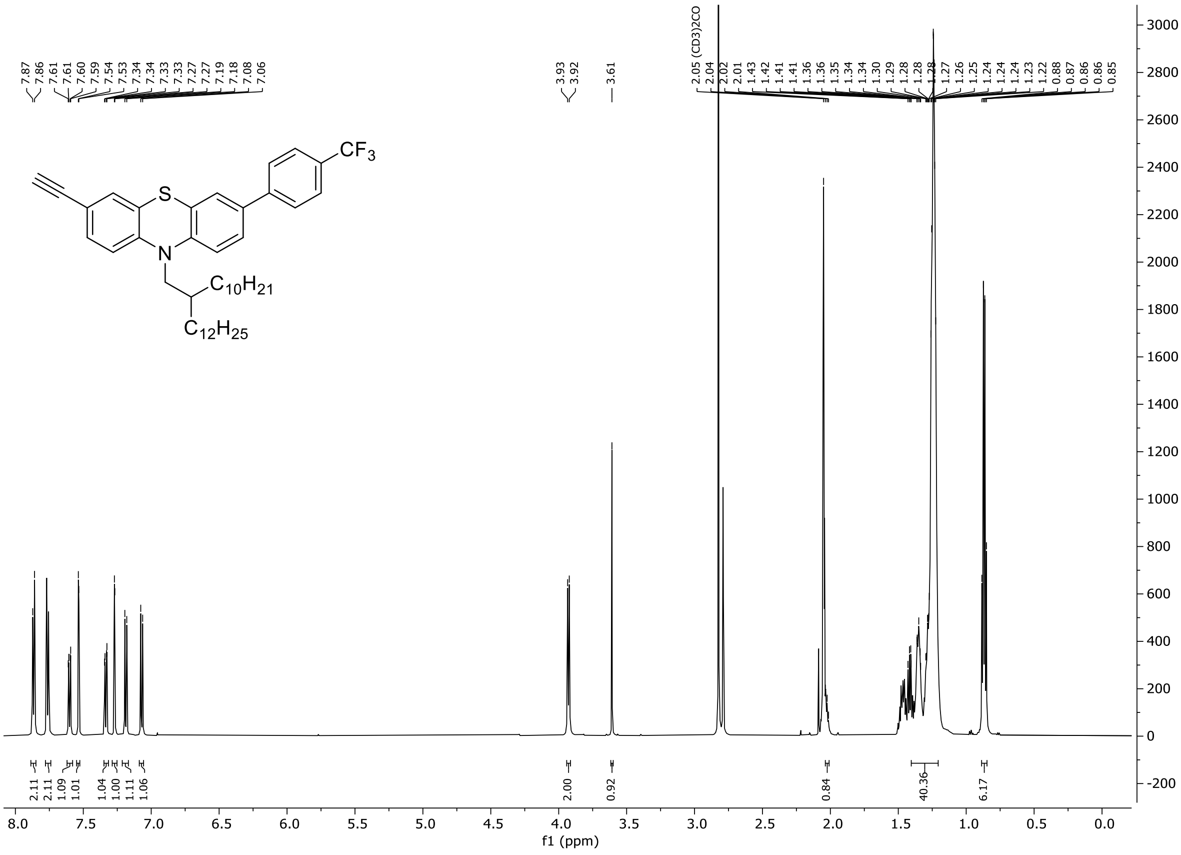


**Figure S175.** $^{1}H$ NMR spectrum of compound **3e** (300 MHz, acetone-d6, *T* = 293 K).

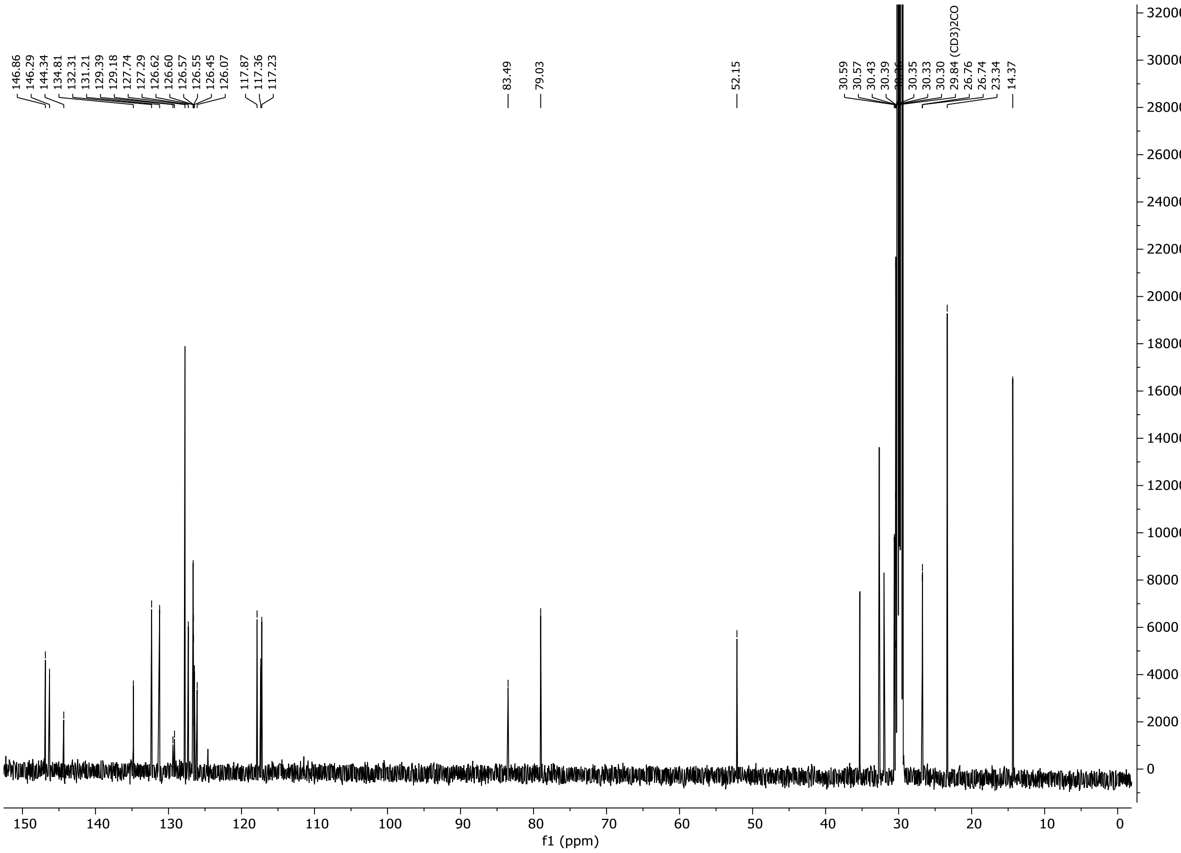

**Figure S176.** $^{13}C$ NMR spectrum of compound **3e** (75 MHz, acetone-d$_6$, $T$ = 293 K).

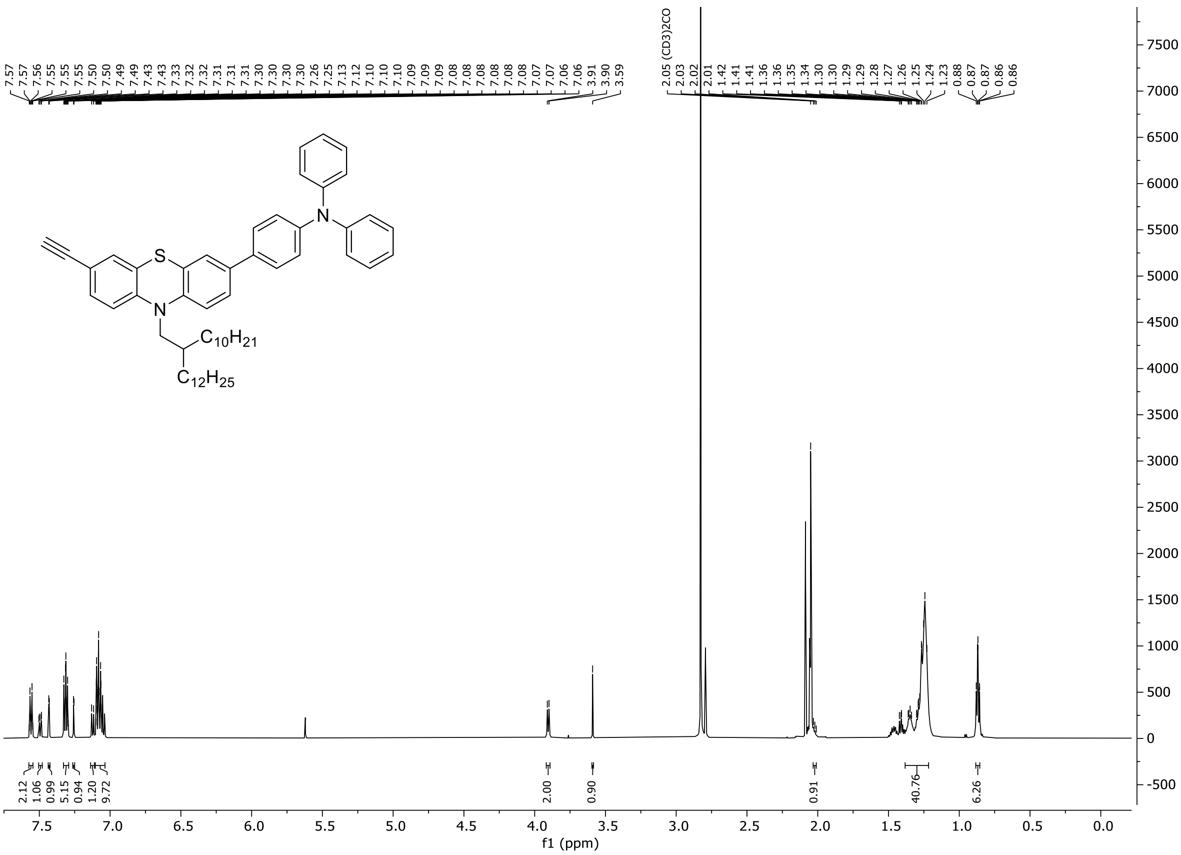


**Figure S177.** $^{1}H$ NMR spectrum of compound **3f** (300 MHz, acetone-d$_6$, $T$ = 293 K).

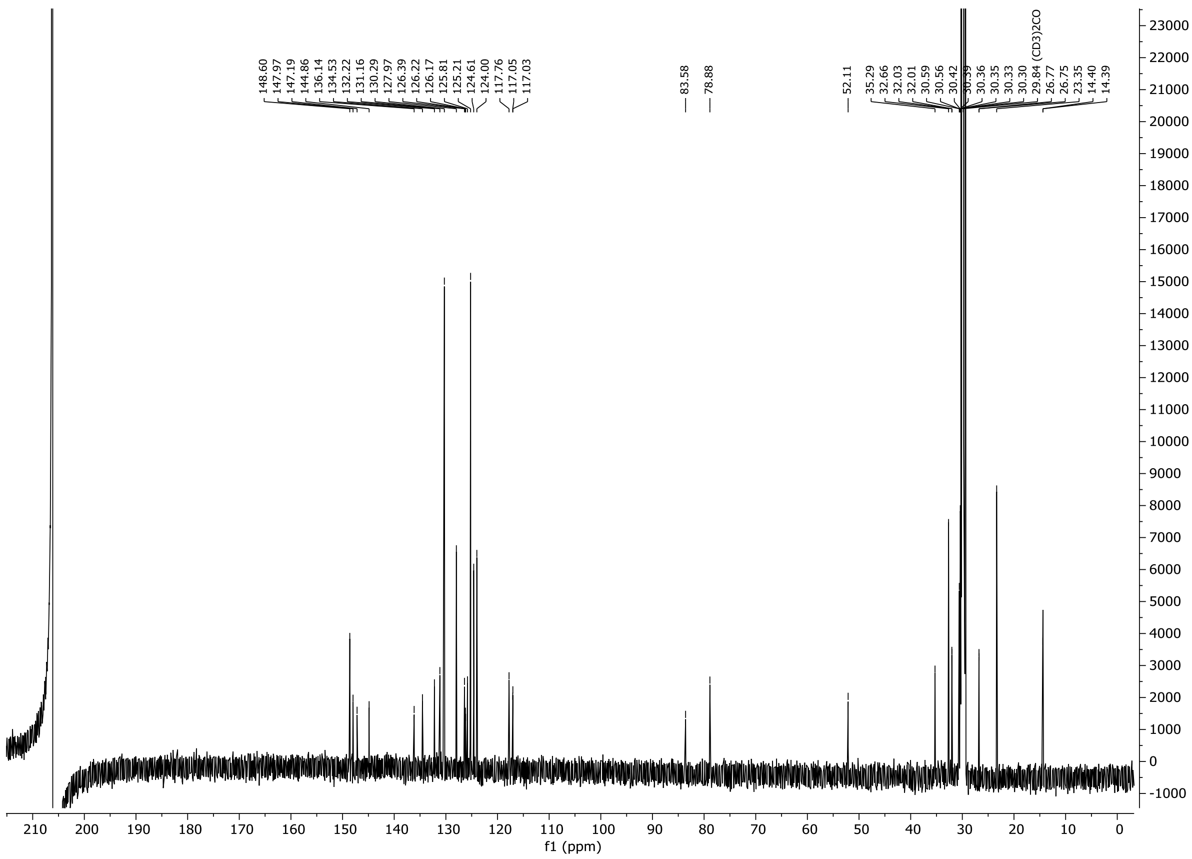

**Figure S178.** $^{13}$C NMR spectrum of compound **3f** (75 MHz, acetone-d$_6$, $T$ = 293 K).

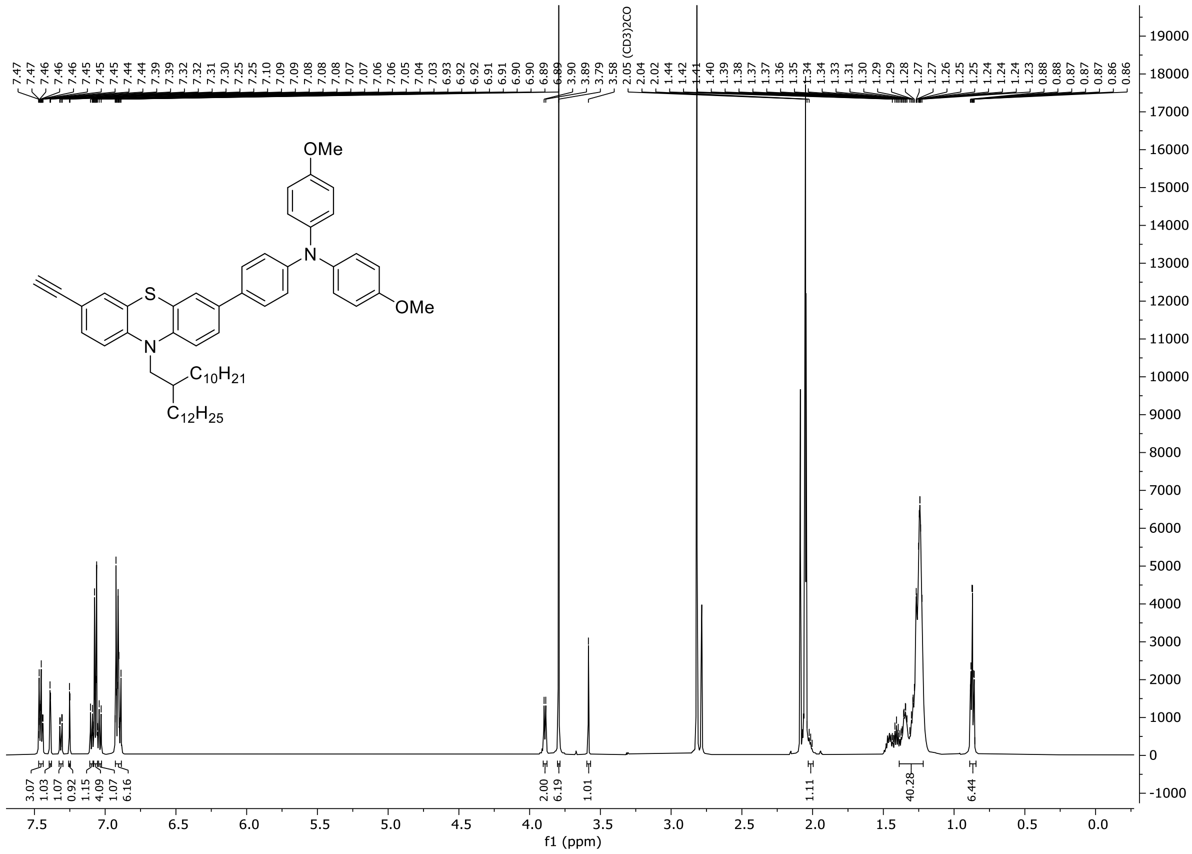

**Figure S179.** $^{1}$H NMR spectrum of compound **3g** (300 MHz, acetone-d$_6$, $T$ = 293 K).

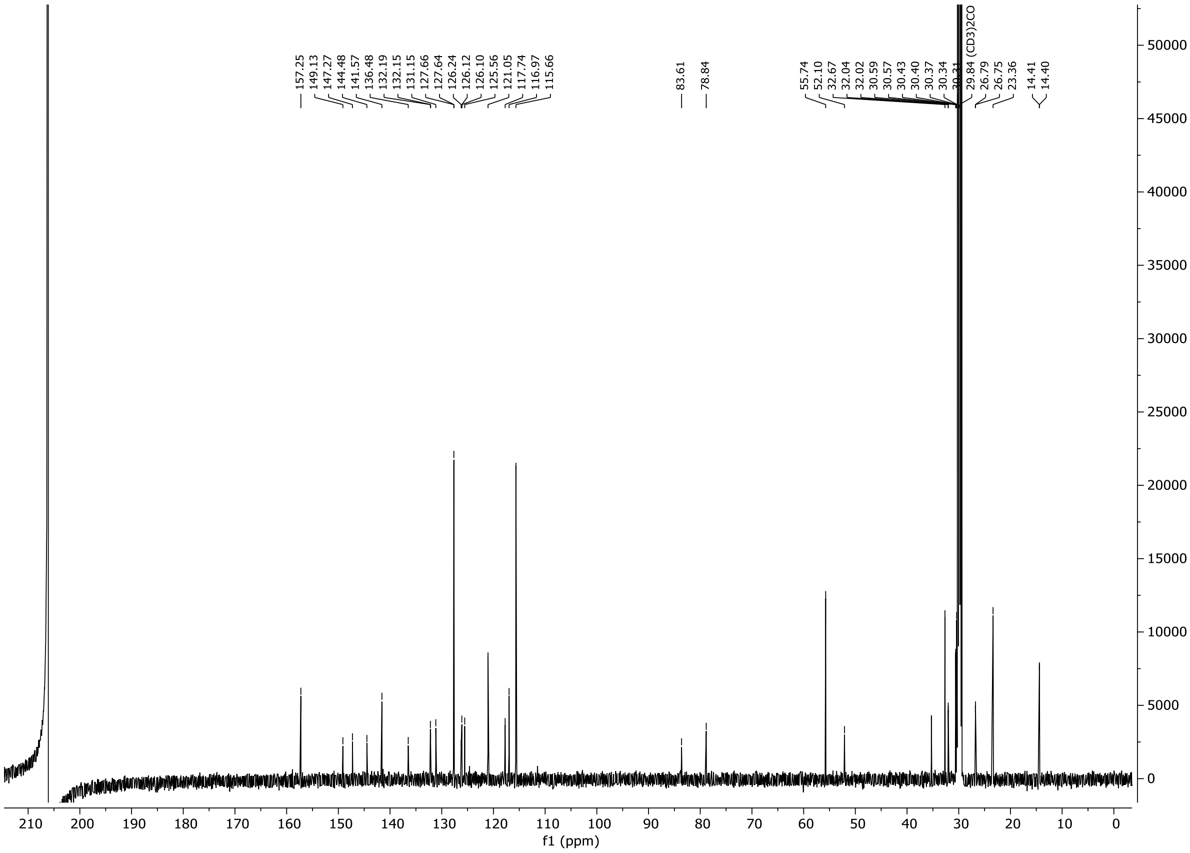


**Figure S180.** $^{13}$C NMR spectrum of compound **3g** (75 MHz, acetone-d$_6$, $T$ = 293 K).

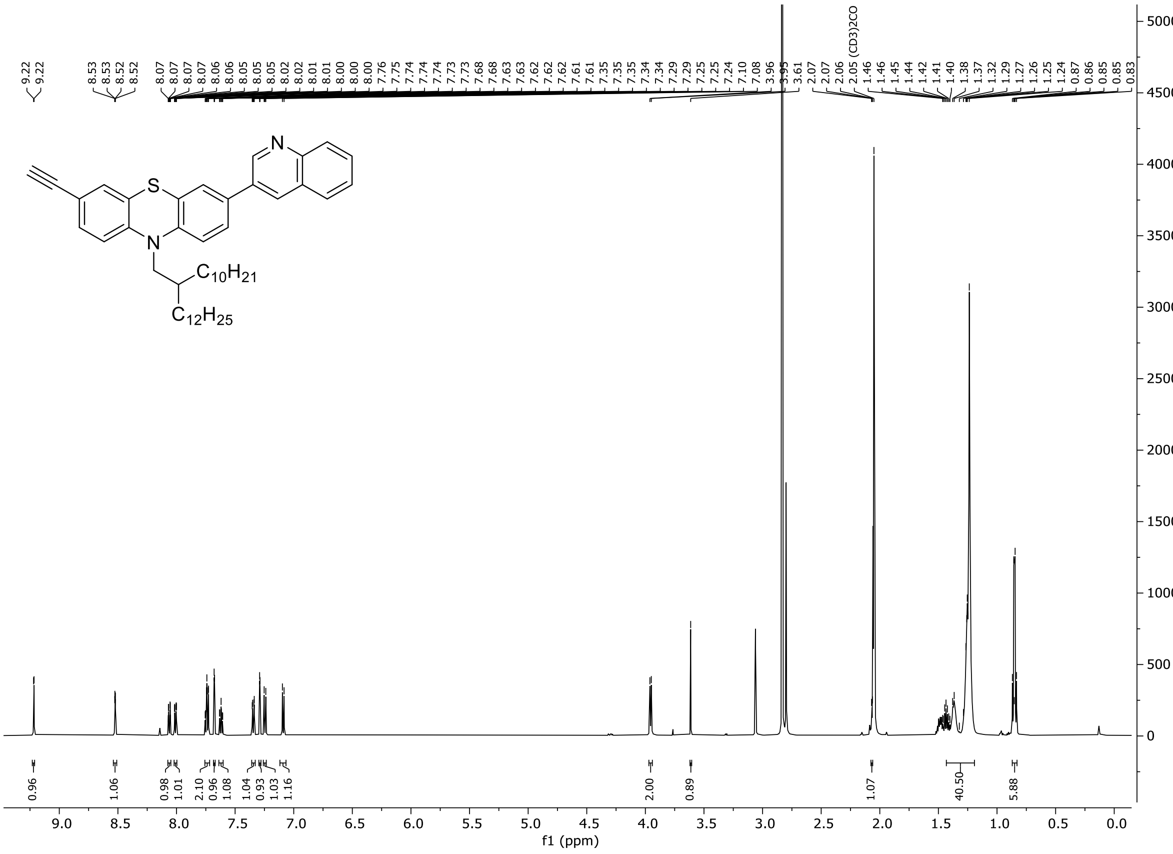


**Figure S181.** $^{1}$H NMR spectrum of compound **3h** (300 MHz, acetone-d$_6$, $T$ = 293 K).

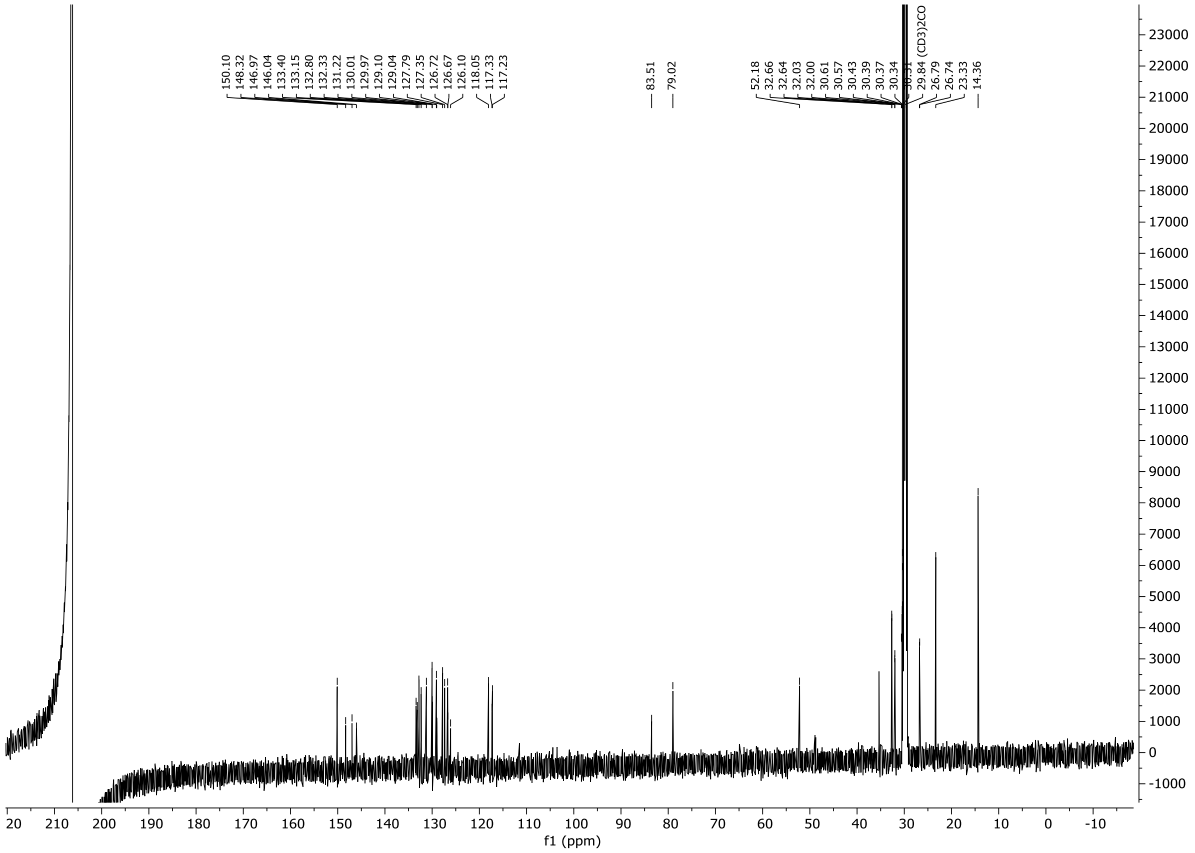

**Figure S182.** $^{13}C$ NMR spectrum of compound **3h** (75 MHz, acetone-d$_6$, $T$ = 293 K).

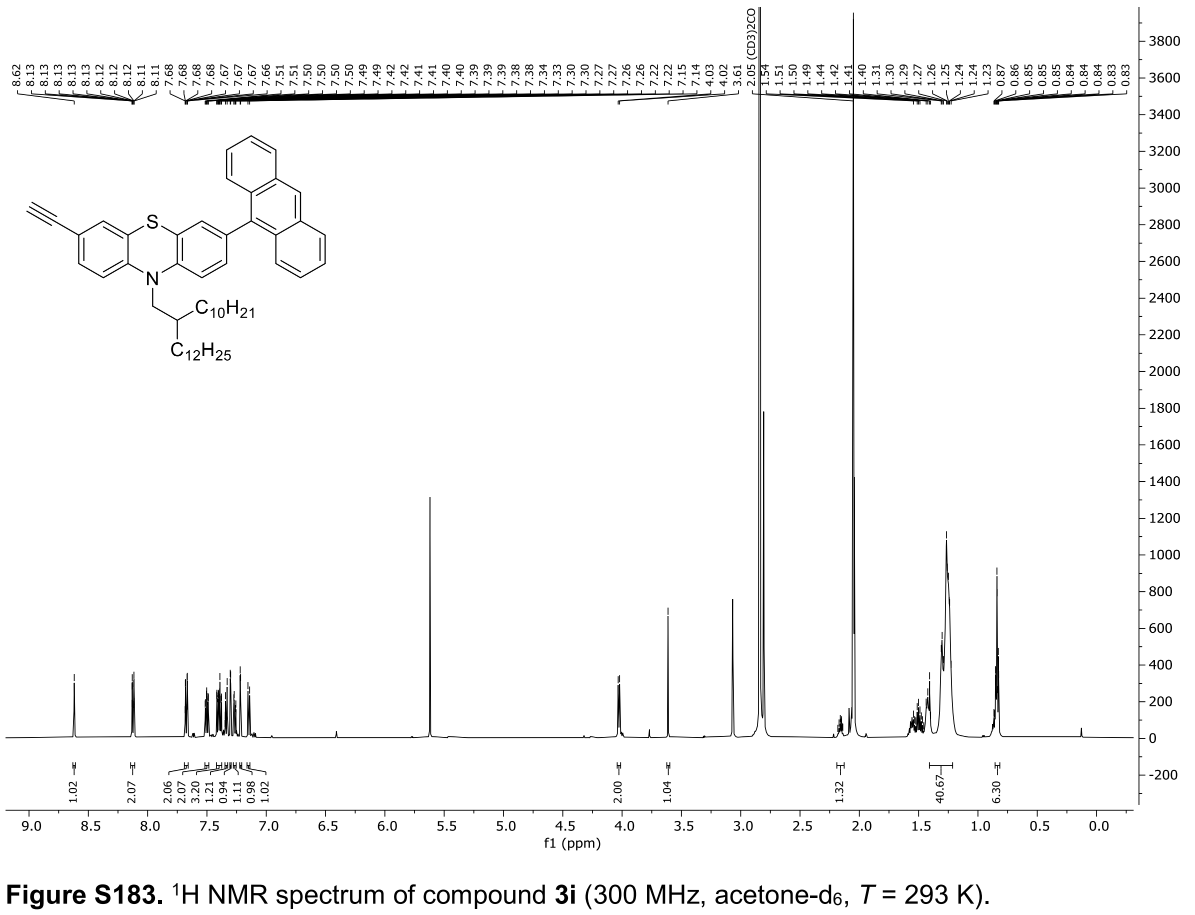

**Figure S183.** $^{1}H$ NMR spectrum of compound **3i** (300 MHz, acetone-d$_6$, $T$ = 293 K).

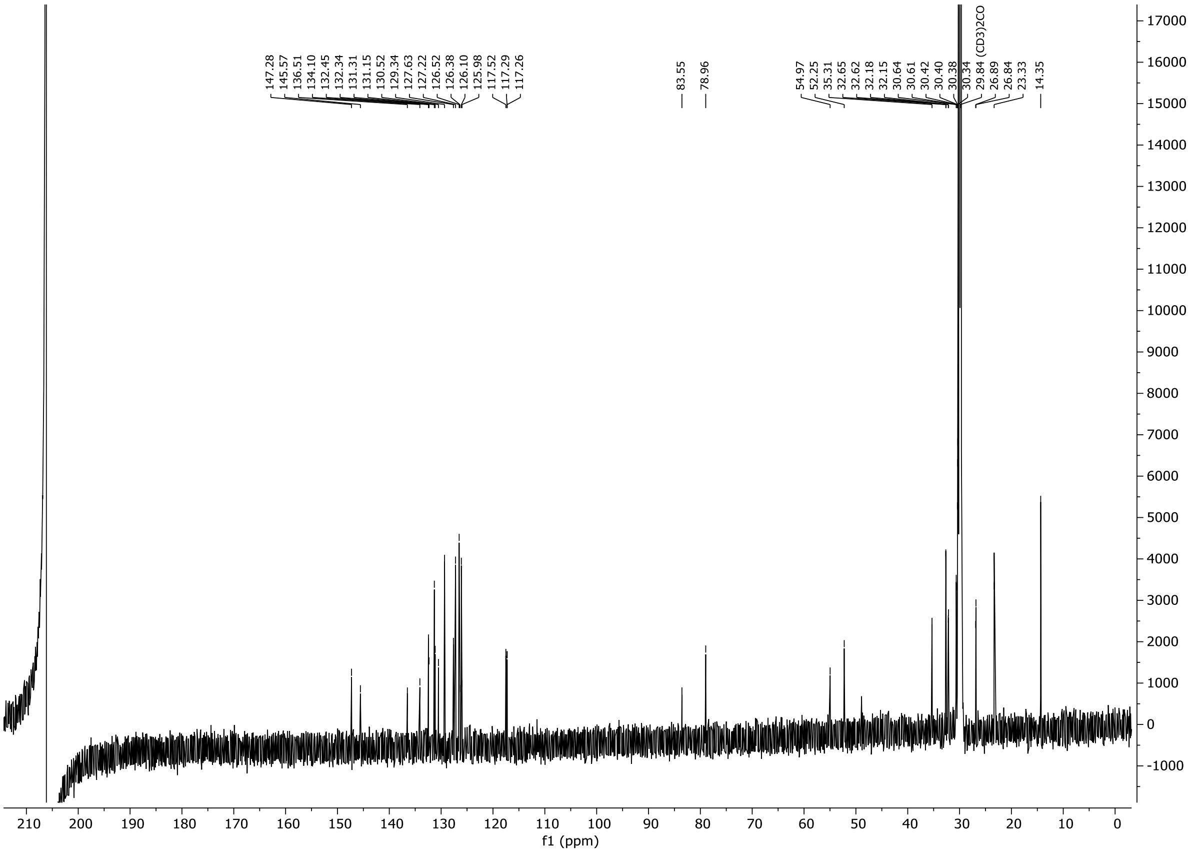

**Figure S184.** $^{13}C$ NMR spectrum of compound **3h** (75 MHz, acetone-d$_6$, $T$ = 293 K).

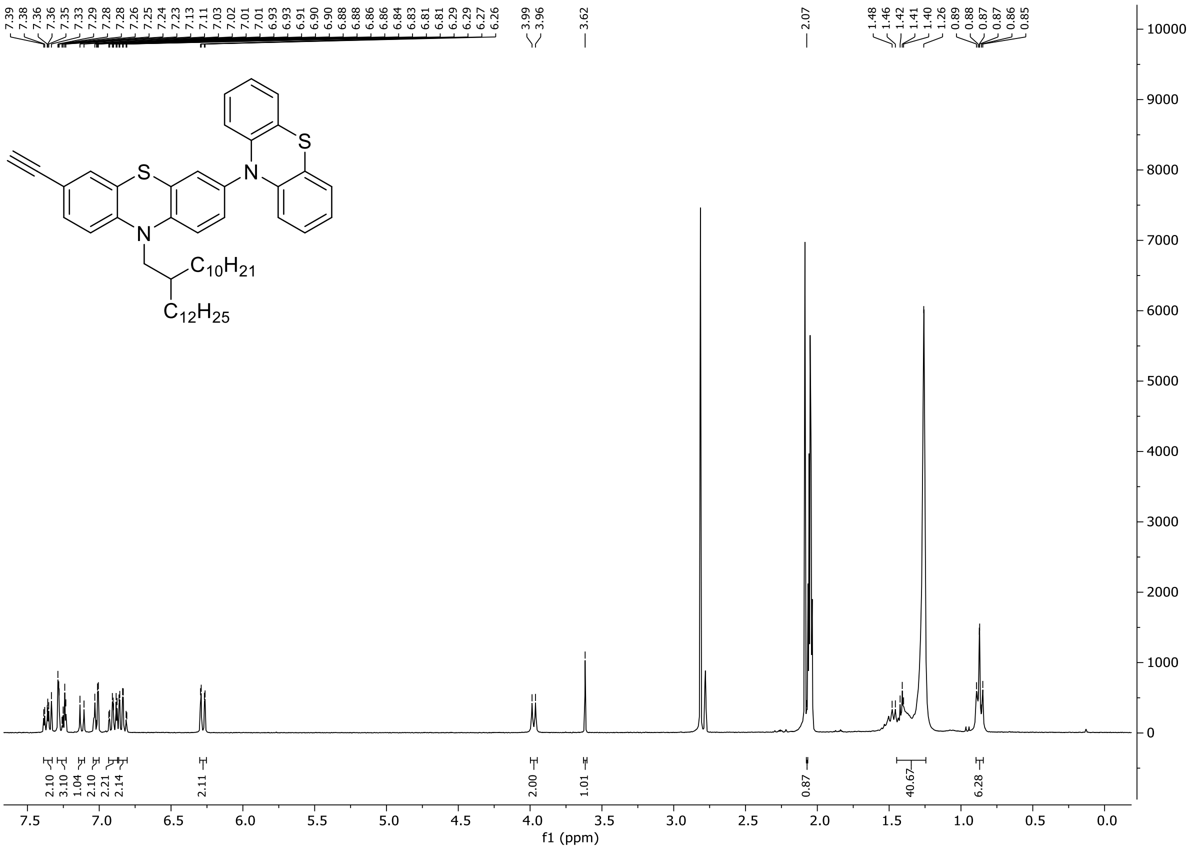

**Figure S185.** $^{1}H$ NMR spectrum of compound **3j** (300 MHz, acetone-d$_6$, $T$ = 293 K).

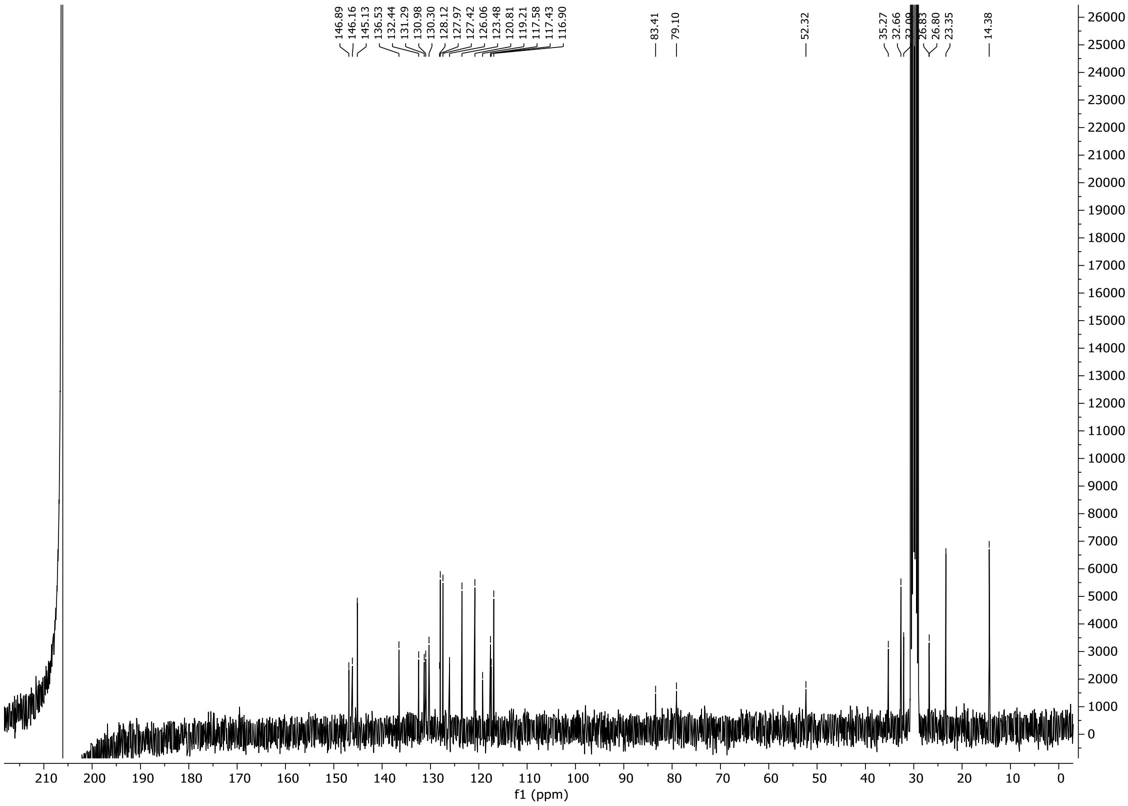


**Figure S186.** $^{13}$C NMR spectrum of compound **3j** (75 MHz, acetone-d$_6$, $T$ = 293 K).

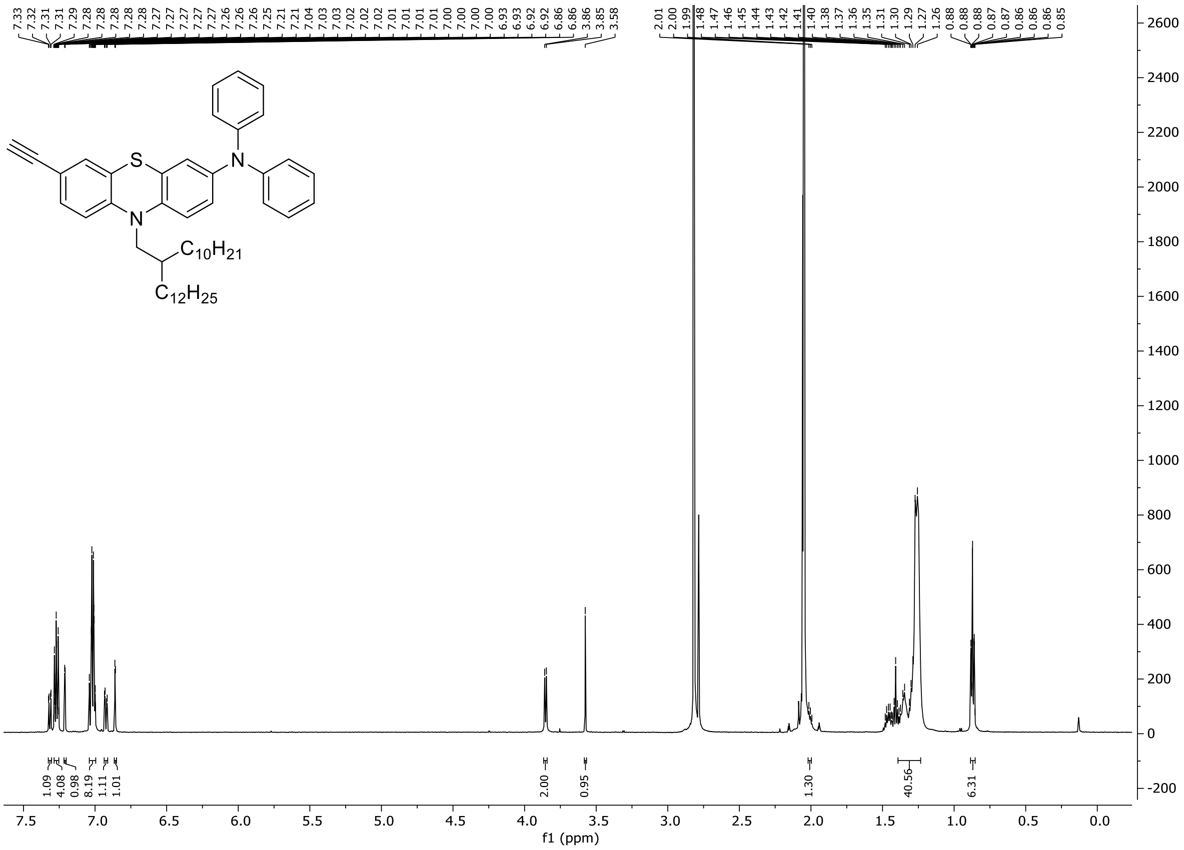

**Figure S187.** $^{1}$H NMR spectrum of compound **3k** (300 MHz, acetone-d$_6$, $T$ = 293 K).

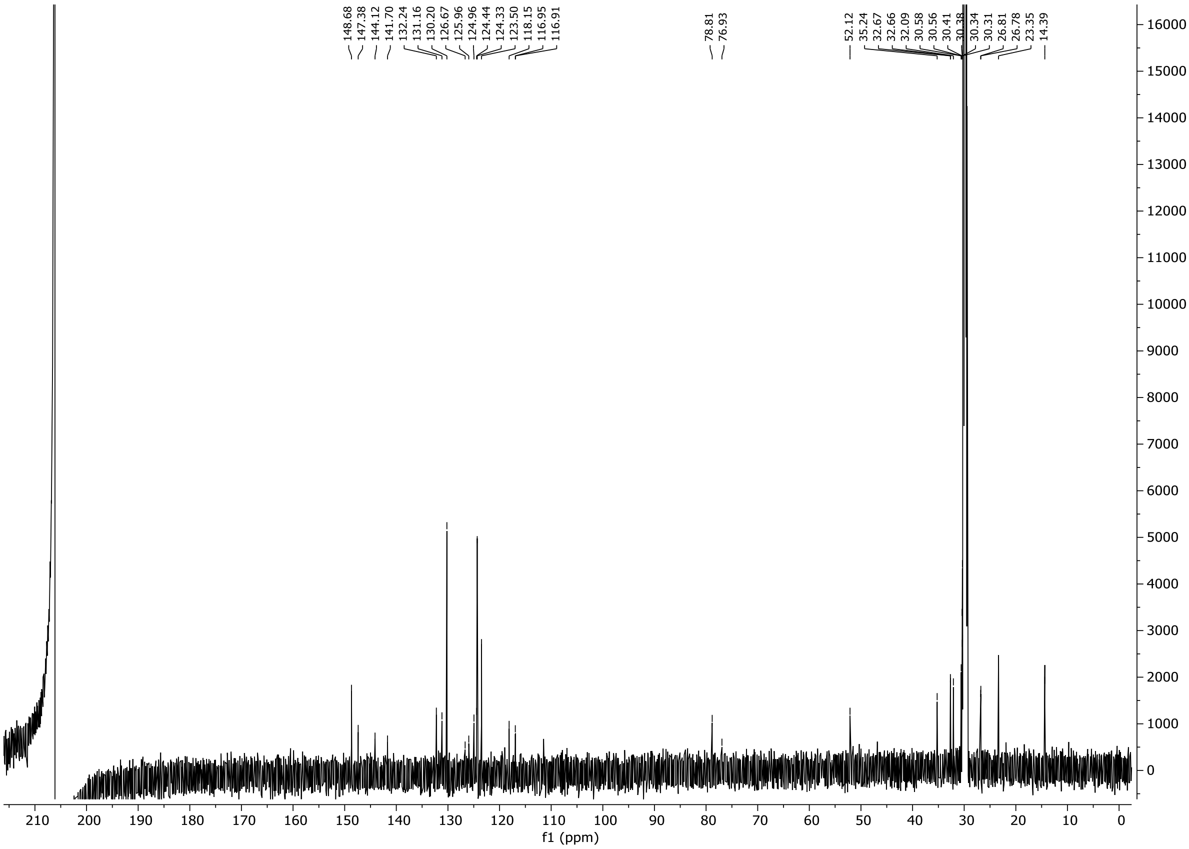


**Figure S188.** $^{13}$C NMR spectrum of compound **3k** (75 MHz, acetone-d$_6$, $T$ = 293 K).

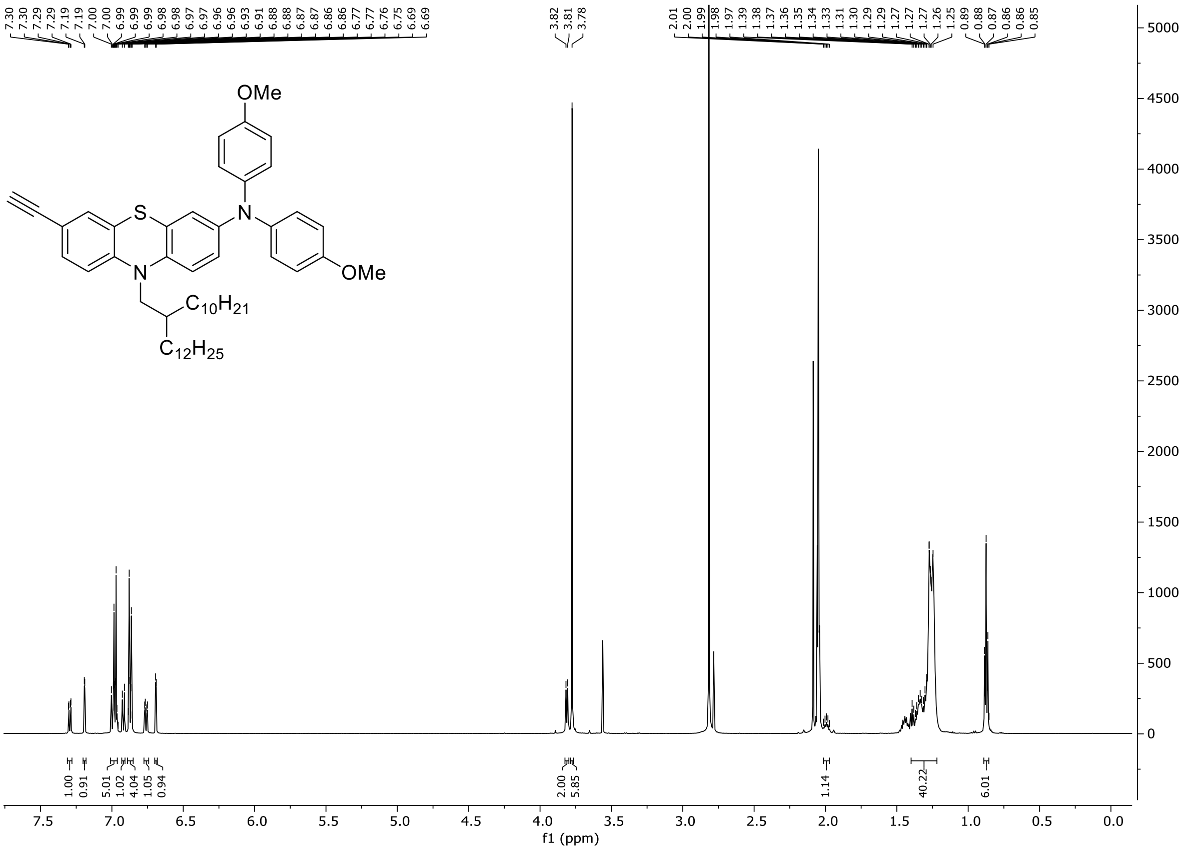


**Figure S189.** $^{1}$H NMR spectrum of compound **3l** (300 MHz, acetone-d$_6$, $T$ = 293 K).

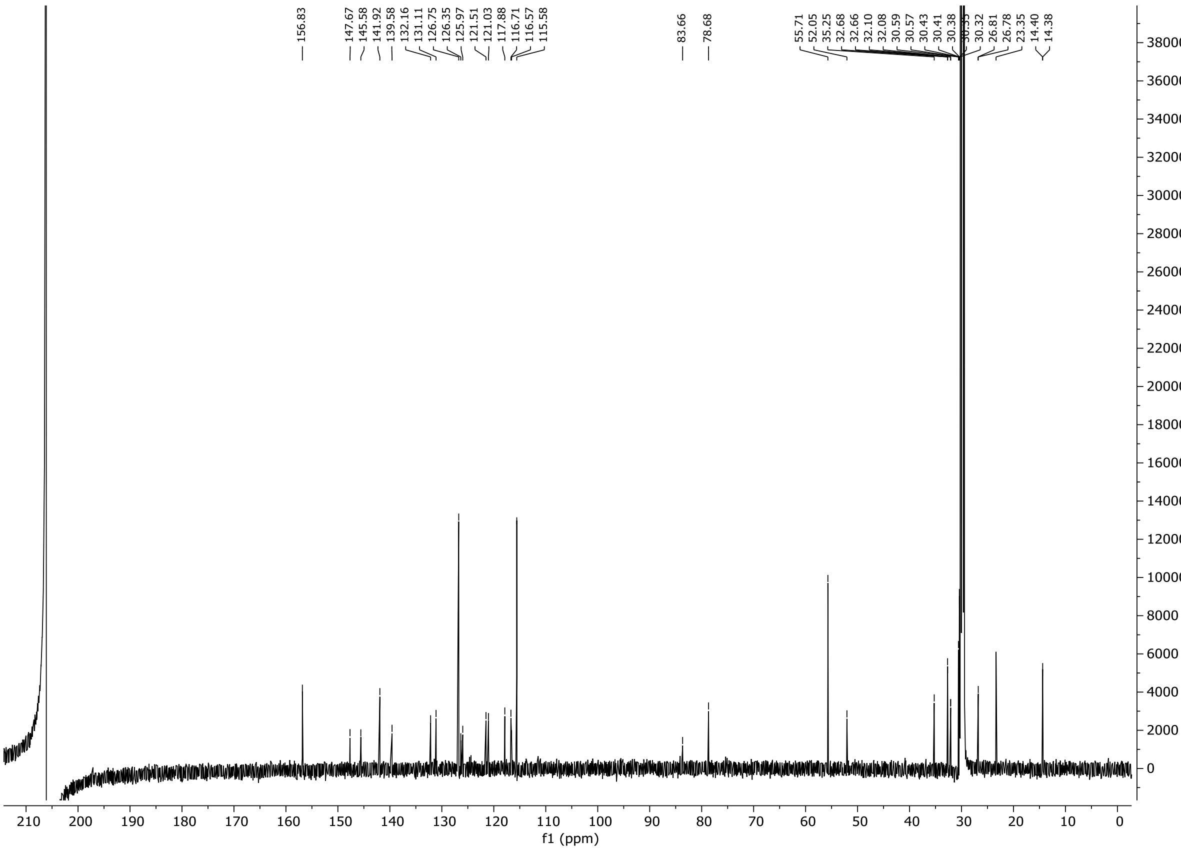


**Figure S190.** $^{13}C$ NMR spectrum of compound **3l** (75 MHz, acetone-d$_6$, $T$ = 293 K).

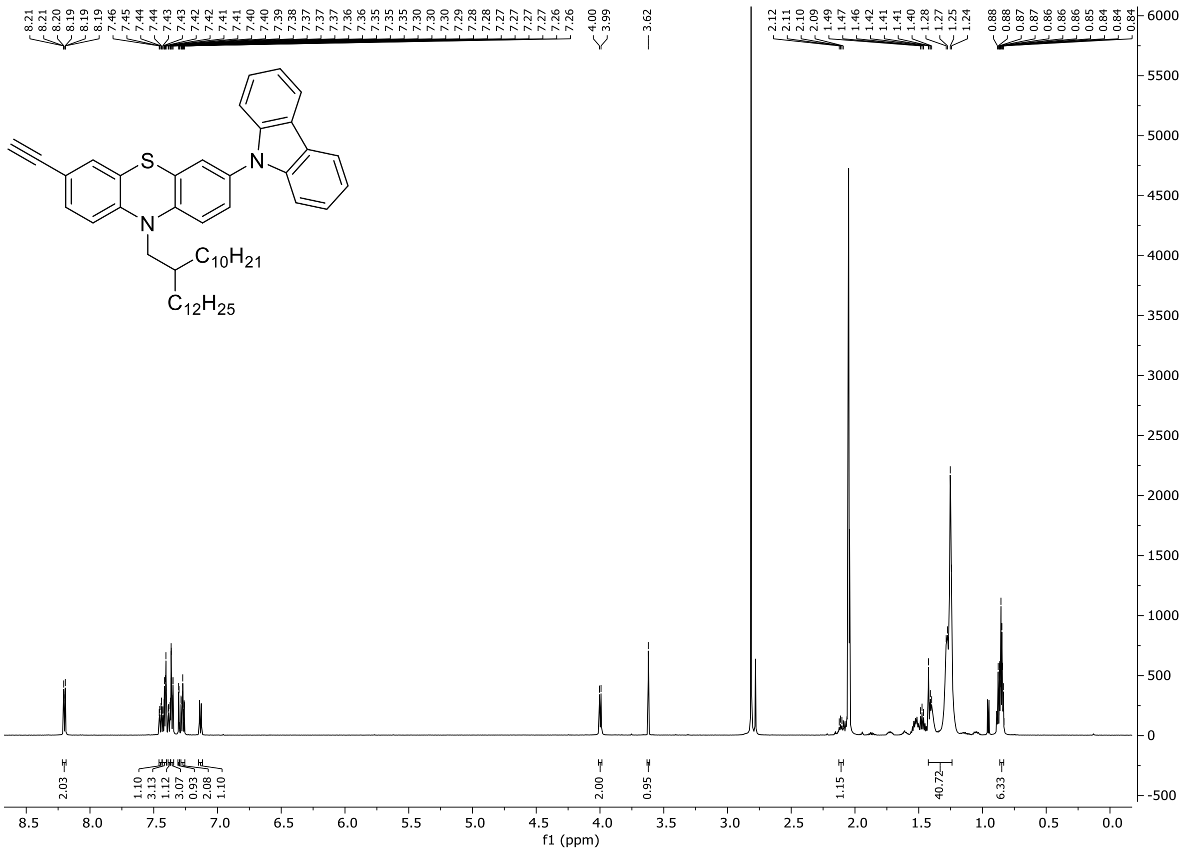


**Figure S191.** $^1H$ NMR spectrum of compound **3m** (300 MHz, acetone-d$_6$, $T$ = 293 K).

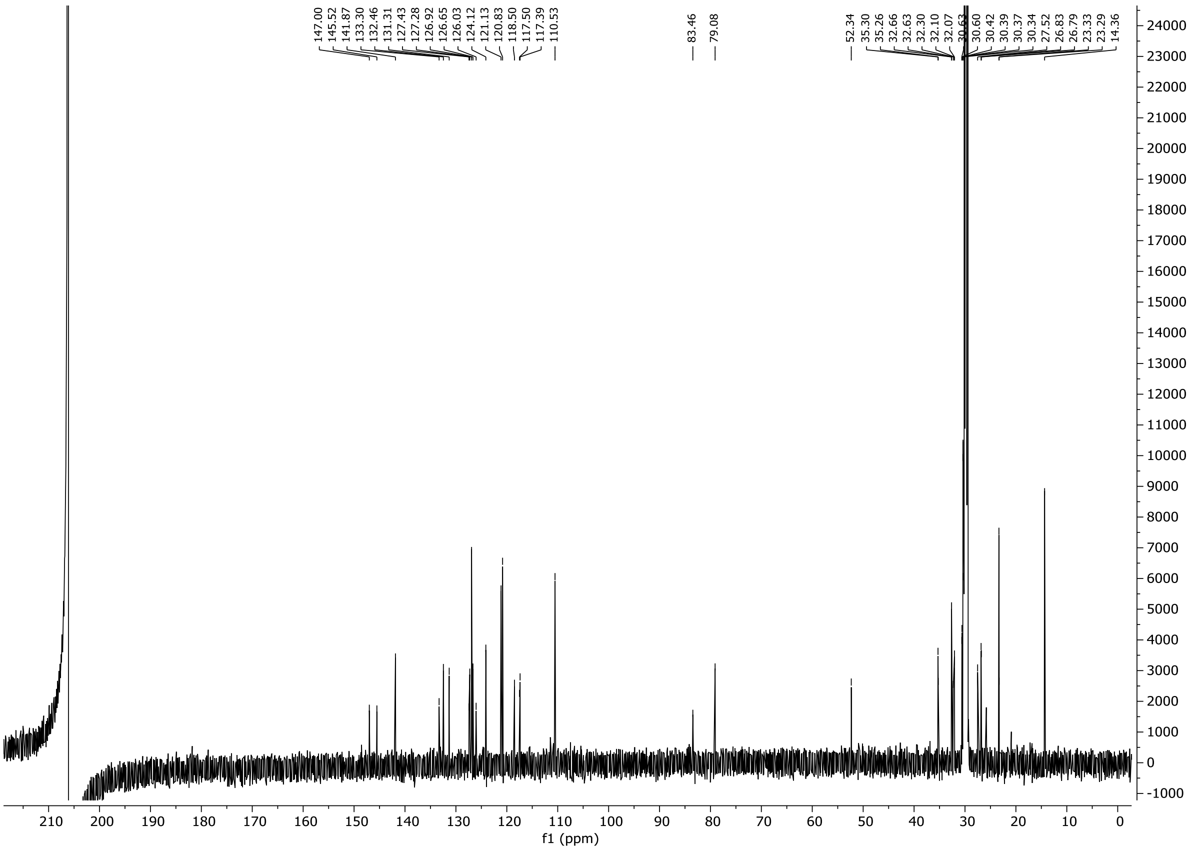


**Figure S192.** $^{13}C$ NMR spectrum of compound **3m** (75 MHz, acetone-d$_6$, $T$ = 293 K).

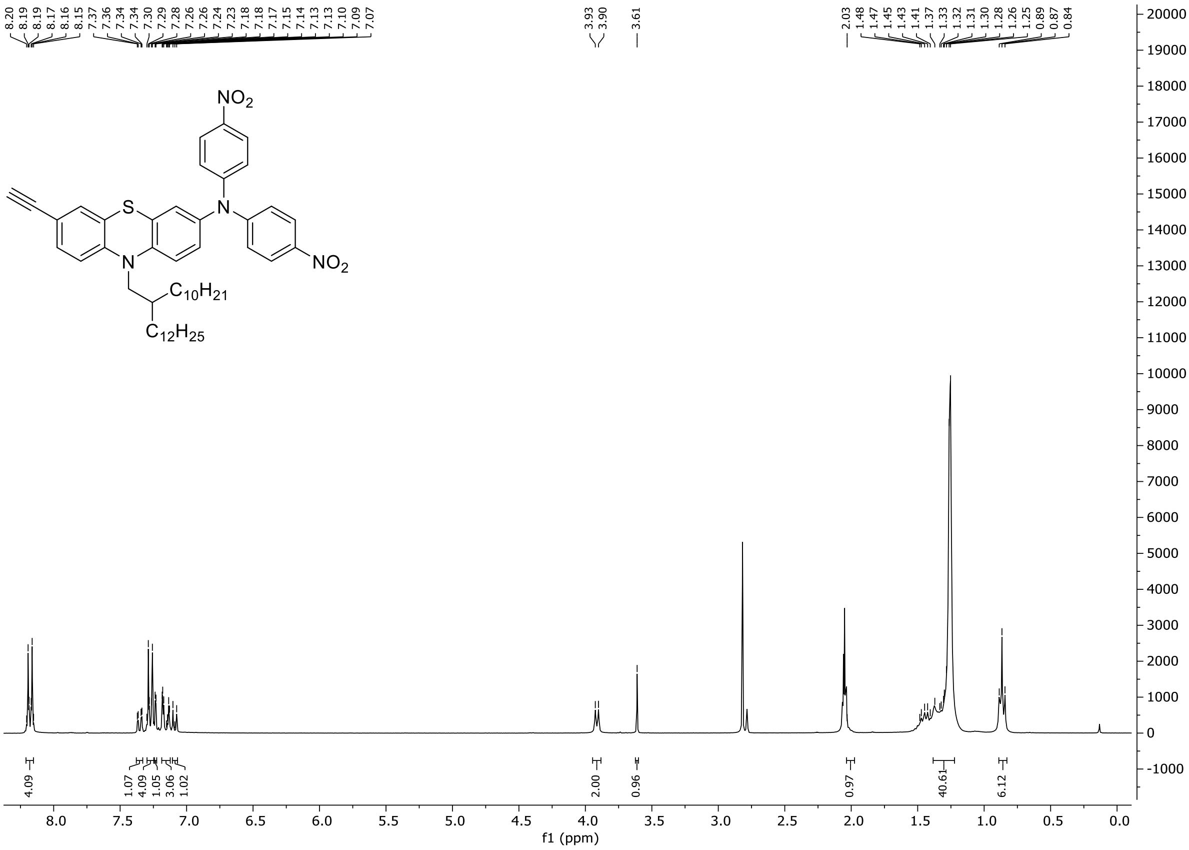


**Figure S193.** $^{1}H$ NMR spectrum of compound **3n** (300 MHz, acetone-d$_6$, $T$ = 293 K).

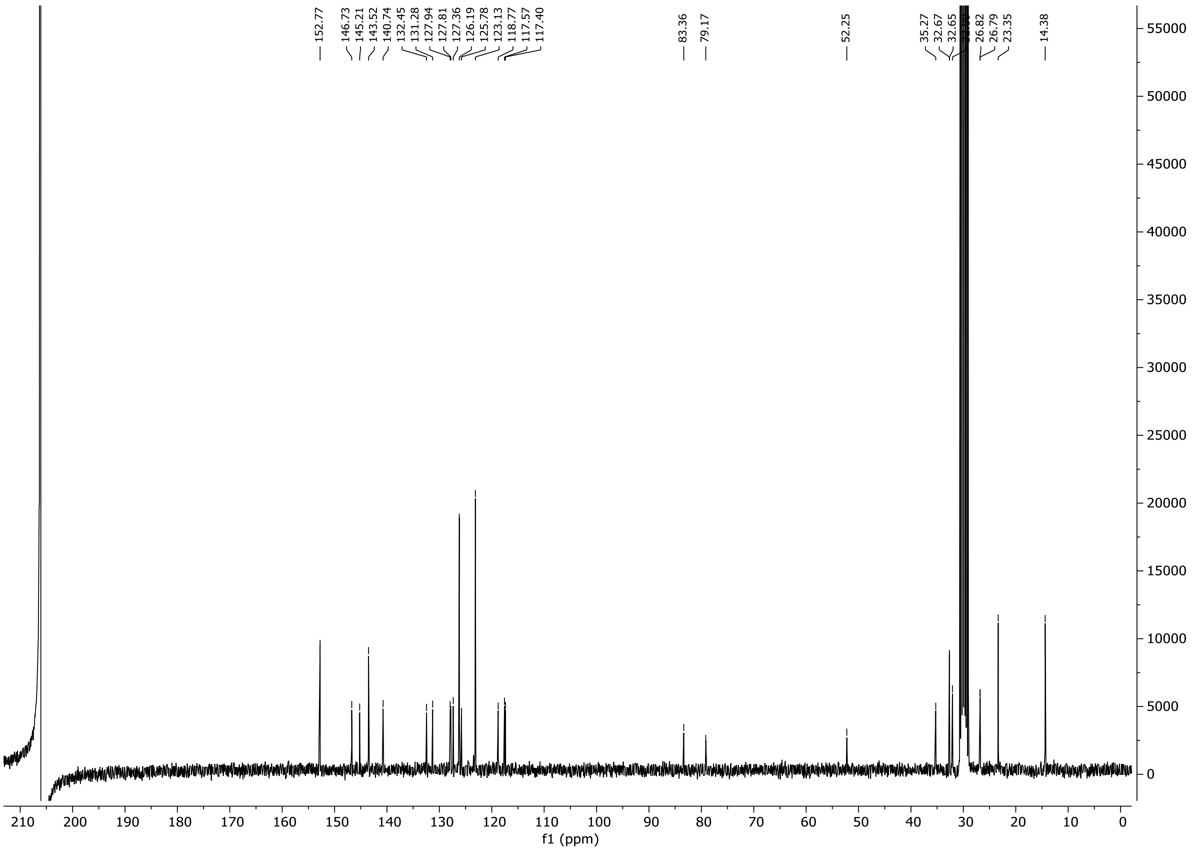


**Figure S194.** $^{13}C$ NMR spectrum of compound **3n** (75 MHz, acetone-d$_6$, *T* = 293 K).

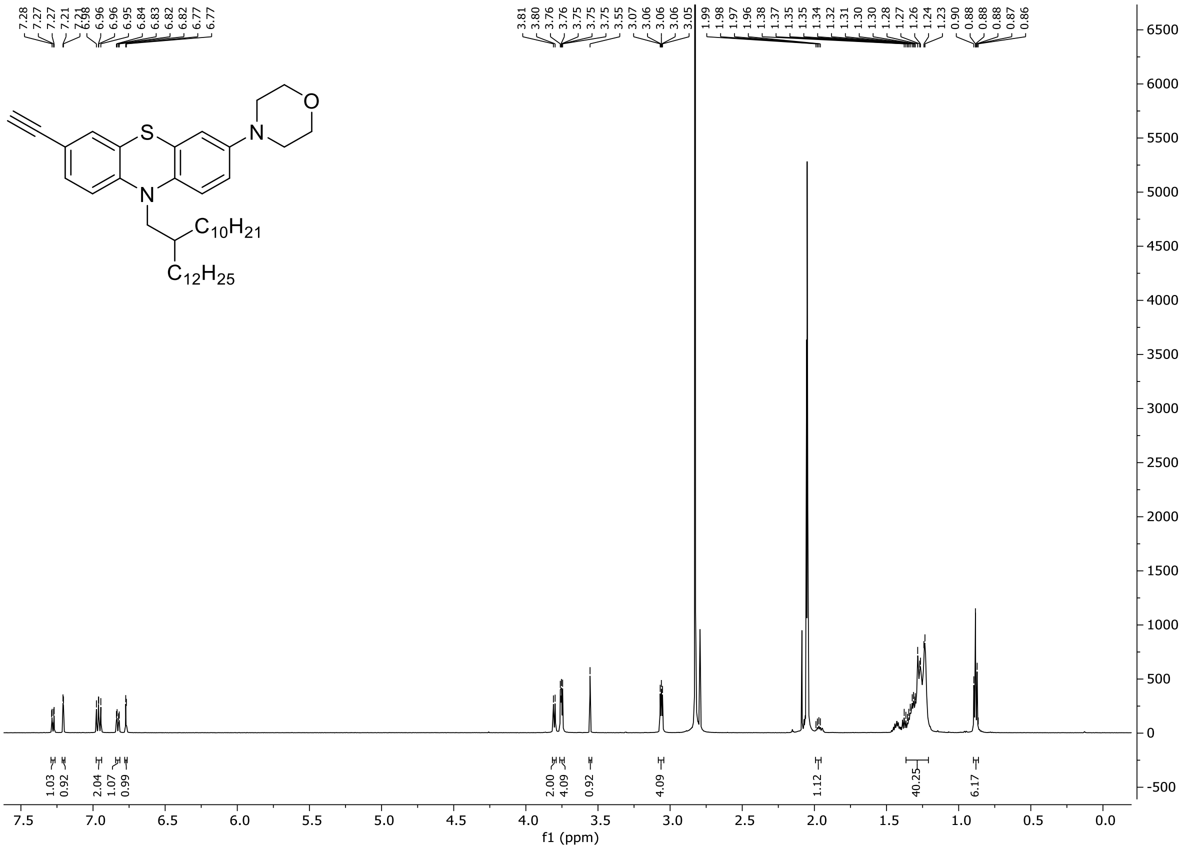


**Figure S195.** $^{1}H$ NMR spectrum of compound **3o** (300 MHz, acetone-d$_6$, *T* = 293 K).

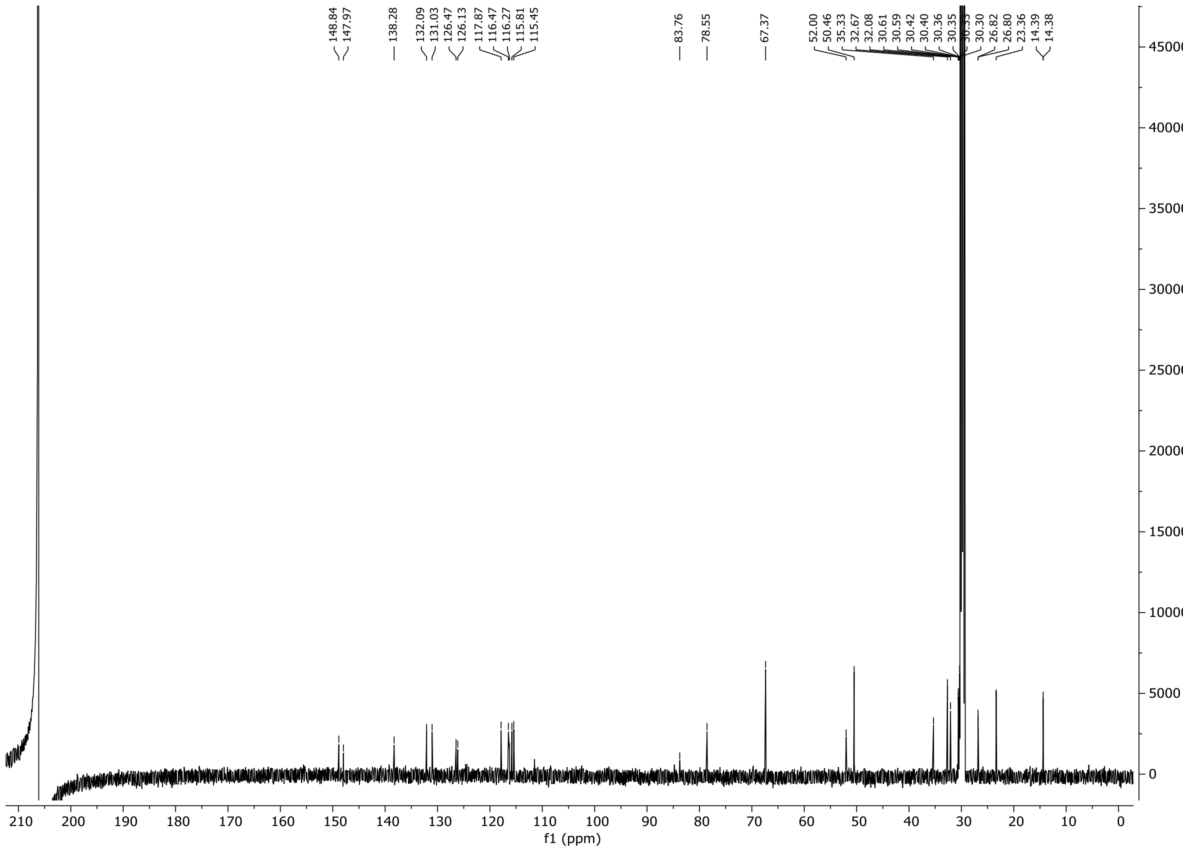


**Figure S196.** $^{13}$C NMR spectrum of compound **3o** (75 MHz, acetone-d$_6$, $T$ = 293 K).

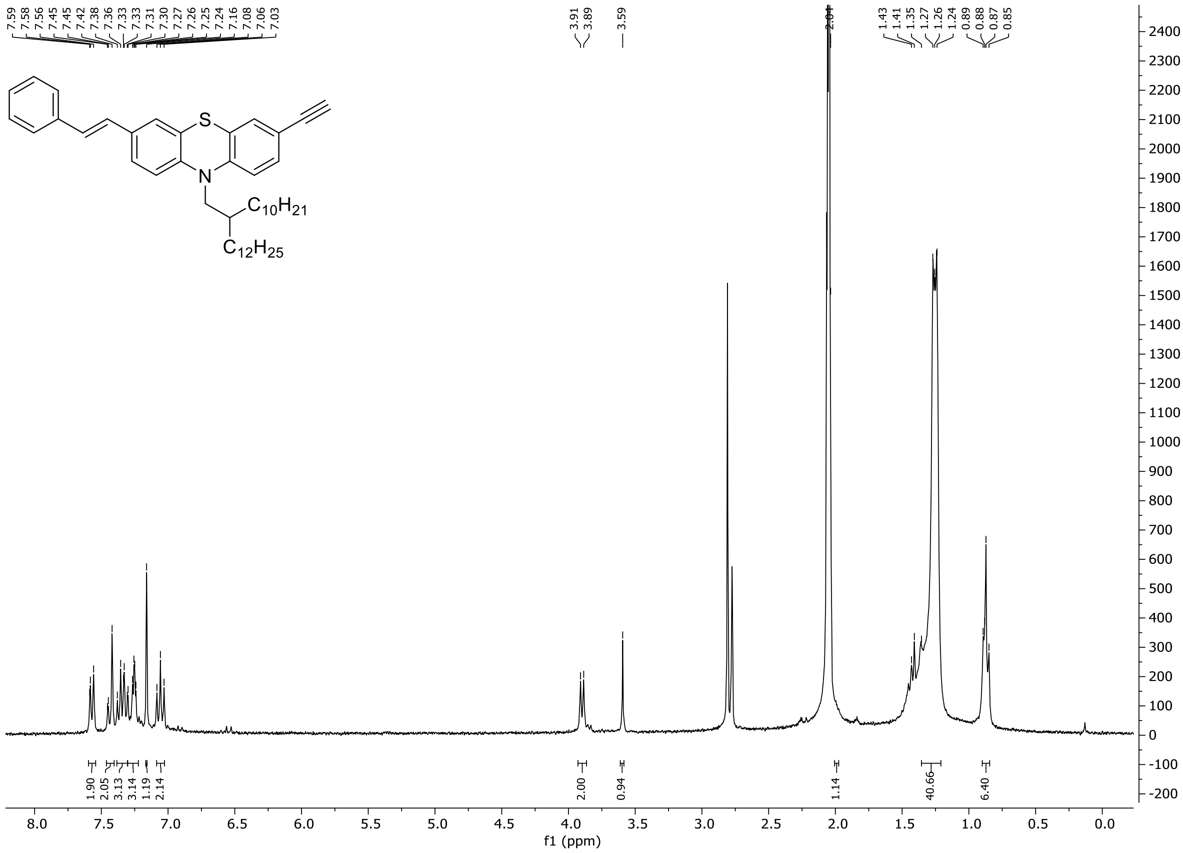


**Figure S197.** $^{1}$H NMR spectrum of compound **3p** (300 MHz, acetone-d$_6$, $T$ = 293 K).

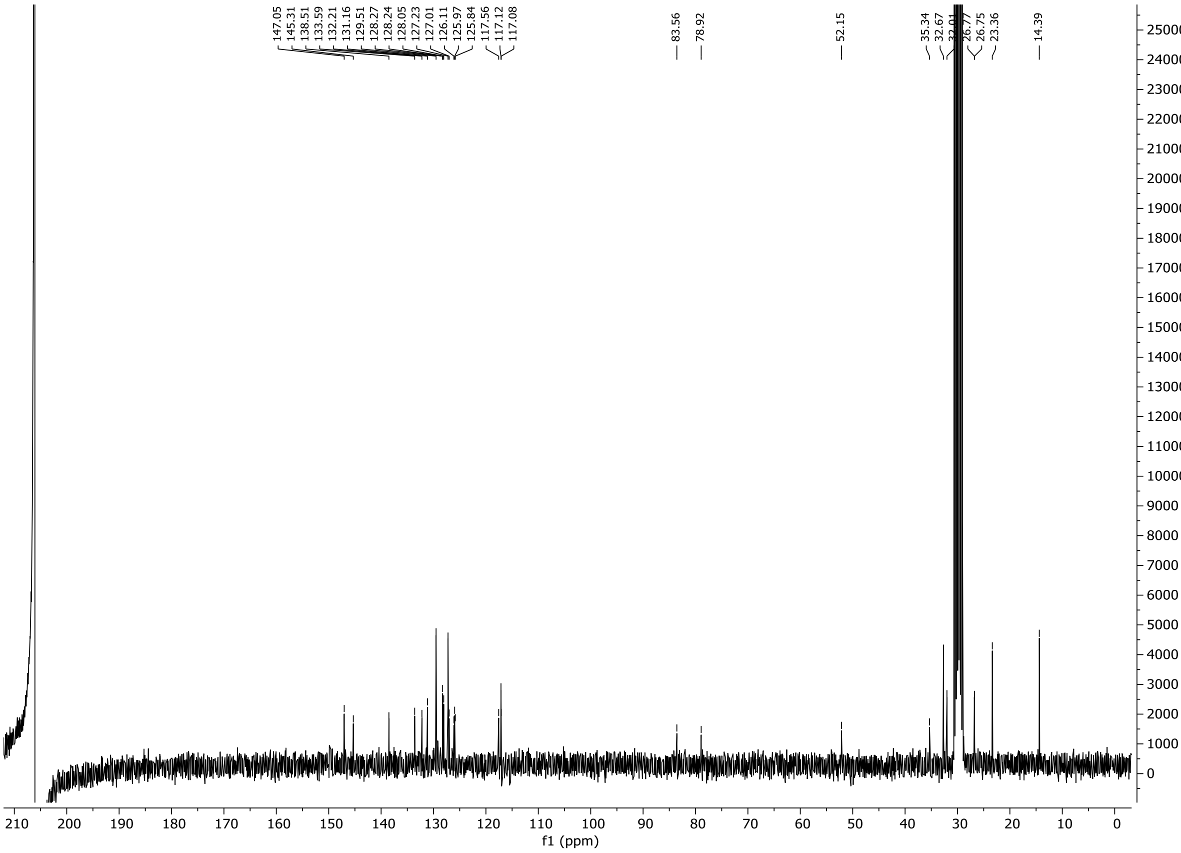

**Figure S198.** $^{13}$C NMR spectrum of compound **3p** (75 MHz, acetone-d$_6$, *T* = 293 K).

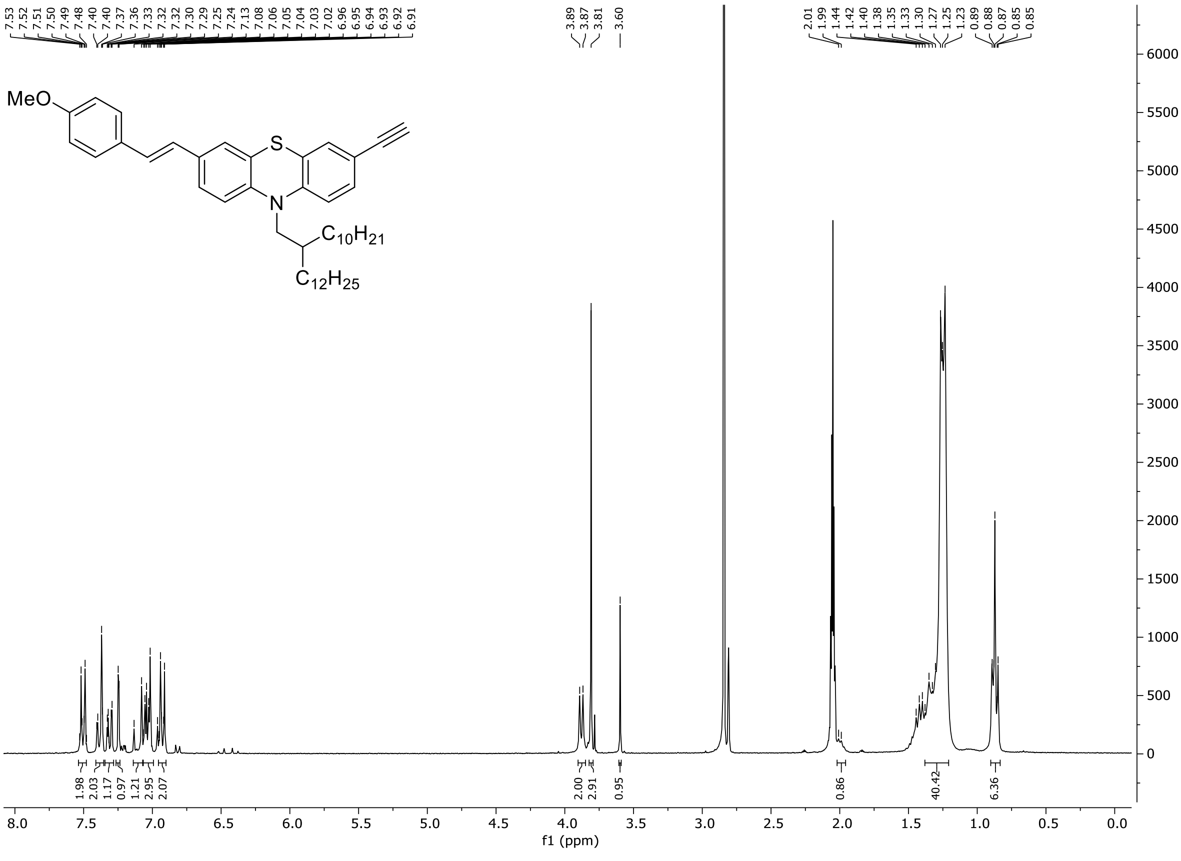


**Figure S199.** $^{1}$H NMR spectrum of compound **3q** (300 MHz, acetone-d$_6$, *T* = 293 K).

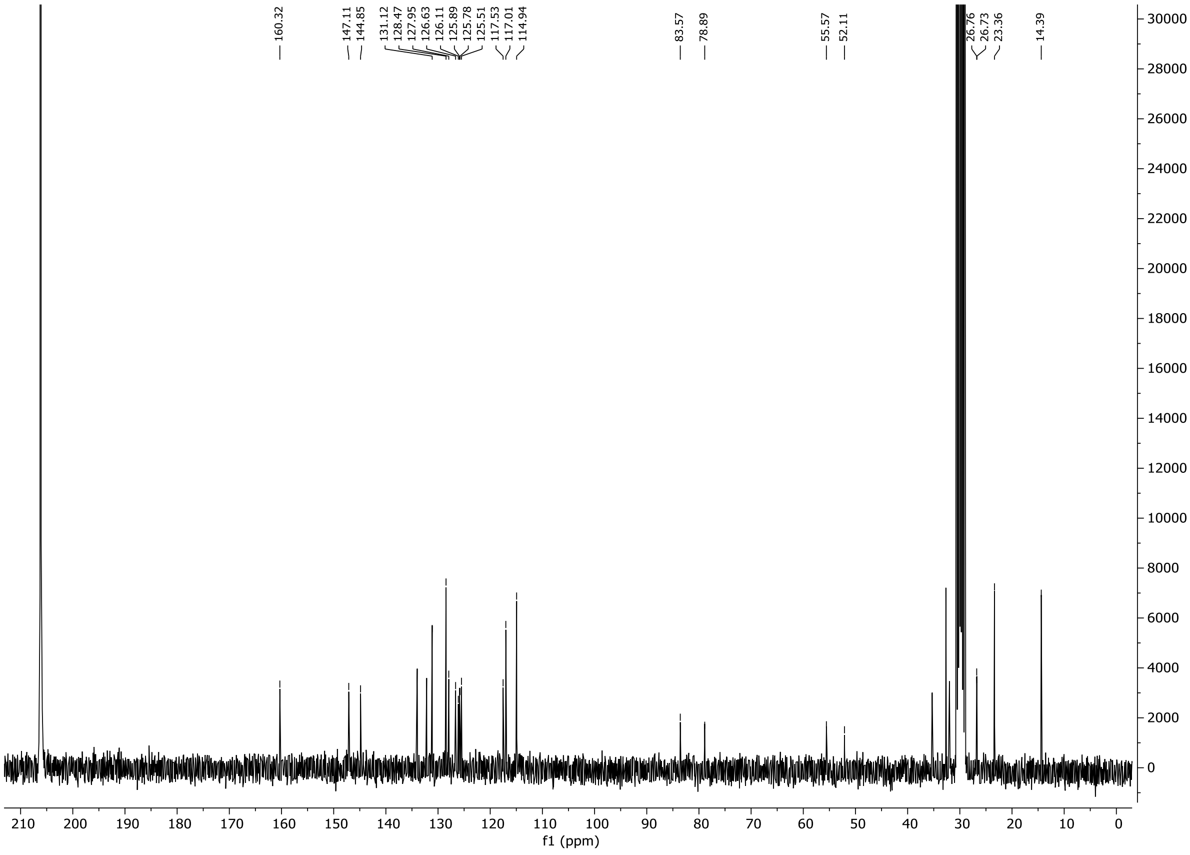


**Figure S200.** $^{13}C$ NMR spectrum of compound **3q** (75 MHz, acetone-$d_6$, *T* = 293 K).

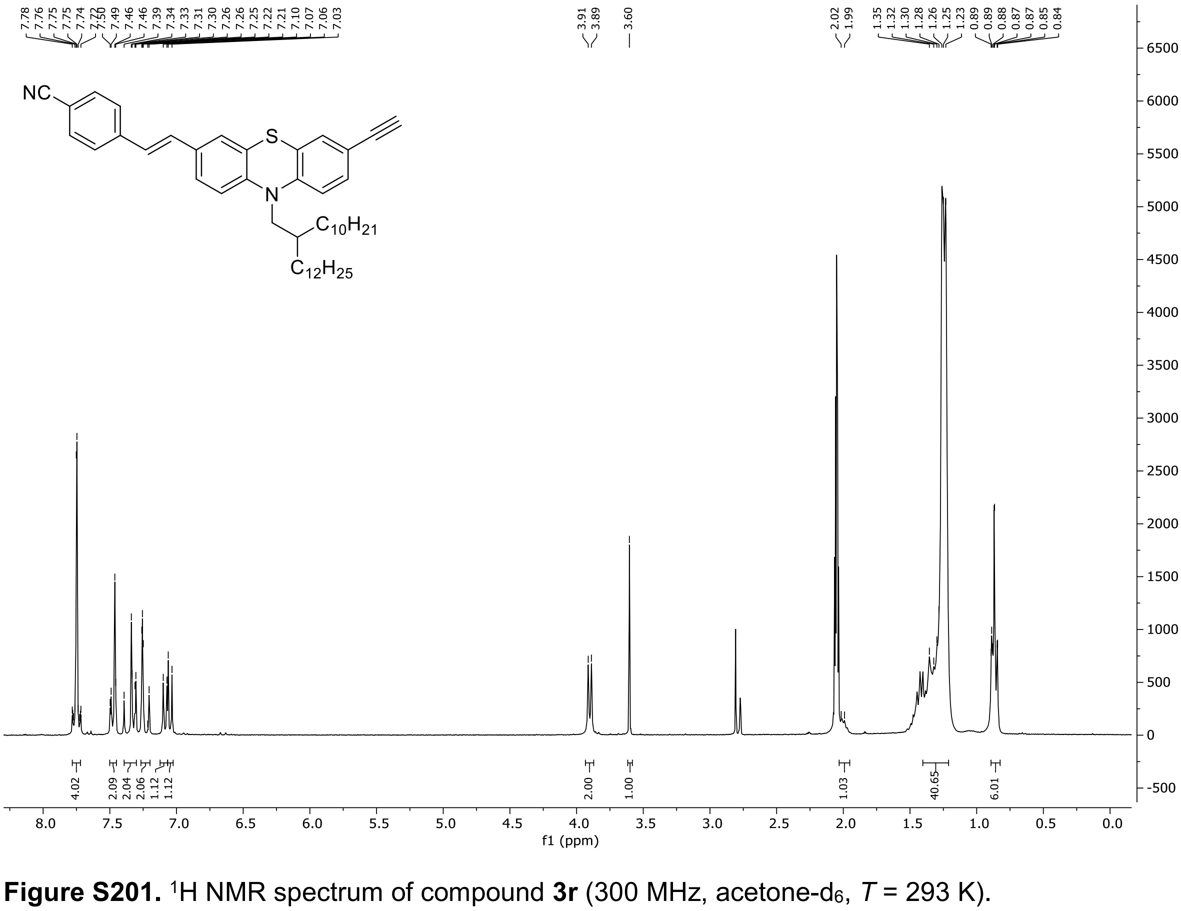


**Figure S201.** $^{1}H$ NMR spectrum of compound **3r** (300 MHz, acetone-$d_6$, *T* = 293 K).

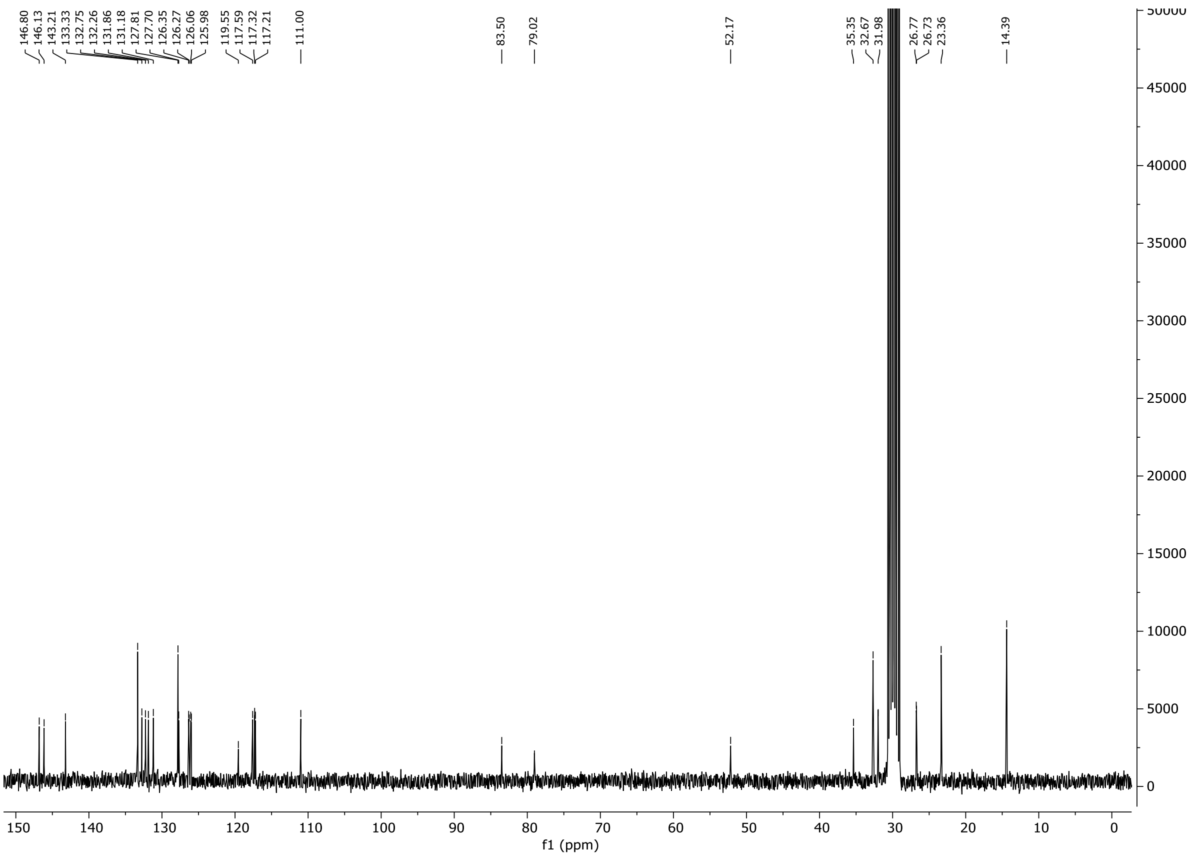

**Figure S202.** $^{13}C$ NMR spectrum of compound **3r** (75 MHz, acetone-$d_6$, *T* = 293 K).

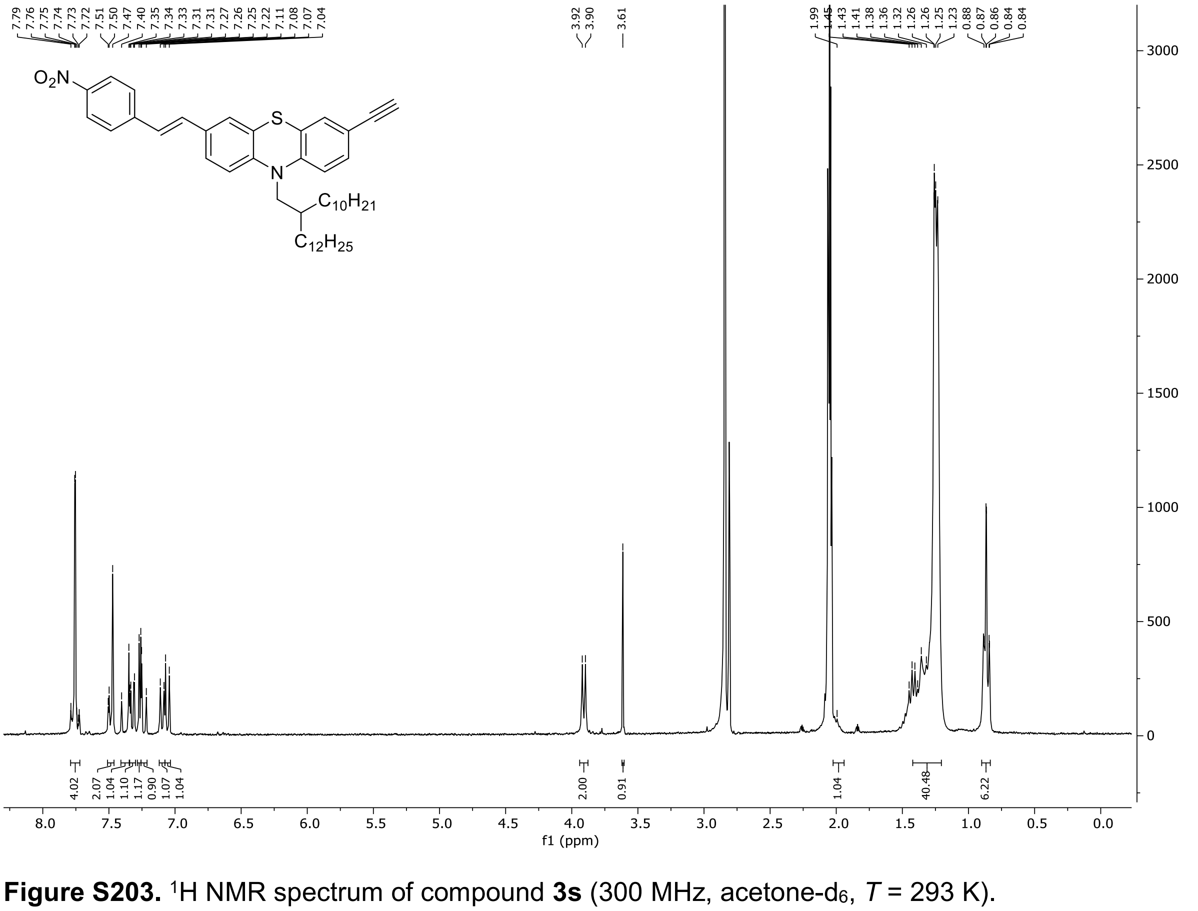

**Figure S203.** $^{1}H$ NMR spectrum of compound **3s** (300 MHz, acetone-$d_6$, *T* = 293 K).

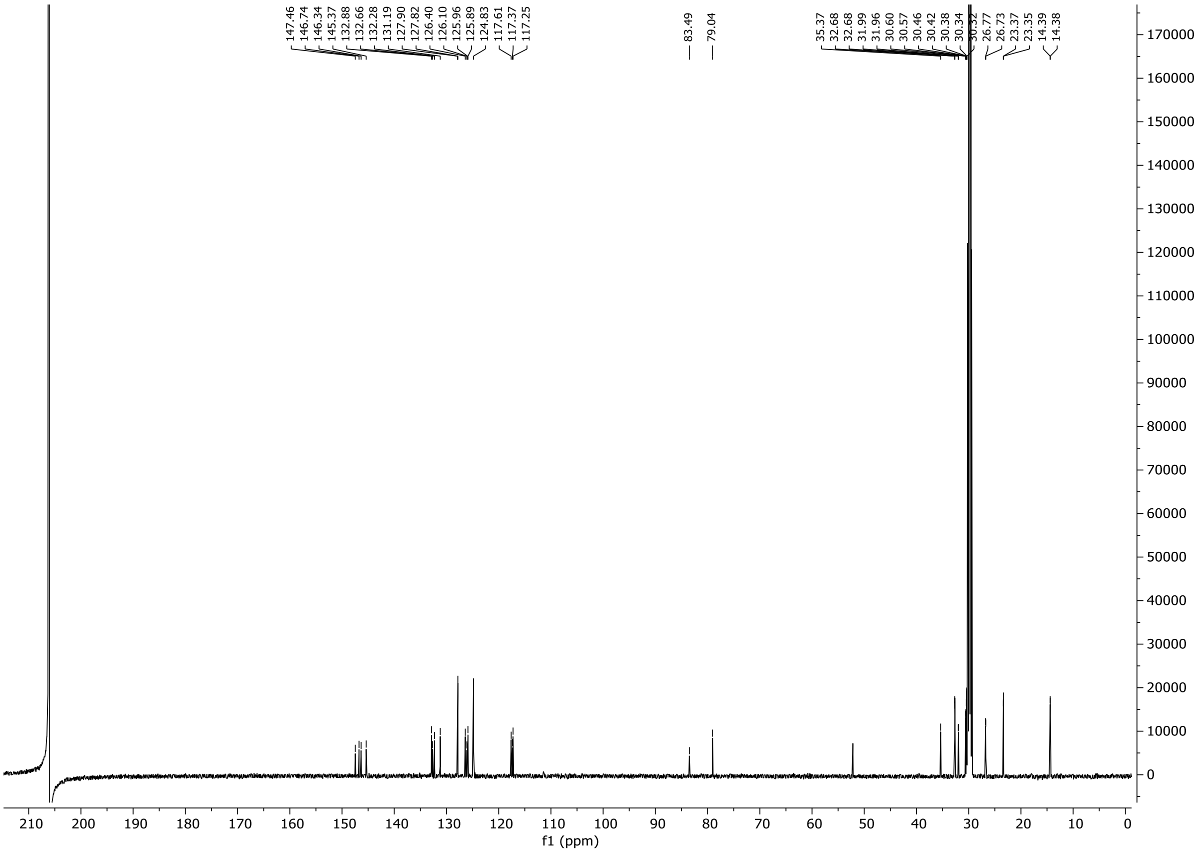


**Figure S204.** $^{13}C$ NMR spectrum of compound **3s** (75 MHz, acetone-d$_6$, $T$ = 293 K).

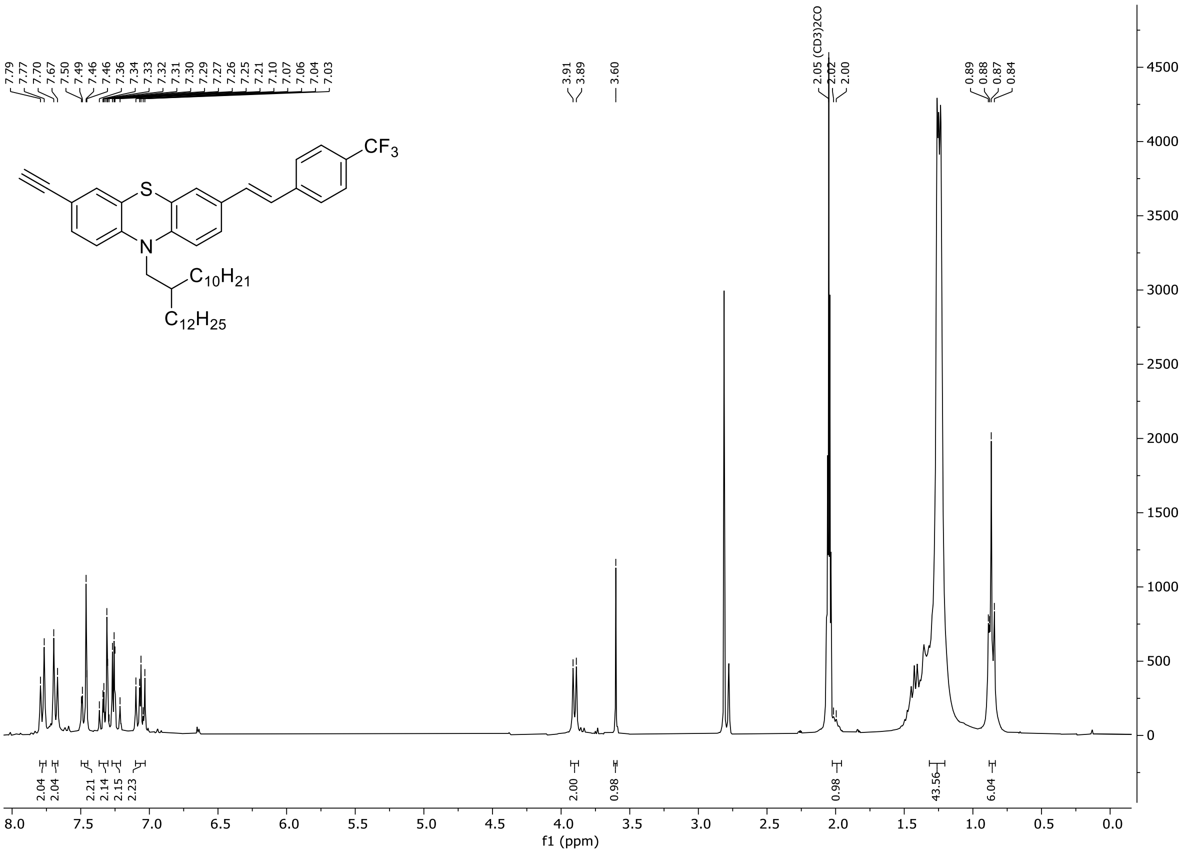


**Figure S205.** $^{1}H$ NMR spectrum of compound **3t** (300 MHz, acetone-d$_6$, $T$ = 293 K).

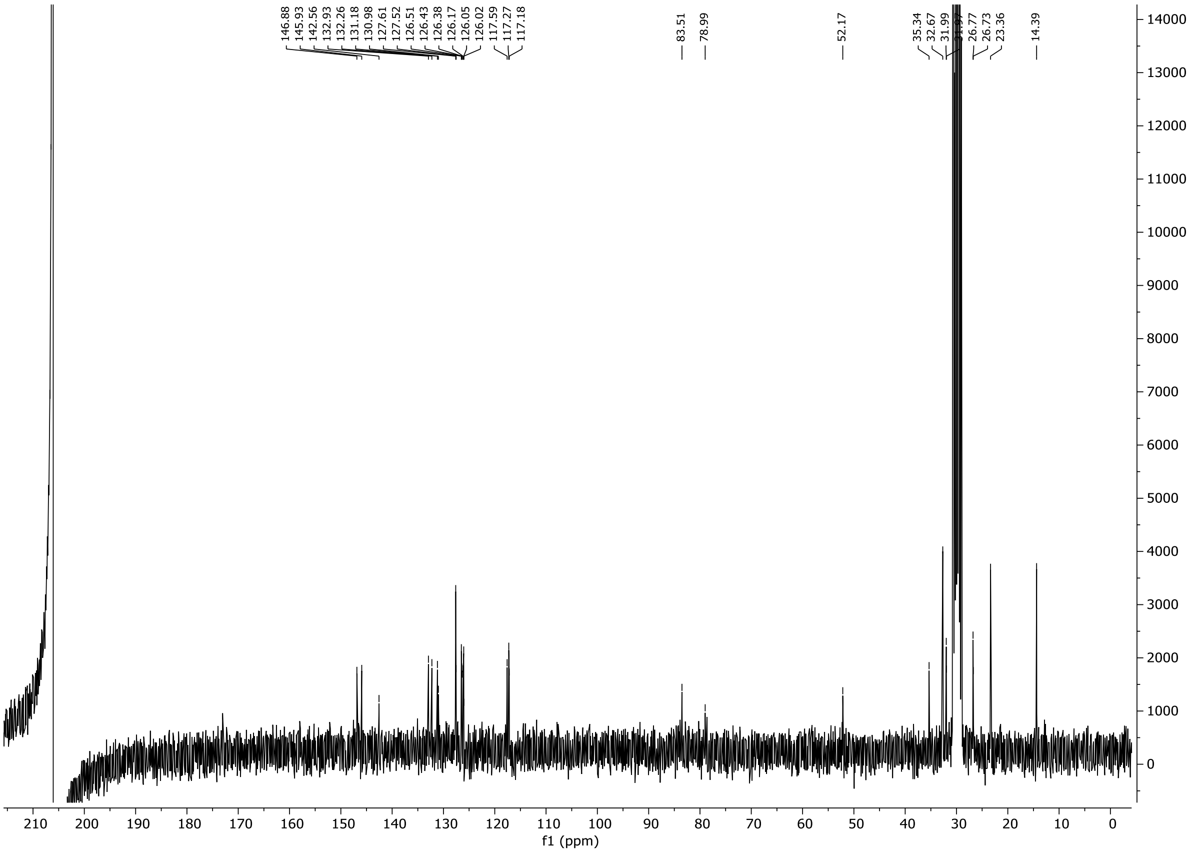

**Figure S206.** $^{13}$C NMR spectrum of compound **3t** (75 MHz, acetone-$d_6$, $T$ = 293 K).

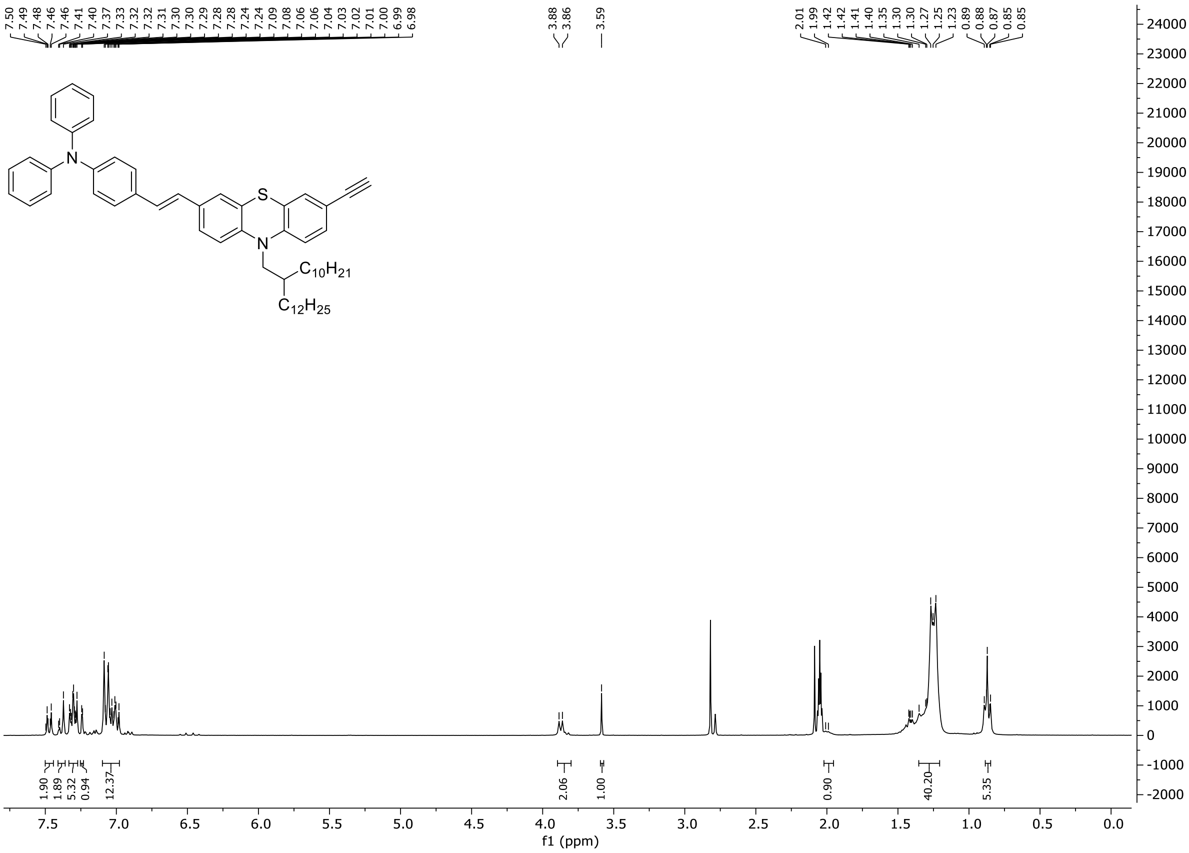

**Figure S207.** $^{1}$H NMR spectrum of compound **3u** (300 MHz, acetone-$d_6$, $T$ = 293 K).

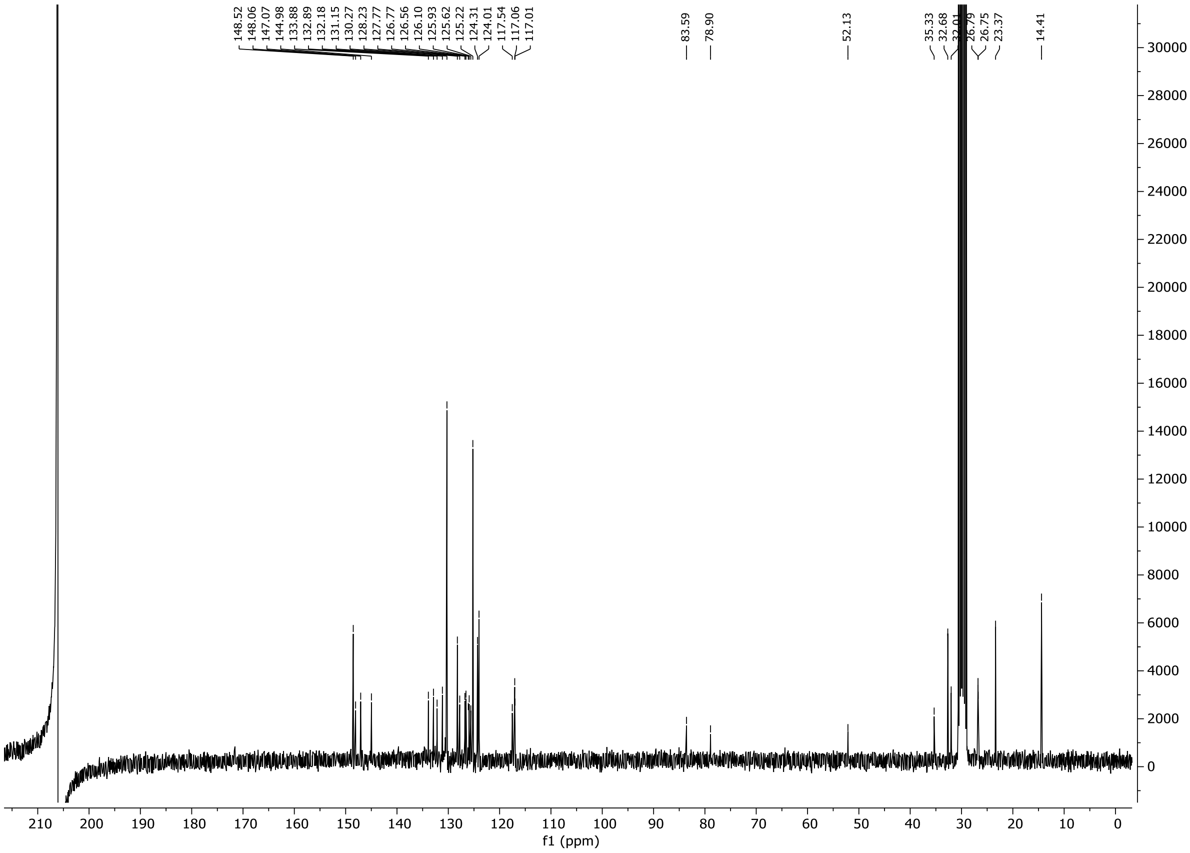

**Figure S208.** $^{13}C$ NMR spectrum of compound **3u** (75 MHz, acetone-d$_6$, *T* = 293 K).

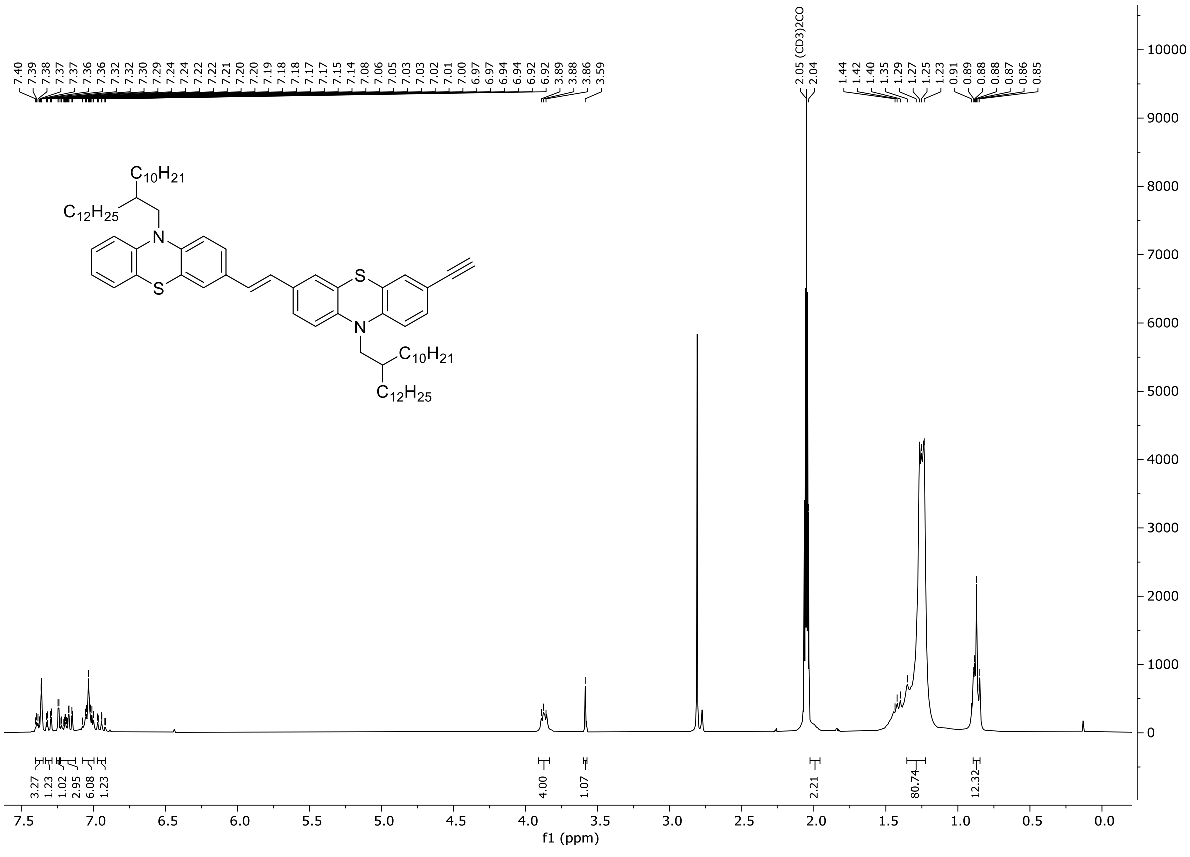

**Figure S209.** $^{1}H$ NMR spectrum of compound **3v** (300 MHz, acetone-d$_6$, *T* = 293 K).

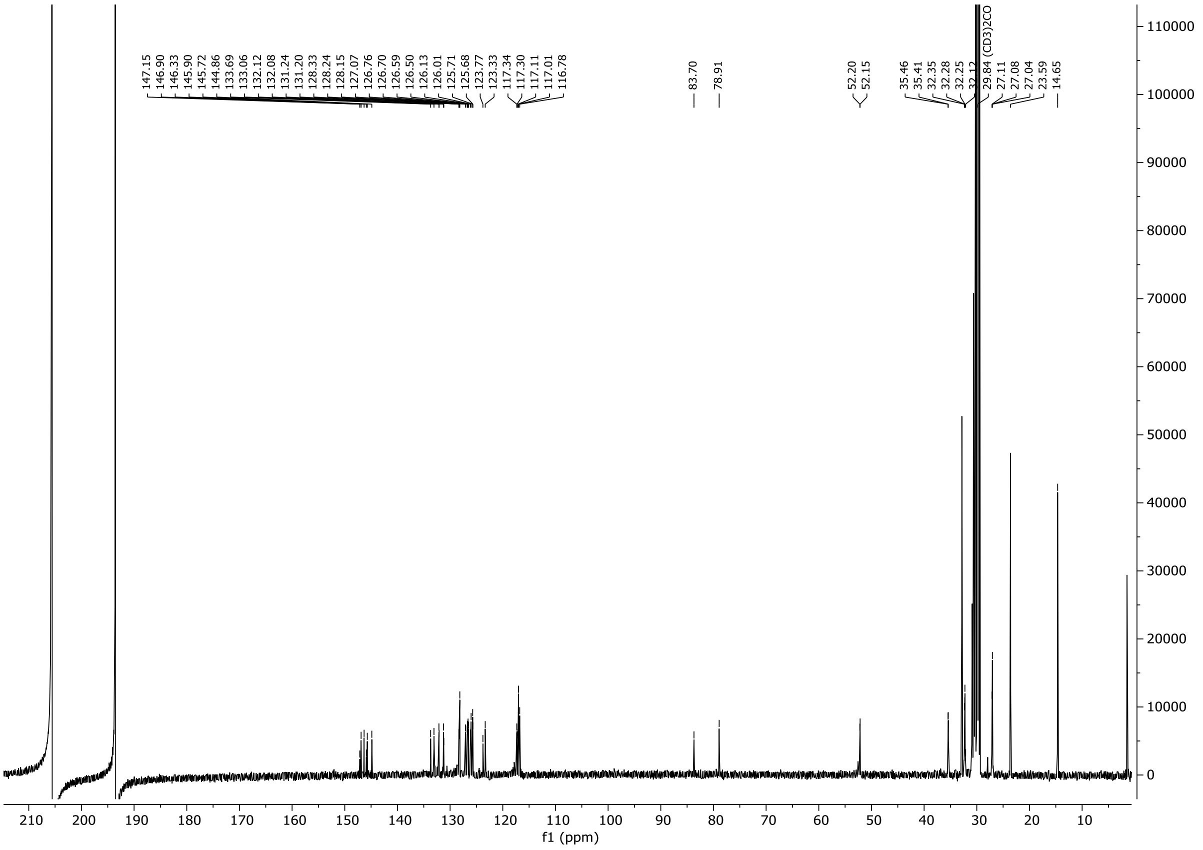


**Figure S210.** $^{13}C$ NMR spectrum of compound **3v** (75 MHz, acetone-$d_6$, $T$ = 293 K).

# 7.0 References


[1] M. Stephan, B. Stute, E. von Lieres and T. J. J. Müller, *Eur. J. Org. Chem.* ***2022***, *2022*.

[2] B. K. Börüsah and T. J. J. Müller, *Organics* **2022**, *3* 502-506.

[3] T. Meyer, D. Ogermann, A. Pankrath, K. Kleinermanns and T. J. J. Müller, *J. Org. Chem.* **2012**, *77*.
